# Supplementary material for: Umpolung reactivity of strained C–C σ-bonds without transition-metal catalysis
Source: Nat Commun. 2024 Apr 2;15:2833. doi: 10.1038/s41467-024-47169-9 (PMC10987681; doi:10.1038/s41467-024-47169-9)
Supplement: Supplementary file 1 — Supplementary Information [file 41467_2024_47169_MOESM1_ESM.pdf]

Supplementary Information

Umpolung Reactivity of Strained C–C σ-Bonds without Transition-metal Catalysis

Dachang Bai,*[1,3] Xiuli Guo,[1] Xinghua Wang,[2] Wenjie Xu,[1] Ruoshi Cheng,[1] Donghui Wei,[2] Yu Lan,[2] Junbiao Chang *[1]

[1] State Key Laboratory of Antiviral Drugs, NMPA Key Laboratory for Research and Evaluation of Innovative Drug, Key Laboratory of Green Chemical Media and Reactions, Ministry of Education, School of Chemistry and Chemical Engineering, Henan Normal University, Pingyuan Laboratory, Xinxiang 453007, China

[2] College of Chemistry and Institute of Green Catalysis, Zhengzhou University, Zhengzhou, Henan, China

[3] State Key Laboratory of Organometallic Chemistry, Shanghai Institute of Organic Chemistry, Chinese Academy of Sciences, Shanghai 200032, P R China

*Corresponding Author(s): baidachang@htu.edu.cn; changjunbiao@zzu.edu.cn

Table of Contents

1. Supplementary Methods …..………………………………………………………..……..……....S3

1.1. General Information …..…………………………..……..…………..……..……....S3

1.2. General Synthetic Procedure of Starting Materials …..…………………..…..……....S3

2. Supplementary Discussion ……………..………………..……………………..…...………...S13

2.1. General procedure for the reaction of BCBs with enones ……………………….......S13

2.2 Optimization of reaction conditions …..…………………………………..………....S14

2.3. Gram-Scale Synthesis and Derivatization Reactions …..……...……………………....S44

2.3.1 Scale-up reaction …..…………………………………………………………....S44

2.3.2 Derivatization Reactions …..……………………………………………………....S45

2.4. Mechanistic Studies …..…………………………………………..………...…....S60

2.4.1 Starting Material Synthesis ..…………………………………….……………....S60

2.4.2 Deuterium experiments …..…………………………………………...……....S62

2.4.3 Reaction with radical inhibitor …..…………………………………………….....S63

2.4.4 EPR experiments ..………………………………………..………….....…....S75

2.5 Kinetic Studies …..…………………………………….……………….....……....S78

2.6 Computational Studies …..………………………………….………………..……....S84

3. Supplementary Tables and Figures ………….………....…..………......…………………...…....S97

3.1. X-Ray Crystallographic Data ……………………………………………..……....S97

3.2. NMR Spectra …..………………………….……..……………………………...…....S99

4. Supplementary References ……………..………………………………….....…………......S279

1. Supplementary Methods

1.1. General Information

All chemicals were obtained from commercial sources and were used as received unless otherwise noted. All the reactions were carried out under argon atmosphere in a argon-filled glove box. The ^{1}H NMR spectra were recorded on a 400 MHz or 600 MHz NMR spectrometer. The ^{13}C NMR spectra were recorded at 100 MHz or 150 MHz. The ^{19}F NMR spectra were recorded at 377 MHz or 565 MHz. Chemical shifts were expressed in parts per million (δ) downfield from the internal standard tetramethylsilane, and were reported as s (singlet), d (doublet), t (triplet), dd (doublet of doublet), dt (doublet of triplet), m (multiplet), brs (broad singlet), etc. The residual solvent signals were used as references and the chemical shifts were converted to the TMS scale. High resolution mass spectra were obtained on an Agilent Q-TOF 6540 spectrometer. Column chromatography was performed on silica gel (300-400 mesh). Thin layer chromatography was performed on pre-coated glass back plates and visualized with UV light at 254 nm. Flash column chromatography was performed on silica gel. enones[1] and BCBs[2] were prepared according to literature reports.

1.2. General Synthetic Procedures of Starting Materials

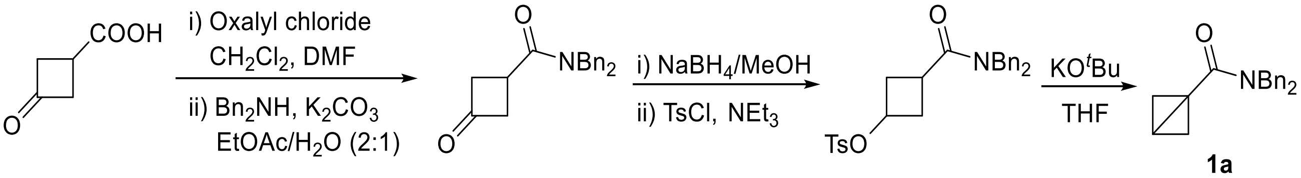

1a

1st Step: Following a literature procedure: In a round-bottom flask 3-oxocyclobutane-1-carboxylic acid (3.4 g, 30.0 mmol, 1.0 equiv) was dissolved in CH_2Cl_2 (120 mL) and DMF (1.0 mL) was added. The solution was cooled to 0 ℃ and subsequently oxalyl chloride (7.6 g, 60.0 mmol, 2.0 equiv) was added dropwise. The reaction was stirred for 2 h at 0 ℃ and was then allowed to warm up to room temperature. After stirring for 1 h at room temperature, the solvent was removed under reduced pressure to yield the crude acid chloride which was used without further purification.

To a round-bottom flask were added dibenzylamine (6.1 g, 36.0 mmol, 1.2 equiv), K_2CO_3 (8.3 g, 60.0 mmol, 2.0 equiv) and a mixture of EtOAc/H_2O (2:1, 100 mL). The mixture was cooled to 0 ℃, and subsequently the crude acid chloride was added dropwise (the flask containing acid chloride was

rinsed with EtOAc to make sure everything was transfered). Then, the reaction was stirred at room temperature overnight. The layers were separated and the aq. layer was extracted with EtOAc. The combined org. layers were dried over Na_2SO_4 and concentrated under reduced pressure. Purification by silica gel column chromatography gave the desired product (7.6 g, 87% yield).

2nd Step: Following a literature procedure: In a round-bottom flask amide (7.6 g, 26.1 mmol, 1.0 equiv) was dissolved in MeOH (26.1 mL), and the mixture was cooled to 0 ℃. $NaBH_4$ (1.5 g, 39.1 mmol, 1.5 equiv) was added portionwise (attention: gas evolution!) until full conversion (by TLC) of the starting material. The reaction was quenched with water, and the aq. layer was extracted with CH_2Cl_2. The combined org. layers were dried over Na_2SO_4 and removing the solvent in vacuo gave the crude product which was used in the next step without further purification.

To an oven-dried Schlenk flask equipped with a Teflon-coated magnetic stir bar was added the crude alcohol and the flask was evacuated and backfilled with argon three times. Subsequently, CH_2Cl_2 (26.1 mL) was added, and the solution was cooled to 0 ℃. TsCl (6.5 g, 33.9 mmol, 1.3 equiv) and NEt_3 (3.4 g, 33.9 mmol, 1.3 equiv) were added successively, and the reaction was allowed to warm up to room temperature, monitored by TLC, the reaction was quenched with water. The org. layer was separated, washed with brine, dried over Na_2SO_4, and concentrated in vacuo. Purification by silica gel column chromatography gave the desired product (8.0 g, 71% yield).

3rd Step: Following a literature procedure: To an oven-dried Schlenk tube equipped with a Teflon-coated magnetic stir bar was added the protected alcohol (8.0 g, 18.5 mmol, 1.0 equiv), and the flask was evacuated and backfilled with argon three times. Subsequently, dry THF (60 mL) was added and the solution was cooled to 0 ℃. KO^tBu (20.3 mL, 1 M in THF, 20.3 mmol, 1.1 equiv) was added dropwise to the reaction flask at 0 ℃. After stirring for 1 h, the reaction was quenched with sat. aq. NH_4Cl, and the org. layer was extracted with EtOAc. The combined org. layers were dried over Na_2SO_4, and concentrated under reduced pressure. Purification by silica gel column chromatography gave the desired product **1a** (3.1 g, 61% yield). ^1H NMR (400 MHz, $CDCl_3$) δ 7.34 – 7.31 (m, 6H), 7.25 – 7.20 (m, 4H), 4.82 (s, 2H), 4.58 (s, 2H), 2.27 (d, J = 3.4 Hz, 2H), 2.12 – 2.09 (m, 1H), 1.11 (d, J = 2.4 Hz, 2H). ^{13}C NMR (151 MHz, $CDCl_3$) δ 172.2, 137.3, 129.0, 128.7, 128.5, 127.6, 126.8, 50.7, 47.9, 37.1, 13.6, 8.4.

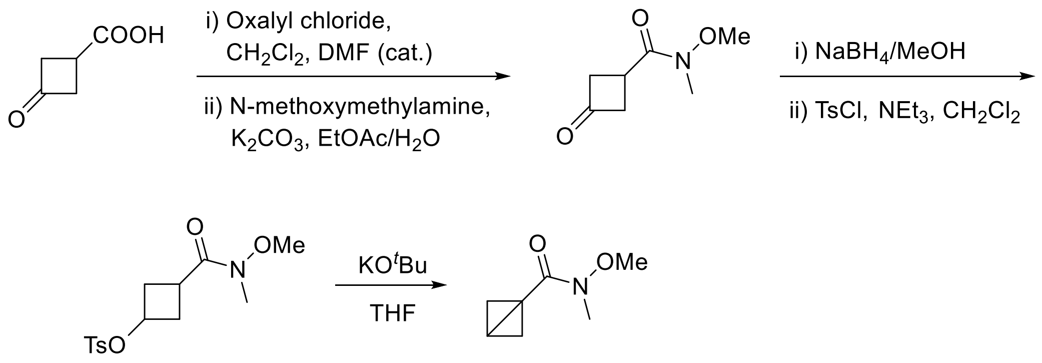

1st Step: In a round-bottom flask 3-oxocyclobutane-1-carboxylic acid (3.4 g, 30.0 mmol, 1.0 equiv) was dissolved in CH_2Cl_2 (120 mL) and DMF (1.0 mL) was added. The solution was cooled to 0 ℃ and subsequently oxalyl chloride (7.6 g, 60.0 mmol, 2.0 equiv) was added dropwise. The reaction was stirred for 2 h at 0 ℃ and was then allowed to warm up to room temperature. After stirring for 1 h at room temperature, the solvent was removed under reduced pressure to yield the crude acid chloride which was used without further purification.

To a round-bottom flask were added the respective N-methoxymethylamine (3.5 g, 36.0 mmol, 1.2 equiv), K_2CO_3 (8.3 g, 60.0 mmol, 2.0 equiv) and a mixture of EtOAc/H_2O (2:1, 100 mL). The mixture was cooled to 0 ℃, and subsequently the crude acid chloride was added dropwise. Then, the reaction was stirred at room temperature overnight. The layers were separated and the aq. layer was extracted with EtOAc. The combined org. layers were dried over Na_2SO_4 and concentrated under reduced pressure. Purification by silica gel column chromatography gave the desired product (2.8 g, 59% yield).

2nd Step: Following a literature procedure: In a round-bottom flask amide (2.8 g, 17.6 mmol, 1.0 equiv) was dissolved in MeOH (17.6 mL), and the mixture was cooled to 0 ℃. $NaBH_4$ (1.0 g, 26.4 mmol, 1.5 equiv) was added portionwise (attention: gas evolution!) until full conversion (by TLC) of the starting material. The reaction was quenched with water, and the aq. layer was extracted with CH_2Cl_2. The combined org. layers were dried over Na_2SO_4 and removing the solvent in vacuo gave the crude product which was used in the next step without further purification.

To an oven-dried Schlenk flask equipped with a Teflon-coated magnetic stir bar was added the crude alcohol and the flask was evacuated and backfilled with argon three times. Subsequently, CH_2Cl_2 (18 mL) was added, and the solution was cooled to 0 ℃. TsCl (4.4 g, 22.8 mmol, 1.3 equiv) and NEt_3 (2.3 g, 22.8 mmol, 1.3 equiv) were added successively, and the reaction was allowed to warm up to

room temperature. monitored by TLC, the reaction was quenched with water. The org. layer was separated, washed with brine, dried over Na_2SO_4, and concentrated in vacuo. Purification by silica gel column chromatography gave the desired product (1.4 g, 26% yield).

3rd Step: Following a literature procedure: To an oven-dried Schlenk tube equipped with a Teflon-coated magnetic stir bar was added the protected alcohol (1.4 g, 4.5 mmol, 1.0 equiv), and the flask was evacuated and backfilled with argon three times. Subsequently, dry THF (23 mL) was added and the solution was cooled to 0 ℃. KO^{t}Bu (5.0 mL, 1 M in THF, 5.0 mmol, 1.1 equiv) was added dropwise to the reaction flask at 0 ℃. After stirring for 1 h, the reaction was quenched with sat. aq. NH_4Cl, and the org. layer was extracted with EtOAc. The combined org. layers were dried over Na_2SO_4, and concentrated under reduced pressure. Purification by silica gel column chromatography gave the desired product Weinreb amide attached BCBs **1s** (0.5 g, 83% yield), which was used quickly for the 1, 4-conjugate addition.

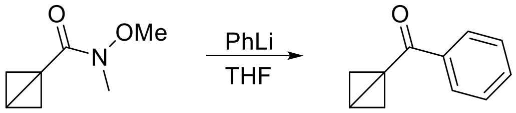

Following a literature procedure:[2] To an oven-dried Schlenk tube equipped with Teflon-coated magnetic stir bar were added bicyclo[1.1.0]butane-1-carboxamide (5.0 mmol, 1.0 equiv) and dry THF (30 mL). The solution was cooled to −78 °C, the PhLi (5.0 mL, 1M in THF, 5.0 mmol, 1.0 equiv) was added dropwise, and stirred for 1 h at this temperature. The reaction was then quenched by addition of sat. aq. $NaHCO_3$, and the mixture was diluted with EtOAc. The layers were separated, and the aq. layer was extracted with EtOAc. The combined org. layers were dried over Na_2SO_4 and concentrated under reduced pressure. Purification by silica gel column chromatography (10% EtOAc in hexane) gave the product. The solvent is removed under reduced pressure to give the ketone attached BCBs **1x** (263 mg, 33% yield) and was used quickly for the 1, 4-conjugate addition.

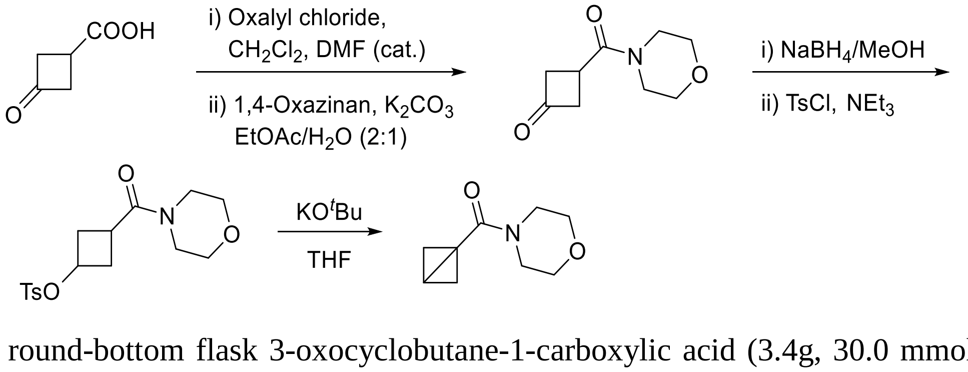

1st Step: In a round-bottom flask 3-oxocyclobutane-1-carboxylic acid (3.4g, 30.0 mmol, 1.0 equiv)

was dissolved in CH_2Cl_2 (120 mL) and DMF (1.0 mL) was added. The solution was cooled to 0 ℃ and subsequently oxalyl chloride (7.6 g, 60.0 mmol, 2.0 equiv) was added dropwise. The reaction was stirred for 2 h at 0 ℃ and was then allowed to warm up to room temperature. After stirring for 1 h at room temperature, the solvent was removed under reduced pressure to yield the crude acid chloride which was used without further purification.

To a round-bottom flask were added the respective 1, 4-Oxazinan (3.1 g, 36.0 mmol, 1.2 equiv), K_2CO_3 (8.3g, 60.0 mmol, 2.0 equiv) and a mixture of EtOAc/H_2O (2:1, 100 mL). The mixture was cooled to 0 ℃, and subsequently the crude acid chloride was added dropwise (the flask containing acid chloride was rinsed with EtOAc to make sure everything was transfered). Then, the reaction was stirred at room temperature overnight. The layers were separated and the aq. layer was extracted with EtOAc. The combined org. layers were dried over Na_2SO_4 and concentrated under reduced pressure. Purification by silica gel column chromatography gave the desired product (1.73 g, 32% yield).

2nd Step: In a round-bottom flask amide (1.73 g, 9.5 mmol, 1.0 equiv) was dissolved in MeOH (9.5 mL), and the mixture was cooled to 0 ℃. $NaBH_4$ (0.5 g, 14.3 mmol, 1.5 equiv) was added portionwise (attention: gas evolution) until full conversion (by TLC) of the starting material. The reaction was quenched with water, and the aq. layer was extracted with CH_2Cl_2. The combined org. layers were dried over Na_2SO_4 and removing the solvent in vacuo gave the crude product which was used in the next step without further purification.

To an oven-dried Schlenk flask equipped with a Teflon-coated magnetic stir bar was added the crude alcohol and the flask was evacuated and backfilled with argon three times. Subsequently, CH_2Cl_2 (10 mL) was added, and the solution was cooled to 0 ℃. TsCl (2.4 g, 12.4 mmol, 1.3 equiv) and NEt_3 (1.3g, 12.4 mmol, 1.3 equiv) were added successively, and the reaction was allowed to warm up to room temperature. monitored by TLC, the reaction was quenched with water. The org. layer was separated, washed with brine, dried over Na_2SO_4, and concentrated in vacuo. Purification by silica gel column chromatography gave the desired product (2.0 g, 62% yield).

3rd Step: To an oven-dried Schlenk tube equipped with a Teflon-coated magnetic stir bar was added the protected alcohol (2.0 g, 5.9 mmol, 1.0 equiv), and the flask was evacuated and backfilled with argon three times. Subsequently, dry THF (20 mL) was added and the solution was cooled to 0 ℃. KO^{t}Bu (6.5 mL, 1 M in THF, 6.5 mmol, 1.1 equiv) was added dropwise to the reaction flask at 0 ℃.

After stirring for 1 h, the reaction was quenched with sat. aq. NH_4Cl, and the org. layer was extracted with EtOAc. The combined org. layers were dried over Na_2SO_4, and concentrated under reduced pressure. Purification by silica gel column chromatography gave the desired product morpholine amide attached BCBs **1t** (0.7 g, 71% yield). ^{1}H NMR (400 MHz, $CDCl_3$) δ 3.79 – 3.53 (m, 8H), 2.17 – 2.16 (m, 2H), 1.96 – 1.91 (m, 1H), 1.15 – 1.08 (m, 2H). ^{13}C NMR (101 MHz, $CDCl_3$) δ 170.5, 66.9, 47.3, 42.8, 37.1, 13.9, 7.6.

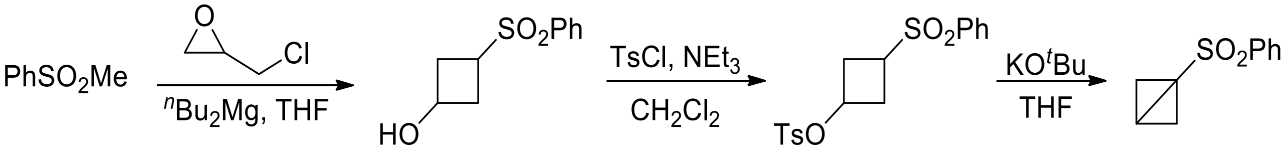

1st Step: Following a literature procedure:[2] An oven-dried microwave vial equipped with a magnetic stirbar was charged with the methylsulfone (5.47g, 35 mmol, 1.0 equiv), capped and flushed with Ar, anhydrous THF (120 mL) was added and the resulting solution was cooled to -78 °C. A solution of n-Bu_2Mg (35 mL (1 M in hexanes), 35 mmol, 1.0 equiv) was slowly added and the reaction was stirred for 30 min at -78 °C. To the resulting mixture was added epichlorohydrin (3.24 g, 35 mmol, 1.0 equiv) via syringe, the solution was warmed to room temperature and stirred until the starting material was consumed as determined by TLC analysis. The reaction was quenched with water, and extracted with CH_2Cl_2. The combined org. layers were dried over Na_2SO_4, and concentrated in vacuo. Purification by silica gel column chromatography gave the desired product (5.79 g, 78% yield).

2nd Step: To an oven-dried Schlenk flask equipped with a Teflon-coated magnetic stir bar was added the crude alcohol and the flask was evacuated and backfilled with argon three times. Subsequently, CH_2Cl_2 (28 mL) was added, and the solution was cooled to 0 ℃. TsCl (6.77 g, 35.5 mmol, 1.3 equiv) and NEt_3 (3.59 g, 35.5 mmol, 1.3 equiv) were added successively, and the reaction was allowed to warm up to room temperature. Upon full conversion (by TLC); the reaction was quenched with water. The org. layer was separated, washed with brine, dried over Na_2SO_4, and concentrated in vacuo. Purification by silica gel column chromatography gave the desired product (8.81 g, 88% yield).

3rd Step: To an oven-dried Schlenk tube equipped with a Teflon-coated magnetic stir bar was added the protected alcohol (8.81 g, 24.1 mmol, 1.0 equiv), and the flask evacuated and backfilled with argon three times. Subsequently, dry THF (60 mL) was added and the solution was cooled to 0 ℃. KO^{t}Bu (26.5 mL (1 M in THF), 26.5 mmol, 1.1 equiv) was added dropwise to the reaction flask at 0 ℃. After stirring for 1 h, the reaction was quenched with sat. aq. NH_4Cl, and the org. layer was

extracted with EtOAc. The combined org. layers were dried over Na_2SO_4, and concentrated under reduced pressure. Purification by silica gel column chromatography gave the desired product sulfone attached BCBs **1u** (4.29 g, 92% yield). ^{1}H NMR (400 MHz, $CDCl_3$) δ 7.96 – 7.93 (m, 2H), 7.65 – 7.62 (m, 1H), 7.58 – 7.54 (m, 2H), 2.58 – 2.55 (m, 1H), 2.52 – 2.51 (m, 2H), 1.39 – 1.38 (m, 2H). ^{13}C NMR (101 MHz, $CDCl_3$) δ 142.1, 133.1, 129.3, 127.2, 38.4, 23.2, 12.7.

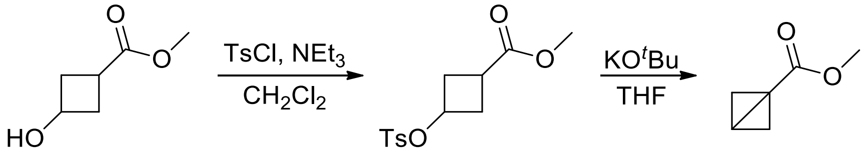

1st Step: The alcohol (2.6 g, 20 mmol, 1.0 equiv) was dissolved in dry DCM (20 mL), cooled to 0 ºC and consecutively TsCl (5.0 g, 26 mmol, 1.3 equiv.) and NEt_3 (2.6 g, 26 mmol, 1.3 equiv.) were added. The reaction mixture was warmed to room temperature and stirred under Ar atmosphere, until TLC indicated full conversion of the alcohol. Subsequently, the reaction mixture was washed with brine. The organic layer was dried over Na_2SO_4, filtrated and concentrated in vacuo. The crude tosylate was purified by column chromatography (2.8 g, 49% yield).

2nd Step: The solution of tosylate (2.8 g, 9.8 mmol, 1.0 equiv.) in dry THF (40 mL) was cooled to 0 ºC, and KO*t*Bu (10.8 mL, 1 M THF solution, 10.8 mmol, 1.0 equiv.) was added dropwise under Ar atmosphere. The reaction mixture was stirred for 15 min. Subsequently, the reaction was quenched with saturated NH_4Cl solution and extracted with DCM. Combined organic fractions were dried over Na_2SO_4, filtrated and concentrated in vacuo. The crude ester attached bicycle[1.1.0]butane was purified by column chromatography **1v** (300 mg, 27% yield). The solvent is removed under reduced pressure and was used quickly for the 1, 4-conjugate addition.

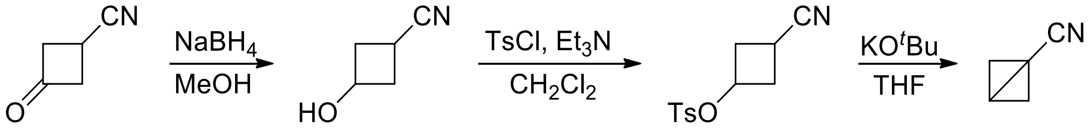

1st Step: The ketone (2.9 g, 30 mmol, 1.0 equiv) was dissolved in MeOH (30 mL), cooled to 0 ºC and $NaBH_4$ (1.7 g, 45 mmol, 1.5 equiv.) was carefully added. The reaction mixture was stirred for 30 min at 0 ºC. After this time, the reaction was quenched with water, and extracted with DCM. Combined organic fractions were dried over Na_2SO_4, filtrated and concentrated in vacuo.

2nd Step: The crude alcohol was redissolved in dry DCM (30 mL), cooled to 0 ºC and consecutively TsCl (8.6g, 45 mmol, 1.3 equiv.) and NEt_3 (4.5 g, 45 mmol, 1.3 equiv.) were added. The reaction

mixture was warmed to rt and stirred under Ar atmosphere, until TLC indicated full conversion of the alcohol. Subsequently, the reaction mixture was washed with water and brine. The organic layer was dried over Na_2SO_4, filtrated and concentrated in vacuo. The crude tosylate was purified by column chromatography (3.8 g, 50% yield).

3rd Step: The solution of tosylate (3.8 g, 15 mmol, 1 equiv.) in dry THF (60 mL) was cooled to 0 ºC, and KO^{t}Bu (16.5 mL, 1 M THF solution, 16.5 mmol, 1 equiv.) was added dropwise under Ar atmosphere. The reaction mixture was stirred for 1 h. Subsequently, the reaction was quenched with saturated NH_4Cl solution and extracted with DCM. Combined organic fractions were dried over Na_2SO_4, filtrated and concentrated in vacuo. The crude nitrile attached bicycle[1.1.0]butane was purified by column chromatography **1w** (233 mg, 28% yield). The solvent is removed under reduced pressure and was used quickly for the 1, 4-conjugate addition.

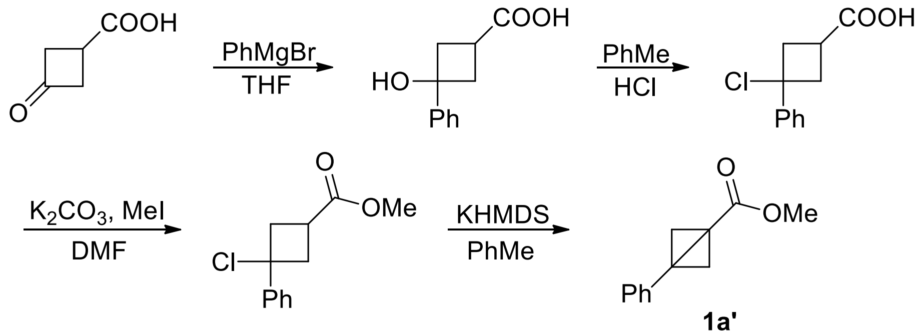

1a'

An oven-dried round bottom flask equipped with a stir bar was cooled under vacuum. After backfilled with Ar and capped with a septum, 3-oxocyclobutanecarboxylic acid (2.3 g, 20.0 mmol, 1.0 eq.) and dry THF (80 mL) were added. The reaction was cooled to 0 ^{o}C in an ice/water bath and PhMgBr (48 mL, 1.0 M in ether, 48 mmol, 2.4 eq.) was added to the solution. The ice/water bath was then removed and the reaction was stirred for 3 h at room temperature before quenched with saturated NH_4Cl solution. The aqueous layer was extracted with EtOAc. The combined organic layers were washed with brine, dried over anhydrous Na_2SO_4, filtered and concentrated by rotary evaporation. The crude acid was directly used in next reaction without further purification.

A round bottom flask equipped with a magnetic stir bar was charged with above crude acid (assuming 20 mmol, 1.0 eq.), concentrated HCl solution (40 mL) and PhMe (40 mL). The flask was capped with a septum. The reaction was vigorously stirred for 4 h at room temperature. The aqueous layer was extracted with EtOAc. The combined organic layers were washed with brine, dried over

anhydrous Na_2SO_4, filtered and concentrated by rotary evaporation. The crude acid was directly used in next reaction without further purification.

A round bottom flask equipped with a magnetic stir bar was charged with above crude acid (assuming 20 mmol, 1.0 eq.) and DMF (80 mL). K_2CO_3 (5.5 g, 40.0 mmol, 2.00 eq.) and MeI (1.90 mL, 30.0 mmol, 1.50 eq.) was added sequentially to the solution. The flask was capped with a septum. The reaction was stirred at room temperature for 12 h. The solution was then diluted with H_2O and extracted with EtOAc. The combined organic layers were washed with brine, dried over anhydrous Na_2SO_4, filtered and concentrated by rotary evaporation. The residue was purified by FCC (Hex : EtOAc = 9:1) to afford ester.

To an oven-dried Schlenk tube equipped with a Teflon-coated magnetic stir bar was added the ester (assuming 20 mmol, 1.0 eq.), and the flask evacuated and backfilled with argon three times. Subsequently, PhMe (60 mL) was added and the solution was cooled to 0 ℃. KHMDS (24.0 mL, 1.0 M in THF, 24.0 mmol, 1.2 eq.) was added dropwise to the reaction flask at 0 ℃. The reaction was vigorously stirred for 4 h at room temperature. The aqueous layer was extracted with EtOAc. The combined organic layers were washed with brine, dried over anhydrous Na_2SO_4, filtered and concentrated by rotary evaporation. Purification by silica gel column chromatography gave the desired product **1a'** (2.7 g, 72% yield). ^{1}H NMR (600 MHz, $CDCl_3$) δ 7.32 – 7.29 (m, 4H), 7.25 – 7.22 (m, 1H), 3.48 (s, 3H), 2.93 (s, 2H), 1.60 (s, 2H). ^{13}C NMR (151 MHz, $CDCl_3$) δ 170.2, 133.7, 128.6, 127.1, 126.1, 51.9, 35. 9, 33.1, 23.4.

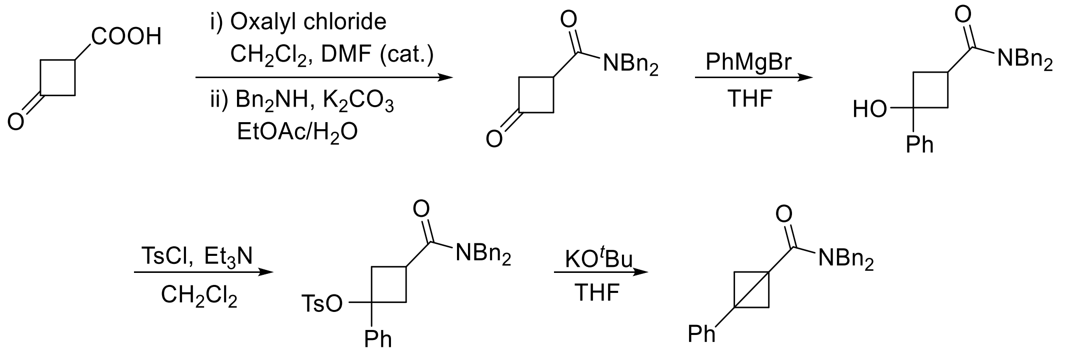

1st Step: Following a literature procedure: In a round-bottom flask 3-oxocyclobutane-1-carboxylic acid (1.7g, 15.0 mmol, 1.0 equiv) was dissolved in CH_2Cl_2 (60 mL) and DMF (0.5 mL) was added. The solution was cooled to 0 ℃ and subsequently oxalyl chloride (3.8 g, 30 mmol, 2.0 equiv) was added dropwise. The reaction was stirred for 2 h at 0 ℃ and was then allowed to warm up to room

temperature. After stirring for 1 h at room temperature, the solvent was removed under reduced pressure to yield the crude acid chloride which was used without further purification.

To a round-bottom flask were added the respective amine hydrochloride (3.1 g, 18 mmol, 1.2 equiv), K_2CO_3 (4.2 g, 30 mmol, 2.0 equiv) and a mixture of EtOAc/H_2O (2:1, 50 mL). The mixture was cooled to 0 ℃, and subsequently the crude acid chloride was added dropwise (the flask containing acid chloride was rinsed with EtOAc to make sure everything was transfered). Then, the reaction was stirred at room temperature overnight. The layers were separated and the aq. layer was extracted with EtOAc. The combined org. layers were dried over Na_2SO_4 and concentrated under reduced pressure. Purification by silica gel column chromatography gave the desired product (4.4 g, 99% yield).

2nd Step: To an oven-dried Schlenk tube equipped with a Teflon-coated magnetic stir bar was added the aldehyde (4.4 g, 15 mmol, 1.0 equiv), and the flask evacuated and backfilled with argon three times. Subsequently, dry THF (30 mL) was added and the solution was cooled to 0 ℃. PhMgBr (22.5 mL, 1 M in THF, 22.5 mmol, 1.5 equiv) was added dropwise to the reaction flask at 0 ℃. After stirring for 1 h at 0 ℃, The reaction was quenched with water, and the aq. layer was extracted with CH_2Cl_2. The combined org. layers were dried over Na_2SO_4, and concentrated in vacuo.

To an oven-dried Schlenk flask equipped with a Teflon-coated magnetic stir bar was added the crude alcohol and the flask was evacuated and backfilled with argon three times. Subsequently, CH_2Cl_2 (15 mL) was added, and the solution was cooled to 0 ℃. TsCl (3.7 g, 19.5 mmol, 1.3 equiv) and NEt_3 (2.0 g, 19.5 mmol, 1.3 equiv) were added successively, and the reaction was allowed to warm up to room temperature. Monitored by TLC, the reaction was quenched with water. The org. layer was separated, washed with brine, dried over Na_2SO_4, and concentrated in vacuo. Purification by silica gel column chromatography gave the desired product (0.8 g, 10% yield).

3rd Step: To an oven-dried Schlenk tube equipped with a Teflon-coated magnetic stir bar was added the protected alcohol (0.8 g, 1.5 mmol, 1.0 equiv), and the flask evacuated and backfilled with argon three times. Subsequently, dry THF (7.5 mL) was added and the solution was cooled to 0 ℃. KO^{t}Bu (1.7 mL, 1 M in THF, 1.7 mmol, 1.1 equiv) was added dropwise to the reaction flask at 0 ℃. After stirring for 1 h, the reaction was quenched with sat. aq. NH_4Cl, and the org. layer was extracted with EtOAc. The combined org. layers were dried over Na_2SO_4, and concentrated under reduced pressure. Purification by silica gel column chromatography gave the desired β-phenyl amide attached BCBs

(349.2 mg, 65% yield). ^{1}H NMR (600 MHz, $CDCl_3$) δ 7.37 – 7.26 (m, 8H), 7.25 – 7.16 (m, 3H), 7.13 – 6.91 (m, 4H), 4.74 (s, 2H), 4.37 (s, 2H), 2.86 (s, 2H), 1.54 (s, 2H). ^{13}C NMR (151 MHz, $CDCl_3$) δ 169.1, 137.3, 133.9, 128.9, 128.5, 128.4, 127.6, 127.3, 126.7, 126.6, 50.6, 47.8, 36.7, 31.5, 22.2.

2. Supplementary Discussion

2.1. General procedure for the reaction of BCBs with enones：

General procedure **A**：Na_2SO_4 (25.0 mg, 0.17 mmol), **1a** (55.4 mg, 0.2 mmol, 2.0 equiv) and **2a** (13.2 mg, 0.1 mmol, 1.0 equiv) in DMSO (0.10 mL) were charged into a 10 mL pressure tube under argon. The reaction tube was then sealed and placed into a metal bath at 80 °C. After being stirred for 24 h, the reaction vessel was removed from the metal bath and cooled to ambient temperature. To the reaction mixture was added H_2O (4.0 mL), and the mixture was exacted with Et_2O. The organic layer was concentrated and purified by silica gel chromatography (PE: EA = 5:1) to give the indicated product **3a**.

General procedure **B**：Na_2SO_4 (25.0 mg, 0.17 mmol), **1a** (27.7 mg, 0.1 mmol, 1.0 equiv) and **2a** (52.8 mg, 0.4 mmol, 4.0 equiv) in DMSO (0.10 mL) were charged into a 10 mL pressure tube under argon. The reaction tube was then sealed and placed into a metal bath at 80 °C. After being stirred for 24 h, the reaction vessel was removed from the metal bath and cooled to ambient temperature. To the reaction mixture was added H_2O (4.0 mL), and the mixture was exacted with Et_2O. The organic layer was concentrated and purified by silica gel chromatography (PE: EA = 5:1) to give the indicated product **3a**.

General procedure **C**：Na_2SO_4 (25.0 mg, 0.17 mmol), **1a** (0.3 mmol, 3.0 equiv) and **2aa** (0.1 mmol, 1.0 equiv) in DMSO (0.10 mL) were charged into a pressure tube under argon. The reaction tube was then sealed and placed into a metal bath at 80 °C. After being stirred for 48 h, the reaction vial was removed from the metal bath and cooled to ambient temperature. To the reaction mixture was added H_2O (4.0 mL), and the mixture was exacted with Et_2O. The organic layer was concentrated and purified by silica gel chromatography (PE: EA = 5:1) to give the indicated product **4a**.

General procedure **D**：Na_2SO_4 (25.0 mg, 0.17 mmol), **1a** (0.2 mmol, 2.0 equiv) and **2aa** (0.1 mmol, 1.0 equiv) in DMSO (1.00 mL) were charged into a pressure tube under argon. The reaction tube was then sealed and placed into a metal bath at 130 °C. After being stirred for 48 h, the reaction vial was

removed from the metal bath and cooled to ambient temperature. To the reaction mixture was added H_2O (4.0 mL), and the mixture was exacted with Et_2O. The organic layer was concentrated and purified by silica gel chromatography (PE: EA = 5:1) to give the indicated product **5a**.

2.2 Optimization of reaction conditions

Supplementary Table 1, Optimization of reaction conditions of **1a** and **2a**. [a]

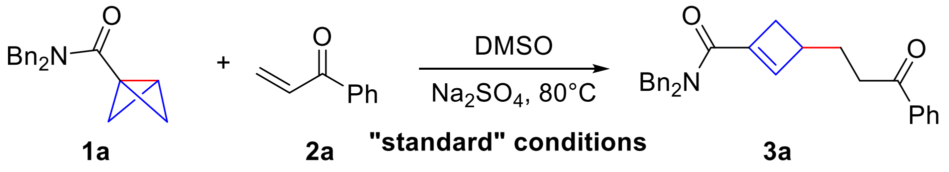

entry	Variation from the "standard" conditions	**3a**, yield (%)
1	none	91
2	no Na_2SO_4	30
3	4Å instead of Na_2SO_4	38
4	$MgSO_4$ instead of Na_2SO_4	82
5	1.0 eq Na_2SO_4 instead of 1.7 eq Na_2SO_4	64
6	DMA instead of DMSO	52
7	DMF instead of DMSO	52
8	DCE instead of DMSO	54
9	MeOH instead of DMSO	54
10	toluene instead of DMSO	42
11	dioxane instead of DMSO	58
12	THF instead of DMSO	42
13	50°C instead of 80°C	22
14	100°C instead of 80°C	4
15	0.2 mL DMSO instead of 0.1 mL DMSO	75
16	1.0 mL DMSO instead of 0.1 mL DMSO	38
17	1 eq **1a** instead of 2 eq **1a**	34
18	under the air	20
19[b]	4 eq **2a** was used	66
20[c]	6 eq **2a** was used	58

[a] Reaction conditions: **1a** (0.2 mmol), **2a** (0.1 mmol), Na_2SO_4 (0.17 mmol), 80°C in DMSO (0.1 mL) under argon, 24h, isolated yield. [b] **1a** (0.1 mmol), **2a** (0.4 mmol), Na_2SO_4 (0.17 mmol), 80°C in DMSO (0.1 mL). [c] **1a** (0.1 mmol), **2a** (0.6 mmol).

Supplementary Table 2, Optimization of reaction conditions of **1u** and **2a**. [a]

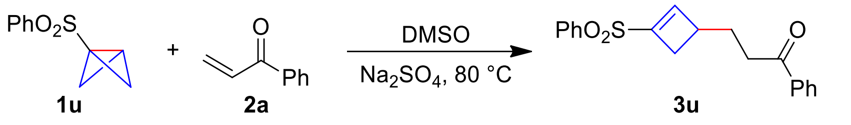

Entry	Variation from the "standard" condition[a]	**3u**, yield (%)
1	none	5
2	1.0 eq Na_2SO_4 instead of 1.7 eq Na_2SO_4	2
3	$MgSO_4$ instead of Na_2SO_4	6
4	toluene instead of DMSO	8
5	dioxane instead of DMSO	7
6	DMF instead of DMSO	NR
7	DMA instead of DMSO	2
8	DCE instead of DMSO	4
9	MeOH instead of DMSO	2
10	CH_3CN instead of DMSO	4
11	50 °C instead of 80 °C	NR
12	100 °C instead of 80 °C	8
13	0.05 mL DMSO instead of 0.1 mL DMSO	7
14[b]	5 eq **1u** used	12

[a] Reaction conditions A: **2a** (0.1 mmol), **1u** (0.2 mmol), Na_2SO_4 (0.17 mmol), 80 °C in DMSO (0.1 mL) under argon, 24 h, isolated yield. [b] Reaction conditions B: **2a** (0.5 mmol), **1u** (0.1 mmol).

Supplementary Table 3, Optimization of reaction conditions of **1xa** and **2a**. [a]

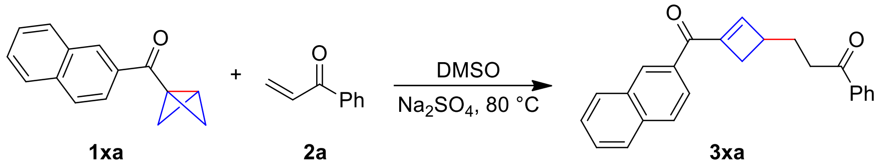

Entry	Variation from the "standard" condition[a]	**3xa**, yield (%)
1	none	19
2	1 eq Na_2SO_4 instead of 1.7 eq Na_2SO_4	18
3	$MgSO_4$ instead of Na_2SO_4	18
4	toluene instead of DMSO	18
5	dioxane instead of DMSO	16
6	DMA instead of DMSO	12
7	DCE instead of DMSO	20
8	MeOH instead of DMSO	NR
9	CH_3CN instead of DMSO	26
10	50 °C instead of 80 °C	11
11	100 °C instead of 80 °C	NR
12[b]	5 eq **2a** used	14
13[b]	50 °C instead of 80 °C	52
14[b,c]	no Na_2SO_4	50
15[b,c]	$MgSO_4$ instead of Na_2SO_4	59

[a] Reaction conditions A: **2a** (0.1 mmol), **1xa** (0.2 mmol), Na_2SO_4 (0.17 mmol), 80 °C in DMSO (0.1 mL) under argon, 24h, isolated yield. [b] Reaction conditions B: **2a** (0.5 mmol), **1xa** (0.1 mmol), Na_2SO_4 (0.17 mmol), neat without any solvent, 80 °C under argon, 48 h, isolated yield. [c] Reaction conditions C: 50 °C.

Supplementary Table 4, Optimization of reaction conditions of **1a** and **2aa**. [a]

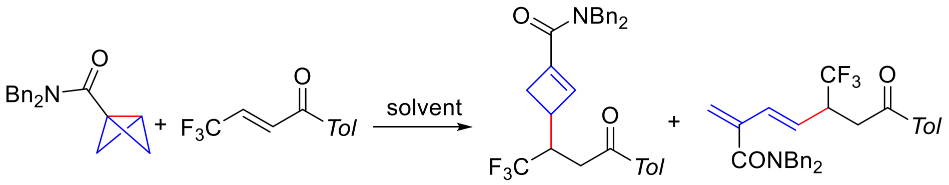

1a **2aa** **4a** **5a**

Entry	**1a**	Solvent	Additive	T (°C)	**4a**, yield (%)	dr of **4a**	**5a**, yield (%)
1	1eq	DMSO	Na_2SO_4	80	35	4.7:1	trace
2	2eq	DMSO	Na_2SO_4	80	57	5:1	1
3	2eq	DMSO	$MgSO_4$	80	56	5:1	--
4	2eq	DMSO	4Å	80	57	5:1	24
5[b]	2eq	DMSO	B_2Pin_2	80	53	4:1	--
6[b]	2eq	DMSO	$B(Ph)_3$	80	52	3.2:1	--
7[b]	2eq	DMSO	$Y(OTf)_3$	80	43	4.3:1	--
8[b]	2eq	DMSO	$Mg(OTf)_2$	80	44	5:1	--
9	2eq	DMSO	--	80	49	5:1	12
10	2eq	THF	Na_2SO_4	80	26	5:1	--
11	2eq	DCE	Na_2SO_4	80	27	3:1	--
12	2eq	CF_3CH_2OH	Na_2SO_4	80	33	4:1	--
13	2eq	^{t}BuOH	Na_2SO_4	80	38	4:1	--
14	2eq	DMA	Na_2SO_4	80	40	4:1	--
15	2eq	Toluene	Na_2SO_4	80	20	3.4:1	--
16	2eq	Dioxane	Na_2SO_4	80	25	3.2:1	--
17	2eq	CH_3CN	Na_2SO_4	80	29	3.5:1	--
18	2eq	DMF	Na_2SO_4	80	44	5:1	--
19	2eq	DMSO	Na_2SO_4	80	63	5:1	3
20[c]	2eq	DMSO	Na_2SO_4	80	64	5:1	trace
21[d].	2eq	DMSO	Na_2SO_4	80	76	4:1	--
22[e]	3eq	DMSO	Na_2SO_4	80	79	5:1	3
23[e]	2eq	DMSO	Na_2SO_4	100	54	5:1	3
24[d]	2eq	DMSO	Na_2SO_4	120	trace	trace	52
25[d]	2eq	DMSO	Na_2SO_4	130	--	--	96
26	2eq	DMSO	Na_2SO_4	130	--	--	95
27[f]	2eq	DMSO	Na_2SO_4	130	--	--	88
28[g]	2eq	DMSO	$MgSO_4$	130	--	--	83
29	2eq	DMSO	4Å	130	--	--	50
30	1eq	DMSO	Na_2SO_4	130	--	--	42

[a] Reactions conditions: **1a** (0.2 mmol), **2aa** (0.1 mmol), Na_2SO_4 (0.17 mmol) solvent (1.0 mL), 48 h, dr of **4a** was determined by ^{1}H NMR, under argon, isolated yield.[b]additive (10% mmol). [c]DMSO (0.5 mL).[d]DMSO (0.2 mL). [e]DMSO (0.1 mL).[f]Na_2SO_4(0.2 mmol).[g]Na_2SO_4(0.1 mmol).

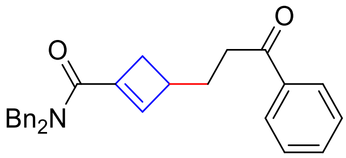

3a

3a, General procedure A (0.1 mmol alkene), yellow oil. 37.3 mg (91% yield). ^{1}H NMR (400 MHz, $CDCl_3$) δ 7.90 (d, *J* = 7.4 Hz, 2H), 7.53 – 7.50 (m, 1H), 7.43 – 7.39 (m, 2H), 7.36 – 7.28 (m, 5H), 7.25 – 7.22 (m, 3H), 7.16 – 7.14 (m, 2H), 6.45 (s, 1H), 4.62 – 4.52 (m, 4H), 3.00 – 2.97 (m, 1H), 2.96 – 2.92 (m, 2H), 2.80 – 2.75 (m, 1H), 2.43 – 2.40 (m, 1H), 1.98 – 1.84 (m, 2H). ^{13}C NMR (101 MHz, $CDCl_3$) δ 199.7, 164.5, 144.4, 139.4, 136.9, 136.8, 136.6, 133.0, 128.9, 128.6, 128.5, 128.0, 127.6, 127.4, 126.6, 50.1, 48.1, 39.1, 37.1, 36.3, 27.8. HRMS (ESI, m/z): calcd for $C_{28}H_{27}NNaO_2^+$ [M + Na] $^+$: 432.1934, found 432.1930.

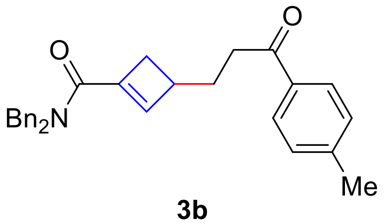

3b

3b, General procedure A (0.1 mmol alkene), yellow oil. 25.1 mg (59% yield). ^{1}H NMR (400 MHz, $CDCl_3$) δ 7.83 – 7.81 (m, 2H), 7.38 – 7.27 (m, 6H), 7.25 – 7.22 (m, 4H), 7.17 – 7.16 (m, 2H), 6.46 (s, 1H), 4.63 – 4.53 (m, 4H), 3.01 – 2.97 (m, 1H), 2.96 – 2.91 (m, 2H), 2.80 – 2.75 (m, 1H), 2.43 – 2.40 (m, 1H), 2.40 (s, 3H), 1.98 – 1.84 (m, 2H). ^{13}C NMR (101 MHz, $CDCl_3$) δ 199.6, 164.7, 144.7, 144.0, 139.5, 137.1, 136.8, 134.5, 129.4, 129.1, 128.7, 128.7, 128.3, 127.7, 127.6, 126.7, 50.2, 48.2, 39.3, 37.3, 36.3, 28.0, 21.8. HRMS (ESI, m/z): calcd for $C_{29}H_{29}NNaO_2^+$ [M + Na] $^+$: 446.2091, found 446.2084.

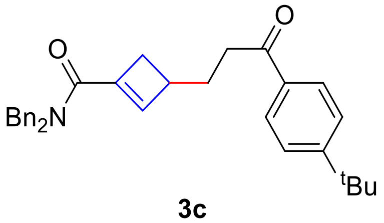

3c

3c, General procedure A (0.2 mmol alkene), yellow oil. 55.4 mg (60% yield). ^{1}H NMR (400 MHz, $CDCl_3$) δ 7.87 – 7.85 (m, 2H), 7.46 – 7.44 (m, 2H), 7.38 – 7.27 (m, 6H), 7.25 – 7.23 (m, 2H), 7.17 – 7.16 (m, 2H), 6.45 (d, *J* = 0.8 Hz, 1H), 4.63 – 4.53 (m, 4H), 3.01 – 2.97 (m, 1H), 2.96 – 2.92 (m, 2H), 2.80 – 2.75 (m, 1H), 2.41 (dd, *J* = 13.2, 1.2 Hz, 1H), 1.99 – 1.84 (m, 2H), 1.33 (s, 9H). ^{13}C

NMR (101 MHz, $CDCl_3$) δ 199.6, 164.7, 156.9, 144.7, 139.5, 137.1, 136.8, 134.4, 129.1, 128.7, 128.7, 128.1, 127.7, 127.6, 126.7, 125.7, 50.2, 48.2, 39.3, 37.3, 36.4, 35.2, 31.2, 28.1. HRMS (ESI, m/z): calcd for $C_{32}H_{35}NNaO_2^+$ [M + Na] $^+$: 488.2560, found 488.2553.

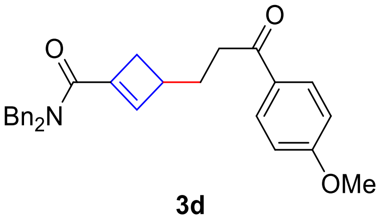

3d

3d, General procedure A (0.2 mmol alkene), yellow oil. 44.6 mg (51% yield). ^{1}H NMR (400 MHz, $CDCl_3$) δ 7.91 – 7.88 (m, 2H), 7.38 – 7.28 (m, 6H), 7.24 – 7.23 (m, 2H), 7.17 – 7.15 (m, 2H), 6.93 – 6.89 (m, 2H), 6.46 (s, 1H), 4.62 – 4.52 (m, 4H), 3.86 (s, 3H), 2.98 (dd, *J* = 13.2, 4.4 Hz, 1H), 2.91 (t, *J* = 7.2 Hz, 2H), 2.80 – 2.75 (m, 1H), 2.41 (dd, *J* = 13.2, 1.2 Hz, 1H), 1.96 – 1.85 (m, 2H). ^{13}C NMR (151 MHz, $CDCl_3$) δ 198.6, 164.7, 163.6, 144.8, 139.4, 137.0, 136.7, 130.4, 130.0, 129.1, 128.7, 128.6, 127.7, 127.6, 126.7, 113.8, 55.6, 50.2, 48.2, 39.4, 37.3, 36.1, 28.2. HRMS (ESI, m/z): calcd for $C_{29}H_{29}NNaO_3^+$ [M + Na] $^+$: 462.2040, found 462.2038.

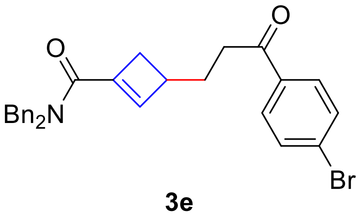

3e

3e, General procedure A (0.2 mmol alkene), yellow oil. 80.4 mg (83% yield). ^{1}H NMR (400 MHz, $CDCl_3$) δ 7.78 – 7.76 (m, 2H), 7.60 – 7.57 (m, 2H), 7.38 – 7.27 (m, 6H), 7.24 – 7.23 (m, 2H), 7.17 – 7.15 (m, 2H), 6.44 (s, 1H), 4.62 – 4.53 (m, 4H), 2.99 (dd, *J* = 13.2, 4.4 Hz, 1H), 2.92 (t, *J* = 7.2 Hz, 2H), 2.79 – 2.75 (m, 1H), 2.40 (dd, *J* = 13.2, 1.2 Hz, 1H), 2.98 – 1.84 (m, 2H). ^{13}C NMR (151 MHz, $CDCl_3$) δ 198.8, 164.6, 144.4, 139.6, 137.0, 136.7, 135.6, 132.1, 129.7, 129.1, 128.7, 128.7, 128.4, 127.8, 127.6, 126.7, 50.2, 48.2, 39.2, 37.2, 36.4, 27.8. HRMS (ESI, m/z): calcd for $C_{28}H_{26}BrNNaO_2^+$ [M + Na] $^+$: 510.1039, found 510.1027.

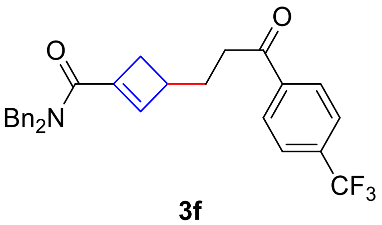

3f

3f, General procedure A (0.2 mmol alkene), yellow oil. 59.7 mg (63% yield). ^{1}H NMR (400 MHz, $CDCl_3$) δ 8.01 (d, *J* = 8.2 Hz, 2H), 7.71 (d, *J* = 8.2 Hz, 2H), 7.38 – 7.27 (m, 6H), 7.24 – 7.23 (m, 2H), 7.17 – 7.15 (m, 2H), 6.45 (d, *J* = 0.4 Hz, 1H), 4.62 – 4.53 (m, 4H), 3.02 – 2.96 (m, 3H), 2.81 – 2.76 (m, 1H), 2.42 (dd, *J* = 13.4, 1.4 Hz, 1H), 2.00 – 1.86 (m, 2H). ^{13}C NMR (101 MHz, $CDCl_3$) δ 198.7, 164.7, 144.6, 139.6, 139.5, 136.9, 136.6, 134.5 (q, *J* = 32.8 Hz), 129.1, 128.8, 128.7, 128.5, 127.8, 127.7, 126.7, 125.8 (q, *J* = 4.0 Hz), 123.7 (q, *J* = 273.0 Hz), 50.3, 48.3, 39.1, 37.2, 36.7, 27.6. ^{19}F NMR (377 MHz, $CDCl_3$) δ -63.10 (s, 3F). HRMS (ESI, m/z): calcd for $C_{29}H_{26}F_3NNaO_2^+$ [M + Na] $^+$: 500.1808, found 500.1805.

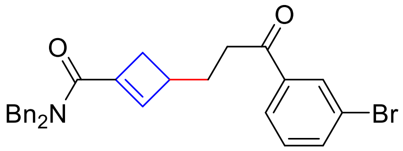

3g

3g, General procedure A (0.2 mmol alkene), yellow oil. 57.3 mg (59% yield). ^{1}H NMR (400 MHz, $CDCl_3$) δ 8.06-8.05 (m, 1H), 7.84 – 7.82 (m, 1H), 7.68 – 7.66 (m, 1H), 7.38 – 7.29 (m, 7H), 7.25 – 7.23 (m, 2H), 7.17 – 7.16 (m, 2H), 6.44 (s, 1H), 4.63 – 4.53 (m, 4H), 2.99 (dd, *J* = 13.2, 4.4 Hz, 1H), 2.92 (t, *J* = 7.2 Hz, 2H), 2.79 – 2.75 (m, 1H), 2.41 (dd, *J* = 13.2, 1.2 Hz, 1H), 1.98 – 1.84 (m, 2H). ^{13}C NMR (101 MHz, $CDCl_3$) δ 198.4, 164.6, 144.4, 139.6, 138.7, 137.0, 136.8, 136.0, 131.2, 130.3, 129.1, 128.7, 128.7, 127.7, 127.6, 126.7, 123.1, 50.2, 48.2, 39.1, 37.2, 36.5, 27.7. HRMS (ESI, m/z): calcd for $C_{28}H_{26}BrNNaO_2^+$ [M + Na] $^+$: 510.1039, found 510.1029.

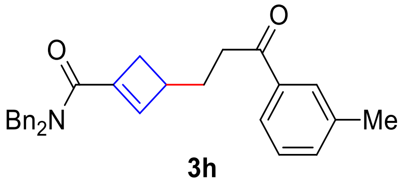

3h

3h, General procedure A (0.2 mmol alkene), yellow oil. 56.5 mg (67% yield). ^{1}H NMR (400 MHz, $CDCl_3$) δ 7.73 – 7.70 (m, 2H), 7.36 – 7.27 (m, 8H), 7.25 – 7.23 (m, 2H), 7.18 – 7.16 (m, 2H), 6.46 (s, 1H), 4.63 – 4.53 (m, 4H), 3.02 – 2.98 (m, 1H), 2.97 – 2.93 (m, 2H), 2.80 – 2.76 (m, 1H), 2.44 – 2.42 (m, 1H), 2.40 (s, 3H), 1.99 – 1.84 (m, 2H). ^{13}C NMR (151 MHz, $CDCl_3$) δ 200.1, 164.7, 144.7, 139.5, 138.5, 137.0, 137.0, 136.7, 133.9, 129.0, 128.7, 128.6, 128.6, 127.7, 127.6, 126.7, 125.4, 50.2, 48.2, 39.3, 37.2, 36.5, 27.9, 21.5. HRMS (ESI, m/z): calcd for $C_{29}H_{29}NNaO_2^+$ [M + Na] $^+$: 446.2091, found 446.2084.

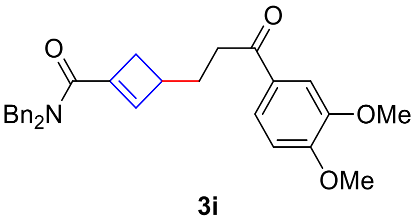

3i

3i, General procedure A (0.2 mmol alkene), yellow oil. 41.7 mg (44% yield). ^{1}H NMR (400 MHz, $CDCl_3$) δ 7.55 – 7.53 (m, 1H), 7.49 (d, *J* = 1.8 Hz, 1H), 7.37 – 7.29 (m, 6H), 7.24 – 7.22 (m, 2H), 7.17 – 7.15 (m, 2H), 6.86 (d, *J* = 8.4 Hz, 1H), 6.46 (s, 1H), 4.62 – 4.52 (m, 4H), 3.94 (s, 3H), 3.91 (s, 3H), 2.99 (dd, *J* = 13.2, 4.4 Hz, 1H), 2.92 (t, *J* = 7.4 Hz, 2H), 2.80 – 2.75 (m, 1H), 2.42 (dd, *J* = 13.2, 1.0 Hz, 1H), 1.98 – 1.84 (m, 2H). ^{13}C NMR (101 MHz, $CDCl_3$) δ 198.6, 164.7, 153.4, 149.2, 144.7, 139.5, 137.0, 136.8, 130.2, 129.0, 128.7, 128.6, 127.7, 127.6, 126.7, 122.8, 110.2, 110.1, 56.2, 56.1, 50.2, 48.1, 39.3, 37.3, 36.0, 28.3. HRMS (ESI, m/z): calcd for $C_{30}H_{31}NNaO_4^+$ [M + Na] $^+$: 492.2145, found 492.2146.

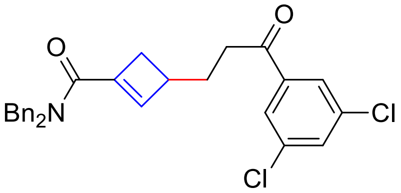

3j

3j, General procedure A (0.2 mmol alkene), yellow oil. 64.4 mg (67% yield). ^{1}H NMR (400 MHz, $CDCl_3$) δ 7.76 (d, *J* = 1.8 Hz, 2H), 7.53 (d, *J* = 1.8 Hz, 1H), 7.38 – 7.29 (m, 6H), 7.25 – 7.23 (m, 2H), 7.17 – 7.16 (m, 2H), 6.43 (s, 1H), 4.63 – 4.54 (m, 4H), 3.01 – 2.97 (m, 1H), 2.90 (t, *J* = 7.2 Hz, 2H), 2.79 – 2.74 (m, 1H), 2.40 (dd, *J* = 13.4, 1.2 Hz, 1H), 1.98 – 1.84 (m, 2H). ^{13}C NMR (151 MHz, $CDCl_3$) δ 197.2, 164.6, 144.2, 139.7, 139.3, 137.0, 136.7, 135.8, 132.8, 129.1, 128.7, 128.7, 127.8, 127.6, 126.7, 126.6, 50.2, 48.2, 39.0, 37.2, 36.6, 27.5. HRMS (ESI, m/z): calcd for $C_{28}H_{25}Cl_2NNaO_2^+$ [M + Na] $^+$: 500.1155, found 500.1139.

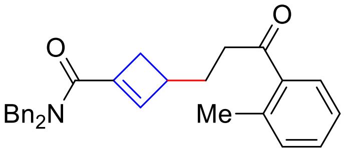

3k

3k, General procedure A (0.2 mmol alkene), yellow oil. 57.4 mg (68% yield). ^{1}H NMR (400 MHz, $CDCl_3$) δ 7.59 – 7.57 (m, 1H), 7.37 – 7.34 (m, 3H), 7.32 – 7.27 (m, 4H), 7.25 – 7.22 (m, 4H), 7.18 – 7.16 (m, 2H), 6.46 (s, 1H), 4.63 – 4.53 (m, 4H), 2.98 (dd, *J* = 13.2, 4.4 Hz, 1H), 2.88 (t, *J* = 7.4 Hz,

2H), 2.78 – 2.74 (m, 1H), 2.45 (s, 3H), 2.42 – 2.39 (m, 1H), 1.95 – 1.81 (m, 2H). ^{13}C NMR (151 MHz, $CDCl_3$) δ 199.6, 164.7, 144.7, 143.9, 139.5, 137.0, 136.8, 134.5, 129.4, 129.0, 128.7, 128.6, 128.3, 127.7, 127.6, 126.7, 50.2, 48.2, 39.3, 37.2, 36.3, 28.0, 21.7. HRMS (ESI, m/z): calcd for $C_{29}H_{29}NNaO_2^+$ [M + Na] $^+$: 446.2091, found 446.2086.

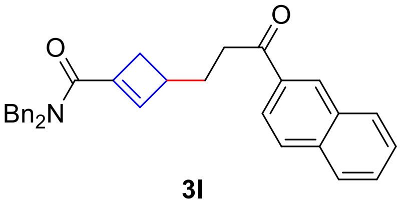

3l

3l, General procedure A (0.1 mmol alkene), yellow oil. 27.9 mg (61% yield). ^{1}H NMR (400 MHz, $CDCl_3$) δ 8.43 (s, 1H), 8.00 – 7.94 (m, 2H), 7.89 – 7.84 (m, 2H), 7.62 – 7.53 (m, 2H), 7.37 – 7.29 (m, 5H), 7.27 – 7.24 (m, 3H), 7.18 – 7.16 (m, 2H), 6.49 (s, 1H), 4.65 – 4.54 (m, 4H), 3.10 (t, J = 7.4 Hz, 2H), 3.03 (dd, J = 13.2, 4.4 Hz, 1H), 2.85 – 2.81 (m, 1H), 2.48 – 2.44 (m, 1H), 2.06 – 1.92 (m, 2H). ^{13}C NMR (151 MHz, $CDCl_3$) δ 199.6, 164.7, 144.6, 139.6, 137.0, 136.8, 135.7, 134.3, 132.6, 129.8, 129.7, 129.0, 128.7, 128.6, 128.6, 128.6, 127.9, 127.7, 127.6, 126.9, 126.7, 123.9, 50.2, 48.2, 39.3, 37.3, 36.5, 28.1. HRMS (ESI, m/z): calcd for $C_{32}H_{29}NNaO_2^+$ [M + Na] $^+$: 482.2091, found 482.2090.

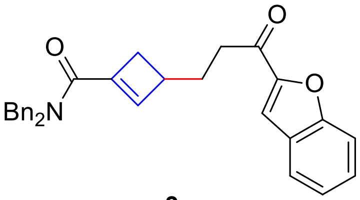

3m

3m, General procedure A (0.2 mmol alkene), yellow oil. 57.9 mg (64% yield). ^{1}H NMR (400 MHz, $CDCl_3$) δ 7.70 – 7.68 (m, 1H), 7.57 – 7.55 (m, 1H), 7.49 – 7.45 (m, 2H), 7.37 – 7.29 (m, 7H), 7.25 – 7.23 (m, 2H), 7.17 – 7.15 (m, 2H), 6.47 (s, 1H), 4.64 – 4.53 (m, 4H), 3.03 – 2.98 (m, 1H), 2.98 – 2.94 (m, 2H), 2.80 (dd, J = 11.6, 7.2 Hz, 1H), 2.45 (dd, J = 13.4, 1.2 Hz, 1H), 2.01 – 1.90 (m, 2H). ^{13}C NMR (151 MHz, $CDCl_3$) δ 191.0, 164.6, 155.7, 152.6, 144.4, 139.6, 137.0, 136.7, 129.0, 128.7, 128.61, 128.4, 127.7, 127.6, 127.1, 126.7, 124.0, 123.4, 112.8, 112.5, 50.2, 48.2, 39.2, 37.2, 36.8, 27.8. HRMS (ESI, m/z): calcd for $C_{30}H_{27}NNaO_3^+$ [M + Na] $^+$: 472.1883, found 472.1880.

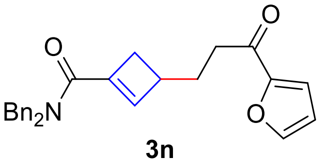

3n

3n, General procedure A (0.2 mmol alkene), yellow oil. 62.9 mg (79% yield). ^{1}H NMR (400 MHz, $CDCl_3$) δ 7.57 – 7.52 (m, 1H), 7.38 – 7.27 (m, 6H), 7.24 – 7.23 (m, 2H), 7.17 – 7.14 (m, 3H), 6.51 – 6.50 (m, 1H), 6.44 (s, 1H), 4.62 – 4.52 (m, 4H), 3.00 – 2.95 (m, 1H), 2.83 – 2.79 (m, 2H), 2.78 – 2.73 (m, 1H), 2.41 (d, J = 13.4 Hz, 1H), 1.95 – 1.84 (m, 2H). ^{13}C NMR (151 MHz, $CDCl_3$) δ 199.6, 175.3, 147.0, 137.0, 133.8, 133.2, 128.7, 128.6, 128.5, 128.5, 128.2, 125.0, 52.2, 50.5, 38.5, 35.3, 30.9. HRMS (ESI, m/z): calcd for $C_{26}H_{25}NNaO_3^+$ [M + Na]$^+$: 422.1727, found 422.1715.

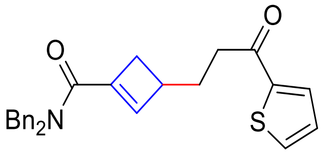

3o

3o, General procedure A (0.2 mmol alkene), yellow oil. 70.2 mg (85% yield). ^{1}H NMR (400 MHz, $CDCl_3$) δ 7.67 (dd, J = 3.8, 0.8 Hz, 1H), 7.61 (dd, J = 5.0, 0.8 Hz, 1H), 7.38 – 7.27 (m, 6H), 7.24 – 7.23 (m, 2H), 7.17 – 7.15 (m, 2H), 7.11 – 7.09 (m, 1H), 6.44 (s, 1H), 4.63 – 4.52 (m, 4H), 3.01 – 2.96 (m, 1H), 2.91 – 2.87 (m, 2H), 2.80 – 2.75 (m, 1H), 2.43 – 2.40 (m, 1H), 1.98 – 1.87 (m, 2H). ^{13}C NMR (151 MHz, $CDCl_3$) δ 192.8, 164.6, 144.4, 144.2, 139.6, 137.0, 136.7, 133.7, 131.9, 129.0, 128.7, 128.6, 128.2, 127.7, 127.6, 126.7, 50.2, 48.2, 39.2, 37.2, 37.2, 28.2. HRMS (ESI, m/z): calcd for $C_{26}H_{25}NNaO_2S^+$ [M + Na]$^+$: 438.1498, found 438.1495.

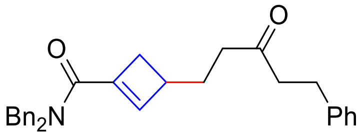

3p

3p, General procedure A (0.2 mmol alkene), yellow oil. 49.7 mg (57% yield). ^{1}H NMR (400 MHz, $CDCl_3$) δ 7.39 – 7.28 (m, 7H), 7.26 – 7.20(m, 3H), 7.19 – 7.14 (m, 5H), 6.39 (s, 1H), 4.63 – 4.53 (m, 4H), 2.93 (dd, J = 13.2, 4.4 Hz, 1H), 2.89 – 2.85 (m, 2H), 2.69 (t, J = 7.6 Hz, 2H), 2.66 – 2.62 (m, 1H), 2.35 (t, J = 7.6 Hz, 2H), 2.32 – 2.30 (m, 1H), 1.78 – 1.67 (m, 2H). ^{13}C NMR (151 MHz, $CDCl_3$) δ 209.7, 164.7, 144.5, 141.1, 139.5, 137.0, 136.8, 129.1, 128.7, 128.6, 128.6, 128.4, 127.7, 127.6, 126.7, 126.2, 50.2, 48.2, 44.4, 40.8, 39.1, 37.1, 29.9, 27.3. HRMS (ESI, m/z): calcd for $C_{30}H_{31}NNaO_2^+$ [M + Na]$^+$: 460.2247, found 460.2245.

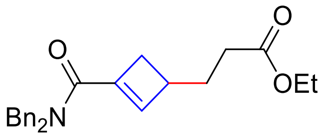

3q

3q, General procedure A (0.2 mmol alkene), yellow oil. 29.4 mg (39% yield). ^{1}H NMR (400 MHz, $CDCl_3$) δ 7.39 – 7.35 (m, 2H), 7.33 – 7.27 (m, 4H), 7.25 – 7.23 (m, 2H), 7.18 – 7.16 (m, 2H), 6.42 (s, 1H), 4.63 – 4.52 (m, 4H), 4.08 (q, *J* = 7.2 Hz, 2H), 2.96 (dd, *J* = 13.4, 4.4 Hz, 1H), 2.73 – 2.68 (m, 1H), 2.38 (dd, *J* = 13.4, 1.2 Hz, 1H), 2.31 – 2.27 (m, 2H), 1.85 – 1.75 (m, 2H), 1.22 (t, *J* = 7.2 Hz, 3H). ^{13}C NMR (101 MHz, $CDCl_3$) δ 173.4, 164.7, 144.2, 139.7, 137.1, 136.8, 129.1, 128.7, 128.7, 127.7, 127.6, 126.7, 60.5, 50.2, 48.2, 39.1, 37.2, 32.5, 28.7, 14.3. HRMS (ESI, m/z): calcd for $C_{24}H_{27}NNaO_3^+$ [M + Na] $^+$: 400.1883, found 400.1874.

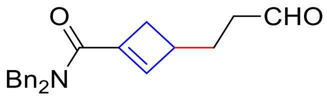

3r

3r, General procedure A (0.2 mmol alkene), yellow oil. 47.8 mg (72% yield). ^{1}H NMR (600 MHz, $CDCl_3$) δ 9.73 (t, *J* = 1.4 Hz, 1H), 7.38 – 7.35 (m, 2H), 7.33 – 7.30 (m, 3H), 7.28 – 7.27 (m, 1H), 7.24 – 7.23 (m, 2H), 7.17 – 7.16 (m, 2H), 6.41 (s, 1H), 4.61 – 4.54 (m, 4H), 2.98 – 2.95 (m, 1H), 2.72 – 2.69 (m, 1H), 2.44 – 2.41 (m, 2H), 2.37 – 2.35 (m, 1H), 1.84 – 1.74 (m, 2H). ^{13}C NMR (151 MHz, $CDCl_3$) δ 201.9, 164.6, 144.1, 139.7, 137.0, 136.7, 129.1, 128.7, 128.6, 127.7, 127.6, 126.6, 50.2, 48.2, 41.8, 38.9, 37.1, 25.6. HRMS (ESI, m/z): calcd for $C_{22}H_{23}NNaO_2^+$ [M + Na] $^+$: 356.1621, found 356.1621.

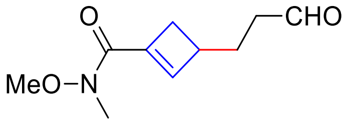

3s

3s, General procedure A (0.1 mmol alkene), yellow oil. 15.5 mg (79% yield). ^{1}H NMR (600 MHz, $CDCl_3$) δ 9.79 (t, *J* = 1.2 Hz, 1H), 6.67 (d, *J* = 0.6 Hz, 1H), 3.68 (s, 3H), 3.23 (s, 3H), 2.92 (dd, *J* = 13.2, 4.2 Hz, 1H), 2.77 – 2.73 (m, 1H), 2.50 (td, *J* = 7.2, 1.2 Hz, 2H), 2.33 (dd, *J* = 13.2, 1.8 Hz, 1H), 1.90 – 1.80 (m, 2H). ^{13}C NMR (151 MHz, $CDCl_3$) δ 202.1, 162.9, 147.0, 138.4, 61.5, 42.0, 39.4, 36.0, 32.7, 25.7. HRMS (ESI, m/z): calcd for $C_{10}H_{15}NNaO_3^+$ [M + Na] $^+$: 220.0944, found 220.0944.

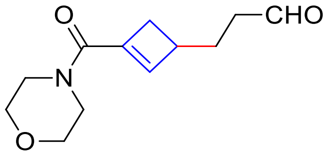

3t

3t, General procedure A (0.2 mmol alkene), yellow oil. 31.2 mg (70% yield). ^{1}H NMR (400 MHz, $CDCl_3$) δ 9.80 (t, *J* = 1.2 Hz, 1H), 6.43 (d, *J* = 0.8 Hz, 1H), 3.72 – 3.68 (m, 4H), 3.66 – 3.64 (m, 4H), 2.96 (dd, *J* = 13.2, 4.4 Hz, 1H), 2.79 – 2.74 (m, 1H), 2.51-2.49 (m, 2H), 2.36 (dd, *J* = 13.2, 1.6 Hz, 1H), 1.91 – 1.80 (m, 2H). ^{13}C NMR (151 MHz, $CDCl_3$) δ 201.6, 162.9, 143.5, 139.6, 66.9, 42.0, 39.4, 37.1, 25.8. HRMS (ESI, m/z): calcd for $C_{12}H_{17}NNaO_3^+$ [M + Na] $^+$: 246.1101, found 246.1101.

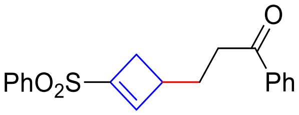

3u

3u, General procedure B (0.1 mmol BCBs), neat without any solvent. yellow oil. 3.9 mg (12% yield). ^{1}H NMR (400 MHz, $CDCl_3$) δ 7.92 – 7.88 (m, 4H), 7.66 – 7.62 (m, 1H), 7.59 – 7.52 (m, 3H), 7.48 – 7.44 (m, 2H), 6.73 (s, 1H), 2.99 – 2.95 (m, 2H), 2.93 – 2.87 (m, 2H), 2.36 (d, *J* = 11.8 Hz, 1H), 1.98 – 1.91 (m, 2H). ^{13}C NMR (151 MHz, $CDCl_3$) δ 199.3, 147.5, 143.4, 138.9, 136.8, 133.8, 133.4, 129.4, 128.8, 128.2, 128.1, 39.5, 36.0, 35.5, 27.0. HRMS (ESI, m/z): calcd for $C_{19}H_{18}NaO_3S^+$ [M + Na] $^+$: 349.0869, found 349.0868.

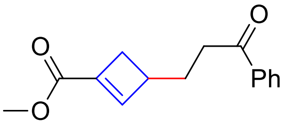

3v

3v, General procedure B (0.1 mmol BCBs), neat without any solvent. yellow oil. 12.1 mg (50% yield), ^{1}H NMR (600 MHz, $CDCl_3$) δ 7.96 – 7.95 (m, 2H), 7.58 – 7.55 (m, 1H), 7.48 – 7.46 (m, 2H), 6.85 (s, 1H), 3.73 (s, 3H), 3.02 (t, *J* = 7.2 Hz, 2H), 2.88 – 2.85 (m, 1H), 2.85 – 2.82 (m, 1H), 2.32 – 2.30 (m, 1H), 2.02 – 1.91 (m, 2H). ^{13}C NMR (151 MHz, $CDCl_3$) δ 199. 8, 163.1, 149.5, 137.5, 137.0, 133.3, 128.8, 128.2, 51.5, 39.5, 36.5, 34.7, 27.7. HRMS (ESI, m/z): calcd for $C_{15}H_{16}NaO_3^+$ [M + Na] $^+$: 267.0992, found 267.0992.

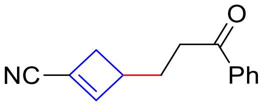

3w

3w, General procedure B (0.1 mmol BCBs), neat without any solvent. yellow oil. 5.0 mg (24% yield). ^{1}H NMR (600 MHz, $CDCl_3$) δ 7.95 (d, J = 7.2 Hz, 2H), 7.59 – 7.57 (m, 1H), 7.49 – 7.47 (m, 2H), 6.86 (s, 1H), 3.01 (t, J = 7.2 Hz, 3H), 2.96 (dd, J = 13.2, 4.4 Hz, 1H), 2.45 (d, J = 13.2 Hz, 1H), 2.05 – 1.94 (m, 2H). ^{13}C NMR (151 MHz, $CDCl_3$) δ 199.3, 156.0, 136.8, 133.4, 128.9, 128.2, 116.7, 114.1, 42.9, 38.0, 36.1, 27.0. HRMS (ESI, m/z): calcd for $C_{14}H_{13}NNaO^+$ [M + Na] $^+$: 234.0889, found 234.0893.

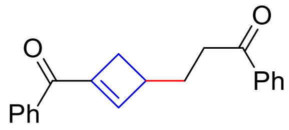

3x

3x, General procedure B (0.1 mmol BCBs), neat without any solvent. yellow oil. 10.0 mg (34% yield). ^{1}H NMR (600 MHz, $CDCl_3$) δ 7.98 – 7.97 (m, 2H), 7.89 – 7.87 (m, 2H), 7.58 – 7.54 (m, 2H), 7.49 – 7.44 (m, 4H), 6.81 (d, J = 1.0 Hz, 1H), 3.09 – 3.06 (m, 2H), 3.06 – 3.03 (m, 1H), 2.96 – 2.93 (m, 1H), 2.48 (dd, J = 13.2, 1.8 Hz, 1H), 2.11 – 2.06 (m, 1H), 2.05 – 1.99 (m, 1H). ^{13}C NMR (151 MHz, $CDCl_3$) δ 199.8, 189.3, 150.0, 144.3, 137.1, 137.0, 133.3, 132.7, 128.9, 128.8, 128.6, 128.2, 39.5, 36.5, 34.7, 27.9. HRMS (ESI, m/z): calcd for $C_{20}H_{18}NaO_2^+$ [M + Na] $^+$: 313.1199, found 313.1192.

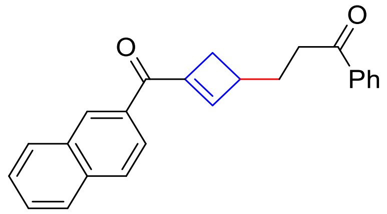

3xa

3xa, General procedure B (0.1 mmol BCBs), neat without any solvent. yellow oil. 20.0 mg (59% yield), ^{1}H NMR (400 MHz, $CDCl_3$) δ 8.43 (s, 1H), 8.00 – 7.98 (m, 2H), 7.96 – 7.93 (m, 2H), 7.90 – 7.87 (m, 2H), 7.62 – 7.53 (m, 3H), 7.50 – 7.46 (m, 2H), 6.89 (d, J = 1.2 Hz, 1H), 3.13 – 3.08 (m, 3H), 3.02 – 2.97 (m, 1H), 2.57 – 2.53 (m, 1H), 2.17 – 2.01 (m, 2H). ^{13}C NMR (101 MHz, $CDCl_3$) δ 199.9, 189.2, 149.9, 144.5, 137.0, 135.5, 134.4, 133.3, 132.6, 130.5, 129.6, 128.8, 128.6, 128.4, 128.2, 127.9, 126.9, 124.8, 39.6, 36.5, 34.9, 27.9. HRMS (ESI, m/z): calcd for $C_{24}H_{20}NaO_2^+$ [M + Na] $^+$: 363.1356, found 363.1357.

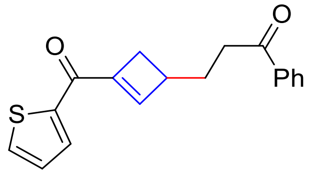

3xb

3xb, General procedure B (0.1 mmol BCBs), neat without any solvent. yellow oil. 11.6 mg (39% yield), ^{1}H NMR (400 MHz, $CDCl_3$) δ 7.99 – 7.96 (m, 2H), 7.80 – 7.79 (m, 1H), 7.65 – 7.63 (m, 1H), 7.60 – 7.55 (m, 1H), 7.50 – 7.46 (m, 2H), 7.15 – 7.12 (m, 1H), 6.97 (d, J = 1.0 Hz, 1H), 3.10 – 3.05 (m, 3H), 2.97 – 2.92 (m, 1H), 2.52 – 2.49 (m, 1H), 2.13 – 1.97 (m, 2H). ^{13}C NMR (101 MHz, $CDCl_3$) δ 199.8, 180.3, 148.7, 144.1, 143.1, 137.0, 133.6, 133.3, 132.6, 128.8, 128.2, 39.7, 36.5, 35.0, 27.8. HRMS (ESI, m/z): calcd for $C_{18}H_{16}NaO_2S^+$ [M + Na] $^+$: 319.0763, found 319.0764.

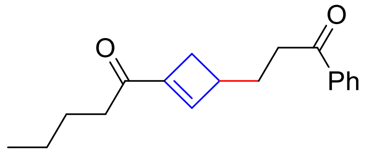

3xc

3xc, General procedure B (0.1 mmol BCBs), neat without any solvent. yellow oil. 9.2 mg (34% yield), ^{1}H NMR (400 MHz, $CDCl_3$) δ 7.97 – 7.94 (m, 2H), 7.59 – 7.55 (m, 1H), 7.49 – 7.45 (m, 2H), 6.77 (s, 1H), 3.04 –3.00 (m, 2H), 2.85 – 2.79 (m, 2H), 2.55 – 2.52 (m, 2H), 2.27 – 2.23 (m, 1H), 2.06 – 1.89 (m, 2H), 1.63 – 1.54 (m, 2H), 1.37 – 1.28 (m, 2H), 0.90 (t, J = 7.4 Hz, 3H). ^{13}C NMR (101 MHz, $CDCl_3$) δ 199.8, 197.6, 147.4, 145.7, 137.0, 133.3, 128.8, 128.2, 38.8, 37.6, 36.4, 33.5, 27.8, 26.6, 22.6, 14.0. HRMS (ESI, m/z): calcd for $C_{18}H_{22}NaO_2^+$ [M + Na] $^+$: 293.1512, found 293.1513.

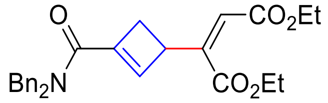

3y

3y, General procedure B (0.1 mmol BCBs), yellow oil. 32.2 mg (72% yield). ^{1}H NMR (400 MHz, $CDCl_3$) δ 7.39 – 7.35 (m, 2H), 7.33 – 7.27 (m, 4H), 7.24 – 7.22 (m, 2H), 7.17 – 7.15 (m, 2H), 6.34 (s, 1H), 5.78 (d, J = 0.8 Hz, 1H), 4.64 – 4.52 (m, 4H), 4.22 (q, J = 7.2 Hz, 2H), 4.16 (q, J = 7.2 Hz, 2H), 3.61 – 3.60 (m, 1H), 3.18 (dd, J = 13.6, 4.8 Hz, 1H), 2.82 (dd, J = 13.6, 1.6 Hz, 1H), 1.26 (t, J = 7.2 Hz, 3H), 1.25 (t, J = 7.2 Hz, 3H). ^{13}C NMR (151 MHz, $CDCl_3$) δ 167.6, 164.9, 164.1, 149.6, 141.7, 139.2, 136.6, 136.3, 129.1, 128.8, 128.6, 127.9, 127.7, 126.6, 119.6, 61.6, 61.0, 50.2, 48.3, 41.7, 37.9, 14.2, 14.1. HRMS (ESI, m/z): calcd for $C_{27}H_{29}NNaO_5^+$ [M + Na] $^+$: 470.1938, found 470.1936.

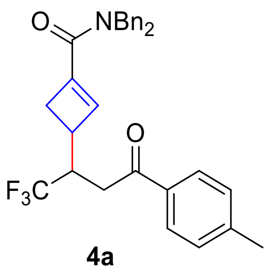

4a

4a, General procedure C (0.1 mmol alkene), yellow oil. 58.8 mg (99% yield), dr = 5: 1, **4a**:**5a** > 20:1. ^{1}H NMR (400 MHz, $CDCl_3$) δ major: δ 7.79 (d, *J* = 8.2 Hz, 2H), 7.33 – 7.24 (m, 4H), 7.22 – 7.20 (m, 4H), 7.17 – 7.16 (m, 2H), 7.04 (d, *J* = 7. 0 Hz, 2H), 6.17 (s, 1H), 4.65 – 4.51 (m, 2H), 4.48 – 4.37 (m, 2H), 3.35 – 3.27 (m, 1H), 3.18 – 3.12 (m, 1H), 3.08 – 3.00 (m, 1H), 2.96 – 2.85 (m, 2H), 2.60 – 2.55 (m, 1H), 2.37 (s, 3H). δ minor: 7.74 (d, *J* = 8.2 Hz, 2H), 7.09 (d, *J* = 7.2 Hz, 2H), 6.15 (s, 1H), 2.38 (s, 3H). ^{13}C NMR (101 MHz, $CDCl_3$) δ mixture of isomers (5:1): 195.8, 195.7, 172.1, 163.9 144.6, 141.1, 140.3, 137.2, 136.7, 136.3, 133.8, 129.5. 129.0, 128.7, 128.5, 128.3, 127.7, 127.7, 126.5, 50.1, 48.1, 40.1 (q, *J* = 25.9 Hz), 37.9, 37.6 (q, *J* = 19.3 Hz), 37.0, 34.4, 34.0, 33.7, 21.7, 13.6, 8.3. ^{19}F NMR (377 MHz, $CDCl_3$) δ major: -69.82 (d, *J* = 9.2 Hz, 3F), δ minor: -70.26 (d, *J* = 9.4 Hz, 3F). HRMS (ESI, m/z): calcd for $C_{30}H_{28}F_3NO_2{}^+$ [M + Na]$^+$: 514.1964, found 514.1965.

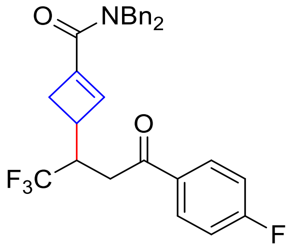

4b

4b, General procedure C (0.1 mmol alkene), yellow oil. 57.0 mg (99% yield) dr = 5: 1, **4b**:**5b** = 17:1. ^{1}H NMR (400 MHz, $CDCl_3$) δ major: 7.96 – 7.91 (m, 2H) 7.33 – 7.27 (m, 6H), 7.20 – 7.18 (m, 2H), 7.15 – 7.14 (m, 2H), 7.08 – 7.06 (m, 2H), 6.20 (s, 1H), 4.56 – 4.49 (m, 4H), 3.35 – 3.27 (m, 1H), 3.20 – 3.13 (m, 1H), 3.07 – 3.03 (m, 1H), 2.98 – 2.86 (m, 2H), 2.58 – 2.56 (m, 1H). δ minor: 7.91 – 7.87 (m, 2H), 6.17 (s, 1H). ^{13}C NMR (151 MHz, $CDCl_3$) δ mixture of isomer (5:1): 194.7, 194.6, 166.2 (d, *J* = 256.0 Hz), 163.9, 140.9, 140.5, 140.1, 136.8, 136.3, 132.7 (d, *J* = 3.1 Hz), 131.0, 130.9, 129.1, 128.7, 128.6, 127.8, 127.7, 126.6, 116.1 (d, *J* = 21.9 Hz). 50.2, 48.2, 40.2 (q, *J* = 26.1 Hz), 37.5, 34.2, 33.9. ^{19}F NMR (565 MHz, $CDCl_3$) δ major: -69.86 (d, *J* = 8.7 Hz, 3F), -104.00 (s, 1F). δ minor: -70.31 (d, *J* = 9.7 Hz, 3F). HRMS (ESI, m/z): calcd for $C_{29}H_{25}F_4NO_2{}^+$ [M + Na]$^+$: 518.1714, found 518.1704.

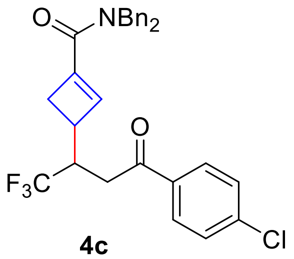

4c

4c, General procedure C (0.1 mmol alkene), yellow oil. 50.5 mg (99% yield) dr = 5:1, **4c**:**5c** > 20:1. ^{1}H NMR (400 MHz, $CDCl_3$) δ 7.86 – 7.83 (m, 2H), 7.45 – 7.41 (m, 2H), 7.33 – 7.27 (m, 6H), 7.21 – 7.19 (m, 2H), 7.08 – 7.06 (m, 2H), 6.20 (s, 1H), 4.56 – 4.49 (m, 4H), 3.36 – 3.26 (m, 1H), 3.21 – 3.13 (m, 1H), 3.08 – 3.04 (m, 1H), 2.97 (dd, *J* = 13.8, 4.4 Hz, 1H), 2.88 (dd, *J* = 18.0, 6.0 Hz, 1H), 2.58 (d, *J* = 13.8 Hz, 1H). δ minor: 7.81 – 7.78 (m, 2H), 7.13 – 7.11 (m, 2H), 6.17 (s, 1H), 4.67-4.58 (m, 4H). ^{13}C NMR (101 MHz, $CDCl_3$) δ mixture of isomers (5:1): 195.0, 164.0, 163.9, 140.8, 140.5, 140.3, 139.9, 136.7, 136.4, 134.5, 129.6, 129.2, 129.1, 128.7, 128.7, 128.6, 127.8, 127.7, 126.5, 126.5, 50.2, 48.3, 48.2, 40.2 (q, *J* = 26.1 Hz), 37.8, 37.5, 37.5, 37.0, 36.0, 34.5, 34.1, 33.9, 33.9. ^{19}F NMR (377 MHz, $CDCl_3$) δ major: -69.82 (d, *J* = 9.3 Hz, 3F), δ minor: -70.29 (d, *J* = 8.9 Hz, 3F). HRMS (ESI, m/z): calcd for $C_{29}H_{25}ClF_3NO_2{}^{+}$ [M + Na]$^{+}$: 534.1418, found 534.1415.

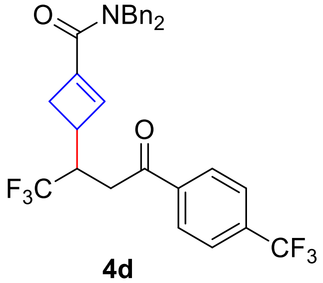

4d

4d, General procedure C (0.1 mmol alkene), yellow oil. 40.5 mg (74% yield), dr = 5: 1, **4d**:**5d** = 20:1. ^{1}H NMR (400 MHz, $CDCl_3$) δ major: 8.00 (d, *J* = 8.2 Hz, 2H), 7.73 – 7.71 (m, 2H), 7.30 – 7.29 (m, 6H), 7.20 – 7.18 (m, 2H), 7.07 (d, *J* = 6.8 Hz, 2H), 6.21 (s, 1H), 4.58 – 4.51 (m, 4H), 3.38 – 3.30 (m, 1H), 3.27 – 3.24 (m, 1H), 3.09 – 3.04 (m, 1H), 2.97 – 2.87 (m, 2H), 2.61 – 2.56 (m, 1H). δ minor: 7.95 (d, *J* = 8.0 Hz, 2H), 7.12 (d, *J* = 6.8 Hz, 2H), 6.17 (s, 1H), 4.66 – 4.56 (m, 4H). ^{13}C NMR (101 MHz, $CDCl_3$) δ mixture of isomers (5:1): 195.4, 195.4, 163.9, 140.7, 140.6, 139.8, 138.8, 136.7, 136.3, 135.0 (q, *J* = 32.7 Hz), 129.8, 128.7, 128.6, 128.6, 127.8, 127.1, 126.5, 125.9 (q, *J* = 3.5 Hz), 123.6 (q, *J* = 273.0 Hz), 50.2, 48.2, 40.2 (q, *J* = 26.2 Hz), 37.5, 34.3, 34.2. ^{19}F NMR (377 MHz,

$CDCl_3$) δ major: -63.16 (s, 3F), -69.86 (d, J = 8.8 Hz, 3F), δ minor: -63.18 (s, 3F), -70.34 (d, J = 9.3 Hz, 3F). HRMS (ESI, m/z): calcd for $C_{30}H_{25}F_6NO_2{}^+$ [M + Na] $^+$: 568.1682, found 568.1686.

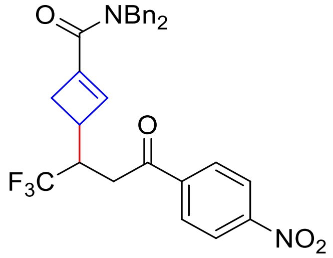

4e

4e, General procedure C (0.1 mmol alkene), yellow oil. 57.2 mg (99% yield) dr = 5: 1, **4e**:**5e** = 8:1. ^{1}H NMR (400 MHz, $CDCl_3$) δ major: 8.29 (d, J = 8.8 Hz, 2H), 8.29 (d, J = 8.8 Hz, 2H), 7.31 – 7.28 (m, 6H), 7.20 – 7.18 (m, 2H), 7.08 – 7.06 (m, 2H), 6.20 (s, 1H), 4.57 – 4.46 (m, 4H), 3.30 – 3.28 (m, 1H), 3.24 – 3.21 (m, 1H), 3.08 – 3.05 (m, 1H), 2.99 – 2.89 (m, 2H), 2.59 – 2.55 (m, 1H). δ minor: 8.04 (d, J = 8.8 Hz, 2H), 7.24 – 7.22 (m, 2H), 6.18 (s, 1H), 4.64 – 4.55 (m, 4H), 3.38 – 3.34 (m, 1H), 3.00 – 2.99 (m, 2H). ^{13}C NMR (101 MHz, $CDCl_3$) δ mixture of isomers (5:1): 195.0, 194.9, 163.9, 150.8, 140.8, 140.6, 140.5, 136.7, 136.4, 129.3, 129.1, 129.0, 128.8, 128.6, 127.9, 127.8, 126.6, 124.1, 50.3, 48.3, 40.3 (q, J = 26.3 Hz), 37.5, 34.6, 34.2. ^{19}F NMR (377 MHz, $CDCl_3$) δ major: -69.87 (d, J = 8.7 Hz, 3F), δ minor: -70.35 (d, J = 8.7 Hz, 3F). HRMS (ESI, m/z): calcd for $C_{29}H_{25}F_3N_2O_4{}^+$ [M + H] $^+$: 523.1839, found 523.1837.

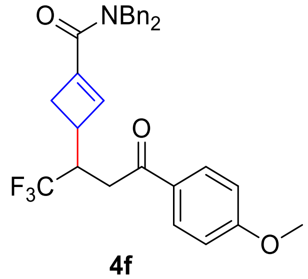

4f

4f, General procedure C (0.1 mmol alkene), yellow oil. 51.4 mg (99% yield), dr = 5:1, **4f**:**5f** >20:1. ^{1}H NMR (400 MHz, $CDCl_3$) δ major : 7.89 (d, J = 8.8 Hz, 2H), 7.32 – 7.28 (m, 6H), 7.18 – 7.17 (m, 2H), 7.07 (d, J = 7.0 Hz, 2H), 6.93 –6.90 (m, 2H), 6.20 (s, 1H), 4.59 – 4.47 (m, 4H), 3.87 (s, 3H), 3.36 – 3.27 (m, 1H), 3.16 – 3.11 (m, 1H), 3.04 – 3.03 (m, 1H), 2.99 – 2.89 (m, 2H), 2.60 – 2.57 (m, 1H). δ minor : 7.85 (d, J = 8.8 Hz, 2H), 7.23 – 7.21 (m, 2H), 7.12 (d, J = 7.0 Hz, 2H), 6.19 (s, 1H), 4.65 – 4.52 (m, 4H), 3.87 (s, 3H). ^{13}C NMR (101 MHz, $CDCl_3$) δ mixture of isomers (5:1): 194.7, 164.1, 164.0, 141.2, 140.5, 140.3, 136.8, 136.4, 130.6, 129.4, 129.1, 128.7, 128.6, 127.8, 127.7, 126.6, 114.0, 55.7, 50.2, 48.2, 40.2 (q, J = 25.8 Hz), 37.6, 36.1, 34.1, 33.6. ^{19}F NMR (377 MHz,

$CDCl_3$) δ major -69.80 (d, *J* = 9.3 Hz, 3F), δ minor : -70.23 (d, *J* = 9.5 Hz, 3F). HRMS (ESI, m/z): calcd for $C_{30}H_{28}F_3NO_3^+$ [M + Na] $^+$: 530.1913, found: 530.1919.

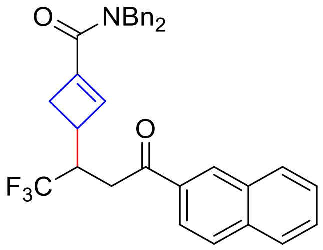

4g

4g, General procedure C (0.1 mmol alkene), yellow oil. 52.1 mg (99% yield) dr = 5:1, **4g**:**5g** = 20:1. ^{1}H NMR (400 MHz, $CDCl_3$) δ major : 8.41 (s 1H), 8.02 – 7.96 (m, 2H), 7.96 – 7.93 (m, 2H), 7.69 – 7.58 (m, 2H), 7.31 – 7.16 (m, 6H), 7.16 – 7.14 (m, 2H), 7.03 – 7.01 (m, 2H), 6.23 (s, 1H), 4.54 – 4.46 (m, 4H), 3.47 – 3.36 (m, 2H), 3.11 – 3.09 (m, 2H), 3.01 – 2.96 (m, 1H), 2.67 – 2.63(m, 1H). δ minor : 8.39 (s, 1H), 6.23 (s, 1H), 4.68 – 4.56 (m, 4H). ^{13}C NMR (101 MHz, $CDCl_3$) δ mixture of isomers (5:1): 196.2, 196.2, 164.0, 141.1, 140.5, 140.4, 136.7, 136.3, 135.9, 133.6, 132.5, 130.0, 129.8, 129.0, 129.0, 128.8, 128.7, 128.5, 127.9, 127.8, 127.6, 127.1, 126.5, 123.8, 50.3, 48.2, 40.3 (q, *J* = 26.0 Hz), 37.6, 34.1, 34.0. ^{19}F NMR (377 MHz, $CDCl_3$) δ major : -69.74 (d, *J* = 8.9 Hz, 3F), δ minor : -70.20 (d, *J* = 9.1 Hz, 3F). HRMS (ESI, m/z): calcd for $C_{33}H_{28}F_3NO_2^+$ [M + Na] $^+$: 550.1964, found 550.1960.

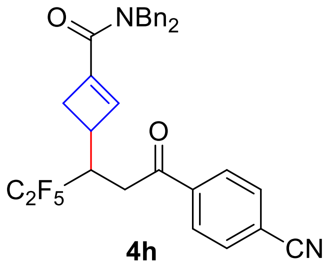

4h

4h, General procedure C (0.1 mmol alkene), yellow oil. 54 mg (98% yield) dr =10:1, **4h**:**5h** > 20:1. ^{1}H NMR (400 MHz, $CDCl_3$) δ major : 7.97 (d, *J* = 8.4 Hz, 2H), 7.73 (d, *J* = 8.4 Hz, 2H), 7.38 – 7.27 (m, 6H), 7.16 – 7.18 (m, 2H), 7.03 – 7.01 (m, 2H), 6.08 (s, 1H), 4.51 – 4.42 (m, 4H), 3.53 – 3.41 (m, 1H), 3.28 – 3.22 (m, 1H), 3.22 – 3.17 (m, 1H), 2.99 – 2.91 (m, 2H), 2.61 (d, *J* = 14.2 Hz, 1H). δ minor : 7.92 (d, *J* = 8.4 Hz, 2H), 6.12 (s, 1H), 4.68 – 4.54 (m, 4H), 2.50 (d, *J* = 14.2 Hz, 1H). ^{13}C NMR (101 MHz, $CDCl_3$) δ mixture of isomers (5:1) : 195.1, 195.0, 163.8, 141.0, 140.4, 138.9, 136.6, 136.2, 132.7, 129.1, 128.8, 128.6, 127.9, 127.8, 126.5, 117.8, 117.1, 50.2, 48.2, 37.3, 37.1, 36.9, 33.7,

33.0. ^{19}F NMR (377 MHz, $CDCl_3$) δ major: -82.13 (s, 3F), -115.19 – -118.96 (m, 2F), δ minor : -82.06 (s, 3F), HRMS (ESI, m/z): calcd for $C_{31}H_{25}F_5N_2O_2^+$ [M + H] $^+$: 553.1909, found 553.1910.

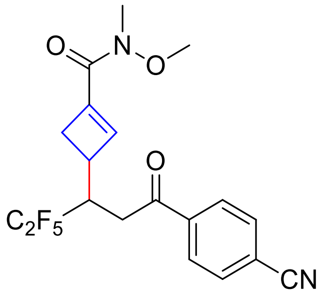

4i

4i, General procedure C (0.1 mmol alkene), yellow oil. 15.0 mg (36% yield), dr = 13: 1, **4a**:**5a** > 20:1. ^{1}H NMR (400 MHz, $CDCl_3$) δ major : 8.03 (d, *J* = 4.4 Hz, 2H), 7.79 (d, *J* = 8.4, 2H), 6.44 (s, 1H), 3.60 (s, 3H), 3.56 – 3.49 (m, 1H), 3.37 – 3.31 (m, 1H), 3.24 – 3.23 (m, 1H), 3.18 (s, 3H), 3.00 – 2.94 (m, 1H), 2.92 – 2.87 (m, 1H), 2.54 (d, *J* = 14.2 Hz, 1H). δ minor : 6.48 (s, 1H), 3.66 (s, 3H), 3.22 (s, 3H) . ^{13}C NMR (101 MHz, $CDCl_3$) δ mixture of isomers (13:1): 195.1, 162.0, 144.1, 139.5, 139.1, 132.8, 128.7, 117.8, 117.1, 61.5, 37.4, 37.3 (t, *J* = 20.3 Hz), 33.3, 32.8, 32.7. ^{19}F NMR (377 MHz, $CDCl_3$) δ major : -82.08 (s, 3F), -115.36 – -118.86 (m, 2F). δ minor : -82.01 (s, 3F) . HRMS (ESI, m/z): calcd for $C_{19}H_{17}F_5N_2NaO_3^+$ [M + Na] $^+$: 439.1052, found 439.1046.

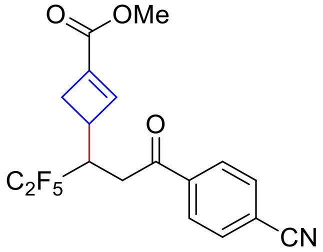

4j

4j, General procedure C (0.1 mmol alkene), yellow oil. 8.0 mg (21% yield), dr = 6: 1, **4a**:**5a** > 20:1. ^{1}H NMR (400 MHz, $CDCl_3$) δ major : 8.07 – 8.02 (m, 2H), 7.82 – 7.78 (m, 2H), 6.60 (s, 1H), 3.69 (s, 3H), 3.60 – 3.47 (m, 1H), 3.40 – 3.33 (m, 1H), 3.24 – 3.21 (m, 1H), 2.94 – 2.88 (m, 1H), 2.84 (dd, *J* = 14.0, 4.4 Hz, 1H), 2.48 (d, *J* = 14.2 Hz, 1H). δ minor : 3.73 (s, 3H). ^{13}C NMR (101 MHz, $CDCl_3$) δ mixture of isomers (6:1): 194.9, 162.2, 146.2, 139.1, 138.7, 132.9, 128.7, 117.8, 117.2, 51.7, 37.4, 37.2 (t, *J* = 17.9 Hz), 33.7, 33.4, 31.8. ^{19}F NMR (377 MHz, $CDCl_3$) δ major : -82.10 (s, 3F), -115.65 – -118.85 (m, 2F). δ minor : -82.10 (s, 3F). HRMS (ESI, m/z): calcd for $C_{18}H_{14}F_5NNaO_3^+$ [M + Na] $^+$: 410.0786, found 410.0780.

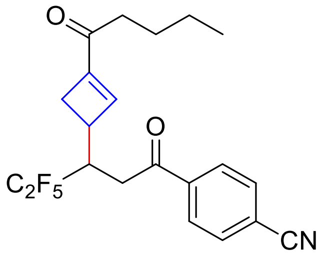

4k

4k, General procedure C (0.1 mmol alkene), yellow oil. 14.0 mg (34% yield), dr = 7: 1, **4a**:**5a** > 20:1. ^{1}H NMR (400 MHz, $CDCl_3$) δ major : 8.06 –8.02 (m, 2H), 7.81 –7.79 (m, 2H), 6.51 (s, 1H), 3.59 – 3.47 (m, 1H), 3.36 – 3.01 (m, 1H), 3.22 – 3.20 (m, 1H), 2.97 – 2.91 (m, 1H), 2.81 – 2.76 (m, 1H), 2.47 – 2.34 (m, 3H), 1.52 – 1.42 (m, 2H), 1.26 – 1.21 (m, 2H), 0.84 (t, J = 7.4 Hz, 3H). δ minor : ^{1}H NMR (400 MHz, $CDCl_3$) δ 0.90 (t, J = 7.4 Hz, 3H). ^{13}C NMR (101 MHz, $CDCl_3$) δ mixture of isomers (7:1): 196.8, 196.7, 195.0, 194.8, 146.9, 146.3, 143.9, 142.2, 139.0, 132.9, 128.7, 128.6, 117.7, 117.2, 37.7, 37.5 (t, J = 20.7 Hz), 36.7, 33.6, 32.6, 30.6, 26.3, 22.5, 13.9. ^{19}F NMR (377 MHz, $CDCl_3$) δ major : -82.05 (s, 3F), -115.63 – -118.48 (m, 2F). δ minor : -82.00 (s, 3F) . HRMS (ESI, m/z): calcd for $C_{21}H_20F_5NNaO_2^+$ [M + Na] $^+$: 436.1306, found 436.1305.

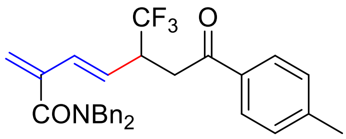

5a

5a, General procedure D (0.1 mmol alkene), yellow oil. 46 mg (95% yield). ^{1}H NMR (400 MHz, $CDCl_3$) δ 7.76 (d, J = 8.2 Hz, 2H), 7.37 – 7.27 (m, 7H), 7.24 – 7.17 (m, 3H), 7.05 (d, J = 6.8 Hz, 2H), 6.40 (d, J = 16.0 Hz, 1H), 5.59 (dd, J = 16.0, 8.8 Hz, 1H), 5.34 (s, 1H), 5.33 (s, 1H), 4.62 – 4.47 (m, 2H), 4.24 – 4.12 (m, 2H), 3.80 – 3.69 (m, 1H), 3.22 (dd, J = 17.4, 3.6 Hz, 1H), 3.13 (dd, J = 17.4, 9.0 Hz, 1H), 2.43 (s, 3H). ^{13}C NMR (101 MHz, $CDCl_3$) δ 194.9, 170.0, 144.7, 142.7, 136.9, 136.2, 134.2, 134.0, 129.6, 128.9, 128.8, 128.8, 128.4, 127.9, 127.8, 127.3, 126.8 (q, J = 279.2 Hz), 126.0 (q, J = 1.8 Hz), 118.7, 50.8, 46.2, 42.5 (q, J = 28.0 Hz), 37.0, 21.8. ^{19}F NMR (377 MHz, $CDCl_3$) δ -70.62 (d, J = 9.3 Hz, 3F). HRMS (ESI, m/z): calcd for $C_{30}H_{28}F_3NO_2^+$ [M + Na] $^+$: 514.1964, found 514.1967.

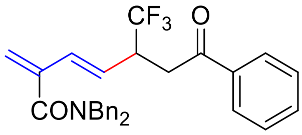

5b

5b, General procedure D (0.1 mmol alkene), yellow oil. 34 mg (72% yield). ^{1}H NMR (400 MHz, $CDCl_3$) δ 7.93 – 7.81 (m, 2H), 7.66 – 7.55 (m, 1H), 7.47 (t, J = 7.4 Hz, 2H), 7.35 – 7.27 (m, 6H), 7.25 – 7.19 (m, 2H), 7.05 (d, J = 6.8 Hz, 2H), 6.41 (d, J = 16.0 Hz, 1H), 5.60 (dd, J = 16.0, 8.8 Hz, 1H), 5.35 (s, 1H), 5.34 (s, 1H), 4.64 – 4.46 (m, 2H), 4.28 – 4.12 (m, 2H), 3.80 – 3.72(m, 1H), 3.26 (dd, J = 17.6, 3.6 Hz, 1H), 3.15 (dd, J = 17.6, 9.0 Hz, 1H). ^{13}C NMR (101 MHz, $CDCl_3$) δ 195.3, 170.0, 142.7, 137.0, 136.4, 136.2, 134.3, 133.8, 128.9, 128.8, 128.8, 128.2, 127.9, 127.8, 127.3, 125.9 (q, J = 2.2 Hz), 118.7, 50.8, 46.3, 42.5 (q, J = 28.1 Hz), 37.2. ^{19}F NMR (377 MHz, $CDCl_3$) δ -70.63 (d, J = 9.2 Hz, 3F). HRMS (ESI, m/z): calcd for $C_{29}H_{26}F_3NO_2^+$ [M + Na] $^+$: 500.1808, found 500.1802.

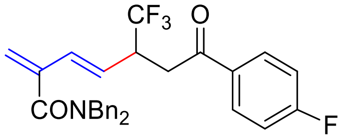

5c

5c, General procedure D (0.1 mmol alkene), yellow oil. 31.9 mg (64% yield). ^{1}H NMR (400 MHz, $CDCl_3$) δ 7.95 – 7.81 (m, 2H), 7.35 – 7.27 (m, 6H), 7.24 – 7.20 (m, 2H), 7.14 (t, J = 8.4 Hz, 2H), 7.07 (d, J = 7.2 Hz, 2H), 6.41 (d, J = 16.0 Hz, 1H), 5.61 (dd, J = 16.0, 8.8 Hz, 1H), 5.36 (s, 1H), 5.35 (s, 1H), 4.62 – 4.50 (m, 2H), 4.29 – 4.14 (m, 2H), 3.79 – 3.71 (m, 1H),3.23 (dd, J = 17.6, 3.6 Hz, 1H), 3.12 (dd, J = 17.6, 9.0 Hz, 1H). ^{13}C NMR (101 MHz, $CDCl_3$) δ 193.6, 170.0, 166.1 (d, J = 255.9 Hz),142.7, 137.0, 136.2, 134.4, 132.9 (d, J = 3.0 Hz), 130.9 (d, J = 9.4 Hz), 128.9, 128.8, 128.7, 127.8, 127.7, 127.3, 126.7 (q, J = 279.3 Hz), 125.8 (q, J = 2.4 Hz), 118.8, 116.0 (d, J = 22.0 Hz), 50.8, 46.3, 42.5 (q, J = 27.8 Hz), 37.1. ^{19}F NMR (377 MHz, $CDCl_3$) δ -70.64 (d, J = 8.6 Hz, 3F), -104.01 - -104.07 (m, 1F). HRMS (ESI, m/z): calcd for $C_{29}H_{25}F_4NO_2^+$ [M + Na] $^+$: 518.1714, found 518.1711.

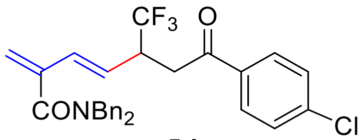

5d

5d, General procedure D (0.1 mmol alkene), yellow oil. 34.2 mg (67% yield). ^{1}H NMR (400 MHz, $CDCl_3$) δ 7.81 – 7.79 (m, 2H), 7.45 – 7.43 (m, 2H), 7.34 – 7.27 (m, 6H), 7.24 – 7.21 (m, 2H), 7.06 (d, *J* = 6.8 Hz, 2H), 6.41 (d, *J* = 15.8 Hz, 1H), 5.59 (dd, *J* = 15.8, 8.8 Hz, 1H), 5.36 (s, 1H), 5.34 (s, 1H), 4.62 – 4.48 (m, 2H), 4.28-4.12 (m, 2H), 3.78 – 3.70 (m, 1H), 3.25 – 3.01 (m, 2H). ^{13}C NMR (101 MHz, $CDCl_3$) δ 194.0, 169.9, 142.6, 140.3, 136.9, 136.1, 134.7, 134.4, 129.6, 129.3, 128.9, 128.8, 128.7, 127.9, 127.8, 127.3, 126.7 (q, *J* = 279.7 Hz), 125.6 (q, *J* = 2.4 Hz), 118.8, 50.8, 46.2, 42.5 (q, *J* = 28.0 Hz), 37.2. ^{19}F NMR (377 MHz, $CDCl_3$) δ -70.64 (d, *J* = 9.3 Hz, 3F). HRMS (ESI, m/z): calcd for $C_{29}H_{25}ClF_3NO_2^+$ [M + Na] $^+$: 534.1418, found 534.1415.

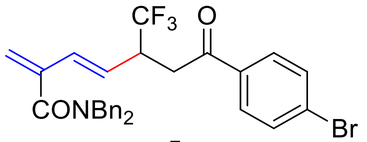

5e

5e, General procedure D (0.1 mmol alkene), yellow oil. 50.9 mg (73% yield). ^{1}H NMR (400 MHz, $CDCl_3$) δ 7.72 (d, *J* = 8.6 Hz, 2H), 7.60 (d, *J* = 8.6 Hz, 2H), 7.35 – 7.27 (m, 6H), 7.22 (d, *J* = 7.6 Hz, 2H), 7.06 (d, *J* = 7.2 Hz, 2H), 6.40 (d, *J* = 16.0 Hz, 1H), 5.59 (dd, *J* = 16.0, 9.0 Hz, 1H), 5.36 (s, 1H), 5.35 (s, 1H), 4.63 – 4.48 (m, 2H) 4.28 – 4.12 (m, 2H) 3.77 – 3.69 (m, 1H) 3.22 (dd, *J* = 17.6, 3.6 Hz, 1H), 3.11 (dd, *J* = 17.6, 9.0 Hz, 1H). ^{13}C NMR (101 MHz, $CDCl_3$) δ 194.3, 170.0, 142.6, 136.9, 136.2, 135.1, 134.4, 132.3, 129.7, 129.1, 129.0, 128.8, 128.8, 127.9, 127.8, 127.3, 126.7 (q, *J* = 279.5 Hz), 125.7 (q, *J* = 2.5 Hz), 118.8, 50.8, 46.3, 42.5 (q, *J* = 27.8 Hz), 37.2. ^{19}F NMR (377 MHz, $CDCl_3$) δ -70.64 (d, *J* = 9.3 Hz, 3F). HRMS (ESI, m/z): calcd for $C_{29}H_{25}BrF_3NO_2^+$ [M + H] $^+$: 556.1094, found 556.1091.

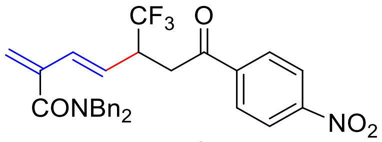

5f

5f, General procedure D (0.1 mmol alkene), yellow oil. 41.1 mg (78% yield). ^{1}H NMR (400 MHz, $CDCl_3$) δ 8.41 – 8.20 (m, 2H), 8.07 – 7.98 (m, 2H), 7.35 – 7.28 (m, 6H), 7.24 – 7.18 (m, 2H), 7.08 (d, *J* = 7.0 Hz, 2H), 6.44 (d, *J* = 15.8 Hz, 1H), 5.63 (dd, *J* = 15.8, 9.0 Hz, 1H), 5.36 (s, 2H), 4.61 – 4.52 (m, 2H), 4.32 – 4.21 (m, 2H), 3.84 – 3.65 (m, 1H), 3.33 (dd, *J* = 17.8, 3.6 Hz, 1H), 3.20 (dd, *J* = 17.8, 9.0 Hz, 1H). ^{13}C NMR (101 MHz, $CDCl_3$) δ 193.9, 169.9, 150.8, 142.5, 140.7, 136.9, 136.1, 134.8, 129.3, 129.0, 128.8, 128.7, 128.0, 127.8, 127.3, 125.3 (q, *J* = 2.3 Hz), 124.2, 119.0, 50.9, 46.3, 42.5

(q, J = 28.0 Hz), 37.9. ^{19}F NMR (377 MHz, $CDCl_3$) δ -70.66 (d, J = 8.9 Hz, 3F). HRMS (ESI, m/z): calcd for $C_{29}H_{25}F_3N_2O_4^+$ [M + Na] $^+$: 545.1659, found 545.1655.

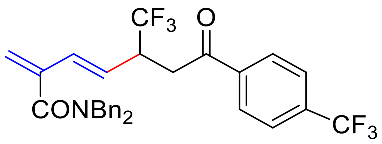

5g

5g, General procedure D (0.1 mmol alkene), yellow oil. 40.8 mg (74% yield). ^{1}H NMR (400 MHz, $CDCl_3$) δ 7.97 (d, J = 8.0 Hz, 2H), 7.74 (d, J = 8.2 Hz, 2H), 7.35 – 7.27 (m, 6H), 7.24 – 7.19 (m, 2H), 7.06 (d, J = 6.8 Hz, 2H), 6.42 (d, J = 16.0 Hz, 1H), 5.61 (dd, J = 16.0, 9.0 Hz, 1H), 5.36 (s, 1H), 5.35 (s, 1H), 4.62 – 4.50 (m, 2H), 4.30 – 4.14 (m, 2H), 3.79 – 3.72 (m, 1H), 3.29 (dd, J = 17.8, 3.6 Hz, 1H), 3.17 (dd, J = 17.8, 9.0 Hz, 1H). ^{13}C NMR (101 MHz, $CDCl_3$) δ 194.4, 170.0, 142.6, 139.0, 137.0, 136.1, 135.1 (q, J = 32.9 Hz), 134.6, 129.0, 128.8, 128.7, 128.6, 127.3, 126.6 (q, J = 279.4 Hz), 126.0 (q, J = 3.7 Hz), 125.5 (q, J = 2.1 Hz), 122.3, 118.9, 50.9, 46.3, 42.5 (q, J = 28.0 Hz), 37.6. ^{19}F NMR (377 MHz, $CDCl_3$) δ -63.21 (s, 3F), -70.67 (d, J = 9.2 Hz, 3F) HRMS (ESI, m/z): calcd for $C_{30}H_{25}F_6NO_2^+$ [M + Na] $^+$: 568.1682, found 568.1678.

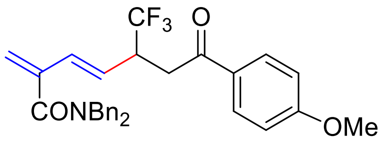

5h

5h, General procedure D (0.1 mmol alkene), yellow oil. 31.8 mg (62% yield). ^{1}H NMR (400 MHz, $CDCl_3$) δ 7.84 (d, J = 8.8 Hz, 2H), 7.35 – 7.28 (m, 6H), 7.24 – 7.21 (m, 2H), 7.05 (d, J = 6.8 Hz, 2H), 6.92 (d, J = 9.0 Hz, 2H), 6.39 (d, J = 16.0 Hz, 1H), 5.59 (dd, J = 15.8, 8.8 Hz, 1H), 5.34 (s, 1H), 5.32 (s, 1H), 4.65 – 4.44 (m, 2H), 4.25 – 4.08 (m, 2H), 3.88 (s, 3H), 3.79 – 3.71 (m, 1H), 3.21 – 3.07 (m, 2H). ^{13}C NMR (101 MHz, $CDCl_3$) δ 193.7, 170.0, 164.0, 142.7, 137.0, 136.2, 134.1, 130.6, 129.5, 128.9, 128.8, 128.7, 127.3, 126.8 (q, J = 279.6 Hz), 126.0 (q, J = 1.9 Hz), 118.6, 114.0, 55.7, 50.7, 46.2, 42.6 (q, J = 27.8 Hz), 36.7. ^{19}F NMR (377 MHz, $CDCl_3$) δ -70.60 (d, J = 8.7 Hz, 3F). HRMS (ESI, m/z): calcd for $C_{30}H_{28}F_3NO_3^+$ [M + Na] $^+$: 530.1913, found 530.1917.

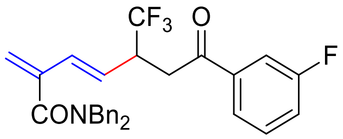

5i

5i, General procedure D (0.1 mmol alkene), yellow oil. 32.5 mg (67% yield). ^{1}H NMR (400 MHz, $CDCl_3$) δ 7.65 (d, *J* = 7.8 Hz, 1H), 7.59 – 7.53 (m, 1H), 7.46 (td, *J* = 8.0, 5.6 Hz, 1H), 7.35 – 7.27 (m, 7H), 7.25 – 7.19 (m, 2H), 7.07 (d, *J* = 7.0 Hz, 2H), 6.42 (d, *J* = 16.0 Hz, 1H), 5.59 (dd, *J* = 16.0, 9.0 Hz, 1H), 5.36 (s, 1H), 5.35 (s, 1H), 4.62 – 4.50 (m, 2H), 4.30 – 4.16 (m, 2H), 3.78 – 3.70 (m, 1H), 3.25 (dd, *J* = 17.8, 3.6 Hz, 1H), 3.11 (dd, *J* = 17.8, 9.0 Hz, 1H). ^{13}C NMR (101 MHz, $CDCl_3$) δ 194.0, 194.0, 170.0, 163.0 (d, *J* = 248.6 Hz), 142.6, 138.4 (d, *J* = 6.1 Hz), 136.9, 136.2, 134.4, 130.6 (d, *J* = 7.8 Hz), 128.9, 128.8, 128.7, 127.9, 127.8, 127.3, 126.7 (q, *J* = 279.5 Hz), 125.6 (q, *J* = 2.2 Hz), 123.9 (d, *J* = 3.0 Hz), 120.8 (d, *J* = 21.4 Hz), 118.8, 115.0 (d, *J* = 22.4 Hz), 50.8, 46.3, 42.4 (q, *J* = 28.0 Hz), 37.4. ^{19}F NMR (377 MHz, $CDCl_3$) δ -70.6 (d, *J* = 8.7 Hz, 3F), -111.- -111.3 (m, 1F). HRMS (ESI, m/z): calcd for $C_{29}H_{25}F_4NO_2^+$ [M + Na] $^+$: 518.1714, found 518.1684.

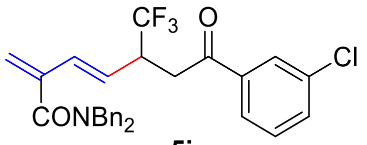

5j

5j, General procedure D (0.1 mmol alkene), yellow oil. 34.9 mg (68% yield). ^{1}H NMR (400 MHz, $CDCl_3$) δ 7.85 (s, 1H), 7.74 (d, *J* = 7.8 Hz, 1H), 7.58 (d, *J* = 8.2 Hz, 1H), 7.42 (t, *J* = 8.0 Hz, 1H), 7.34 – 7.27 (m, 6H), 7.23 – 7.21 (m, 2H), 7.07 (d, *J* = 7.2 Hz, 2H), 6.41 (d, *J* = 16.0 Hz, 1H), 5.58 (dd, *J* = 16.0, 9.0 Hz, 1H), 5.36 (s, 1H), 5.35 (s, 1H), 4.62 – 4.49 (m, 2H), 4.30 – 4.15 (m, 2H), 3.77 – 3.70 (m, 1H), 3.24 (dd, *J* = 17.8, 3.6 Hz, 1H), 3.09 (dd, *J* = 17.8, 9.0 Hz, 1H). ^{13}C NMR (101 MHz, $CDCl_3$) δ 194.0, 170.0, 142.6, 137.9, 136.9, 136.2, 135.4, 134.5, 133.7, 130.3, 129.0, 128.8, 128.7, 128.3, 127.9, 127.8, 127.3, 126.7 (q, *J* = 279.6 Hz), 125.6 (q, *J* = 2.4 Hz), 118.8, 50.9, 46.3, 42.4 (q, *J* = 27.9 Hz), 37.4. ^{19}F NMR (377 MHz, $CDCl_3$) δ -70.62 (d, *J* = 8.3 Hz, 3F). HRMS (ESI, m/z): calcd for $C_{29}H_{25}ClF_3NO_2^+$ [M + Na] $^+$: 534.1418, found 534.1420.

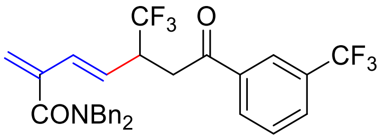

5k

5k, General procedure D (0.1 mmol alkene), yellow oil. 34.9 mg (68% yield). ^{1}H NMR (400 MHz, $CDCl_3$) δ 8.14 (s, 1H), 8.05 (d, *J* = 7.8 Hz, 1H), 7.87 (d, *J* = 7.6 Hz, 1H), 7.63 (t, *J* = 7.8 Hz, 1H), 7.36 – 7.26 (m, 6H), 7.25 – 7.17 (m, 2H), 7.07 (d, *J* = 7.2 Hz, 2H), 6.44 (d, *J* = 16.0 Hz, 1H), 5.59 (dd, *J* = 15.8, 8.8 Hz, 1H), 5.36 (s, 2H), 4.31 – 4.18 (m, 2H), 4.62 – 4.48 (m, 2H), 3.84 – 3.70 (m,

1H), 3.30 (dd, J = 18.0, 3.4 Hz, 1H), 3.15 (dd, J = 18.0, 9.0 Hz, 1H). ^{13}C NMR (101 MHz, $CDCl_3$) δ 193.9, 170.0, 142.6, 136.9 (q, J = 6.7 Hz), 136.2, 134.6, 131.3, 130.2 (q, J = 3.6 Hz), 129.7, 129.0, 128.8 (q, J = 6.8 Hz), 127.9, 127.8, 127.3, 126.7 (q, J = 279.6 Hz), 125.6 (q, J = 2.3 Hz), 125.1 (q, J = 4.0 Hz), 125.3, 122.4, 118.9, 50.9, 46.3, 42.3 (q, J = 28.2 Hz), 37.5. ^{19}F NMR (377 MHz, $CDCl_3$) δ -62.85 (s, 3F), -70.63 (d, J = 9.2 Hz, 3F). HRMS (ESI, m/z): calcd for $C_{30}H_{25}F_6NO_2^+$ [M + Na] $^+$: 568.1682, found 568.1684.

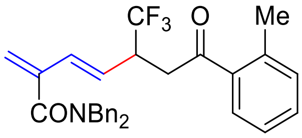

5l

5l, General procedure D (0.1 mmol alkene), yellow oil. 34.2 mg (69% yield). ^1H NMR (400 MHz, $CDCl_3$) δ 7.52 (d, J = 7.6 Hz, 1H), 7.40 (t, J = 7.6 Hz, 1H), 7.36 – 7.27 (m, 6H), 7.26-7.21 (m, 4H), 7.09 (d, J = 7.2 Hz, 2H), 6.40 (d, J = 16.0 Hz, 1H), 5.63 (dd, J = 16.0, 8.8 Hz, 1H), 5.36 (s, 1H), 5.34 (s, 1H), 4.62 – 4.53 (m, 2H), 4.36 – 4.26 (m, 2H), 3.83 – 3.65 (m, 1H), 3.26 – 3.37 (m, 1H) 3.14 – 3.07 (m, 1H), 2.44 (s, 3H). ^{13}C NMR (101 MHz, $CDCl_3$) δ 198.8, 170.0, 142.7, 138.8, 137.1, 137.0, 136.2, 134.2, 132.3, 132.0, 129.0, 128.8, 128.7, 128.6, 127.9, 127.8, 127.3, 126.8 (q, J = 279.4 Hz), 126.0, 118.6, 50.9, 46.3, 42.7 (q, J = 27.8 Hz), 39.9, 21.4. ^{19}F NMR (376 MHz, $CDCl_3$) δ -70.59 (d, J = 9.3 Hz, 3F). HRMS (ESI, m/z): calcd for $C_{30}H_{28}F_3NO_2^+$ [M + Na] $^+$: 514.1964, found 514.1950.

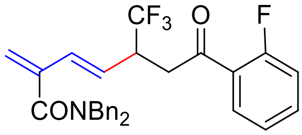

5m

5m, General procedure D (0.1 mmol alkene), yellow oil. 23.4 mg (48% yield). ^1H NMR (400 MHz, $CDCl_3$) δ 7.82 – 7.80 (m, 1H), 7.57 – 7.53 (m, 1H), 7.36 – 7.27 (m, 6H), 7.24 – 7.16 (m, 4H), 7.10 (d, J = 7.0 Hz, 2H), 6.41 (d, J = 16.0 Hz, 1H), 5.59 (dd, J = 16.0, 8.8 Hz, 1H), 5.36 (s, 1H), 5.35 (s, 1H), 4.74 – 4.45 (m, 2H), 4.32 – 4.22 (m, 2H), 3.78 – 3.71 (m, 1H), 3.37 – 3.31 (m, 1H), 3.18 – 3.10 (m, 1H). ^{13}C NMR (101 MHz, $CDCl_3$) δ 193.5 (d, J = 3.8 Hz), 170.0, 162.2 (d, J = 254.8 Hz), 142.8, 136.9, 136.3, 135.3 (d, J = 9.2 Hz), 134.2, 130.8 (d, J = 2.2 Hz), 128.9, 128.7 (d, J = 9.0 Hz), 127.8 (d, J = 10.5 Hz), 127.3, 126.7 (q, J = 279.5 Hz), 126.0 (q, J = 2.3 Hz), 125.0 (q, J = 12.4 Hz), 124.8 (d, J = 3.4 Hz), 117.0 (d, J = 23.5 Hz), 50.9, 46.2, 42.1 (q, J = 11.6 Hz). ^{19}F NMR (377 MHz, $CDCl_3$)

δ -70.64 (s, 3F), -108.88 (s, 1F). HRMS (ESI, m/z): calcd for $C_{29}H_{25}F_4NO_2^+$ [M + H] $^+$: 496.1894, found 496.1897.

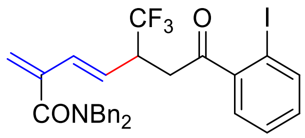

5n

5n, General procedure D (0.1 mmol alkene), yellow oil. 28.5 mg (47% yield). ^{1}H NMR (400 MHz, $CDCl_3$) δ 7.92 (dd, *J* = 8.0, 0.8 Hz, 1H), 7.41 (td, *J* = 7.6, 1.2 Hz, 1H), 7.37 – 7.27 (m, 7H), 7.25 – 7.22 (m, 2H), 7.16 (dd, *J* = 7.6, 1.6 Hz, 1H), 7.14 – 7.09 (m, 2H), 6.44 (d, *J* = 16.0 Hz, 1H), 5.63 (dd, *J* = 16.0, 8.8 Hz, 1H), 5.37 (s, 1H), 5.36 (s, 1H), 4.62 – 4.53 (m, 2H), 4.36 – 4.26 (m, 2H), 3.76 – 3.68 (m, 1H), 3.26 – 3.21 (m, 1H), 3.14 – 3.07 (m, 1H), ^{13}C NMR (101 MHz, $CDCl_3$) δ 199.5, 170.0, 143.4, 142.7, 141.1, 137.0, 136.2, 134.6, 132.3, 129.0, 128.8, 128.7, 128.3, 128.3, 127.35, 126.6 (q, *J* = 279.5 Hz), 125.4 (q, *J* = 2.2 Hz), 118.9, 91.2, 51.0, 46.3, 42.7 (q, *J* = 28.3 Hz), 40.5. ^{19}F NMR (377 MHz, $CDCl_3$) δ -70.62 (d, *J* = 9.2 Hz, 3F).HRMS (ESI, m/z): calcd for $C_{29}H_{25}F_3INO_2^+$ [M + Na] $^+$: 626.0774, found 626.0770.

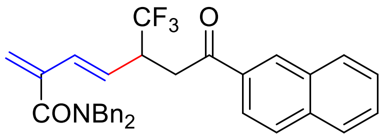

5o

5o, General procedure D (0.1 mmol alkene), yellow oil. 28.7 mg (54% yield). ^{1}H NMR (400 MHz, $CDCl_3$) δ 8.38 (s, 1H), 7.99 – 7.92 (m, 2H), 7.91 – 7.89 (m, 2H), 7.66 – 7.58 (m, 2H), 7.35 – 7.26 (m, 6H), 7.21 – 7.20 (m, 2H), 7.00 (d, *J* = 7.0 Hz, 2H), 6.44 (d, *J* = 15.8 Hz, 1H), 5.64 (dd, *J* = 15.8, 8.8 Hz, 1H), 5.35 (s, 1H), 5.34 (s, 1H), 4.65 – 4.43 (m, 2H) 4.28 – 4.09 (m, 2H), 3.86 – 3.79 (m, 1H), 3.42 – 3.37 (m, 1H), 3.33 – 3.26 (m, 1H). ^{13}C NMR (101 MHz, $CDCl_3$) δ 195.2, 170.0, 142.8, 136.9, 136.2, 136.0, 134.3, 133.8, 132.6, 130.1, 129.8, 129.0, 128.9, 128.9, 128.8, 128.7, 128.0, 127.8, 127.8, 127.3, 127.2, 126.8 (q, *J* = 279.5 Hz), 125.9 (q, *J* = 2.3 Hz), 123.7, 118.7, 50.8, 46.2, 42.6 (q, *J* = 27.7 Hz), 37.3. ^{19}F NMR (377 MHz, $CDCl_3$) δ -70.54 (d, *J* = 9.1 Hz, 3F).HRMS (ESI, m/z): calcd for $C_{33}H_{28}F_3NO_2^+$ [M + Na] $^+$: 550.1964, found 550.1965.

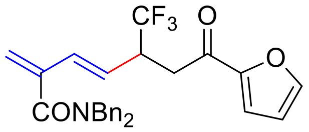

5p

5p, General procedure D (0.1 mmol alkene), yellow oil. 26.5 mg (44% yield). ^{1}H NMR (400 MHz, $CDCl_3$) δ 7.58 (s, 1H), 7.38 – 7.27 (m, 6H), 7.24 – 7.22 (m, 2H), 7.12 (d, *J* = 3.6 Hz, 1H), 7.08 (d, *J* = 7.2 Hz, 2H), 6.55 – 6.54 (m, 1H), 6.38 (d, *J* = 16.0 Hz, 1H), 5.60 (dd, *J* = 16.0, 8.8 Hz, 1H), 5.34 (s, 1H), 5.32 (s, 1H), 4.64 – 4.48 (m, 2H), 4.27 – 4.12 (m, 2H), 3.73 – 3.65 (m, 1H), 3.14 – 3.02 (m, 2H). ^{13}C NMR (101 MHz, $CDCl_3$) δ 184.4, 170.0, 152.3, 147.0, 142.6, 137.0, 136.2, 134.4, 128.9, 128.8, 128.7, 127.9, 127.8, 127.2, 126.6 (q, *J* = 279.3 Hz), 125.5 (q, *J* = 2.1 Hz), 118.8, 117.9, 112.7, 50.7, 46.3, 42.4 (q, *J* = 28.1 Hz), 36.9. ^{19}F NMR (377 MHz, $CDCl_3$) δ -70.80 (d, *J* = 8.6 Hz, 3F). HRMS (ESI, m/z): calcd for $C_{27}H_{24}F_3NO_3^+$ [M + Na] $^+$: 490.1600, found 490.1599.

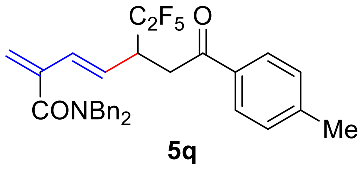

5q

5q, General procedure D (0.1 mmol alkene), yellow oil. 16.3 mg (30% yield). ^{1}H NMR (400 MHz, $CDCl_3$) δ 7.75 (d, *J* = 8.0 Hz, 2H), 7.32 – 7.24 (m, 10H), 7.03 (d, *J* = 6.8 Hz, 2H), 6.37 (d, *J* = 16.0 Hz, 1H), 5.54 (dd, *J* = 16.0, 9.6 Hz, 1H), 5.33 (s, 1H), 5.30 (s, 1H), 4.62 – 4.48 (m, 2H), 4.17 – 4.08 (m, 2H), 3.87 – 3.79 (m, 1H), 3.29 – 3.25 (m, 1H), 3.14 – 3.08 (m, 1H), 2.43 (s, 3H). ^{13}C NMR (101 MHz, $CDCl_3$) δ 194.8, 170.0, 144.7, 142.7, 137.0, 136.3, 134.1, 134.1, 129.6, 128.9, 128.8, 128.4, 127.8, 127.8, 127.1, 125.5 (m), 118.7, 50.6, 46.3, 40.5 (t, *J* = 21.3 Hz), 36.1, 21.8. ^{19}F NMR (377 MHz, $CDCl_3$) δ -81.49 (s, 3F), -115.44 – -122.36 (m, 2F). HRMS (ESI, m/z): calcd for $C_{31}H_{28}F_5NO_2^+$ [M + Na] $^+$: 564.1932, found 564.1939.

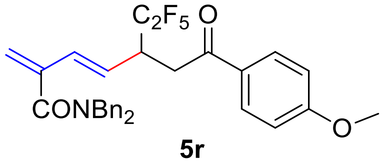

5r

5r, General procedure D (0.1 mmol alkene), yellow oil. 34 mg (60% yield). ^{1}H NMR (400 MHz, $CDCl_3$) δ 7.82 (d, *J* = 9.0 Hz, 2H), 7.37 – 7.28 (m, 6H), 7.22 (dd, *J* = 6.8, 2.4 Hz, 2H), 7.02 (d, *J* = 6.7 Hz, 2H), 6.93 – 6.89 (m, 2H), 6.36 (d, *J* = 16.0 Hz, 1H), 5.53 (dd, *J* = 16.0, 9.6 Hz, 1H), 5.33 (s,

1H), 5.31 (s, 1H), 4.60 – 4.48 (m, 2H),, 4.15 – 4.05 (m, 2H), 3.88 (s, 3H), 3.86 – 3.74 (m, 1H), 3.25 – 3.20 (m, 1H), 3.13 – 3.07 (m, 1H),^{13}C NMR (101 MHz, $CDCl_3$) δ 193.7, 170.0, 164.1, 142.7, 137.0, 136.3, 134.0, 130.6, 129.6, 128.9, 128.8, 127.8, 127.1, 125.6 (m), 118.7, 114.1, 55.7, 50.6, 46.3, 40.6 (t, *J* = 21.6 Hz), 35.8. ^{19}F NMR (377 MHz, $CDCl_3$) δ -81.49 (s, 3F), -115.48 – -122.34 (m, 2F). HRMS (ESI, m/z): calcd for $C_{31}H_{28}F_5NO_3^+$ [M + Na] $^+$: 580.1882, found 580.1889.

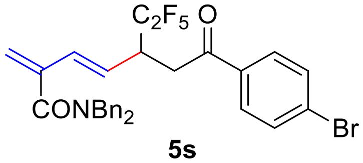

5s

5s, General procedure D (0.1 mmol alkene), yellow oil. 30.4 mg (50% yield). ^{1}H NMR (400 MHz, $CDCl_3$) δ 7.77 – 7.69 (m, 2H), 7.64 – 7.54 (m, 2H), 7.36 – 7.27 (m, 6H), 7.25 – 7.21 (m, 2H), 7.05 (d, *J* = 7.0 Hz, 2H), 6.37 (d, *J* = 15.8 Hz, 1H), 5.54 (dd, *J* = 15.8, 9.6 Hz, 1H), 5.34 (s, 1H), 5.32 (s, 1H), 4.65 – 4.45 (m, 2H), 4.18 – 4.10 (m, 2H), 3.90 – 3.72 (m, 1H), 3.27 (dd, *J* = 17.6, 3.4 Hz, 1H), 3.10 (dd, *J* = 17.6, 8.8 Hz, 1H). ^{13}C NMR (101 MHz, $CDCl_3$) δ 194.3, 169.9, 142.6, 136.9, 136.2, 135.2, 134.3, 132.3, 129.7, 129.1, 129.0, 128.8, 127.9, 127.8, 127.1, 125.2 (m), 118.9, 50.6, 46.3, 40.7 (t, *J* = 20.8 Hz), 36.3. ^{19}F NMR (377 MHz, $CDCl_3$) δ -81.47 (s, 3F), -115.32 – -122.50 (m, 2F). HRMS (ESI, m/z): calcd for $C_{30}H_{25}BrF_5NO_2^+$ [M + H] $^+$: 606.1062, found 606.1049.

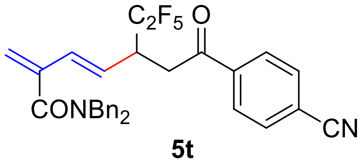

5t

5t, General procedure D (0.1 mmol alkene), yellow oil. 36 mg (64% yield). ^{1}H NMR (400 MHz, $CDCl_3$) δ 8.06 – 7.89 (m, 2H), 7.78 – 7.76 (m, 2H) 7.36 – 7.27 (m, 6H), 7.24 – 7.18 (m, 2H), 7.07 (d, *J* = 7.0 Hz, 2H), 6.40 (d, *J* = 15.8 Hz, 1H), 5.57 (dd, *J* = 15.8, 9.6 Hz, 1H), 5.35 (s, 1H), 5.33 (s, 1H), 4.78 – 4.35 (m, 2H), 4.25 – 4.15 (m, 2H), 3.93 – 3.72 (m, 1H), 3.35 (dd, *J* = 17.9, 3.4 Hz, 1H), 3.16 (dd, *J* = 18.0, 8.8 Hz, 1H). ^{13}C NMR (101 MHz, $CDCl_3$) δ 194.0, 169.8, 142.4, 139.3, 136.9, 136.1, 134.6, 132.8, 129.0, 128.8, 128.7, 128.7, 128.0, 127.8, 127.1, 125.0 (m), 119.1, 117.8, 117.1, 50.71, 46.4, 40.6 (t, *J* = 21.2 Hz), 36.8. ^{19}F NMR (377 MHz, $CDCl_3$) δ -81.46 (s, 3F), -115.15 – -122.67 (m, 2F). HRMS (ESI, m/z): calcd for $C_{31}H_{25}F_5N_2O_2^+$ [M + Na] $^+$: 575.1728, found 575.1730.

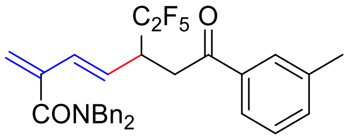

5u

5u, General procedure D (0.1 mmol alkene), yellow oil. 24.3 mg (44% yield). ^{1}H NMR (400 MHz, $CDCl_3$) δ 7.69 (s, 1H), 7.63 (d, *J* = 7.6 Hz, 1H), 7.41 (d, *J* = 7.6 Hz, 1H), 7.36 – 7.28 (m, 7H), 7.22 – 7.20 (m, 2H), 7.03 (d, *J* = 6.8 Hz, 2H), 6.38 (d, *J* = 15.8 Hz, 1H), 5.54 (dd, *J* = 15.8, 9.6 Hz, 1H), 5.34 (s, 1H), 5.31 (s, 1H), 4.63 – 4.46 (m, 2H), 4.18 – 4.09 (m, 2H), 3.83 – 3.79 (m, 1H), 3.32 – 3.27 (m, 1H), 3.16 – 3.10 (m, 1H), 2.41 (s, 3H). ^{13}C NMR (101 MHz, $CDCl_3$) δ 195.4, 170.0, 142.7, 138.8, 136.9, 136.5, 136.3, 134.6, 134.1, 128.9, 128.8, 128.7, 127.9, 127.8, 127.1, 125.6 (m), 125.5, 118.7, 50.6, 46.3, 40.4 (t, *J* = 21.4 Hz), 36.4, 21.5. ^{19}F NMR (377 MHz, $CDCl_3$) δ -81.49 (s, 3F), -115.42 – -122.36 (m, 2F). HRMS (ESI, m/z): calcd for $C_{31}H_{28}F_5NO_2^+$ [M + Na] $^+$: 564.1932, found 564.1927.

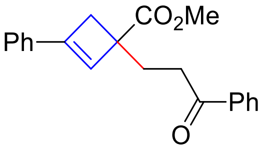

6a

6a, General procedure A (0.1 mmol alkene), yellow oil. 31.9 mg (99% yield). ^{1}H NMR (400 MHz, $CDCl_3$) δ 7.97 (d, *J* = 7.6 Hz, 2H), 7.57 – 7.54 (m, 1H), 7.47 – 7.44 (m, 2H), 7.37 – 7.27 (m, 5H), 6.42 (s, 1H), 3.72 (s, 3H), 3.24 (d, *J* = 13.0 Hz, 1H), 3.15 – 3.01 (m, 2H), 2.72 (d, *J* = 13.0 Hz, 1H), 2.44 – 2.37 (m, 1H), 2.32 – 2.35 (m, 1H). ^{13}C NMR (151 MHz, $CDCl_3$) δ 199.6, 175.3, 147.0, 136.9, 133.8, 133.2, 128.7, 128.6, 128.5, 128.2, 124.9, 52.2, 50.5, 38.4 35.2, 30.9. HRMS (ESI, m/z): calcd for $C_{21}H_{20}NaO_3^+$ [M + Na]$^+$: 343.1305, found 343.1300.

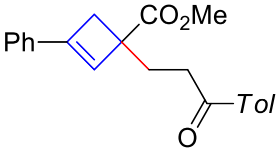

6b

6b, General procedure A (0.2 mmol alkene), yellow solid, mp: 79 ~ 80 °C. 35.7 mg (53% yield). ^{1}H NMR (400 MHz, $CDCl_3$) δ 7.87 (d, *J* = 8.2 Hz, 2H), 7.37 – 7.34 (m, 4H), 7.30 – 7.27 (m, 1H), 7.26- 7.24 (m, 2H), 6.42 (s, 1H), 3.72 (s, 3H), 3.23 (d, *J* = 13.0 Hz, 1H), 3.12 – 2.97 (m, 2H), 2.72 (d, *J* = 13.0 Hz, 1H), 2.43 – 2.36 (m, 4H), 2.32 – 2.24 (m, 1H). ^{13}C NMR (151 MHz, $CDCl_3$) δ 199.2, 175.3,

147.0, 143.9, 134.5, 133.8, 129.4, 128.6, 128.5, 128.5, 128.3, 124.9, 52.1, 50.5, 38.4, 35.1, 31.0, 21.7. HRMS (ESI, m/z): calcd for $C_{22}H_{22}NaO_3^+$ $[M + Na]^+$: 357.1461, found 357.1439.

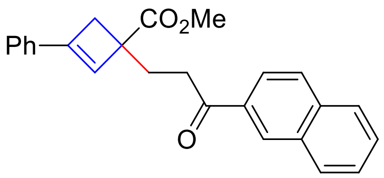

6c

6c, General procedure A (0.2 mmol alkene), yellow solid, mp: 101 ~ 102 °C. 44.8 mg (61% yield). ^1H NMR (400 MHz, $CDCl_3$) δ 8.48 (s, 1H), 8.05 – 8.03 (m, 1H), 7.97 – 7.95 (m, 1H), 7.90 – 7.86 (m, 2H), 7.62 – 7.53 (m, 2H), 7.38 – 7.28 (m, 5H), 6.45 (s, 1H), 3.75 (s, 3H), 3.35 – 3.29 (m, 1H), 3.26 – 3.18 (m, 2H), 2.76 (d, *J* = 13.0 Hz, 1H), 2.52 – 2.44 (m, 1H), 2.40 – 2.33 (m, 1H). ^{13}C NMR (151 MHz, $CDCl_3$) δ 199.5, 175.4, 147.1, 135.7, 134.3, 133.8, 132.7, 129.9, 129.7, 128.6, 128.6, 128.5, 128.5, 127.9, 126.9, 124.9, 124.0, 52.2, 50.5, 38.5, 35.3, 31.1. HRMS (ESI, m/z): calcd for $C_{25}H_{22}NaO_3^+$ $[M + Na]^+$: 393.1461, found 393.1458.

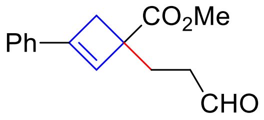

6d

6d, General procedure A (0.2 mmol alkene), yellow oil. 26.5 mg (59% yield). ^1H NMR (600 MHz, $CDCl_3$) δ 9.79 (t, *J* = 1.2 Hz, 1H), 7.36 – 7.33 (m, 4H), 7.31 – 7.28 (m, 1H), 6.35 (s, 1H), 3.70 (s, 3H), 3.20 (d, *J* = 13.2 Hz, 1H), 2.66 (d, *J* = 13.2 Hz, 1H), 2.61 – 2.51 (m, 2H), 2.32 – 2.27 (m, 1H), 2.19 – 2.13 (m, 1H). ^{13}C NMR (101 MHz, $CDCl_3$) δ 201.5, 175.1, 147.2, 133.7, 128.7, 128.6, 128.2, 125.0, 52.2, 50.2, 40.6, 38.3, 28.6. HRMS (ESI, m/z): calcd for $C_{15}H_{17}O_3^+$ $[M + H]^+$: 245.1172, found 245.1172.

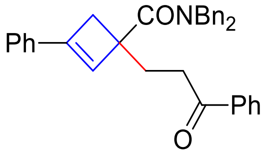

6e

6e, General procedure A (0.1 mmol alkene), yellow oil. 47.0 mg (97% yield). ^1H NMR (600 MHz, $CDCl_3$) δ 7.88 – 7.87 (m, 2H), 7.53 – 7.50 (m, 1H), 7.41 – 7.38 (m, 2H), 7.35 – 7.32 (m, 2H), 7.29 – 7.23 (m, 6H), 7.21 – 7.16 (m, 5H), 7.14 – 7.13 (m, 2H), 6.48 (s, 1H), 4.70 – 4.62 (m, 2H), 4.52 (d, *J* = 16.2 Hz, 1H), 4.34 (d, *J* = 14.4 Hz, 1H), 3.38 (d, *J* = 12.6 Hz, 1H), 3.15 – 3.05 (m, 2H), 2.77 (d, *J*

= 12.6 Hz, 1H), 2.46 – 2.41 (m, 1H), 2.28 – 2.23 (m, 1H). ^{13}C NMR (151 MHz, $CDCl_3$) δ 199.7, 174.4, 145.4, 137.4, 136.9, 136.2, 133.7, 133.2, 129.5, 129.1, 128.7, 128.5, 128.5, 128.2, 127.8, 127.5, 127.1, 125.0, 51.4, 50.2, 47.5, 39.3, 34.7, 31.3. HRMS (ESI, m/z): calcd for $C_{34}H_{31}NNaO_2^+$ [M + Na]$^+$: 508.2247, found 508.2248.

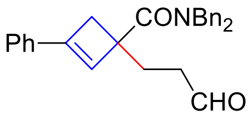

6f

6f, General procedure A (0.1 mmol alkene), yellow oil. 40.8 mg (99% yield). ^{1}H NMR (600 MHz, $CDCl_3$) δ 9.77 (s, 1H), 7.40 – 7.37 (m, 2H), 7.34 – 7.27 (m, 9H), 7.20 – 7.19 (m, 2H), 7.15 – 7.14 (m, 2H), 6.43 (s, 1H), 4.71 – 4.65 (m, 2H), 4.49 (d, *J* = 16.2 Hz, 1H), 4.33 (d, *J* = 14.4 Hz, 1H), 3.36 (d, *J* = 12.6 Hz, 1H), 2.74 (d, *J* = 12.6 Hz, 1H), 2.65 (t, *J* = 7.4 Hz, 2H), 2.35 – 2.30 (m, 1H), 2.16 – 2.11 (m, 1H). ^{13}C NMR (151 MHz, $CDCl_3$) δ 201.5, 174.3, 145.5, 137.4, 136.2, 133.6, 129.2, 128.8, 128.6, 128.6, 128.5, 127.9, 127.6, 127.0, 125.0, 51.1, 50.1, 47.6, 40.2, 39.1, 28.9. HRMS (ESI, m/z): calcd for $C_{28}H_{27}NNaO_2^+$ [M + Na]$^+$: 432.1934, found 432.1934.

2.3. Gram-Scale Synthesis and Derivatization Reactions

2.3.1 Scale-up reaction.

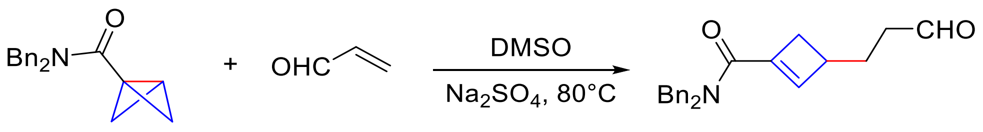

Na_2SO_4 (750.0 mg, 5.3 mmol), **1a** (1.66 g, 6.0 mmol) and acrolein (168.0 mg, 3.0 mmol) in DMSO (3.0 mL) were charged into a 10 mL pressure tube under argon. The reaction tube was then sealed and placed into a metal bath at 80 °C. After stirred for 24 h, the reaction vessel was removed from the metal bath and cooled to ambient temperature. To the reaction mixture was added H_2O (4.0 mL), and the mixture was exacted with Et_2O. The organic layer was concentrated and purified by silica gel chromatography (PE: EA = 5:1) to give the indicated product **3r** (700.0 mg, 70% yield). ^{1}H NMR (600 MHz, $CDCl_3$) δ 9.75 – 9.70 (m, 1H), 7.38 – 7.35 (m, 2H), 7.33 – 7.30 (m, 3H), 7.28 – 7.27 (m, 1H), 7.24 – 7.23 (m, 2H), 7.17 – 7.16 (m, 2H), 6.41 (s, 1H), 4.61 – 4.54 (m, 4H), 2.98 – 2.95 (m, 1H), 2.72 – 2.69 (m, 1H), 2.44 – 2.41 (m, 2H), 2.37 – 2.35 (m, 1H), 1.84 – 1.74 (m, 2H).

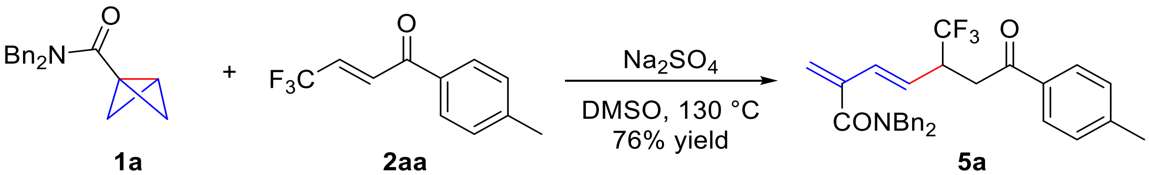

The mixture of **1a** (6.0 mmol, 2.0 equiv), **2aa** (3.0 mmol, 1 equiv), Na_2SO_4 (750 mg, 5.3 mmol), in DMSO (30.0 mL) was stirred at 130 °C in the oil bath for 48 h under argon atmosphere. The reaction vessel was removed from the oil bath and cooled to ambient temperature. To the reaction mixture was added H_2O (50 mL), and the mixture was exacted with Et_2O. The organic layer was concentrated and purified by silica gel chromatography (PE: EA = 5:1) to give the indicated product **5a** (1.074g, 76% yield). ^{1}H NMR (400 MHz, $CDCl_3$) δ 7.76 (d, J = 8.2 Hz, 2H), 7.37 – 7.27 (m, 7H), 7.24 – 7.17 (m, 3H), 7.05 (d, J = 6.8 Hz, 2H), 6.40 (d, J = 16.0 Hz, 1H), 5.59 (dd, J = 15.8, 8.8 Hz, 1H), 5.33 (d, J = 6.8 Hz, 2H), 4.62 (d, J = 14.4 Hz, 1H), 4.47 (d, J = 14.4 Hz, 1H), 4.24 (d, J = 16.0 Hz, 1H), 4.12 (d, J = 16.0 Hz, 1H), 3.80 – 3.69 (m, 1H), 3.22 (dd, J = 17.4, 3.6 Hz, 1H), 3.13 (dd, J = 17.4, 9.0 Hz, 1H), 2.43 (s, 3H).

2.3.2 Derivatization Reactions

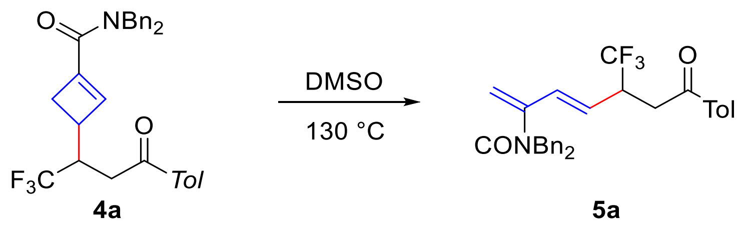

4a (49.0 mg, 0.1 mmol) in DMSO (0.1 mL) were charged into a 10 mL pressure tube under argon. The reaction tube was then sealed and placed into an oil bath at 130 °C. After stirred for 24 h, the reaction vessel was removed from the oil bath and cooled to ambient temperature. To the reaction mixture was added H_2O (4.0 mL), and the mixture was exacted with Et_2O. The organic layer was concentrated and purified by silica gel chromatography (PE: EA = 5:1) to give the indicated product **5a** (48.5 mg, 99% yield). ^{1}H NMR (400 MHz, $CDCl_3$) δ 7.76 (d, J = 8.2 Hz, 2H), 7.37 – 7.27 (m, 7H), 7.24 – 7.17 (m, 3H), 7.05 (d, J = 6.8 Hz, 2H), 6.40 (d, J = 16.0 Hz, 1H), 5.59 (dd, J = 16.0, 8.8 Hz, 1H), 5.34 (s, 1H), 5.33 (s, 1H), 4.62-4.47 (m, 2H), 4.24 - 4.12 (m, 2H), 3.80 – 3.69 (m, 1H), 3.22 (dd, J = 17.4, 3.6 Hz, 1H), 3.13 (dd, J = 17.4, 9.0 Hz, 1H), 2.43 (s, 3H).

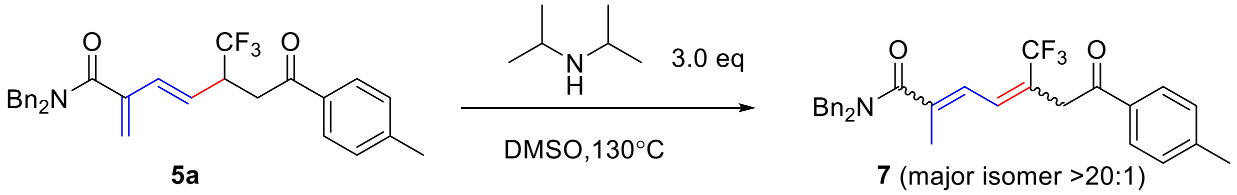

5a (25.0mg, 0.05 mmol) and ^{i}Pr$_2$NH (15.2 mg, 0.15 mmol), and DMSO (1.0 mL) were charged into a pressure tube under argon. The reaction tube was then sealed and placed into an oil bath at 130 °C. After stirred for 24 h, the reaction vessel was removed from the oil bath and cooled to ambient temperature. To the reaction mixture was added H_2O (4.0 mL), and the mixture was exacted with Et_2O. The organic layer was concentrated and purified by silica gel chromatography (PE: EA = 5:1) to give the indicated product **7** (10.3 mg. 42% yield). ^{1}H NMR (400 MHz, $CDCl_3$) δ 7.87 (d, J = 8.4 Hz, 2H), 7.39 – 7.27 (m, 8H), 7.27 – 7.26 (m, 2H), 7.17 (d, J =7.0 Hz, 2H), 6.88 (d, J = 11.6 Hz, 1H), 6.02 (d, J = 11.6 Hz, 1H), 4.91 – 4.11 (m, 4H), 3.94 (s, 2H), 2.42 (s, 3H), 2.04 (s, 3H). ^{13}C NMR (101 MHz, $CDCl_3$) δ 193.5, 171.1, 144.8, 142.1, 136.6, 136.0, 133.7, 130.4, 129.6, 129.0 (d, J = 10.4 Hz), 128.6 (d, J = 21.0 Hz), 127.9 (d, J = 7.1 Hz), 127.5, 123.3 (q, J = 30.4 Hz), 121.9, 50.7, 46.6, 35.2, 21.8, 21.7. ^{19}F NMR (377 MHz, $CDCl_3$) δ -67.56 (s, 3 F). HRMS (ESI, m/z): calcd for

$C_{30}H_{28}F_3NO_2^+$ [M + Na] $^+$: 514.1964, found 514.1964.

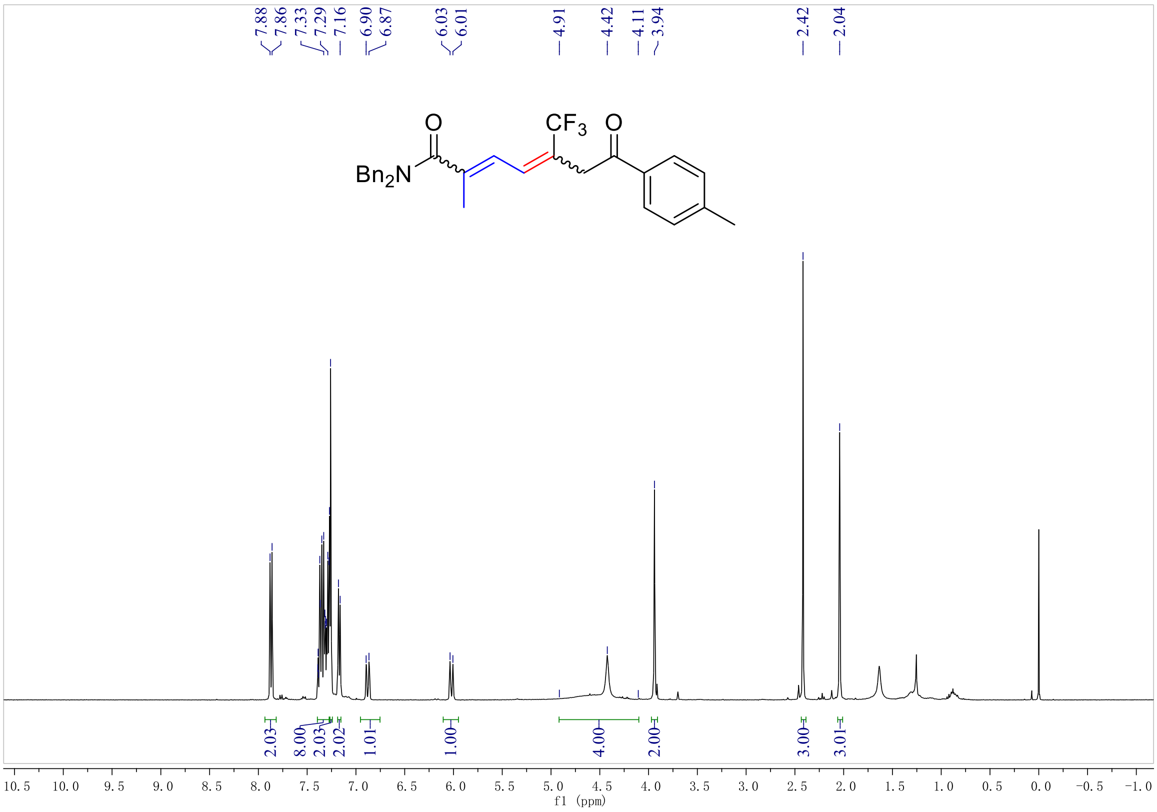

Supplementary Fig. 1 ^{1}H NMR (400 MHz, $CDCl_3$) of **7**

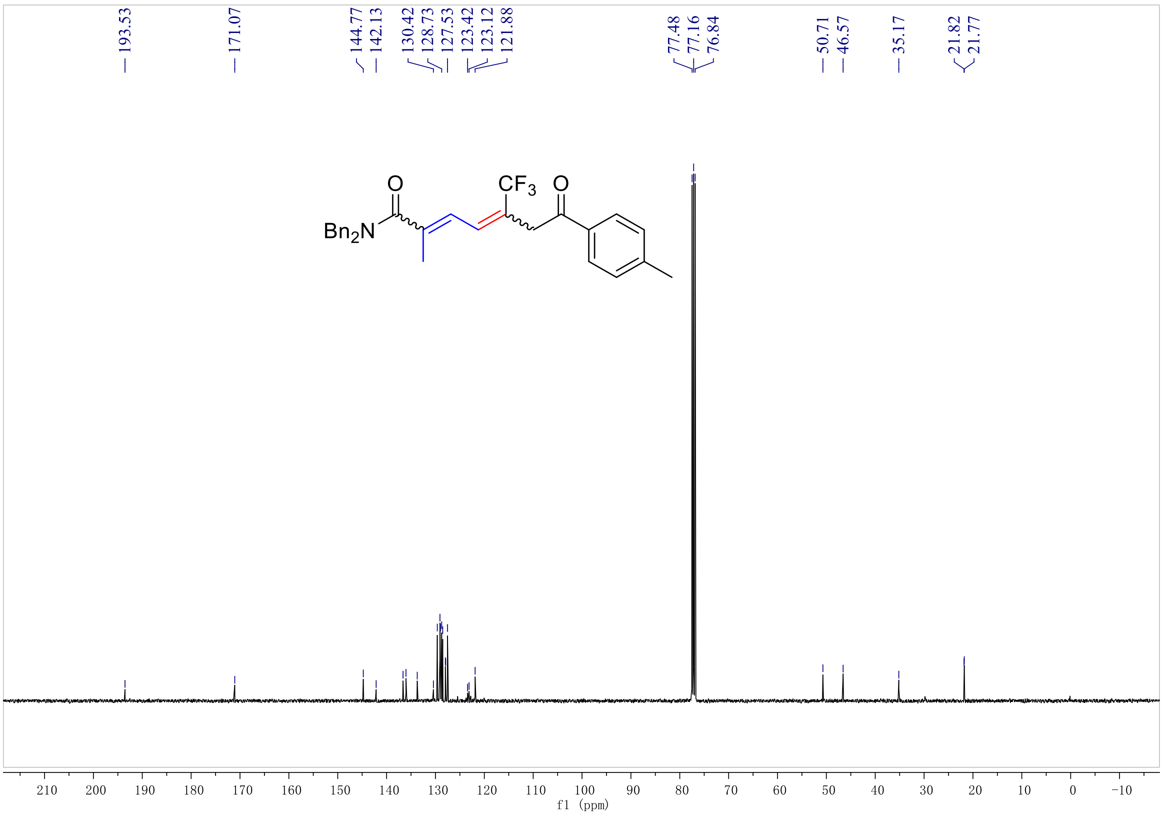

Supplementary Fig. 2 ^{13}C NMR (101 MHz, $CDCl_3$) of **7**

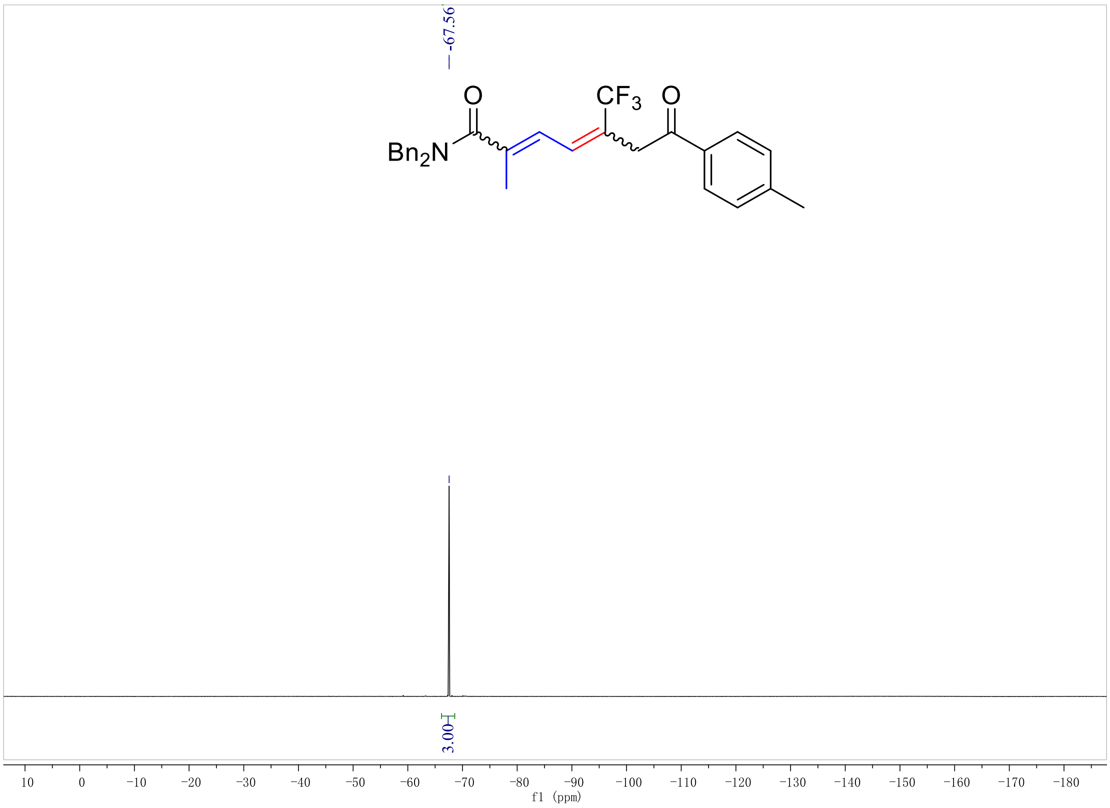

Supplementary Fig. 3 ^{19}F NMR (377 MHz, $CDCl_3$) of **7**

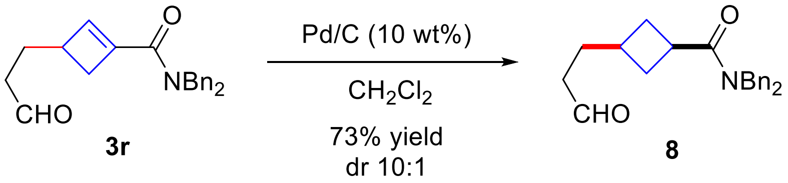

3r (66.6 mg, 0.2 mmol) and CH_2Cl_2 (2.0 mL) were added to a flame dried schlenk tube under hydrogen, the Pd/C (21.2 mg, 10 wt%) was added and stirred at room temperature. After stirred for 2 h, the reaction mixture was filtered through a pad of celite eluting with ethyl acetate. The crude mixture was concentrated and purified by silica gel chromatography (PE: EA = 2:1) to give the product **8** (49.2 mg, 73% yield, dr = 10: 1). ^{1}H NMR (600 MHz, $CDCl_3$) δ major 9.74 – 9.73 (m, 1H), 7.37 – 7.34 (m, 2H), 7.32 – 7.28 (m, 3H), 7.27 – 7.26 (m, 1H), 7.19 – 7.17 (m, 2H), 7.12 – 7.11 (m, 2H), 4.56 (s, 2H), 4.36 (s, 2H), 3.18 – 3.12 (m, 1H), 2.36 – 2.33 (m, 2H), 2.26 – 2.21 (m, 2H), 2.20 – 2.14 (m, 1H), 2.07 – 2.02 (m, 2H), 1.77 – 1.73 (m, 2H). δ minor 9.76 – 9.75 (m, 1H), 4.58 (s, 2H), 4.31 – 4.30 (m, 2H), 4.35 – 4.30 (m, 1H), 2.57 –2.52 (m, 2H). ^{13}C NMR (101 MHz, $CDCl_3$) δ 202.4, 174.9, 137.6, 136.8, 129.0, 128.7, 128.4, 127.7, 127.5, 126.6. 49.5, 48.2, 41.5, 33.5, 31.4, 30.9, 28.9. HRMS (ESI, m/z): calcd for $C_{22}H_{25}NNaO_2^+$ [M + Na] $^+$: 358.1778, found 358.1780.

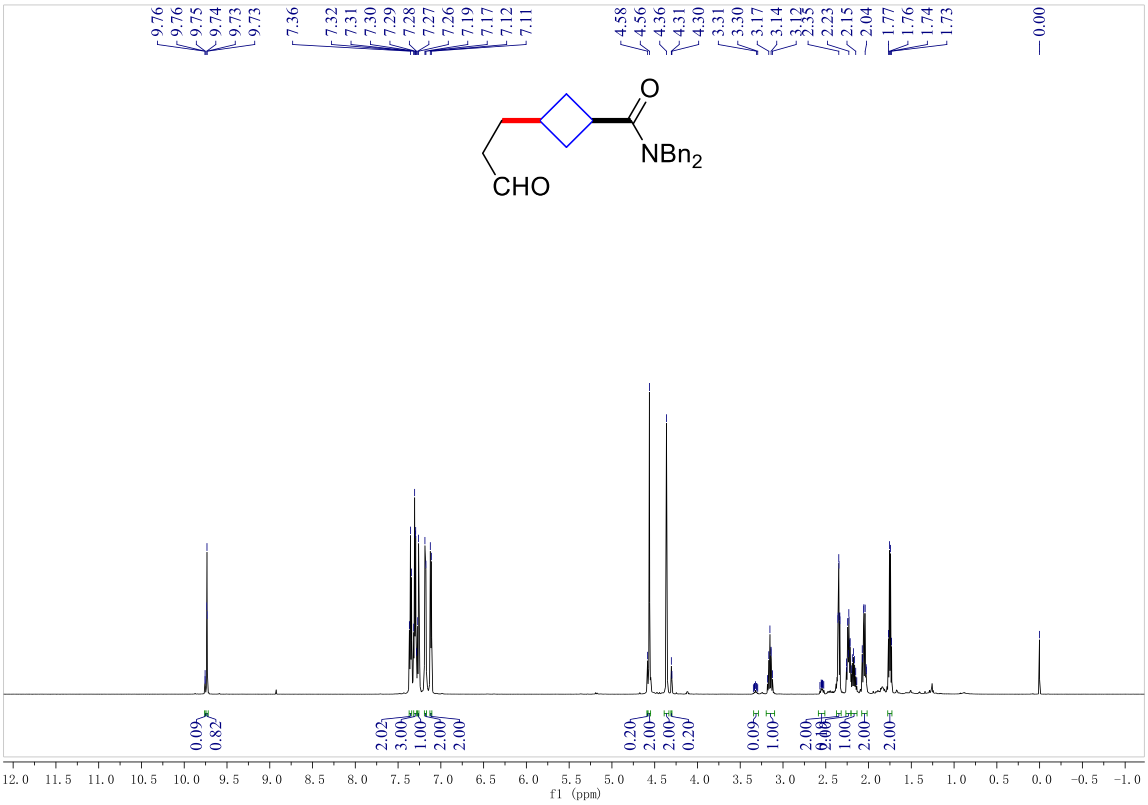

Supplementary Fig. 4 ^{1}H NMR (600 MHz, $CDCl_3$) of **8**

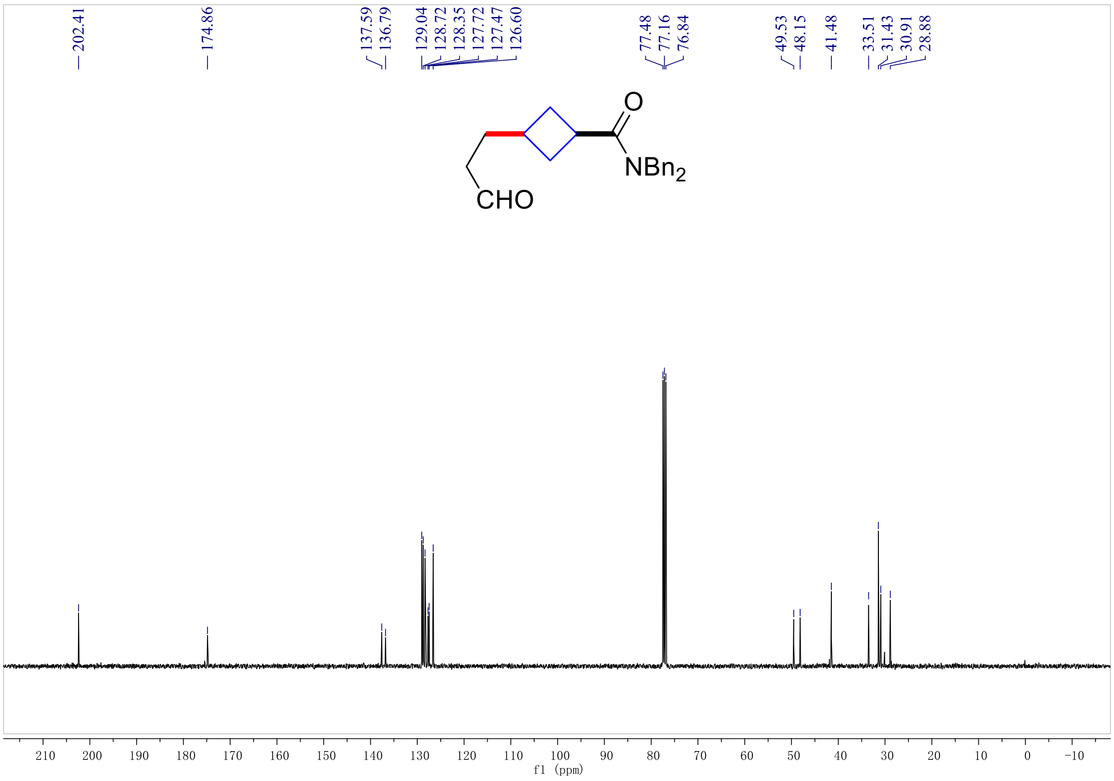

Supplementary Fig. 5 ^{13}C NMR (101 MHz, $CDCl_3$) of **8**

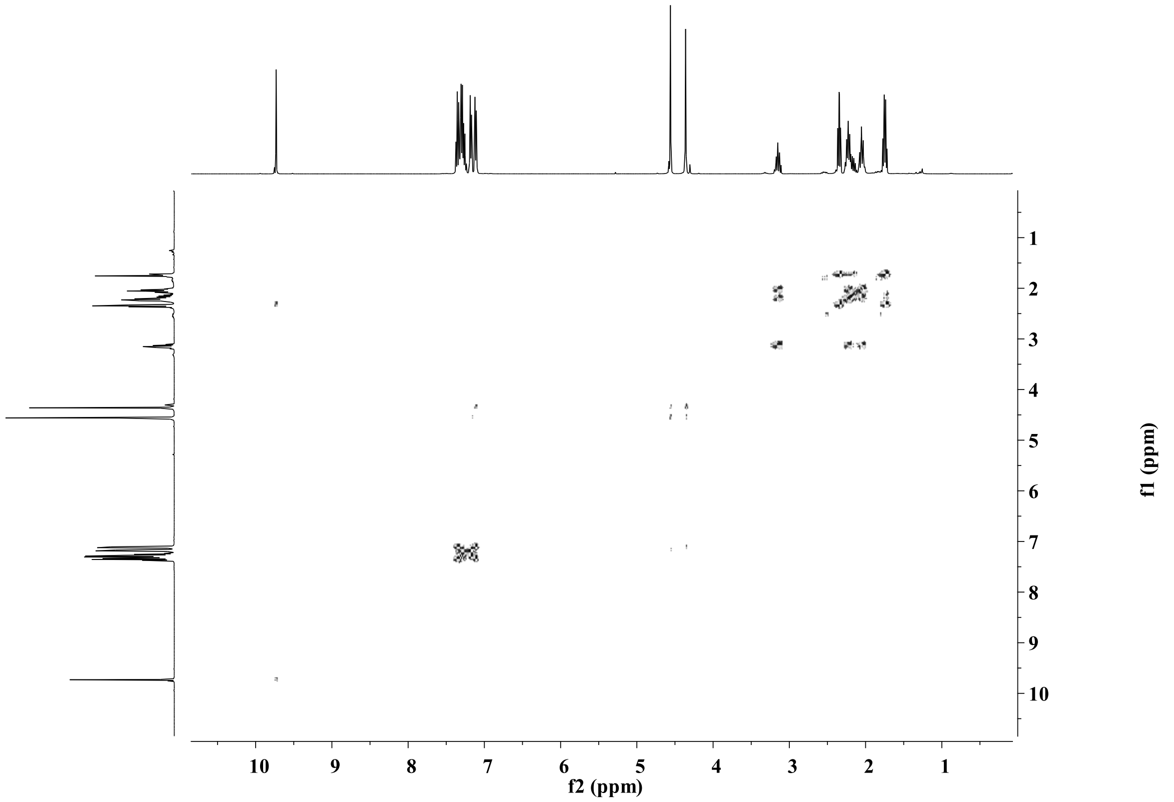

Supplementary Fig. 6 H-H COSY of **8** (400 MHz, $CDCl_3$)

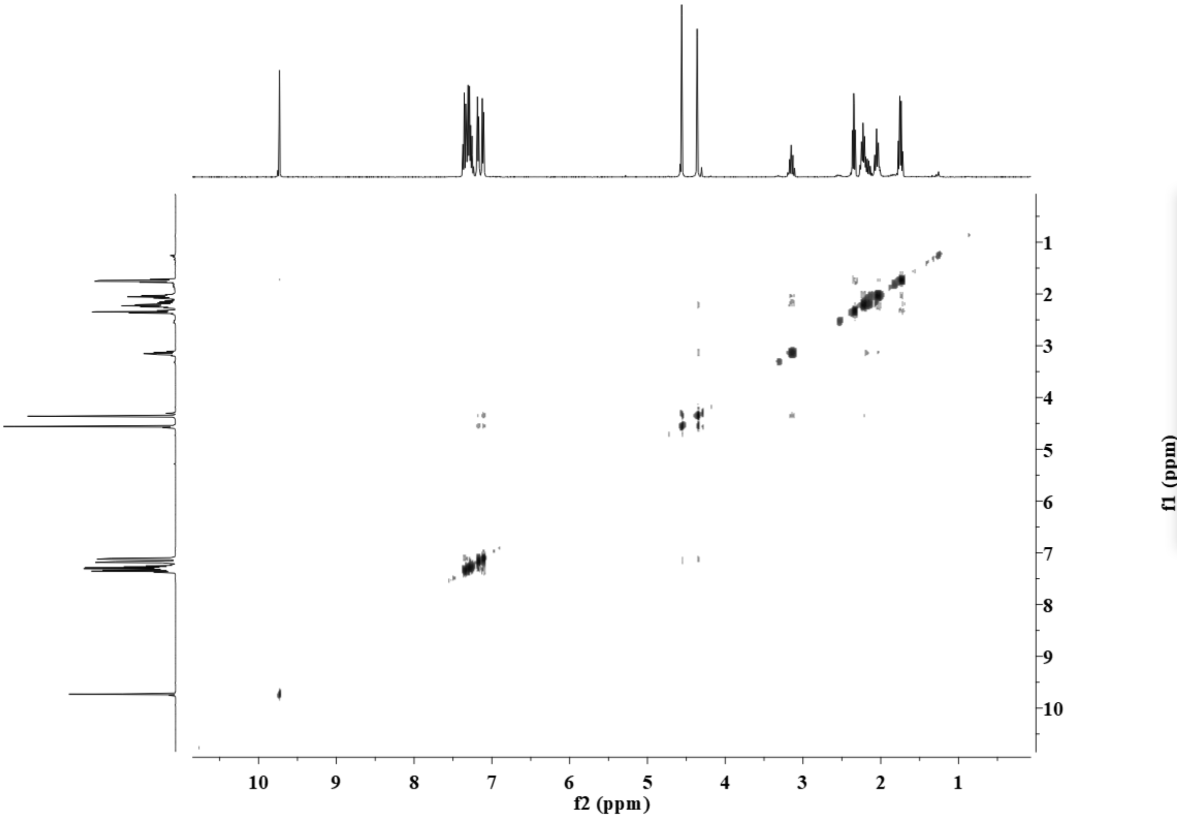

Supplementary Fig. 7 NOESY - 2D ^{1}H NMR of **8** (400 MHz, $CDCl_3$)

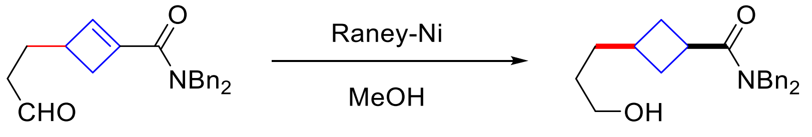

3r **9**

68% yield

dr 17:1

3r (16.7 mg, 0.05 mmol) and MeOH (0.5 mL) were added to a flame dried schlenk tube under hydrogen, the Raney-Ni was added and stirred at room temperature. After stirred for 5 h, the reaction mixture was filtered through a pad of celite eluting with ethyl acetate. The crude mixture was concentrated and purified by silica gel chromatography (PE: EA = 2:1) to give the product **9** (11.4 mg, 68% yield, dr =17: 1). ^{1}H NMR (400 MHz, $CDCl_3$) δ major 7.37 – 7.34 (m, 2H), 7.32 – 7.28 (m, 3H), 7.27 – 7.26 (m, 1H), 7.19 – 7.17 (m, 2H), 7.13 – 7.12 (m, 2H), 4.56 (s, 2H), 4.37 (s, 2H), 3.62 – 3.60 (m, 2H), 3.18 – 3.12 (m, 1H), 2.26 – 2.22 (m, 2H), 2.20 – 2.11 (m, 1H), 2.07 – 2.02 (m, 2H), 1.51 – 1.45 (m, 4H). δ minor 4.58-4.56 (m, 2H), 4.3 – 4.31 (m, 2H). ^{13}C NMR (101 MHz, $CDCl_3$) δ 175.1, 137.6, 136.8, 129.0, 128.7, 128.3, 127.7, 127.5, 126.6, 63.05, 49.4, 48.0, 33.8, 32.8, 31.8, 31.4, 30.3. HRMS (ESI, m/z): calcd for $C_{22}H_{27}NNaO_2^+$ [M + Na] $^+$: 360.1934, found 360.1933.

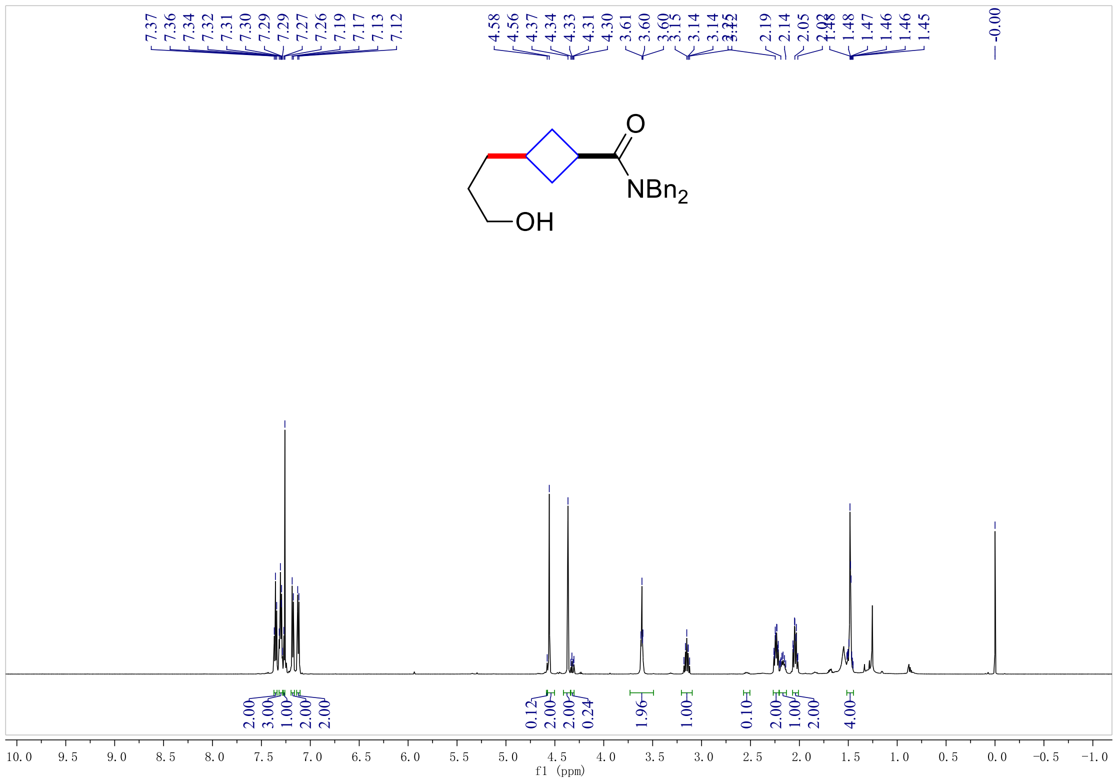

Supplementary Fig. 8 ^{1}H NMR (400 MHz, $CDCl_3$) of **9**

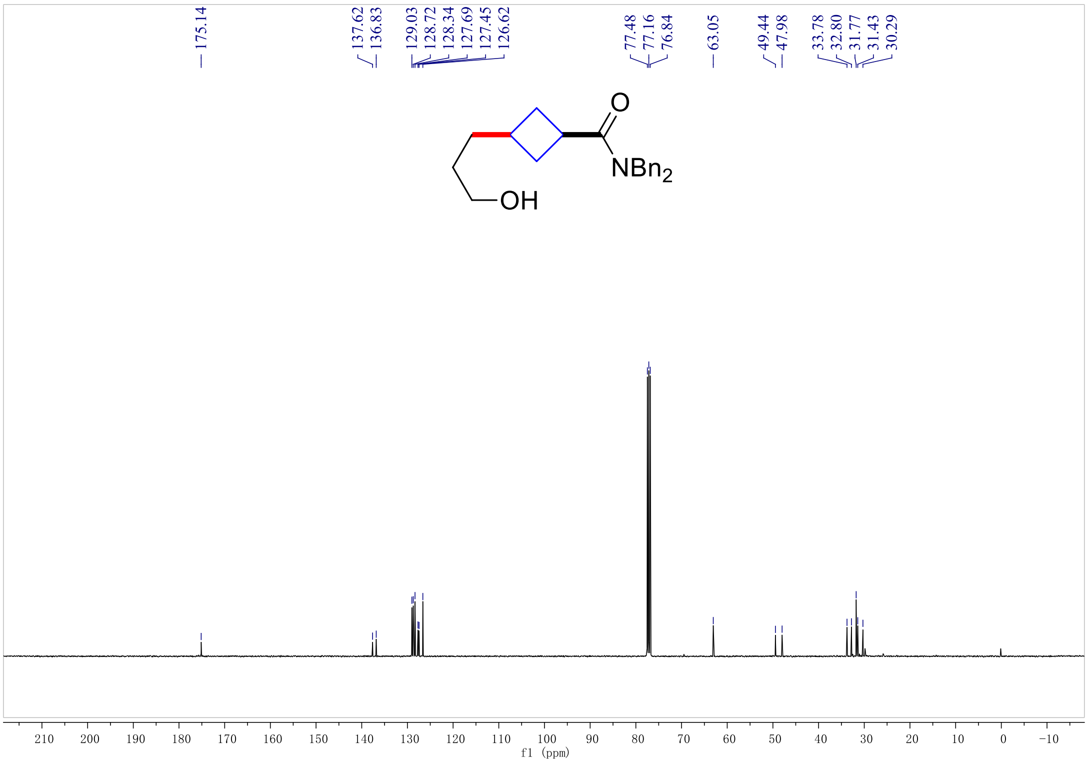

Supplementary Fig. 9 ^{13}C NMR (101 MHz, $CDCl_3$) of **9**

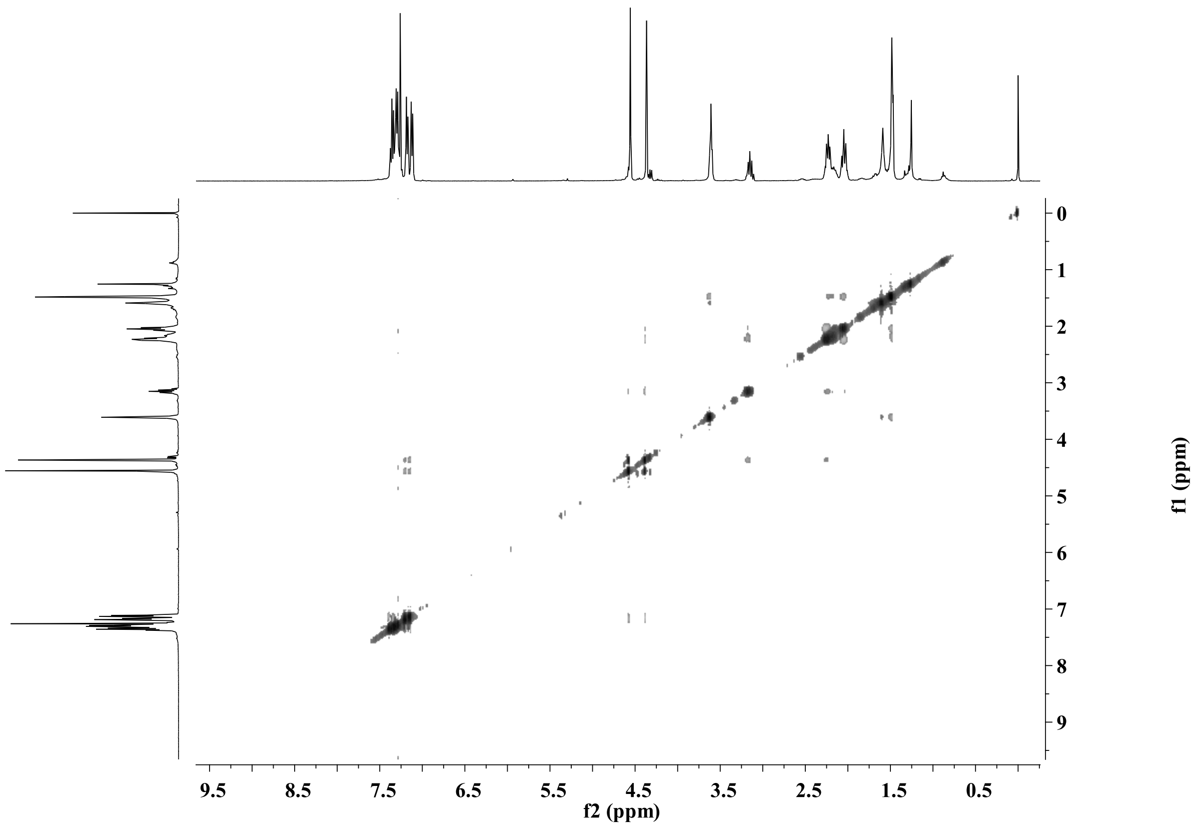

Supplementary Fig. 10 H-H COSY of **9** (400 MHz, $CDCl_3$)

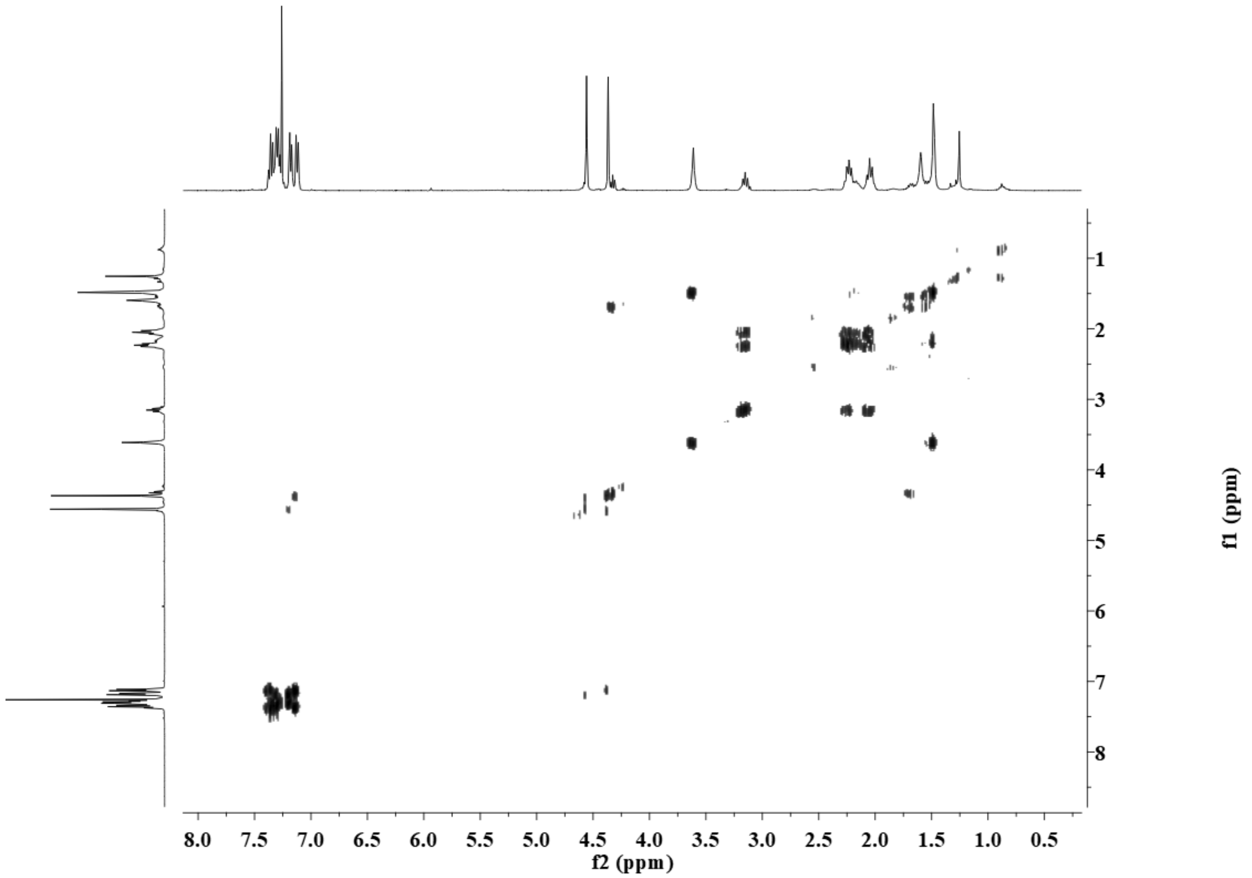

Supplementary Fig. 11 NOESY - 2D ^{1}H NMR of **9** (400 MHz, $CDCl_3$)

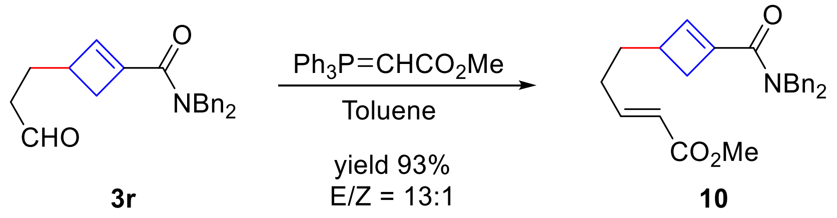

3r (16.7 mg, 0.05 mmol), Ph_3P=$CHCO_2Me$ (16.7 mg, 0.05 mmol) and toluene (0.5 mL) was added to a flame dried schlenk tube under argon. After stirred for 3 h at room temperature, the reaction mixture was purified by silica gel chromatography (PE: EA = 2:1) to give the indicated product **10**. (18.1 mg, 93% yield, E/Z = 13:1). ^{1}H NMR (400 MHz, $CDCl_3$) δ 7.39 – 7.35 (m, 2H), 7.32 – 7.27 (m, 4H), 7.25 – 7.23 (m, 2H), 7.18 – 7.16 (m, 2H), 6.96 – 6.89 (m, 1H), 6.44 (s, 1H), 5.80 (dt, J = 15.6, 1.4 Hz, 1H), 4.62 – 4.53 (m, 4H), 3.71 (s, 3H), 2.98 – 2.94 (m, 1H), 2.72 – 2.67 (m, 1H), 2.37 (dd, J = 13.2, 1.4 Hz, 1H), 2.23 – 2.17 (m, 2H), 1.67 – 1.57 (m, 2H). ^{13}C NMR (101 MHz, $CDCl_3$) δ 167.1, 164.7, 148.9, 144.6, 139.6, 137.0, 136.8, 129.1, 128.7, 128.7, 127.8, 127.6, 126.7, 121.4, 51.6, 50.2, 48.2, 39.3, 37.4, 31.9, 30.5. HRMS (ESI, m/z): calcd for $C_{25}H_{27}NNaO_3^+$ [M + Na] $^+$: 412.1883,

found 412.1884.

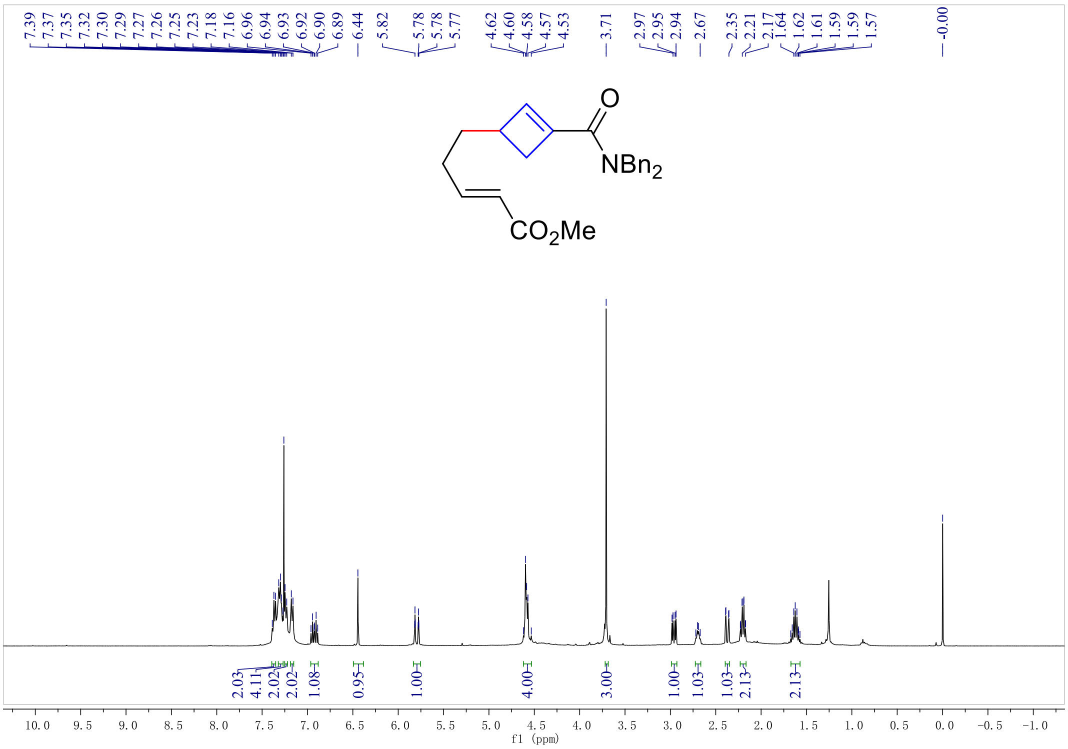

Supplementary Fig. 12 ^{1}H NMR (400 MHz, $CDCl_3$) of **10**

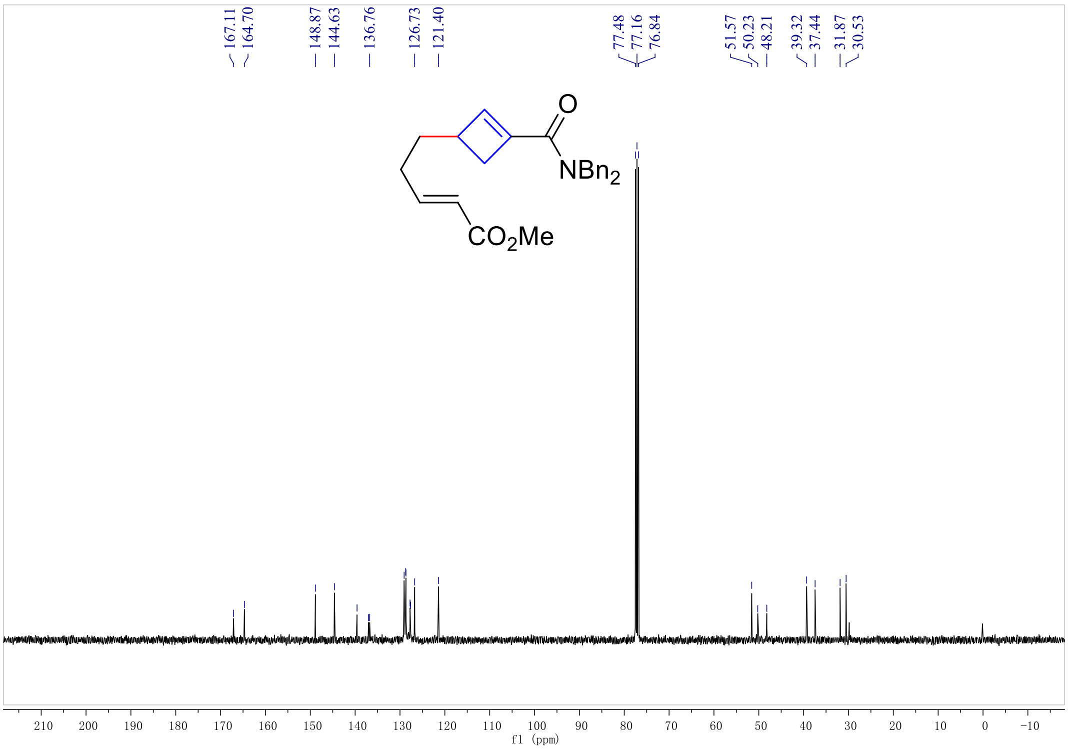

Supplementary Fig. 13 ^{13}C NMR (101 MHz, $CDCl_3$) of **10**

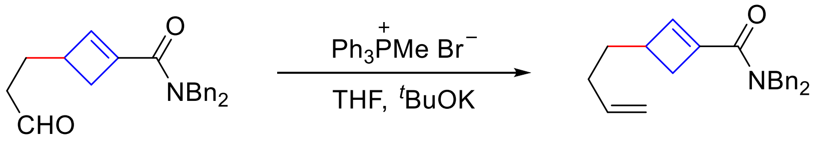

3r **11**

^{t}BuOK (16.8 mg, 0.15 mmol) was added to a solution of Ph_3PMeBr (60.6 mg, 0.15 mmol) in THF (1.5 mL) under argon. After stirred for 30 min at room temperature, **3r** (33.3 mg, 0.1 mmol) was added to the reaction mixture. The reaction mixture was stirred for 4 h at room temperature, then warmed to 60 °C and stirred for an additional 12 h. The mixture quenched with sat. aq. NH_4Cl (2 mL). The mixture was partitioned between EtOAc (10 mL) and water (10 mL). the phases were separated, and the aqueous phase was extracted with EtOAc (10 mL×2), after combined organic phases and dried by Na_2SO_4. The organic phases was concentrated under reduced pressure and purified by silica gel chromatography (PE: EA = 2:1) to give the indicated product **11**. (27.0 mg, 81% yield). ^{1}H NMR (600 MHz, $CDCl_3$) δ 7.38 – 7.36 (m, 2H), 7.33 – 7.31 (m, 3H), 7.28 – 7.27 (m, 1H), 7.25 – 7.24 (m, 2H), 7.19 – 7.18 (m, 2H), 6.49 (s, 1H), 5.81 – 5.74 (m, 1H), 5.00 – 4.97 (m, 1H), 4.93 (d, *J* = 10.2, 1H), 4.61 – 4.55 (m, 4H), 2.96 (dd, *J* = 13.2, 4.2 Hz, 1H), 2.70 (dd, *J* = 12.0, 7.2 Hz, 1H), 2.38 (dd, *J* = 13.2, 1.2 Hz, 1H), 2.07 – 2.04 (m, 2H), 1.61 – 1.51 (m, 2H). ^{13}C NMR (101 MHz, $CDCl_3$) δ 164.8, 145.3, 139.4, 138.5, 137.2, 136.9, 129.1, 128.7, 127.7, 127.6, 126.8, 114.9, 50.2, 48.1, 39.6, 37.6, 32.8, 32.1. HRMS (ESI, m/z): calcd for $C_{23}H_{25}NNaO^+$ [M + Na] $^+$: 354.1828, found 354.1816.

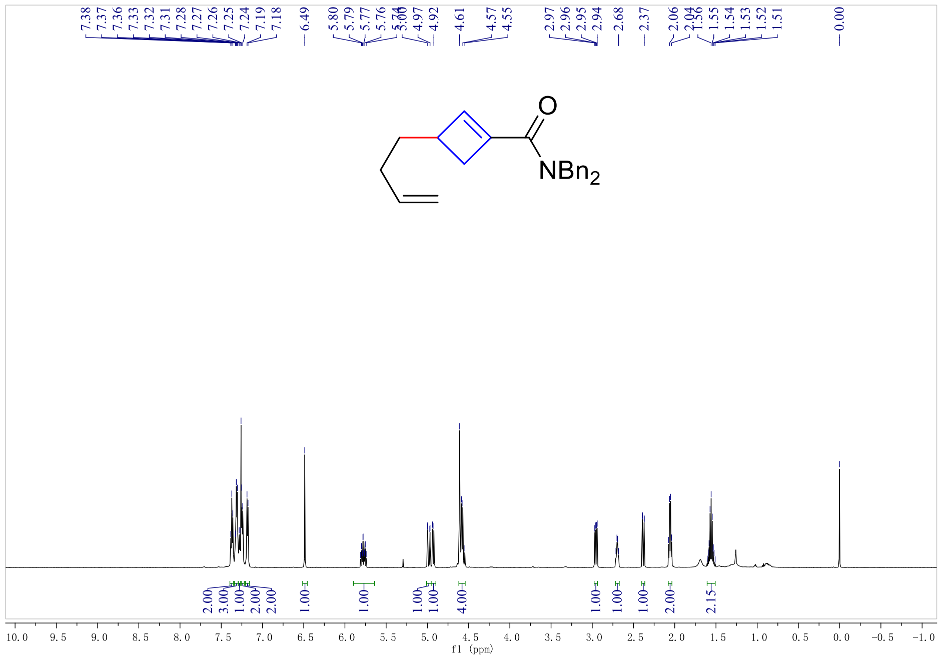

Supplementary Fig. 14 ^{1}H NMR (600 MHz, $CDCl_3$) of **11**

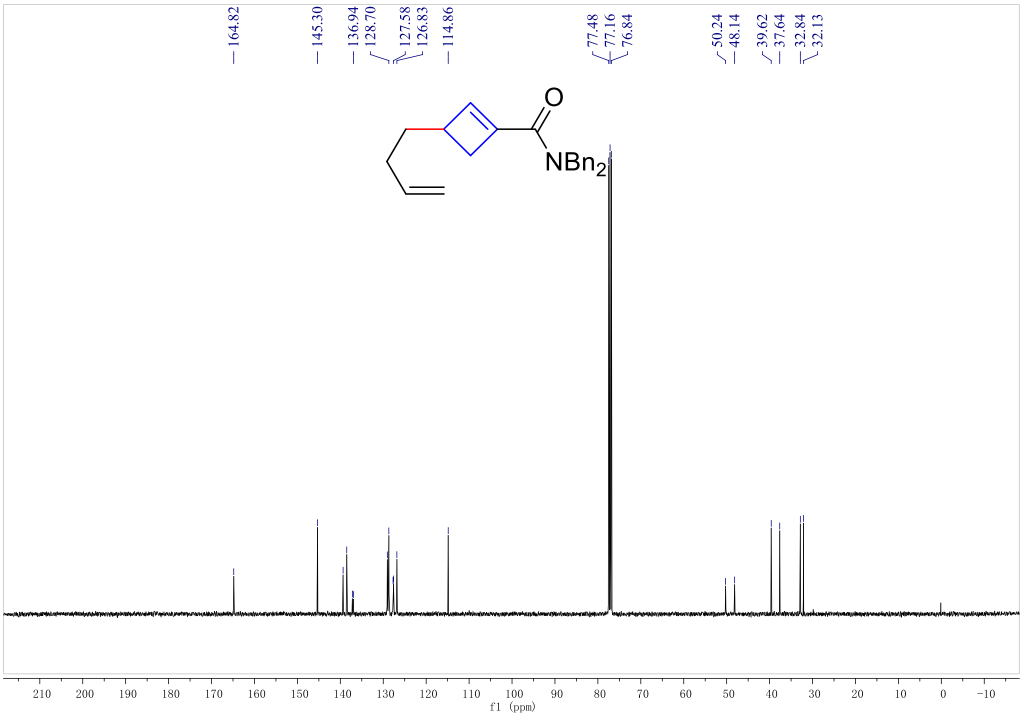

Supplementary Fig. 15 ^{13}C NMR (101 MHz, $CDCl_3$) of **11**

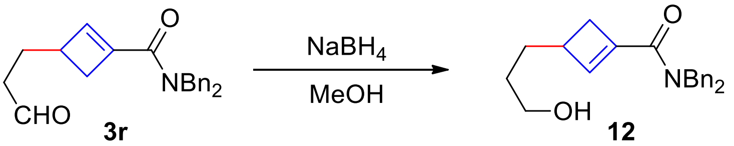

3r (66.6 mg, 0.2 mmol) and MeOH (0.2 mL) was added to a flame dried schlenk tube under air. The $NaBH_4$ (15.2 mg, 0.4 mmol) was added slowly at 0 °C. After stirred for 4 h at room temperature, H_2O (1.0 mL) was added, and then the aqueous phase was washed with CH_2Cl_2 (3×10 mL), after combined organic phases and dried by Na_2SO_4. The organic phases was concentrated under reduced pressure and purified by silica gel chromatography (PE: EA = 2:1) to give the indicated product **12**. (57.0 mg, 85% yield). ^{1}H NMR (400 MHz, $CDCl_3$) δ 7.38 – 7.35 (m, 2H), 7.33 – 7.27 (m, 4H), 7.25 – 7.23 (m, 2H), 7.18 – 7.16 (m, 2H), 6.47 (d, J = 0.8 Hz 1H), 4.60 – 4.57 (m, 4H), 3.63 – 3.60 (m, 2H), 2.96 (dd, J = 13.2, 4.4 Hz, 1H), 2.71 – 2.67 (m, 1H), 2.37 (dd, J = 13.2, 1.4 Hz, 1H), 1.60 – 1.48 (m, 4H). ^{13}C NMR (151 MHz, $CDCl_3$) δ 164.8, 145.4, 139.2, 137.0, 136.7, 129.0, 128.6, 128.6, 127.7, 127.5, 126.7, 62.6, 50.1, 48.1, 39.7, 37.5, 30.9, 29.7. HRMS (ESI, m/z): calcd for $C_{22}H_{25}NNaO_2^{+}$ [M + H] $^{+}$: 336.1958, found 336.1953.

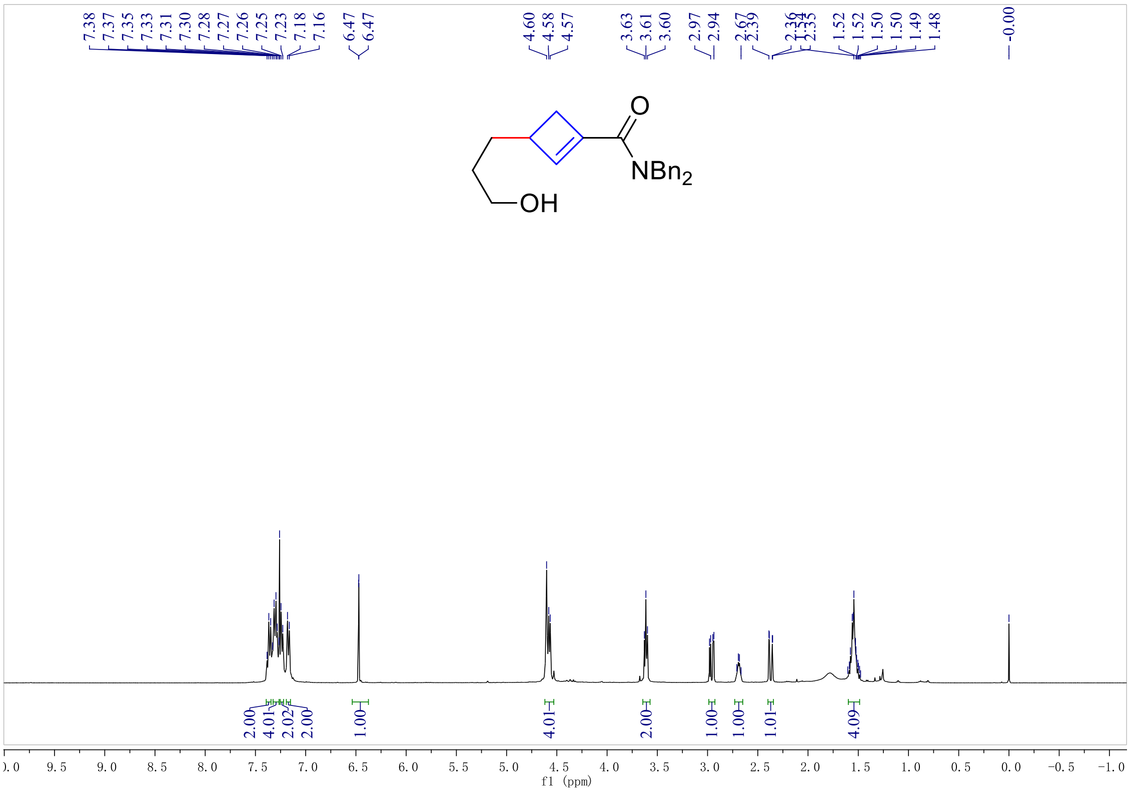

Supplementary Fig. 16 ^{1}H NMR (400 MHz, $CDCl_3$) of **12**

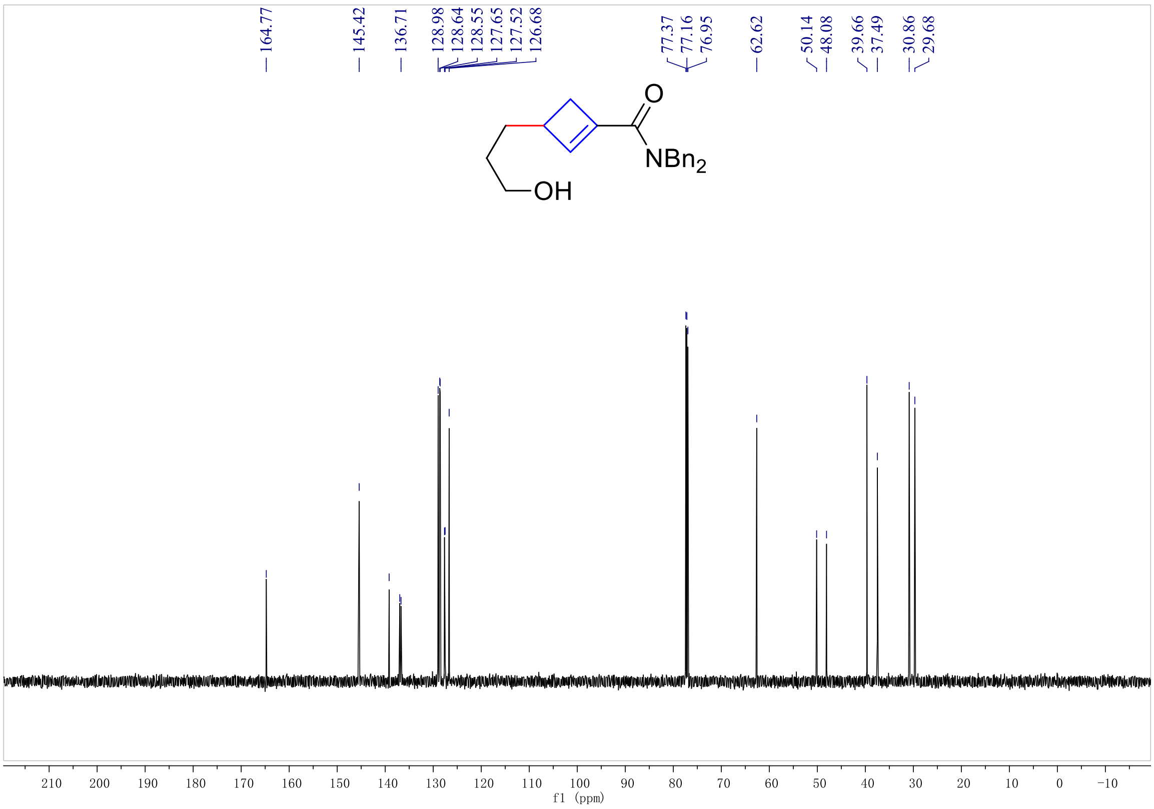

Supplementary Fig. 17 ^{13}C NMR (151 MHz, $CDCl_3$) of **12**

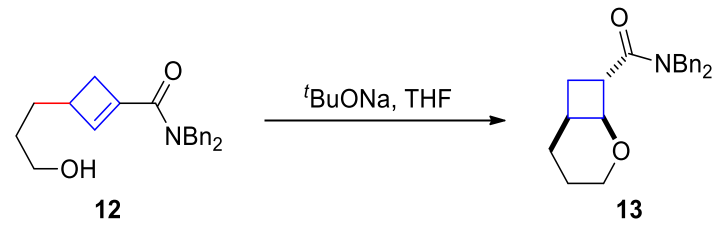

12 (16.8 mg, 0.05 mmol), ^{t}BuONa (9.8 mg, 0.1 mmol) and THF (0.1 mL) was added to a flame dried schlenk tube under argon. After stirred for 24 h at room temperature, the reaction mixture was purified by silica gel chromatography (PE: EA = 2:1) to give the indicated product **13** (15.1 mg, 90.0% yield, dr = 6:1). ^{1}H NMR (600 MHz, $CDCl_3$) δ major: 7.38 – 7.35 (m, 2H), 7.31 – 7.29 (m, 3H), 7.26 – 7.25 (m, 1H), 7.20 – 7.19 (m, 2H), 7.17 – 7.16 (m, 2H), 4.87 (d, *J* = 15.0 Hz, 1H), 4.67 (d, *J* = 16.8 Hz, 1H), 4.44 – 4.42 (m, 1H), 4.34 – 4.28 (m, 2H), 3.74 – 3.70 (m, 1H), 3.68 – 3.65 (m, 1H), 3.51 – 3.47 (m, 1H), 2.44 – 2.39 (m, 1H), 2.22 – 2.18 (m, 1H), 2.04 – 2.00 (m, 1H), 1.67 – 1.64 (m, 1H), 1.51 – 1.46 (m, 2H), 1.45 – 1.40 (m, 1H). δ minor: 5.06 (d, *J* = 15.0 Hz, 1H), 4.54 (d, *J* = 16.8 Hz, 1H), 4.46 (d, *J* = 5.4 Hz, 1H), 4.14 (d, *J* = 15.0 Hz, 1H), 3.90 (d, *J* = 11.4 Hz, 1H), 3.22 – 3.16 (m, 2H), 2.95 – 2.90 (m, 1H), 2.31 – 2.26 (m, 1H), 1.96 – 1.90 (m, 1H), 1.62 – 1.61 (m, 1H), 1.38 – 1.35 (m, 1H), 1.03 – 1.01 (m, 1H), 0.89 – 0.87 (m, 1H), 0.76 – 0.73 (m, 1H). ^{13}C NMR (151 MHz, $CDCl_3$) δ major: 173.8, 137.6, 137.1, 129.0, 128.7, 128.3, 127.6, 127.4, 126.6, 72.5, 63.0, 49.3, 48.3, 40.3, 30.5, 26.1, 24.2, 23.1. HRMS (ESI, m/z): calcd for $C_{22}H_{25}NNaO_2^+$ [M + Na] $^+$: 358.1778, found 358.1782.

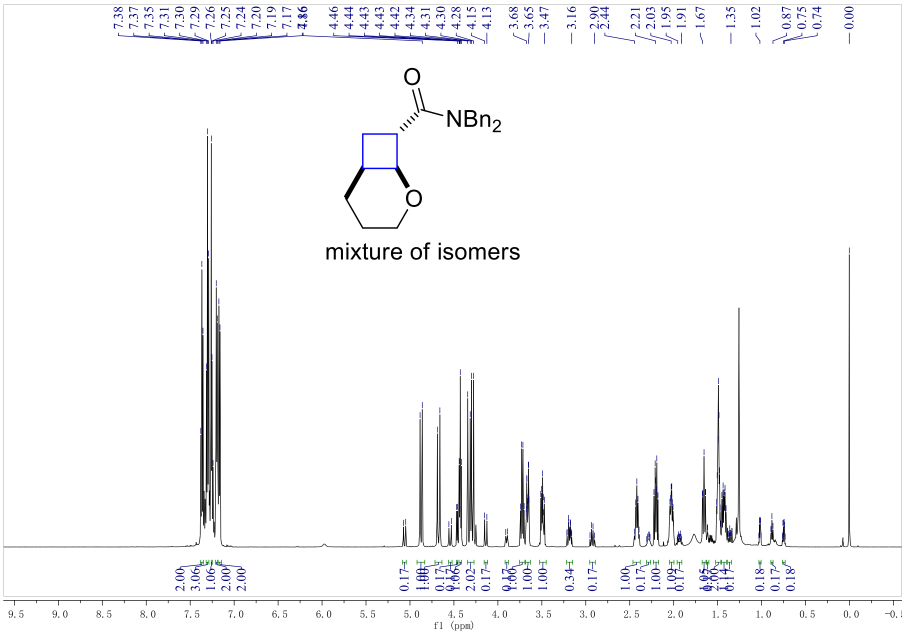

Supplementary Fig. 18 ^{1}H NMR (600 MHz, $CDCl_3$) of **13** (mixture of isomers)

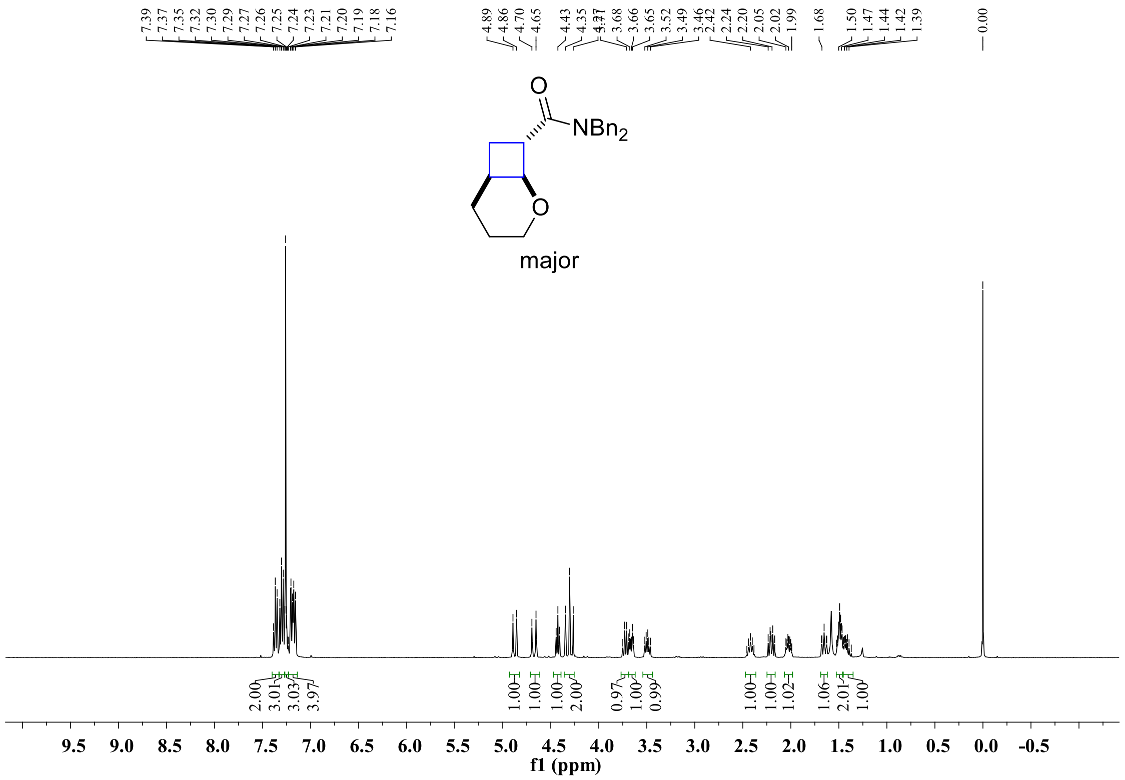

Supplementary Fig. 19 ^{1}H NMR (400 MHz, $CDCl_3$) of **13** (major)

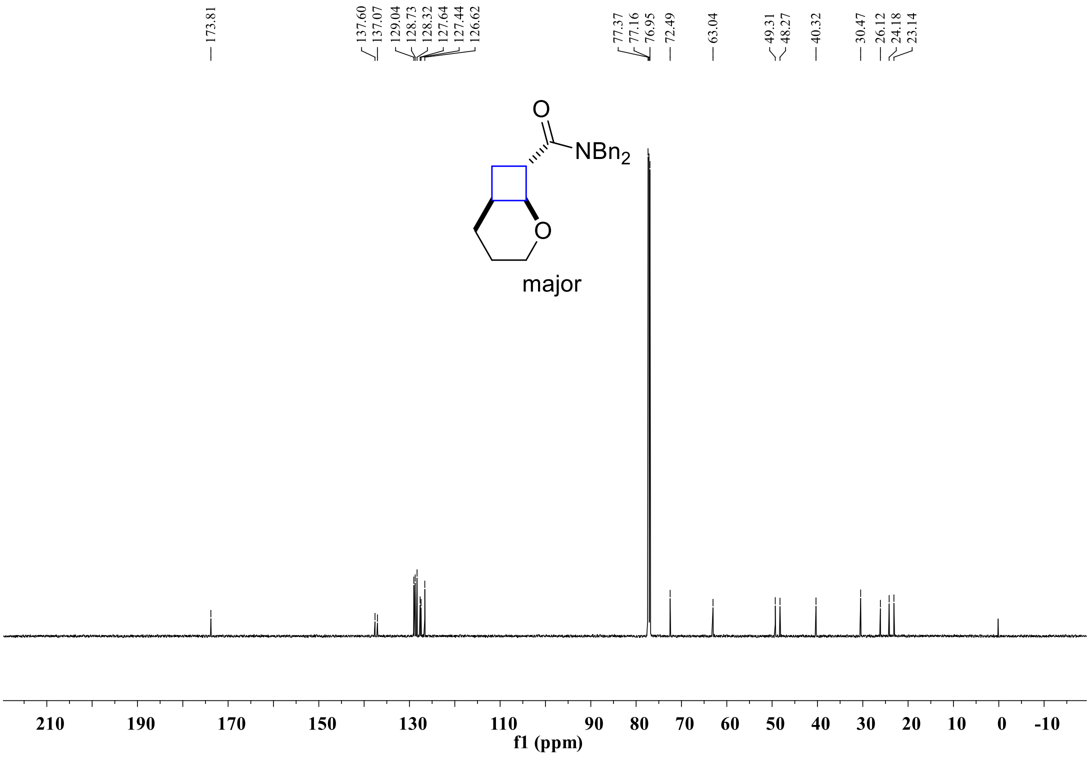

Supplementary Fig. 20 ^{13}C NMR (101 MHz, $CDCl_3$) of **13** (major)

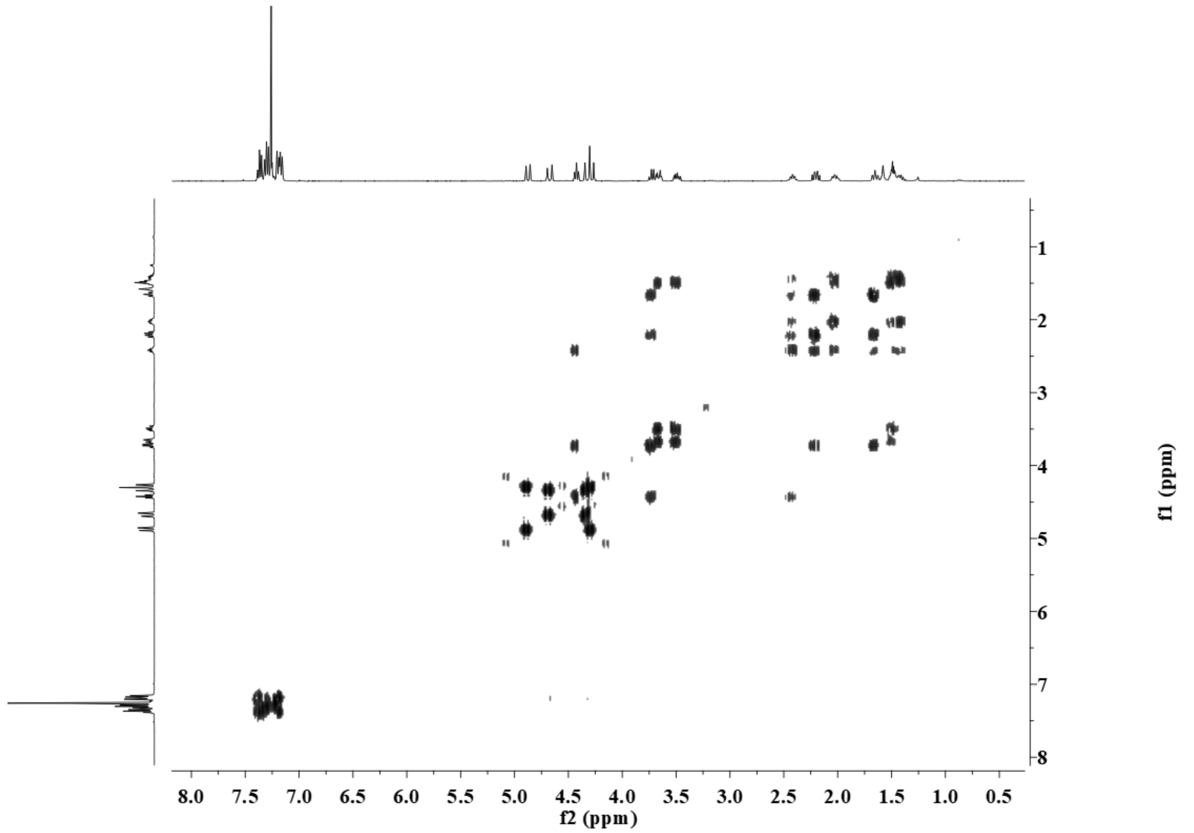

Supplementary Fig. 21 H-H COSY of **13** (400 MHz, $CDCl_3$)

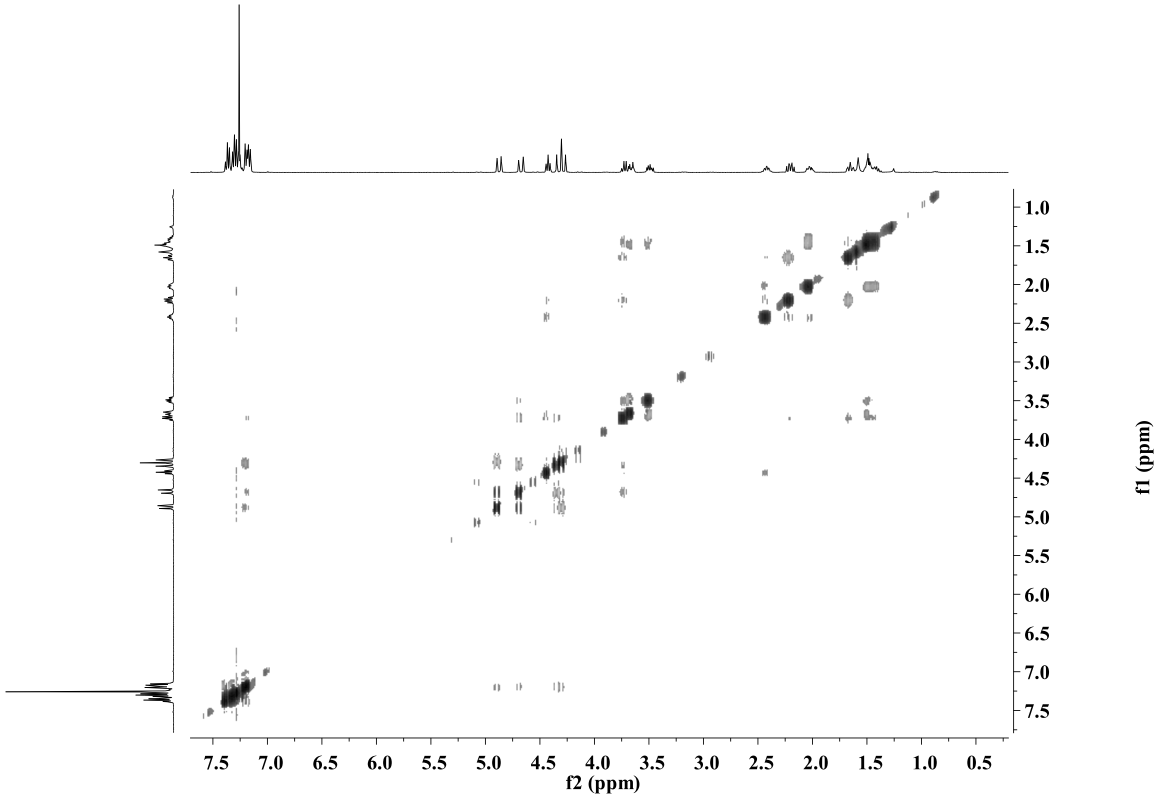

Supplementary Fig. 22 NOESY - 2D ^{1}H NMR of **13** (400 MHz, $CDCl_3$)

2.4. Mechanistic Studies

2.4.1 Starting Material Synthesis

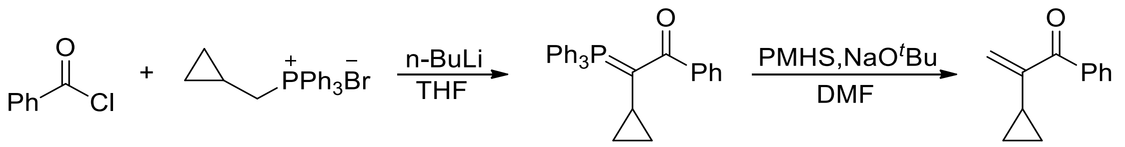

2y

The corresponding phosphoniumbromide salt (11.9 g, 30 mmol, 2.1 equiv) in dry THF (120 mL) was treated with n-BuLi (38.7 mL, 62 mmol, 1.6 M solution in hexane, 2.1 equiv) and the resulting mixture was stirred for 30 min at 0 °C. To the above reaction mixture was added a solution of the corresponding acyl chloride (4.2 g, 30 mmol, 1 equiv) in THF (3 mL), and the reaction mixture was stirred at room temperature for 3 hours. Water was added then the mixture was extracted with DCM. The organic layer was washed with brine and dried with anhydrous Na_2SO_4. Evaporation of the solvent afforded the crude phosphorus ylide which was used without further purification.

An oven-dried Schlenk tube was charged with the crude phosphorus ylide and NaO^{t}Bu (0.28 mg, 3.0 mmol, 0.10 equiv). Then the tube was evacuated and back-filled with carbon dioxide (CO_2) for 3 times. Anhydrous DMF (60 mL) and PMHS (10.4 g, 90 mmol, 6.0 equiv) were added via syringe. Then the Schlenk tube was sealed at atmospheric pressure of CO_2 (1 atm) and the resulting mixture was stirred at 100 °C for 12 h. The mixture was cooled to room temperature and concentrated under reduced pressure. The residue was purified by column chromatography to give the pure desired product **2y**. (855.3 mg, 17% yield). ^{1}H NMR (600 MHz, $CDCl_3$) δ 7.77 (d, J = 7.2 Hz, 2H), 7.53 (t, J = 7.2 Hz, 1H), 7.43 (t, J = 7.8 Hz, 2H), 5.50 (s, 1H), 5.41 (s, 1H), 1.90 – 1.85 (m, 1H), 0.88 – 0.85 (m, 2H), 0.62 – 0.60 (m, 2H). ^{13}C NMR (151 MHz, $CDCl_3$) δ 198.4, 150.1, 137.8, 132.4, 129.7, 128.3, 120.2, 12.7, 7.9.

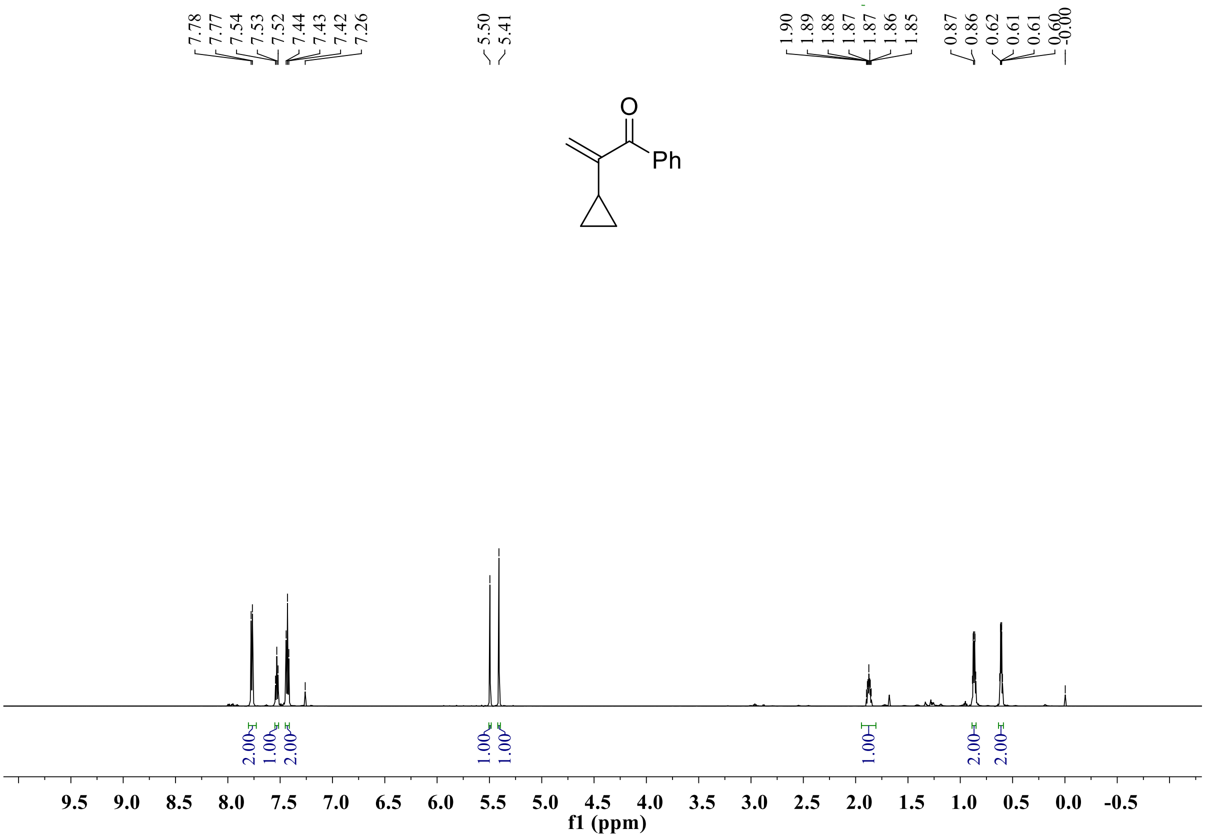

Supplementary Fig. 23 ^{1}H NMR (600 MHz, $CDCl_3$) of **2y**

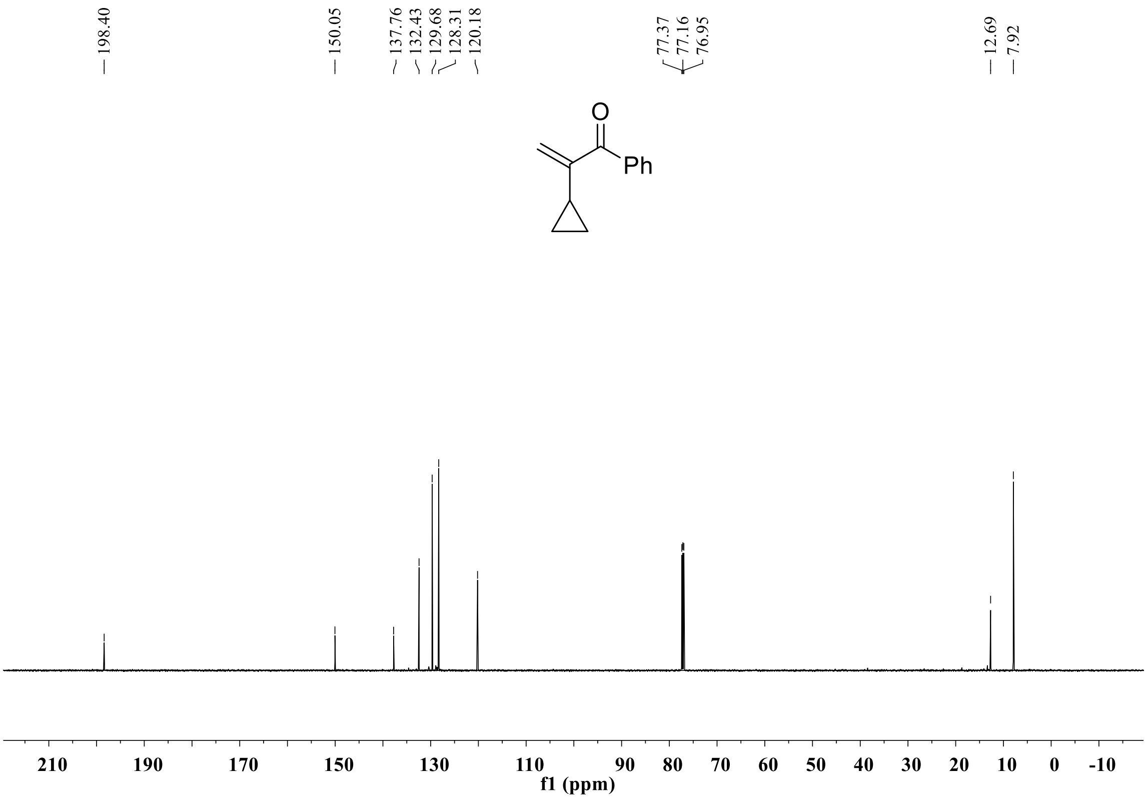

Supplementary Fig. 24 ^{13}C NMR (151 MHz, $CDCl_3$) of **2y**

2.4.2 Deuterium experiments

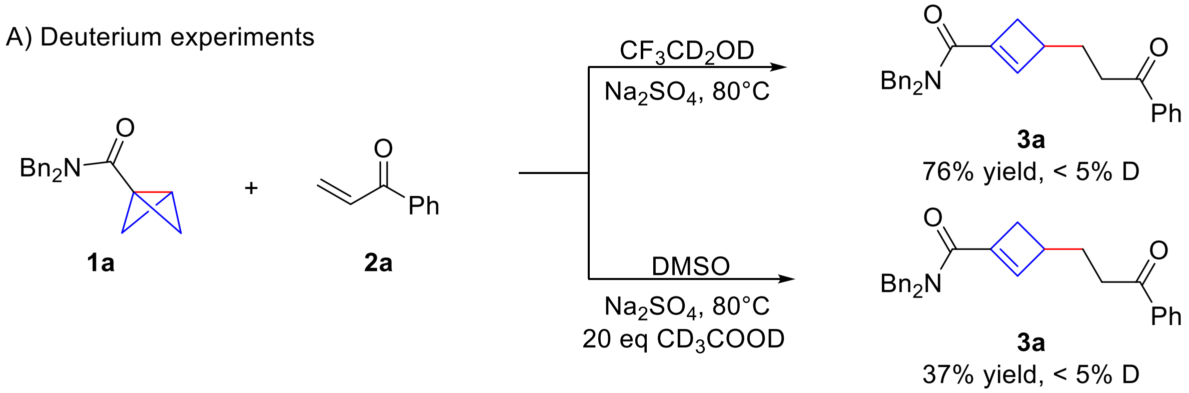

Na_2SO_4 (25.0 mg, 0.17 mmol), **1a** (55.4 mg, 0.2 mmol) and **2a** (13.2 mg, 0.1 mmol) in CF_3CD_2OD (0.1 mL) were charged into a 10 mL pressure tube under argon. The reaction tube was then sealed and placed into an oil bath at 80 °C. After stirred for 24 h, the reaction mixture was purified by silica gel chromatography (PE: EA = 5:1) to give the indicated product **3a** (31.1 mg, 76% yield). ^{1}H NMR (600 MHz, $CDCl_3$) δ 7.92 (d, J = 7.8 Hz, 2H), 7.56 – 7.54 (m, 1H), 7.45 – 7.43 (m, 2H), 7.37 – 7.35 (m, 2H), 7.33 – 7.29 (m, 4H), 7.25 – 7.24 (m, 2H), 7.17 – 7.16 (m, 2H), 6.46 (s, 1H), 4.62 – 4.54 (m, 4H), 3.01 – 2.95 (m, 3H), 2.80 – 2.77 (m, 1H), 2.43 – 2.41 (m, 1H), 1.98 – 1.87 (m, 2H).

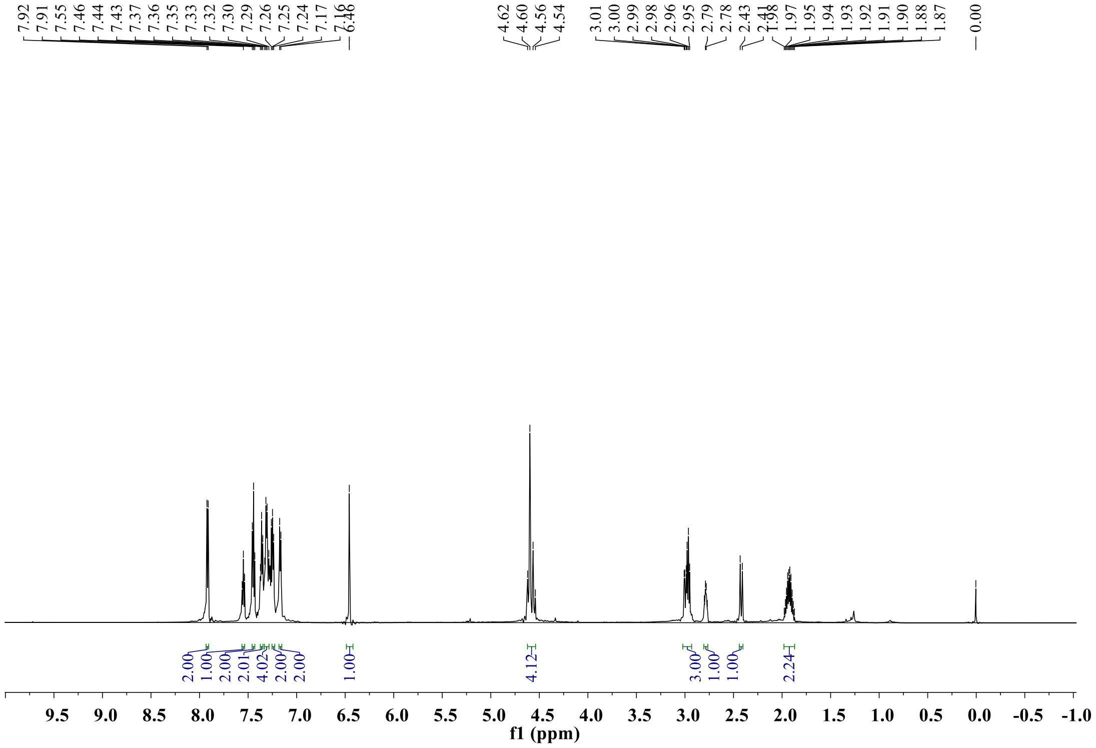

Supplementary Fig. 25 ^{1}H NMR (600 MHz, $CDCl_3$) of **3a**

Na$_2$SO$_4$ (25.0 mg, 0.17 mmol), **1a** (55.4 mg, 0.2 mmol), **2a** (13.2 mg, 0.1 mmol) and CD$_3$COOD (128.2 mg, 2.0 mmol) in DMSO (0.1 mL) were charged into a 10 mL pressure tube under argon. The reaction tube was then sealed and placed into an oil bath at 80 °C. After stirred for 24 h, the reaction mixture was purified by silica gel chromatography (PE: EA = 5:1) to give the indicated product **3a** (15.1 mg, 37% yield). ^{1}H NMR (400 MHz, CDCl$_3$) δ 7.93 – 7.91 (m, 2H), 7.57 – 7.53 (m, 1H), 7.46 – 7.42 (m, 2H), 7.38 – 7.29 (m, 6H), 7.25 – 7.23 (m, 2H), 7.18 – 7.16 (m, 2H), 6.45 (s, 1H), 4.63 – 4.53 (m, 4H), 3.02 – 3.95 (m, 3H), 2.81 – 2.76 (m, 1H), 2.42 (dd, J = 13.4, 1.4 Hz, 1H), 2.00 – 1.85 (m, 2H).

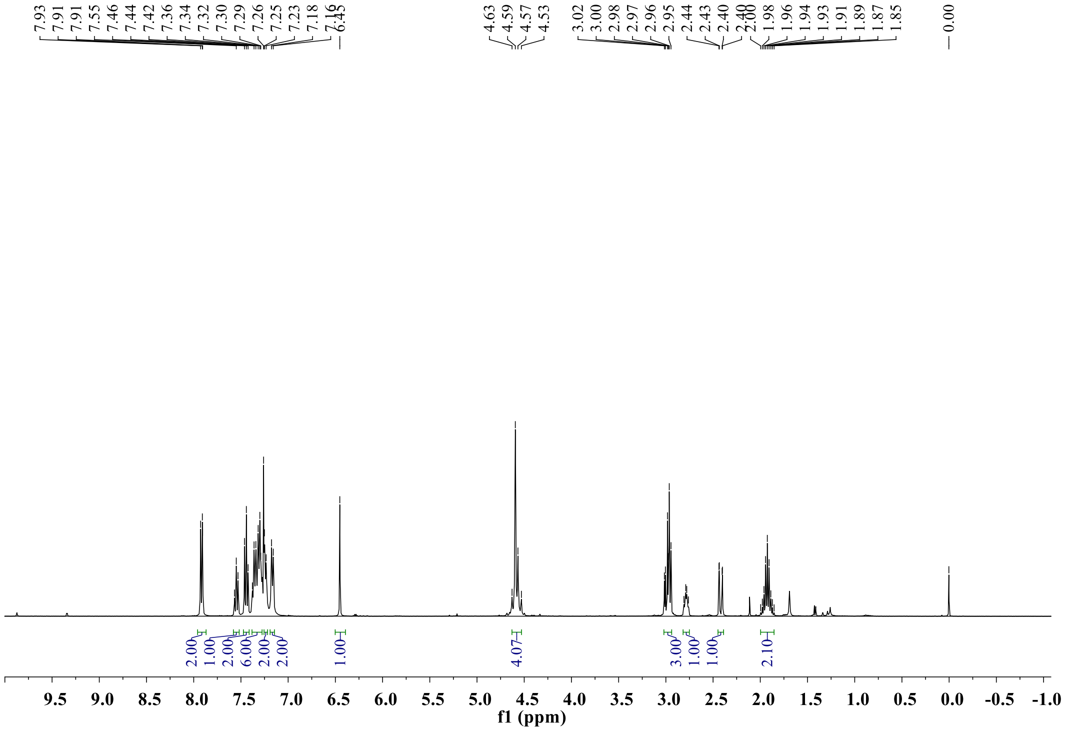

Supplementary Fig. 26 ^{1}H NMR (400 MHz, CDCl$_3$) of **3a**

2.4.3 Reaction with radical inhibitor

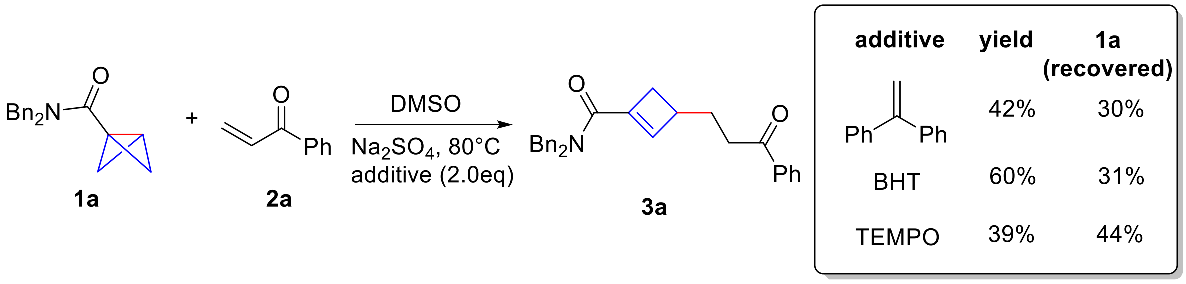

Na$_2$SO$_4$ (25.0 mg, 0.17 mmol), **1a** (55.4 mg, 0.2 mmol), ethene-1,1-diyldibenzene (36.1 mg, 0.2 mmol) and **2a** (13.2 mg, 0.1 mmol) in DMSO (0.1 mL) were charged into a 10 mL pressure tube

under argon. The reaction tube was then sealed and placed into an oil bath at 80 °C. After stirred for 24 h, the reaction mixture was purified by silica gel chromatography (PE: EA = 5:1) to give the indicated product **3a** (17.2 mg, 42% yield, **1a**: 16.0 mg).

Na_2SO_4 (25.0 mg, 0.17 mmol), **1a** (55.4 mg, 0.2 mmol), BHT (44.1 mg, 0.2 mmol) and **2a** (13.2 mg, 0.1 mmol) in DMSO (0.1 mL) were charged into a 10 mL pressure tube under argon. The reaction tube was then sealed and placed into an oil bath at 80 °C. After stirred for 24 h, the reaction mixture was purified by silica gel chromatography (PE: EA = 5:1) to give the indicated product **3a** (24.6 mg, 60% yield, **1a**: 16.8 mg).

Na_2SO_4 (25.0 mg, 0.17 mmol), **1a** (55.4 mg, 0.2 mmol), TEMPO (31.3 mg, 0.2 mmol) and **2a** (13.2 mg, 0.1 mmol) in DMSO (0.1 mL) were charged into a 10 mL pressure tube under argon. The reaction tube was then sealed and placed into an oil bath at 80 °C. After stirred for 24 h, the reaction mixture was purified by silica gel chromatography (PE: EA = 5:1) to give the indicated product **3a** (16.0 mg, 39% yield, **1a**: 24.5 mg).

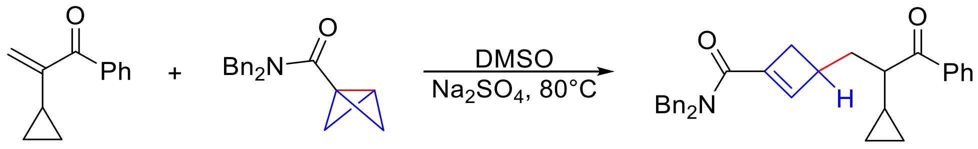

2y **1a** **14**

23% yield, dr 1:1

Na_2SO_4 (25.0 mg, 0.17 mmol), **1a** (55.4 mg, 0.2 mmol) and **2y** (17.2 mg, 0.1 mmol) in DMSO (0.1 mL) were charged into a 10 mL pressure tube under argon. The reaction tube was then sealed and placed into an oil bath at 80 °C. After stirred for 24 h, the reaction mixture was purified by silica gel chromatography (PE: EA = 5:1) to give the indicated product **14** (10.2 mg, 23% yield, dr = 1:1). ^{1}H NMR (600 MHz, $CDCl_3$) δ (mixture of isomers) 7.86 – 7.83 (m, 4H), 7.56 – 7.52 (m, 2H), 7.44 – 7.41 (m, 4H), 7.36 – 7.28 (m, 12H), 7.23 – 7.20 (m, 4H), 7.15 – 7.13 (m, 2H), 7.10 – 7.08 (m, 2H), 6.33 (s, 1H), 6.32 (s, 1H), 4.60 – 4.45 (m, 8H), 2.93 – 2.88 (m, 2H), 2.84 – 2.68 (m, 4H), 2.41 – 2.36 (m, 2H), 2.16 – 2.09 (m, 2H), 1.88 – 1.77 (m, 2H), 1.04 – 0.96 (m, 2H), 0.58 – 0.54 (m, 2H), 0.45 – 0.40 (m, 2H), 0.20 – 0.15 (m, 2H), 0.13 – 0.08 (m, 2H). ^{13}C NMR (151 MHz, $CDCl_3$) δ (mixture of isomers) 203.7, 203.7, 164.6, 164.5, 144.9, 144. 9, 139.4, 139.3, 137.6, 137.5, 137.1, 137.1, 136.8, 136.8, 133.2, 133.2, 129.0, 128.8, 128.7, 128.7, 128.4, 128.3, 127.7, 127.6, 126.7, 126.7, 50.2, 50.1,

49.7, 49.2, 48.2, 48.2, 38.2, 38.1, 37.8, 37.5, 36.8, 36.4, 14.0, 13.9, 4.7, 4.6, 4.1, 4.0. HRMS (ESI, m/z): calcd for $C_{31}H_{31}NNaO_2^+$ [M + Na] $^+$: 472.2247, found 472.2239.

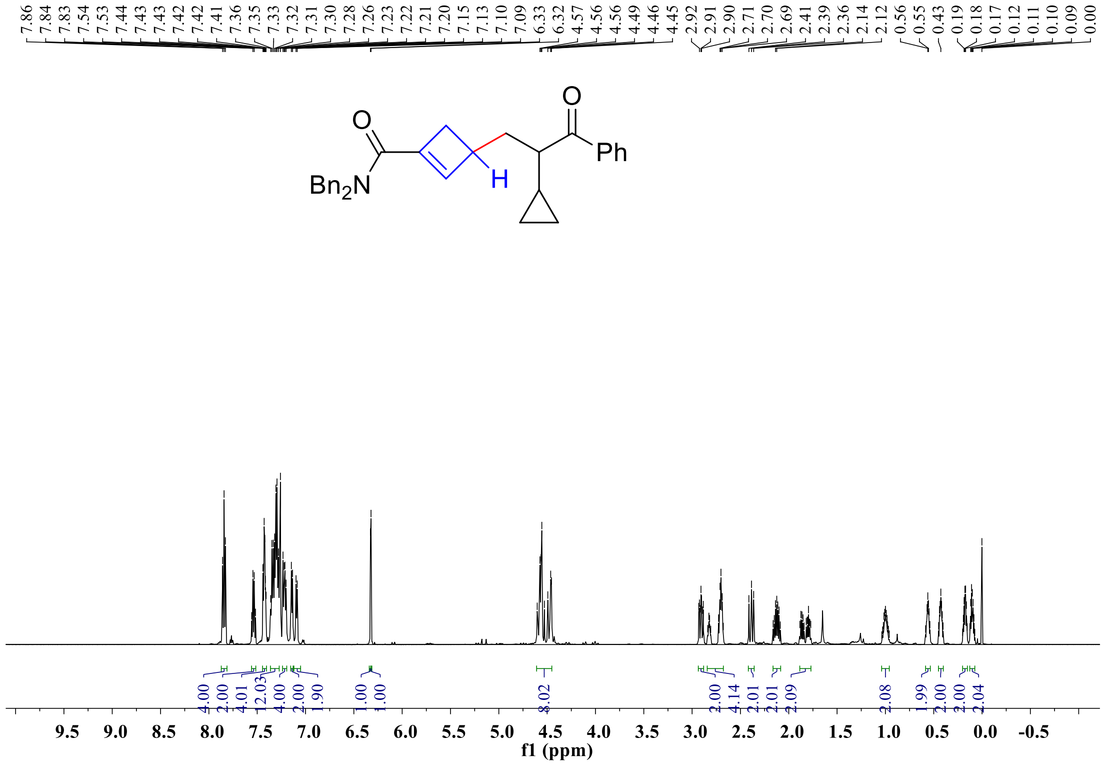

Supplementary Fig. 27 ^{1}H NMR (600 MHz, $CDCl_3$) of **14**

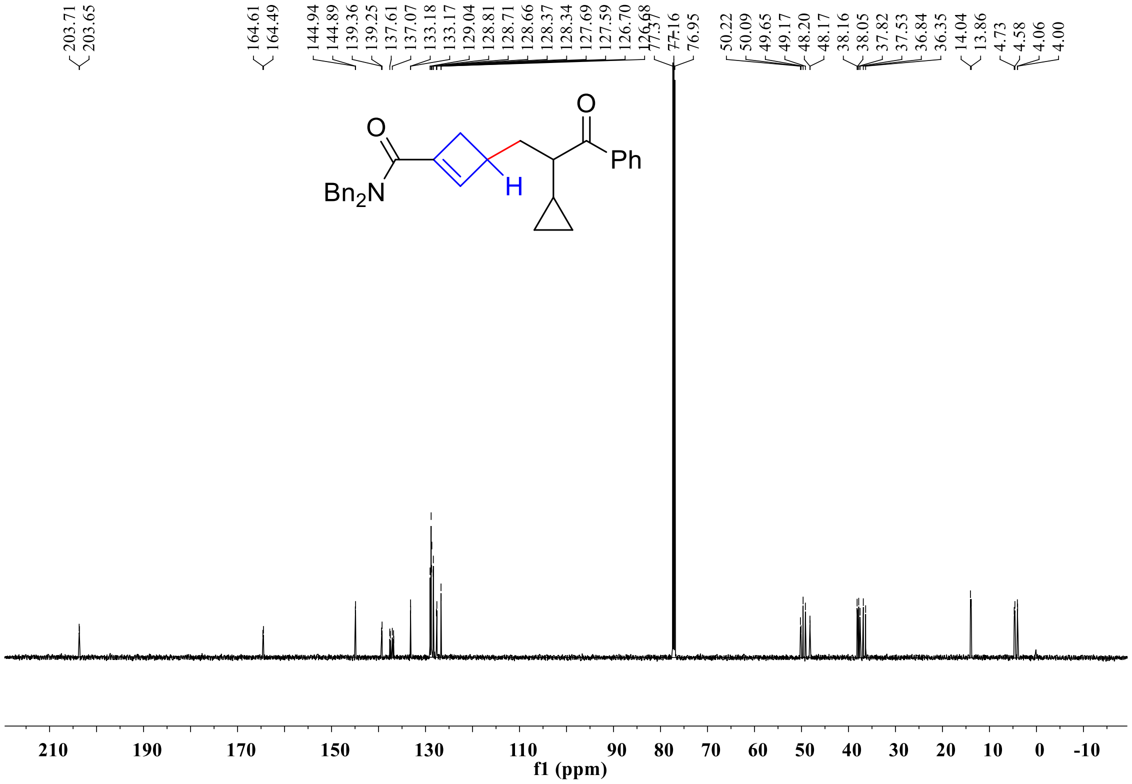

Supplementary Fig. 28 ^{13}C NMR (151 MHz, $CDCl_3$) of **14**

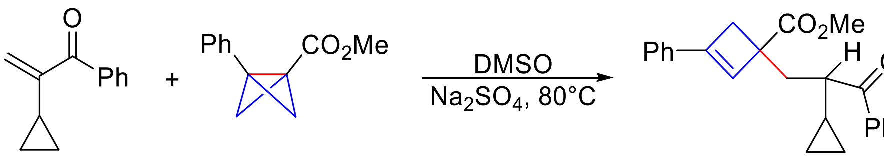

2y **1a'** **15**

52% yield, dr 1:1

Na_2SO_4 (25.0 mg, 0.17 mmol), **1a'** (37.6 mg, 0.2 mmol) and **2y** (17.2 mg, 0.1 mmol) in DMSO (0.1 mL) were charged into a 10 mL pressure tube under argon. The reaction tube was then sealed and placed into an oil bath at 80 °C. After stirred for 24 h, the reaction mixture was purified by silica gel chromatography (PE: EA = 10:1) to give the indicated product **15** (19.9 mg, 52% yield, dr = 1:1). ^1H NMR (600 MHz, $CDCl_3$) δ (mixture of isomers) 7.91 (d, *J* = 7.8 Hz, 2H), 7.82 (d, *J* = 7.8 Hz, 2H), 7.56 – 7.54 (m, 1H), 7.49 – 7.46 (m, 1H), 7.46 – 7.43 (m, 2H), 7.35 – 7.33 (m, 2H), 7.29 – 7.28 (m, 3H), 7.24 – 7.23 (m, 3H), 7.16 – 7.14 (m, 2H), 7.14 – 7.11 (m, 2H), 6.20 (s, 1H), 6.19 (s, 1H), 3.62 (s, 3H), 3.61 (s, 3H), 3.21 (d, *J* = 13.2 Hz, 1H), 3.11 (d, *J* = 13.2 Hz, 1H), 2.89 – 2.83 (m, 2H), 2.82 – 2.77 (m, 2H), 2.73 (d, *J* = 13.2 Hz, 1H), 2.65 (d, *J* = 13.2 Hz, 1H), 2.30 (dd, *J* = 13.8, 4.8 Hz, 1H), 2.24 (dd, *J* = 13.0, 3.0 Hz, 1H), 1.01 – 0.92 (m, 2H), 0.61 – 0.56 (m, 2H), 0.45 – 0.40 (m, 2H), 0.24 – 0.17 (m, 2H), 0.14 – 0.10 (m, 2H). ^1H NMR (600 MHz, $CDCl_3$) δ (one isomer) 7.82 (d, *J* = 7.6 Hz, 2H), 7.49 – 7.46 (m, 1H), 7.35 – 7.33 (m, 2H), 7.25 – 7.22 (m, 3H), 7.15 – 7.14 (m, 2H), 6.19 (s, 1H), 3.62 (s, 3H), 3.11 (d, *J* = 13.2 Hz, 1H), 2.85 – 2.77 (m, 2H), 2.65 (d, *J* = 13.2 Hz, 1H), 2.24 (dd, *J* = 13.2, 3.0 Hz, 1H), 0.98 – 0.92 (m, 1H), 0.61 – 0.56 (m, 1H), 0.43 – 0.39 (m, 1H), 0.21 – 0.17 (m, 1H), 0.14 – 0.10 (m, 1H). ^{13}C NMR (151 MHz, $CDCl_3$) δ (one isomer) 203.4, 175.4, 146.2, 137.7, 133.6, 132.8, 129.7, 128.6, 128.5, 128.5, 128.4, 124.9, 52.0, 50.3, 47.3, 38.5, 38.1, 15.0, 5.0, 4.7. HRMS (ESI, m/z): calcd for $C_{24}H_{24}NaO_3^+$ [M + Na] $^+$: 383.1618, found 383.1616.

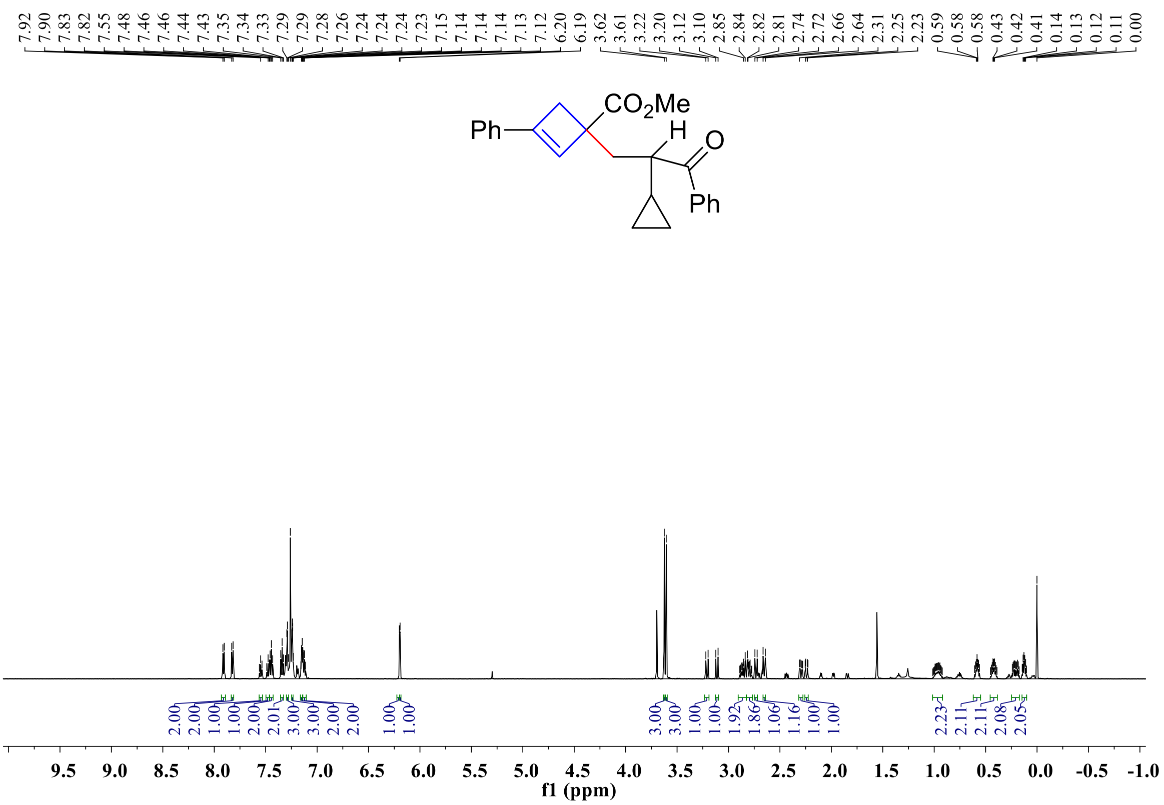

Supplementary Fig. 29 ^{1}H NMR (600 MHz, $CDCl_3$) of **15** (mixture of isomers)

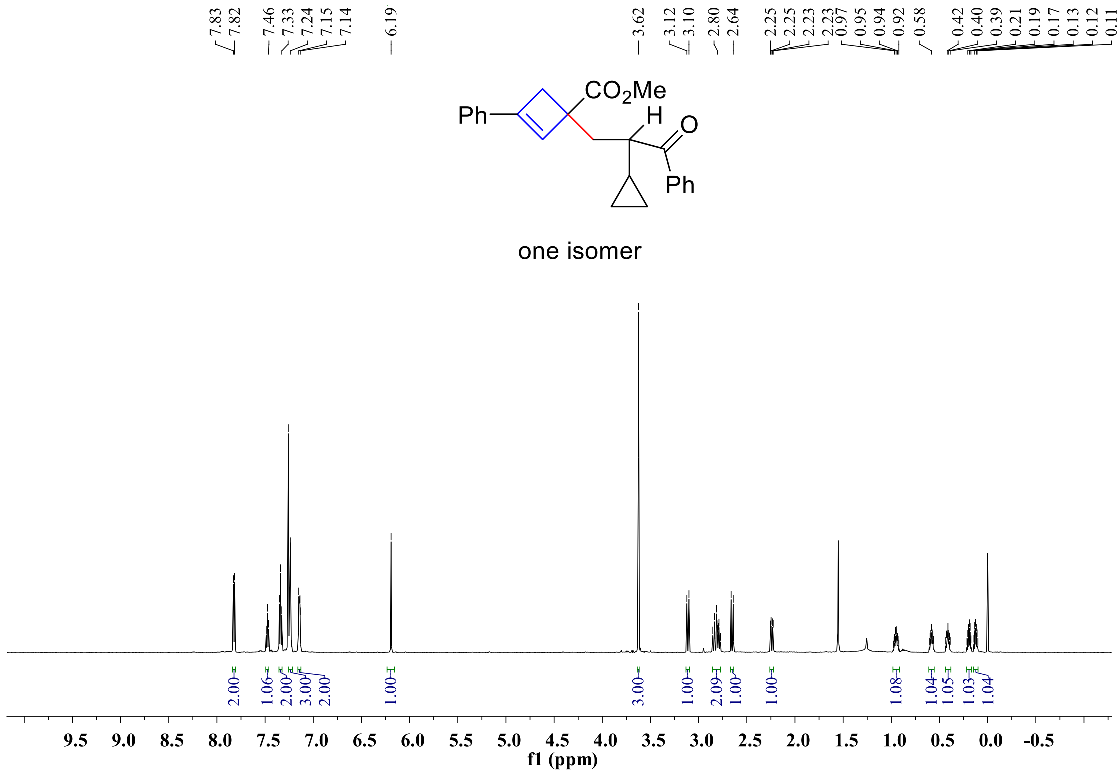

Supplementary Fig. 30 ^{1}H NMR (600 MHz, $CDCl_3$) of **15** (one isomer)

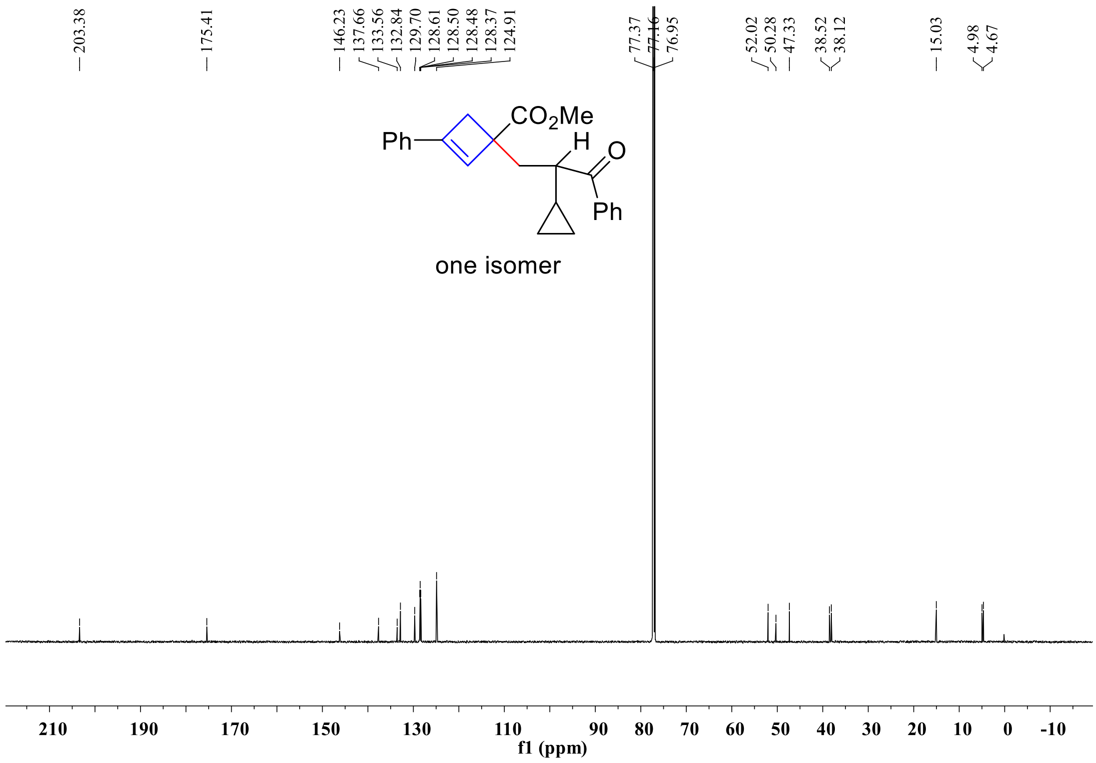

Supplementary Fig. 31 ^{13}C NMR (151 MHz, $CDCl_3$) of **15** (one isomer)

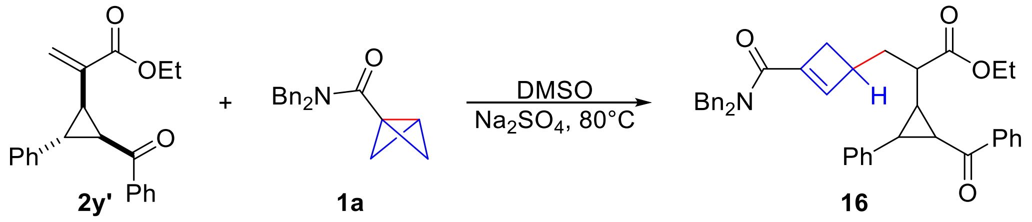

Na_2SO_4 (25.0 mg, 0.17 mmol), **1a** (55.4 mg, 0.2 mmol) and **2y'** (32.0 mg, 0.1 mmol) in DMSO (0.1 mL) were charged into a 10 mL pressure tube under argon. The reaction tube was then sealed and placed into an oil bath at 80 °C. After stirred for 24 h, the reaction mixture was purified by silica gel chromatography (PE: EA = 5:1) to give the indicated product **16** (10.1 mg, 17% yield, dr = 2:1). ^{1}H NMR (400 MHz, CD_2Cl_2) δ major: 7.96 – 7.95 (m, 2H), 7.56 – 7.53 (m, 1H), 7.45 – 7.41 (m, 2H), 7.35 – 7.26 (m, 8H), 7.22 – 7.11 (m, 7H), 6.24 (s, 1H), 4.57 – 4.39 (m, 4H), 4.11 – 4.04 (m, 2H), 3.12 – 3.08 (m, 1H), 2.95 – 2.93 (m, 1H), 2.81 – 2.71 (m, 2H), 2.64 – 2.58 (m, 1H), 2.17 – 2.08 (m, 2H), 1.93 – 1.85 (m, 1H), 1.49 – 1.30 (m, 1H), 1.18 (t, J = 7.2 Hz, 3H). ^{1}H NMR (400 MHz, CD_2Cl_2) δ minor: 6.16 (s, 1H). ^{13}C NMR (101 MHz, CD_2Cl_2) δ mixture of isomers 197.2, 175.0, 164.6, 144.2, 144.0, 140.3, 139.8, 138.8, 137.7, 137.4, 133.4, 129.2, 129.0, 128.9, 128.8, 128.6, 128.4, 128.3, 127.8, 127.7, 127.2, 127.1, 127.0, 61.0, 50.6, 48.3, 41.4, 41.3, 37.9, 37.8, 36.7, 36.7, 36.4, 36.4, 33.3,

33.1, 32.4, 14.5. HRMS (ESI, m/z): calcd for $C_{40}H_{39}NNaO_4^+$ [M + Na] $^+$: 620.2771, found 620.2770.

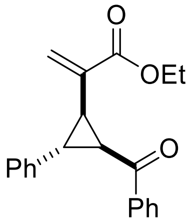

2y', synthesized and conformed according to the literature report[3]. ^{1}H NMR (400 MHz, $CDCl_3$) δ 8.02 – 7.99 (m, 2H), 7.55 – 7.51 (m, 1H), 7.46 – 7.42 (m, 2H), 7.34 – 7.30 (m, 2H), 7.27 – 7.21 (m, 3H), 6.43 (s, 1H), 5.83 (t, J = 1.2 Hz, 1H), 4.04 (q, J = 7.0 Hz, 2H), 3.34 – 3.28 (m, 2H), 2.98 – 2.94 (m, 1H), 1.12 (t, J = 7.2 Hz, 3H). ^{13}C NMR (101 MHz, $CDCl_3$) δ 195.3, 166.4, 139.5, 138.3, 134.5, 132.9, 128.7, 128.5, 128.4, 128.2, 126.9, 60.9, 35.9, 34.1, 29.8, 14.1.

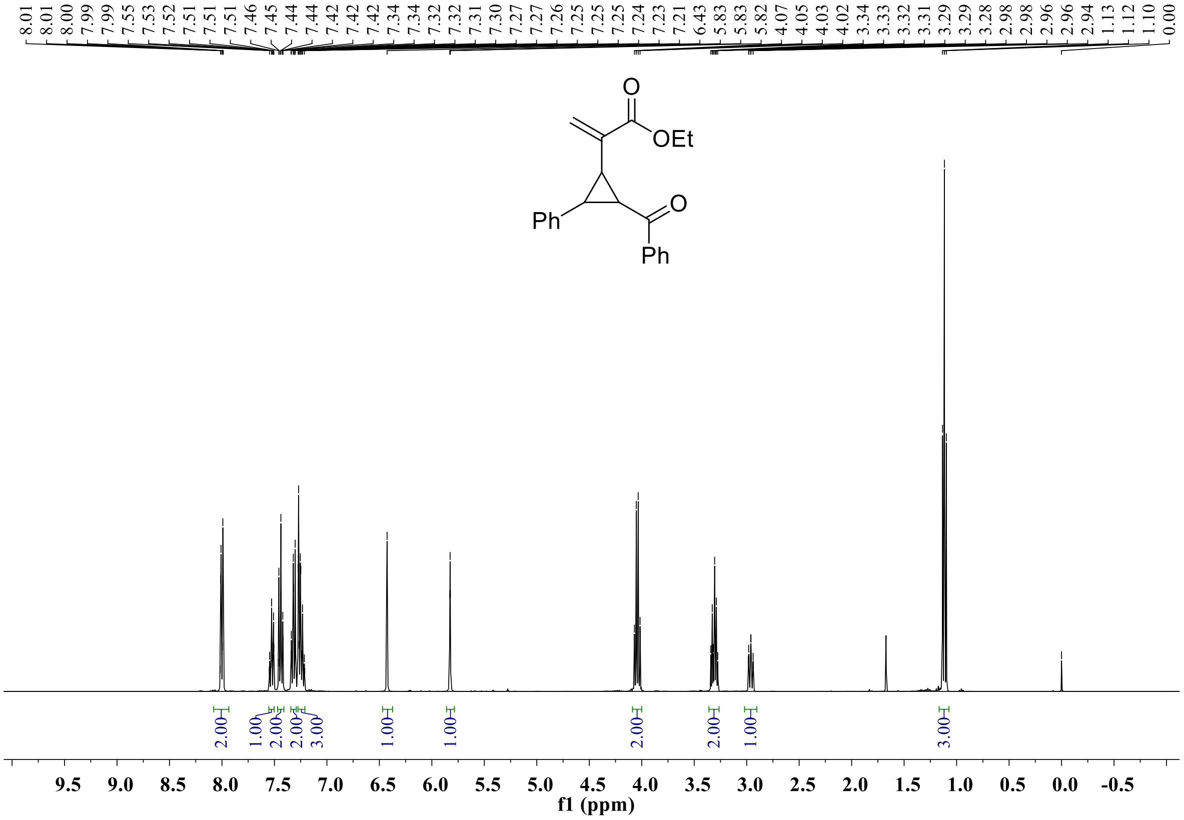

Supplementary Fig. 32 ^{1}H NMR (400 MHz, $CDCl_3$) of **2y'**

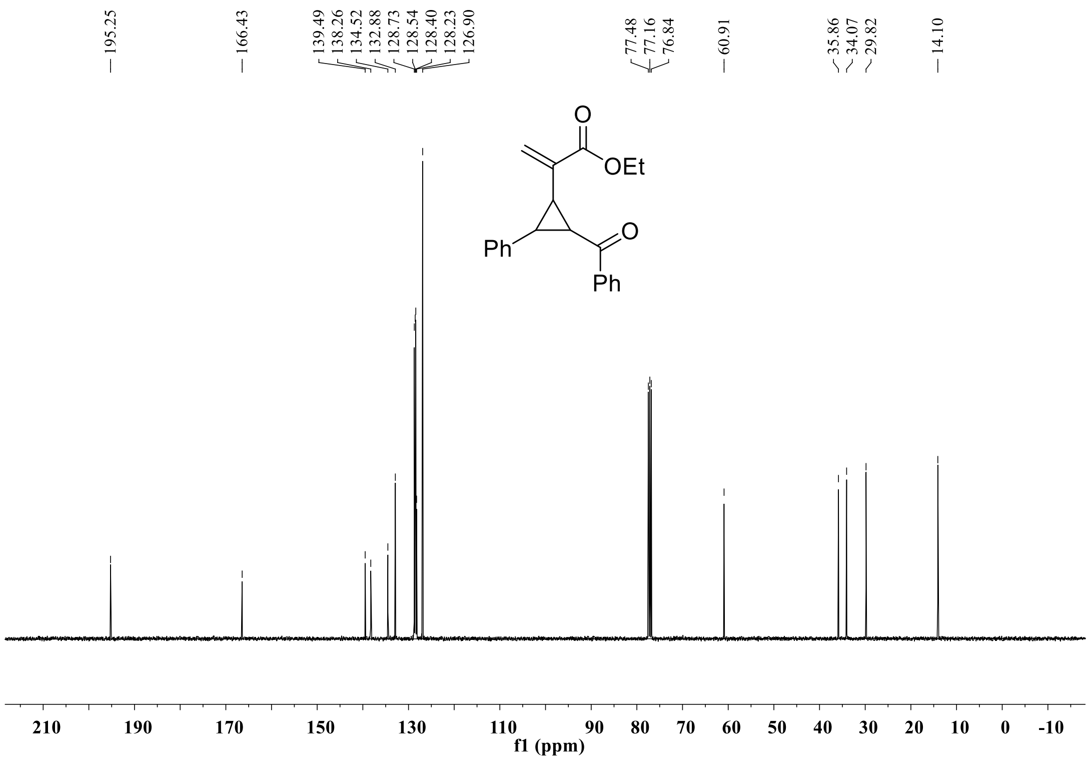

Supplementary Fig. 33 ^{13}C NMR (101 MHz, $CDCl_3$) of **2y'**

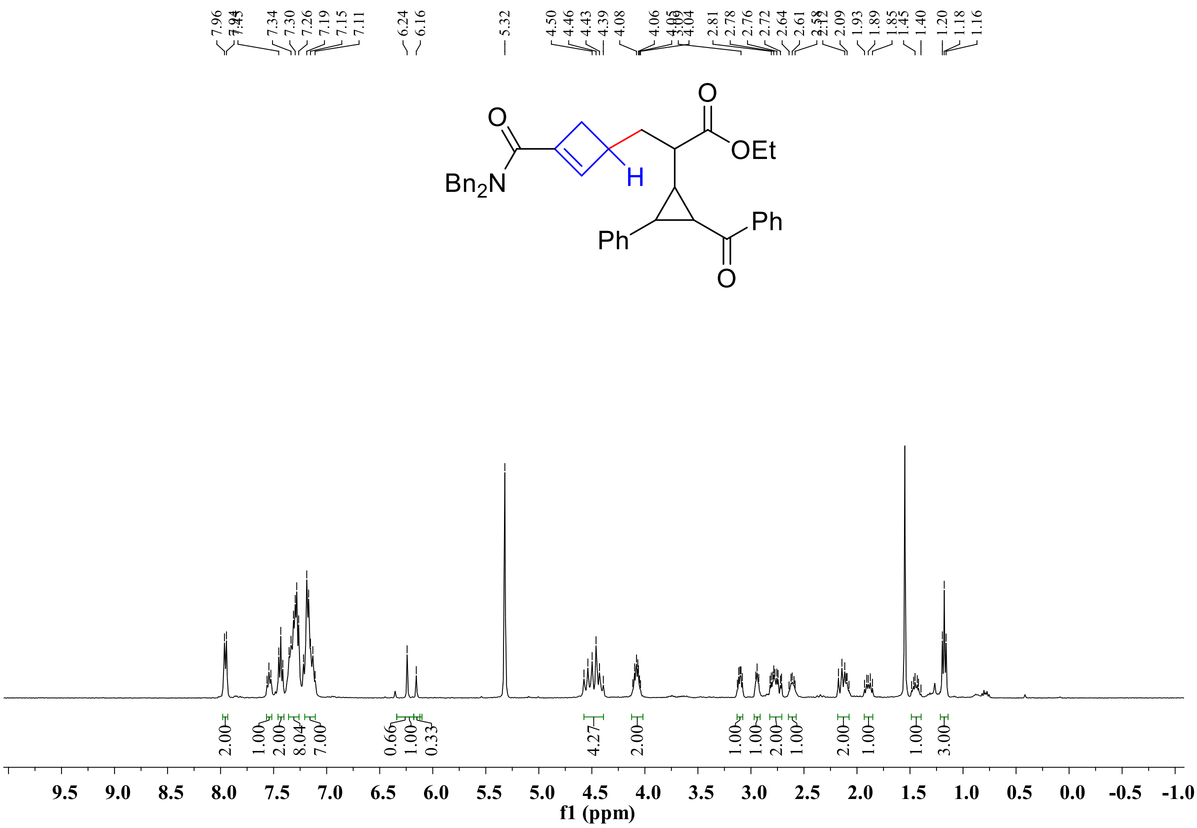

Supplementary Fig. 34 ^{1}H NMR (400 MHz, CD_2Cl_2) of **16** (mixture of isomers)

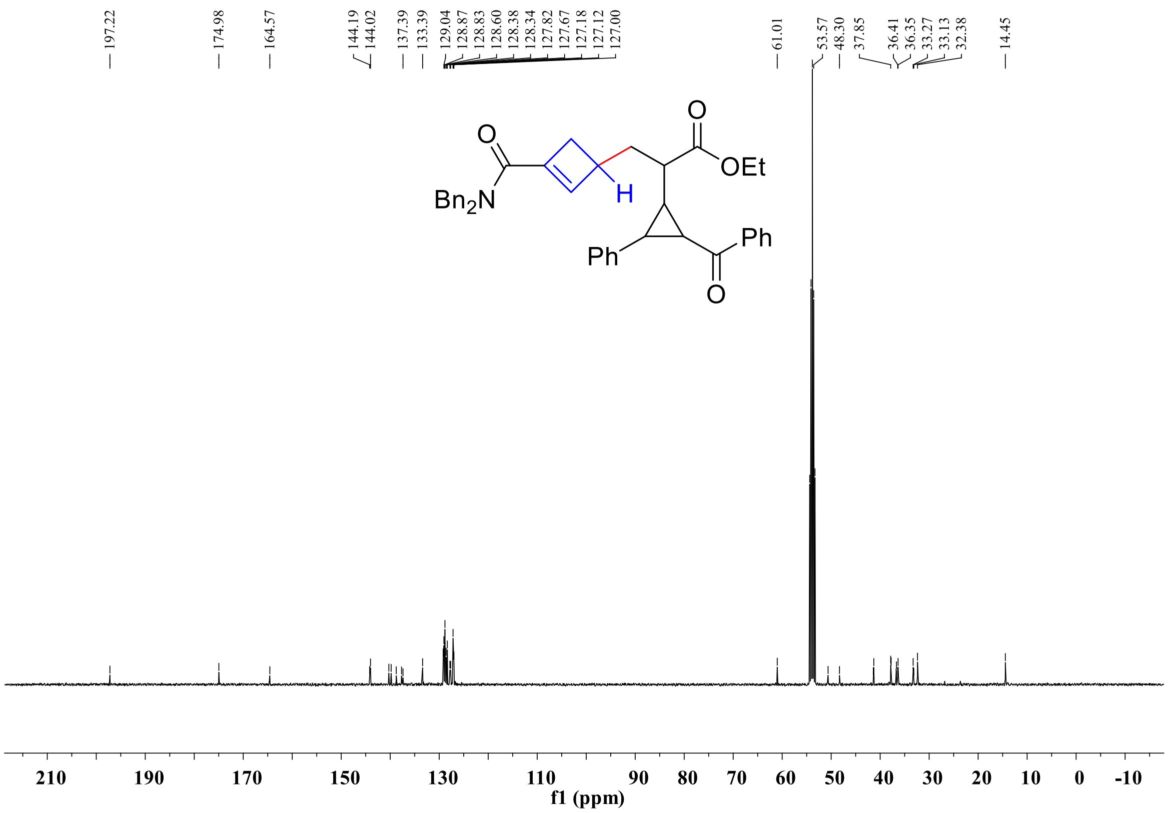

Supplementary Fig. 35 ^{13}C NMR (101 MHz, CD_2Cl_2) of **16** (mixture of isomers)

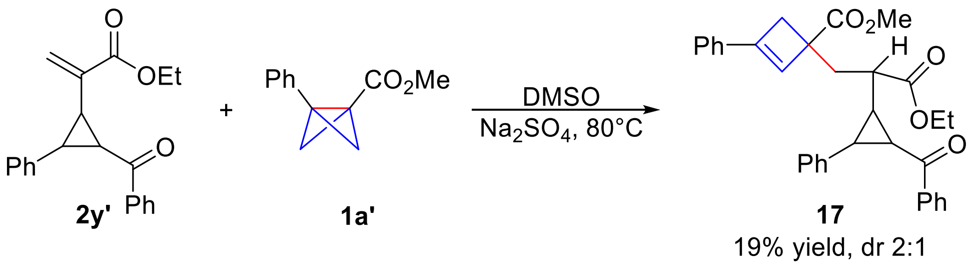

Na_2SO_4 (25.0 mg, 0.17 mmol), **1a'** (37.6 mg, 0.2 mmol) and **2y'** (32.0 mg, 0.1 mmol) in DMSO (0.1 mL) were charged into a 10 mL pressure tube under argon. The reaction tube was then sealed and placed into an oil bath at 80 °C. After stirred for 24 h, the reaction mixture was purified by silica gel chromatography (PE: EA = 10:1) to give the indicated product **17** (9.5 mg, 19% yield, dr = 2:1). ^{1}H NMR (400 MHz, CD_2Cl_2) δ major: 8.03 – 7.96 (m, 2H), 7.54 – 7.50 (m, 1H), 7.43 – 7.39 (m, 2H), 7.35 – 7.30 (m, 3H), 7.28 – 7.26 (m, 4H), 7.22 – 7.20 (m, 1H), 7.17 – 7.15 (m, 2H), 6.14 (s, 1H), 4.19 – 4.03 (m, 2H), 3.55 (s, 3H), 3.16 – 3.12 (m, 1H), 3.05 (t, J = 6.0 Hz, 1H), 2.99 (d, J = 13.0 Hz, 1H), 2.81 – 2.75 (m, 1H), 2.56 – 2.47 (m, 2H), 2.15 – 2.08 (m, 1H), 1.71 (dd, J = 14.0, 2.2 Hz, 1H), 1.19 (t, J = 7.2 Hz, 3H). ^{13}C NMR (101 MHz, CD_2Cl_2) δ 196.9, 175.1, 174.8, 146.9, 140.2, 138.9,

134.0, 133.3, 128.9, 128.9, 128.7, 128.7, 128.4, 127.1, 127.0 125.1, 61.1, 52.1, 50.0, 39.9, 38.4, 38.2, 37.2, 32.8, 32.5, 14.4. ^{1}H NMR (400 MHz, CD_2Cl_2) δ minor: 8.03 – 7.98 (m, 2H), 7.55 – 7.51 (m, 1H), 7.45 – 7.39 (m, 2H), 7.33 – 7.22 (m, 8H), 7.18 – 7.16 (m, 2H), 6.18 (s, 1H), 4.20 – 4.07 (m, 2H), 3.52 (s, 3H), 3.15 – 3.12 (m, 1H), 3.05 – 3.02 (m, 1H), 2.98 (d, J = 13.0 Hz, 1H), 2.86 – 2.78 (m, 1H), 2.50 – 2.45 (m, 1H), 2.41 – 2.34 (m, 1H), 2.16 – 2.09 (m, 1H), 1.90 – 1.86 (m, 1H), 1.23 (t, J = 7.2 Hz, 3H). ^{13}C NMR (101 MHz, CD_2Cl_2) 196.9, 174.9, 174.7, 146.3, 140.2, 138.9, 134.1, 133.3, 129.1, 129.0, 128.9, 128.7, 128.5, 127.1, 127.0, 125.1, 61.1, 52.0, 50.0, 40.6, 39.5, 39.4, 37.3, 32.7, 32.6, 14.4. HRMS (ESI, m/z): calcd for $C_{33}H_{32}NaO_5^+$ [M + Na] $^+$: 531.2142, found 531.2143.

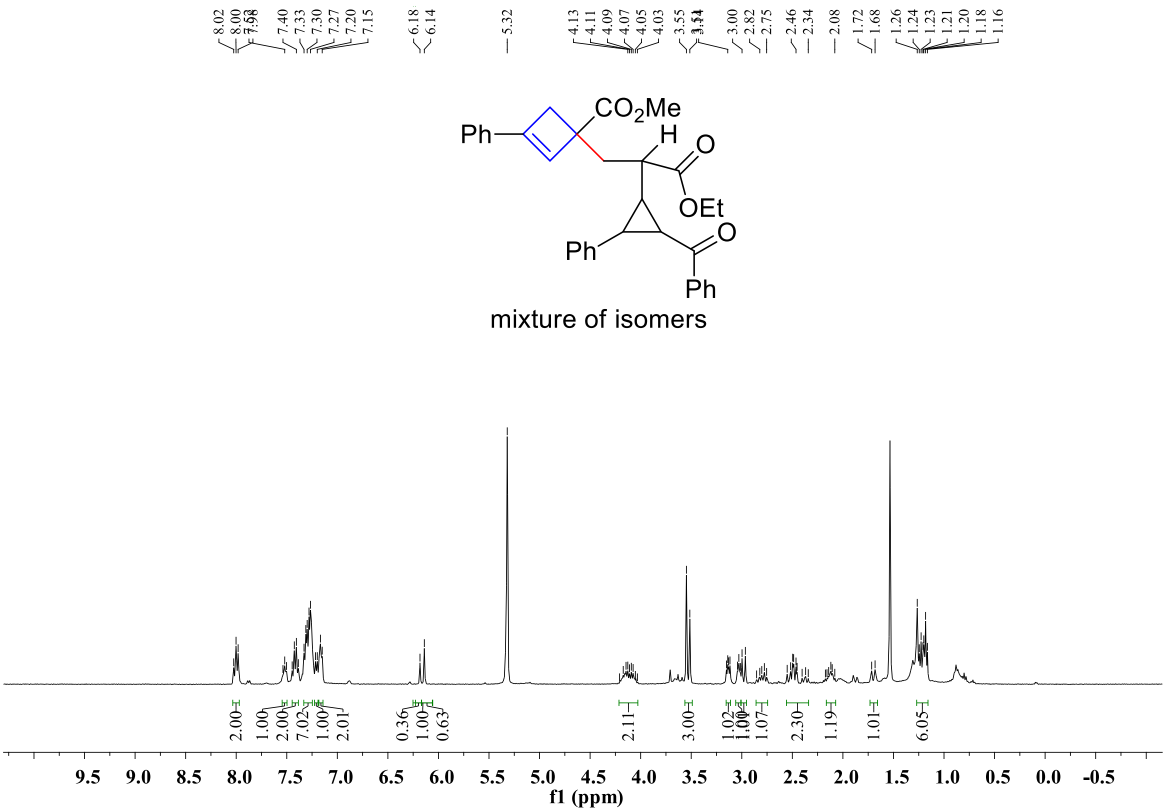

Supplementary Fig. 36 ^{1}H NMR (400 MHz, CD_2Cl_2) of **17** (mixture of isomers)

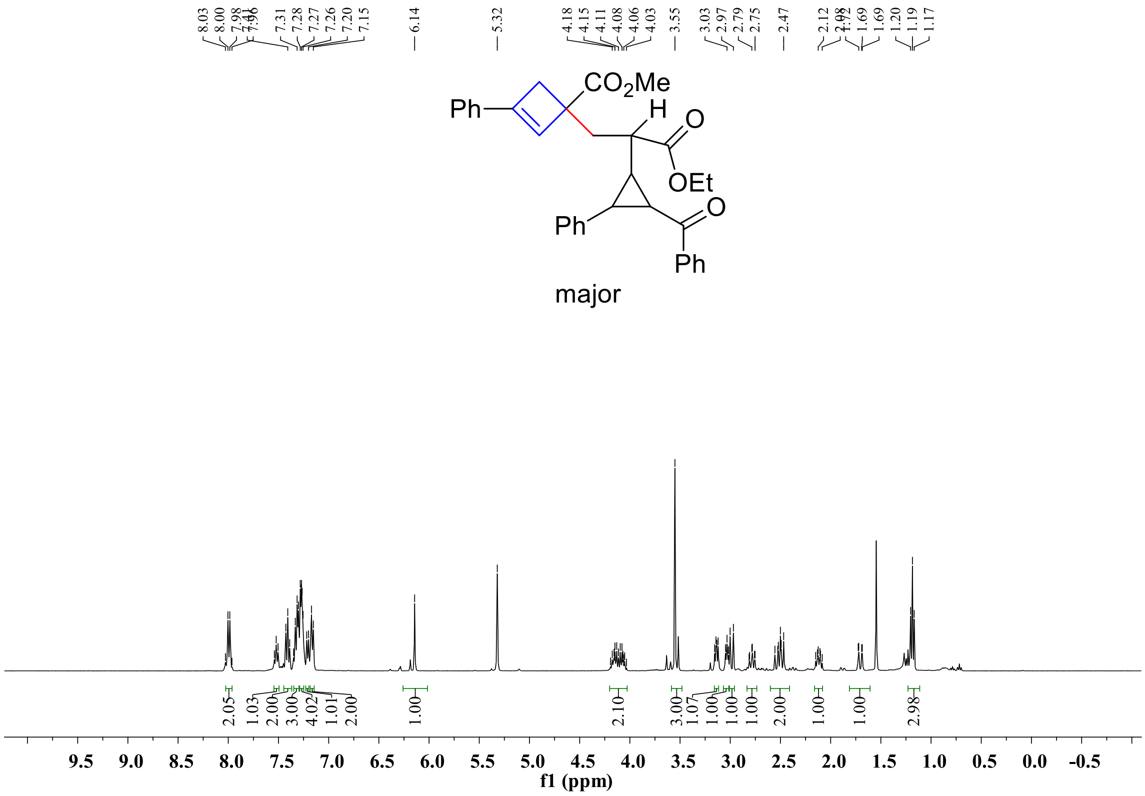

Supplementary Fig. 37 ^{1}H NMR (400 MHz, CD_2Cl_2) of **17** (major)

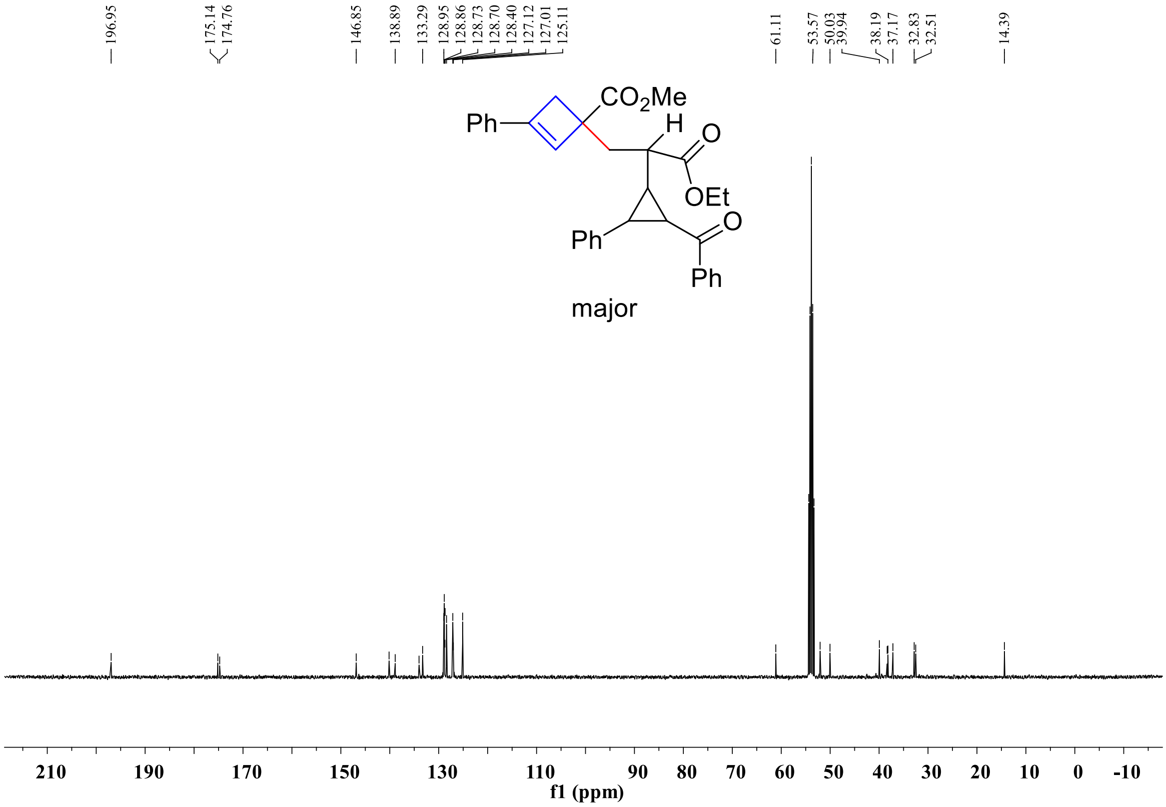

Supplementary Fig. 38 ^{13}C NMR (101 MHz, CD_2Cl_2) of **17** (major)

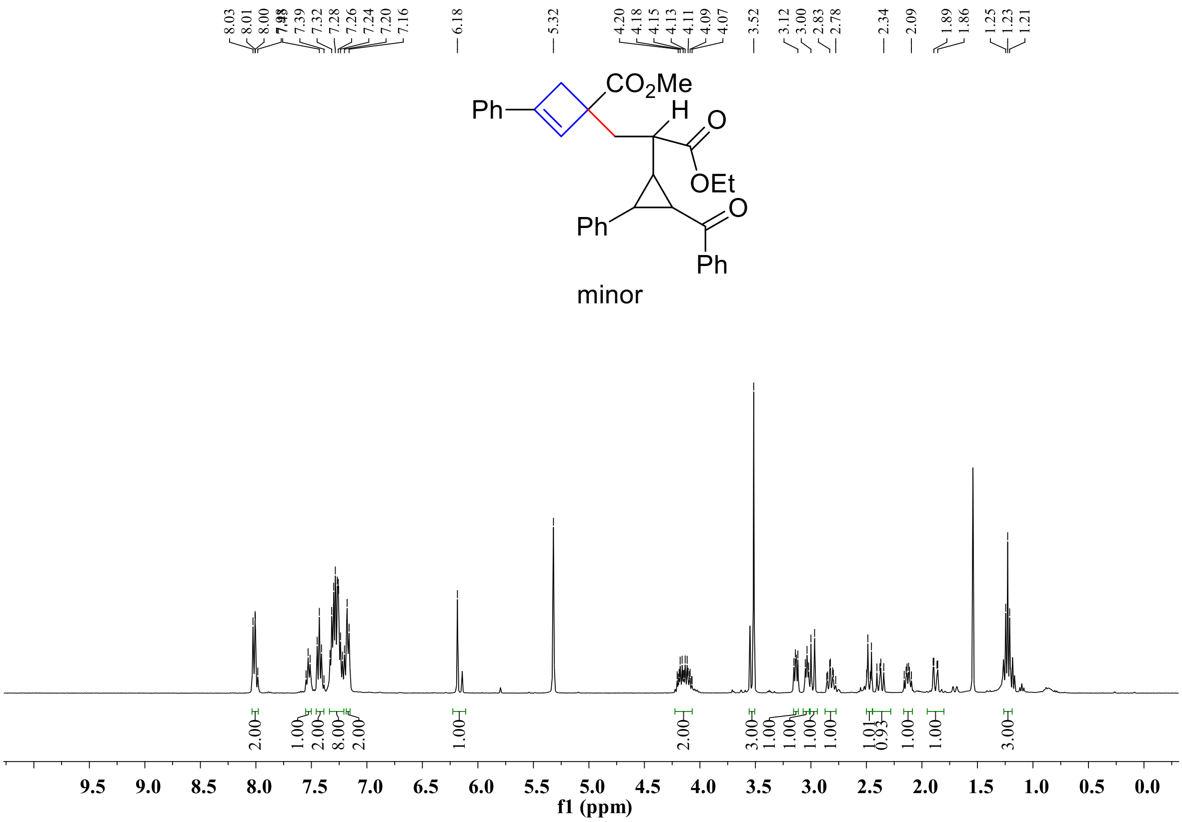

Supplementary Fig. 39 ^{1}H NMR (400 MHz, CD_2Cl_2) of **17** (minor)

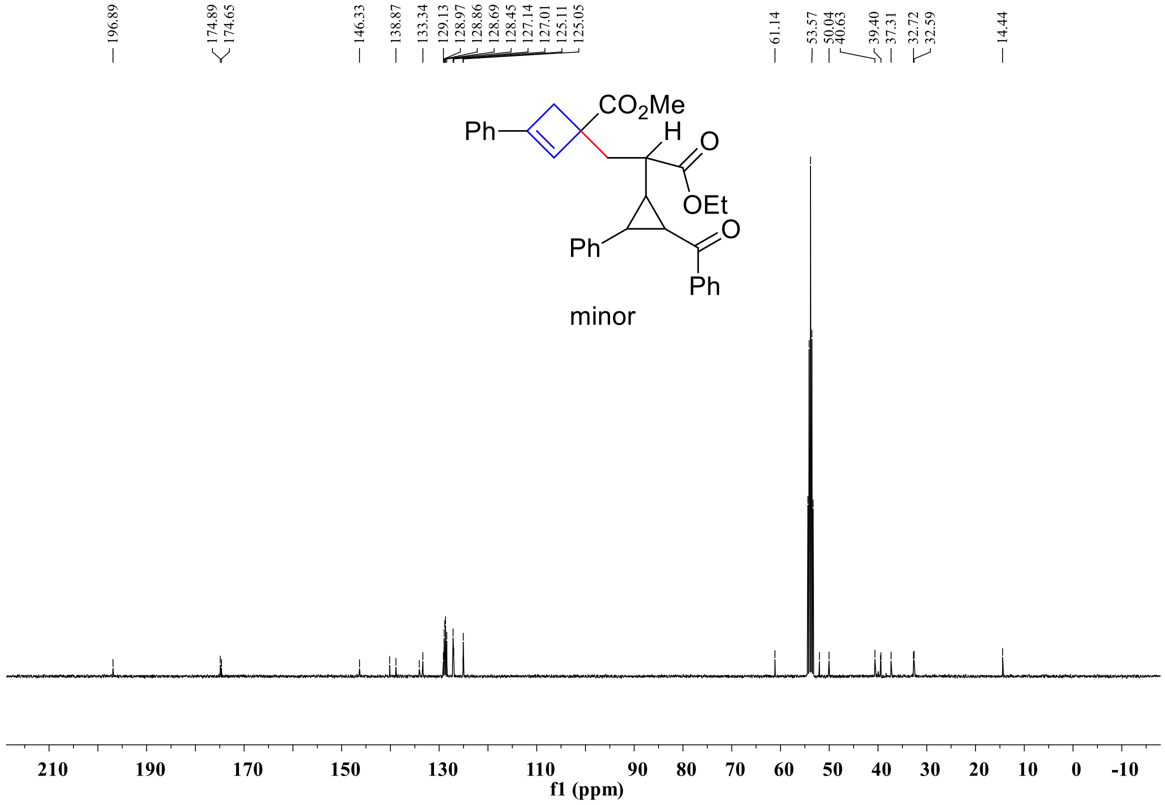

Supplementary Fig. 40 ^{13}C NMR (101 MHz, CD_2Cl_2) of **17** (minor)

2.4.4 EPR experiments

EPR measurements were carried out on a Bruker EPR EMX plus (X-band) with a dual cavity utilizing modulation and microwave frequencies of 100 kHz and 9.77 GHz. EPR signals were recorded at X-band with the following conditions: modulation amplitude 0.1 mT; time constant 20.48. Center field 3480 G, Microwave power 2.00 Mw, Modulation amplitude 4.0 G, Scan range 1000 G, Time constant 40.96 ms, Conversion time 163.84 ms, Sweep time 150 s, Receiver gain 30 db, Resolution 1024 point.

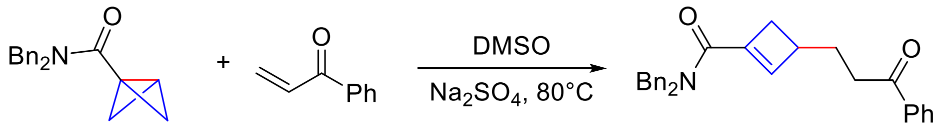

Na_2SO_4 (25.0 mg, 0.17 mmol), **1a** (55.4 mg, 0.2 mmol) and **2a** (13.2 mg, 0.1 mmol) in DMSO (0.1 mL) were charged into a 10 mL pressure tube under argon. The mixture was heated at 80 °C for 2h. A portion of the crude reaction solution was introduced into an EPR tube with 2 mm inner diameter under argon. After cooling with liquid nitrogen, this solution was subjected to EPR measurements at 77 K under argon.

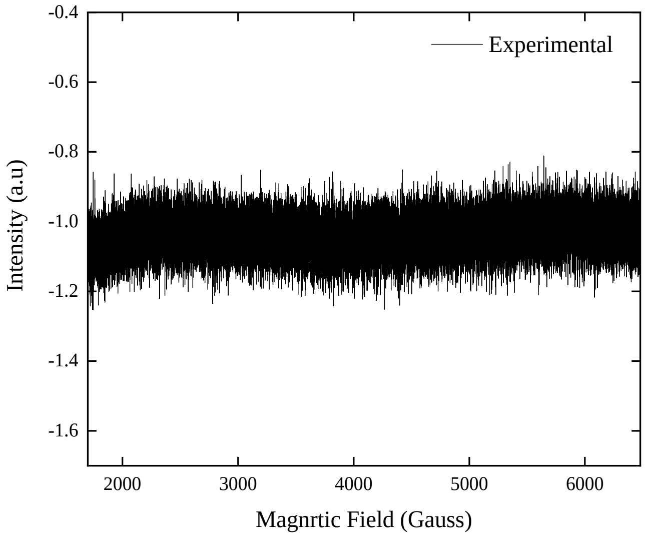

Supplementary Fig. 41 The EPR spectrum of the reaction mixture for the synthesis of **3a**

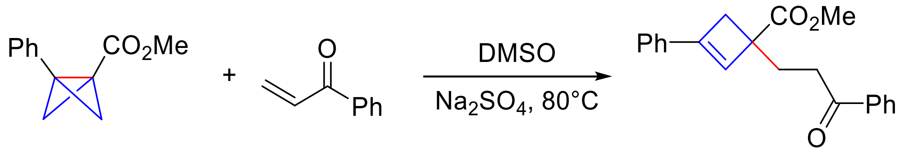

Na_2SO_4 (25.0 mg, 0.17 mmol), **1a'** (37.6 mg, 0.2 mmol) and **2a** (13.2 mg, 0.1 mmol) in DMSO (0.1

mL) were charged into a 10 mL pressure tube under argon. The mixture was heated at 80 °C for 2h. A portion of the crude reaction solution was introduced into an EPR tube with 2 mm inner diameter under argon. After cooling with liquid nitrogen, this solution was subjected to EPR measurements at 77 K under argon.

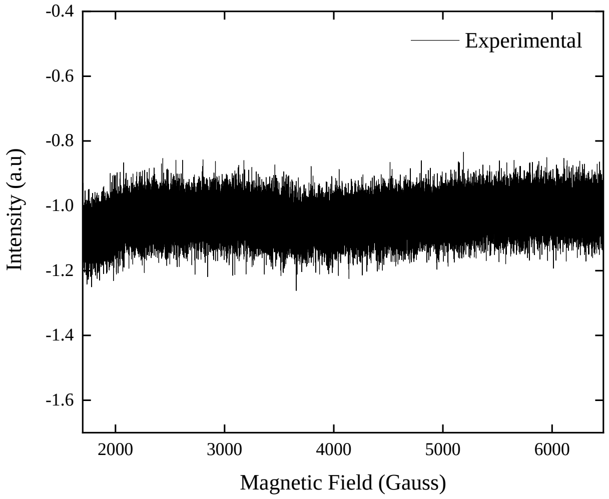

Supplementary Fig. 42 The EPR spectrum of the reaction mixture for the synthesis of **6a**

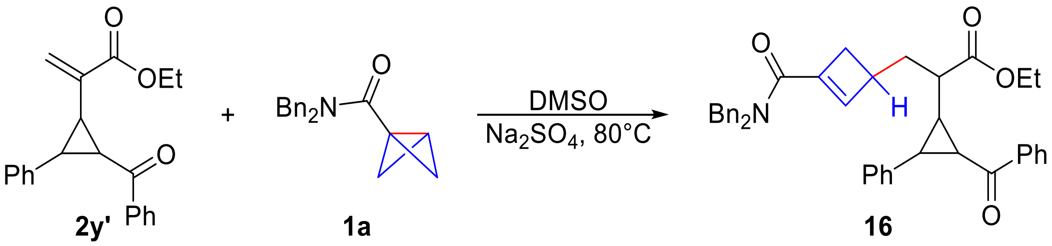

Na_2SO_4 (25.0 mg, 0.17 mmol), **1a** (55.4 mg, 0.2 mmol) and **2y'** (32.0 mg, 0.1 mmol) in DMSO (0.1 mL) were charged into a 10 mL pressure tube under argon. The mixture was heated at 80 °C for 2h. A portion of the crude reaction solution was introduced into an EPR tube with 2 mm inner diameter under argon. After cooling with liquid nitrogen, this solution was subjected to EPR measurements at 77 K under argon.

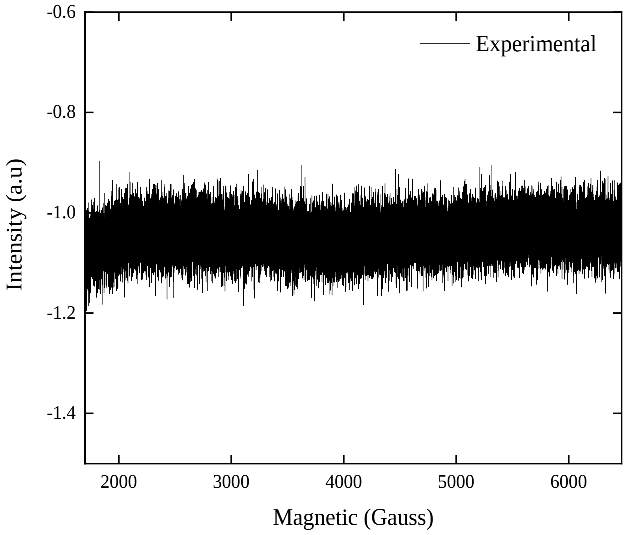

Supplementary Fig. 43 The EPR spectrum of the reaction mixture for the synthesis of **16**

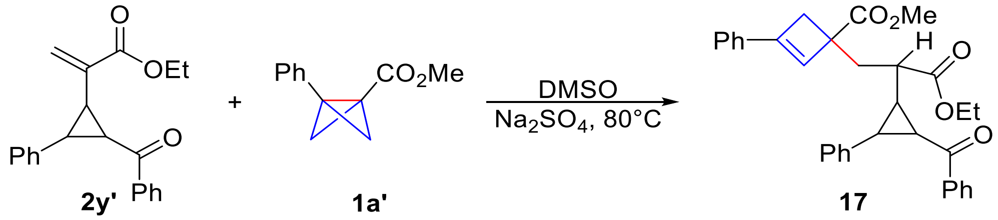

Na_2SO_4 (25.0 mg, 0.17 mmol), **1a'** (37.6 mg, 0.2 mmol) and **2y'** (32.0 mg, 0.1 mmol) in DMSO (0.1 mL) were charged into a 10 mL pressure tube under argon. The mixture was heated at 80 °C for 2h. A portion of the crude reaction solution was introduced into an EPR tube with 2 mm inner diameter under argon. After cooling with liquid nitrogen, this solution was subjected to EPR measurements at 77 K under argon.

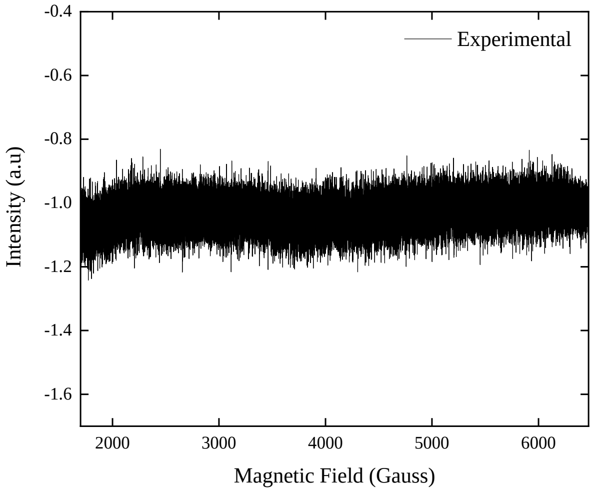

Supplementary Fig. 44 The EPR spectrum of the reaction mixture for the synthesis of **17**

2.5 **Kinetic Studies**

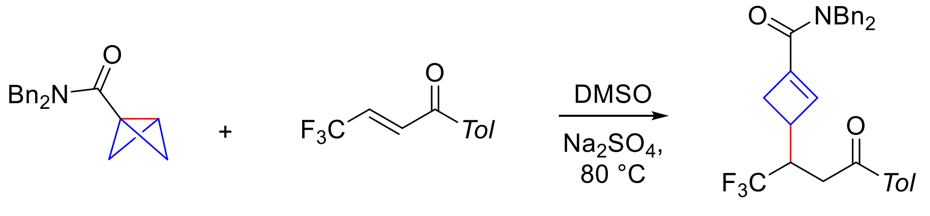

Plot of product concentration versus time for the reaction of 1a and 2aa at 90 °C.

These reactions were conducted with general procedure C. Na_2SO_4 (25.0 mg, 0.17 mmol), **1a** (81.3 mg, 0.3 mmol) and **2aa** (21.4 mg, 0.1 mmol) in DMSO (0.1 mL) were charged into several 10 mL pressure tube under argon. These reaction tubes were then sealed and placed into a metal bath at 90 °C. These reactions were monitored and removed the reactions for yield analysis one by one after the interval of 5 min, 10 min, 15 min, 20 min, 25 min. These pressure tubes were cooled in an ice water bath and then immediately extracted with CH_2Cl_2 and H_2O, the org. layers were dried over Na_2SO_4, and concentrated under reduced pressure. All samples were analyzed by ^{19}F NMR spectroscopy to determine the conversion and yields of **4a** with (trifluoromethyl)benzene as an internal standard.

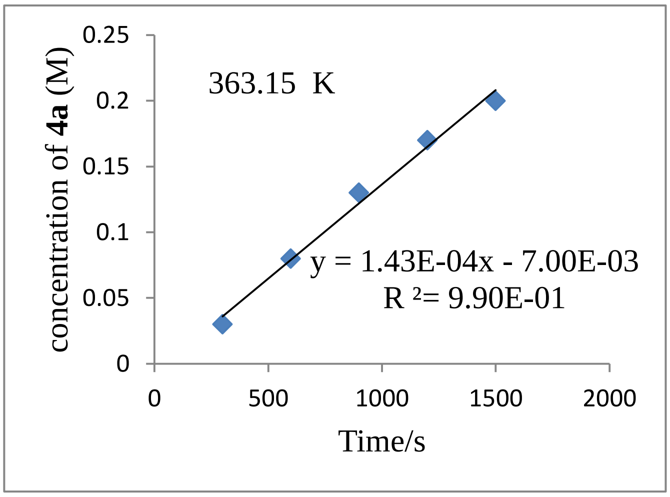

Supplementary Fig. 45 Plot of initial reaction rate at 90 °C.

Plot of product concentration versus time for the reaction of 1a and 2aa at 80 °C.

These reactions were conducted with general procedure C. Na_2SO_4 (25.0 mg, 0.17 mmol), **1a** (81.3 mg, 0.3 mmol) and **2aa** (21.4 mg, 0.1 mmol) in DMSO (0.1 mL) were charged into several 10 mL pressure tubes under argon. These reaction tubes were then sealed and placed into a metal bath at 80 °C. These reactions were monitored and removed the reactions for yield analysis one by one after the interval of 10 min, 15 min, 20 min, 25 min, 30 min, 40 min, 50 min. These pressure tubes were

cooled in an ice water bath and then immediately extracted with CH_2Cl_2 and H_2O, the org. layers were dried over Na_2SO_4, and concentrated under reduced pressure. All samples were analyzed by ^{19}F NMR spectroscopy to determine the conversion and yields of **4a** with (trifluoromethyl)benzene as an internal standard.

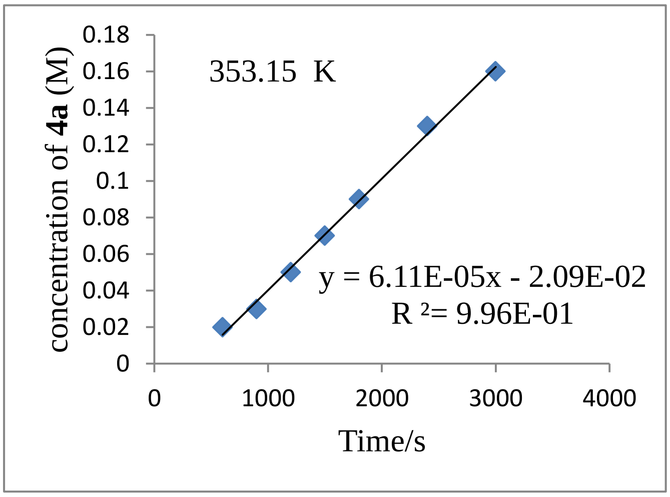

Supplementary Fig. 46 Plot of initial reaction rate at 80 °C.

Plot of product concentration versus time for the reaction of 1a and 2aa at 70 °C.

These reactions were conducted with general procedure C. Na_2SO_4 (25.0 mg, 0.17 mmol), **1a** (81.3 mg, 0.3 mmol) and **2aa** (21.4 mg, 0.1 mmol) in DMSO (0.1 mL) were charged into several 10 mL pressure tubes under argon. These reaction tubes were then sealed and placed into a metal bath at 70 °C. These reactions were monitored and removed the reactions for yield analysis one by one after the interval of 10 min, 20 min, 30 min, 40 min, 50 min, 70 min, 90 min. These pressure tubes were cooled in an ice water bath and then immediately extracted with CH_2Cl_2 and H_2O, the org. layers were dried over Na_2SO_4, and concentrated under reduced pressure. All samples were analyzed by ^{19}F NMR spectroscopy to determine the conversion and yields of **4a** with (trifluoromethyl)benzene as an internal standard.

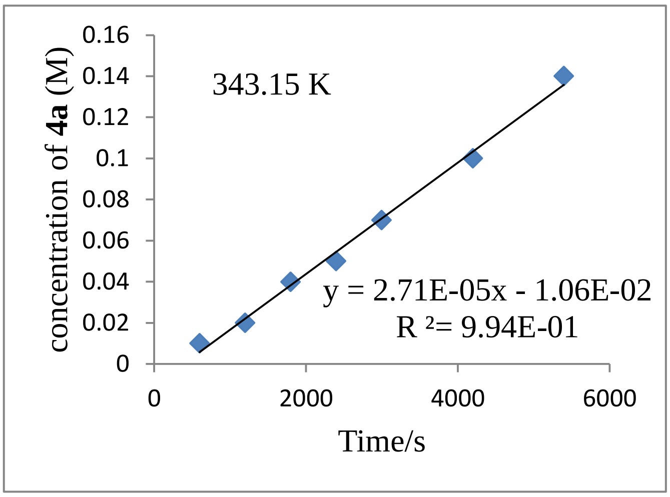

Supplementary Fig. 47 Plot of initial reaction rate at 70 °C.

Plot of product concentration versus time for the reaction of 1a and 2aa at 60 °C.

These reactions were conducted with general procedure C. Na_2SO_4 (25.0 mg, 0.17 mmol), **1a** (81.3 mg, 0.3 mmol) and **2aa** (21.4 mg, 0.1 mmol) in DMSO (0.1 mL) were charged into several 10 mL pressure tubes under argon. These reaction tubes were then sealed and placed into a metal bath at 60 °C. These reactions were monitored and removed the reactions for yield analysis one by one after the interval of 25 min, 35 min, 45 min, 60 min, 90 min, 120 min, 150 min. These pressure tubes were cooled in an ice water bath and then immediately extracted with CH_2Cl_2 and H_2O, the org. layers were dried over Na_2SO_4, and concentrated under reduced pressure. All samples were analyzed by ^{19}F NMR spectroscopy to determine the conversion and yields of **4a** with (trifluoromethyl)benzene as an internal standard.

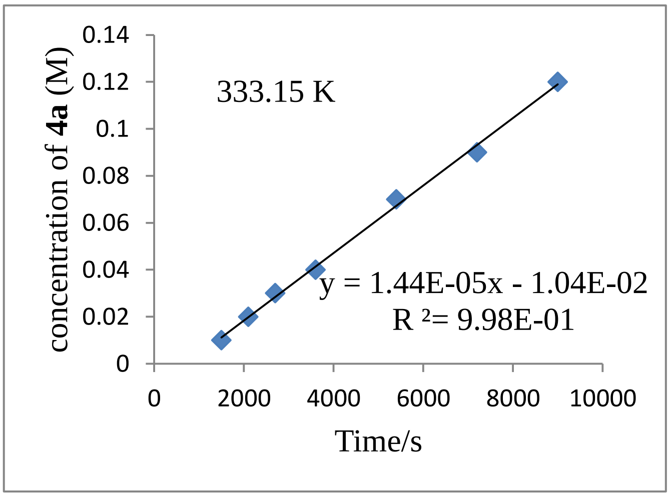

Supplementary Fig. 48 Plot of initial reaction rate at 60 °C.

Derivation of activation parameters

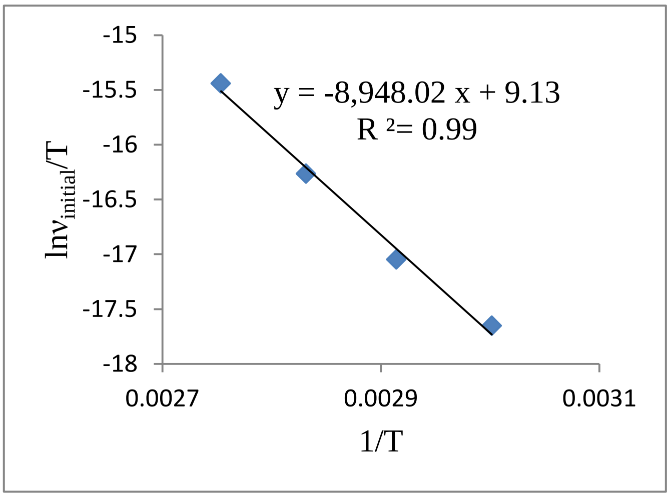

Supplementary Fig. 49 Eyring Plot of $\ln v_{initial}/T$ versus 1/T

Gibbs free energy of activation from Eyring equation

$$v_{initial} = \frac{d[\mathbf{4a}]}{dt} = k[\mathbf{1a}][\mathbf{2aa}] \Rightarrow \ln\frac{v_{initial}}{[\mathbf{1a}][\mathbf{2aa}]\mathrm{T}} = ln\frac{k}{T}$$

$$k = \frac{k_B T}{h} e^{-\Delta G^{\ddagger}/RT} \Rightarrow \ln\frac{k}{T} = -\frac{\Delta H}{RT} + \frac{\Delta S}{R} + ln\frac{k_B}{h}$$

$$\Rightarrow \ln\frac{v_{initial}}{[\mathbf{1a}][\mathbf{2a}]T} = -\frac{\Delta H}{RT} + \frac{\Delta S}{R} + ln\frac{k_B}{h}$$

$$\Delta H = 17.797\ kcal/mol$$

$$\Delta S = -0.0291\ kcal/mol.K$$

$$\Delta G\ (353\ K) = 28.1\ kcal/mol$$

Plot of initial yield versus time under different concentration of [1a].

These reactions were conducted with general procedure C. Na_2SO_4 (25.0 mg, 0.17 mmol), **1a** (41.6 mg, 0.15 mmol) and **2aa** (21.4 mg, 0.1 mmol) in DMSO (0.1 mL) were charged into several 10 mL pressure tubes under argon. These reaction tubes were then sealed and placed into a metal bath at 80 °C. These reactions were monitored and removed the reactions for yield analysis one by one after the interval of 10 min, 20 min, 30 min, 40 min, 50 min, 60 min. These pressure tubes were cooled in an ice water bath and then immediately extracted with CH_2Cl_2 and H_2O, the org. layers were dried over Na_2SO_4, and concentrated under reduced pressure. All samples were analyzed by ^{19}F NMR spectroscopy to determine the conversion and yields of **4a** with (trifluoromethyl)benzene as an internal standard. The same experimental procedure was repeated using 0.15, 0.18, 0.20, 0.24, 0.26 mmol of **1a** under otherwise identical conditions, and their corresponding conversions were measured over time.

(a) Initial Rate = $2.88*10^{-5}$M·s^{-1}

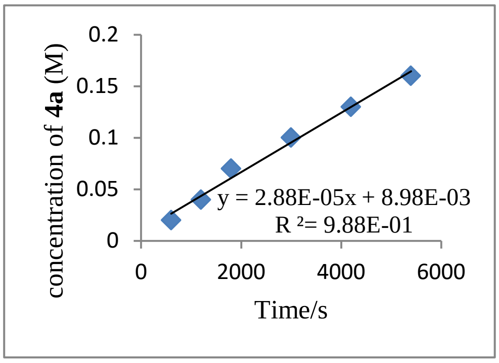

(b) Initial Rate = $3.64*10^{-5}$M·s^{-1}

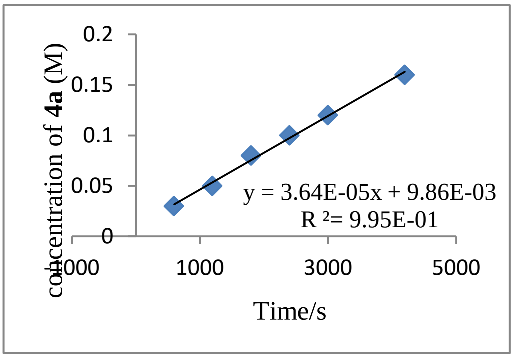

(c) Initial Rate = $4.19*10^{-5}$M·s^{-1}

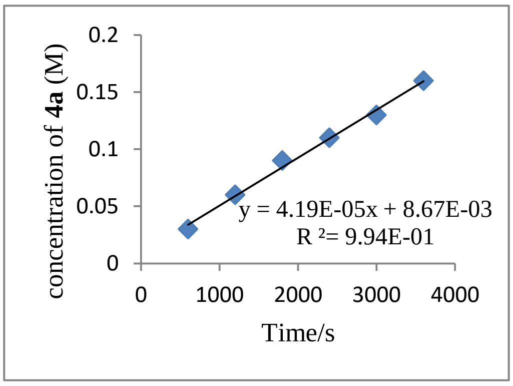

(d) Initial Rate = $4.83*10^{-5}$M·s^{-1}

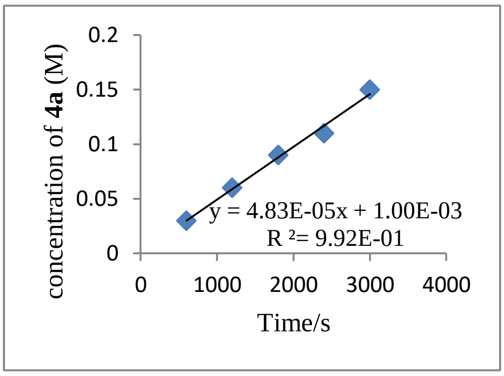

(e) Initial Rate = $5.57*10^{-5}$M·s^{-1}

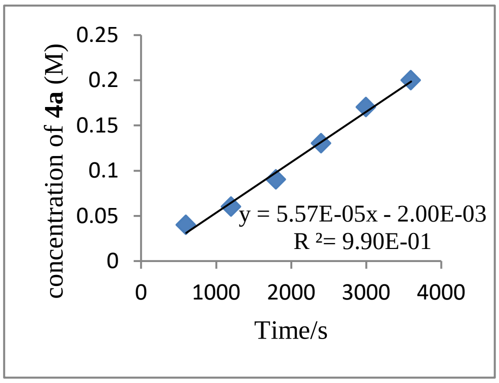

(f) Plot of Initial Rate *vs* [**1a**]

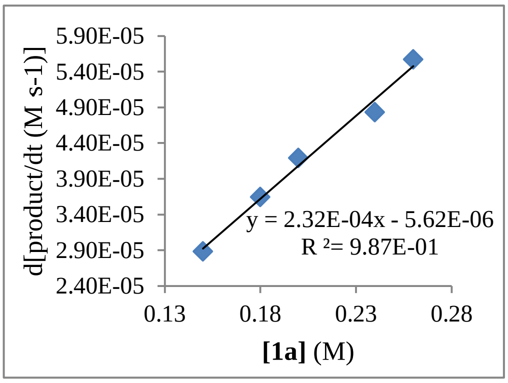

Supplementary Fig. 50. (a), (b), (c), (d), (e) initial rates for the formation of **4a** using 0.15, 0.18, 0.20, 0.24, 0.26 mmol of [**1a**], respectively. (f) plot of yield versus time with different [**1a**].

Plot of initial yield versus time under different concentration of [2aa].

These reactions were conducted with general procedure C. Na_2SO_4 (25.0 mg, 0.17 mmol), **1a** (81.3 mg, 0.3 mmol) and **2aa** (6.4 mg, 0.03 mmol) in DMSO (0.1 mL) were charged into several 10 mL pressure tubes under argon. These reaction tubes were then sealed and placed into a metal bath at 80 ^{o}C. These reactions were monitored and removed the reactions for yield analysis one by one after the interval of 10 min, 15 min, 20 min, 25 min, 35 min, 45 min. These pressure tubes were cooled in an ice water bath and then immediately extracted with CH_2Cl_2 and H_2O, the org. layers were dried over

Na_2SO_4, and concentrated under reduced pressure. All samples were analyzed by ^{19}F NMR spectroscopy to determine the conversion and yields of **4a** with (trifluoromethyl)benzene as an internal standard. The same experimental procedure was repeated using 0.03, 0.04, 0.05, 0.06, 0.08 mmol of **2aa** under otherwise identical conditions, and their corresponding conversions were measured over time.

(a) Initial Rate = $2.41*10^{-5} M\cdot s^{-1}$

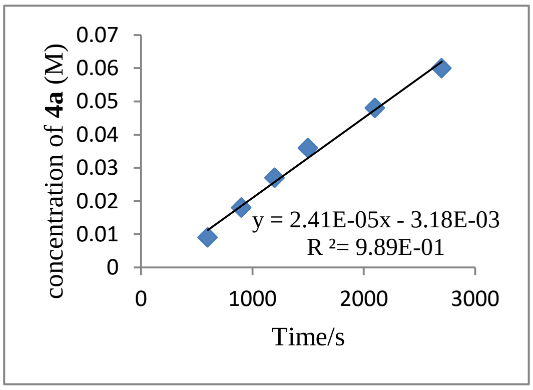

(b) Initial Rate = $3.02*10^{-5} M\cdot s^{-1}$

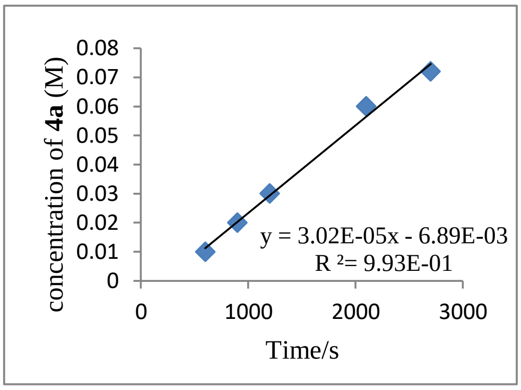

(c) Initial Rate = $3.92*10^{-5} M\cdot s^{-1}$

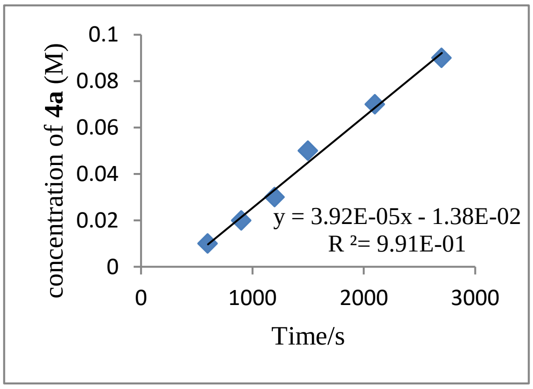

(d) Initial Rate = $4.61*10^{-5} M\cdot s^{-1}$

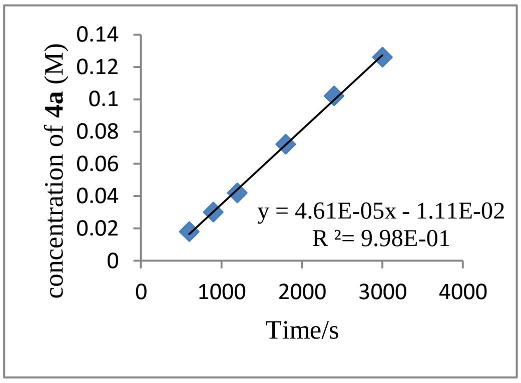

(e) Initial Rate = $5.50*10^{-5} M\cdot s^{-1}$

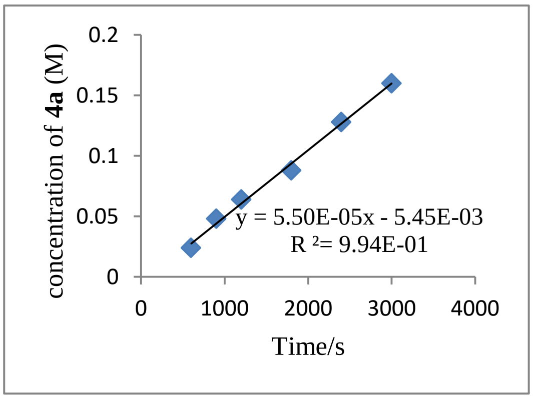

(f) Plot of Initial Rate *vs* [**2aa**]

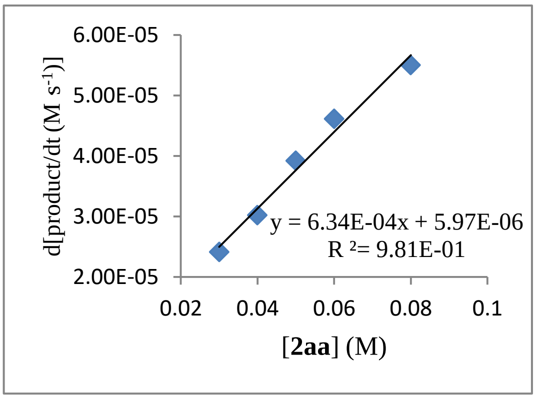

Supplementary Fig. 51. (a), (b), (c), (d), (e) initial rates for the formation of **4a** using 0.03, 0.04, 0.05, 0.06, 0.08 mmol of [**2aa**], respectively. (f) plot of yield versus time with different [**2aa**].

2.6 Computational Studies

DFT calculations were carried out by Gaussian 09 program[4] to explore the possible mechanisms and chemoselectivity of the selected model reaction of **1a** with β-trifluoromethyl enone **2aa** depicted in Supplementary Figure S5. Geometry optimizations were completed at the M06-2X[5]/def2-SVP[6] level in explicit solvent DMSO with the solvation model based on density (SMD)[7]. Single-point energy corrections were then obtained at the M06-2X/ma-TZVPP[8]/SMD_{DMSO} level. Since the radical mechanism has been ruled out by the control experiments, we have just considered and investigated three possible non-radical pathways (Supplementary Figure S1), including direct C-C σ bond activation pathway (Path A), S_N2 type C-C σ bond activation pathway (Path B), and C-C σ bond activation together with proton transfer pathway (Path C). We have tried but are failed to locate any transition states and intermediates involved in pathway B, and the reaction coordinate scanning results shown in Supplementary Figure S2 and Supplementary Figure S3 indicate that the energy would become higher and higher along the reaction coordinates of Paths A and B, so we can exclude the possibility of these two paths. At the same time, we have located the transition state **TS1** of the concerted path C, which has been further confirmed by the corresponding intrinsic reaction coordinate (IRC) results of **TS1** (Supplementary Figure S4).

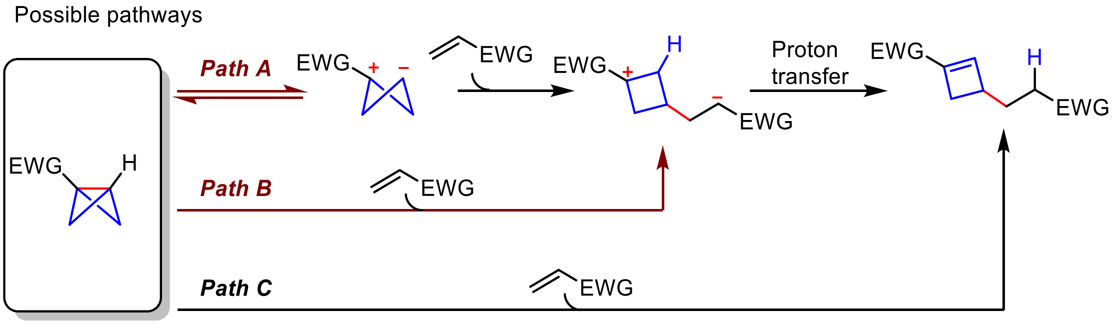

Supplementary Figure 52. Possible pathways.

Path A: direct C-C σ bond activation pathway

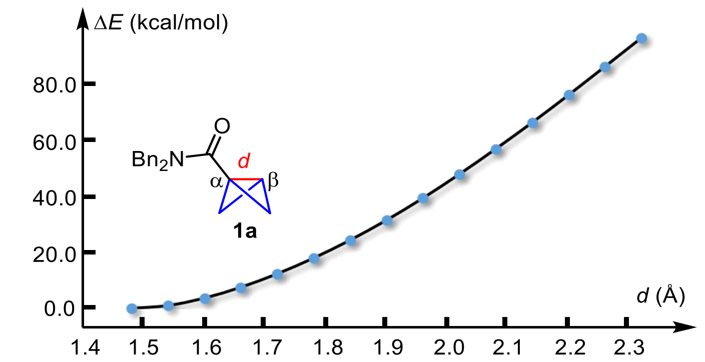

Supplementary Figure 53. Scanning curve along the distance of C_α-C_β for the reaction of **1a**

Path B: S_N2 type C-C σ bond activation pathway

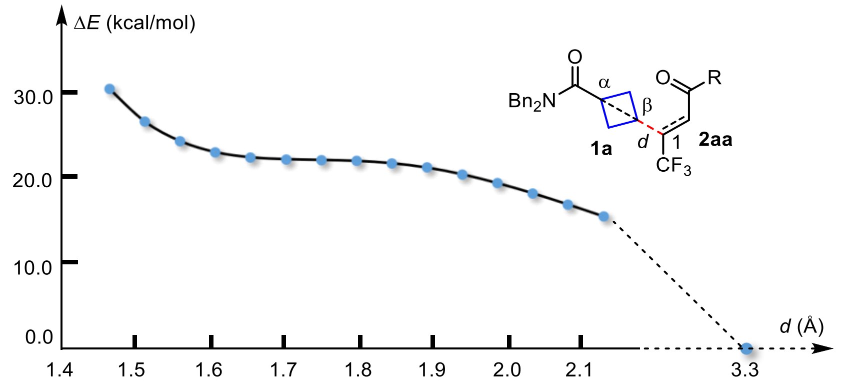

Supplementary Figure 54. Scanning curve along the distance of C_β-C_1 for the reaction between substrates **1a** and **2aa**

Path C: C-C σ bond activation coupled with proton transfer pathway

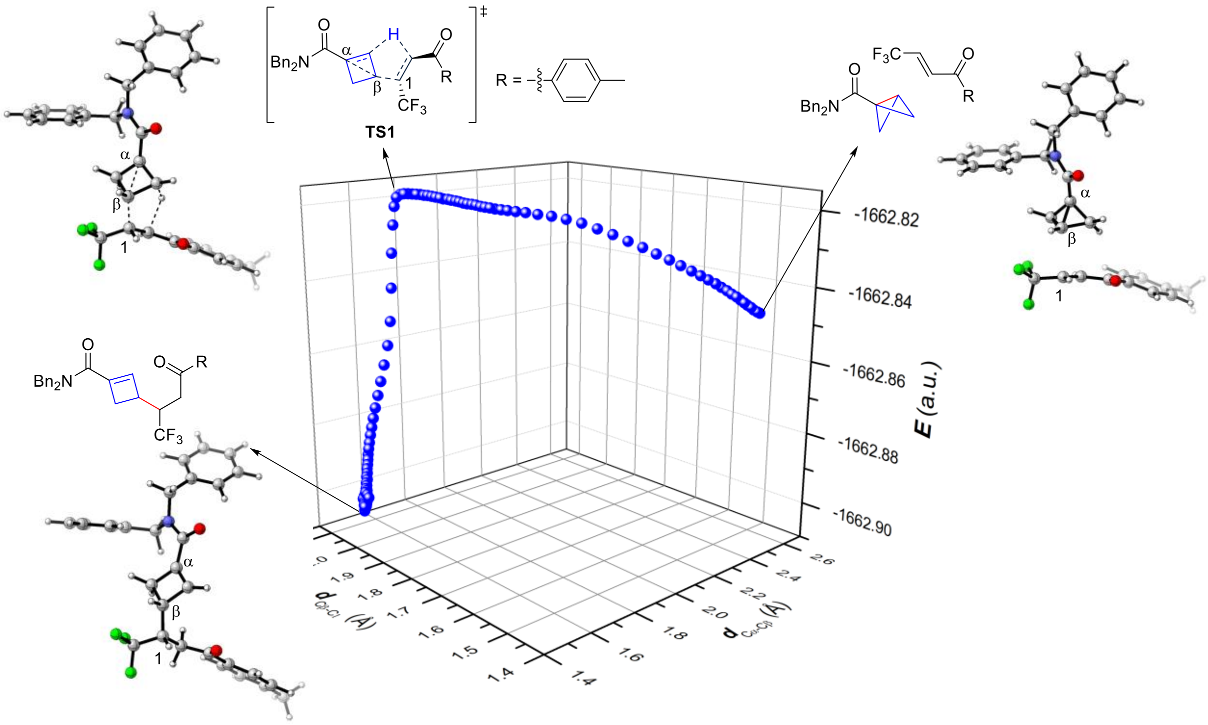

Supplementary Figure 55. IRC plot of transition state TS1.

As shown in Supplementary Figure S5, both the α site and the β site of **1a** were considered to be electrophilically attacked by β-trifluoromethyl enone **2aa**, and the calculated results demonstrate that the energy barrier of electrophilic attack on β site via transition state **TS1** ($\Delta G^{\ddagger}$ =27.9 kcal/mol) is 6.9 kcal/mol lower than that of electrophilic attack on α site of **1a** via transition state TS1′ ($\Delta G^{\ddagger}$ =34.8 kcal/mol). Therefore, **4a** is the main product in kinetics, which is consistent with the chemoselectivity in experiment. In addition, when the reaction temperature is raised from 80 °C to 130 °C, the four-membered ring opening of **4a** could irreversibly occur to generate the diene product **5a** via transition state **TS2** ($\Delta G^{\ddagger}$ = 32.9 kcal/mol). All the calculated results are consistent with the experimental results.

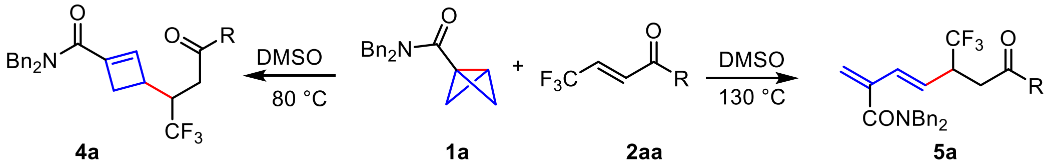

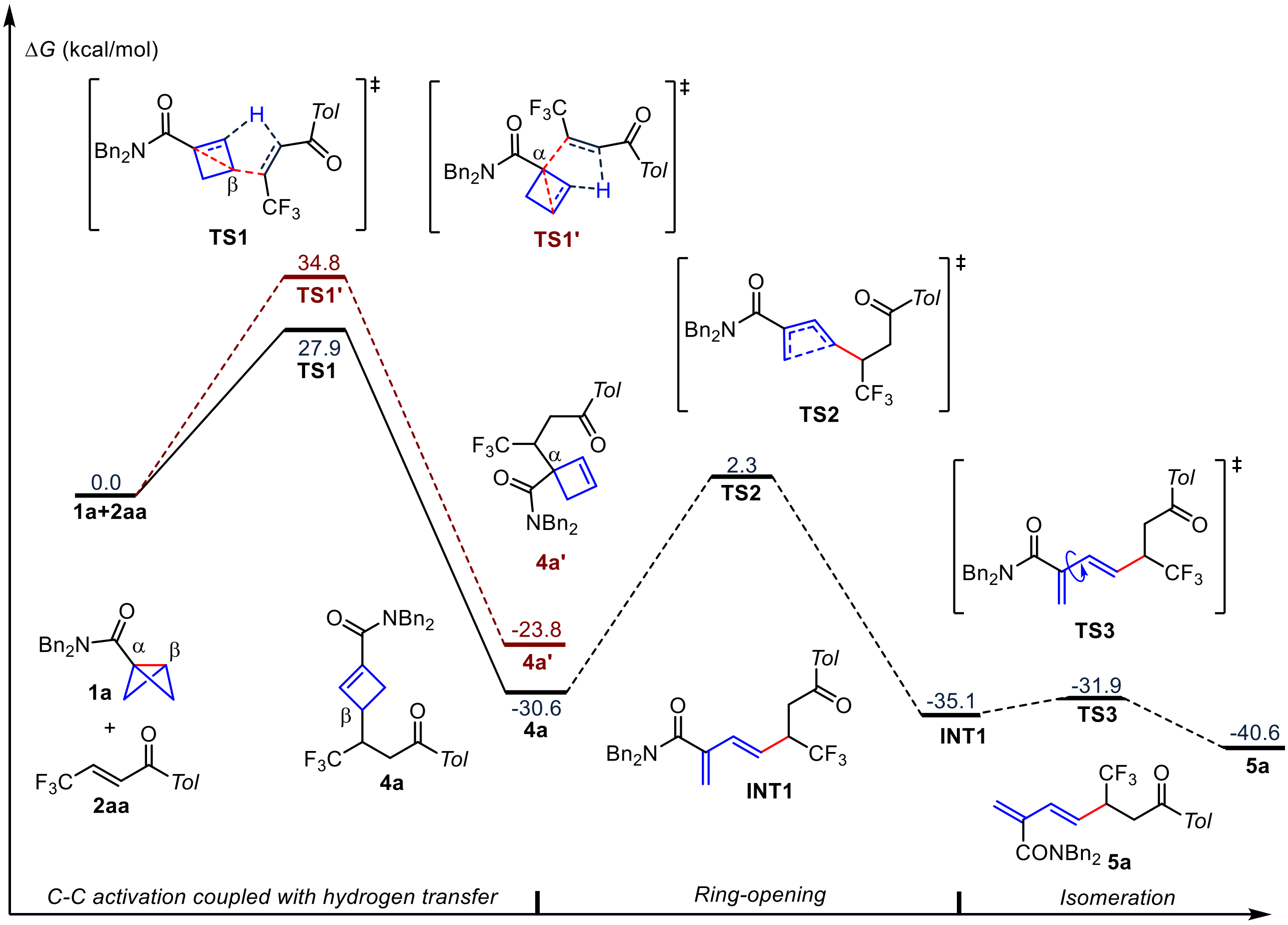

Supplementary Figure 56. The corresponding energy profiles of the chemoselective pathways for

the selected model reaction between **1a** and β-trifluoromethyl enone **2aa**

When the substrate has a phenyl group on the β site, the calculated results shown in Supplementary Figure S6, indicate that the energy barrier for electrophilic attack on the α site of the **1a′** via transition state **TS4** ($\Delta G^{\ddagger}$ = 24.9 kcal/mol) is 12.8 kcal/mol lower than that for electrophilic attack on the β site via transition state TS4′ ($\Delta G^{\ddagger}$ =37.7 kcal/mol). Thus, electrophilic attack on the α site should be more energetically favorable, and the chemoselectivity could be reversed by replacing the substrate **1a** by **1a′** with phenyl substituent at the β site, which is in agreement with the experimental result.

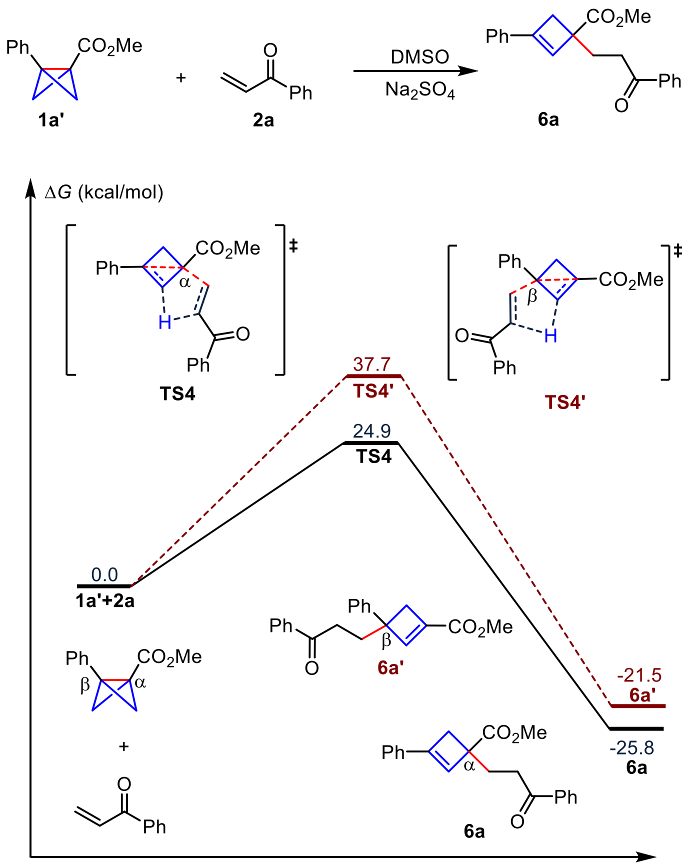

Supplementary Figure 57. The effect of β site substitution of BCBs in polarity-reversed C-C σ-bond conjugate addition.

Supplementary Table 1. The single point energies (E), Gibbs free energy corrections (GFEC), Gibbs free energies (GFE=*E*+GFEC) of the stationary points (SPs), and imaginary frequency involved in the reaction models (unit: a.u.)

SP	GFEC	E	GFE	Imaginary Frequency

**1a+2aa**	0.442954	-1664.737373	-1664.294419	
**TS1**	0.444326	-1664.694313	-1664.249987	157.8849*i*
**4a**	0.450636	-1664.793793	-1664.343157	
**TS1'**	0.444138	-1664.683049	-1664.238911	345.7498*i*
**4a'**	0.451528	-1664.783814	-1664.332286	
**TS2**	0.444878	-1664.735565	-1664.290687	571.6445*i*
**INT1**	0.447133	-1664.797532	-1664.350399	
**1a'+2a**	0.303841	-1037.863853	-1037.560012	
**TS4**	0.307075	-1037.827426	-1037.520351	26.0418*i*
**6a**	0.311742	-1037.912811	-1037.601069	
**TS4'**	0.304298	-1037.804232	-1037.499934	247.0009*i*
**6a'**	0.309297	-1037.903631	-1037.594334	

Geometrical Coordinates of the Listed Compounds

1a+2aa

C	0.376358	0.034925	-0.891253
C	-0.513004	-0.928254	-1.596528
C	-0.123756	-1.182915	-0.181287
H	0.592265	-1.991453	-0.006827
H	-0.878759	-1.049388	0.606188
C	-0.927488	0.487883	-1.481846
H	-0.860834	1.101171	-2.383955
H	-1.732506	0.746887	-0.779071
H	-0.257618	-1.498287	-2.487369
C	1.710855	0.451519	-1.396755
O	1.814423	0.948242	-2.511077
N	2.791428	0.252785	-0.587763
C	4.120335	0.558671	-1.110141
H	4.075027	0.469398	-2.202042
H	4.820385	-0.198317	-0.726383
C	2.729662	-0.105539	0.814807
H	3.366071	0.595228	1.380900
H	1.707567	0.046409	1.184923
C	3.178090	-1.522883	1.118010
C	3.490794	-1.869693	2.438036
C	3.267415	-2.500716	0.122976
C	3.873215	-3.170401	2.759641
H	3.430834	-1.108549	3.220451
C	3.654690	-3.804014	0.442993
H	3.032898	-2.239888	-0.912306
C	3.955728	-4.143556	1.761170
H	4.111514	-3.425829	3.794069
H	3.722344	-4.557045	-0.344647
H	4.257143	-5.162439	2.011131
C	4.597431	1.939912	-0.716322
C	3.990192	3.077163	-1.267629
C	5.625180	2.104960	0.217056
C	4.406032	4.352435	-0.890136
H	3.188955	2.944750	-1.998350
C	6.043530	3.383024	0.595764
H	6.102091	1.222368	0.651490
C	5.434486	4.508476	0.043076
H	3.928958	5.231164	-1.328681
H	6.845822	3.497934	1.327106
H	5.759516	5.508025	0.338010
C	-3.411671	-2.330114	-0.896616
C	-3.720116	-1.282738	-0.134868
H	-3.690075	-2.374549	-1.953145
H	-3.440085	-1.259936	0.918876
C	-4.462007	-0.128397	-0.743447
O	-4.877074	-0.204027	-1.881119
C	-2.683216	-3.524814	-0.372782
F	-2.252337	-3.367054	0.880312
F	-1.616454	-3.816212	-1.125749
F	-3.467541	-4.610061	-0.384497

C	-4.675087	1.100098	0.077555	C	-5.131277	3.484688	1.516043
C	-4.072742	1.303083	1.325247	H	-3.817891	2.632608	2.998966
C	-5.504591	2.104057	-0.447015	H	-6.385378	4.043202	-0.154066
H	-3.412747	0.551839	1.760009	C	-5.386610	4.756551	2.273957
C	-4.298641	2.485031	2.029549	H	-6.457312	4.862453	2.505326
H	-5.968364	1.945190	-1.421801	H	-4.823567	4.781224	3.215713
C	-5.731903	3.273226	0.263014	H	-5.099340	5.632558	1.673219

TS1

C	0.443962	-0.084340	-0.830865	C	3.936059	4.667013	-0.635088
C	-0.809354	-1.431754	-1.377475	H	2.806563	3.187851	-1.736697
C	0.154721	-1.353620	-0.153226	C	5.805694	3.830292	0.644174
H	0.956234	-2.087170	-0.013275	H	6.126196	1.693087	0.554000
H	-0.465432	-1.224975	0.753399	C	5.018017	4.898630	0.218358
C	-0.824459	0.123200	-1.418426	H	3.318132	5.501698	-0.971785
H	-0.954048	0.759139	-2.297799	H	6.650004	4.004857	1.313747
H	-1.697993	0.223080	-0.637161	H	5.245420	5.913475	0.549983
H	-0.344825	-1.909613	-2.246257	C	-2.282859	-1.965939	-1.056264
C	1.728469	0.497533	-1.354423	C	-3.018514	-1.032889	-0.199558
O	1.726470	0.990689	-2.471790	H	-2.770602	-2.045469	-2.039044
N	2.824700	0.416625	-0.562432	H	-3.056592	-1.200709	0.876036
C	4.104109	0.888080	-1.089673	C	-3.911405	-0.110051	-0.842820
H	4.048834	0.837742	-2.183583	O	-3.992691	-0.022590	-2.073177
H	4.886279	0.194755	-0.747904	C	-2.178250	-3.359921	-0.492917
C	2.811744	0.044307	0.841359	F	-1.710835	-3.378122	0.764764
H	3.324921	0.837485	1.409508	F	-1.350767	-4.130008	-1.214508
H	1.778566	0.038766	1.214328	F	-3.361927	-3.979647	-0.472115
C	3.480519	-1.284568	1.132880	C	-4.754636	0.809203	0.010051
C	3.887976	-1.565437	2.442040	C	-4.655601	0.894711	1.403493
C	3.679492	-2.247258	0.138640	C	-5.685890	1.628223	-0.642306
C	4.472085	-2.791309	2.755032	H	-3.935578	0.284926	1.950909
H	3.744917	-0.813077	3.221844	C	-5.469370	1.772907	2.119415
C	4.270519	-3.473425	0.450078	H	-5.755890	1.562210	-1.728917
H	3.373840	-2.035069	-0.889154	C	-6.497927	2.499730	0.074497
C	4.665746	-3.750064	1.758165	C	-6.403773	2.588172	1.471395
H	4.782758	-2.997909	3.780982	H	-5.375504	1.825798	3.206645
H	4.422065	-4.215553	-0.336040	H	-7.220946	3.126949	-0.453534
H	5.128633	-4.708384	2.000332	C	-7.278967	3.540727	2.239113
C	4.434569	2.293885	-0.638917	H	-8.341860	3.368099	2.013563
C	3.646035	3.372936	-1.061966	H	-7.133007	3.432424	3.321977
C	5.511858	2.532750	0.218564	H	-7.055868	4.584049	1.967414

4a

F	2.834070	4.308600	-1.395790	O	-3.031826	1.930913	1.400671

N	-2.803010	-0.099119	0.417109	H	-0.768621	-2.951410	-1.753454
F	1.587401	5.070094	0.181169	C	-0.603489	3.073567	0.121879
F	3.603891	4.454702	0.605228	C	5.164341	-1.919673	-1.449690
C	-2.380718	1.165177	0.700312	H	5.837239	-1.889234	-2.309907
O	2.315795	0.396772	1.605653	C	5.642525	-4.386882	-1.258170
C	0.056624	1.141006	-0.362596	C	3.459972	-1.989034	0.745878
C	0.694884	2.502705	-0.527707	H	2.790290	-2.001103	1.607377
H	0.801611	2.781969	-1.589680	C	0.556704	-3.877728	1.247118
C	-1.081285	1.641826	0.152511	H	1.233654	-4.615864	1.681892
C	-2.153187	-1.000545	-0.510142	C	0.304708	-3.879510	-0.127435
H	-1.641670	-0.426341	-1.293721	H	0.785578	-4.618989	-0.771062
H	-2.943187	-1.569998	-1.026930	C	-0.052591	-2.920921	2.056562
C	-1.189778	-1.983657	0.130138	H	0.145345	-2.905907	3.130181
C	3.668742	-0.778984	0.074243	C	4.088850	-3.153553	0.316170
C	3.079119	1.722534	-0.220788	H	3.911068	-4.093548	0.844149
H	3.011383	1.508138	-1.297599	C	-5.654473	0.740149	-0.332189
H	4.100413	2.102898	-0.043908	H	-5.112591	1.638971	-0.027135
C	2.024123	2.747394	0.187113	C	-6.758068	0.832581	-1.177052
H	1.873597	2.693885	1.275540	H	-7.083979	1.808996	-1.541012
C	2.958613	0.437242	0.578803	C	-7.450872	-0.320989	-1.555336
C	-4.019127	-0.594294	1.052698	H	-8.315480	-0.248835	-2.217797
H	-4.183374	-0.001034	1.960881	C	-5.924630	-1.655642	-0.238327
H	-3.851224	-1.640895	1.347708	H	-5.594695	-2.631379	0.127511
C	4.530232	-0.754475	-1.032084	C	-7.033014	-1.564301	-1.084152
H	4.719764	0.174574	-1.571943	H	-7.568751	-2.469354	-1.377061
C	2.511502	4.144650	-0.104406	H	0.414854	0.135487	-0.596523
C	-0.917737	-1.974384	1.500235	H	-1.184679	3.762208	-0.508653
H	-1.388600	-1.224839	2.140706	H	-0.445202	3.527792	1.110569
C	4.952570	-3.137858	-0.786889	H	6.735530	-4.262964	-1.227688
C	-5.227979	-0.506900	0.146265	H	5.374519	-5.251596	-0.637254
C	-0.564114	-2.940790	-0.679584	H	5.373593	-4.612119	-2.301294

TS1'

C	-0.120499	-1.376355	0.782088	N	-2.272837	-0.247618	0.391389
C	0.135234	-3.252487	0.132097	C	-3.715680	-0.117843	0.591071
C	0.146332	-2.002215	-0.625853	H	-3.959792	0.949662	0.687486
H	-0.551550	-1.868423	-1.461070	H	-3.963000	-0.617236	1.534733
H	1.186888	-1.735311	-0.909390	C	-1.724423	0.580393	-0.673958
C	0.535686	-2.680356	1.352929	H	-0.689299	0.293250	-0.900255
H	0.390581	-3.132790	2.336954	H	-2.299208	0.390539	-1.596155
H	1.629258	-2.237609	1.194311	C	-1.753048	2.067243	-0.376697
H	-0.275007	-4.237058	-0.112404	C	-1.785731	2.973864	-1.442234
C	-1.625303	-1.259544	1.035676	C	-1.699804	2.560720	0.930786
O	-2.195176	-2.052257	1.766103	C	-1.753112	4.347857	-1.206536

H	-1.837622	2.596612	-2.466764	H	2.909911	-0.580936	1.662676
C	-1.671863	3.936097	1.168078	C	2.640788	0.064882	-0.410842
H	-1.687954	1.862178	1.769469	O	1.880320	0.606831	-1.226451
C	-1.695207	4.833700	0.100899	C	0.622416	0.136121	2.675056
H	-1.778399	5.042868	-2.047937	F	1.165583	-0.818728	3.444035
H	-1.632661	4.306071	2.194434	F	-0.660964	0.237585	3.057963
H	-1.674391	5.909022	0.286715	F	1.211973	1.286482	3.010782
C	-4.500505	-0.721837	-0.552804	C	4.103316	-0.062974	-0.758493
C	-5.294835	0.077908	-1.378712	C	5.002116	-0.852774	-0.032930
C	-4.405450	-2.094604	-0.820699	C	4.581681	0.641120	-1.871476
C	-5.992344	-0.481847	-2.451923	H	4.660596	-1.433335	0.825869
H	-5.363560	1.150831	-1.180906	C	6.343682	-0.925797	-0.406828
C	-5.097706	-2.653737	-1.892785	H	3.876016	1.248035	-2.440614
H	-3.786098	-2.722763	-0.175022	C	5.920825	0.570047	-2.238503
C	-5.894338	-1.847952	-2.710985	C	6.827755	-0.215024	-1.511033
H	-6.609807	0.154054	-3.089356	H	7.029094	-1.550269	0.171007
H	-5.020031	-3.724497	-2.091091	H	6.276315	1.131937	-3.106334
H	-6.435830	-2.288345	-3.550319	C	8.272378	-0.293348	-1.922000
C	0.776683	-0.146172	1.197315	H	8.714981	0.710795	-2.000887
C	2.188871	-0.435002	0.857982	H	8.861918	-0.876581	-1.202455
H	0.426840	0.755128	0.673693	H	8.373710	-0.767640	-2.910458

4a'

C	-0.263262	-1.631803	0.589488	C	-1.103842	4.520494	-0.265780
C	0.353531	-3.378040	-0.403412	H	-1.302714	3.098384	-1.878064
C	0.065694	-1.987599	-0.911304	C	-1.259335	3.615670	1.964070
H	-0.805408	-1.902842	-1.581462	H	-1.577655	1.483590	2.101023
H	0.905906	-1.441784	-1.363149	C	-1.088662	4.710470	1.117443
C	0.062456	-3.091733	0.869127	H	-0.977495	5.371812	-0.937385
H	0.036818	-3.670097	1.793444	H	-1.254285	3.755148	3.046767
H	2.390291	-1.775132	0.634020	H	-0.950230	5.710132	1.533442
H	0.663273	-4.289545	-0.919015	C	-4.476100	-0.347137	-0.820511
C	-1.759302	-1.351707	0.793978	C	-5.226073	0.600433	-1.521976
O	-2.455399	-2.176467	1.363719	C	-4.411705	-1.660242	-1.308696
N	-2.280622	-0.192601	0.294671	C	-5.908751	0.245618	-2.688091
C	-3.716113	0.034858	0.431934	H	-5.272261	1.628119	-1.152363
H	-3.882638	1.098662	0.655408	C	-5.088754	-2.014573	-2.473964
H	-4.064728	-0.557026	1.286366	H	-3.831452	-2.406165	-0.759274
C	-1.588737	0.755545	-0.559052	C	-5.841080	-1.061904	-3.166392
H	-0.585630	0.389175	-0.810671	H	-6.489994	0.996662	-3.226525
H	-2.133773	0.836765	-1.514969	H	-5.033729	-3.040627	-2.843062
C	-1.451757	2.138903	0.048111	H	-6.373108	-1.340079	-4.078076
C	-1.287155	3.243688	-0.794872	C	0.663072	-0.599454	1.275557
C	-1.439814	2.337139	1.432792	C	2.137974	-0.734764	0.893871

H	0.346501	0.422973	1.026231	H	4.643164	-1.388660	0.677660
H	2.791194	-0.475885	1.742896	C	6.204859	-0.773886	-0.660733
C	2.537148	0.175312	-0.252079	H	3.579614	1.582982	-2.257389
O	1.744121	0.941214	-0.759990	C	5.623065	0.885853	-2.304988
C	0.523123	-0.672328	2.780840	C	6.585908	0.035733	-1.736685
F	0.996401	-1.815216	3.294628	H	6.940562	-1.439389	-0.204039
F	-0.752358	-0.569079	3.176740	H	5.903504	1.525953	-3.144961
F	1.190014	0.323288	3.378319	C	7.983858	-0.002212	-2.286552
C	3.949058	0.103326	-0.738377	H	8.413284	1.009213	-2.336025
C	4.902874	-0.744706	-0.163910	H	8.639200	-0.631724	-1.670883
C	4.325296	0.920909	-1.814434	H	7.985029	-0.402494	-3.312058

TS2

F	-2.351273	-2.697626	-2.533687	C	4.840888	2.212501	-0.444635
O	2.939439	0.304879	-2.651863	C	2.746765	-1.628215	2.802457
N	3.333524	0.249453	-0.425917	H	2.305820	-0.836277	3.413640
F	-0.906952	-3.591440	-1.215152	C	0.364960	-0.746524	-2.497904
F	-3.008205	-3.835165	-0.832914	C	-6.959540	1.538966	-0.273776
C	2.556513	0.014583	-1.522721	H	-7.751658	1.407041	-1.013901
O	-2.713060	0.183076	1.458181	C	-8.307886	3.412846	0.762307
C	0.394692	-1.009896	-0.305610	C	-4.940125	1.863143	1.611197
C	-0.917658	-0.817009	-0.785689	H	-4.141570	1.978260	2.345817
H	-1.187642	0.172212	-1.163464	C	3.475350	-3.920150	2.552963
C	1.211916	-0.613523	-1.366591	H	3.606572	-4.921512	2.966763
C	2.918877	0.056431	0.946646	C	2.914229	-2.905233	3.332817
H	1.864505	0.341621	1.067104	H	2.605618	-3.111787	4.359369
H	3.488356	0.768226	1.565514	C	3.863926	-3.645390	1.243350
C	3.134686	-1.345332	1.486102	H	4.302202	-4.431934	0.626123
C	-4.804588	0.884203	0.615891	C	-6.070016	2.668099	1.657912
C	-3.426126	-1.007887	-0.473859	H	-6.164178	3.428184	2.437264
H	-3.558283	-0.534275	-1.459364	C	4.178219	3.088297	-1.316783
H	-4.253209	-1.727091	-0.359691	H	3.585382	2.670505	-2.134195
C	-2.075648	-1.706418	-0.397837	C	4.282099	4.466880	-1.145957
H	-1.894119	-2.061319	0.630479	H	3.765000	5.140194	-1.832395
C	-3.568982	0.040596	0.611927	C	5.049839	4.989377	-0.101015
C	4.701579	0.716671	-0.627349	H	5.130370	6.069777	0.032250
H	5.001758	0.431751	-1.643111	C	5.605712	2.742608	0.597912
H	5.351376	0.192381	0.088905	H	6.123911	2.065507	1.281640
C	-5.826705	0.728321	-0.327104	C	5.712085	4.125459	0.769388
H	-5.752631	-0.027808	-1.110347	H	6.312369	4.526605	1.588202
C	-2.085904	-2.960116	-1.246410	H	0.645626	-1.504251	0.637737
C	3.693138	-2.364352	0.711097	H	0.418369	-0.050065	-3.344989
H	3.994712	-2.159702	-0.318629	H	-0.070065	-1.719366	-2.723942
C	-7.099470	2.520731	0.713850	H	-9.089806	3.065553	0.074696

H	-8.725541	3.455090	1.778585	H	-8.038016	4.442723	0.481132

INT1

F	-2.862891	-3.685129	-1.589053	C	0.615682	0.482695	2.518968
O	3.961075	-1.534514	-1.995387	H	0.270884	1.449949	2.147156
N	2.998495	-0.475789	-0.244449	C	1.479719	-1.762587	-3.260893
F	-1.396712	-4.319466	-0.150803	C	-6.006755	2.149131	-0.590298
F	-3.350668	-3.714346	0.505439	H	-7.037442	1.900552	-0.852722
C	2.945577	-1.208809	-1.397923	C	-6.696432	4.575046	-0.377579
O	-1.515800	0.694200	0.171876	C	-3.378955	2.771876	0.075037
C	0.496965	-1.949127	-0.994498	H	-2.344387	3.004159	0.333479
C	-0.797807	-1.733859	-1.242370	C	0.612064	-1.223181	4.234048
H	-1.112435	-1.296015	-2.196937	H	0.272716	-1.586649	5.205799
C	1.591793	-1.633590	-1.931650	C	0.181388	0.017894	3.758671
C	1.894332	0.240903	0.370577	H	-0.495876	0.627930	4.359761
H	1.018389	0.247769	-0.290849	C	1.481111	-1.991032	3.460817
H	2.194510	1.297297	0.478472	H	1.827656	-2.960459	3.824253
C	1.482250	-0.286283	1.731930	C	-4.336731	3.774593	0.013492
C	-3.725341	1.439584	-0.193730	H	-4.055303	4.808769	0.226469
C	-3.056274	-1.043870	-0.365125	C	5.096187	1.491273	-1.431379
H	-3.494999	-1.111811	-1.374447	H	4.966213	0.706075	-2.179582
H	-3.859956	-1.307791	0.339491	C	5.539606	2.758971	-1.802958
C	-1.882235	-2.008859	-0.231965	H	5.755954	2.972523	-2.851691
H	-1.458740	-1.942485	0.782805	C	5.712029	3.754795	-0.837807
C	-2.657811	0.394179	-0.105626	H	6.059328	4.747156	-1.131577
C	4.315053	-0.168410	0.312064	C	4.989878	2.205406	0.870722
H	5.009557	-0.944087	-0.031287	H	4.769443	1.988139	1.919249
H	4.243661	-0.224904	1.408227	C	5.437274	3.475721	0.500008
C	-5.049843	1.137108	-0.528492	H	5.566947	4.249161	1.259495
H	-5.349209	0.110515	-0.744913	H	0.787428	-2.335024	-0.011599
C	-2.375363	-3.434574	-0.367193	H	2.318028	-1.510882	-3.913298
C	1.913060	-1.524607	2.216728	H	0.558575	-2.132253	-3.717581
H	2.591275	-2.133699	1.613476	H	-6.375772	5.376100	-1.059964
C	-5.668817	3.479857	-0.320554	H	-6.832748	5.032152	0.614462
C	4.817622	1.202375	-0.087796	H	-7.668300	4.195602	-0.718518

1a'+2a

C	1.239067	-1.054145	-0.593732	H	-0.371537	-1.075831	-2.143181
C	0.226774	0.038136	-0.325124	C	0.637359	1.360771	0.165582
C	0.140318	-1.264244	0.396332	O	1.073336	2.242182	-0.534244
H	0.412308	-1.259796	1.455187	C	-1.959826	3.350448	-0.372816
H	-0.688670	-1.930894	0.124818	C	-2.505104	2.232749	0.113223
C	0.435961	-0.473299	-1.708649	H	-1.650693	3.400466	-1.419867
H	0.951193	0.198086	-2.400399	H	-2.807542	2.170945	1.158943

C	-2.708958	1.052171	-0.781319	H	0.278701	3.525056	1.619246
O	-2.600752	1.157194	-1.986784	H	1.943128	2.919602	1.875548
C	-3.050054	-0.273393	-0.164194	H	-1.808791	4.232567	0.253340
C	-3.046682	-0.493489	1.219122	H	-3.807007	-3.814363	1.260542
C	-3.326490	-1.341708	-1.028405	C	2.692613	-0.977079	-0.357282
H	-2.811979	0.312720	1.914185	C	3.235998	-1.382892	0.871294
C	-3.316757	-1.764302	1.726579	C	3.554067	-0.476223	-1.345251
H	-3.321216	-1.158205	-2.103760	C	4.604251	-1.271421	1.111426
C	-3.601055	-2.608279	-0.521096	H	2.583520	-1.797906	1.641635
C	-3.595377	-2.821161	0.859776	C	4.921665	-0.366233	-1.100484
H	-3.306450	-1.929864	2.805106	H	3.153345	-0.176799	-2.315263
H	-3.818801	-3.433654	-1.201187	C	5.452629	-0.759957	0.128472
O	0.504236	1.463659	1.489791	H	5.010116	-1.594334	2.072032
C	0.878922	2.713724	2.054327	H	5.578746	0.025740	-1.879077
H	0.686532	2.638880	3.129501	H	6.524615	-0.677744	0.315852

TS4

C	1.565023	-0.588957	0.420518	C	-5.828604	-0.964444	-1.004544
C	0.728890	1.236877	0.331207	C	-6.088271	-1.682735	0.163739
C	1.353286	0.406259	1.495222	H	-5.366787	-2.202683	2.132125
H	2.213890	0.782112	2.061749	H	-6.535561	-0.999932	-1.836071
H	0.522518	0.115105	2.167527	O	2.023217	3.186180	0.421178
C	0.490932	-0.104418	-0.403206	C	2.939790	4.133020	-0.121677
H	0.468249	-0.231972	-1.489750	H	3.024300	4.938383	0.614661
H	-0.528063	-0.348780	0.073160	H	2.565273	4.529358	-1.074970
C	1.744837	2.130855	-0.340869	H	3.921751	3.668563	-0.285178
O	2.233438	1.945806	-1.425554	H	-0.384840	2.604809	1.579917
C	-0.602809	1.996789	0.690359	H	-6.996876	-2.281833	0.250067
C	-1.727033	1.060283	0.884970	C	2.666041	-1.457665	0.150231
H	-0.814305	2.657358	-0.165001	C	3.685696	-1.630347	1.111797
H	-2.043184	0.784132	1.890335	C	2.732163	-2.159201	-1.074069
C	-2.522134	0.734675	-0.246093	C	4.744331	-2.489329	0.852020
O	-2.264754	1.135247	-1.396502	H	3.634491	-1.087908	2.057552
C	-3.750995	-0.134229	-0.052698	C	3.792359	-3.018089	-1.322826
C	-4.016375	-0.866752	1.112932	H	1.946220	-2.021713	-1.818832
C	-4.665834	-0.203464	-1.110739	C	4.797104	-3.181363	-0.362251
H	-3.309664	-0.853831	1.944196	H	5.532722	-2.624742	1.593355
C	-5.175596	-1.635219	1.219146	H	3.844839	-3.563349	-2.265979
H	-4.438376	0.359634	-2.016916	H	5.631386	-3.855855	-0.563948

6a

C	-0.420413	-1.042680	0.125963	H	-2.063487	-2.045593	-0.959531
C	-2.221221	0.011013	-0.050175	H	-1.251897	-0.788269	-1.943943
C	-1.515491	-1.091569	-0.919894	C	-1.073048	-0.171199	0.917886

H	-0.851176	0.298944	1.878112
H	-0.539065	1.205423	-1.941380
C	-3.570627	-0.420304	0.481735
O	-3.799241	-0.737733	1.620630
C	-2.324329	1.396026	-0.714858
C	-0.991481	1.921198	-1.247741
H	-2.717793	2.113568	0.021958
H	-1.183112	2.840243	-1.826374
C	-0.039963	2.324554	-0.136960
O	-0.447048	2.992731	0.790382
C	1.407168	1.913582	-0.189501
C	1.975360	1.222967	-1.268219
C	2.209031	2.226115	0.918177
H	1.380708	0.962749	-2.144387
C	3.321719	0.856190	-1.238399
H	1.755770	2.761548	1.753808
C	3.549771	1.855794	0.950555
C	4.109421	1.169659	-0.130684
H	3.755569	0.320351	-2.084682
H	4.162203	2.100936	1.820221
O	-4.501026	-0.419368	-0.475310
C	-5.809920	-0.819867	-0.084222
H	-6.429771	-0.775856	-0.985727
H	-6.210443	-0.140820	0.680839
H	-5.799476	-1.843388	0.314383
H	-3.050636	1.334969	-1.537181
H	5.160914	0.876328	-0.107663
C	0.898880	-1.673777	0.170126
C	1.350524	-2.433429	-0.917725
C	1.753609	-1.473650	1.266982
C	2.637432	-2.971520	-0.917860
H	0.689116	-2.593341	-1.772764
C	3.035437	-2.014602	1.266341
H	1.409015	-0.881409	2.118374
C	3.483616	-2.759546	0.170789
H	2.980975	-3.556598	-1.773017
H	3.695700	-1.848562	2.119866
H	4.492558	-3.175996	0.169567

TS4'

C	1.342639	1.563799	0.216453
C	0.928661	-0.189830	0.883134
C	1.434357	1.098119	1.605814
H	2.407308	1.134578	2.105056
H	0.621968	1.464165	2.256093
C	0.304037	0.729775	-0.240615
H	0.198094	0.474925	-1.300047
H	-0.742148	0.854283	0.284104
C	2.392195	2.182241	-0.631085
O	2.261762	2.377571	-1.812930
C	-0.284277	-0.918652	1.698729
C	-1.564076	-0.272481	1.453768
H	-0.279583	-1.940782	1.297038
H	-2.039118	0.344057	2.216033
C	-2.290776	-0.696316	0.284460
O	-1.786981	-1.466200	-0.542354
C	-3.687522	-0.179107	0.038273
C	-4.262929	0.873969	0.761665
C	-4.433136	-0.792742	-0.976637
H	-3.699501	1.380922	1.546279
C	-5.560187	1.298443	0.474620
H	-3.968920	-1.605801	-1.536399
C	-5.731519	-0.375424	-1.257758
C	-6.298643	0.673220	-0.531464
H	-5.997431	2.123480	1.039979
H	-6.303678	-0.867984	-2.046346
O	3.482560	2.469946	0.069038
C	4.572026	3.023897	-0.665822
H	5.386428	3.164889	0.051088
H	4.881799	2.337677	-1.465483
H	4.284708	3.987738	-1.107185
H	0.023020	-0.909614	2.752283
H	-7.315468	1.004755	-0.750381
C	1.971828	-1.159570	0.381495
C	3.272863	-1.225575	0.888563
C	1.583953	-2.059316	-0.622661
C	4.177600	-2.163462	0.384562
H	3.594115	-0.550076	1.682584
C	2.487474	-2.993168	-1.123839
H	0.553883	-2.020262	-0.990490
C	3.790411	-3.045074	-0.622977
H	5.191650	-2.203825	0.786660
H	2.173543	-3.684758	-1.907917
H	4.500734	-3.775370	-1.015032

6a'

C	-0.556829	-1.530510	-0.133758
C	-1.537082	0.240645	0.382759
C	-1.318208	-1.138599	1.107258
H	-2.242992	-1.709359	1.275018
H	-0.741835	-1.096028	2.043467
C	-0.769346	-0.375042	-0.780416
H	-0.492271	-0.003816	-1.768291
H	0.918594	0.278116	1.619991
C	0.152619	-2.760160	-0.543531
O	0.737385	-2.902191	-1.588368
C	-0.853902	1.435862	1.076814
C	0.648062	1.287574	1.272666
H	-1.049977	2.341218	0.483936
H	1.001566	1.972366	2.062344
C	1.453316	1.613457	0.031921
O	0.935704	2.092173	-0.954524
C	2.933719	1.352475	0.056507
C	3.594811	0.875450	1.195576
C	3.667952	1.607399	-1.109506
H	3.044953	0.674662	2.115985
C	4.971886	0.656625	1.164405
H	3.141321	1.981447	-1.988730
C	5.041155	1.385107	-1.140446
C	5.695027	0.908700	-0.001483
H	5.481837	0.286553	2.055417
H	5.605670	1.584404	-2.053014
O	0.061944	-3.709087	0.389703
C	0.707835	-4.942146	0.093970
H	0.510759	-5.606334	0.941531
H	0.304642	-5.380784	-0.828975
H	1.790034	-4.793771	-0.024544
H	-1.343341	1.572403	2.053428
H	6.772360	0.734002	-0.022931
C	-2.983213	0.585784	0.092821
C	-3.949195	0.486729	1.102645
C	-3.371678	1.059876	-1.165398
C	-5.274479	0.844952	0.858267
H	-3.658417	0.122900	2.091750
C	-4.698286	1.417028	-1.412666
H	-2.630086	1.153103	-1.962225
C	-5.653904	1.309175	-0.402564
H	-6.015718	0.756979	1.655034
H	-4.986560	1.778882	-2.401600
H	-6.692367	1.583625	-0.597133

3. Supplementary Tables and Figures

3.1. X-Ray Crystallographic Data

Crystal structure details for **5g** (CCDC 2216789). Thermal ellipsoids are shown at 50 % probability level (two molecules in each unit).

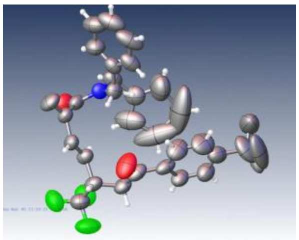

Identification code	**5g**
Empirical formula	$C_{29}H_{23}F_6NO_3$
Formula weight	547.48
Temperature/K	293
Crystal system	monoclinic
Space group	I2/a
a/Å	17.0723 (5)
b/Å	14.5508(4)
c/Å	23.8979(8)
α/°	90
β/°	108.335(3)
γ/°	90
Volume/Å^3	5635.2(3)
Z	8
ρ_{calc}g/cm^3	1.291
μ/mm^{-1}	0.950
F(000)	2256.0
Crystal size/mm^3	0.1 × 0.1 × 0.1
Radiation	Cu Kα (λ = 1.54184)
2Θ range for data collection/ °	7.794 to 142.528
Index ranges	$-12 \leq h \leq 18, -13 \leq k \leq 17, -29 \leq l \leq 28$
Reflections collected	13034
Independent reflections	5343 [R_{int} = 0.0196, R_{sigma} = 0.0203]
Data/restraints/parameters	5343/2/364
Goodness-of-fit on F^2	1.090
Final R indexes [I>=2σ (I)]	R_1 = 0.0968, wR_2 = 0.2896
Final R indexes [all data]	R_1 = 0.1200, wR_2 = 0.3153
Largest diff. peak/hole / e Å^{-3}	0.49/-0.39

Crystal structure details for 6b (CCDC 2217250). Thermal ellipsoids are shown at 50 % probability level (two molecules in each unit).

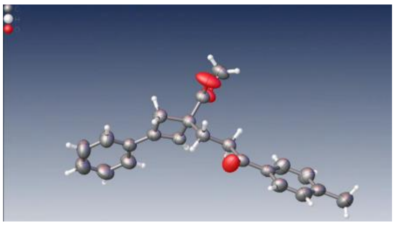

Identification code	**6b**
Empirical formula	$C_{22}H_{22}NaO_3^+$
Formula weight	334.16
Temperature/K	293(2)
Crystal system	Triclinic
Space group	P-1
a/Å	5.7361(3)
b/Å	11.8218(6)
c/Å	13.6824(6)
α/°	93.563(4)
β/°	95.962(4)
γ/°	90.638(4)
Volume/Å3	920.87(8)
Z	18
ρcalcg/cm3	1.397
μ/mm-1	1.109
F(000)	396.0
Crystal size/mm3	0.5 × 0.2 × 0.2
Radiation	Cu Kα (λ = 1.54184)
2Θ range for data collection/°	7.494 to 143.002
Index ranges	-6 ≤ h ≤ 7, -12 ≤ k ≤ 14, -16 ≤ l ≤ 16
Reflections collected	6593
Independent reflections	3478 [Rint = 0.0240, Rsigma = 0.0369]
Data/restraints/parameters	3478/0/228
Goodness-of-fit on F2	1.151
Final R indexes [I>=2σ (I)]	R1 = 0.0589, wR2 = 0.1755
Final R indexes [all data]	R1 = 0.0750, wR2 = 0.1838
Largest diff. peak/hole / e Å-3	0.18/-0.18

3.2. NMR Spectra

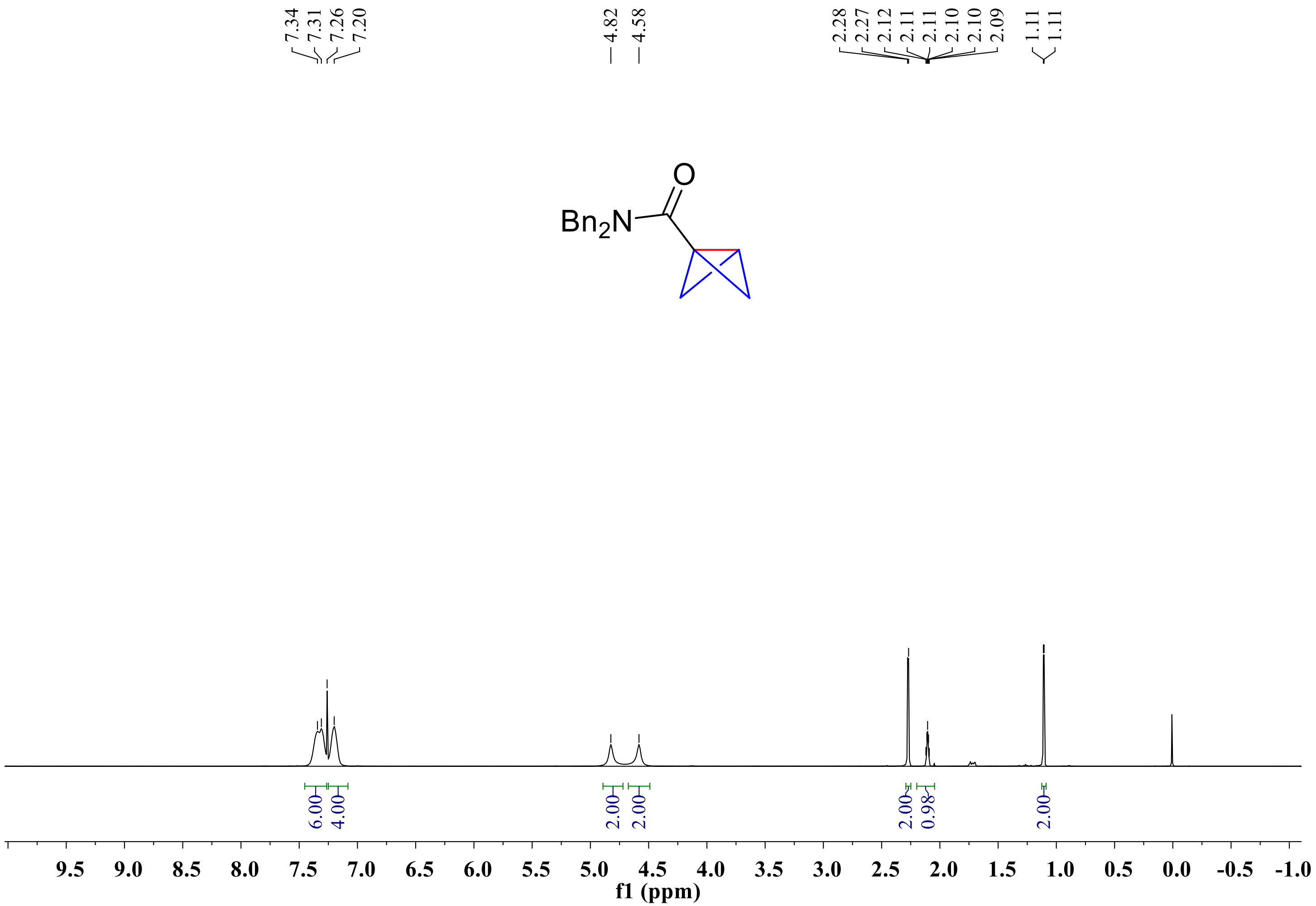

Supplementary Fig. 58 ^{1}H NMR (400 MHz, $CDCl_3$) of **1a**

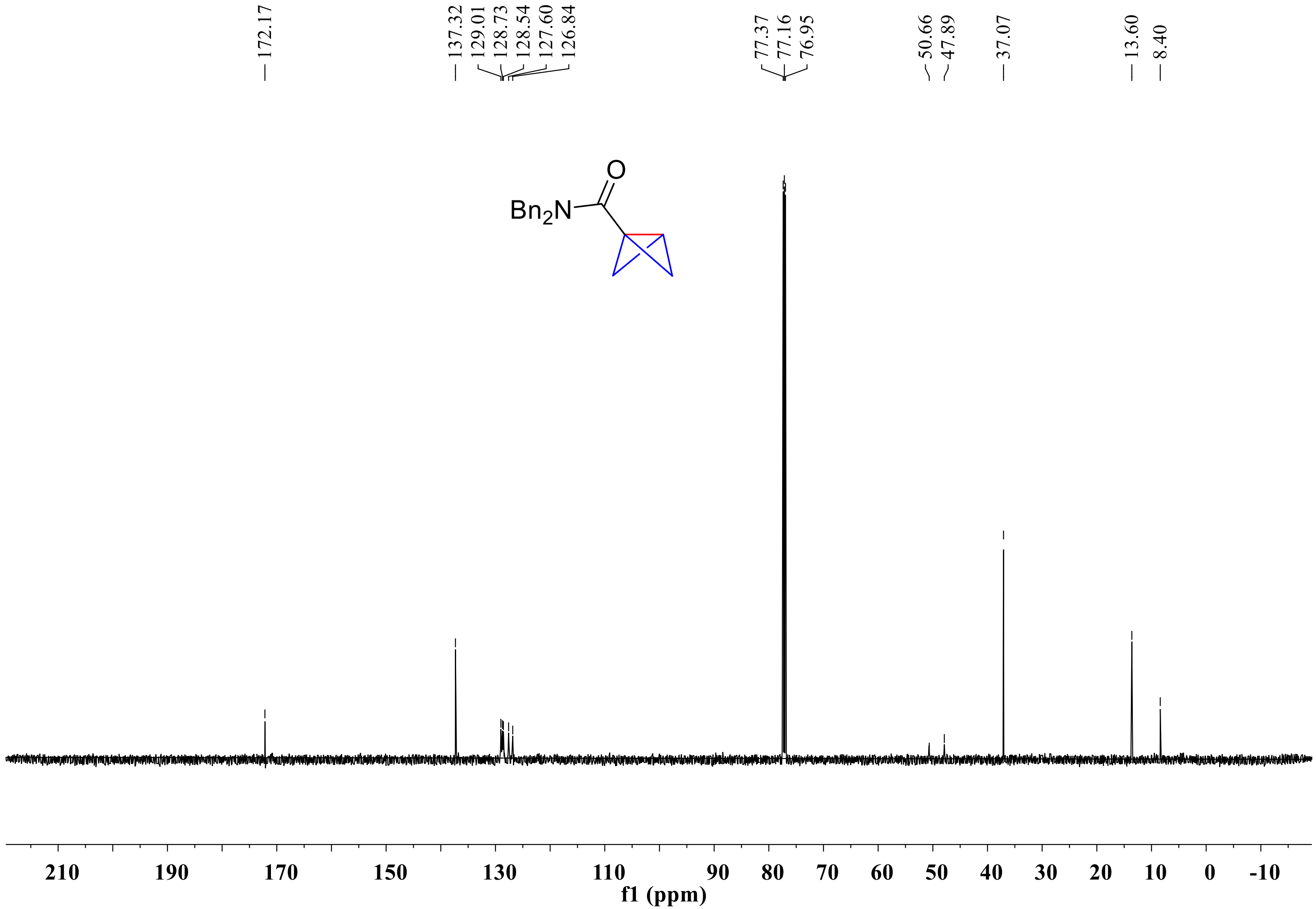

Supplementary Fig. 59 ^{13}C NMR (151 MHz, $CDCl_3$) of **1a**

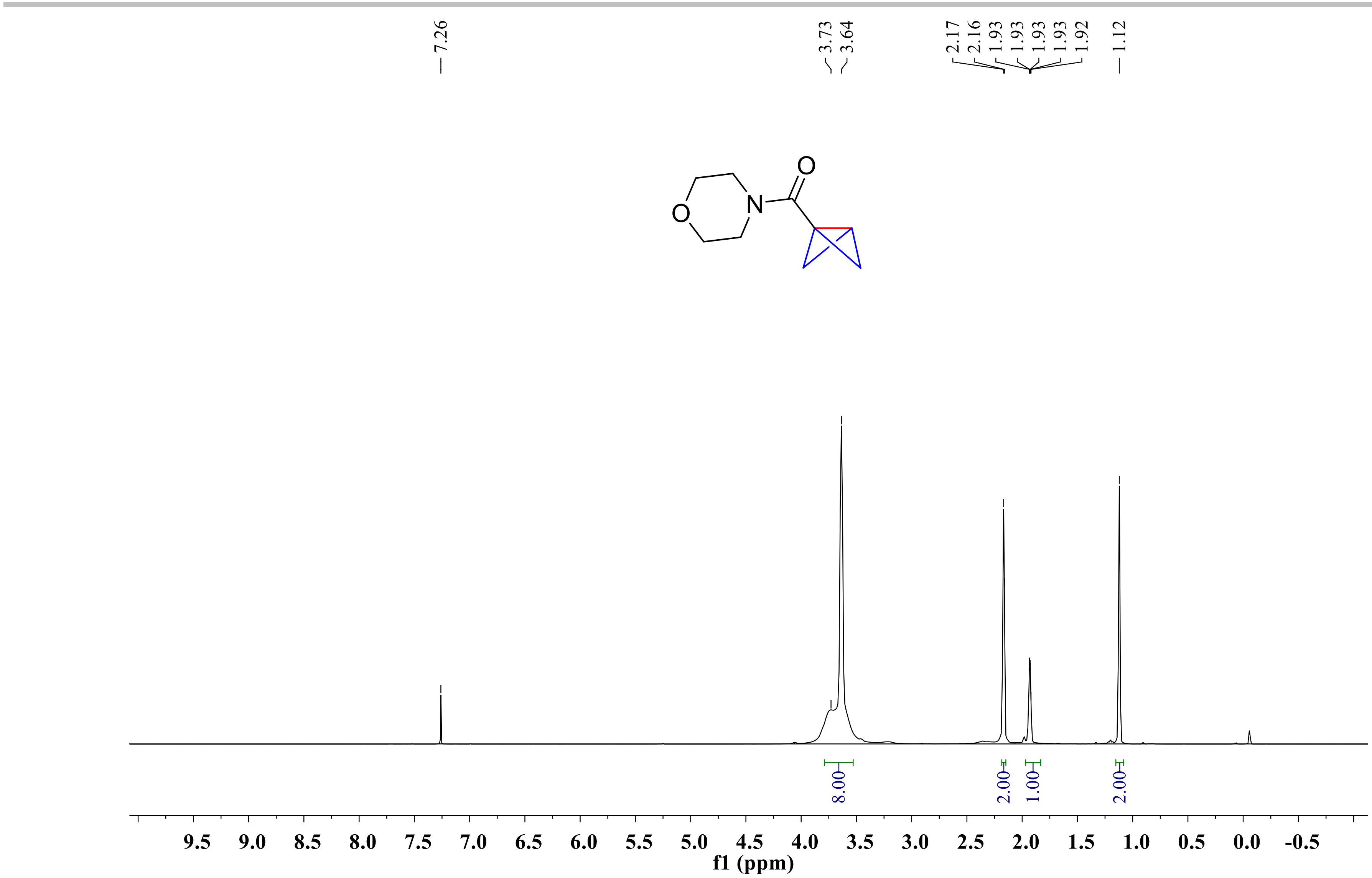

Supplementary Fig. 60 ^{1}H NMR (400 MHz, $CDCl_3$) of Morpholine amide **1t**

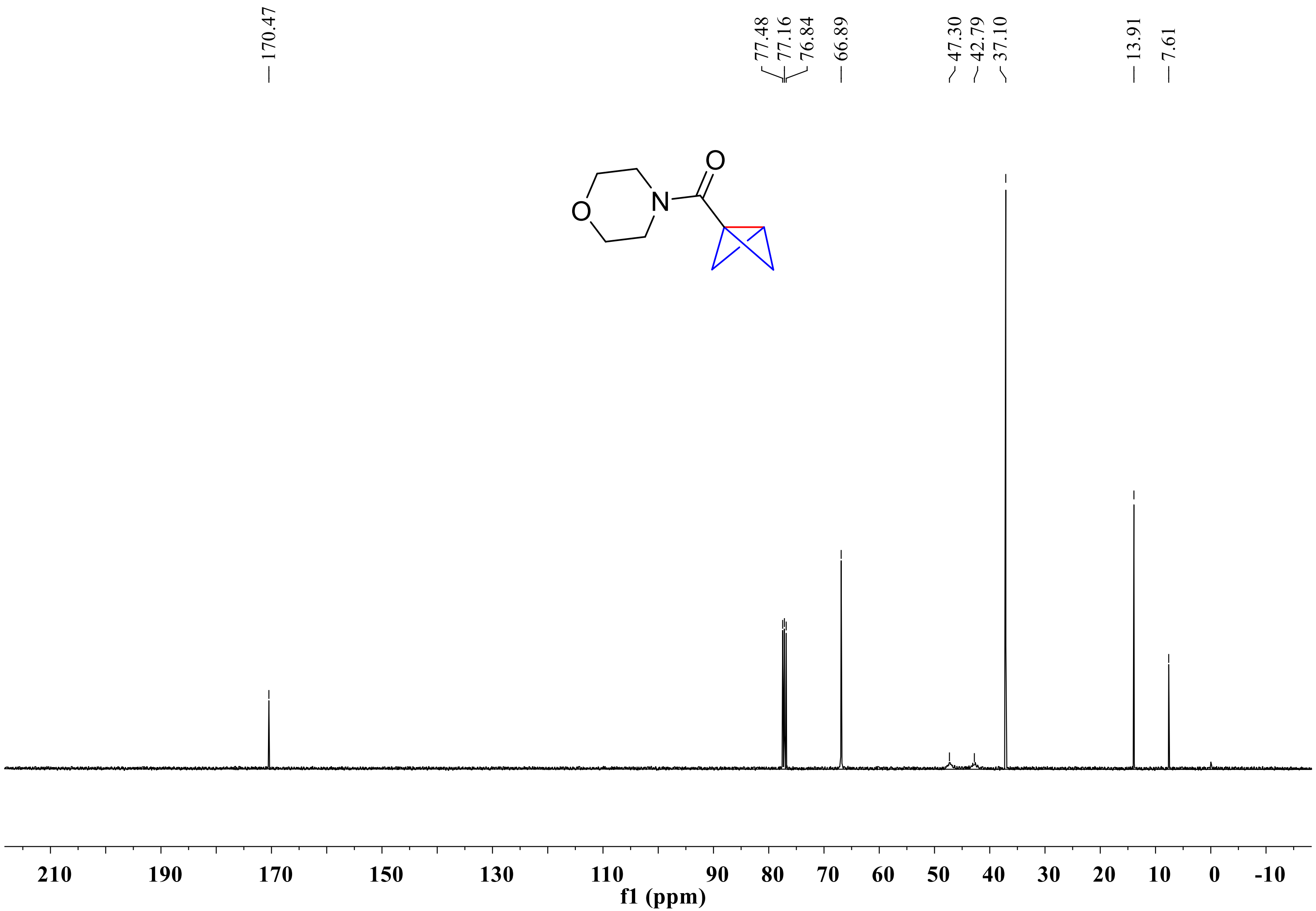

Supplementary Fig. 61 ^{13}C NMR (101 MHz, $CDCl_3$) of Morpholine amide **1t**

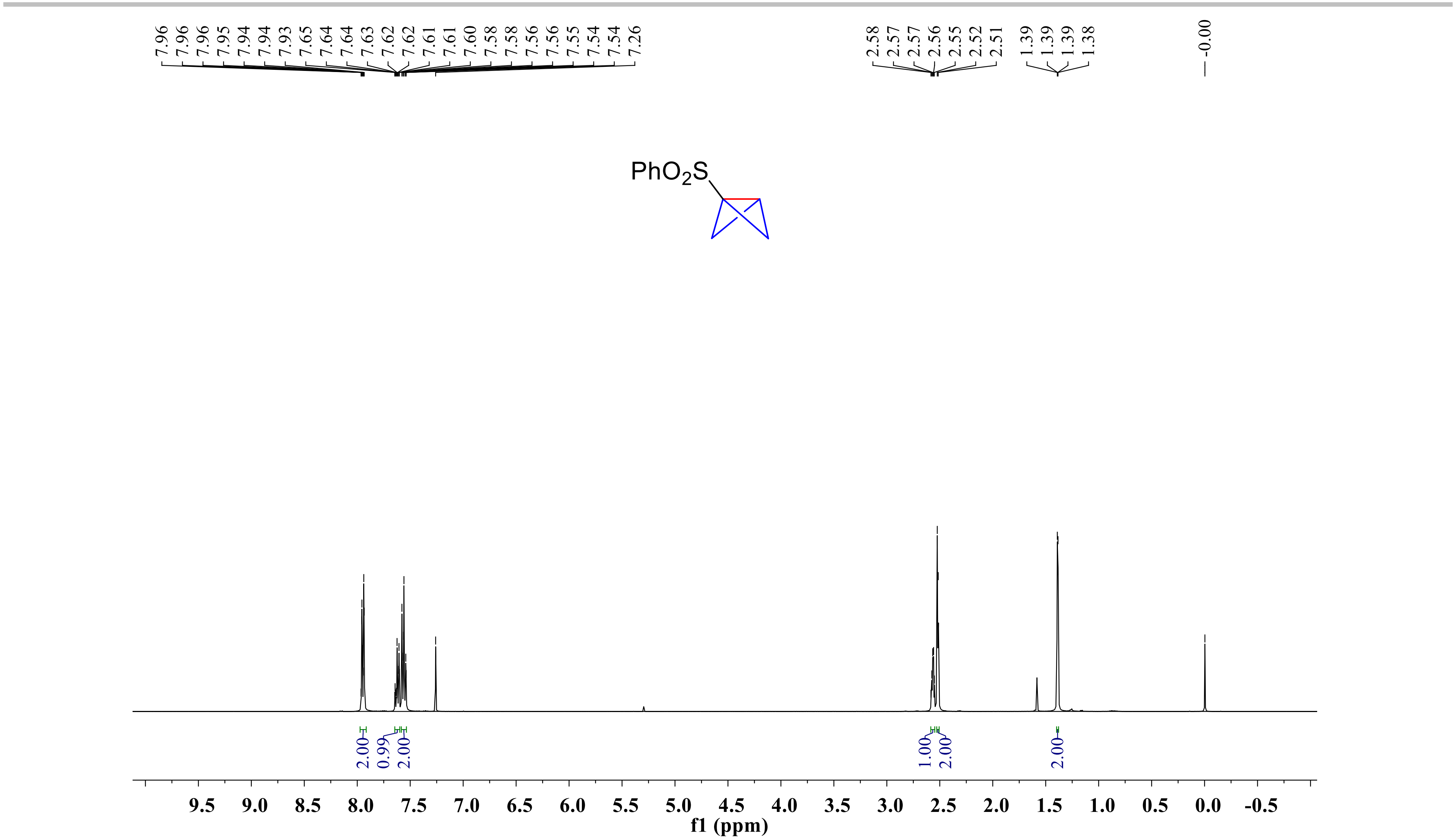

Supplementary Fig. 62 ^{1}H NMR (400 MHz, $CDCl_3$) of **1u**

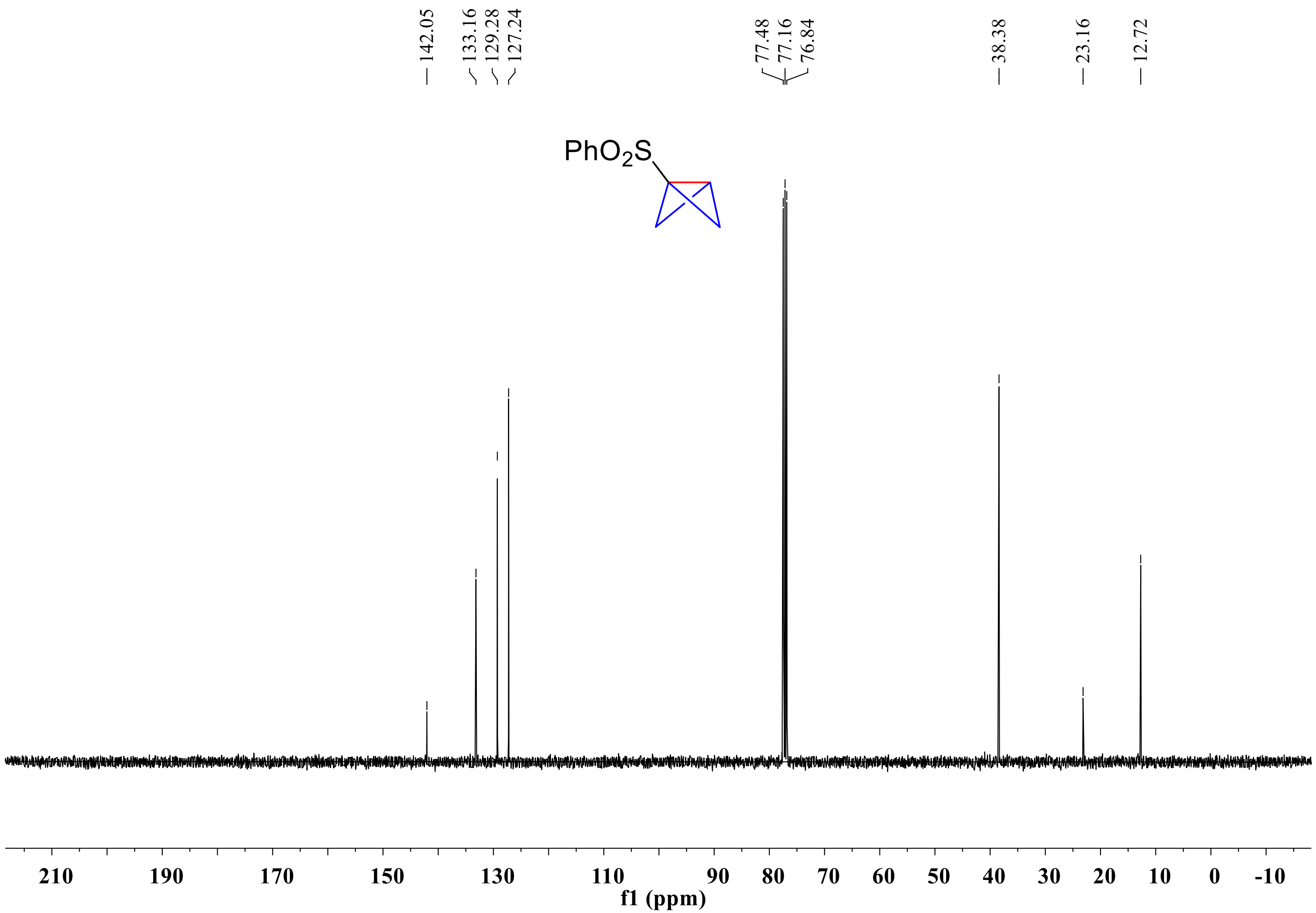

Supplementary Fig. 63 ^{13}C NMR (101 MHz, $CDCl_3$) of **1u**

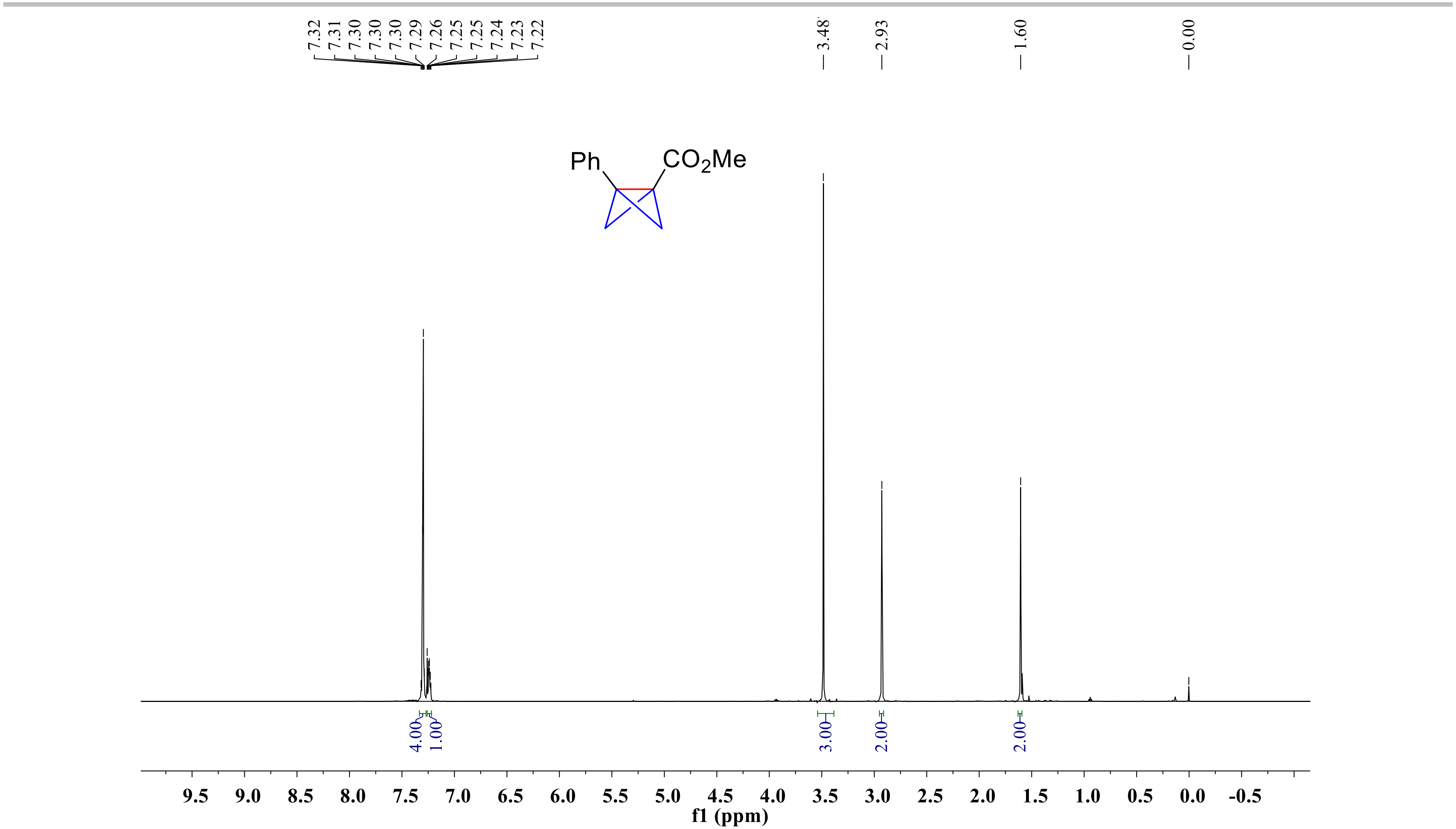

Supplementary Fig. 64 ^{1}H NMR (600 MHz, $CDCl_3$) of **1a’**

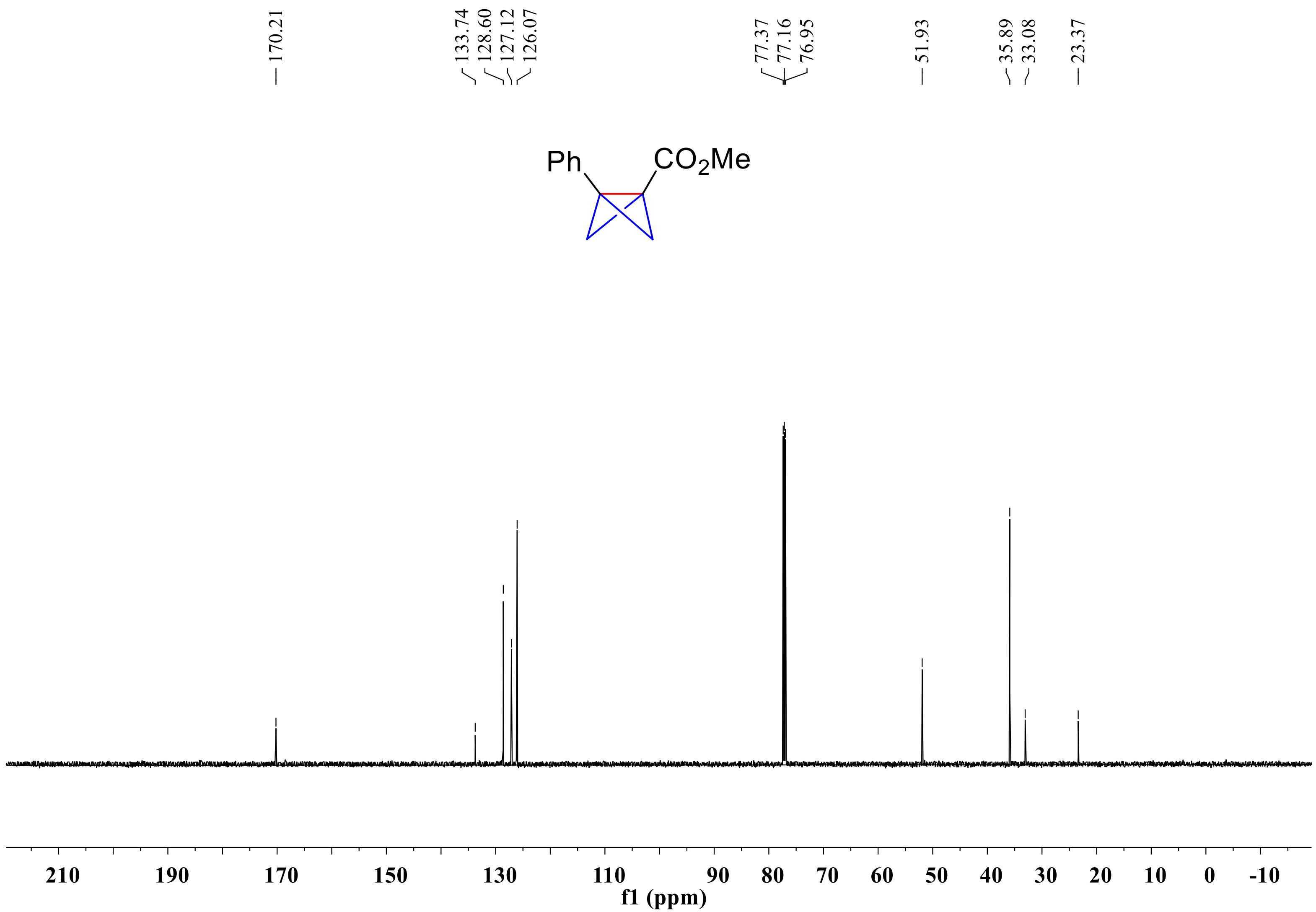

Supplementary Fig. 65 ^{13}C NMR (151 MHz, $CDCl_3$) of **1a'**

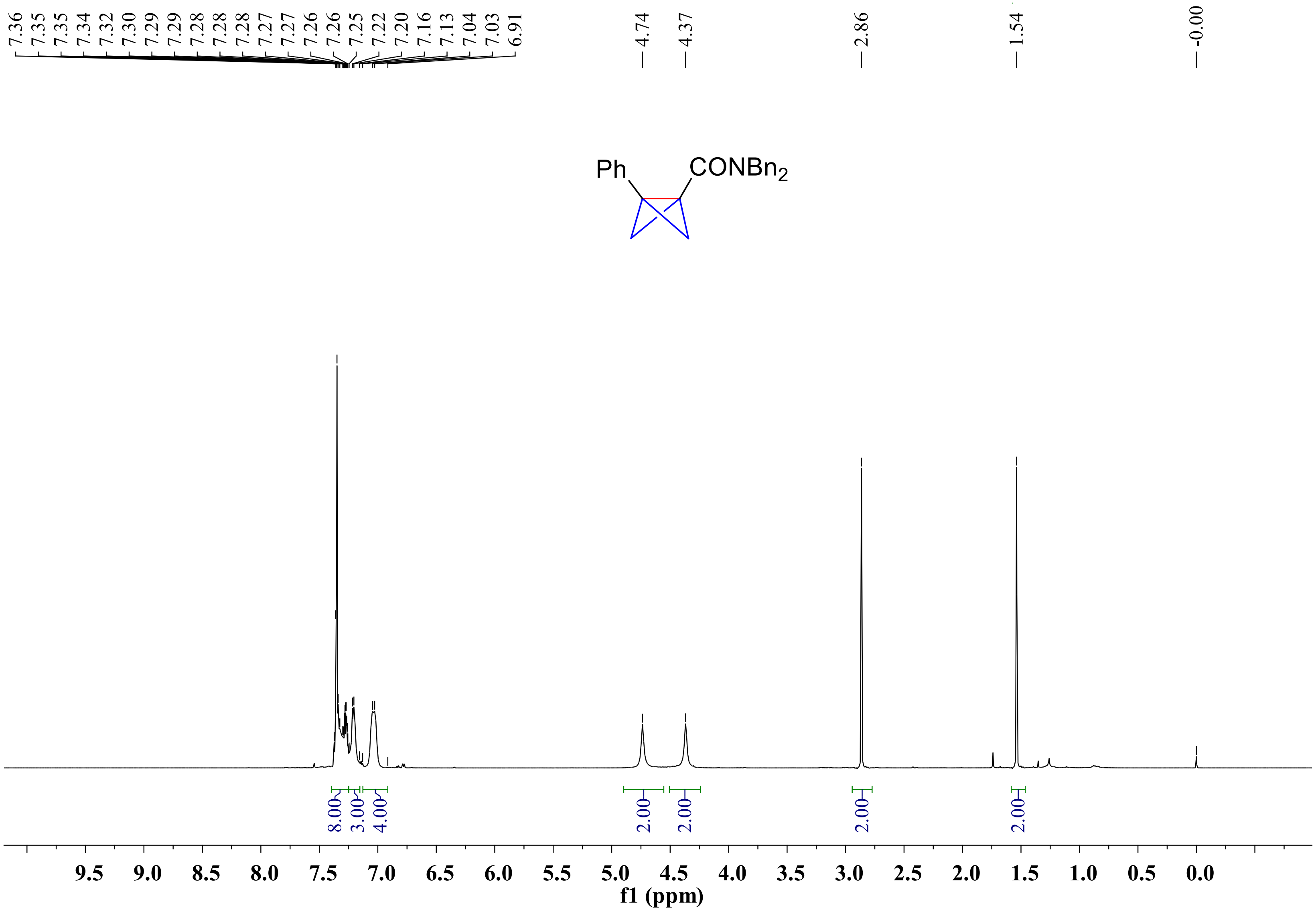

Supplementary Fig. 66 ^{1}H NMR (600 MHz, $CDCl_3$) of β-phenyl amide BCBs

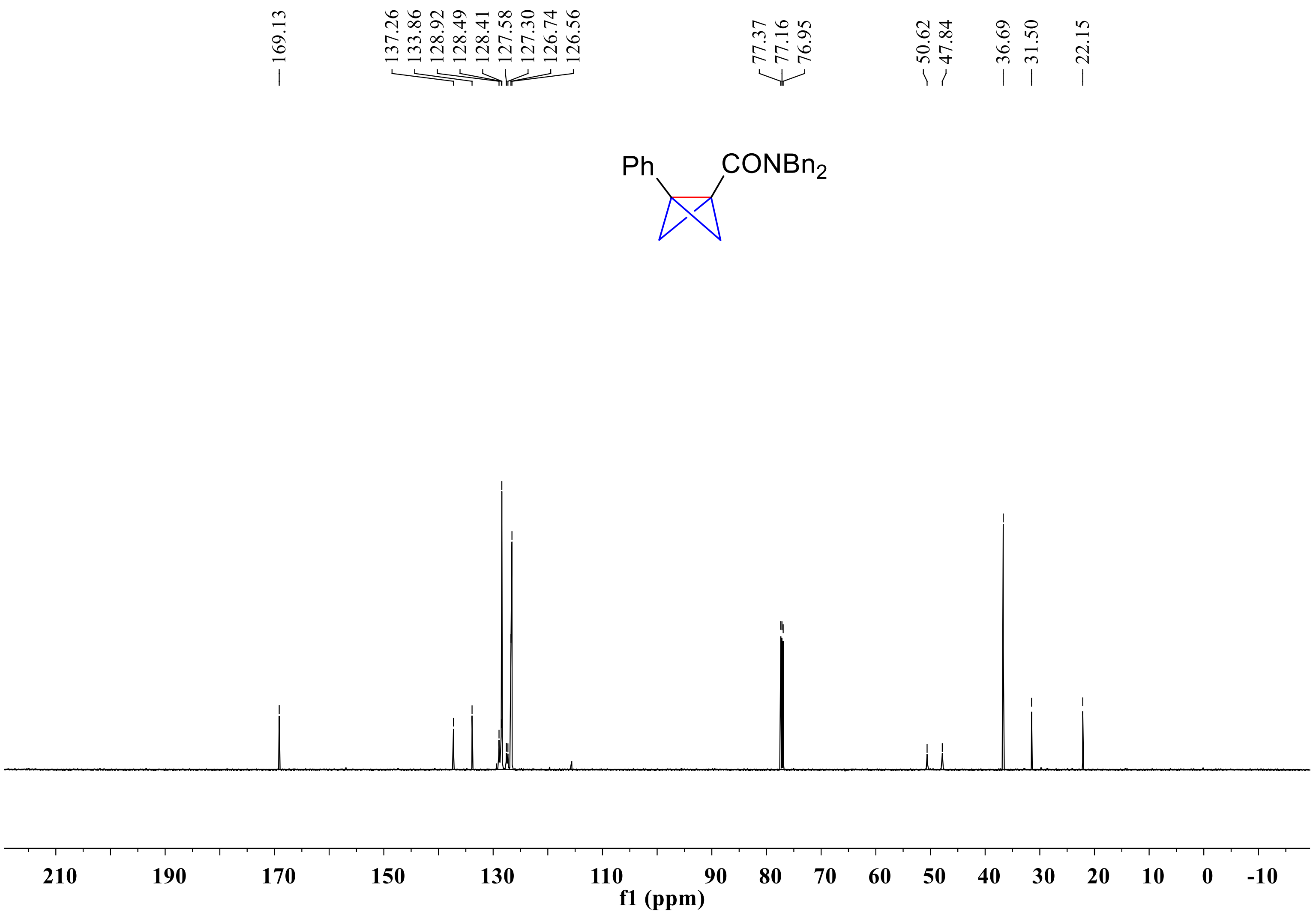

Supplementary Fig. 67 ^{13}C NMR (151 MHz, $CDCl_3$) of β -phenyl amide BCBs

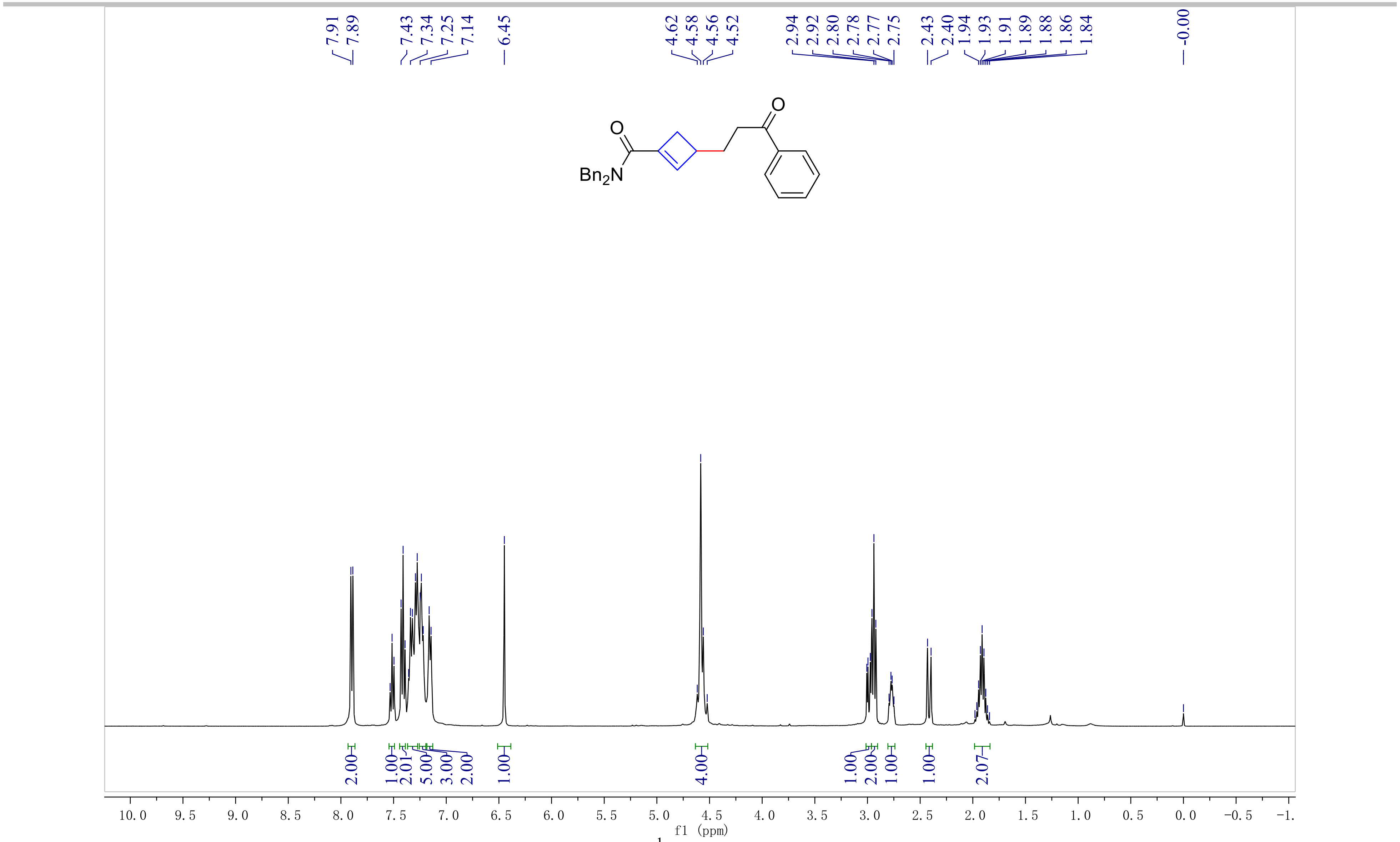

Supplementary Fig. 68 ^{1}H NMR (400 MHz, $CDCl_3$) of **3a**

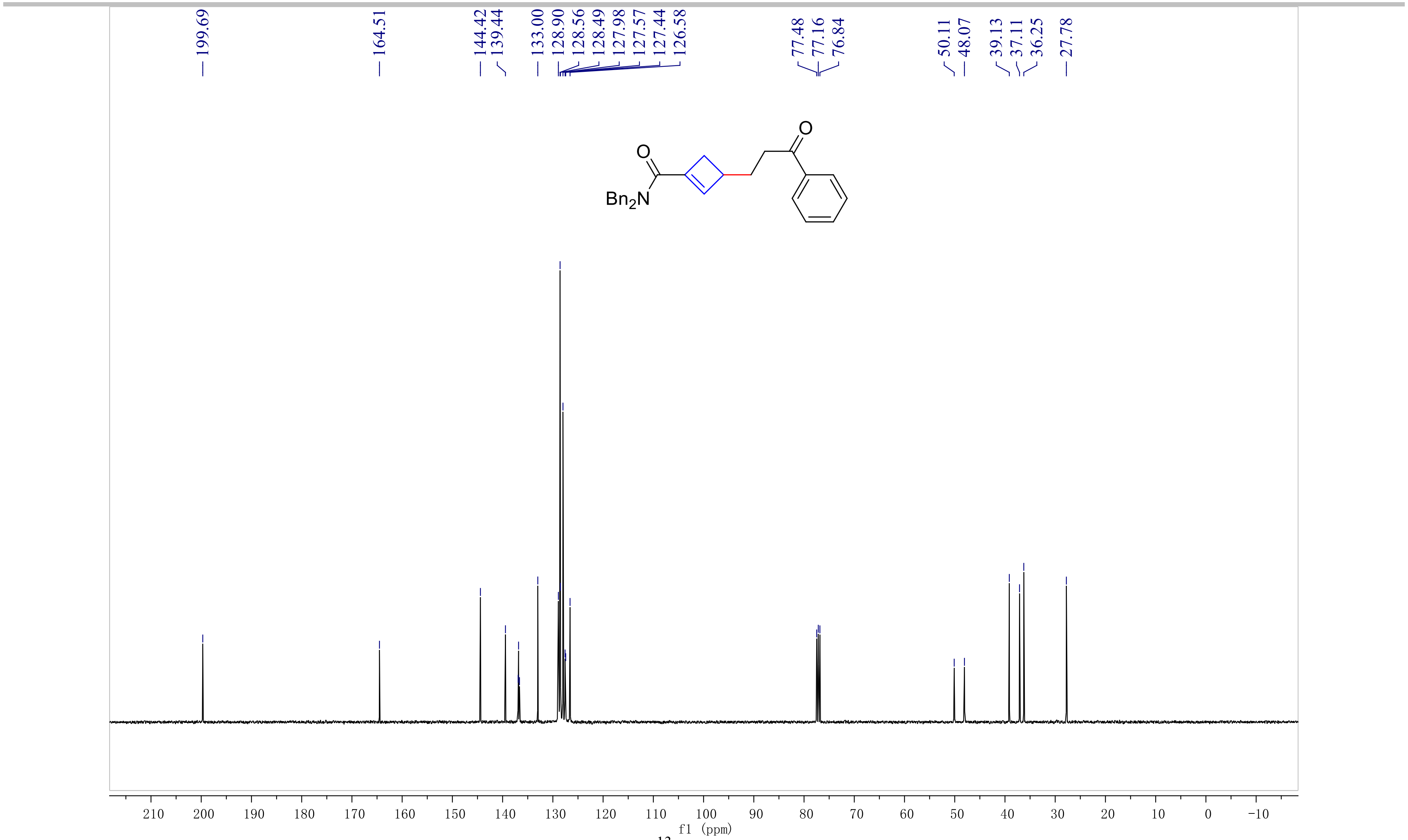

Supplementary Fig. 69 ^{13}C NMR (101 MHz, $CDCl_3$) of **3a**

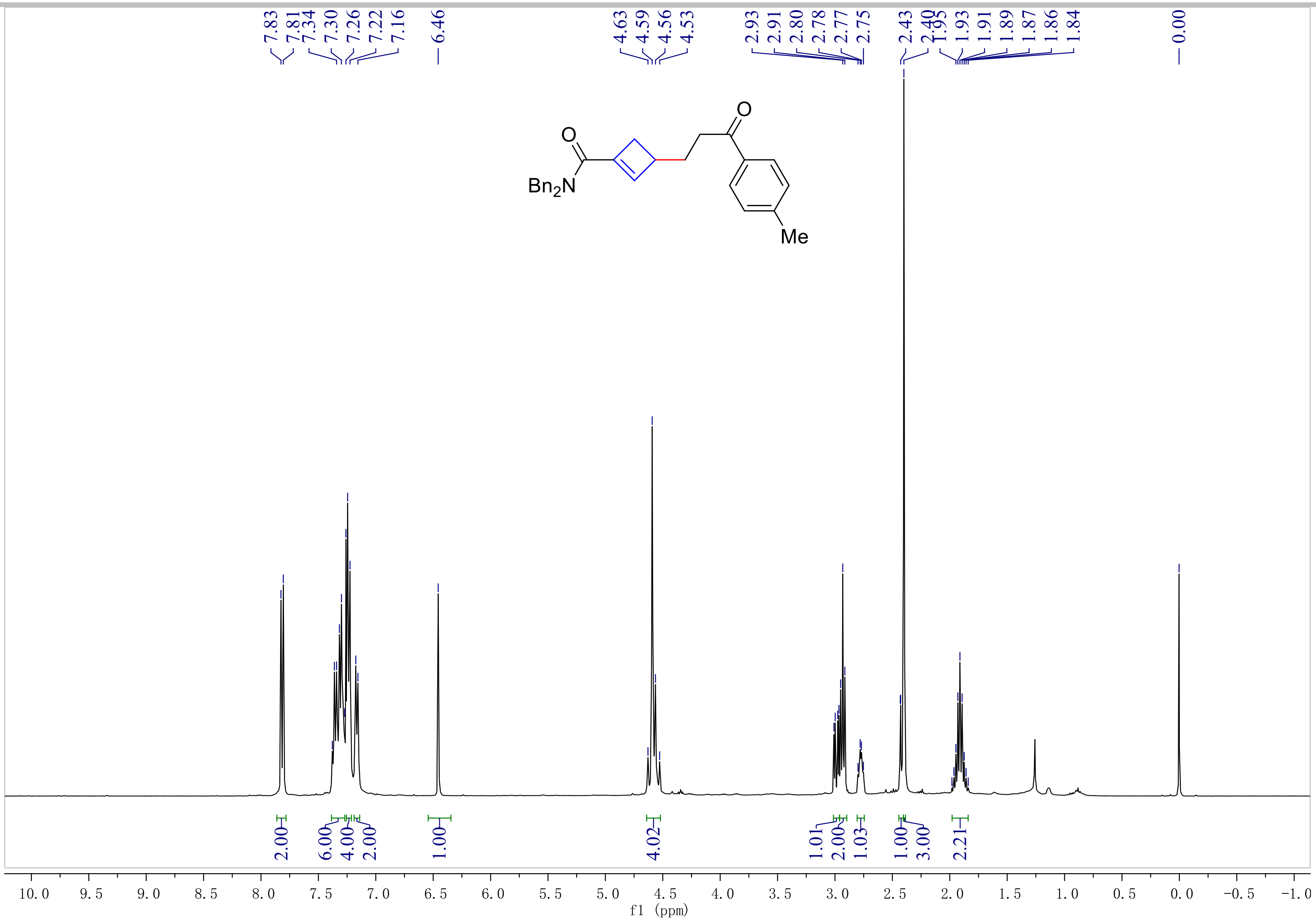

Supplementary Fig. 70 ^{1}H NMR (400 MHz, $CDCl_3$) of **3b**

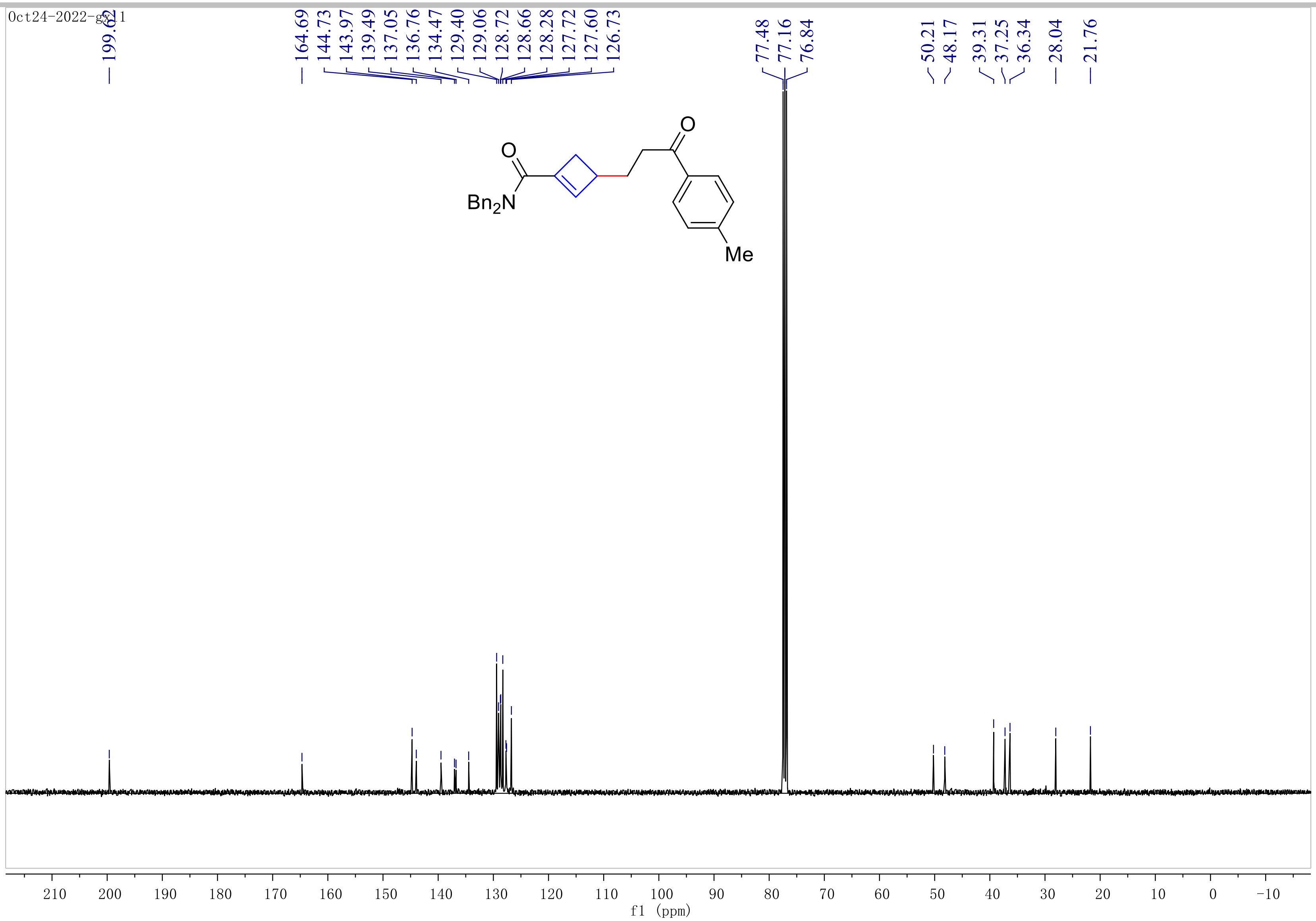

Supplementary Fig. 71 ^{13}C NMR (151 MHz, $CDCl_3$) of **3b**

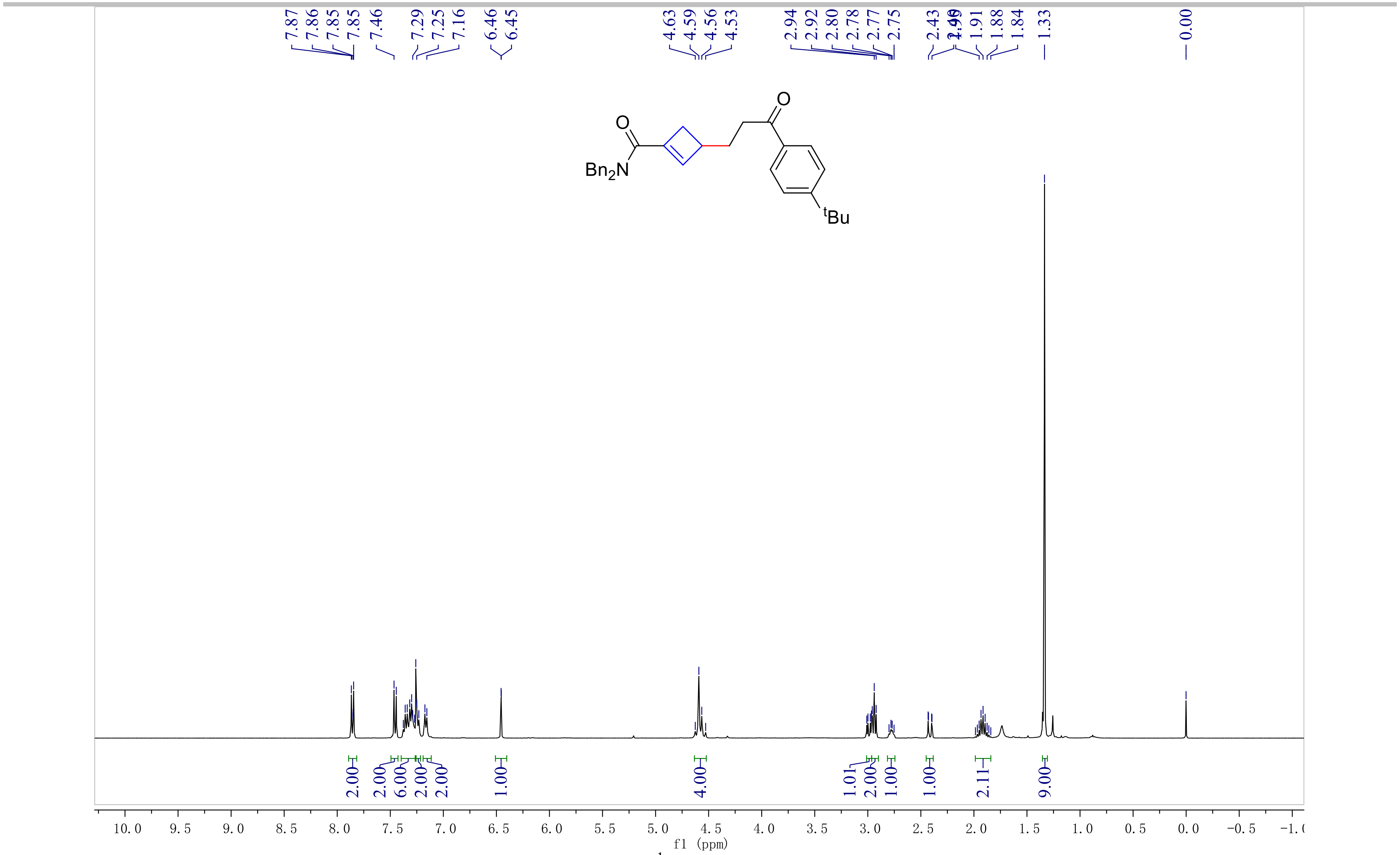

Supplementary Fig. 72 ^{1}H NMR (400 MHz, $CDCl_3$) of **3c**

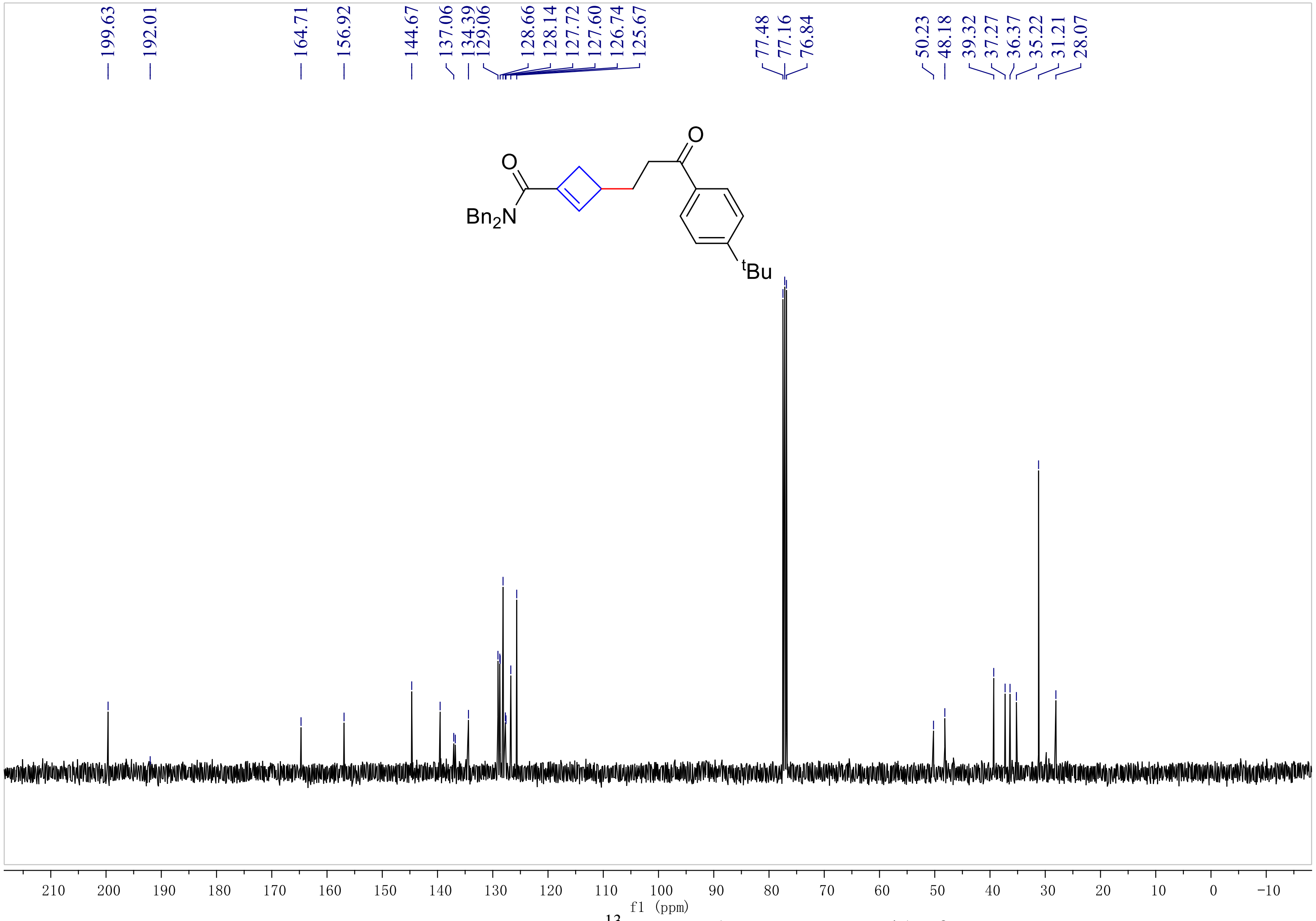

Supplementary Fig. 73 ^{13}C NMR (101 MHz, $CDCl_3$) of **3c**

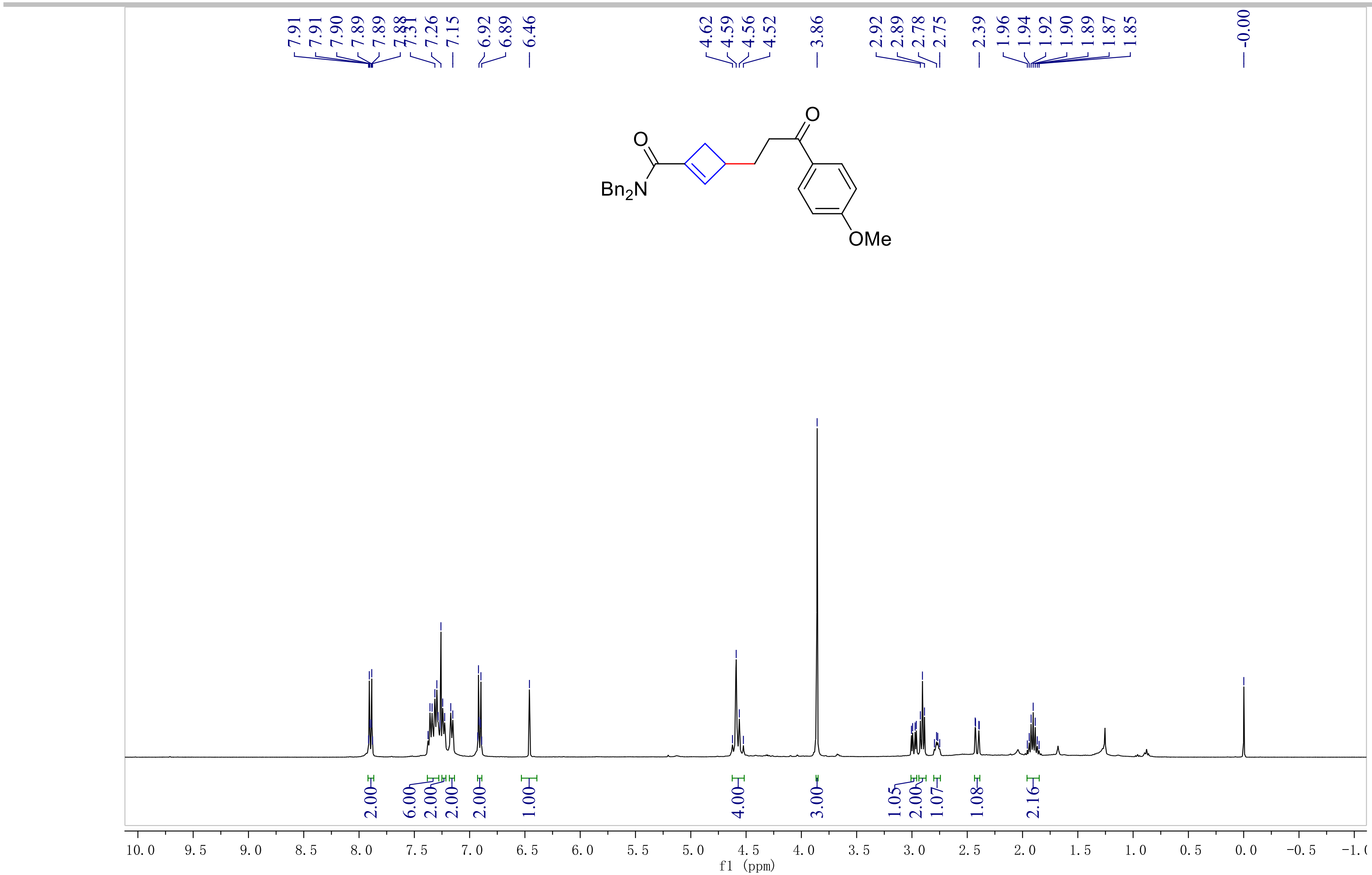

Supplementary Fig. 74 ^{1}H NMR (400 MHz, $CDCl_3$) of **3d**

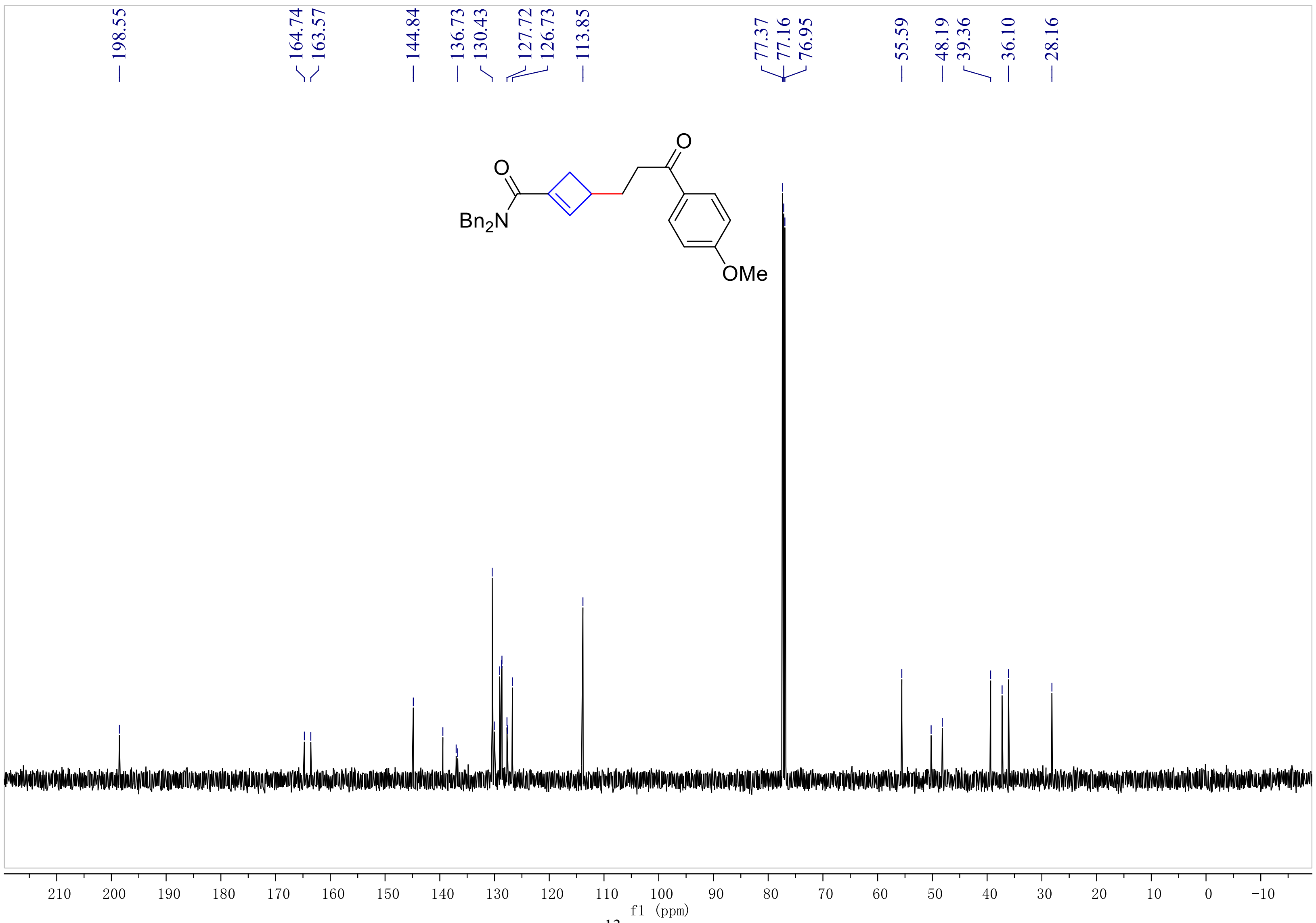

Supplementary Fig. 75 ^{13}C NMR (151 MHz, $CDCl_3$) of **3d**

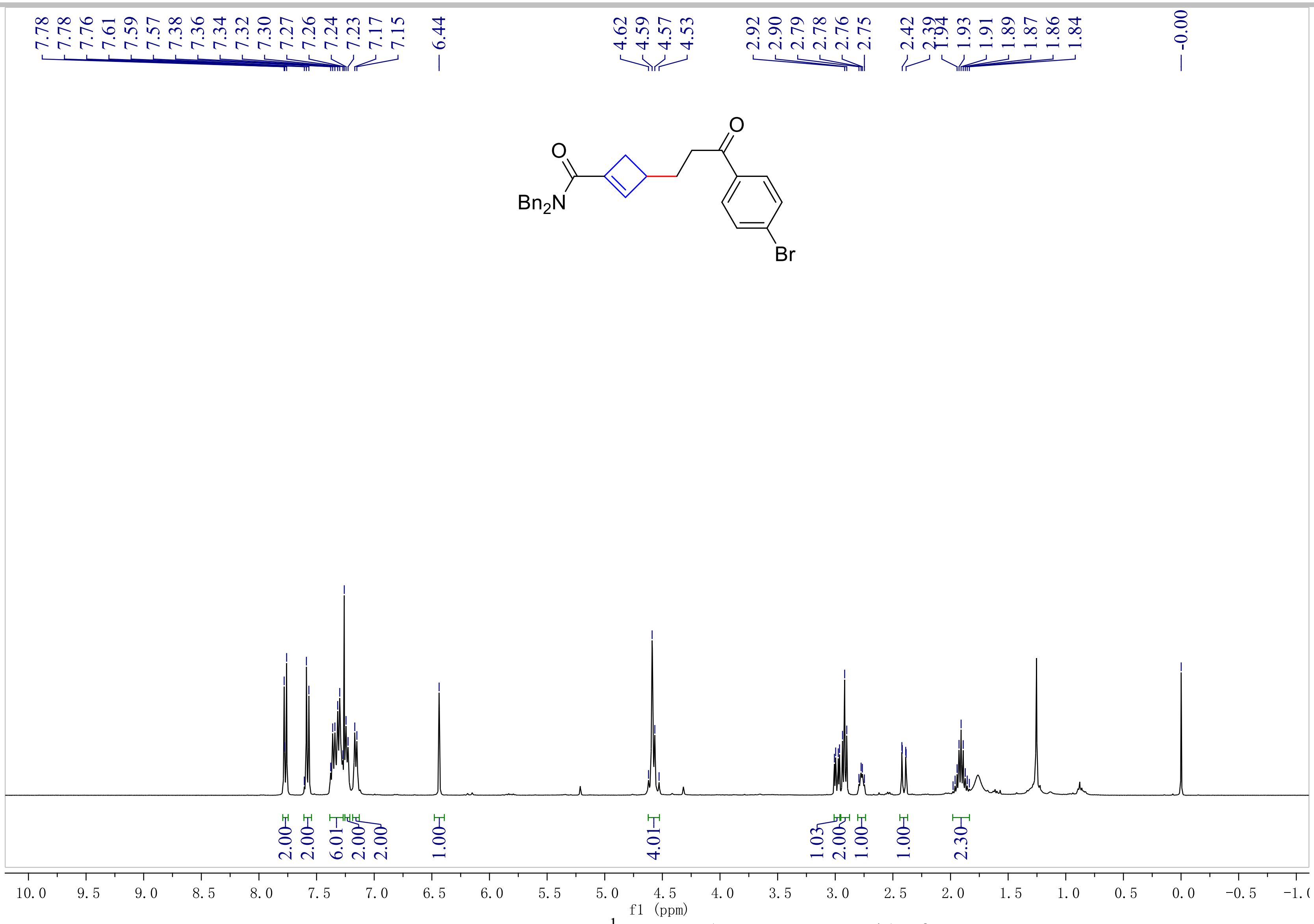

Supplementary Fig. 76 ^{1}H NMR (400 MHz, $CDCl_3$) of **3e**

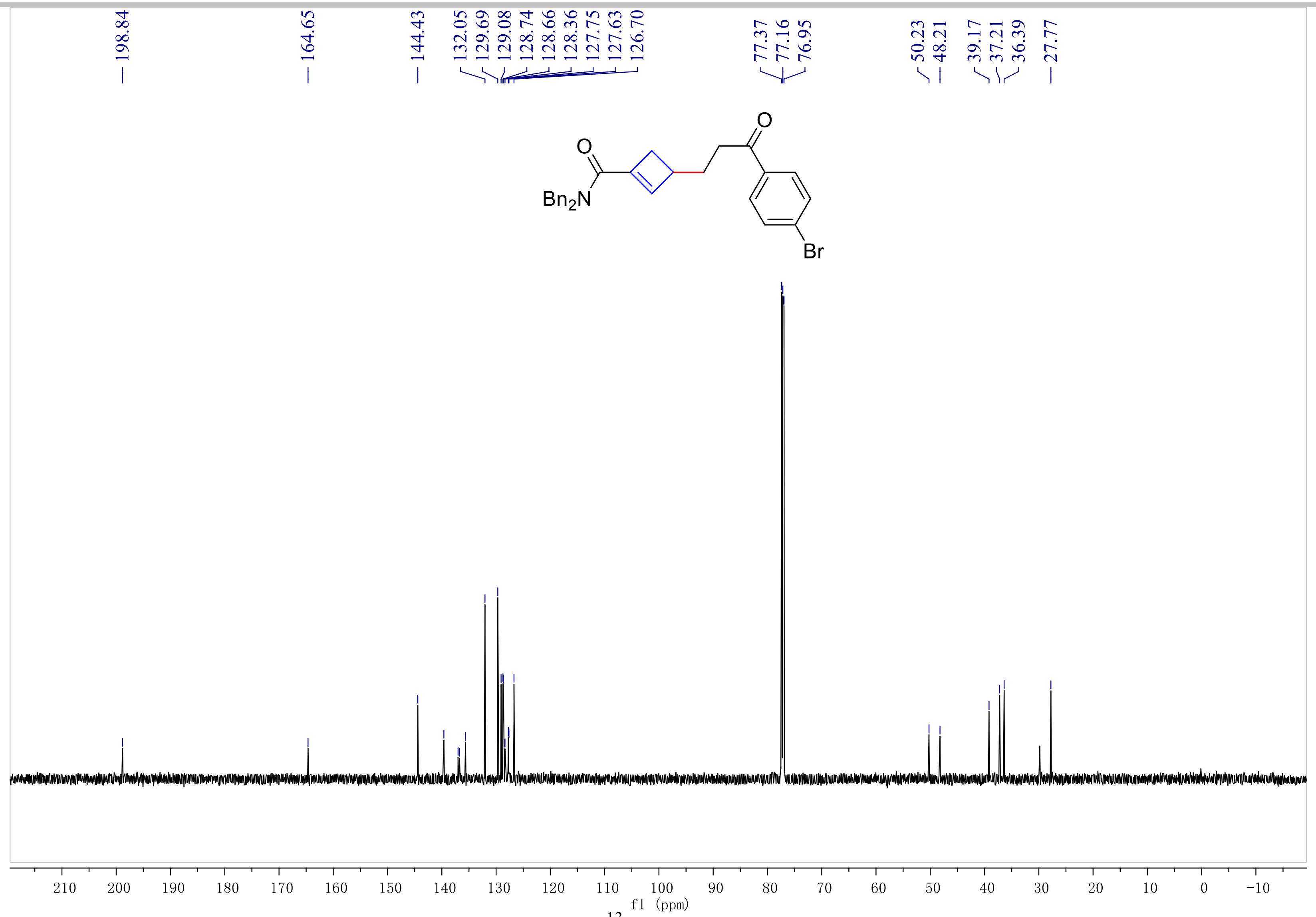

Supplementary Fig. 77 ^{13}C NMR (151 MHz, $CDCl_3$) of **3e**

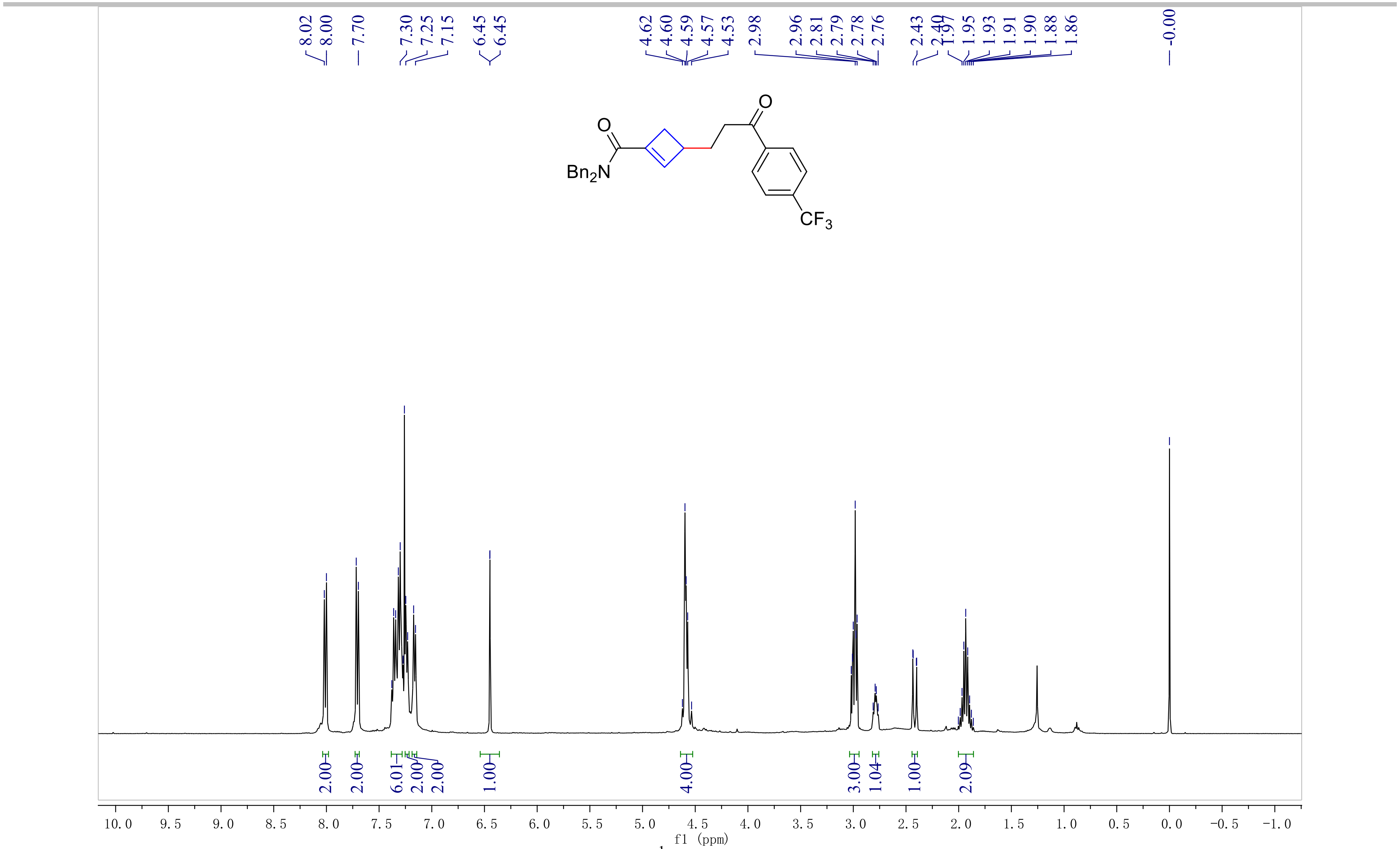

Supplementary Fig. 78 ^{1}H NMR (400 MHz, $CDCl_3$) of **3f**

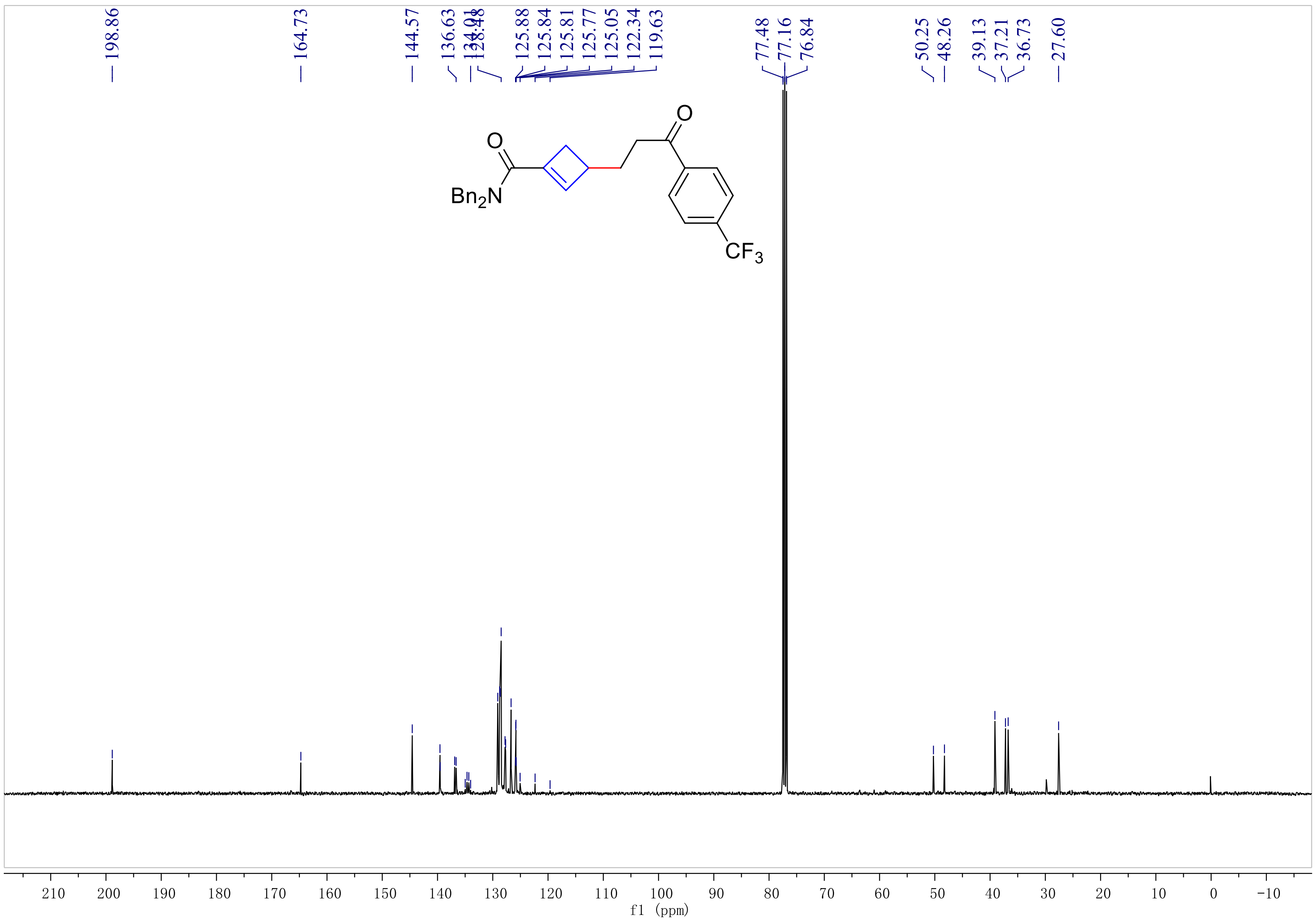

Supplementary Fig. 79 ^{13}C NMR (101 MHz, $CDCl_3$) of **3f**

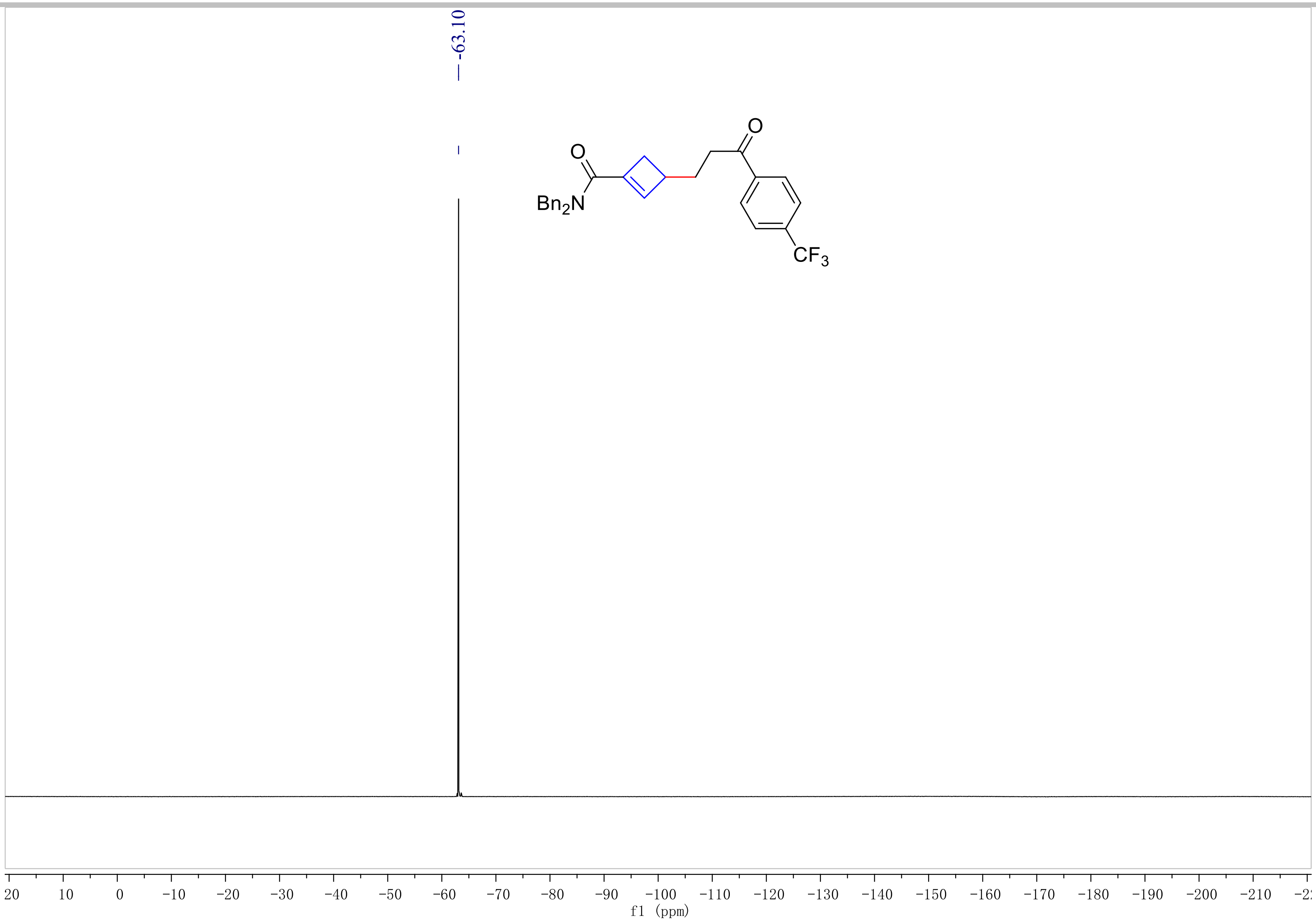

Supplementary Fig. 80 ^{19}F NMR (377 MHz, $CDCl_3$) of **3f**

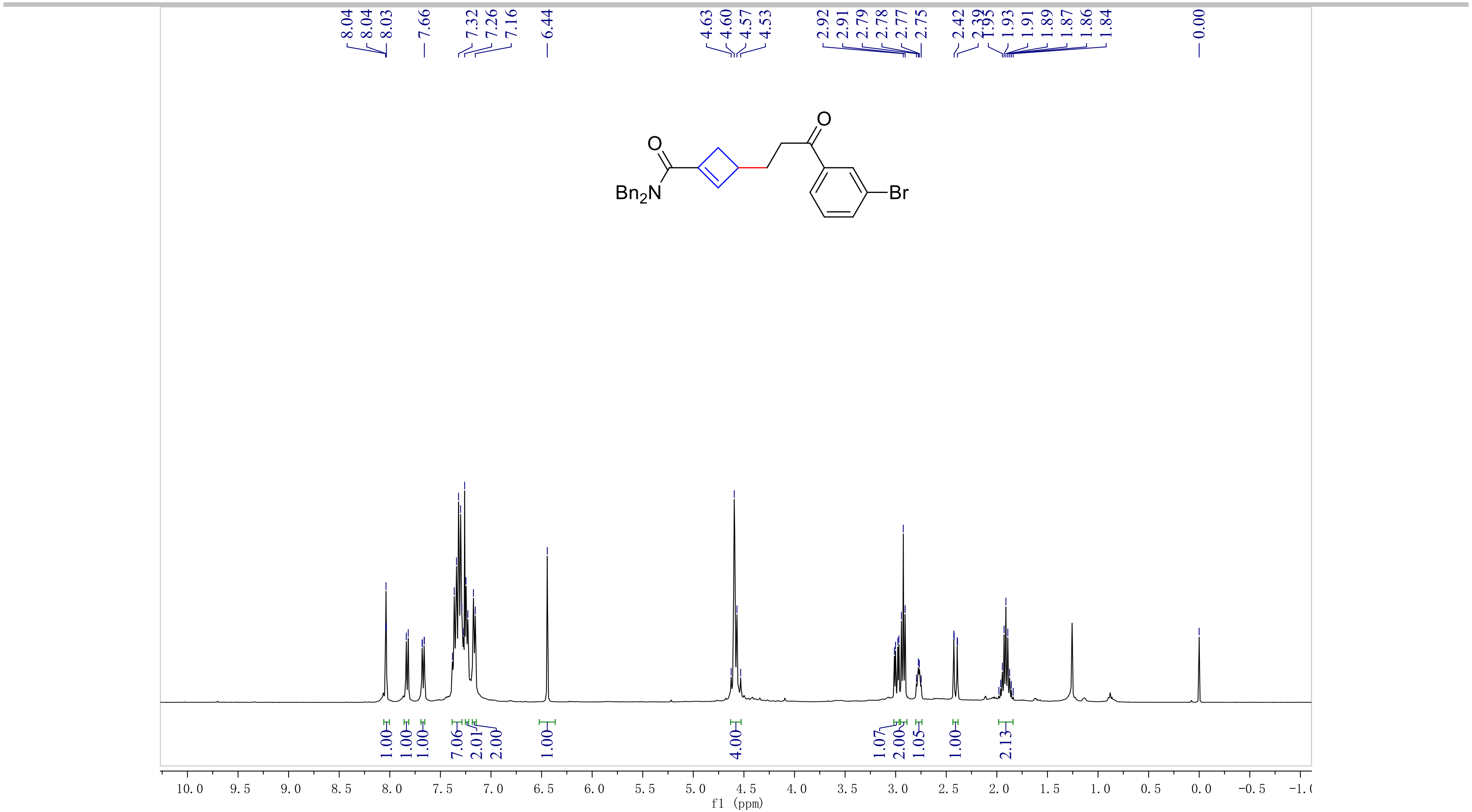

Supplementary Fig. 81 ^{1}H NMR (400 MHz, $CDCl_3$) of **3g**

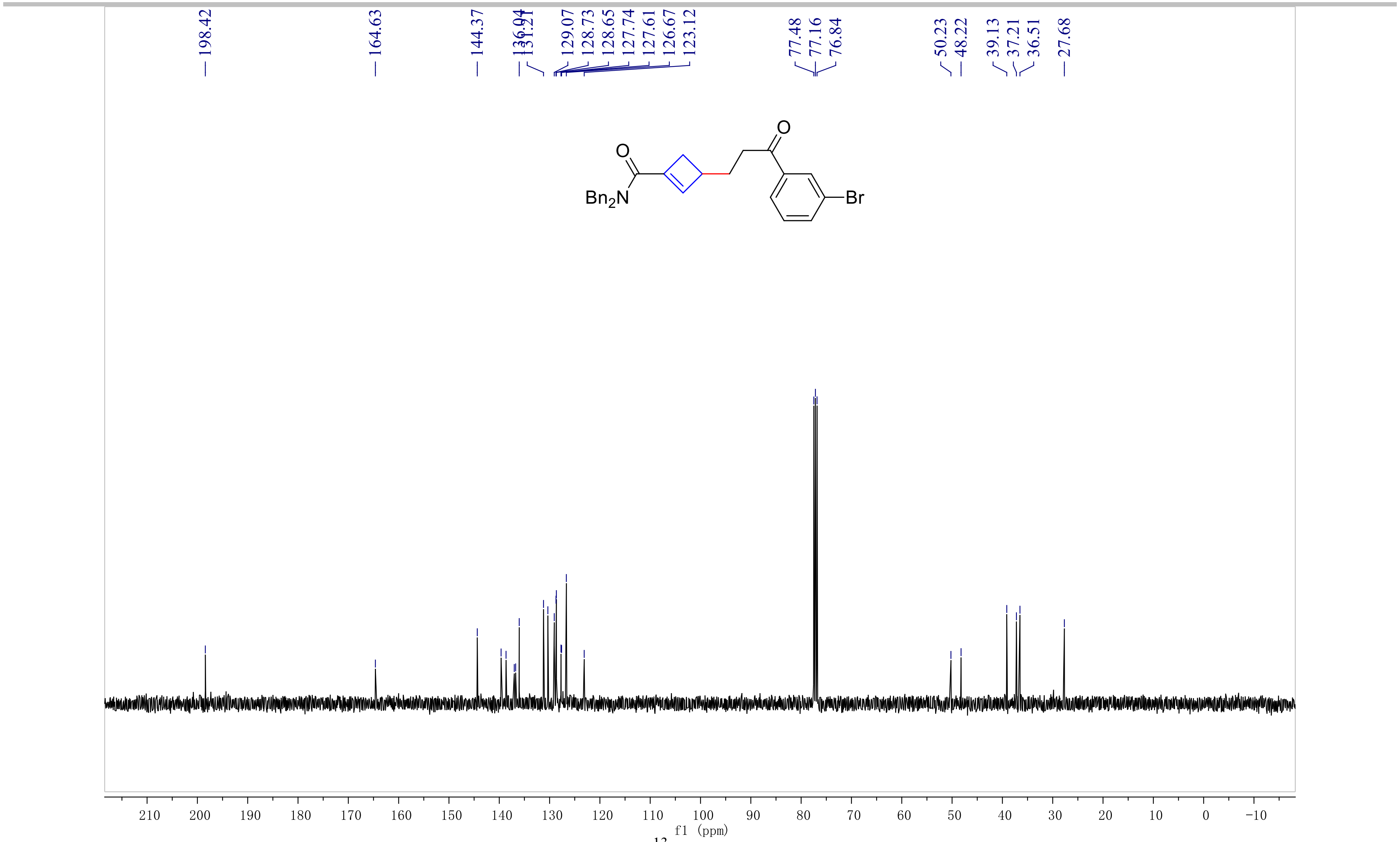

Supplementary Fig. 82 ^{13}C NMR (101 MHz, $CDCl_3$) of **3g**

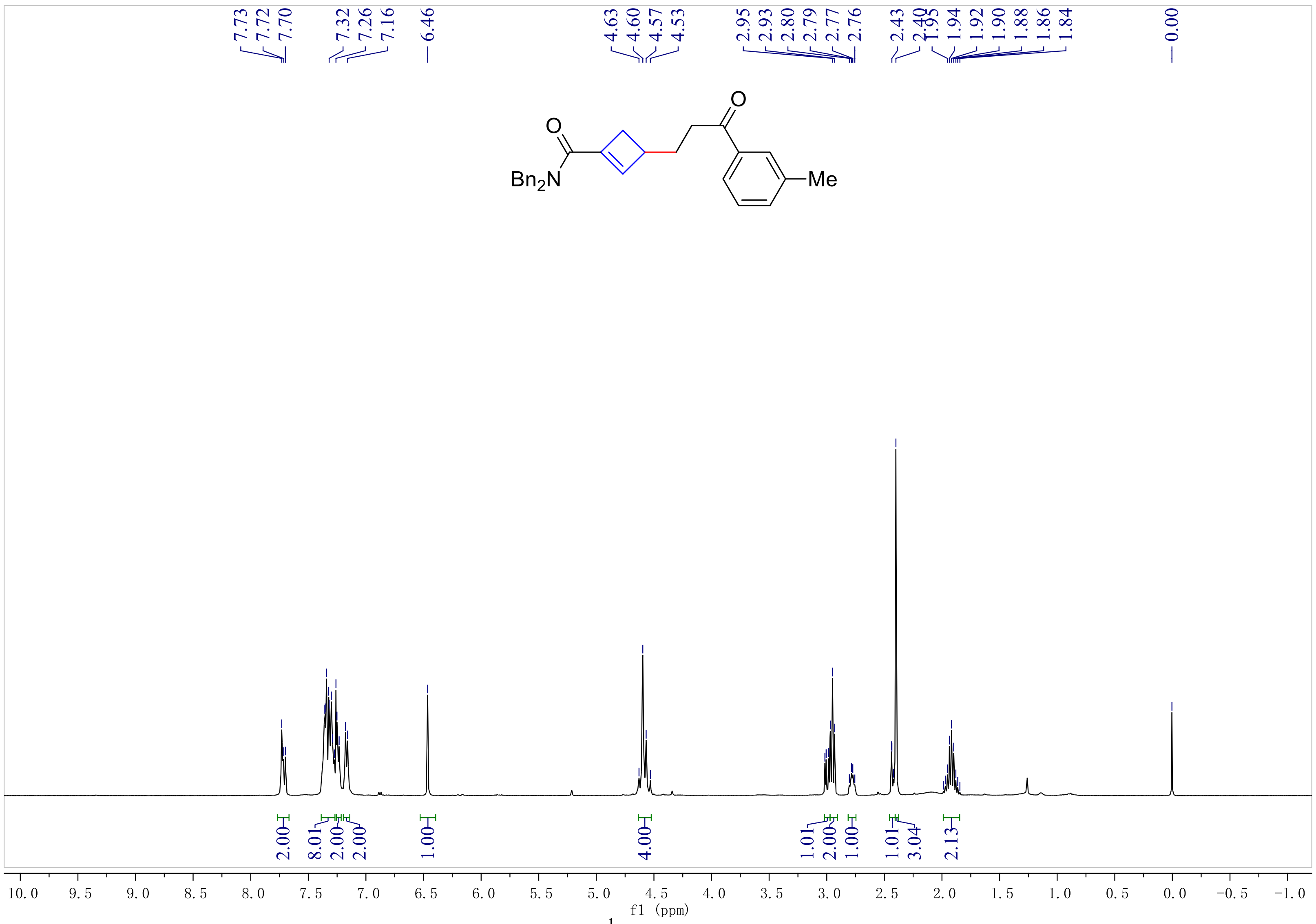

Supplementary Fig. 83 ^{1}H NMR (400 MHz, $CDCl_3$) of **3h**

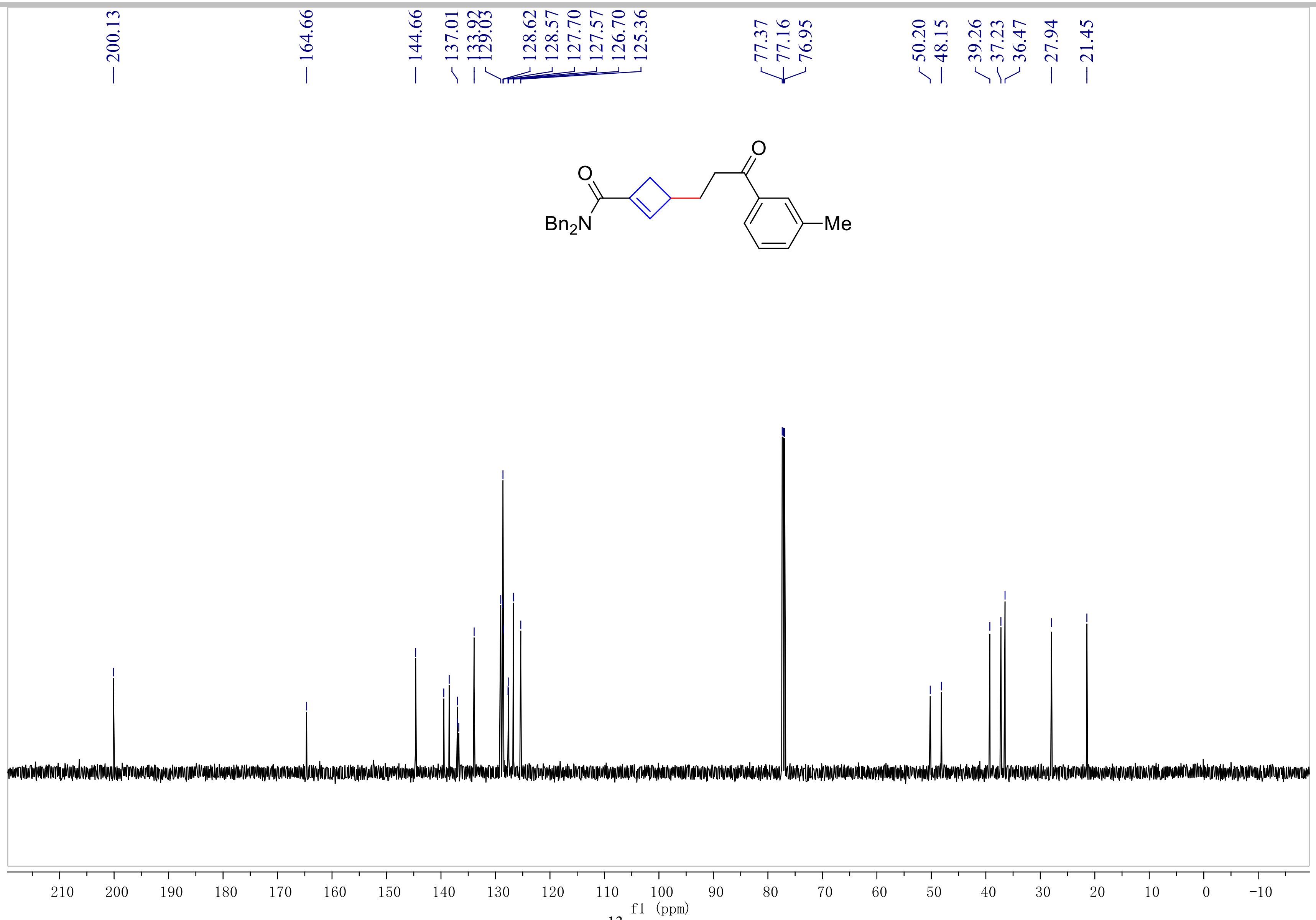

Supplementary Fig. 84 ^{13}C NMR (151 MHz, $CDCl_3$) of **3h**

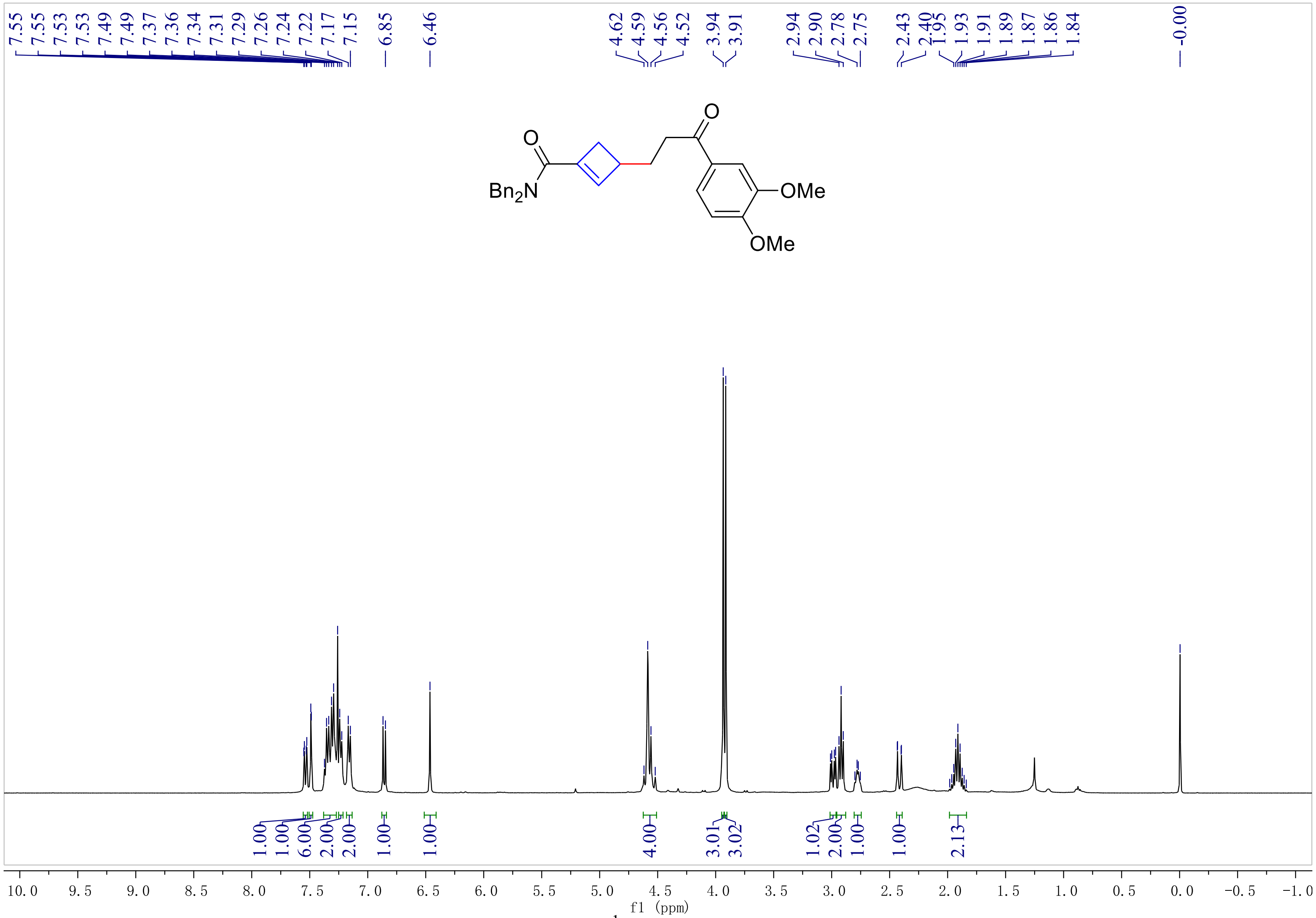

Supplementary Fig. 85 ^{1}H NMR (400 MHz, $CDCl_3$) of **3i**

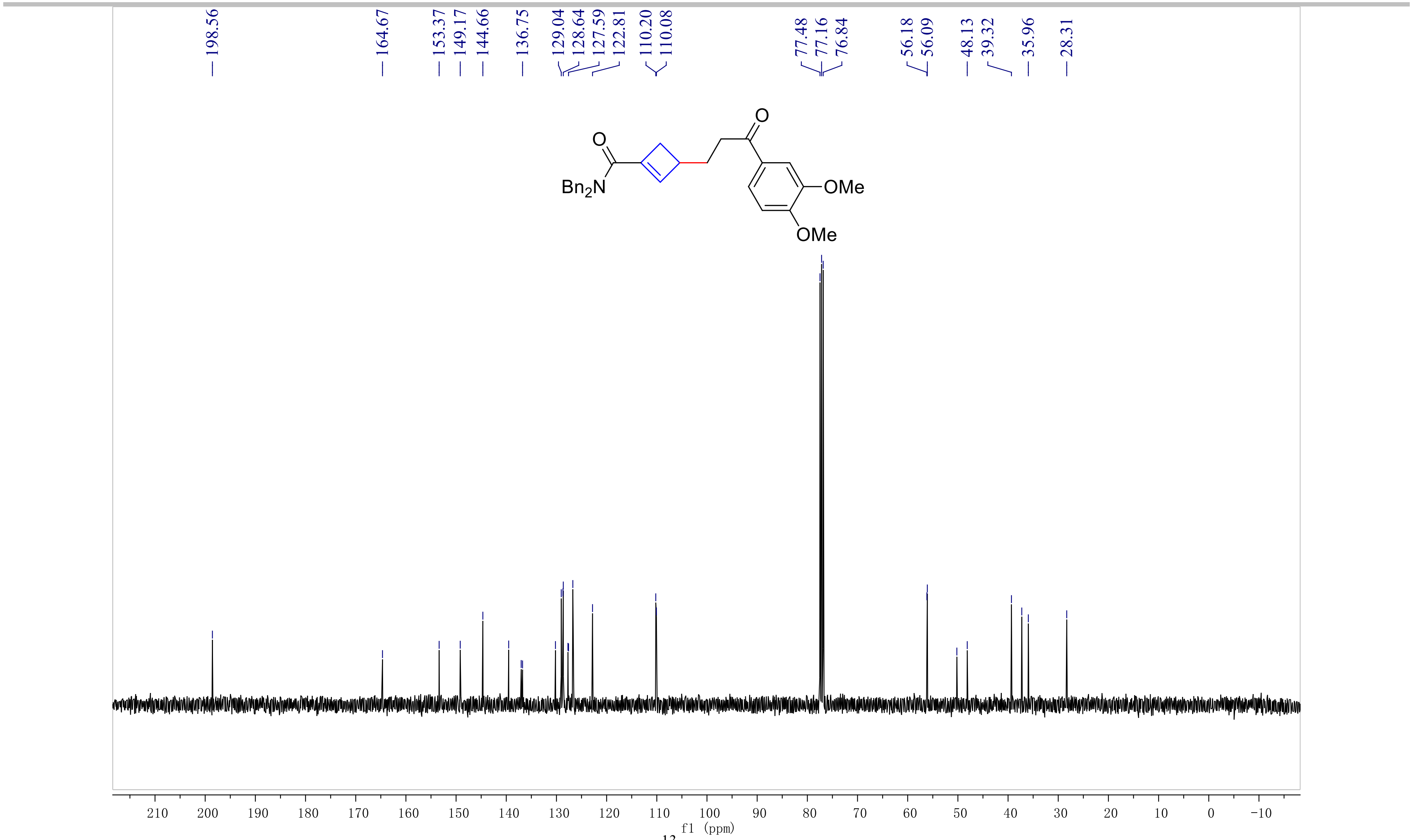

Supplementary Fig. 86 ^{13}C NMR (101 MHz, $CDCl_3$) of **3i**

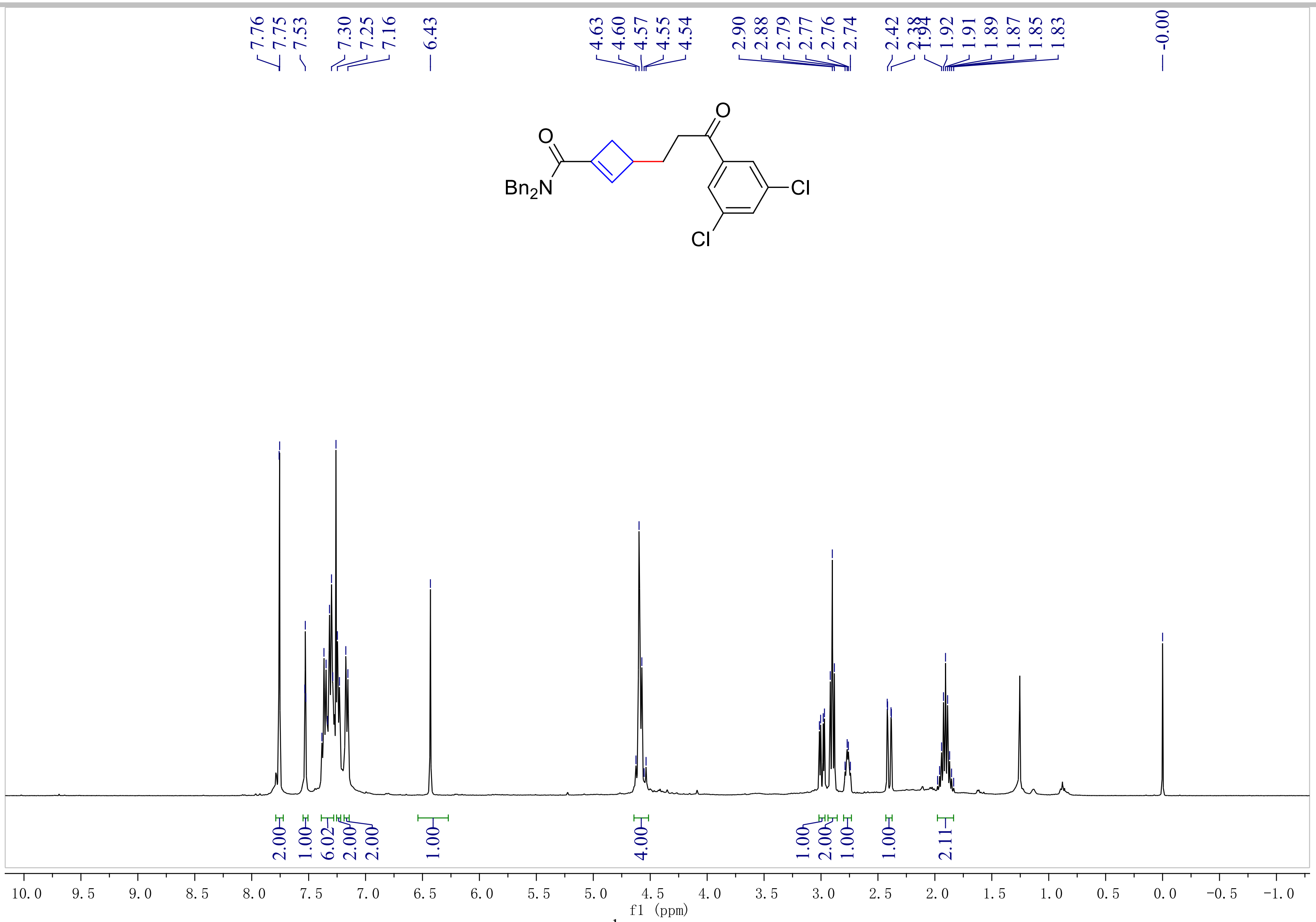

Supplementary Fig. 87 ^{1}H NMR (400 MHz, $CDCl_3$) of **3j**

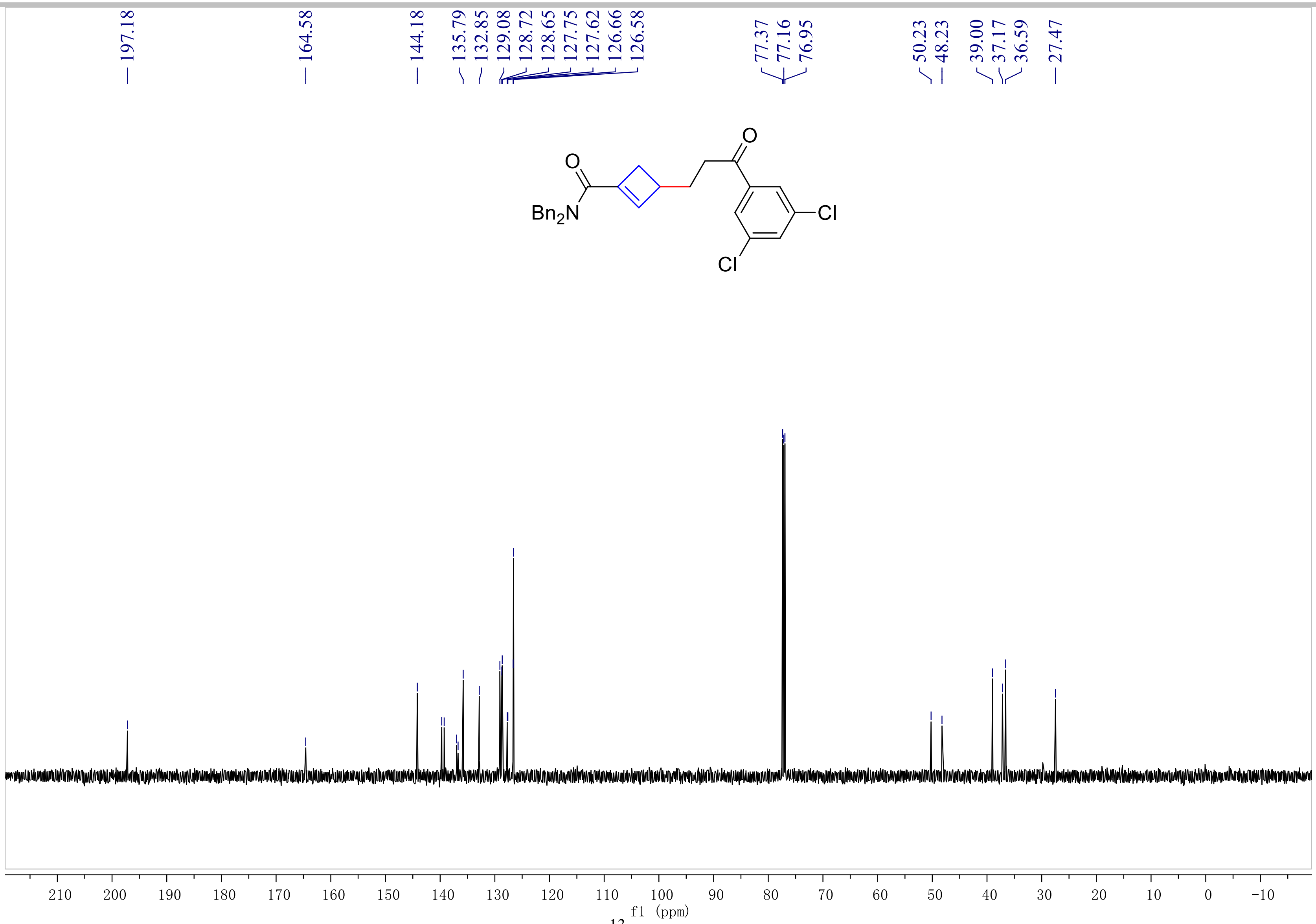

Supplementary Fig. 88 ^{13}C NMR (151 MHz, $CDCl_3$) of **3j**

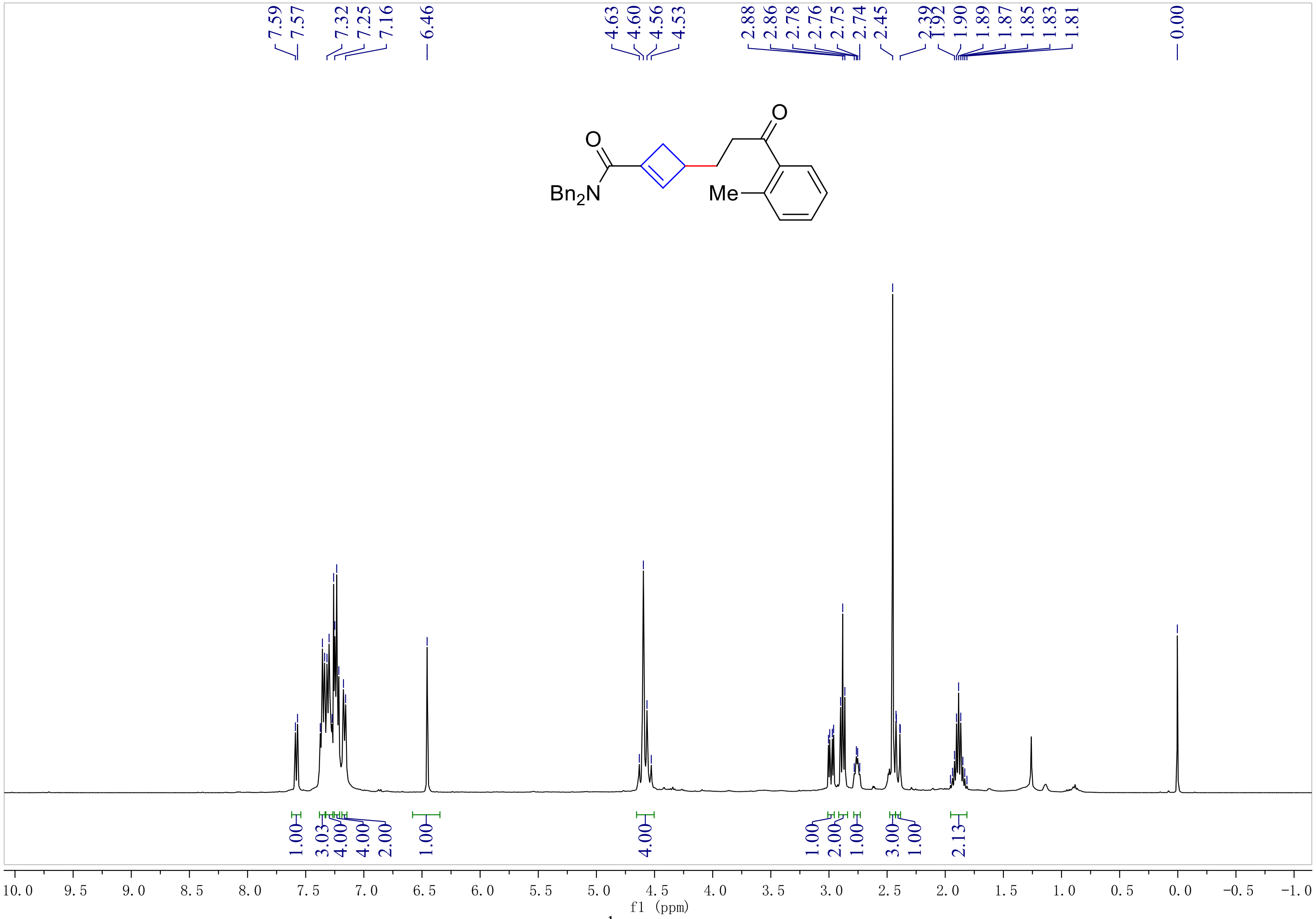

Supplementary Fig. 89 ^{1}H NMR (400 MHz, $CDCl_3$) of **3k**

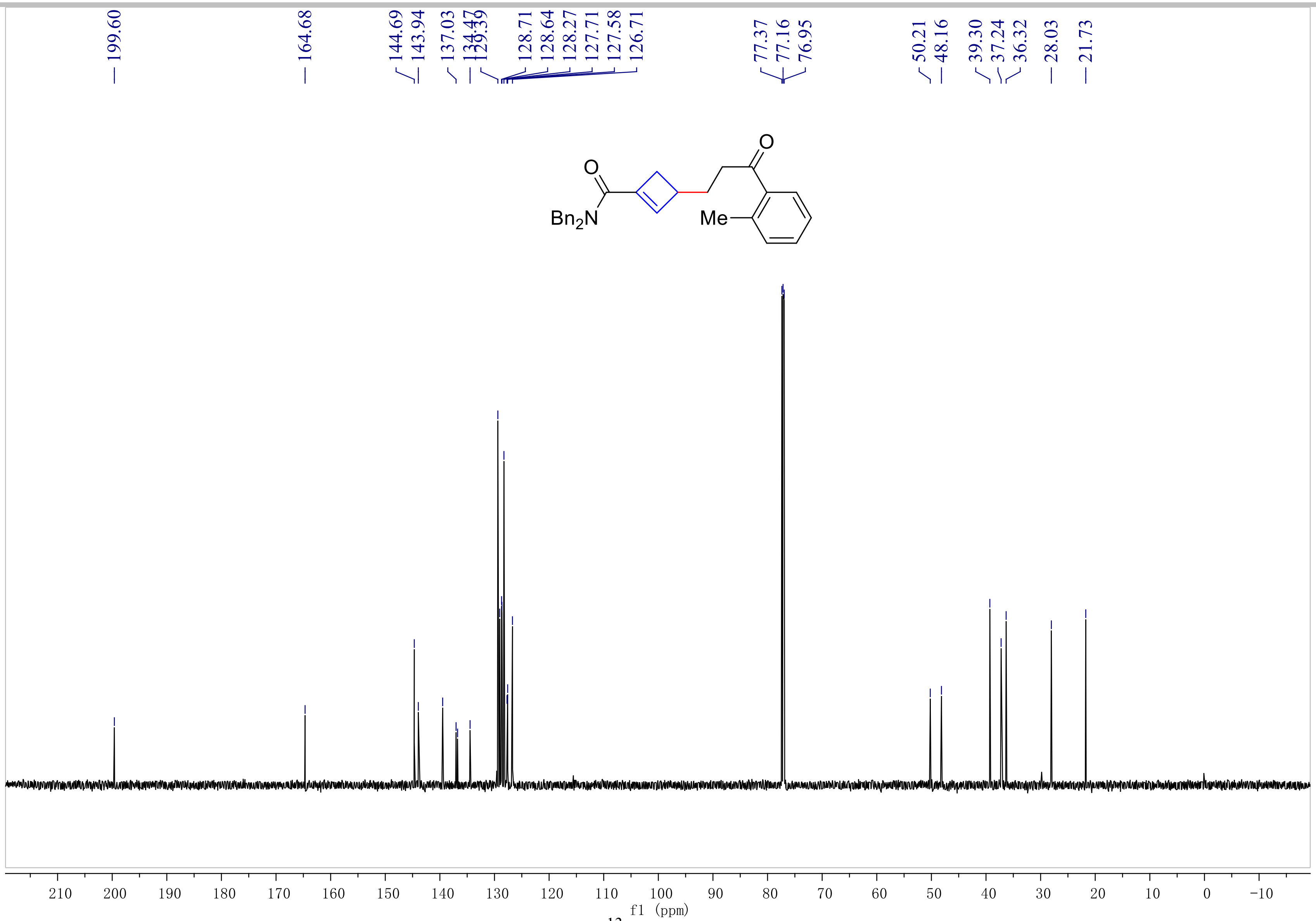

Supplementary Fig. 90 ^{13}C NMR (151 MHz, $CDCl_3$) of **3k**

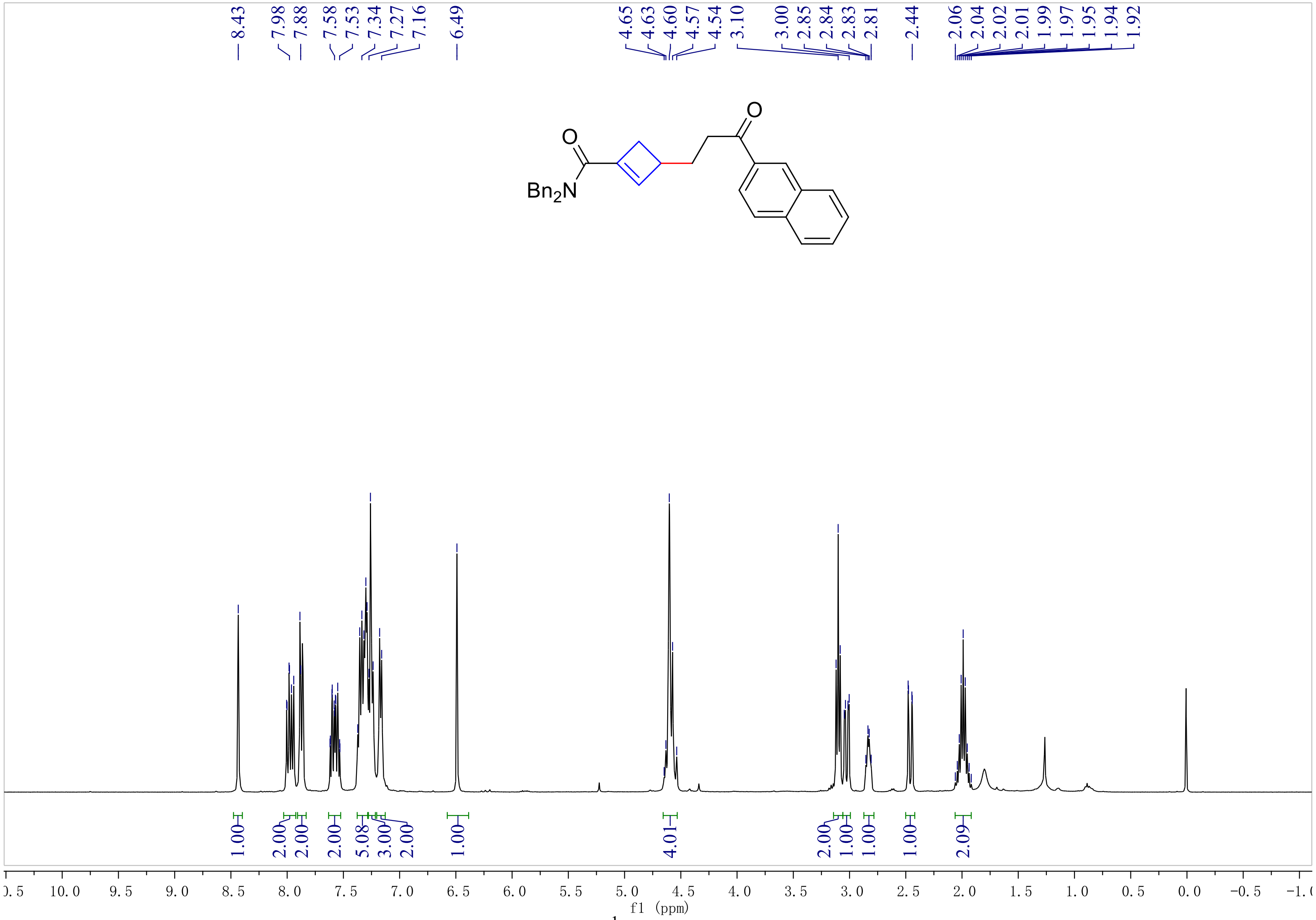

Supplementary Fig. 91 ^{1}H NMR (400 MHz, $CDCl_3$) of **3l**

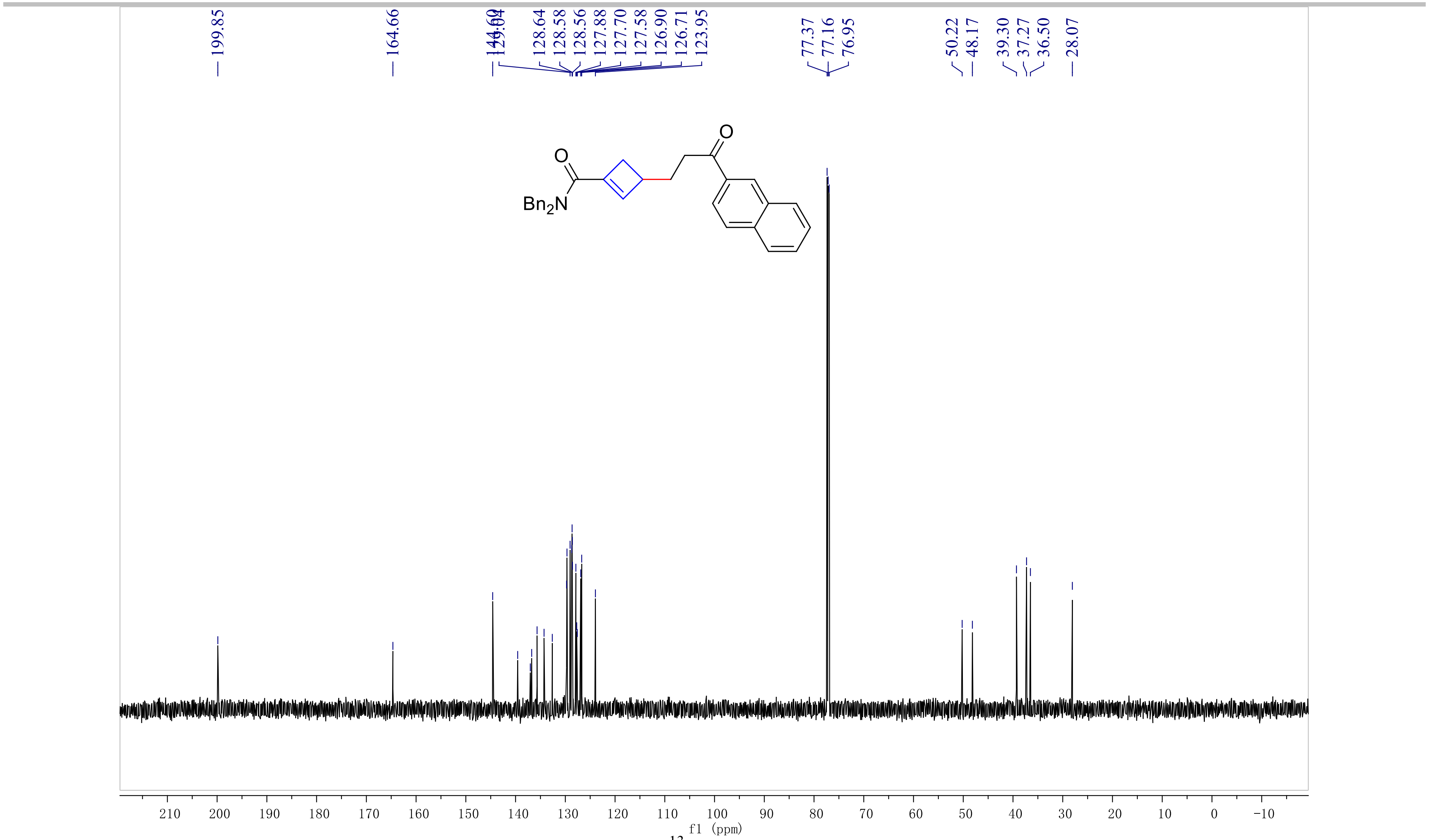

Supplementary Fig. 92 ^{13}C NMR (151 MHz, $CDCl_3$) of **3l**

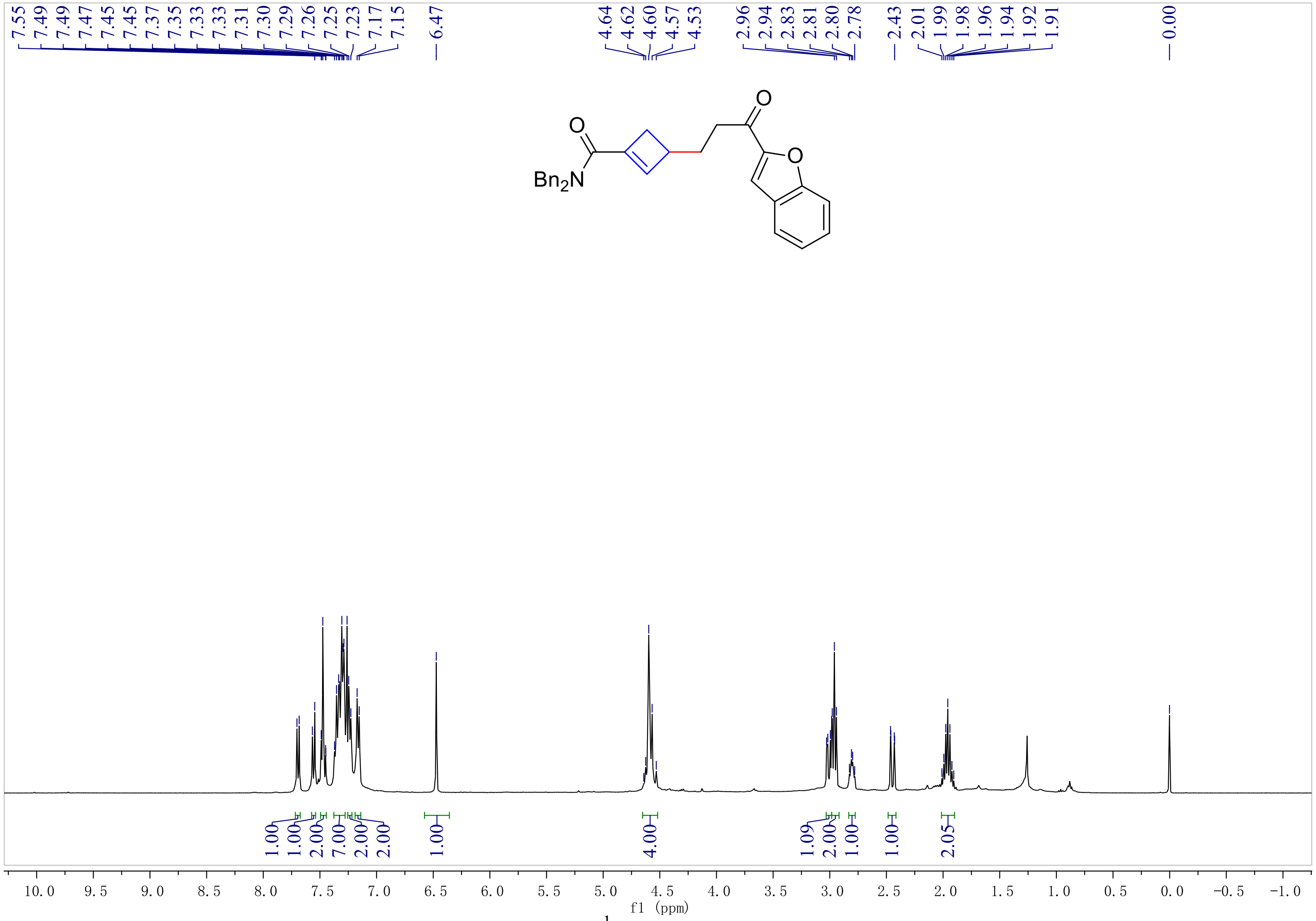

Supplementary Fig. 93 ^{1}H NMR (400 MHz, $CDCl_3$) of **3m**

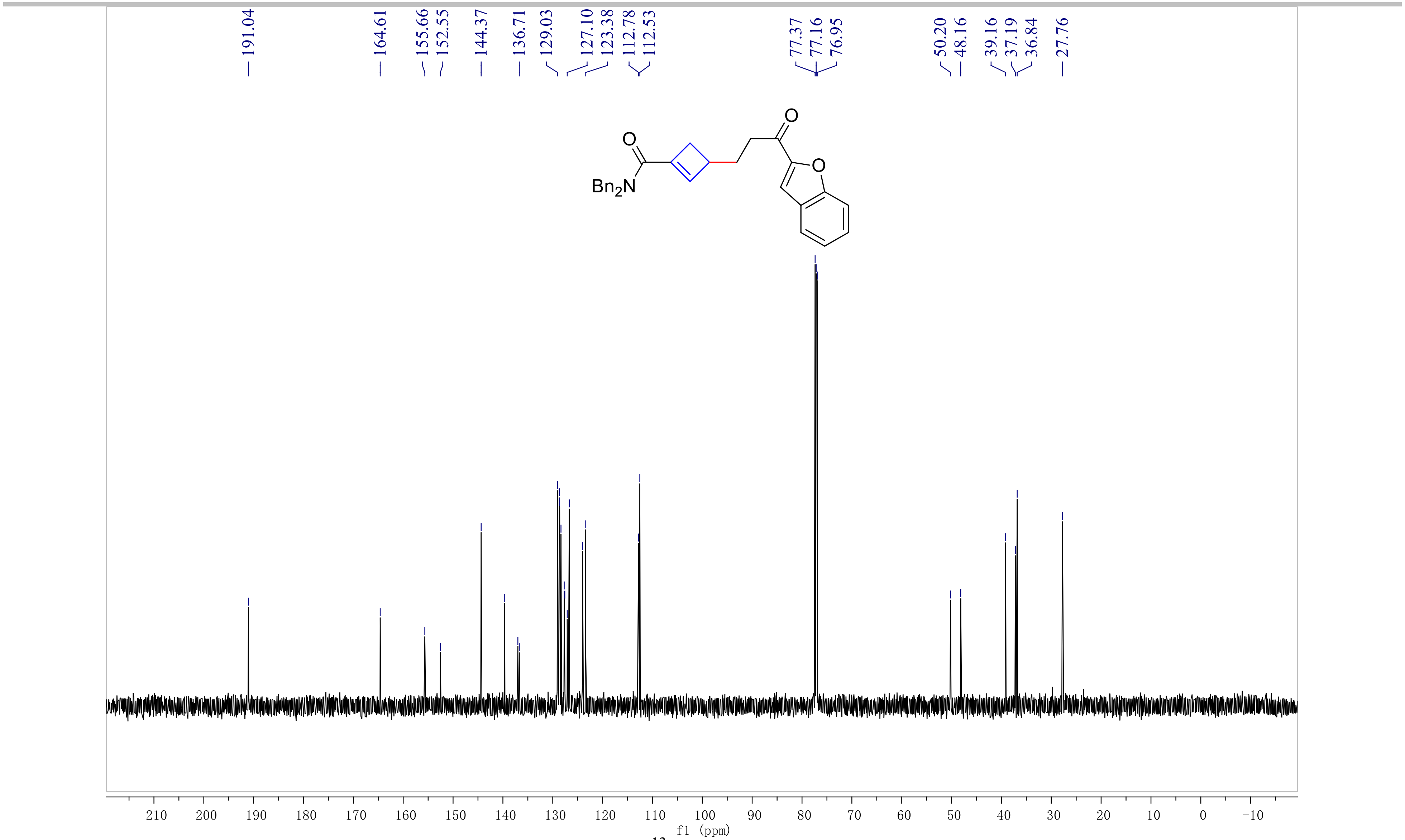

Supplementary Fig. 94 ^{13}C NMR (151 MHz, $CDCl_3$) of **3m**

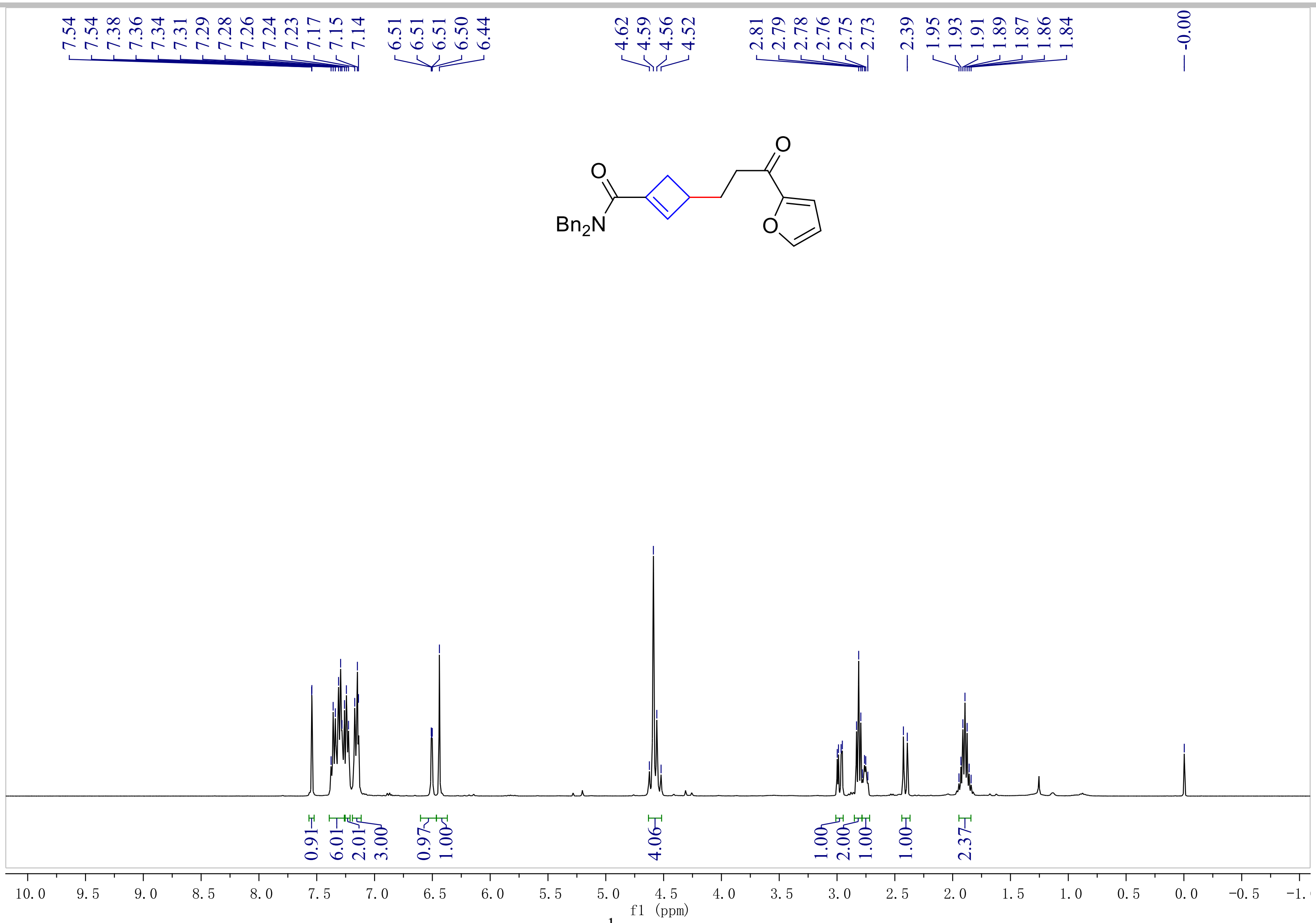

Supplementary Fig. 95 ^{1}H NMR (400 MHz, $CDCl_3$) of **3n**

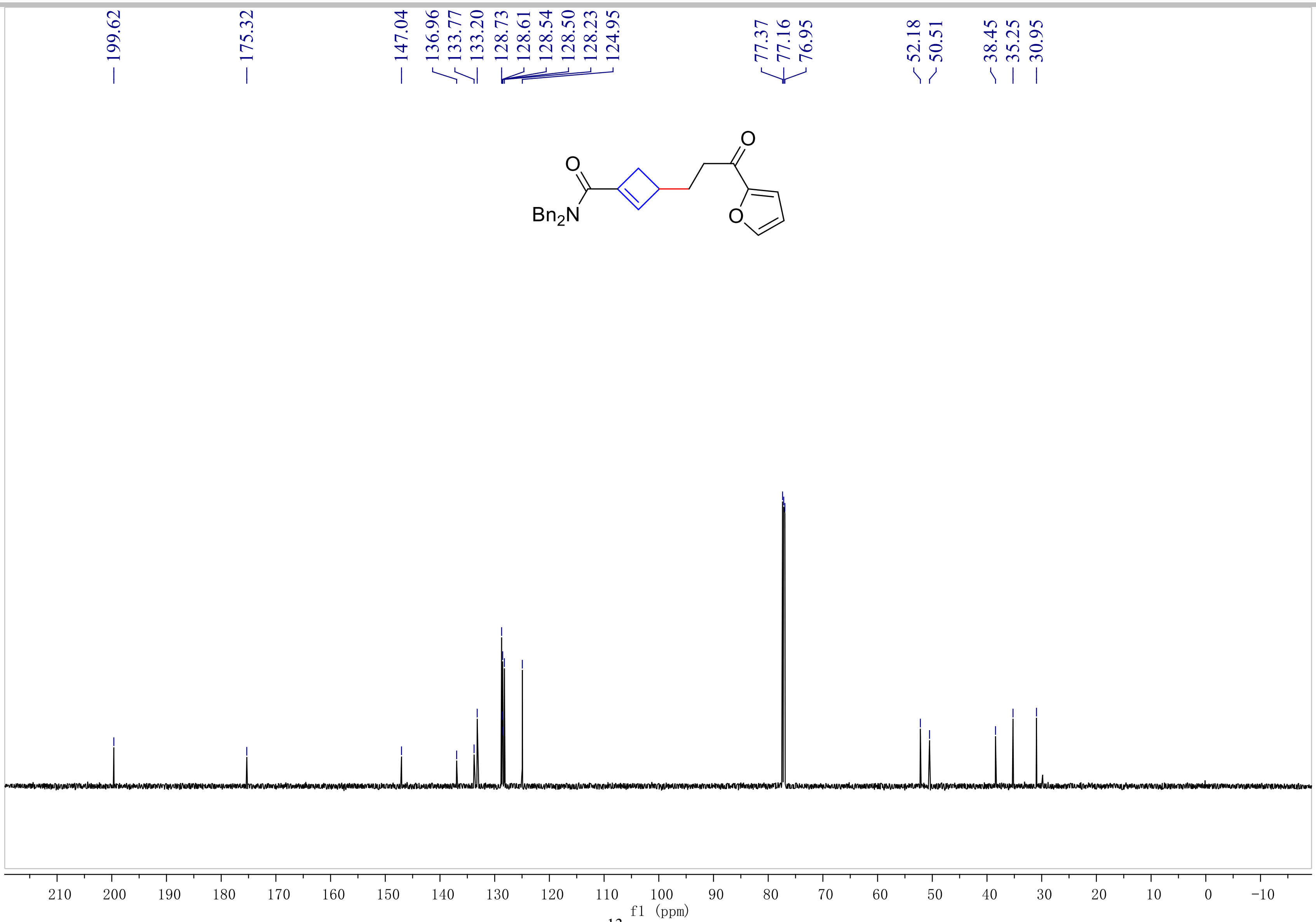

Supplementary Fig. 96 ^{13}C NMR (151 MHz, $CDCl_3$) of **3n**

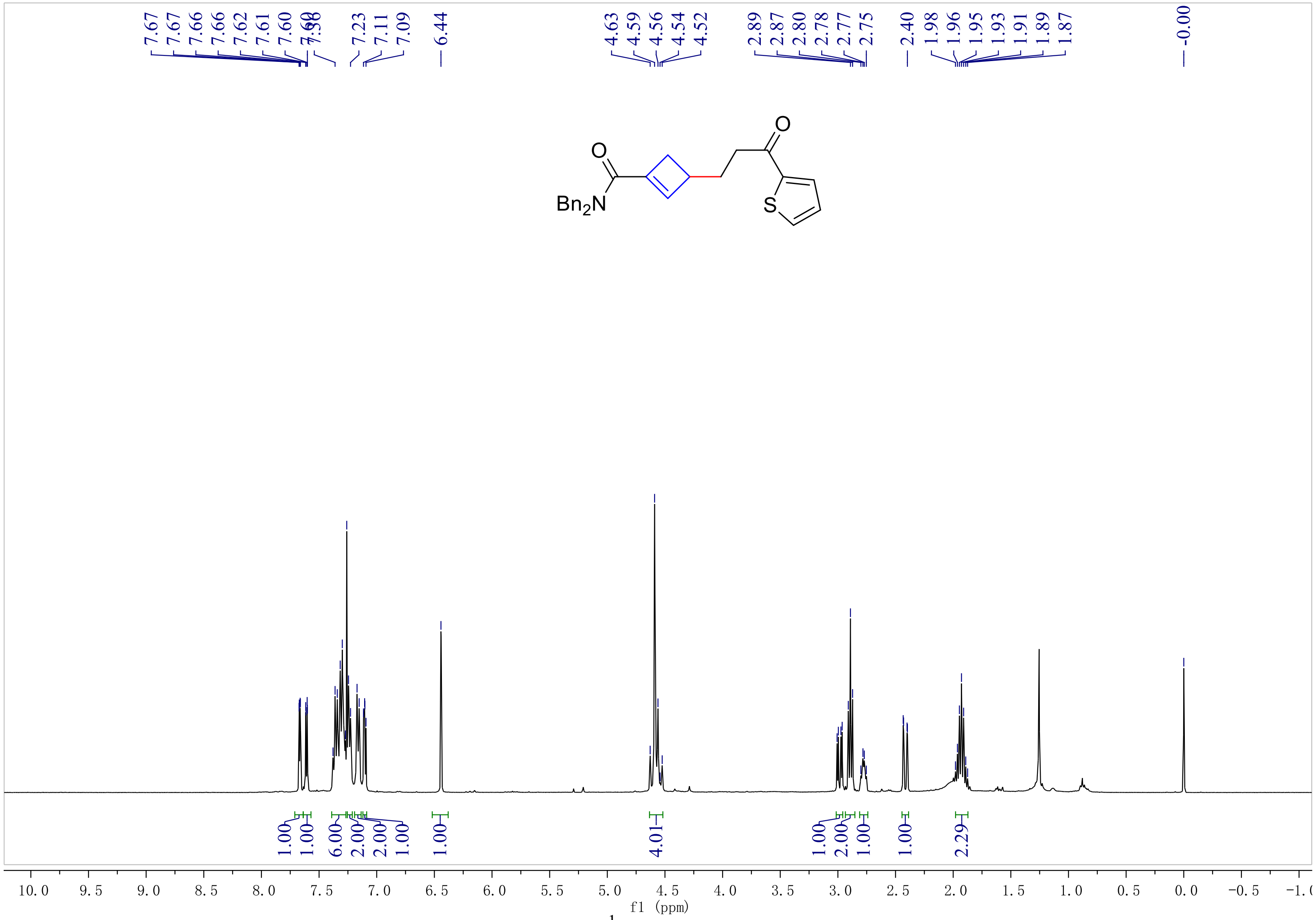

Supplementary Fig. 97 ^{1}H NMR (400 MHz, $CDCl_3$) of **3o**

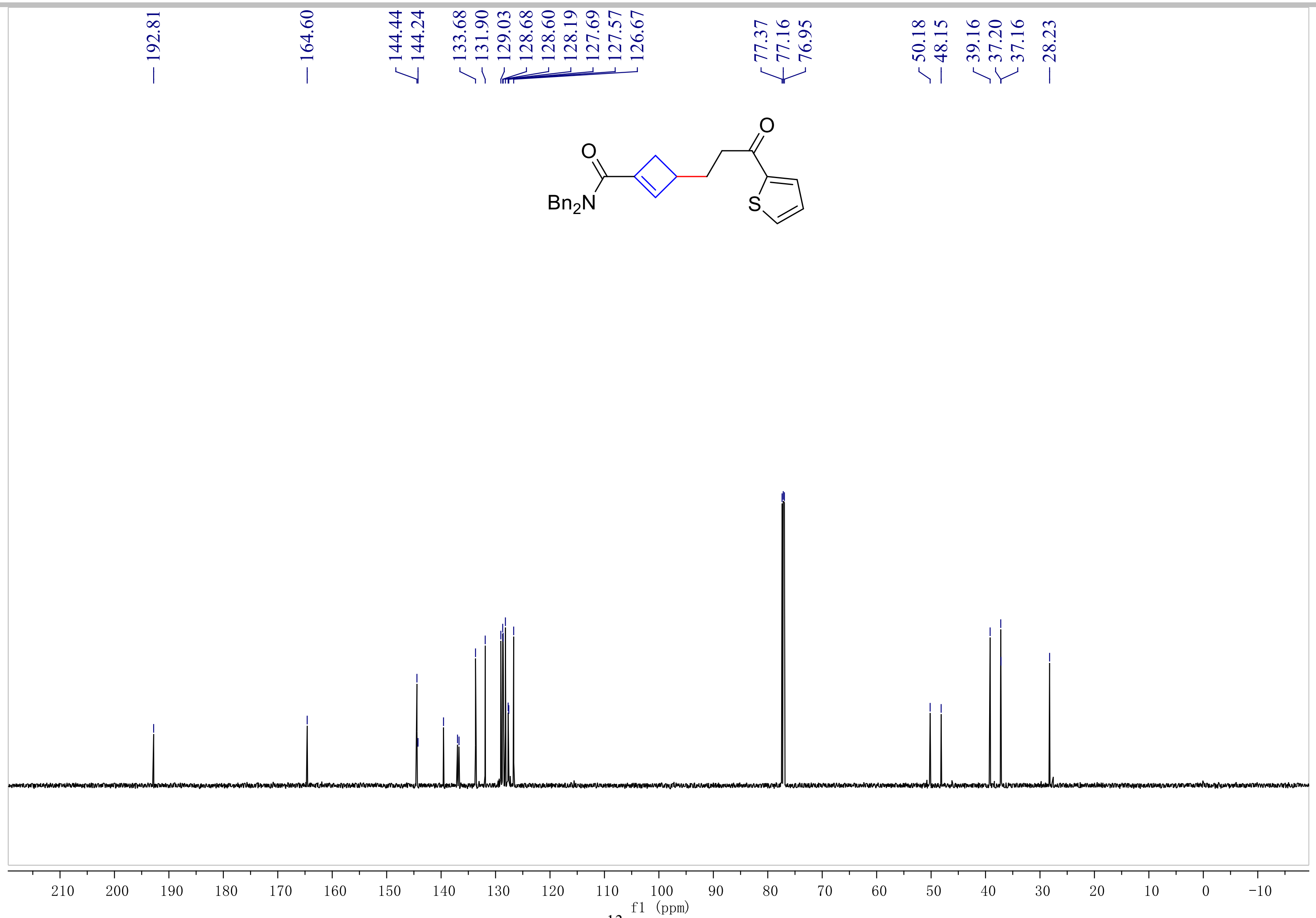

Supplementary Fig. 98 ^{13}C NMR (151 MHz, $CDCl_3$) of **3o**

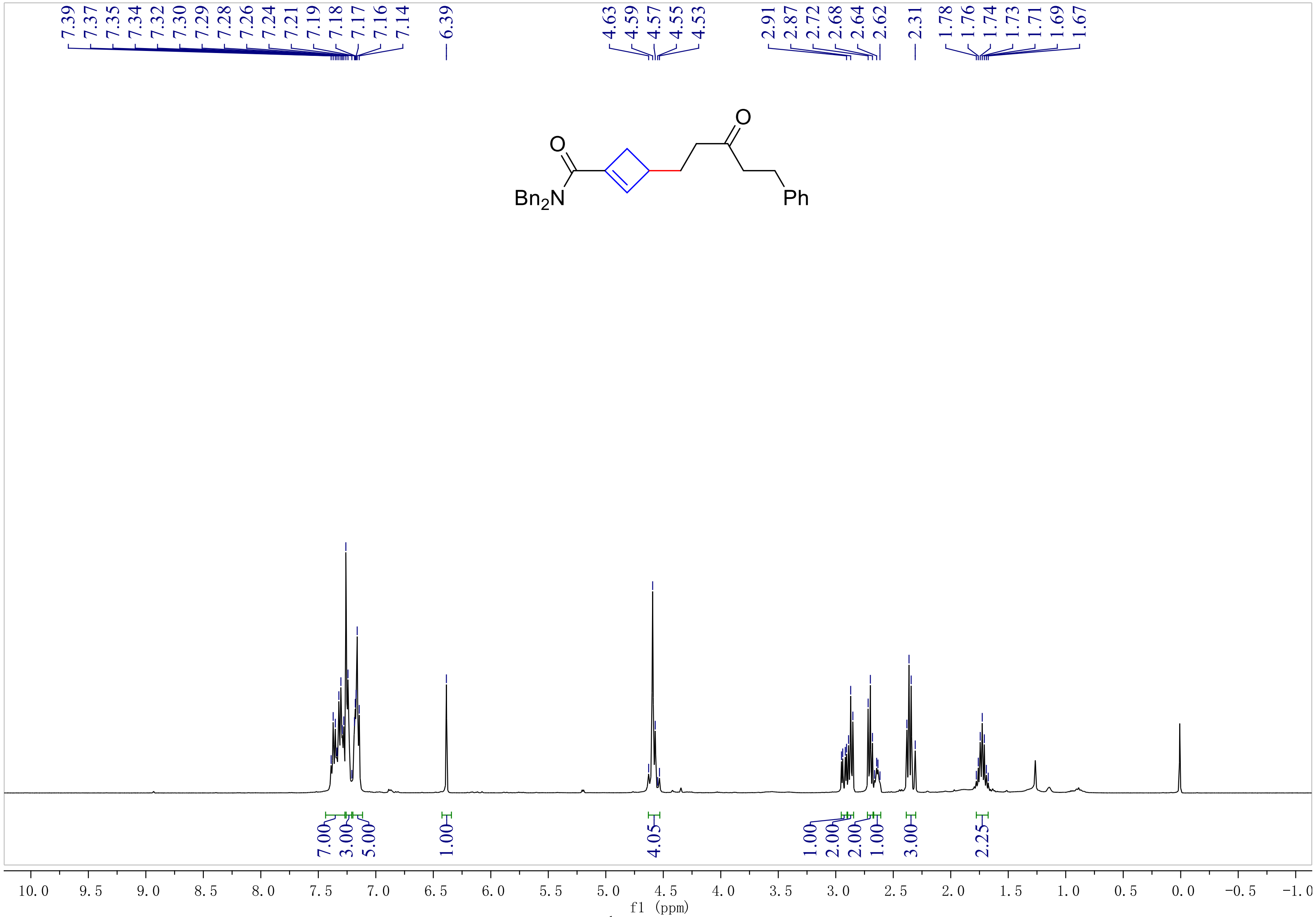

Supplementary Fig. 99 ^{1}H NMR (400 MHz, $CDCl_3$) of **3p**

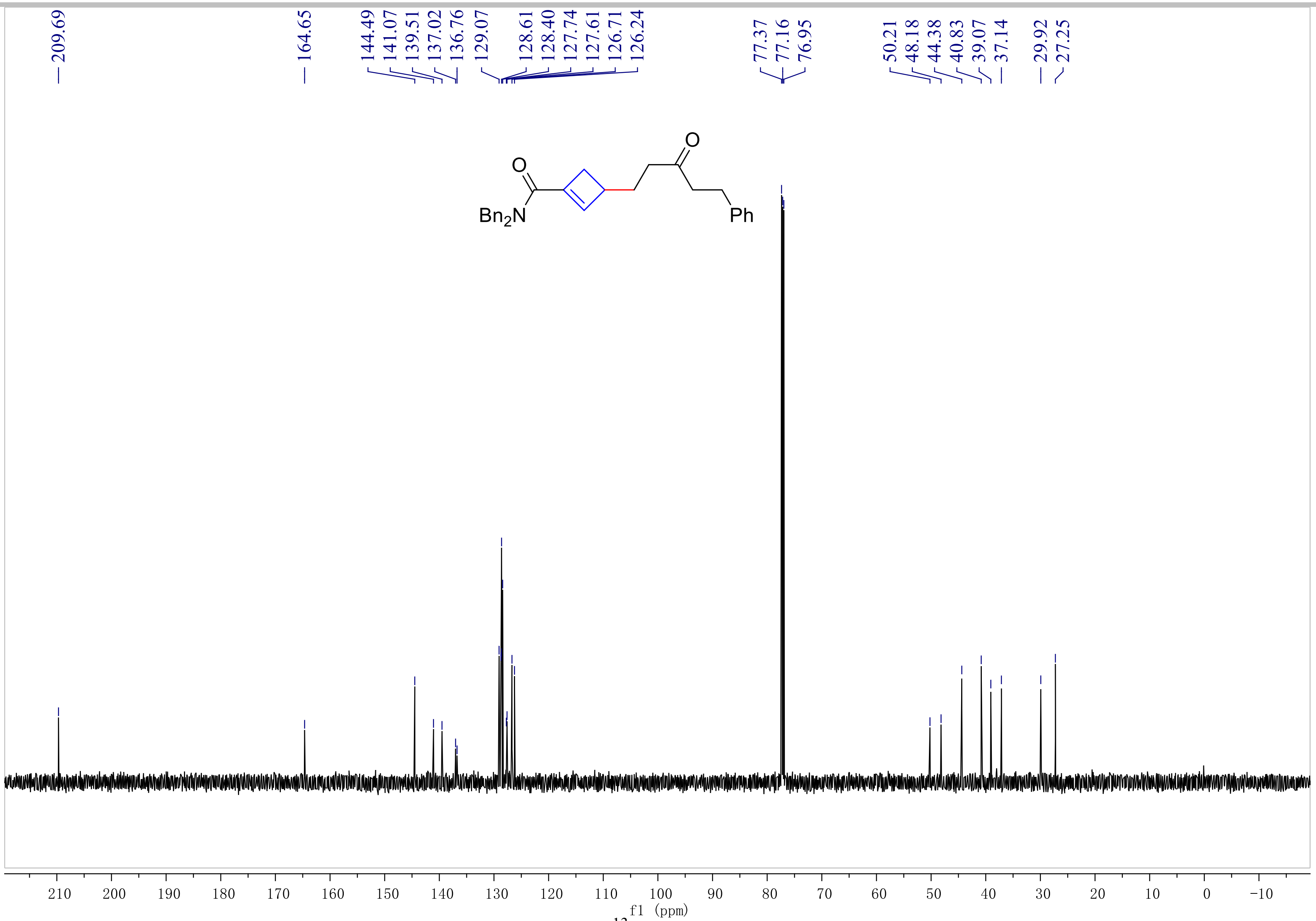

Supplementary Fig. 100 ^{13}C NMR (151 MHz, $CDCl_3$) of **3p**

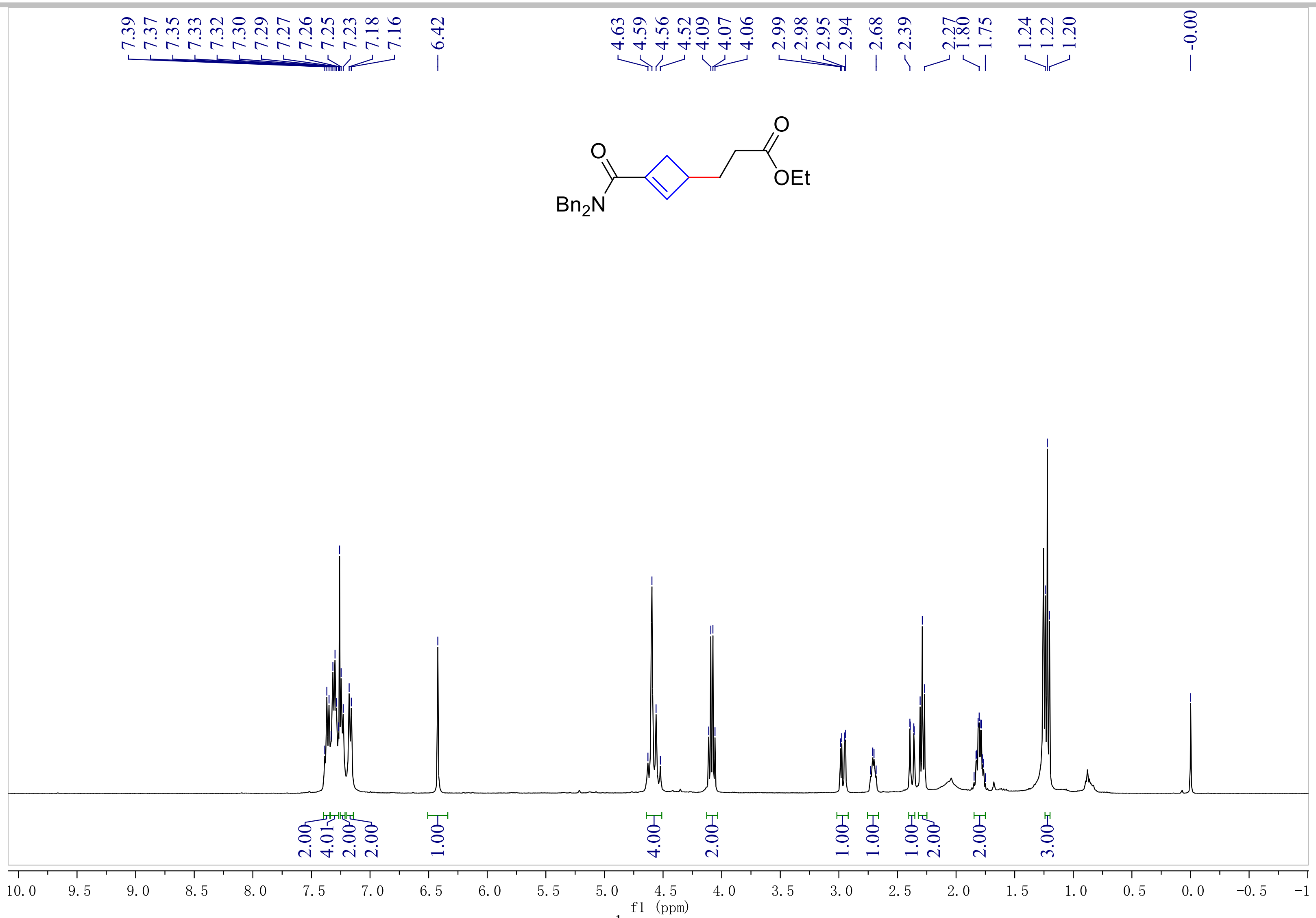

Supplementary Fig. 101 ^{1}H NMR (400 MHz, $CDCl_3$) of **3q**

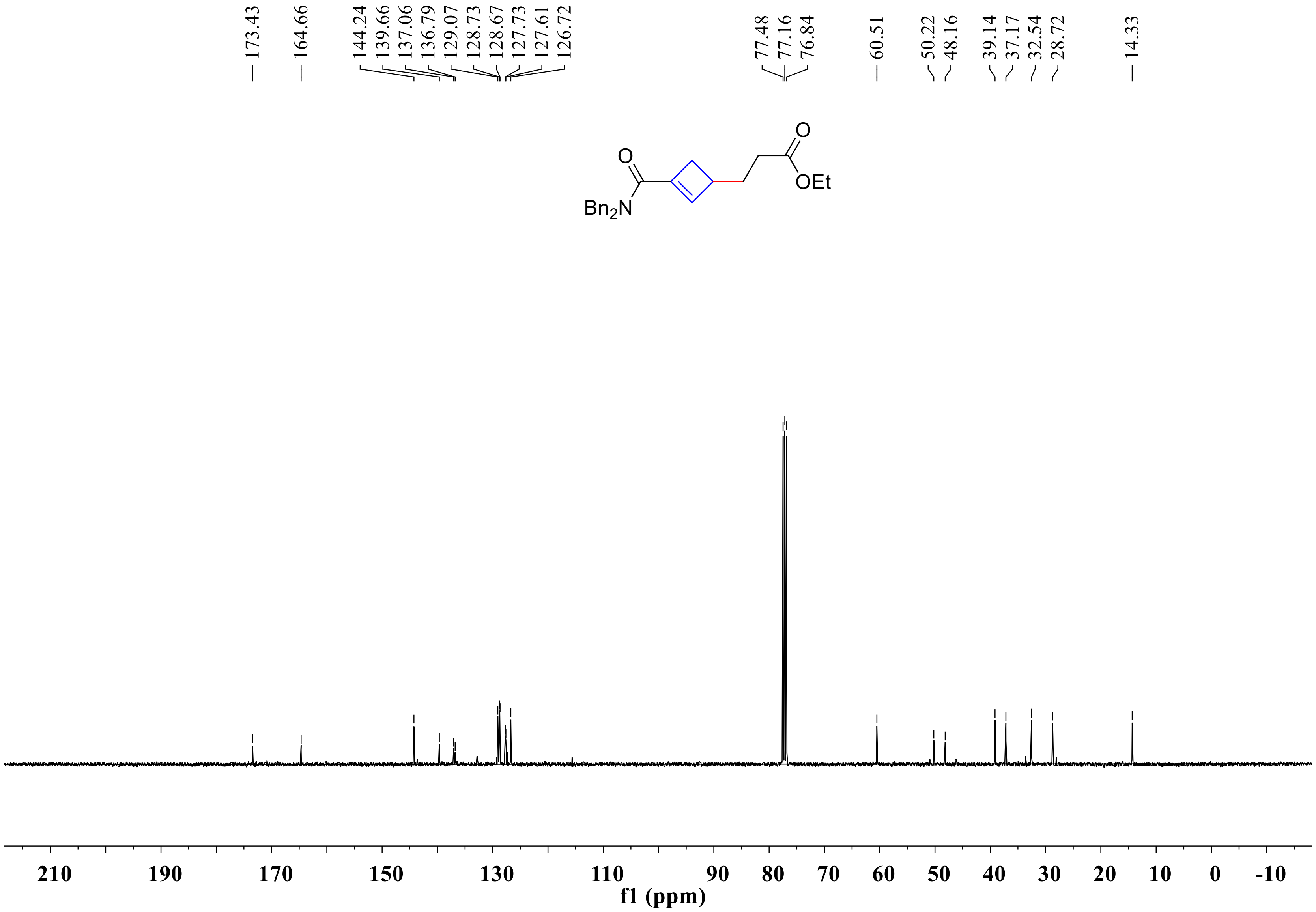

Supplementary Fig. 102 ^{13}C NMR (151 MHz, $CDCl_3$) of **3q**

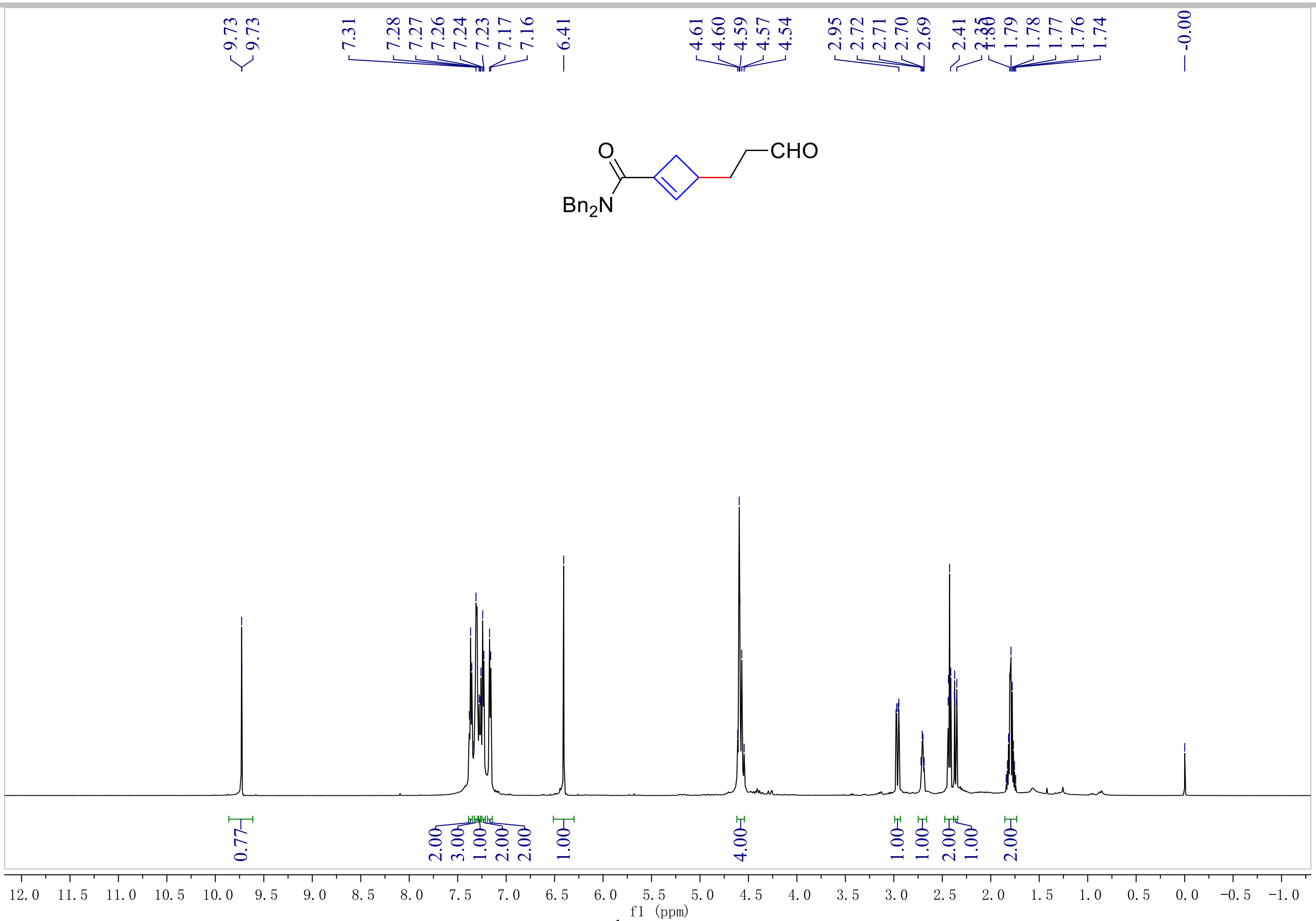

Supplementary Fig. 103 ^{1}H NMR (600 MHz, $CDCl_3$) of **3r**

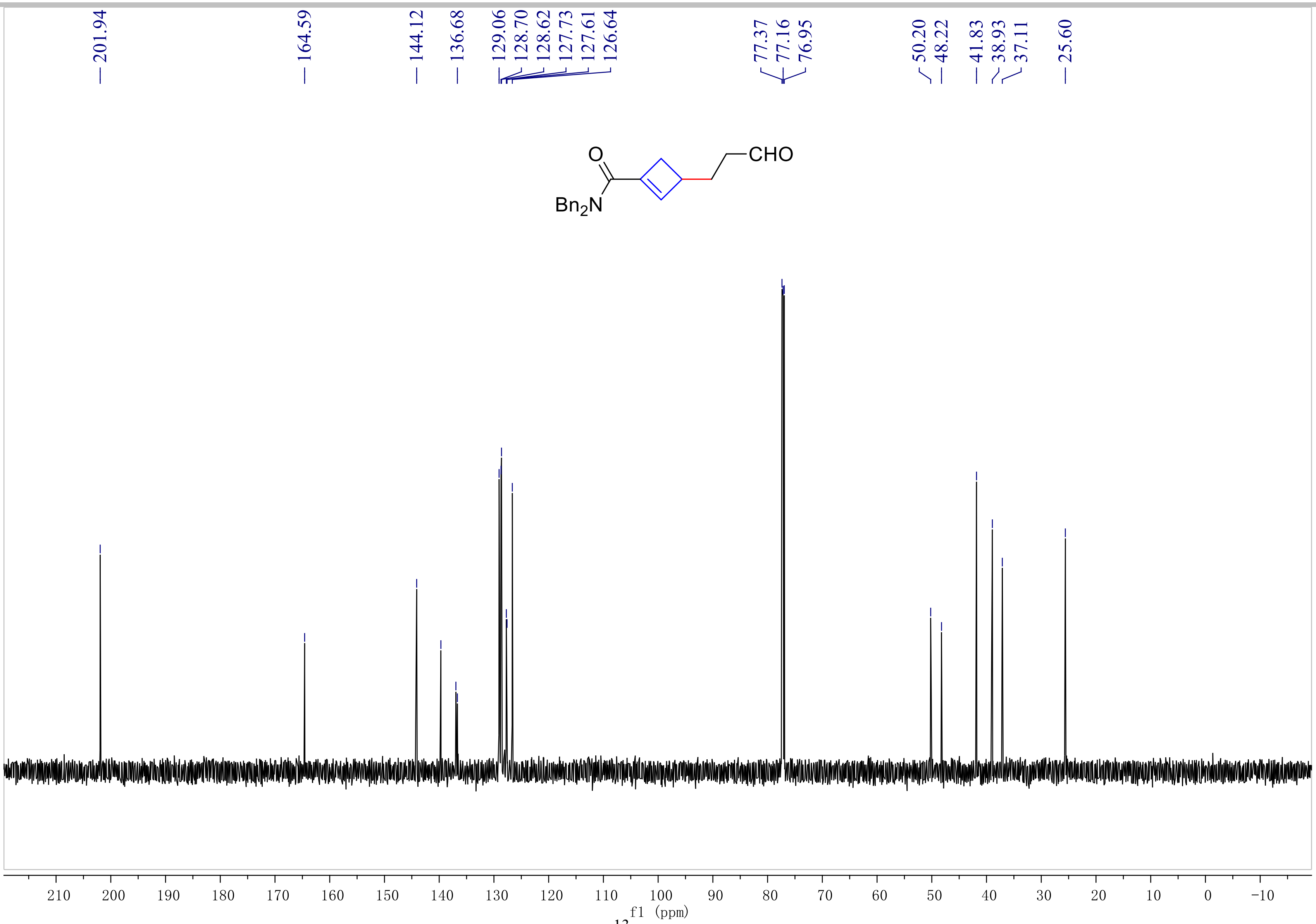

Supplementary Fig. 104 ^{13}C NMR (151 MHz, $CDCl_3$) of **3r**

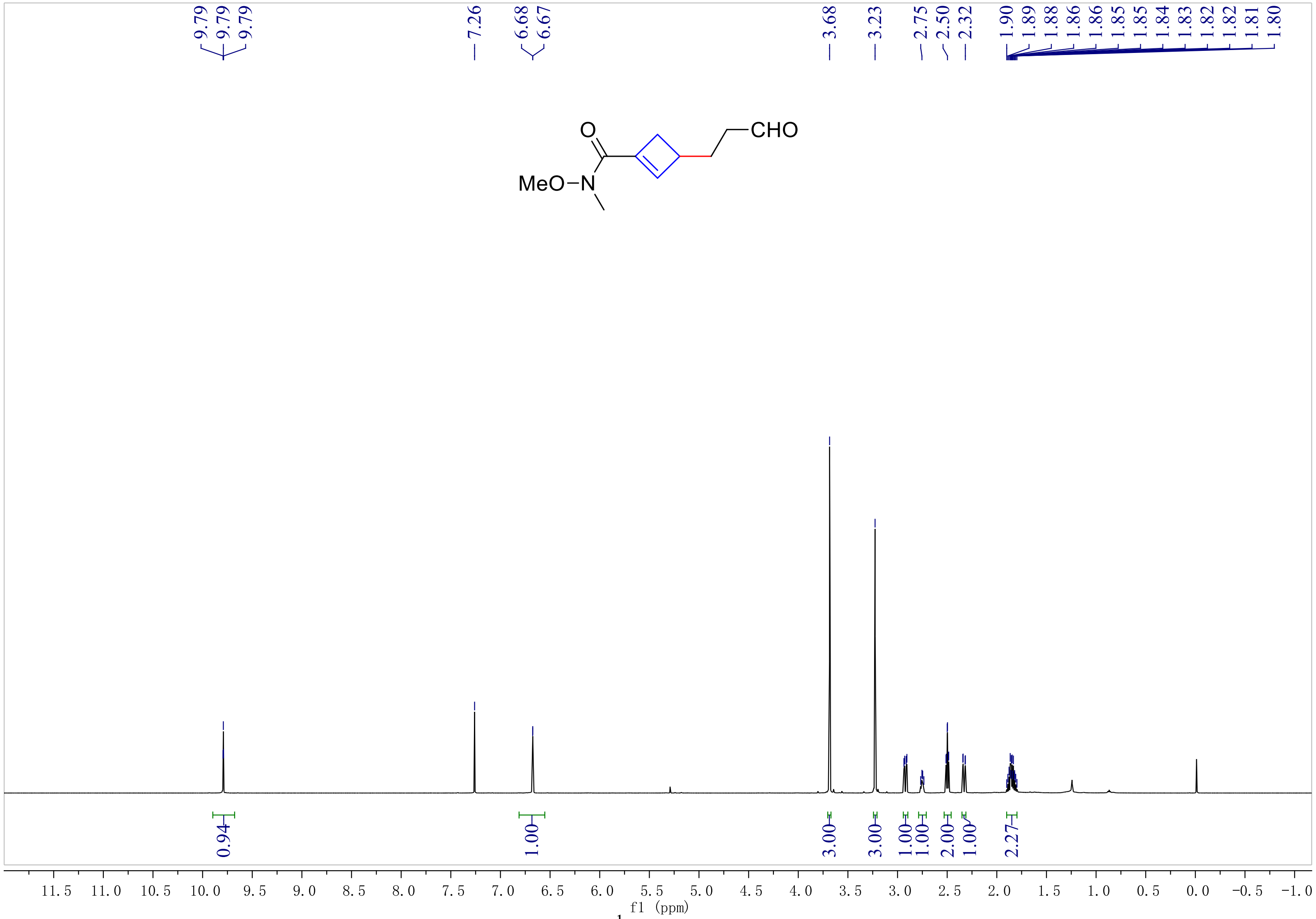

Supplementary Fig. 105 ^{1}H NMR (600 MHz, $CDCl_3$) of **3s**

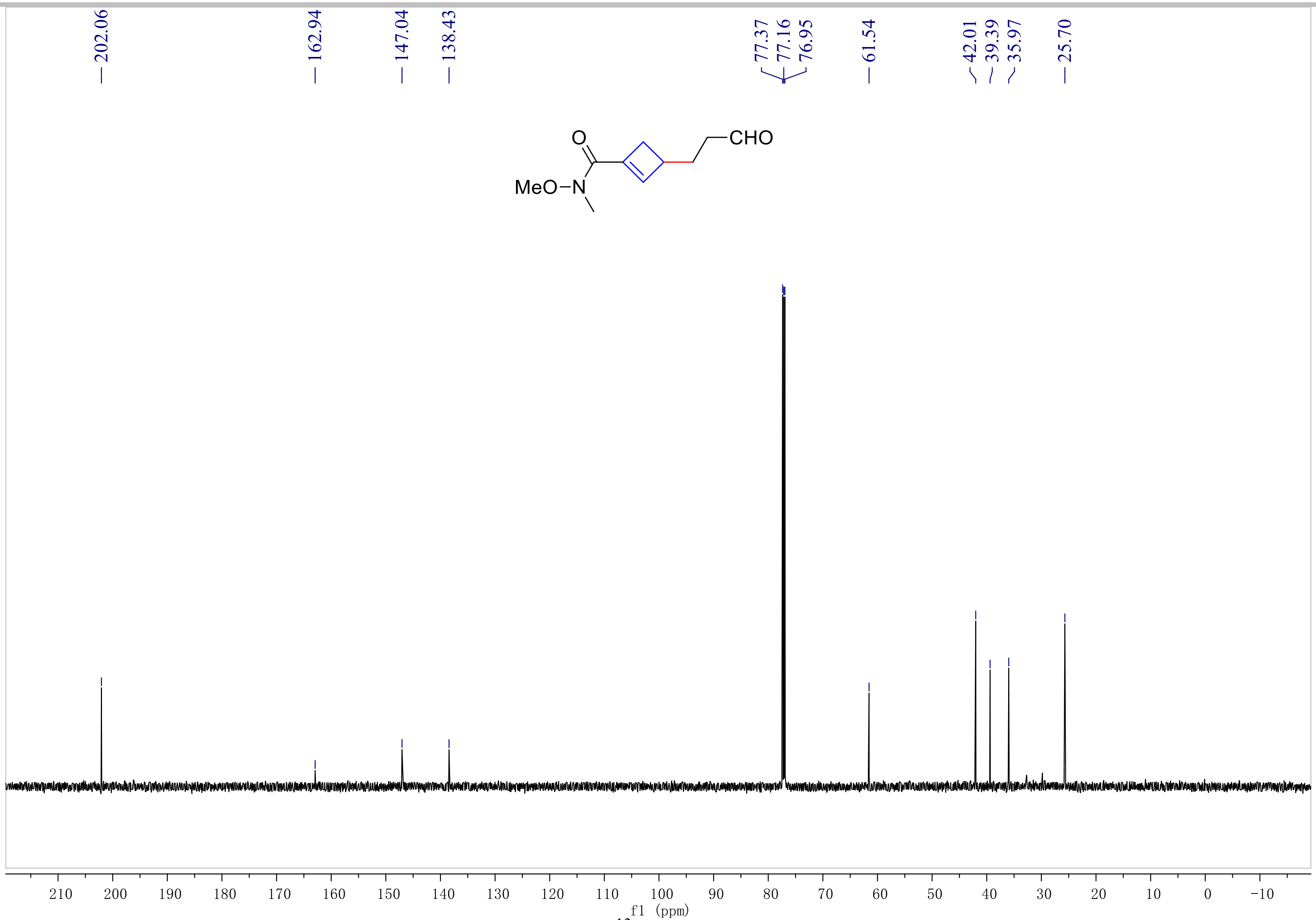

Supplementary Fig. 106 ^{13}C NMR (151 MHz, $CDCl_3$) of **3s**

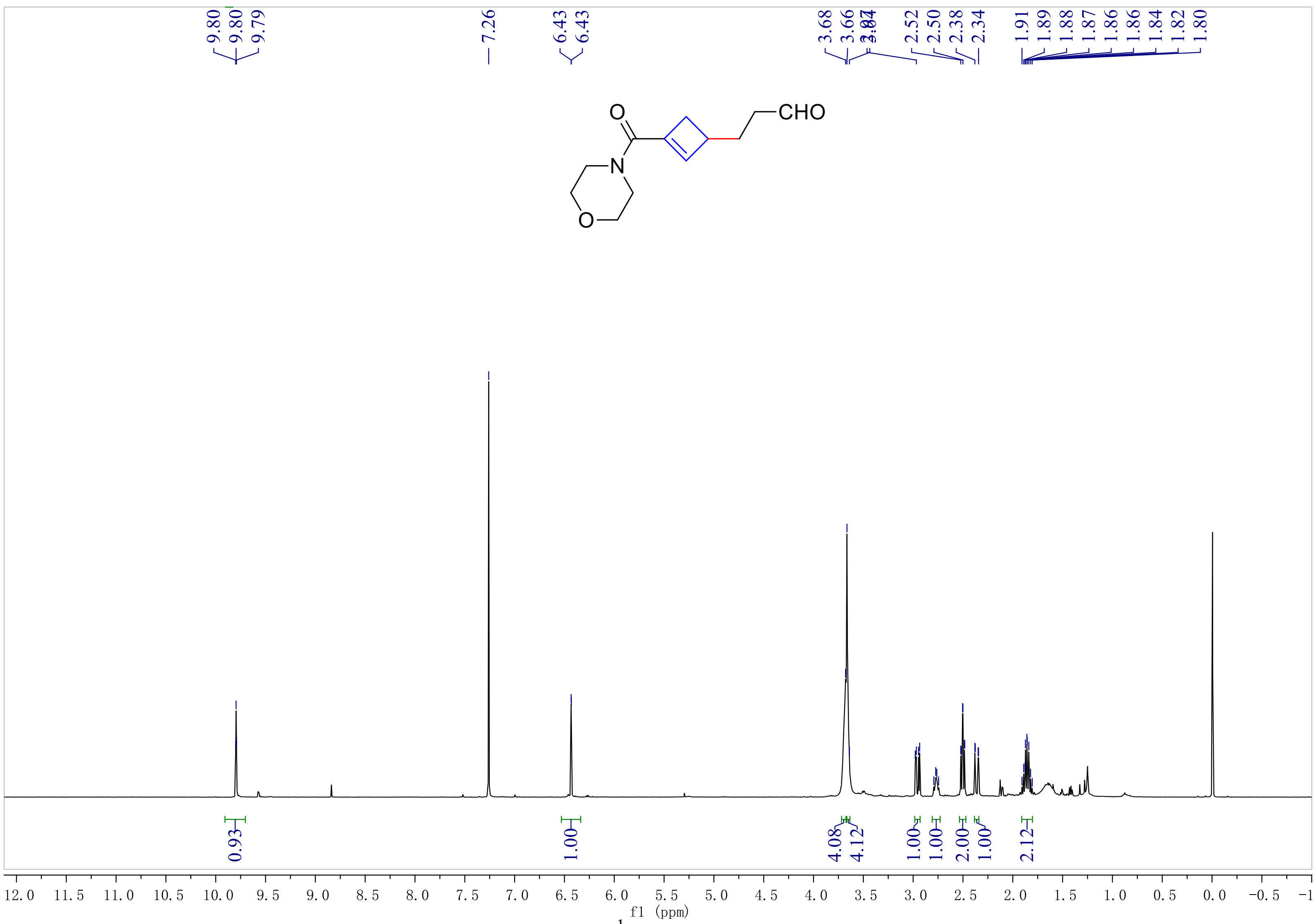

Supplementary Fig. 107 ^{1}H NMR (400 MHz, $CDCl_3$) of **3t**

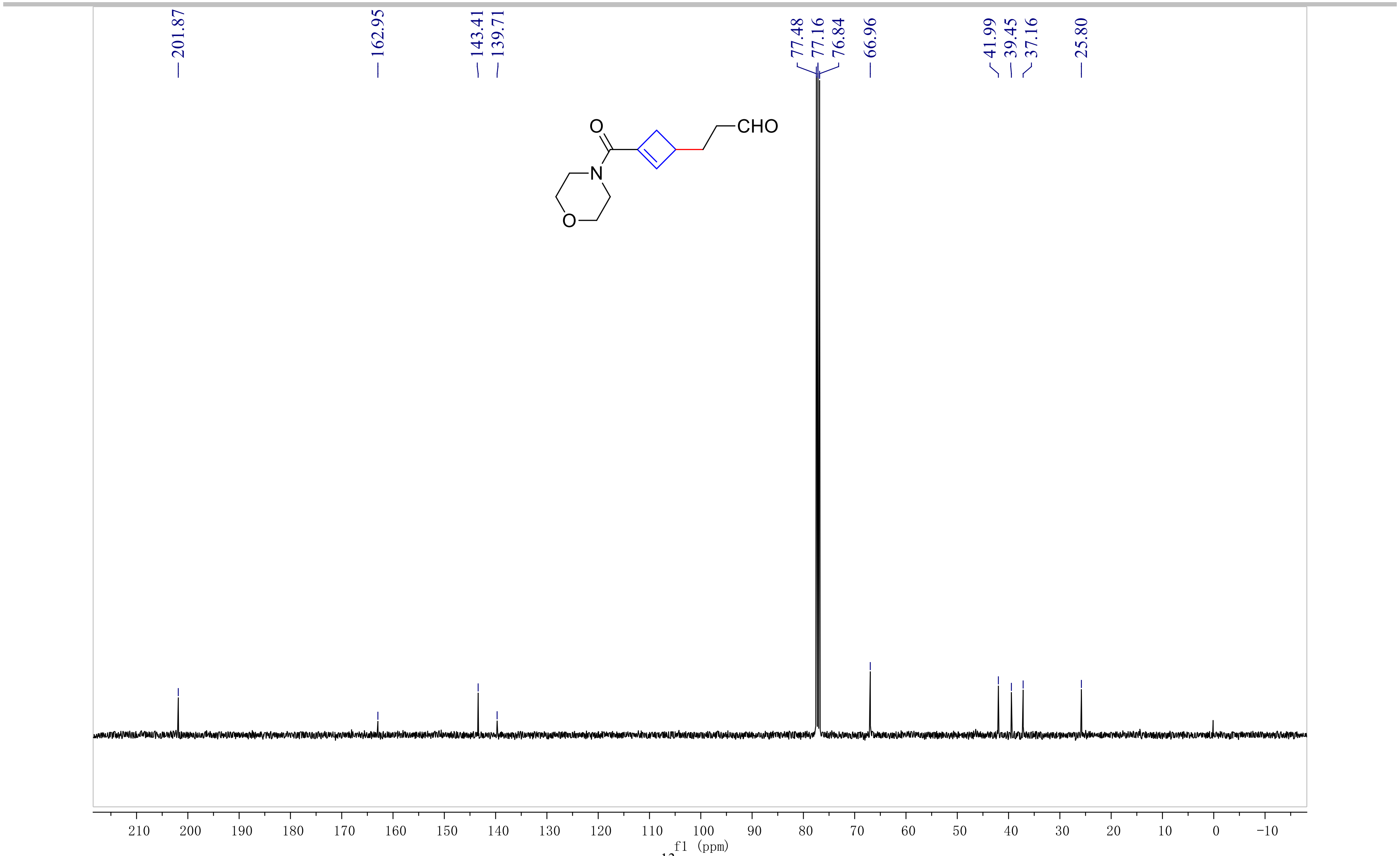

Supplementary Fig. 108 ^{13}C NMR (151 MHz, $CDCl_3$) of **3t**

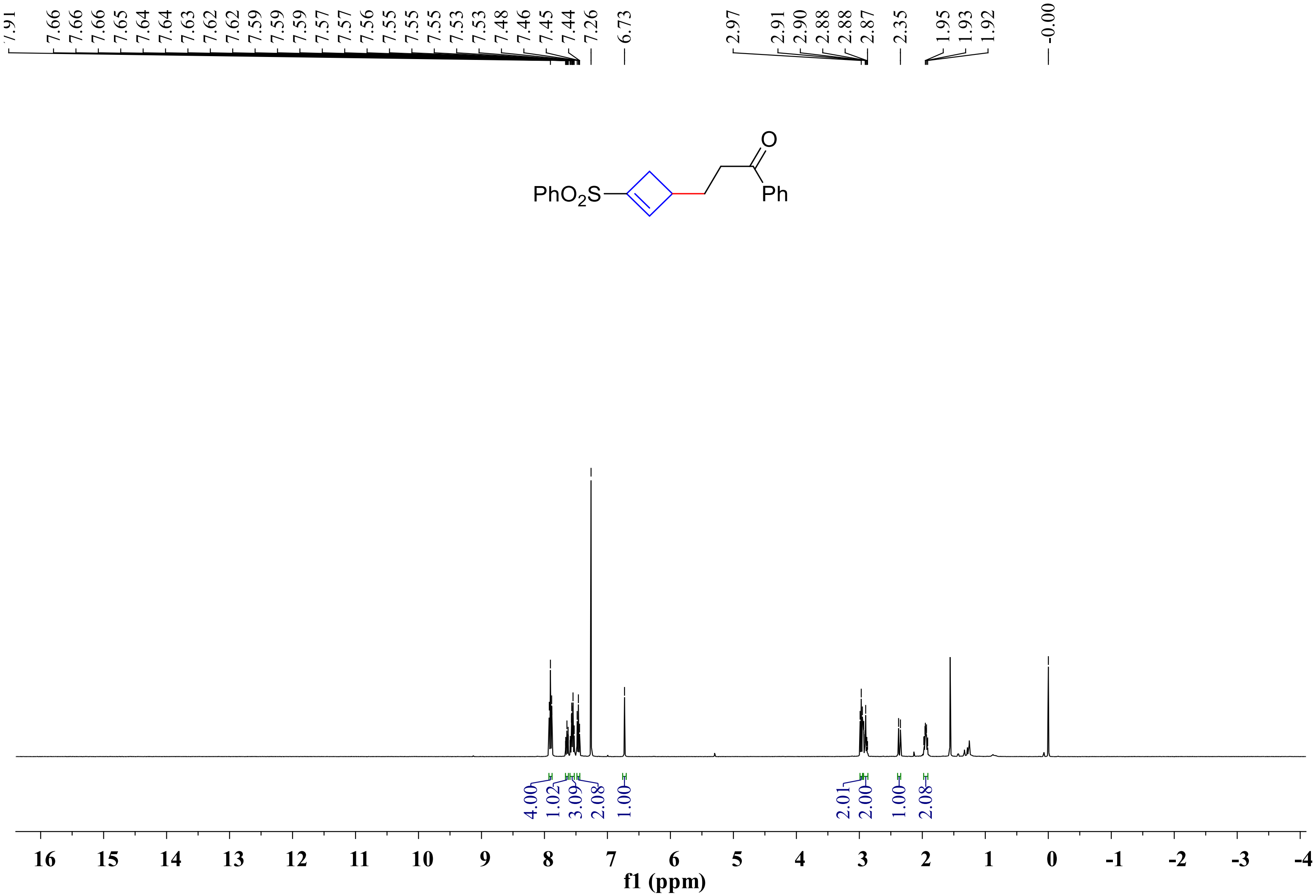

Supplementary Fig. 109 ^{1}H NMR (400 MHz, $CDCl_3$) of **3u**

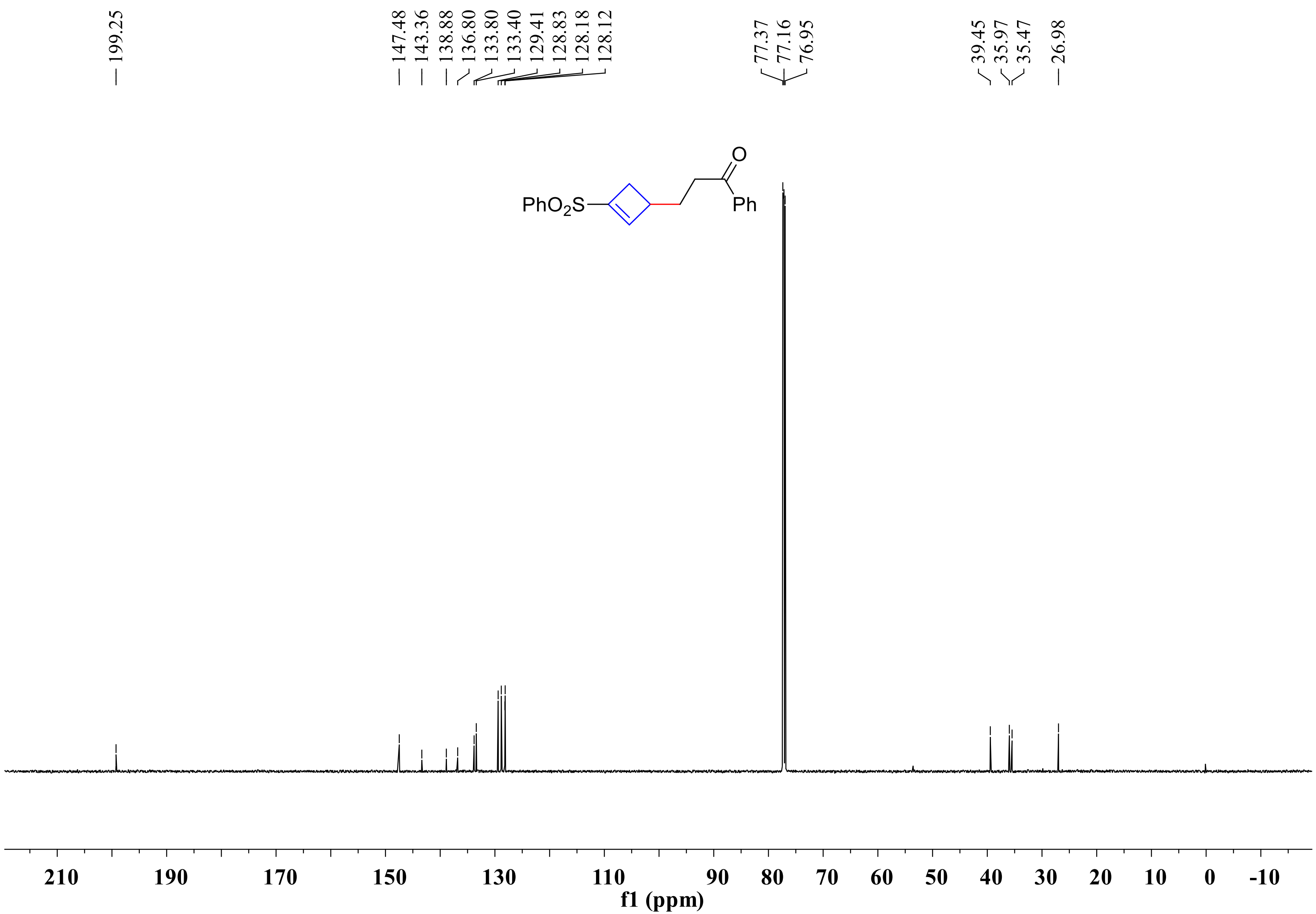

Supplementary Fig. 110 ^{13}C NMR (151 MHz, $CDCl_3$) of **3u**

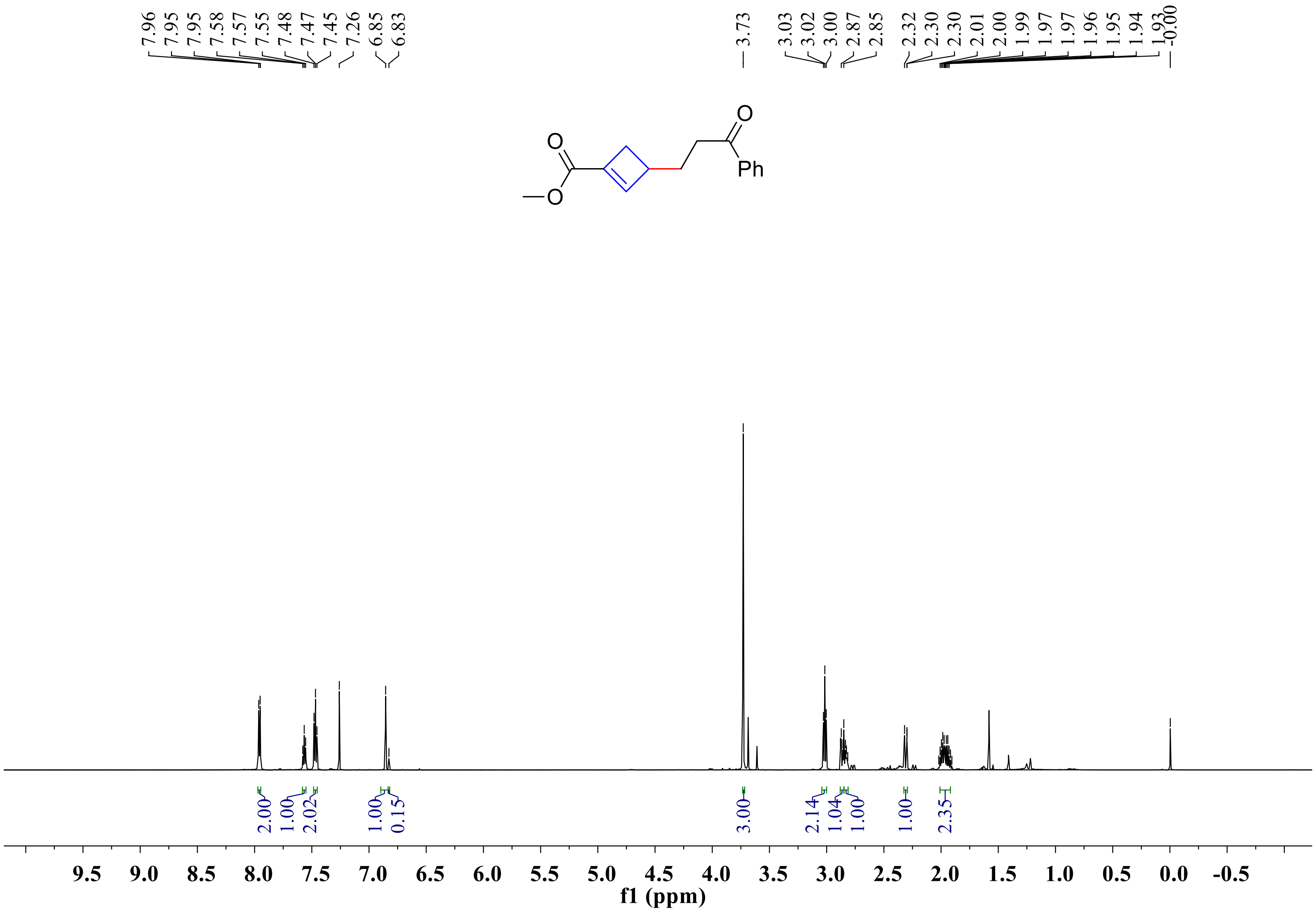

Supplementary Fig. 111 ^{1}H NMR (600 MHz, $CDCl_3$) of **3v**

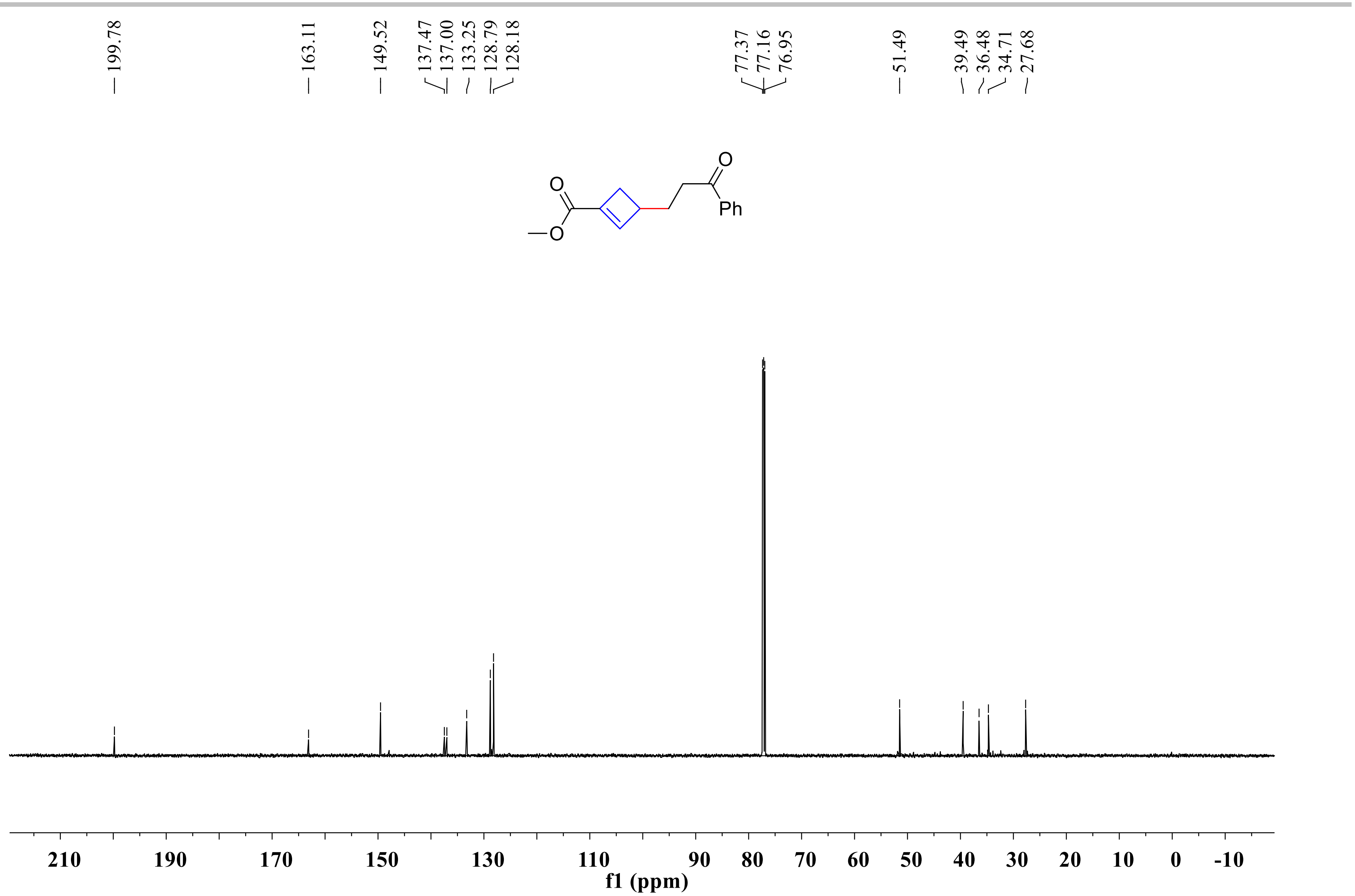

Supplementary Fig. 112 ^{13}C NMR (151 MHz, $CDCl_3$) of **3v**

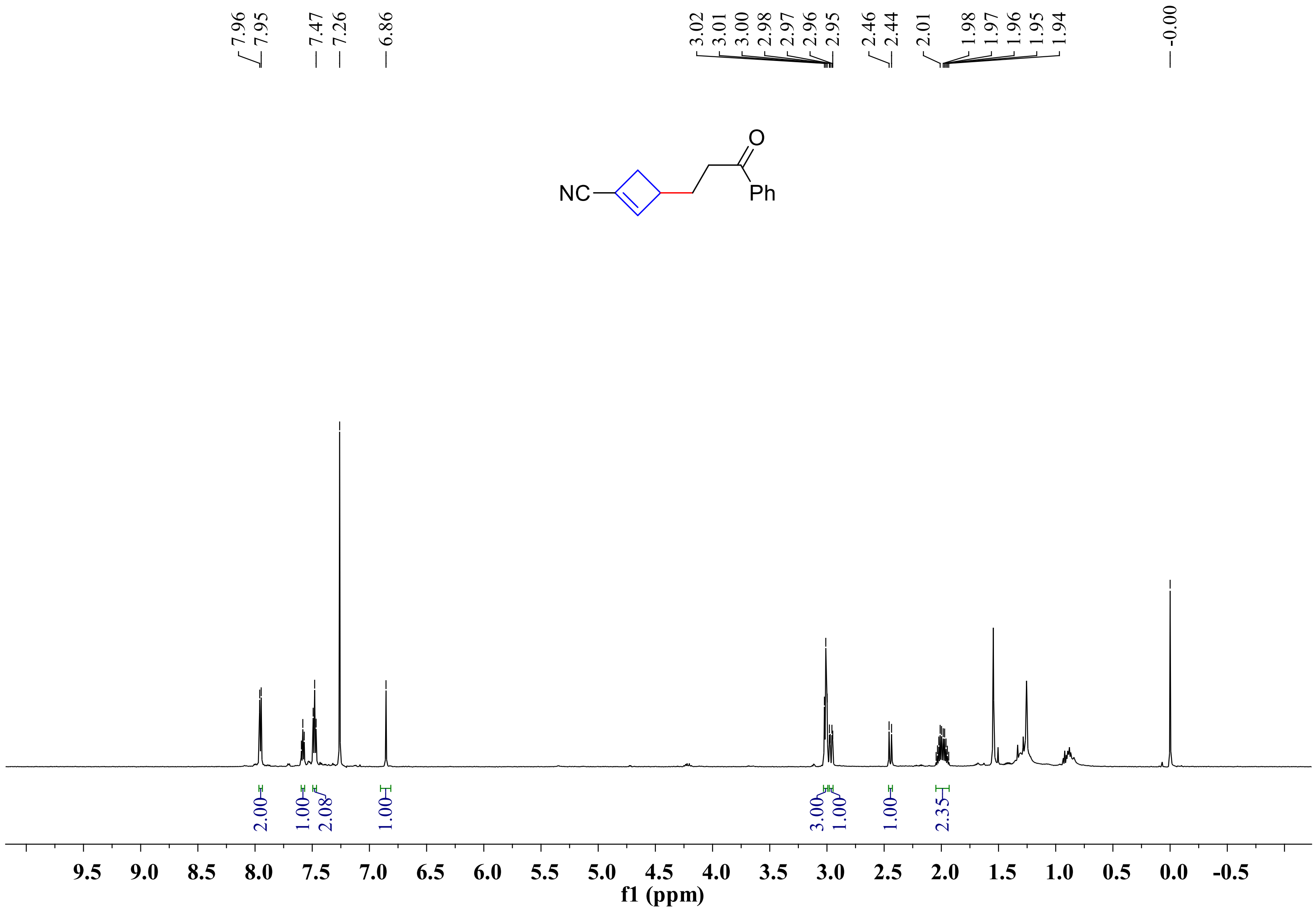

Supplementary Fig. 113 ^{1}H NMR (600 MHz, $CDCl_3$) of **3w**

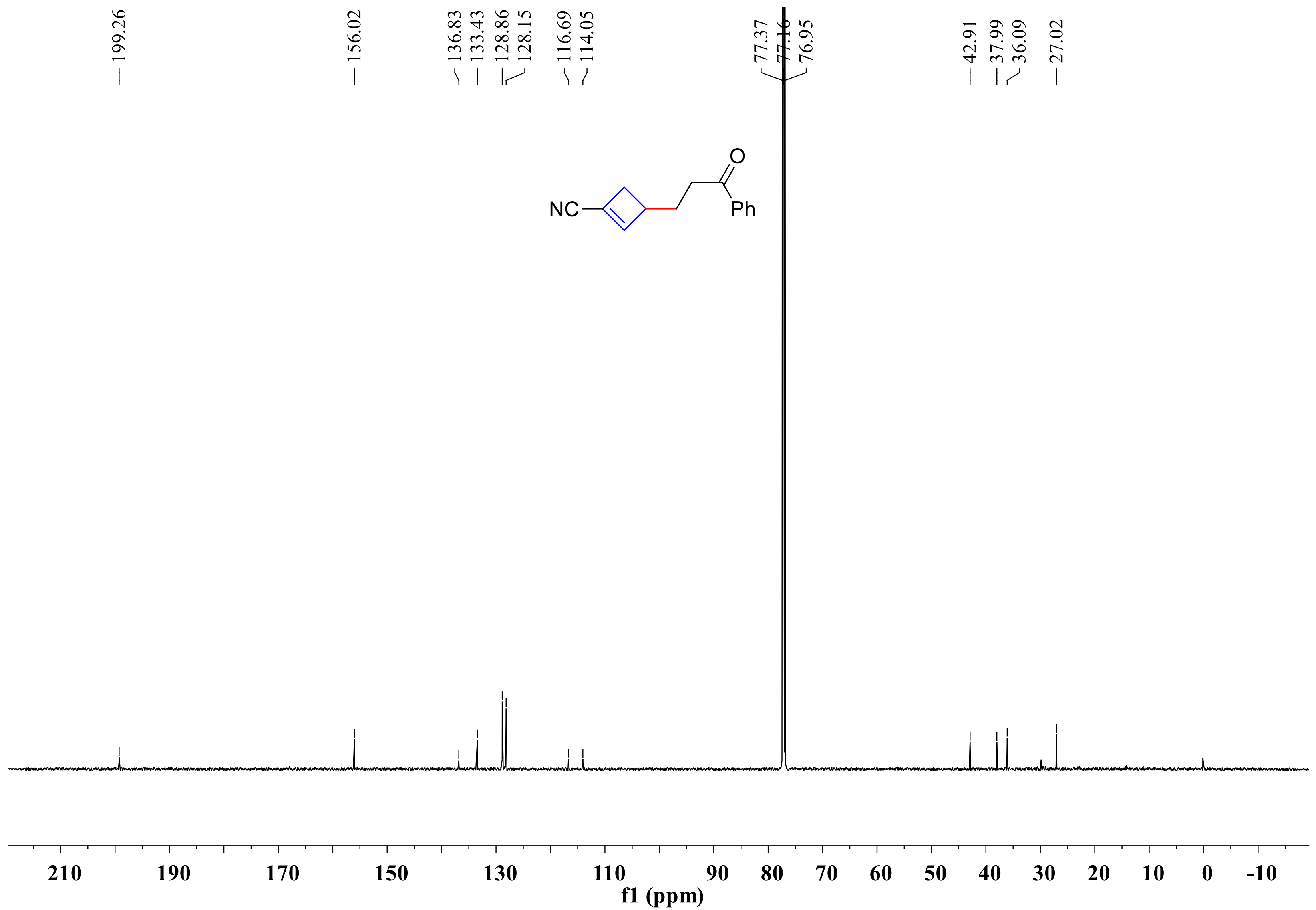

Supplementary Fig. 114 ^{13}C NMR (151 MHz, $CDCl_3$) of **3w**

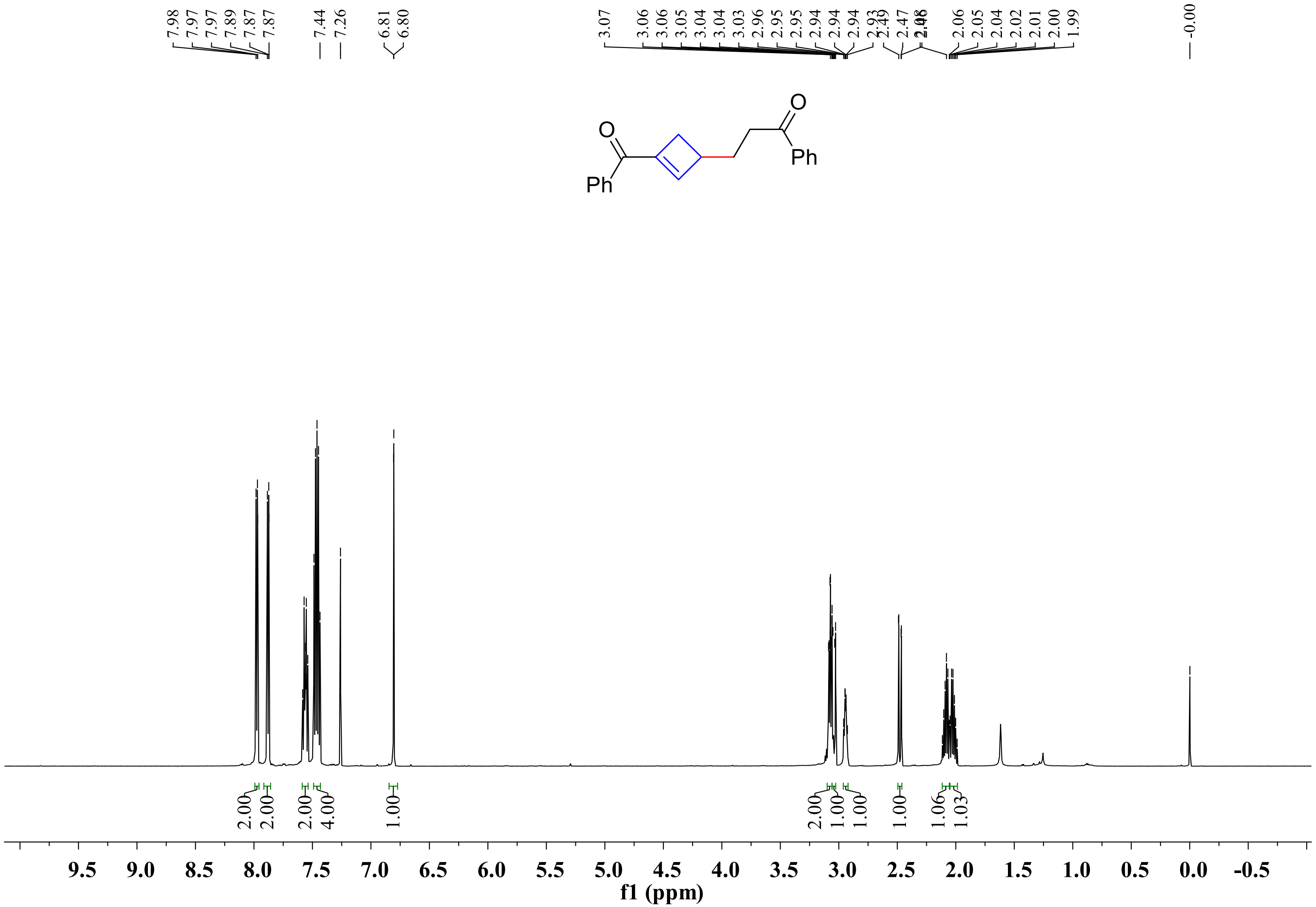

Supplementary Fig. 115 ^{1}H NMR (600 MHz, $CDCl_3$) of **3x**

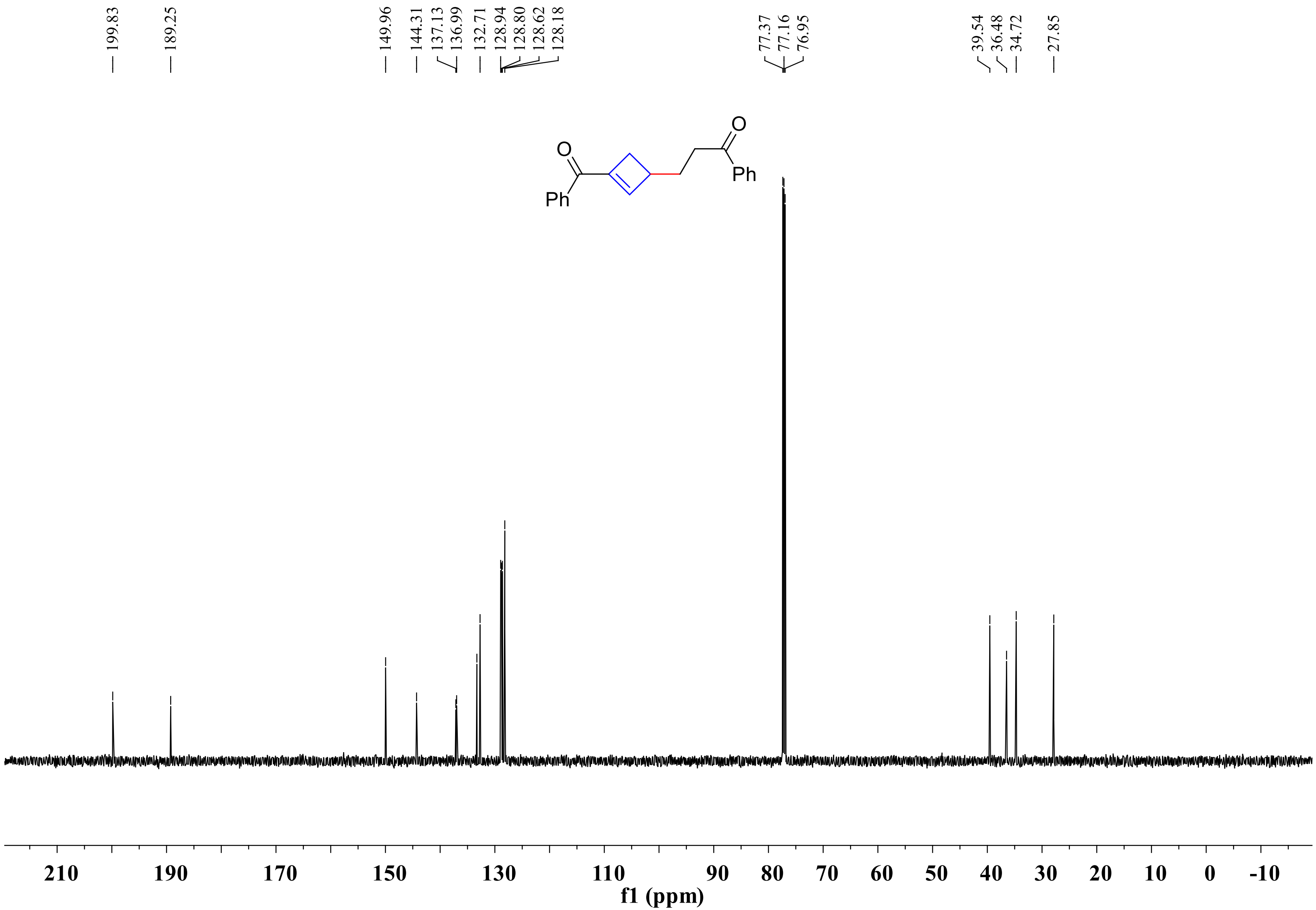

Supplementary Fig. 116 ^{13}C NMR (151 MHz, $CDCl_3$) of **3x**

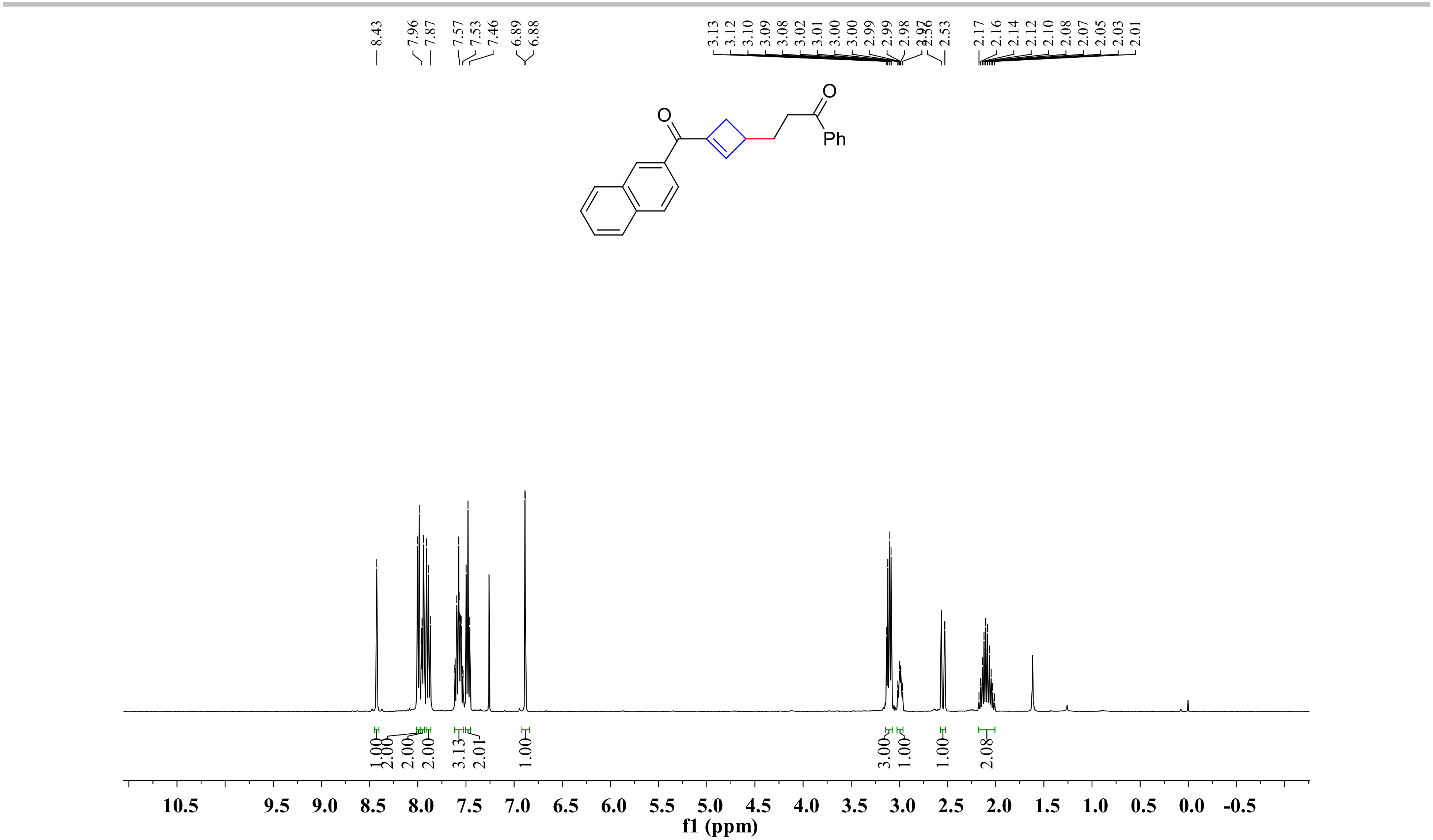

Supplementary Fig. 117 ^{1}H NMR (400 MHz, $CDCl_3$) of **3xa**

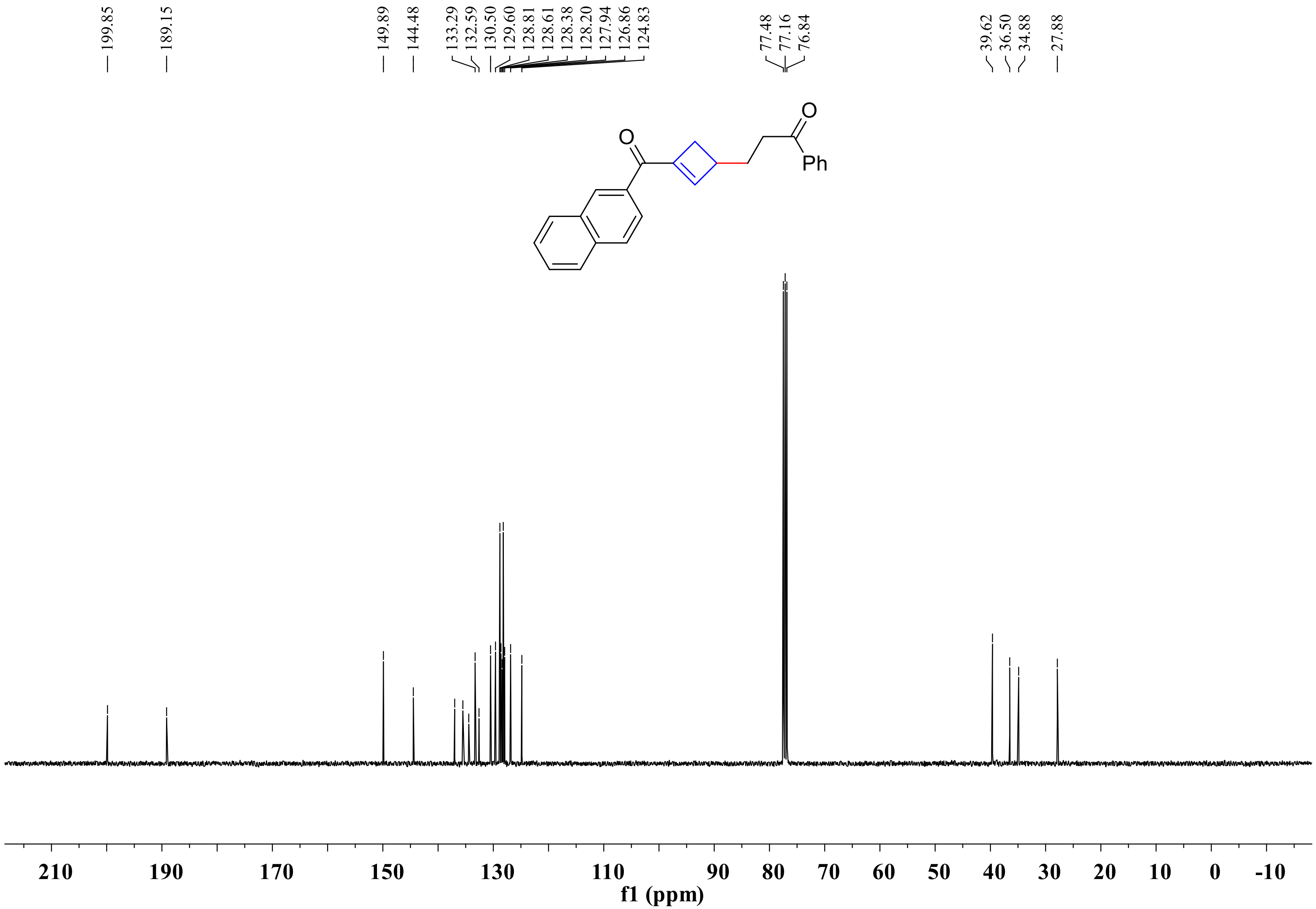

Supplementary Fig. 118 ^{13}C NMR (101 MHz, $CDCl_3$) of **3xa**

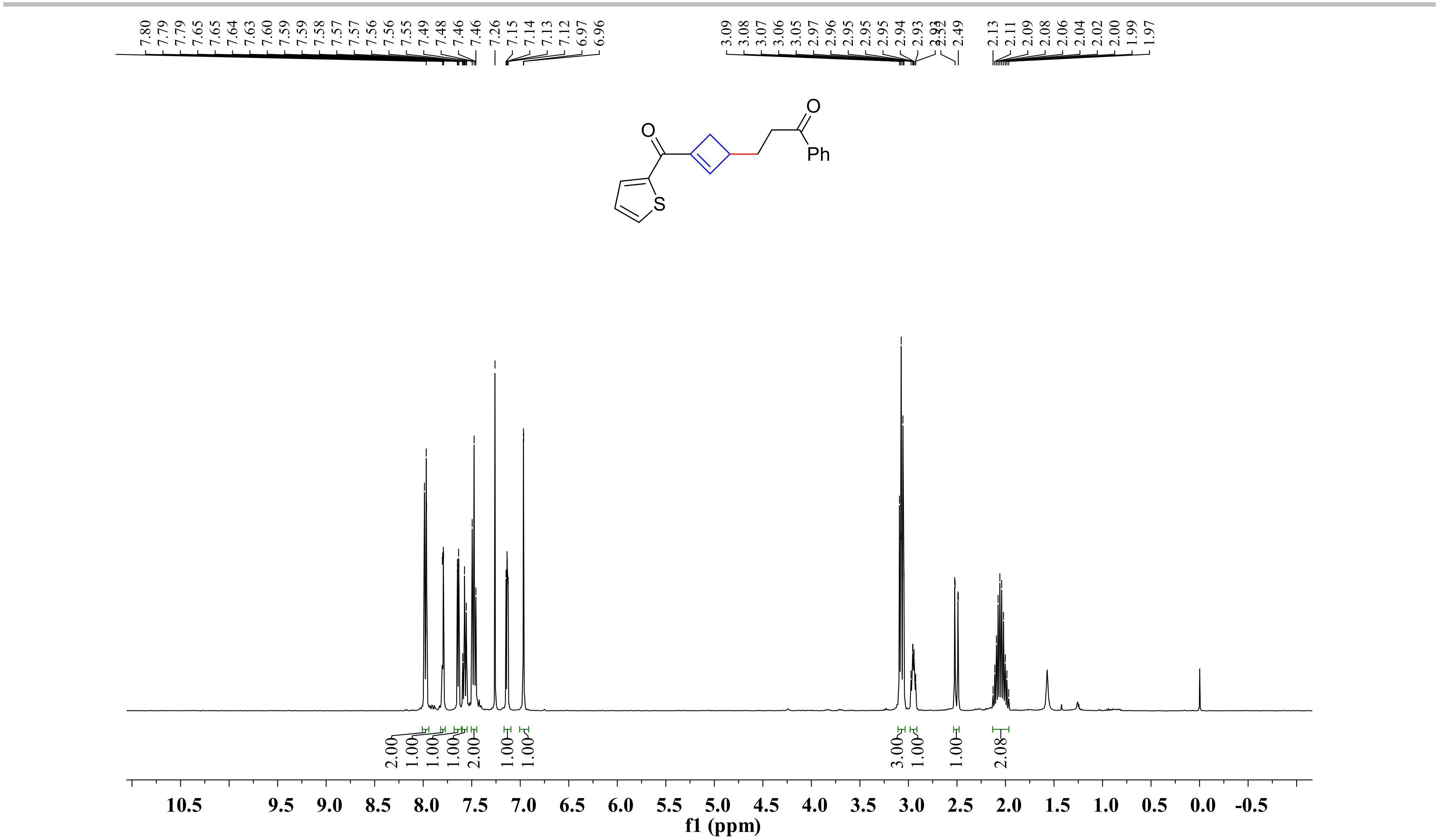

Supplementary Fig. 119 ^{1}H NMR (400 MHz, $CDCl_3$) of **3xb**

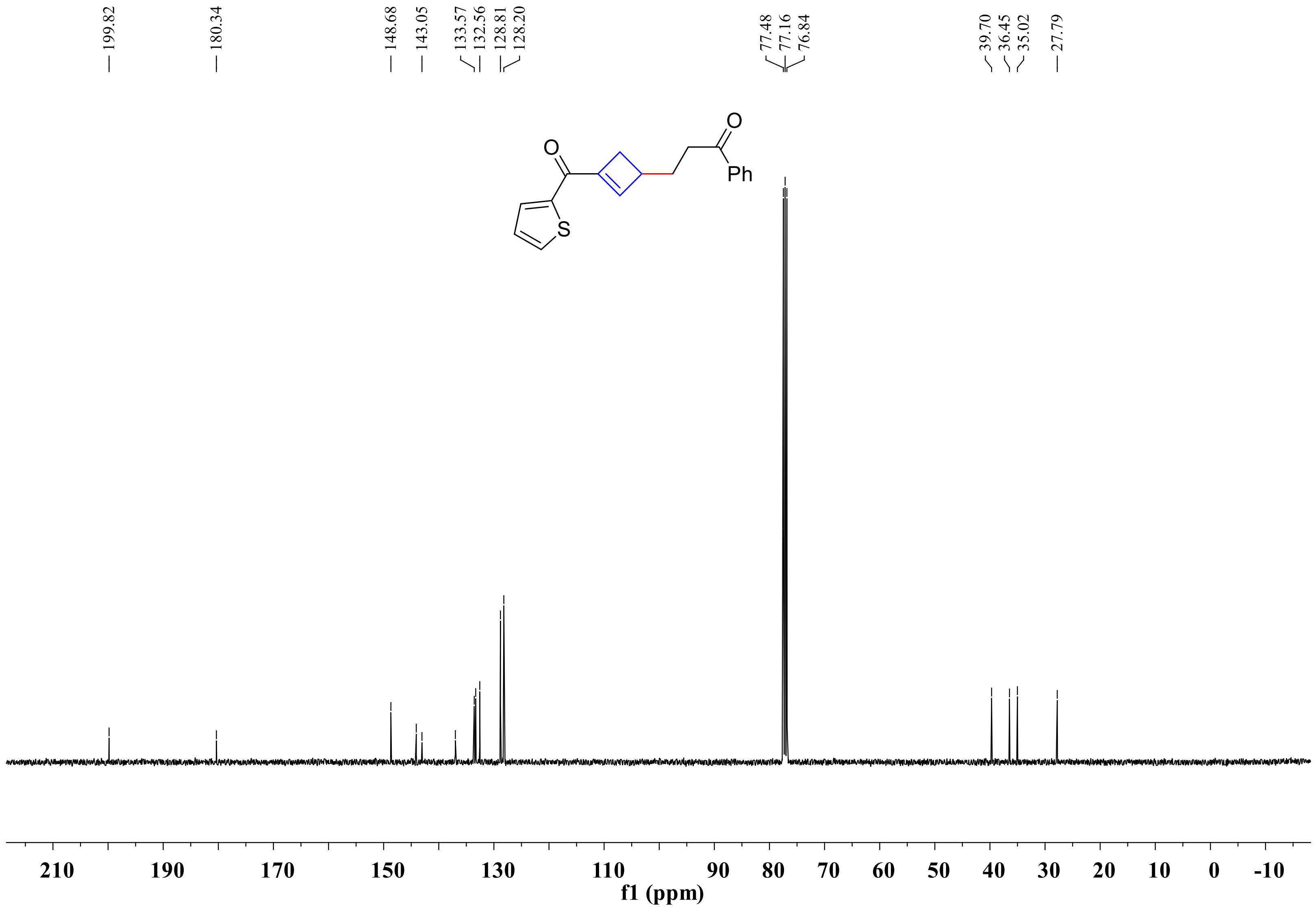

Supplementary Fig. 120 ^{13}C NMR (101 MHz, $CDCl_3$) of **3xb**

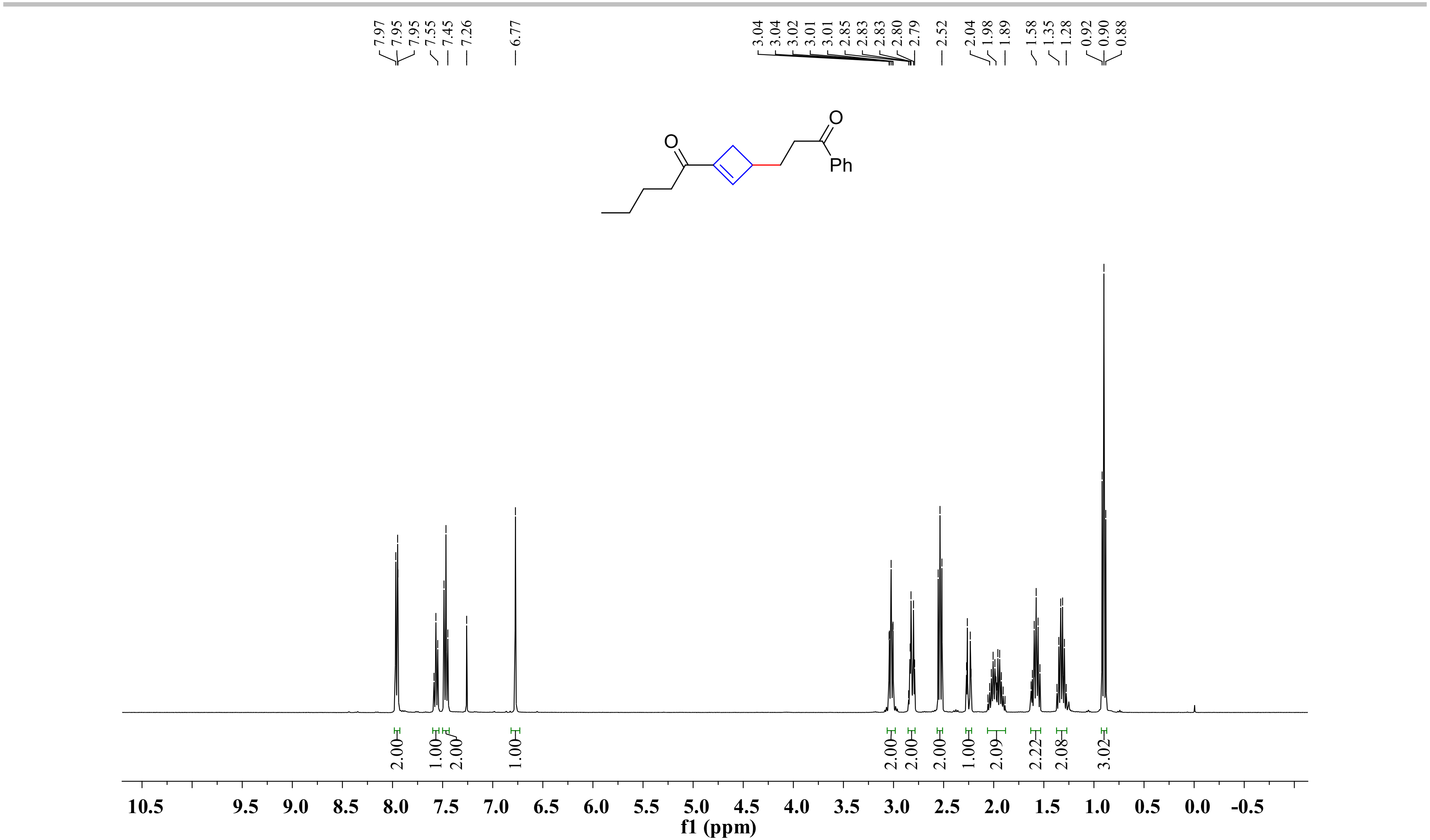

Supplementary Fig. 121 ^{1}H NMR (400 MHz, $CDCl_3$) of **3xc**

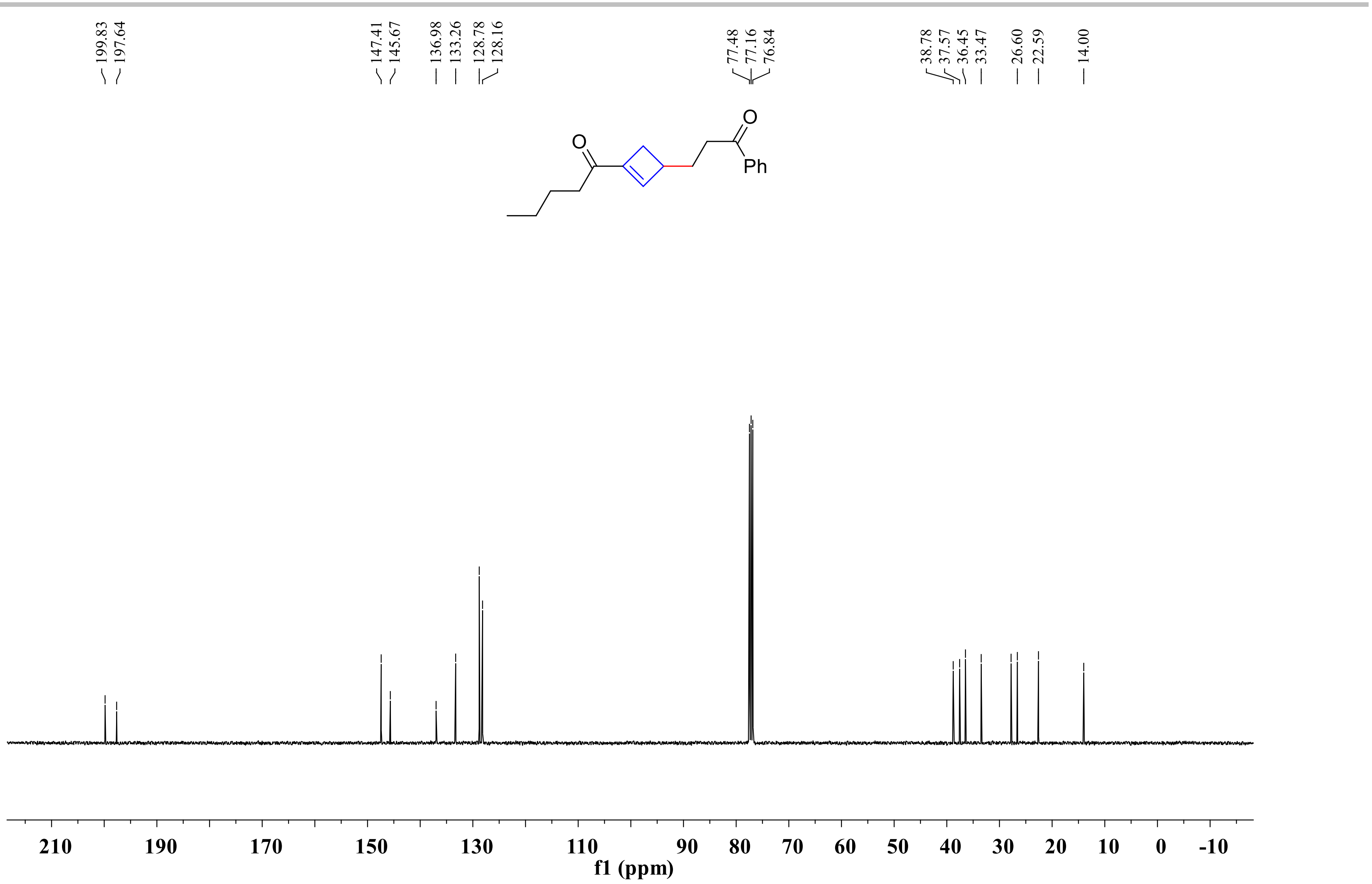

Supplementary Fig. 122 ^{13}C NMR (101 MHz, $CDCl_3$) of **3xc**

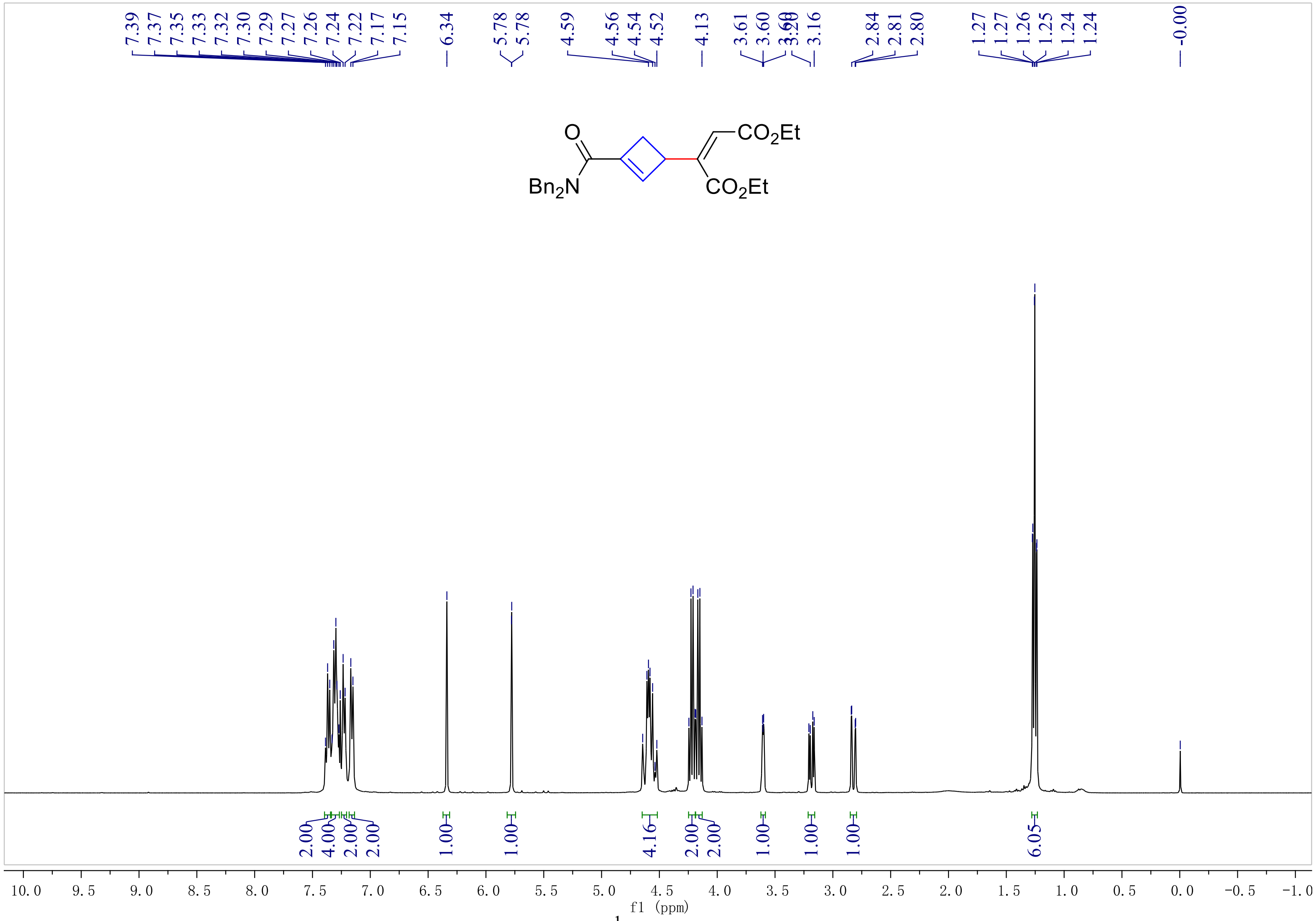

Supplementary Fig. 123 ^{1}H NMR (400 MHz, $CDCl_3$) of **3y**

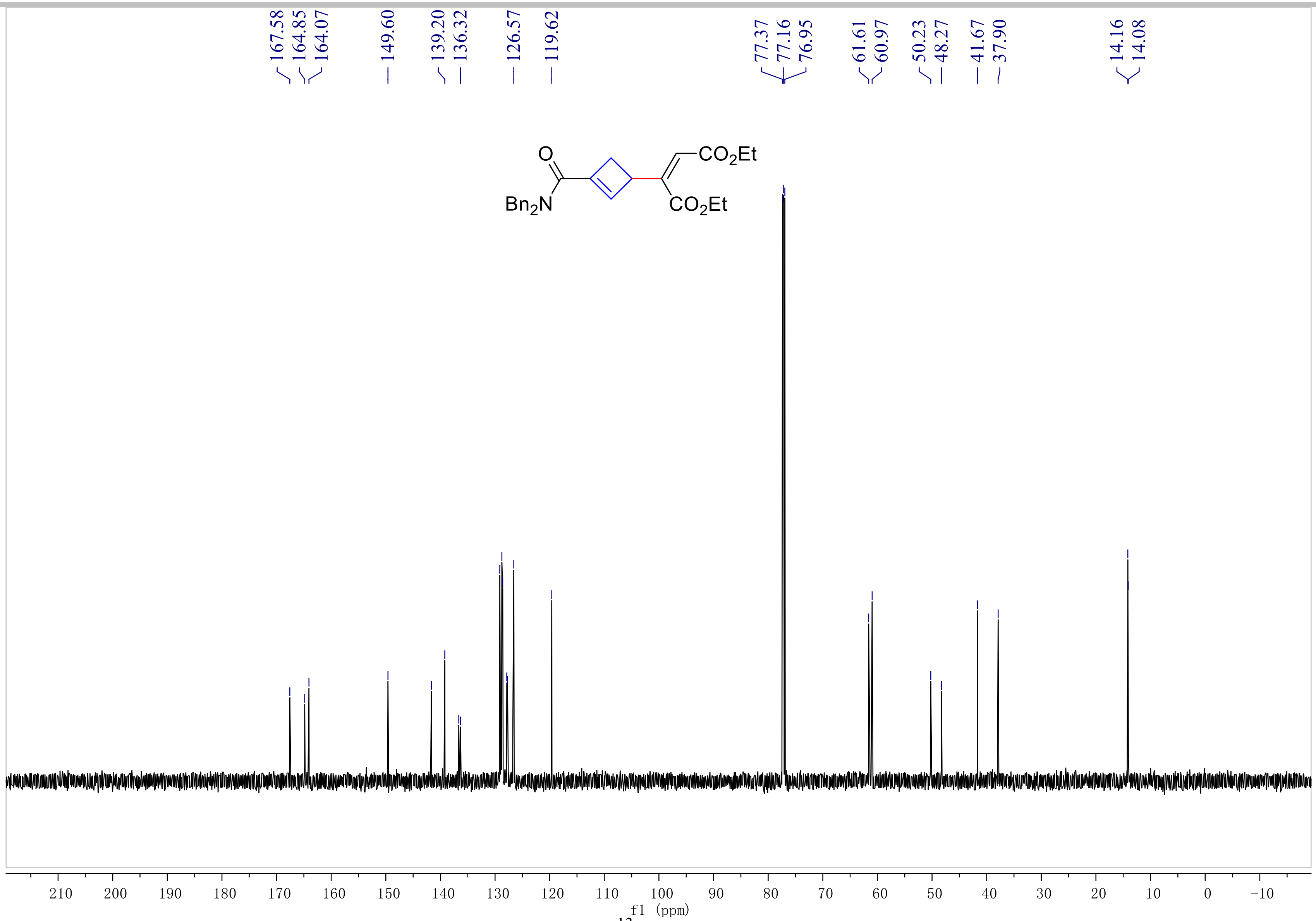

Supplementary Fig. 124 ^{13}C NMR (151 MHz, $CDCl_3$) of **3y**

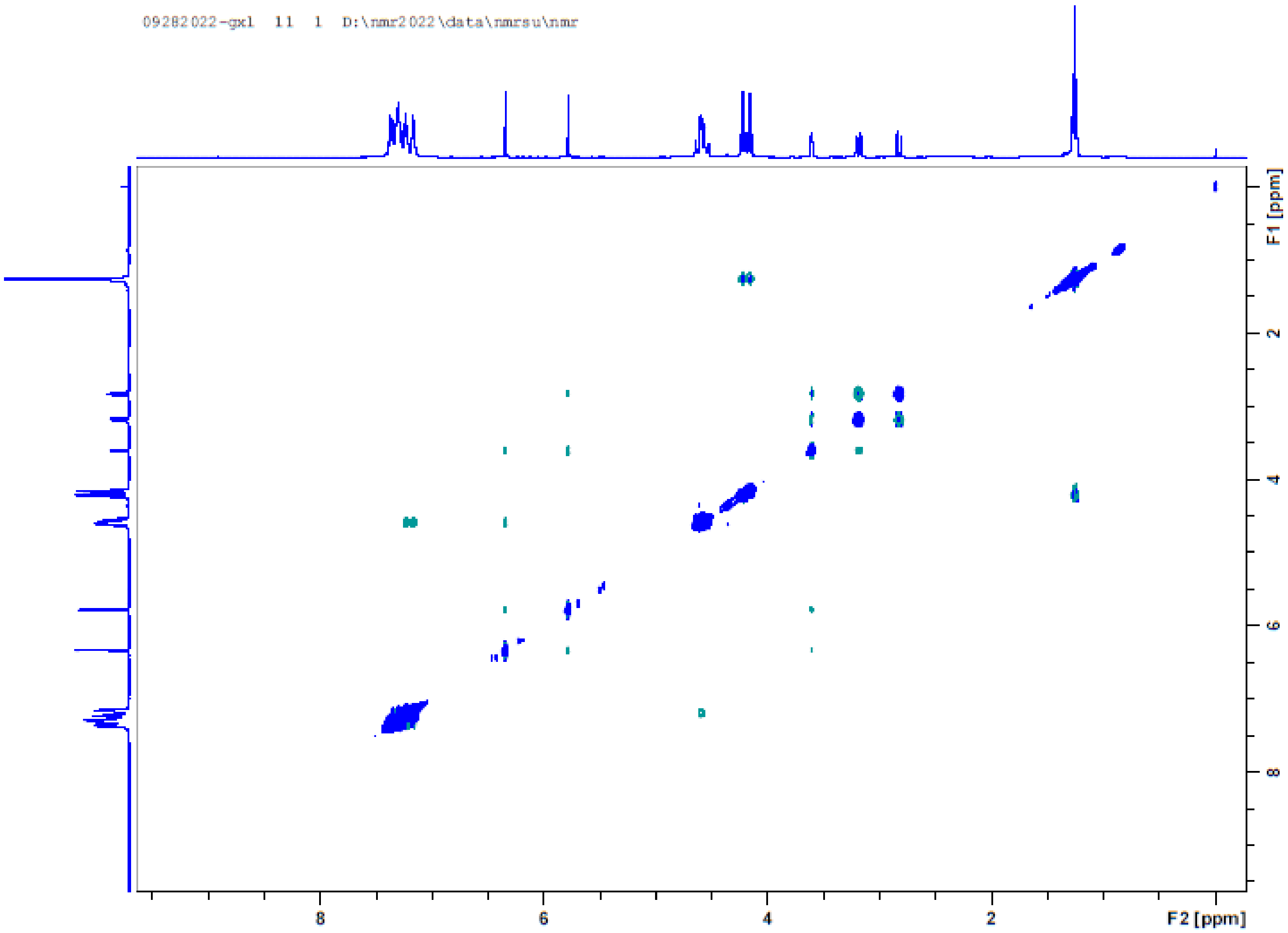

Supplementary Fig. 125 NOESY - 2D ^{1}H NMR of **3y** (400 MHz, $CDCl_3$)

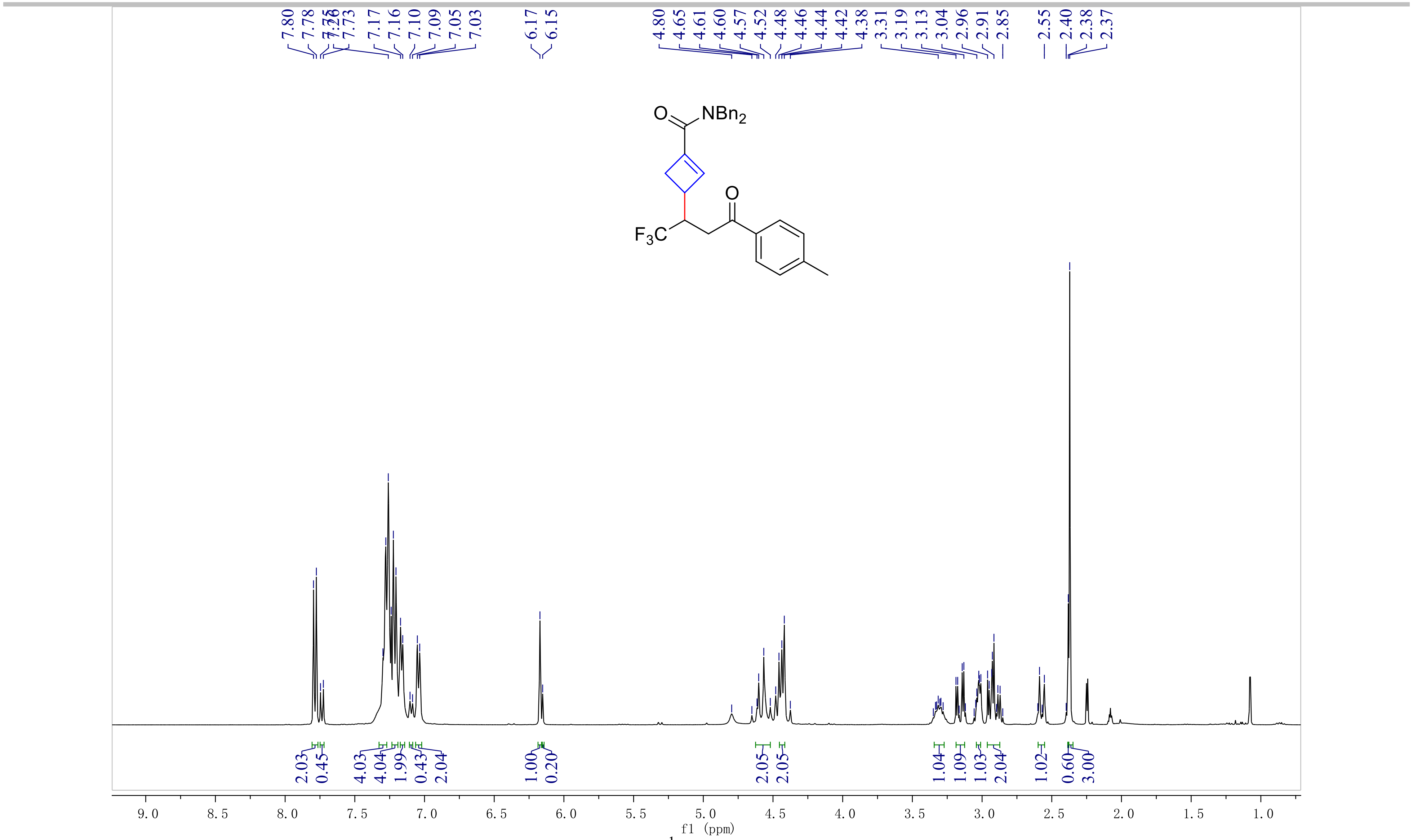

Supplementary Fig. 126 ^{1}H NMR (400 MHz, $CDCl_3$) of **4a**

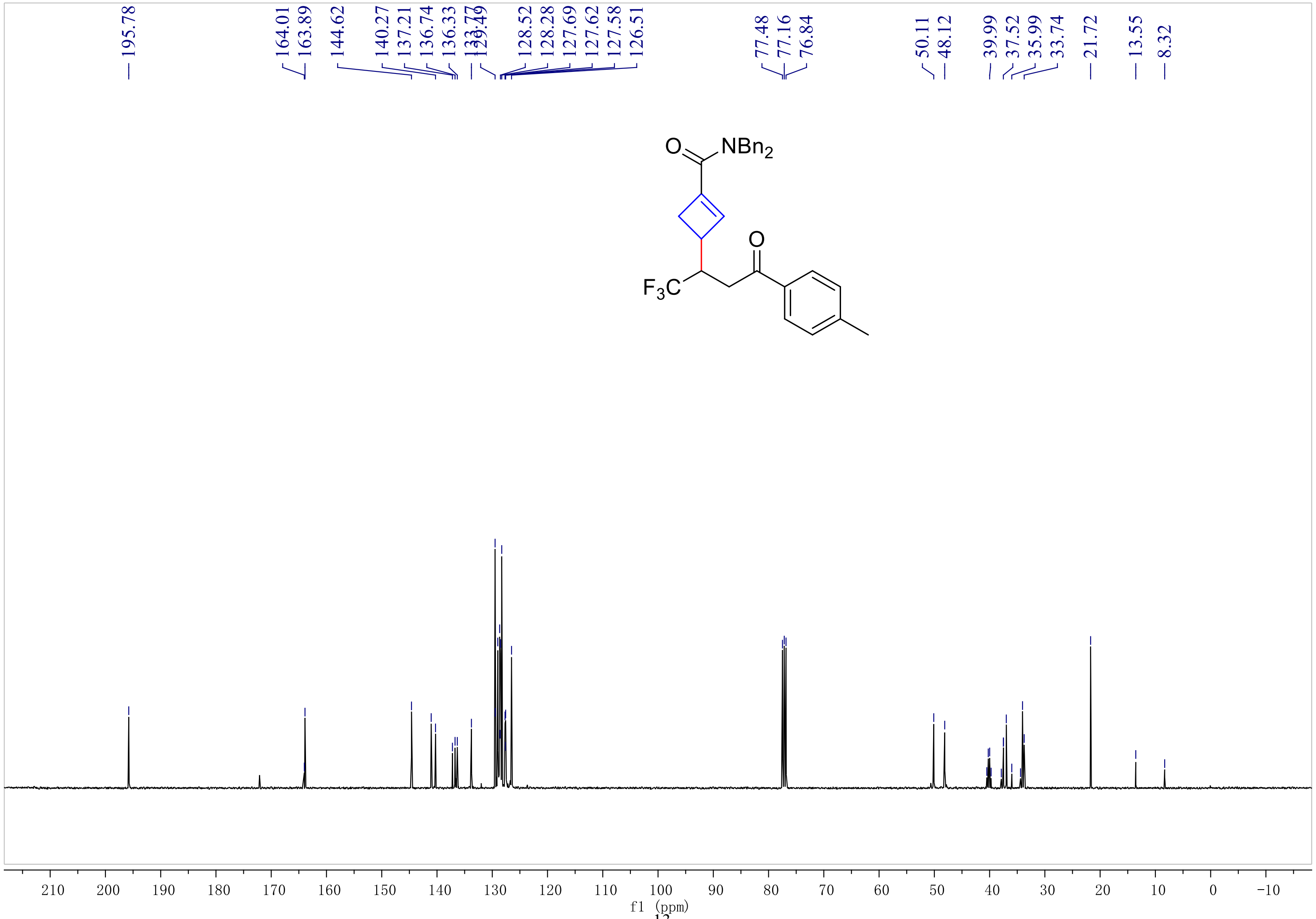

Supplementary Fig. 127 ^{13}C NMR (101 MHz, $CDCl_3$) of **4a**

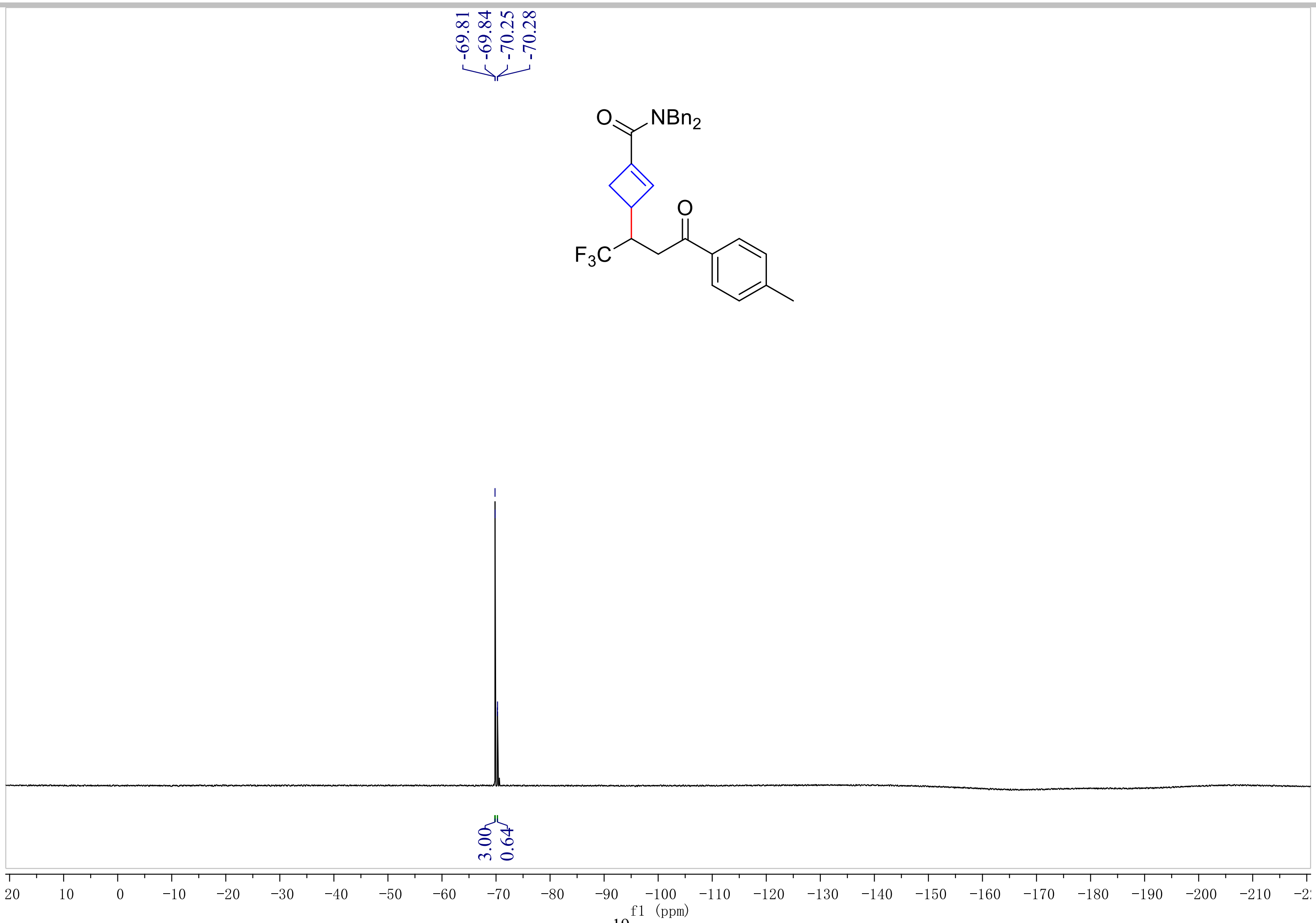

Supplementary Fig. 128 ^{19}F NMR (377 MHz, $CDCl_3$) of **4a**

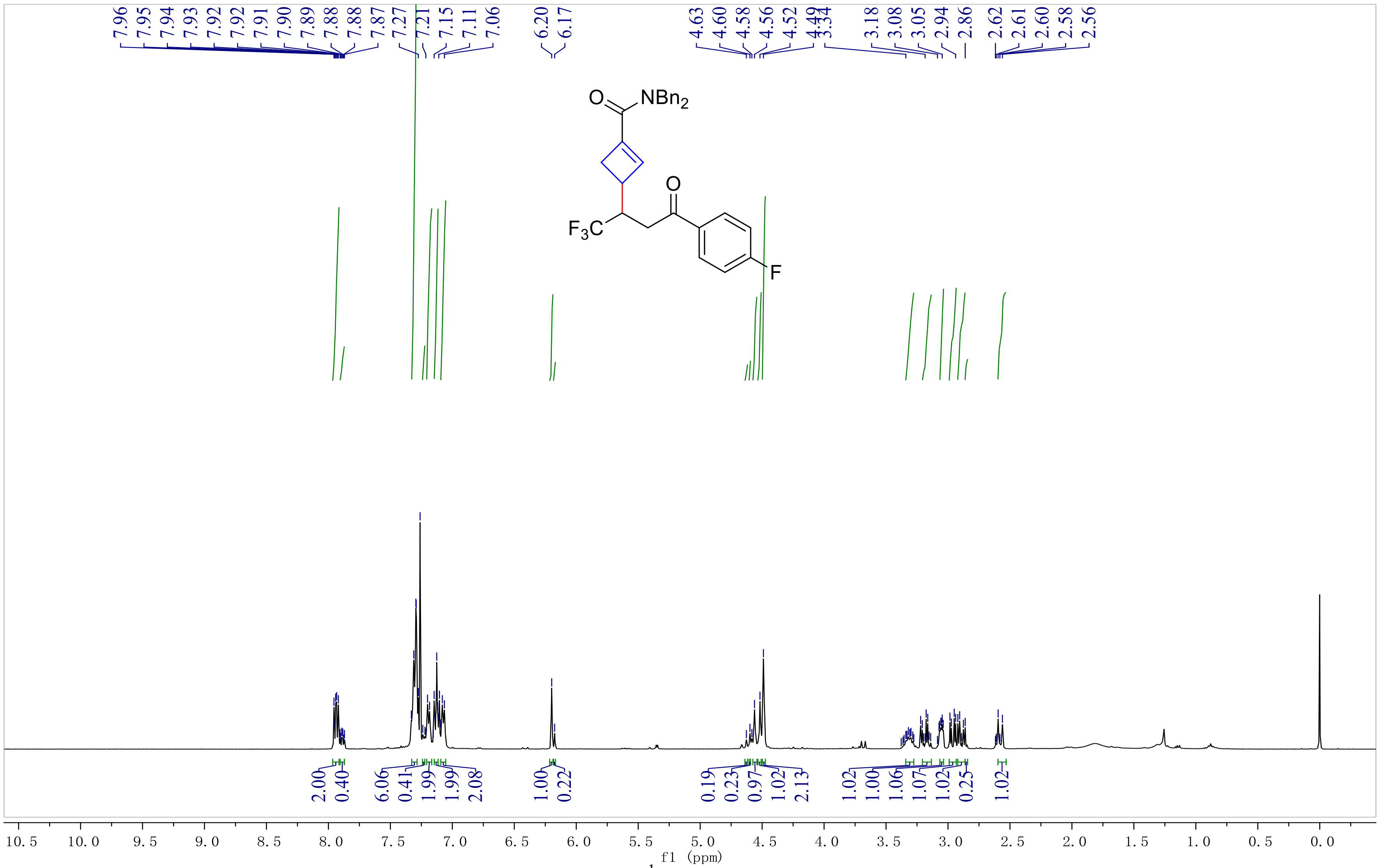

Supplementary Fig. 129 ^{1}H NMR (400 MHz, $CDCl_3$) of **4b**

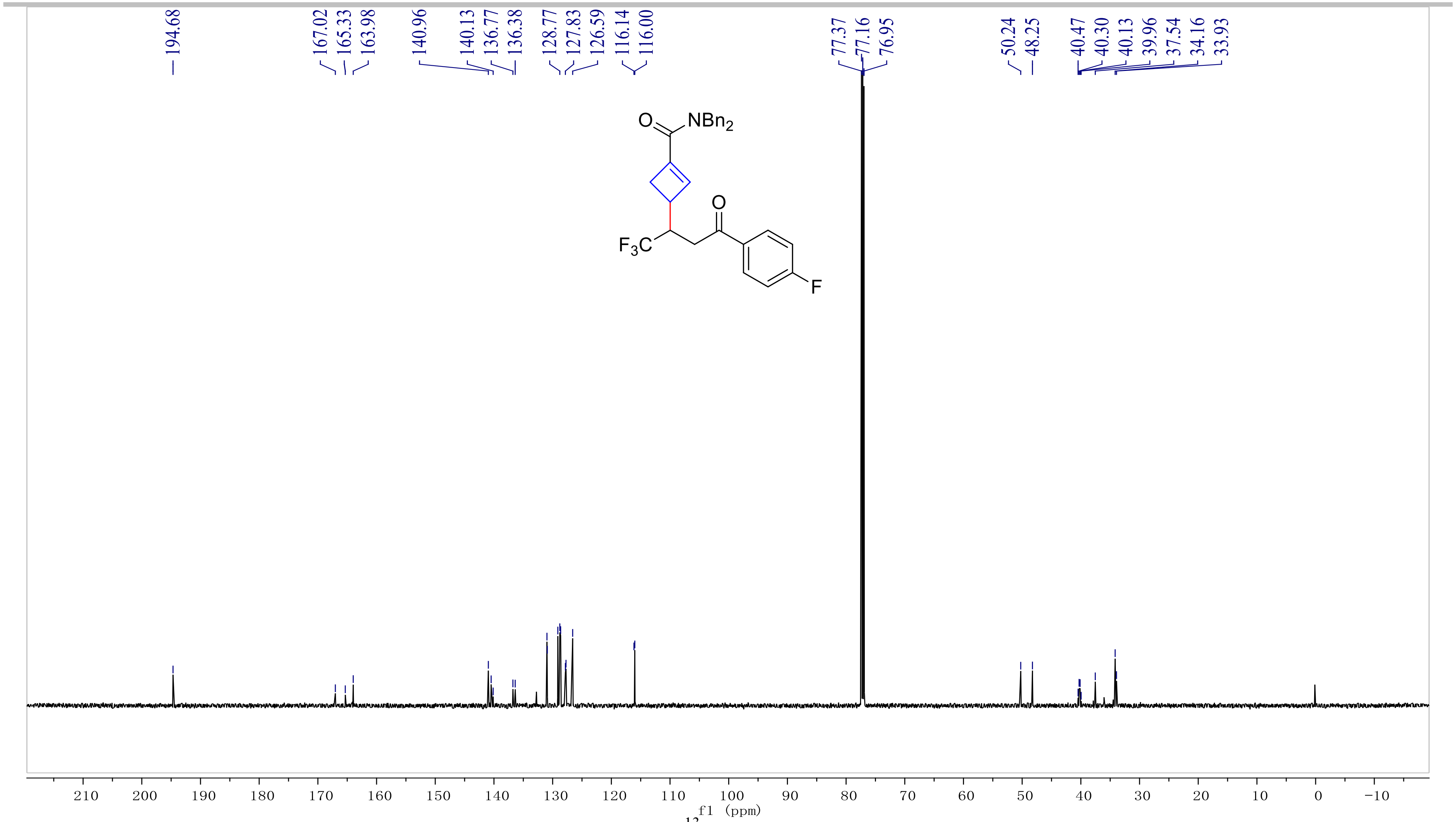

Supplementary Fig. 130 ^{13}C NMR (151 MHz, $CDCl_3$) of **4b**

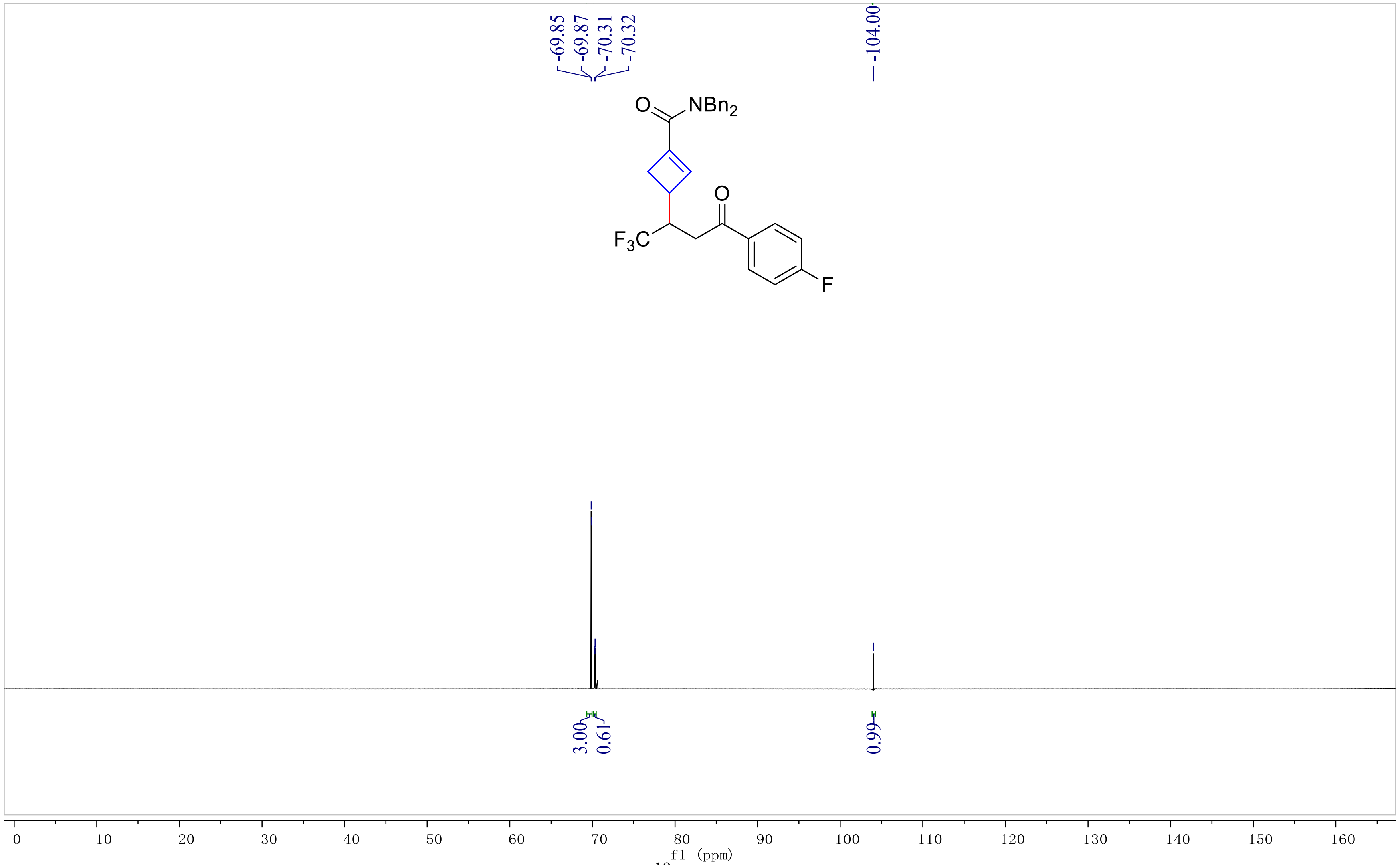

Supplementary Fig. 131 ^{19}F NMR (565 MHz, $CDCl_3$) of **4b**

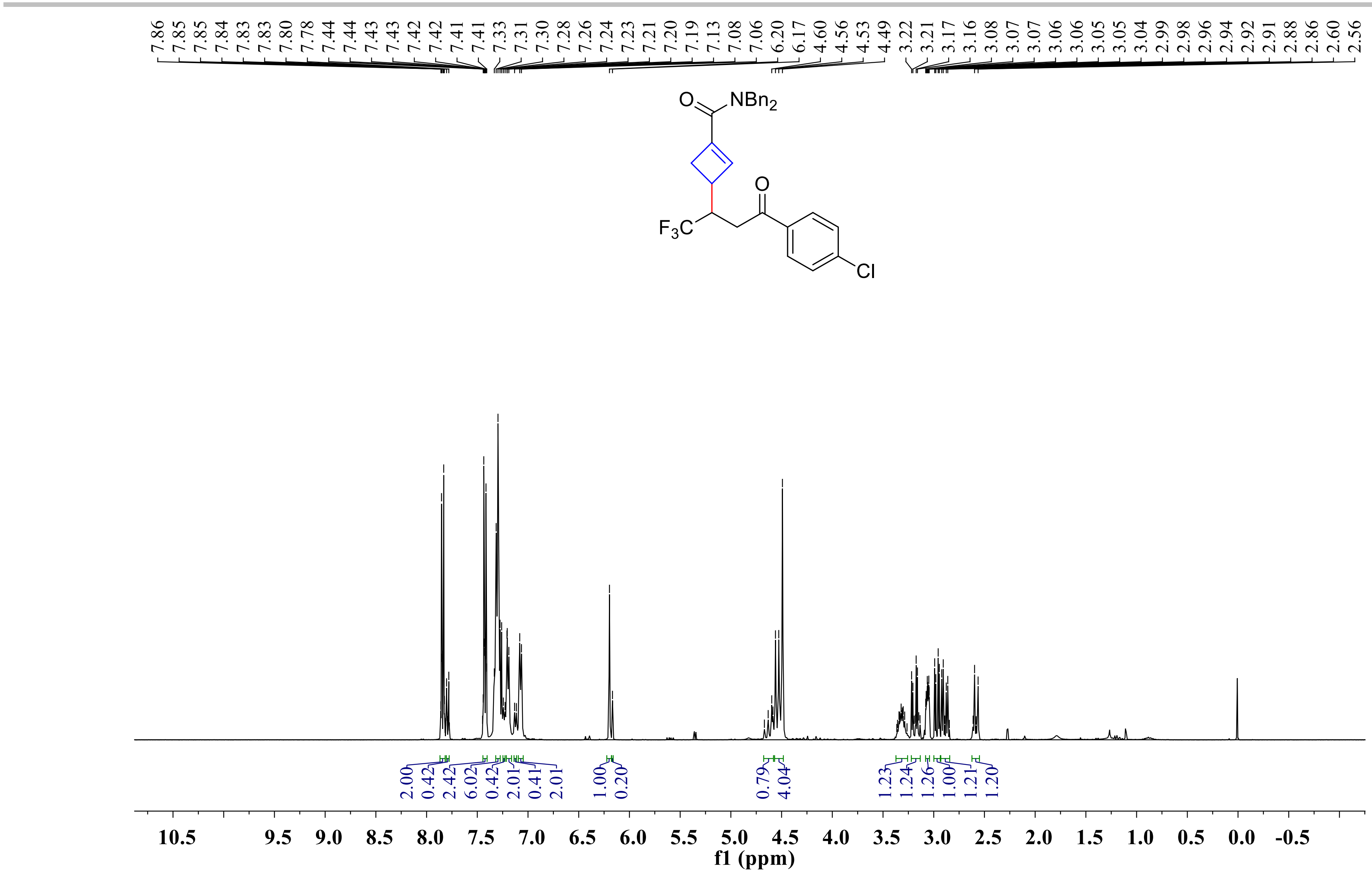

Supplementary Fig. 132 ^{1}H NMR (400 MHz, $CDCl_3$) of **4c**

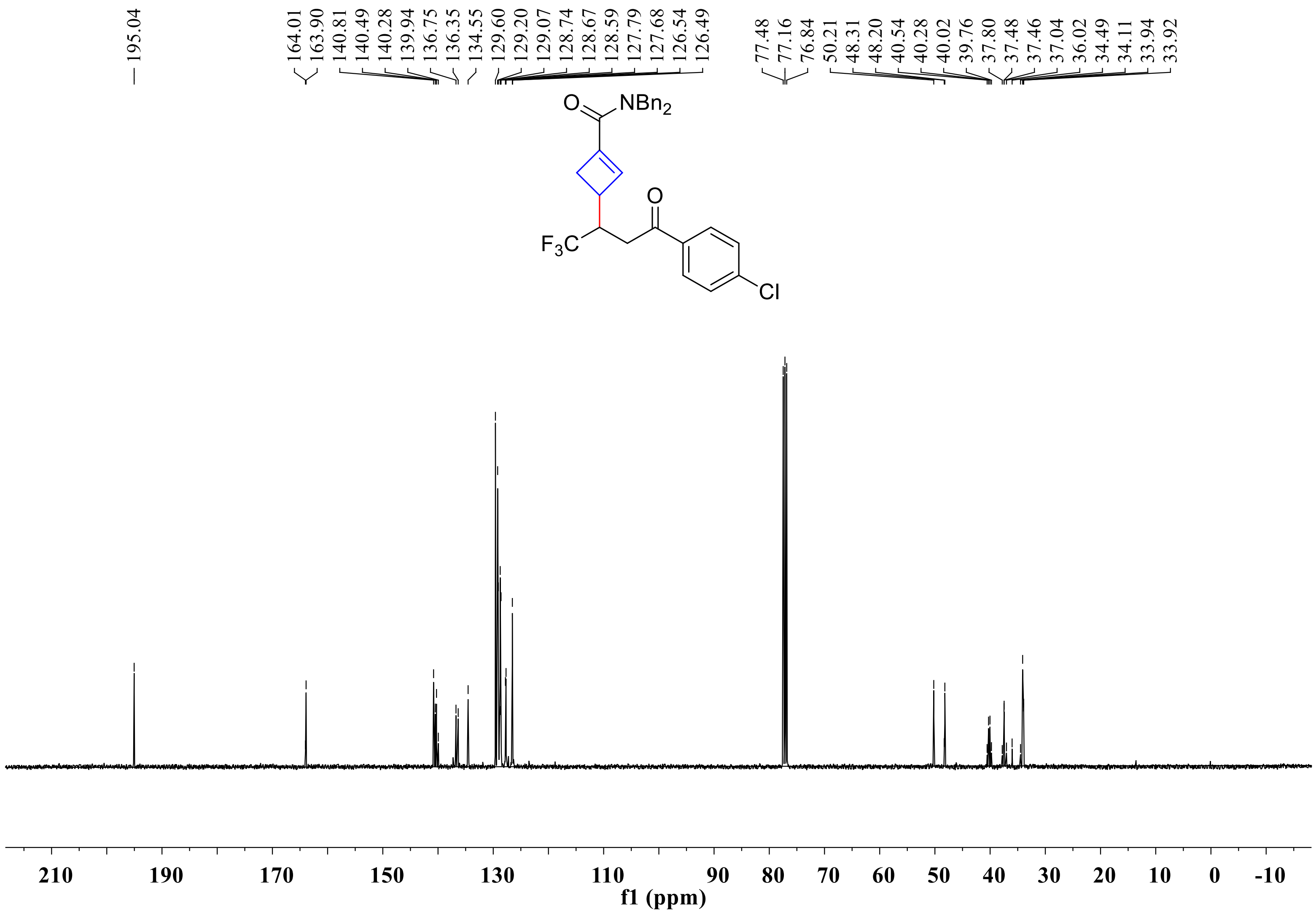

Supplementary Fig. 133 ^{13}C NMR (151 MHz, $CDCl_3$) of **4c**

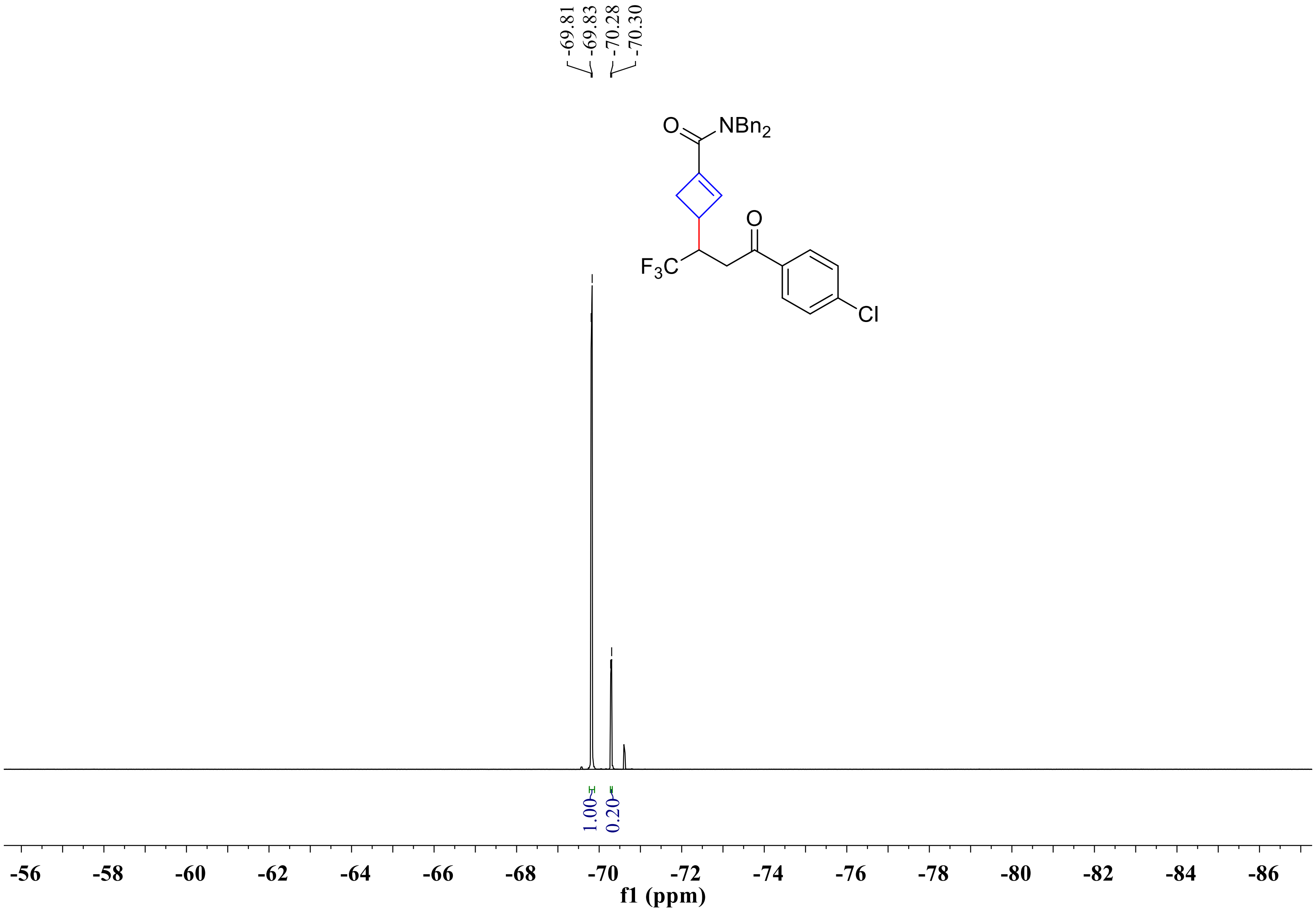

Supplementary Fig. 134 ^{19}F NMR (565 MHz, $CDCl_3$) of **4c**

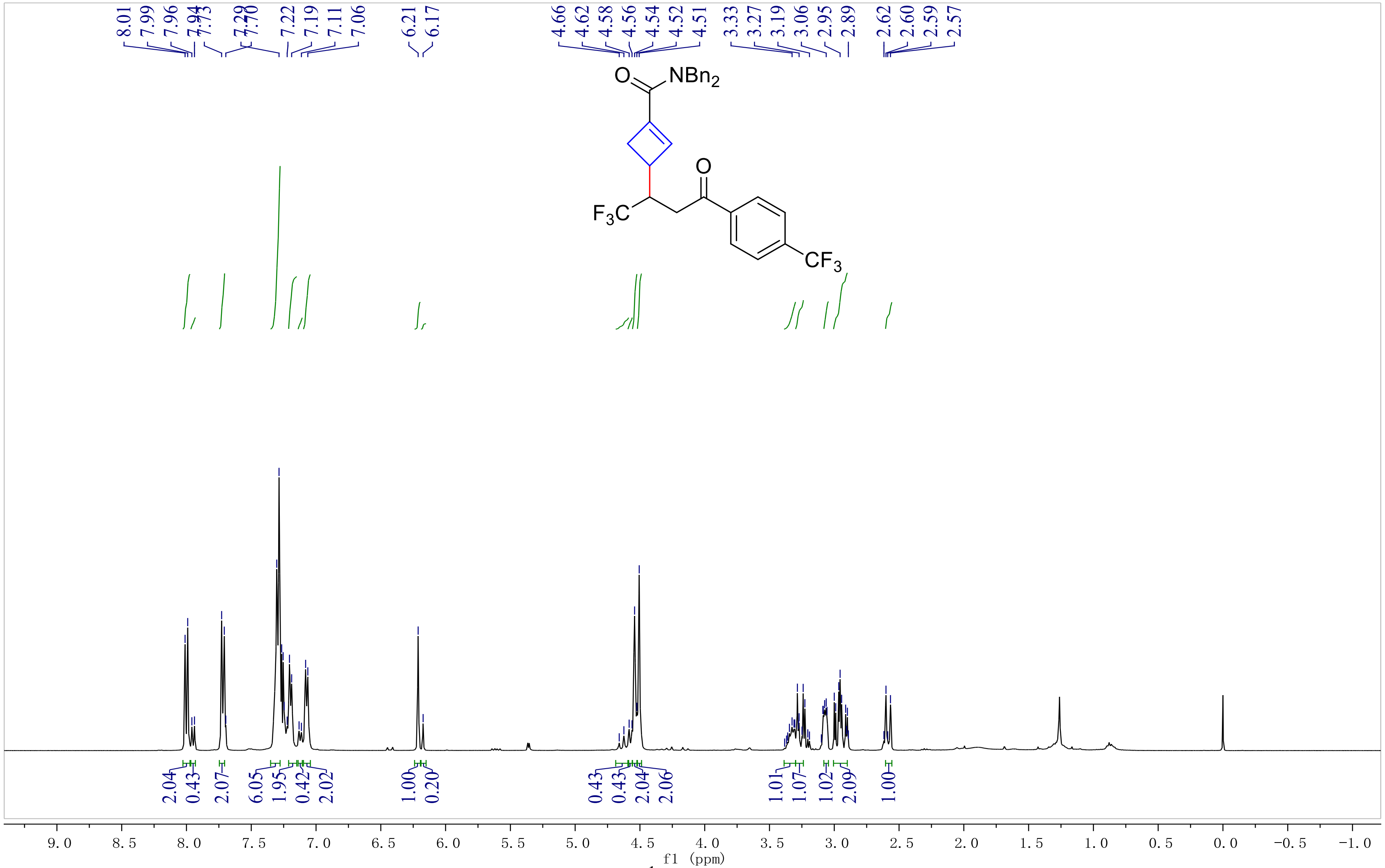

Supplementary Fig. 135 ^{1}H NMR (400 MHz, $CDCl_3$) of **4d**

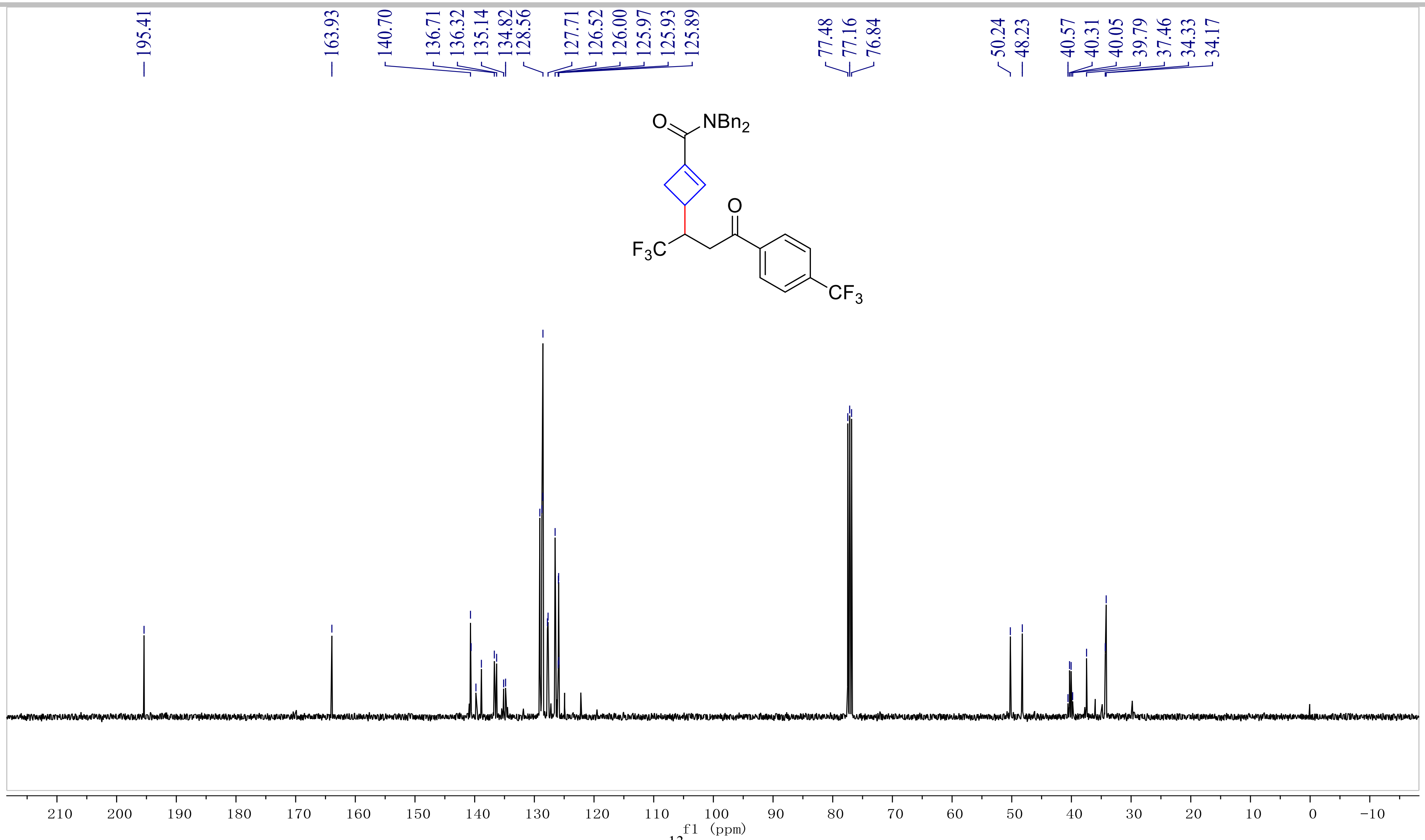

Supplementary Fig. 136 ^{13}C NMR (101 MHz, $CDCl_3$) of **4d**

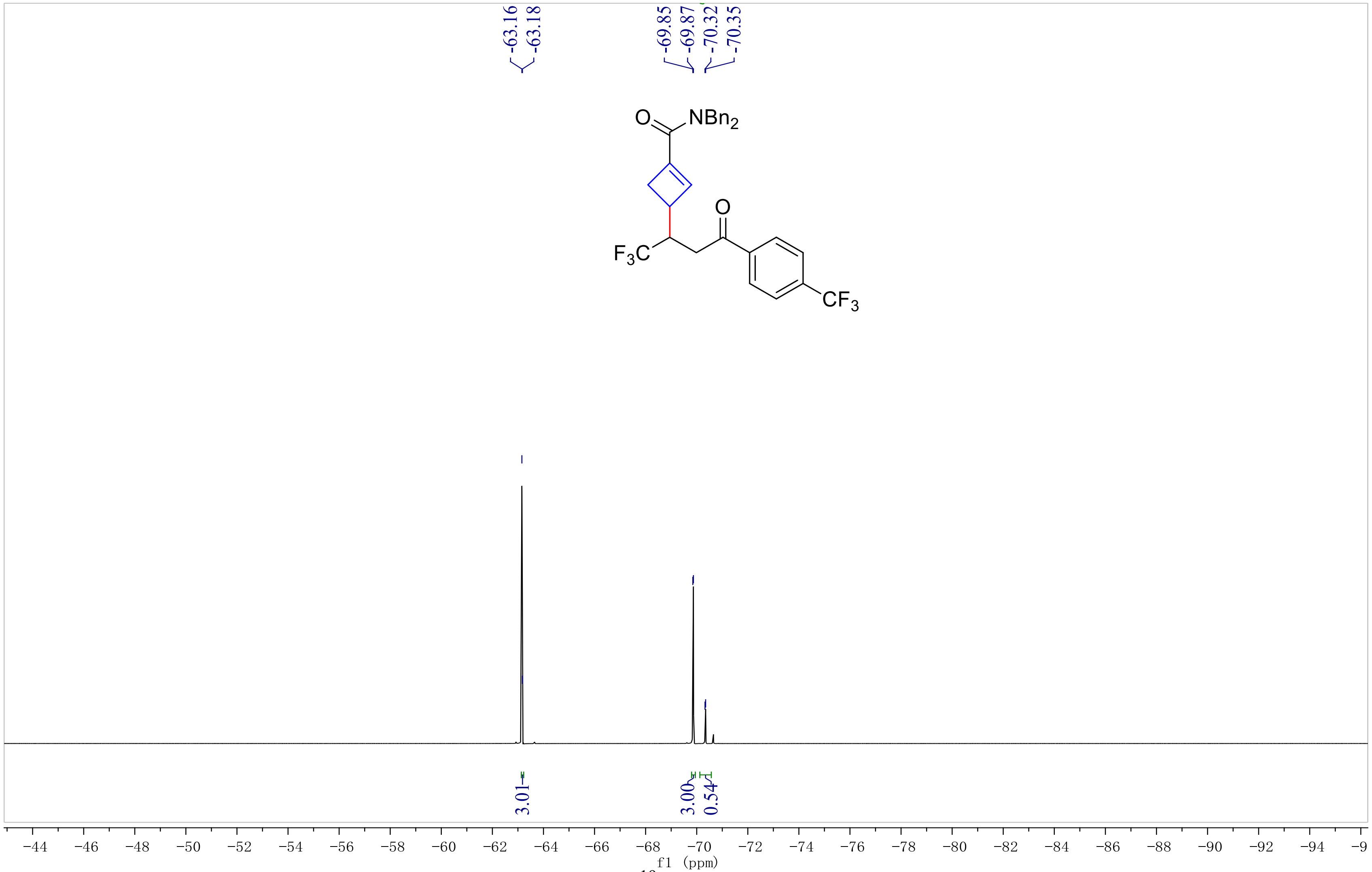

Supplementary Fig. 137 ^{19}F NMR (377 MHz, $CDCl_3$) of **4d**

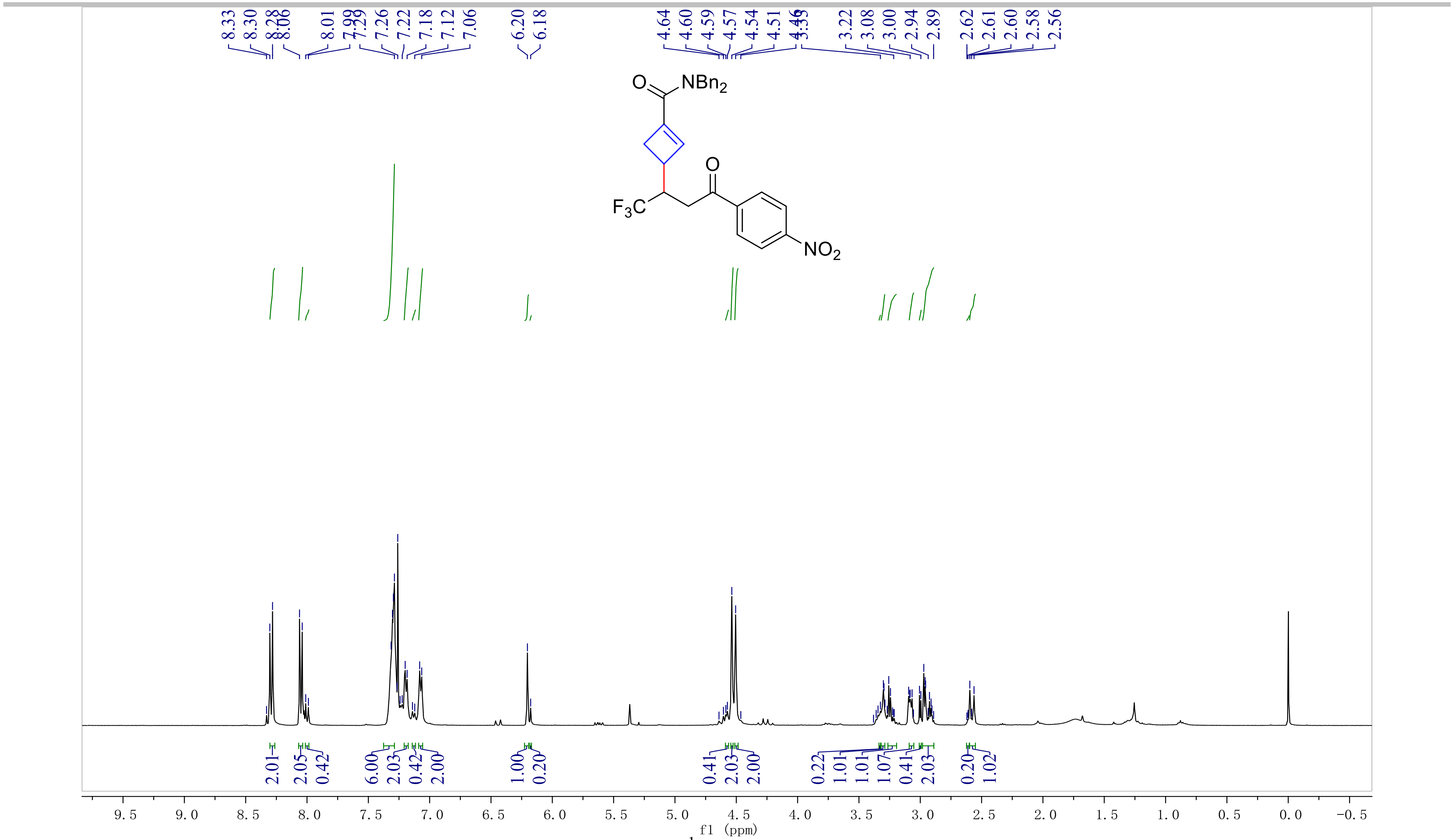

Supplementary Fig. 138 ^{1}H NMR (400 MHz, $CDCl_3$) of **4e**

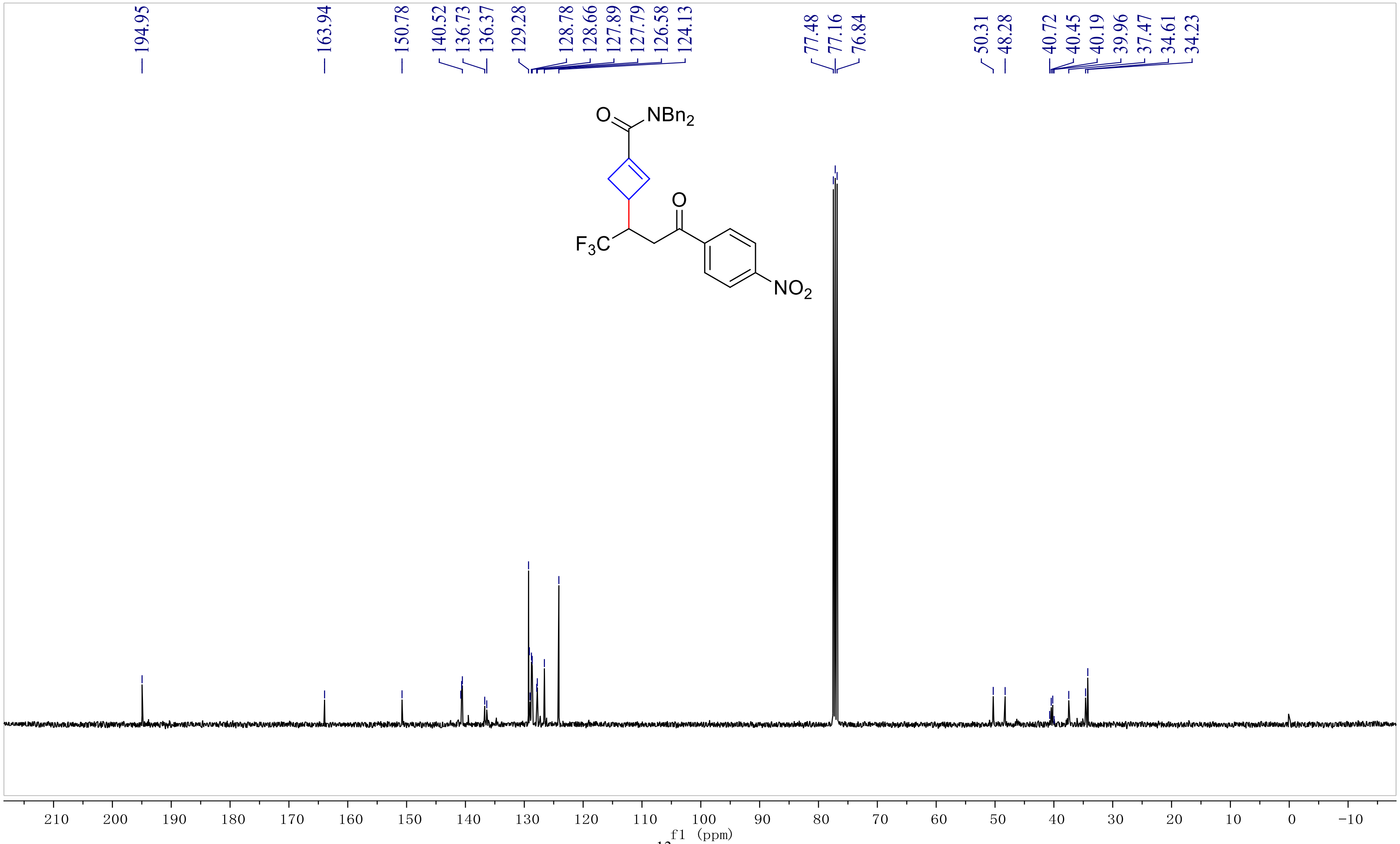

Supplementary Fig. 139 ^{13}C NMR (101 MHz, $CDCl_3$) of **4e**

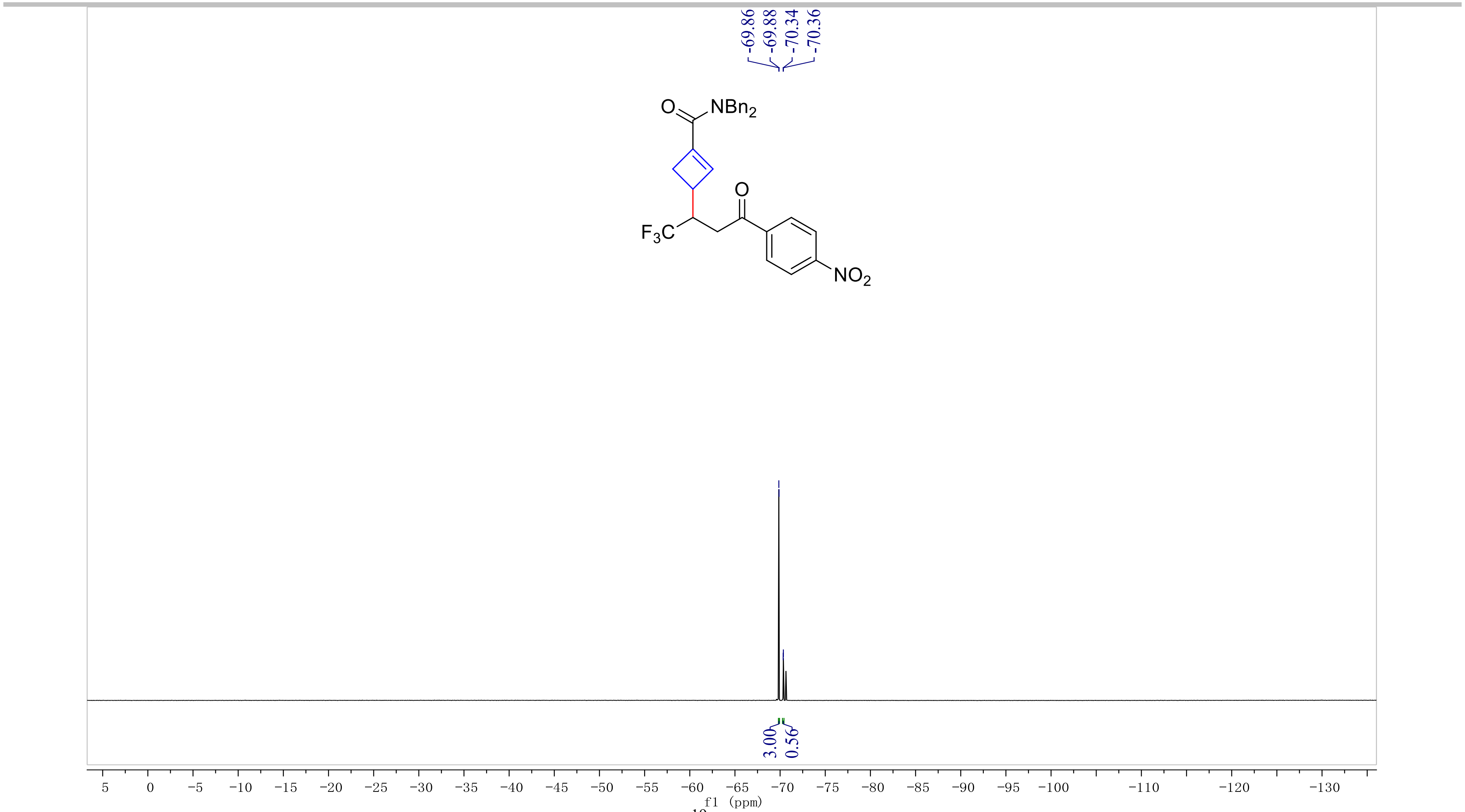

Supplementary Fig. 140 ^{19}F NMR (377 MHz, $CDCl_3$) of **4e**

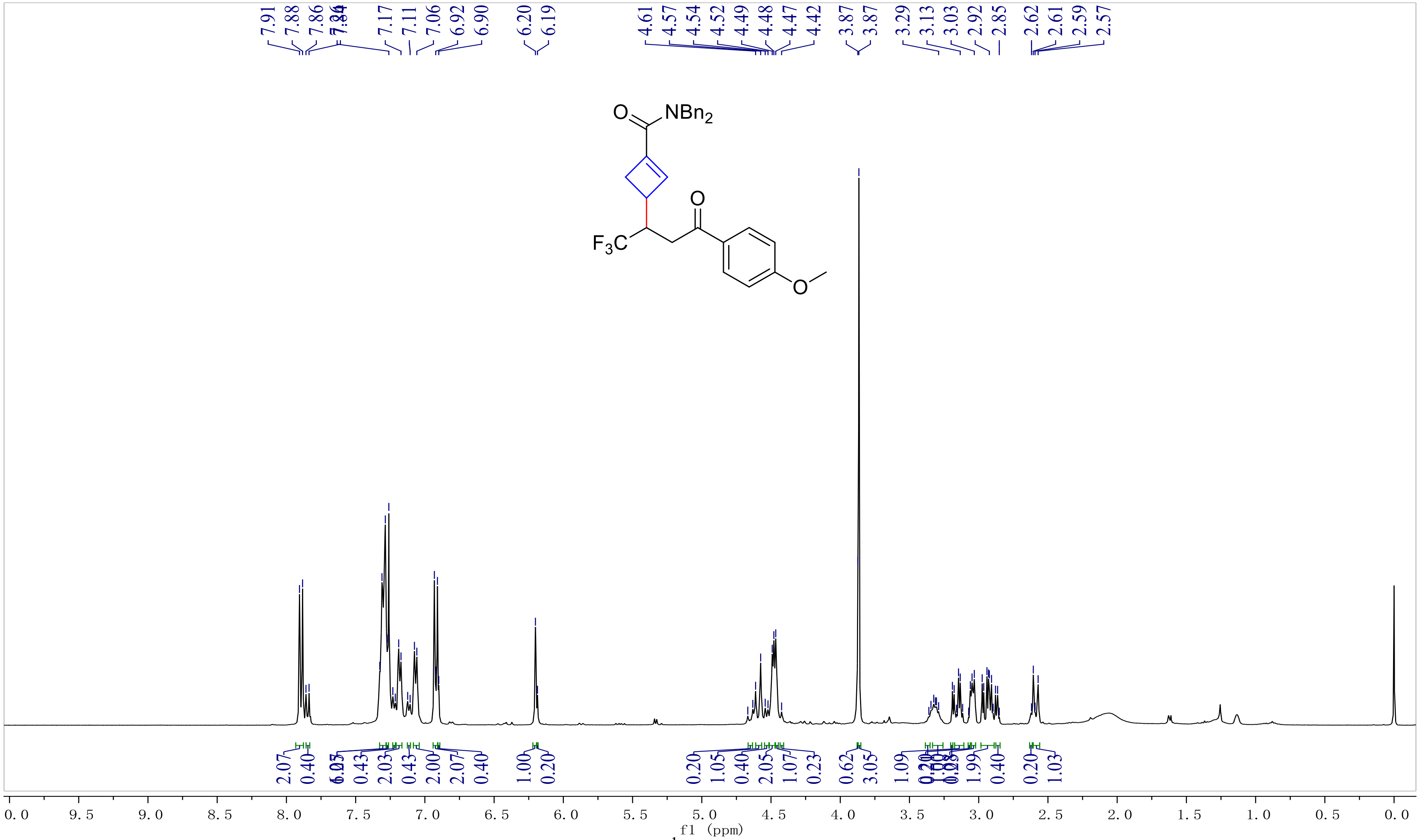

Supplementary Fig. 141 ^{1}H NMR (400 MHz, $CDCl_3$) of **4f**

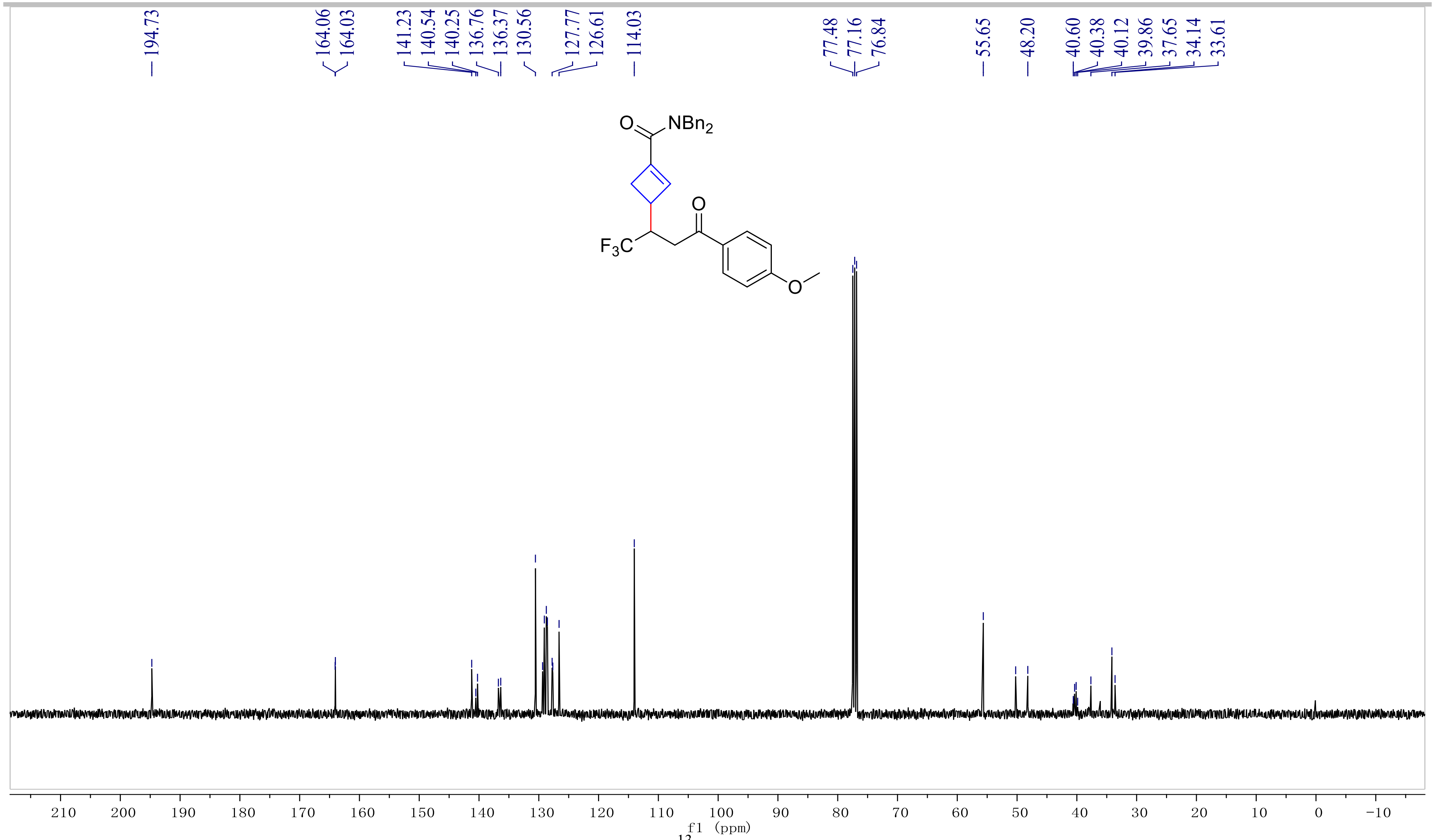

Supplementary Fig. 142 ^{13}C NMR (101 MHz, $CDCl_3$) of **4f**

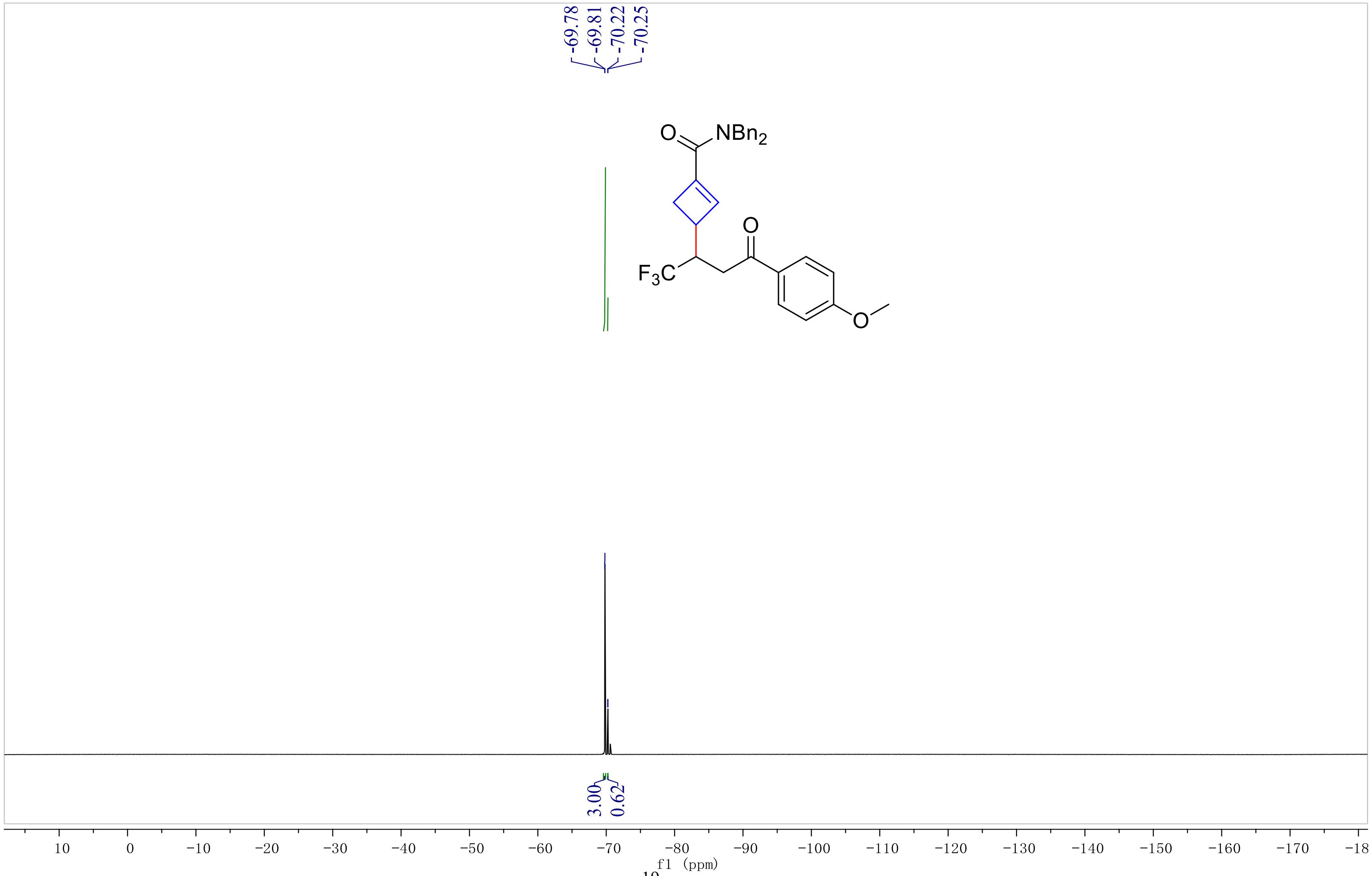

Supplementary Fig. 143 ^{19}F NMR (377 MHz, $CDCl_3$) of **4f**

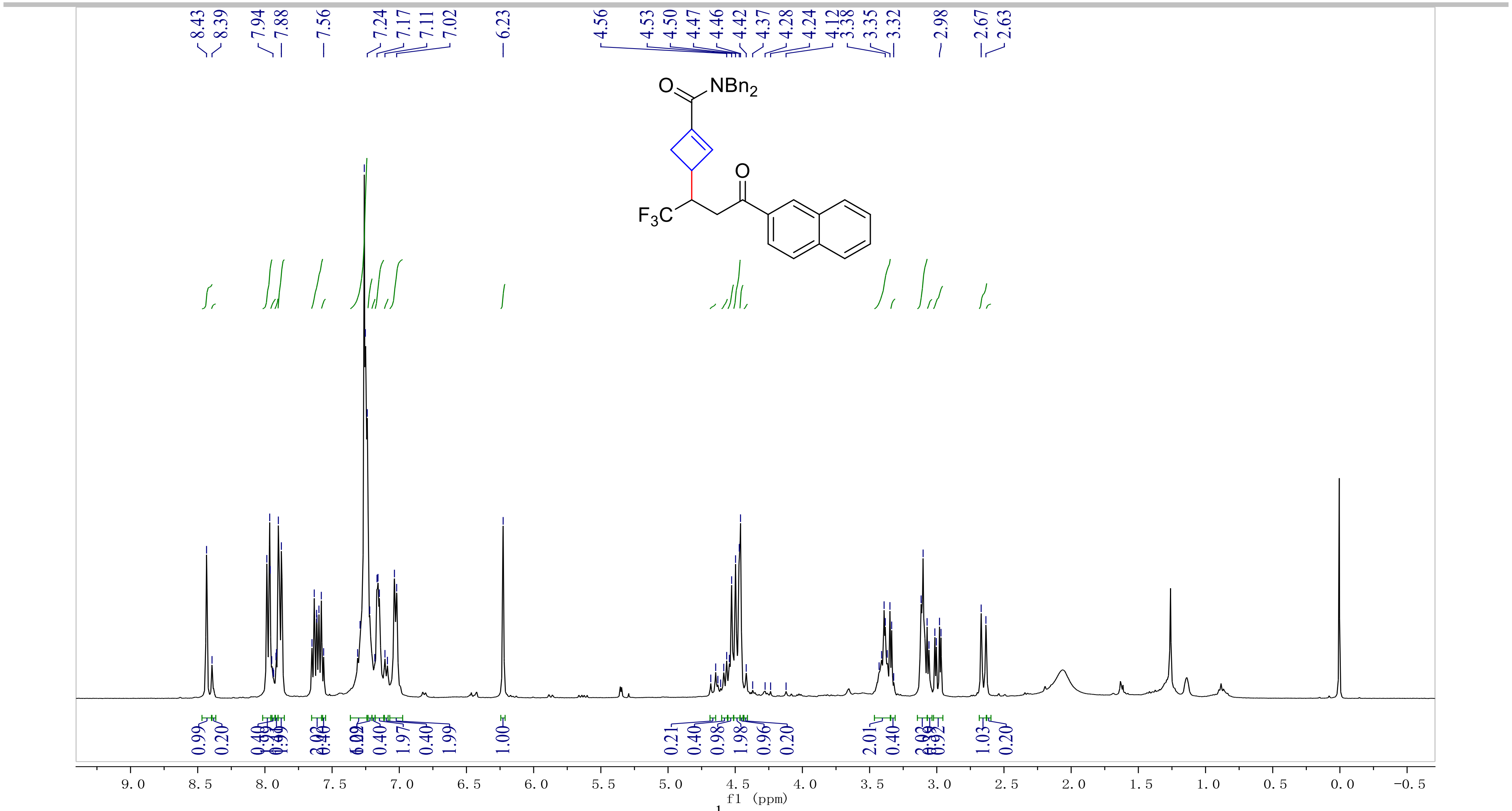

Supplementary Fig. 144 ^{1}H NMR (400 MHz, $CDCl_3$) of **4g**

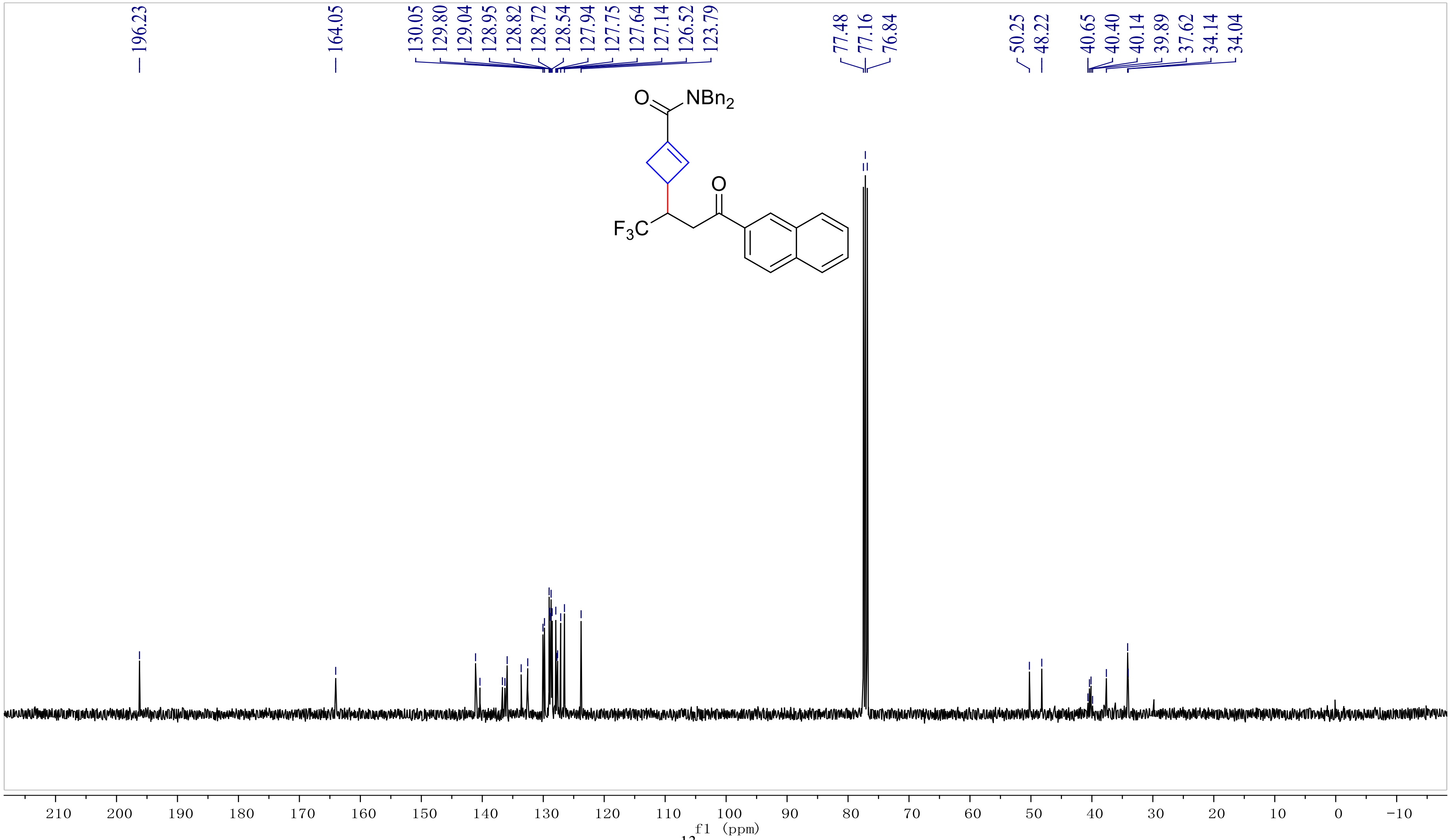

Supplementary Fig. 145 ^{13}C NMR (101 MHz, $CDCl_3$) of **4g**

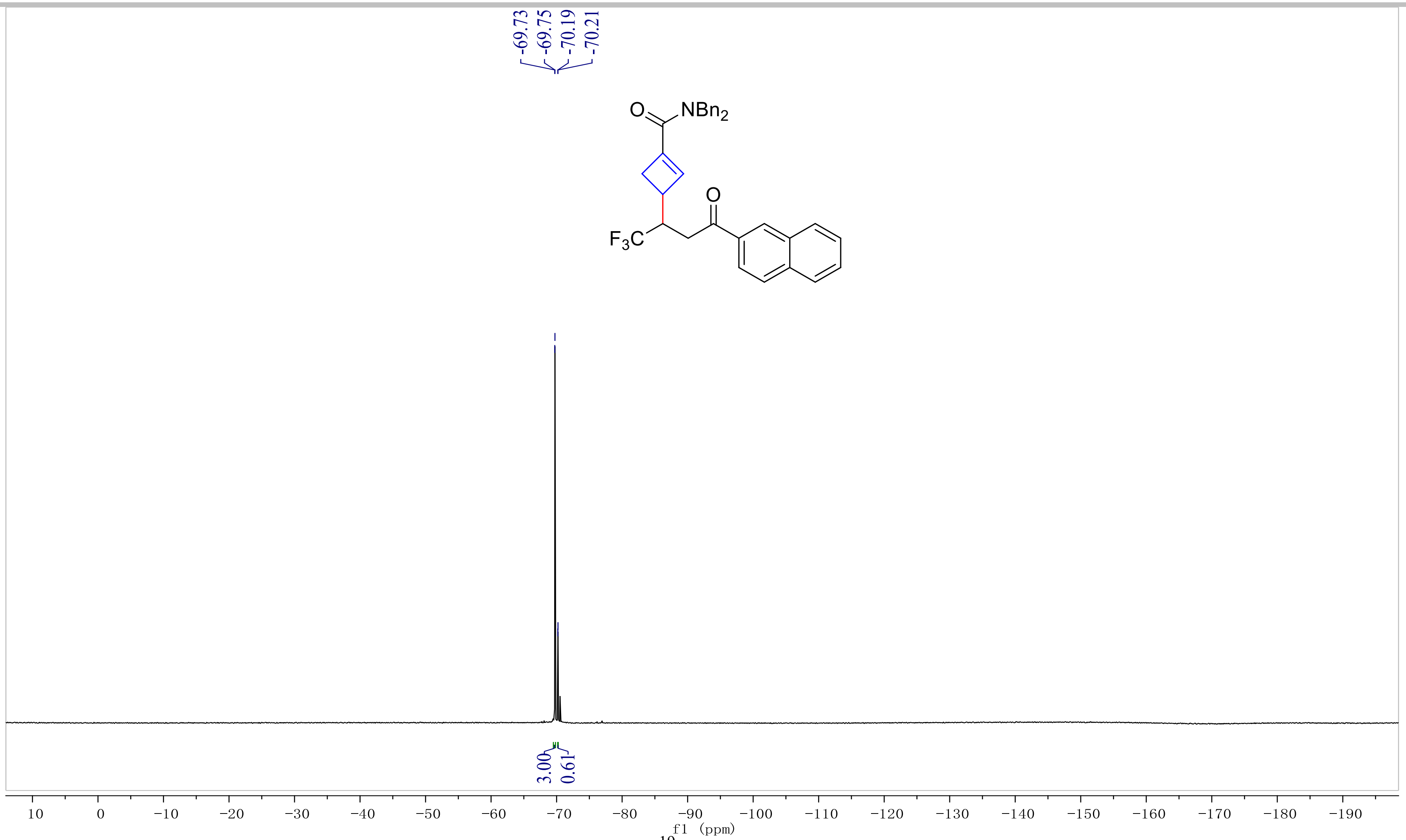

Supplementary Fig. 146 ^{19}F NMR (377 MHz, $CDCl_3$) of **4g**

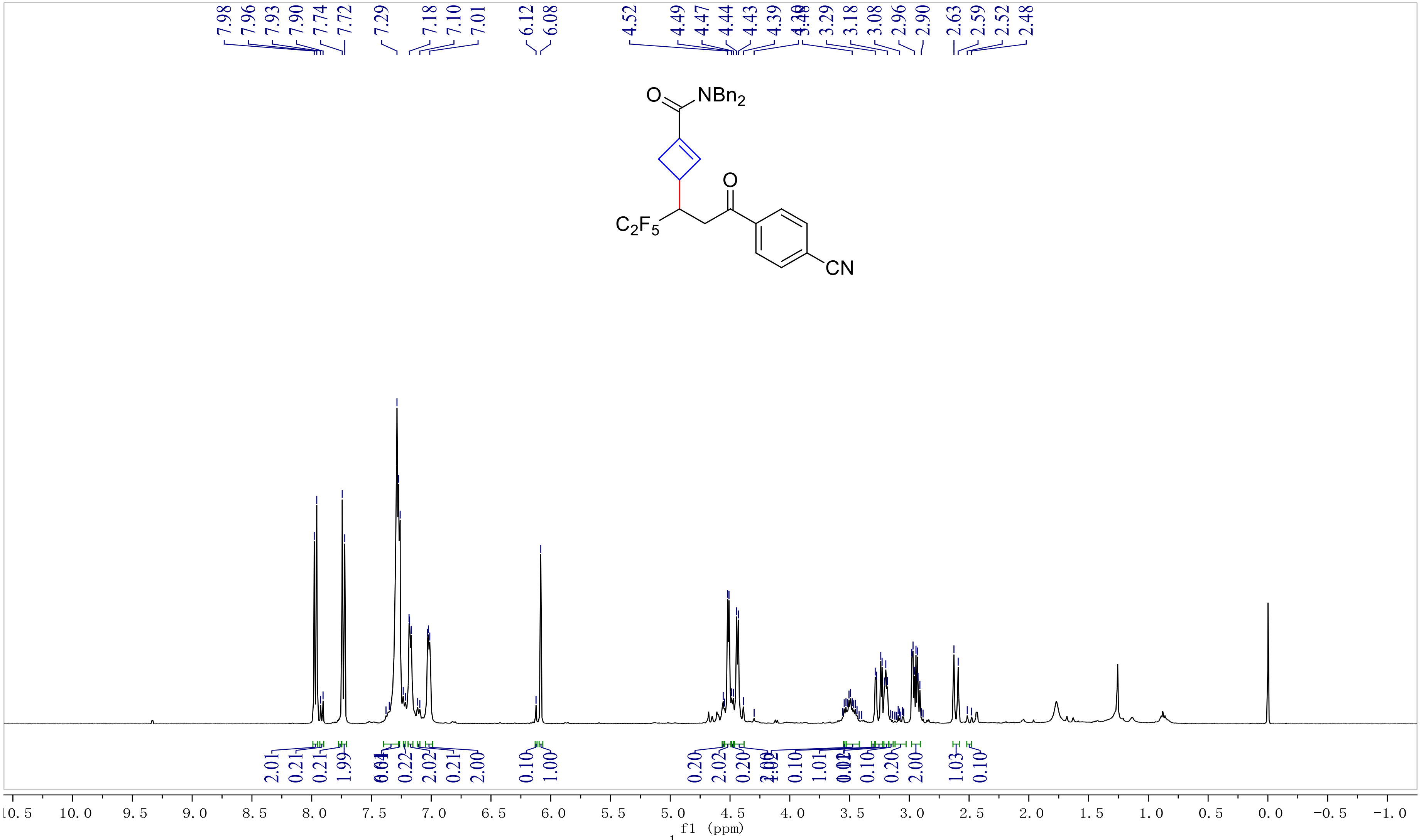

Supplementary Fig. 147 ^{1}H NMR (400 MHz, $CDCl_3$) of **4h**

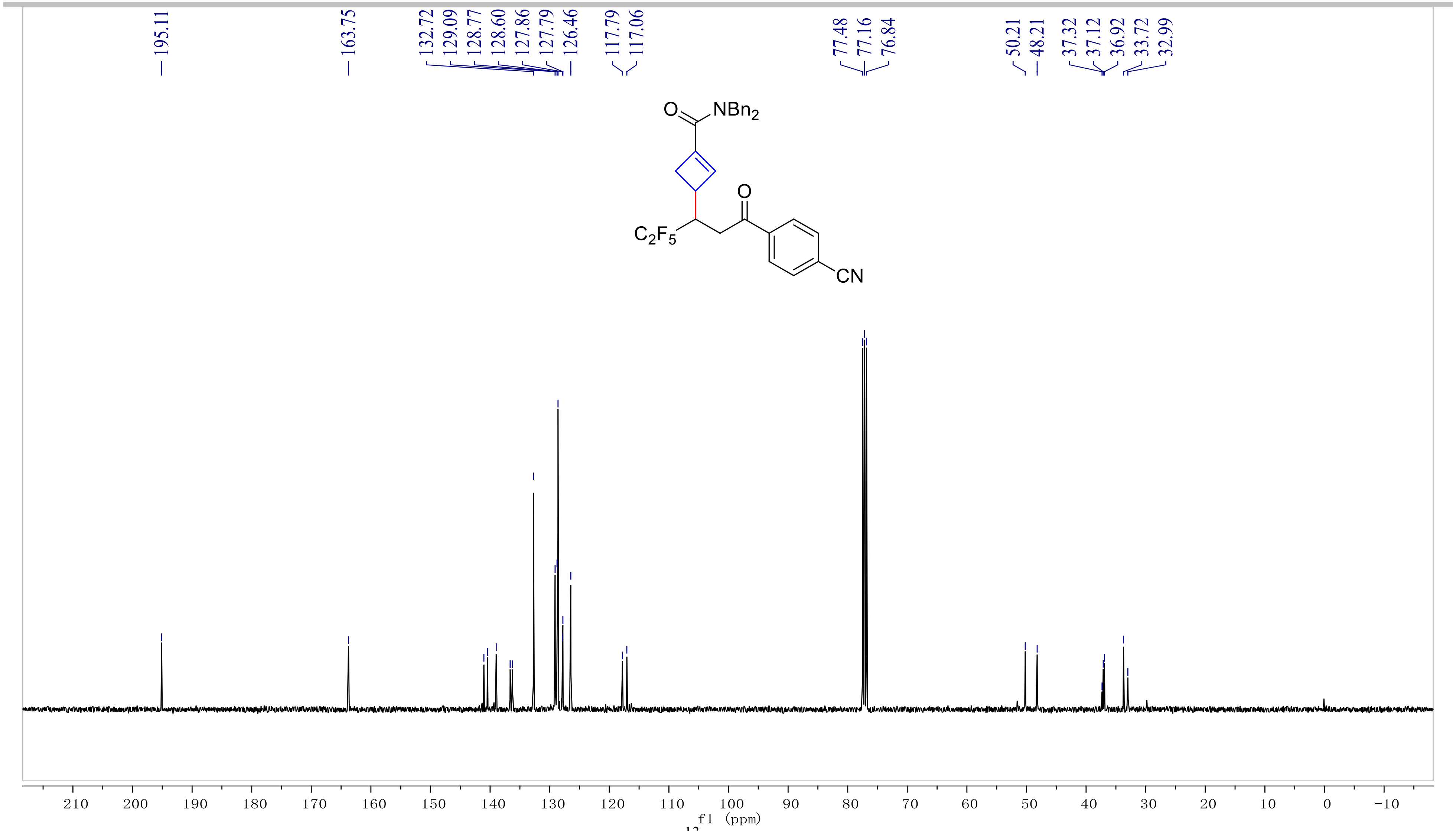

Supplementary Fig. 148 ^{13}C NMR (101 MHz, $CDCl_3$) of **4h**

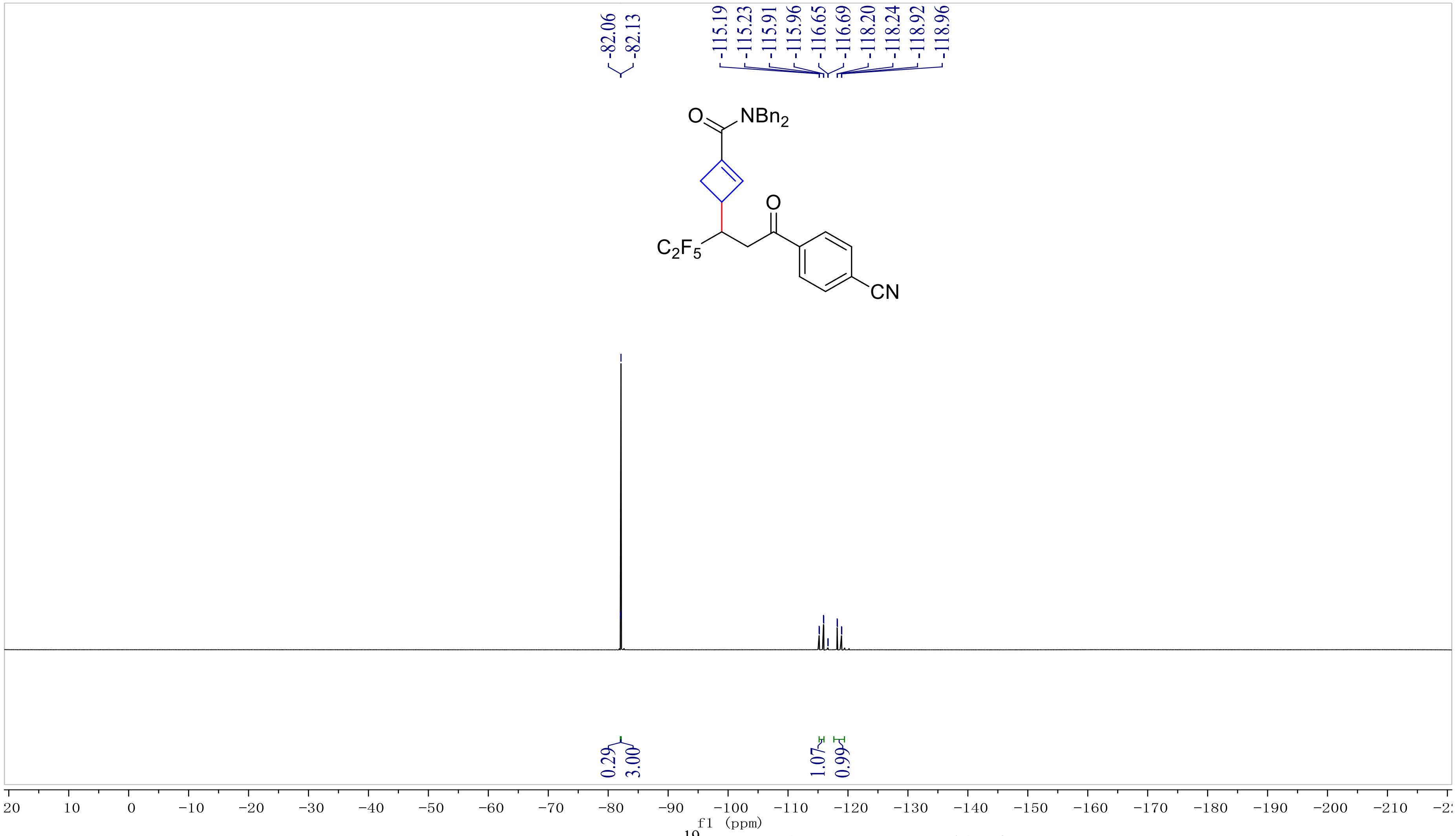

Supplementary Fig. 149 ^{19}F NMR (377 MHz, $CDCl_3$) of **4h**

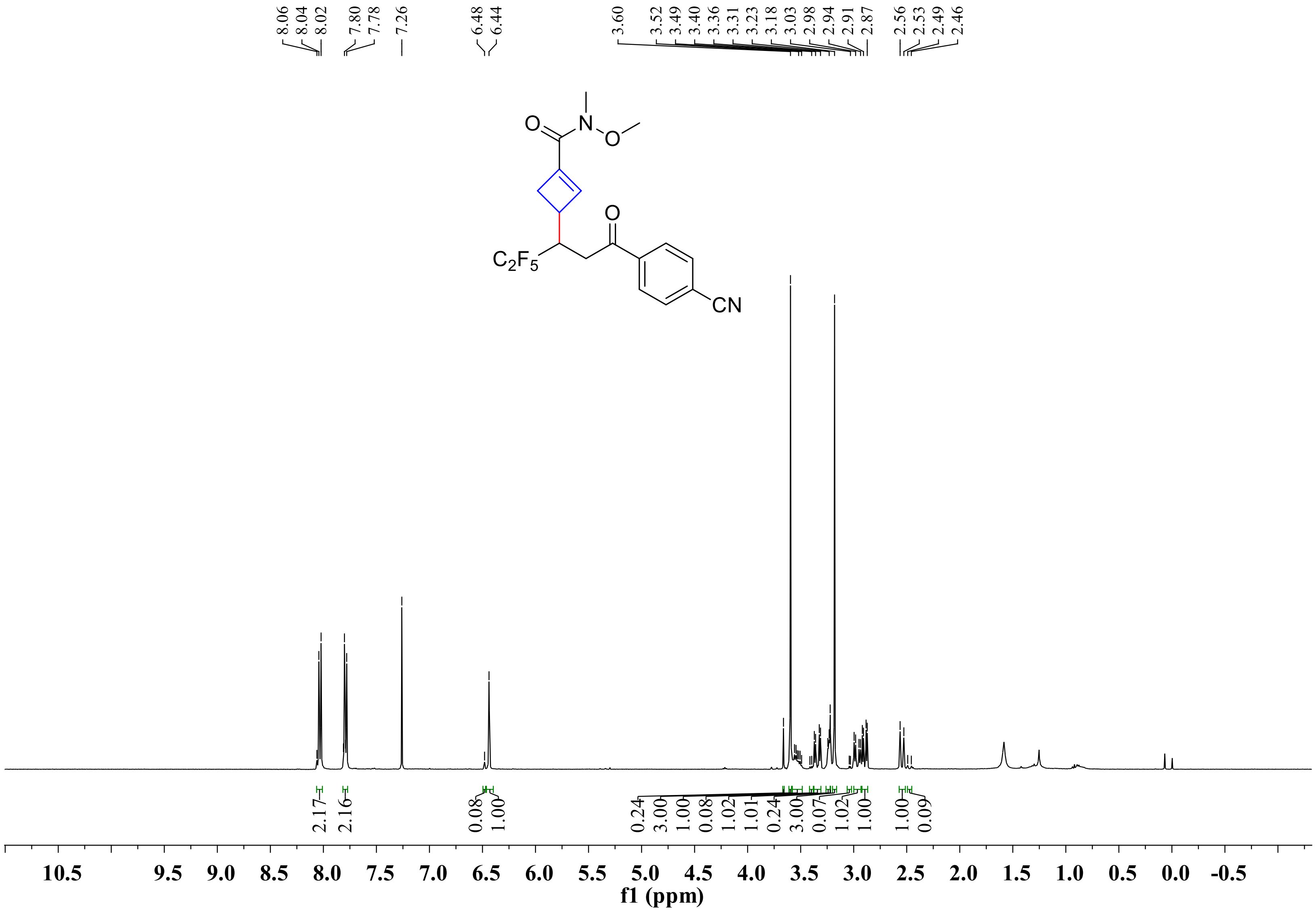

Supplementary Fig. 150 ^{1}H NMR (400 MHz, $CDCl_3$) of **4i**

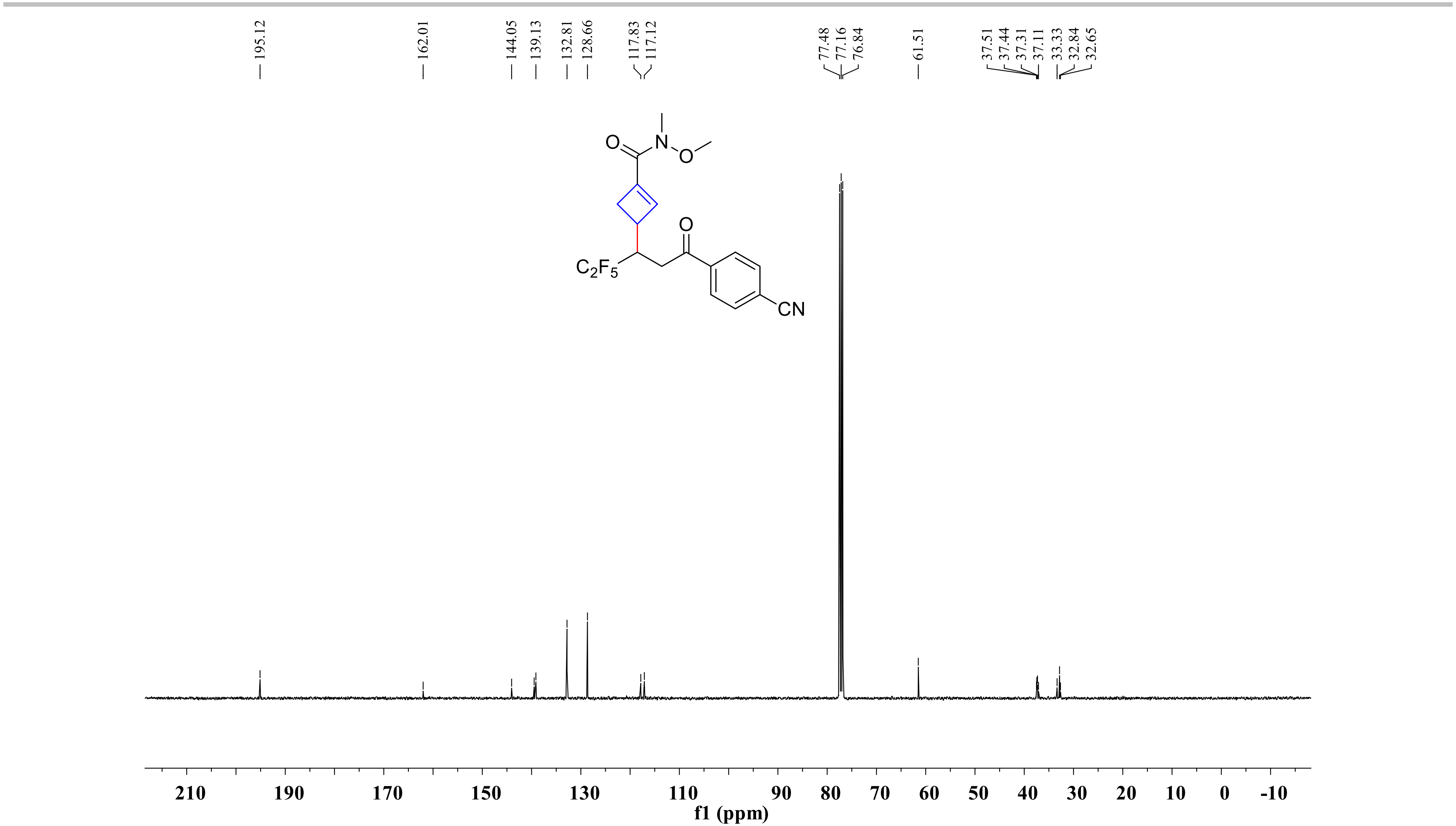

Supplementary Fig. 151 ^{13}C NMR (101 MHz, $CDCl_3$) of **4i**

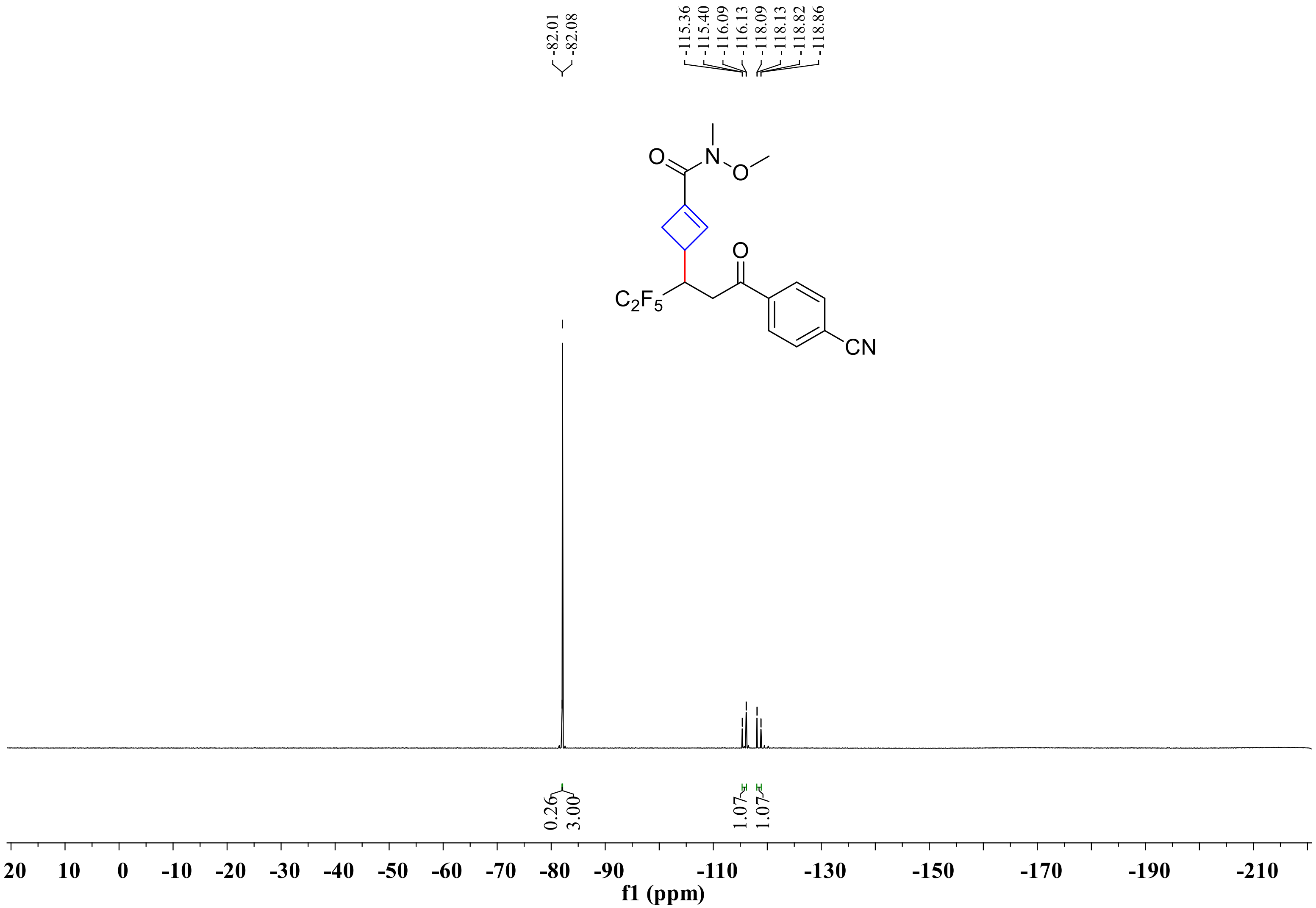

Supplementary Fig. 152 ^{19}F NMR (377 MHz, $CDCl_3$) of **4i**

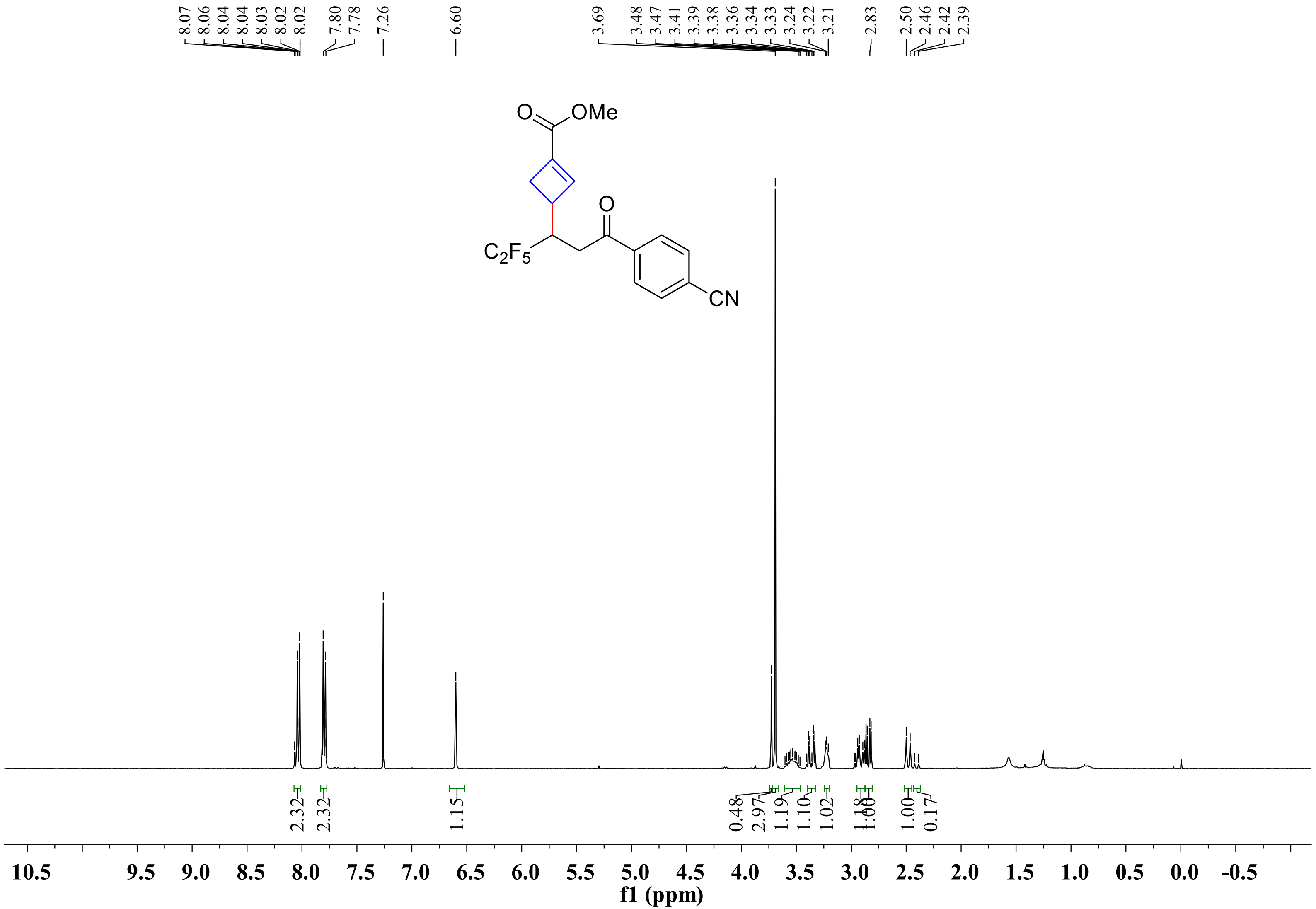

Supplementary Fig. 153 ^{1}H NMR (400 MHz, $CDCl_3$) of **4j**

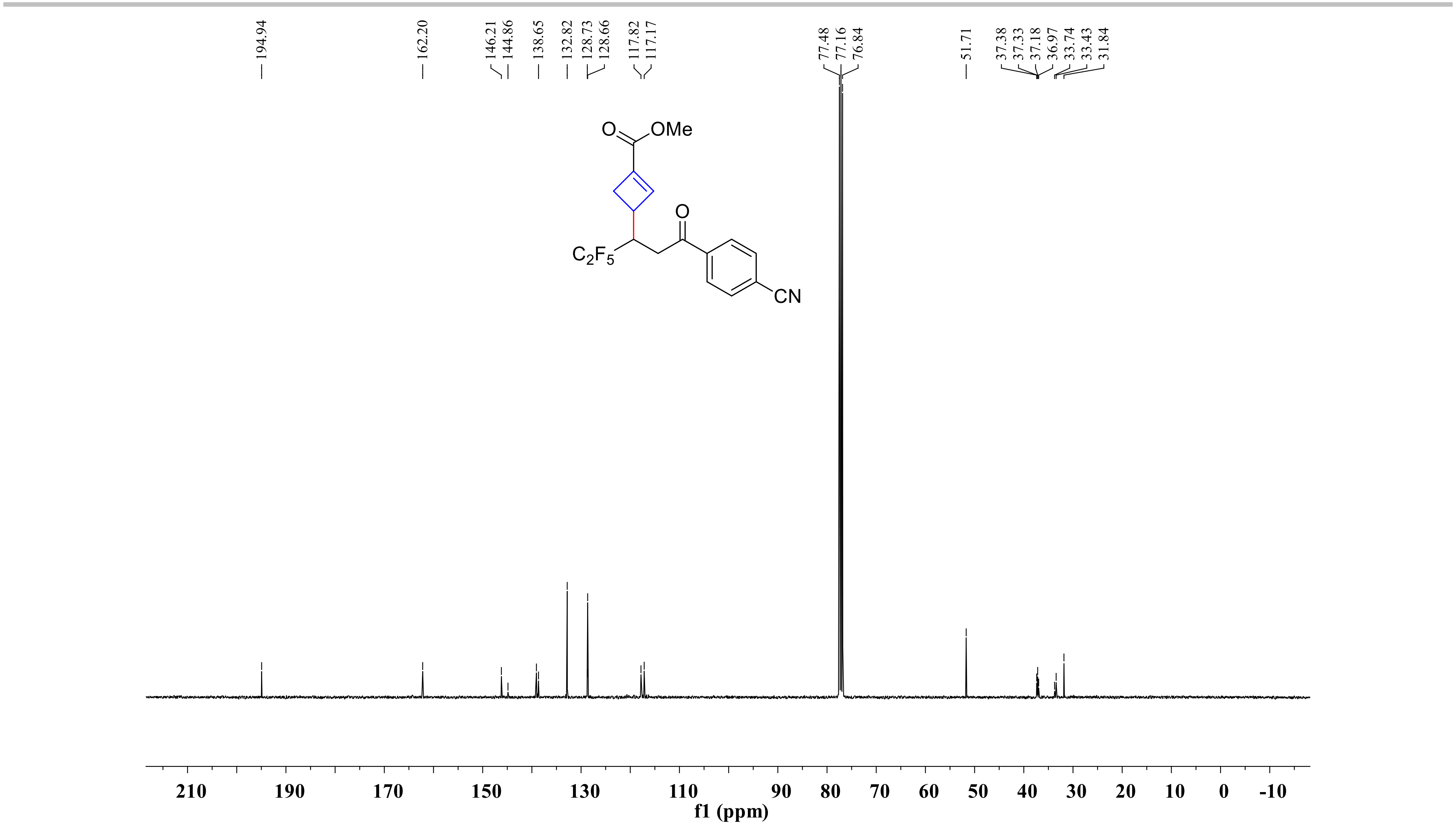

Supplementary Fig. 154 ^{13}C NMR (101 MHz, $CDCl_3$) of **4j**

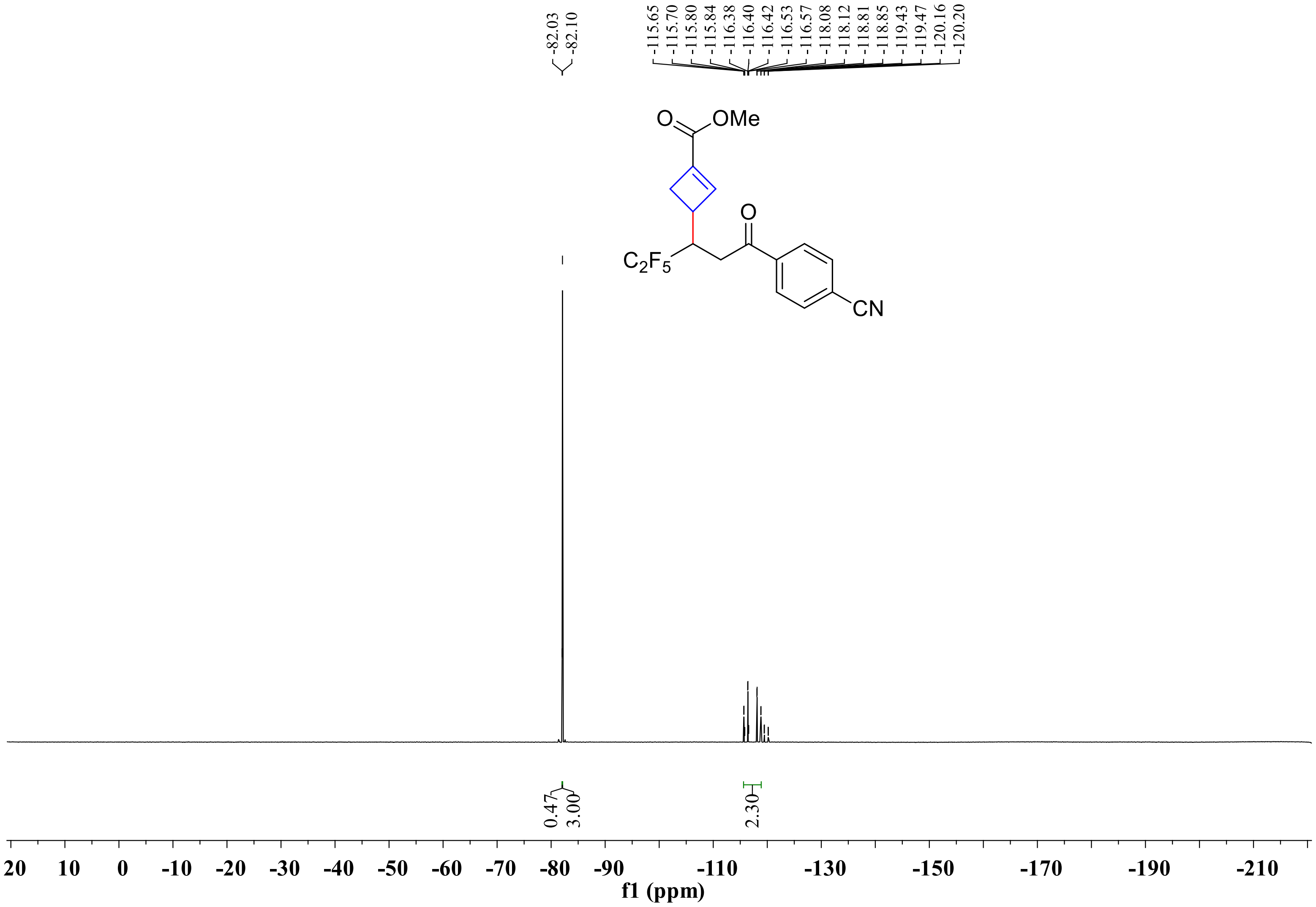

Supplementary Fig. 155 ^{19}F NMR (377 MHz, $CDCl_3$) of **4j**

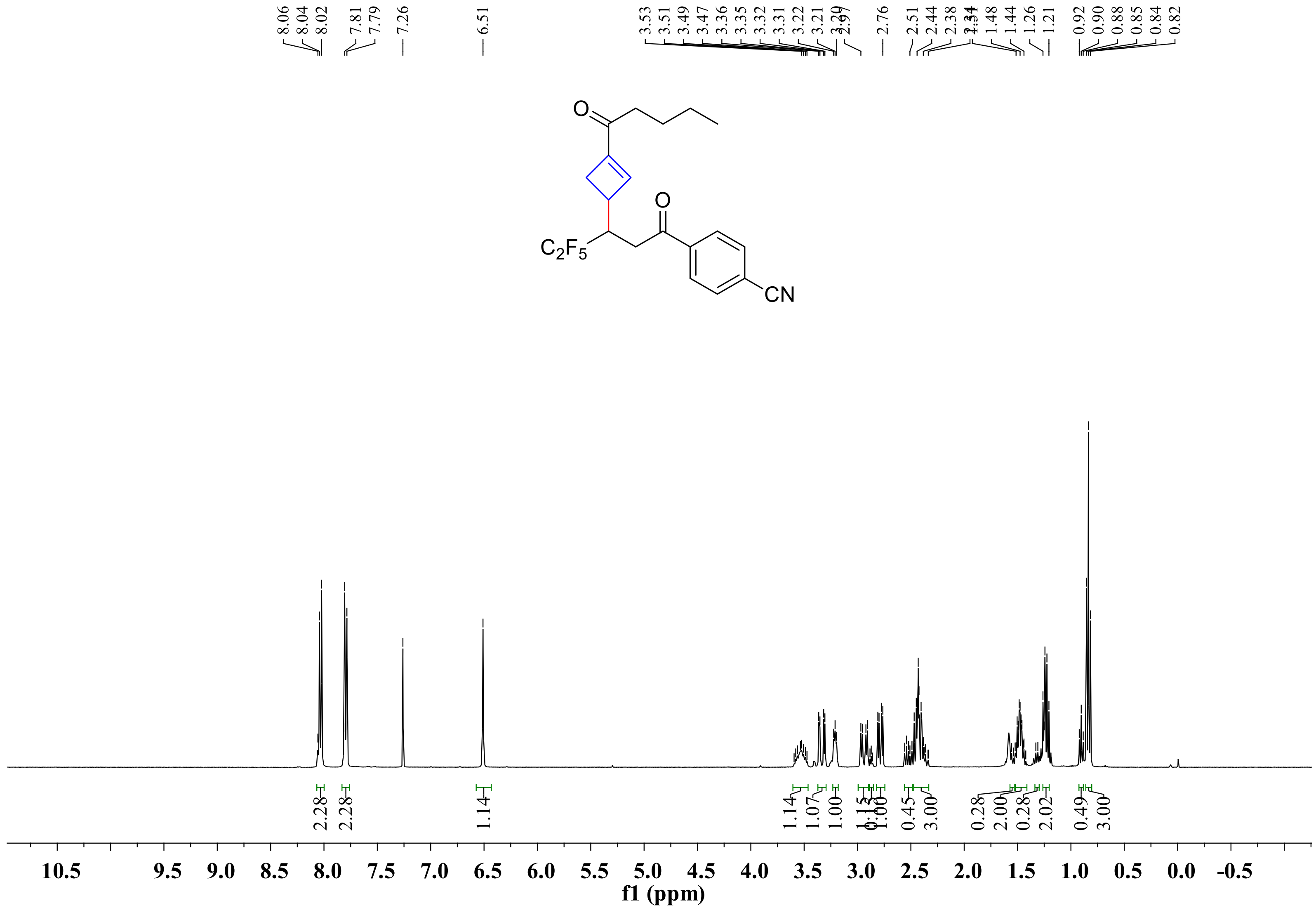

Supplementary Fig. 156 ^{1}H NMR (400 MHz, $CDCl_3$) of **4k**

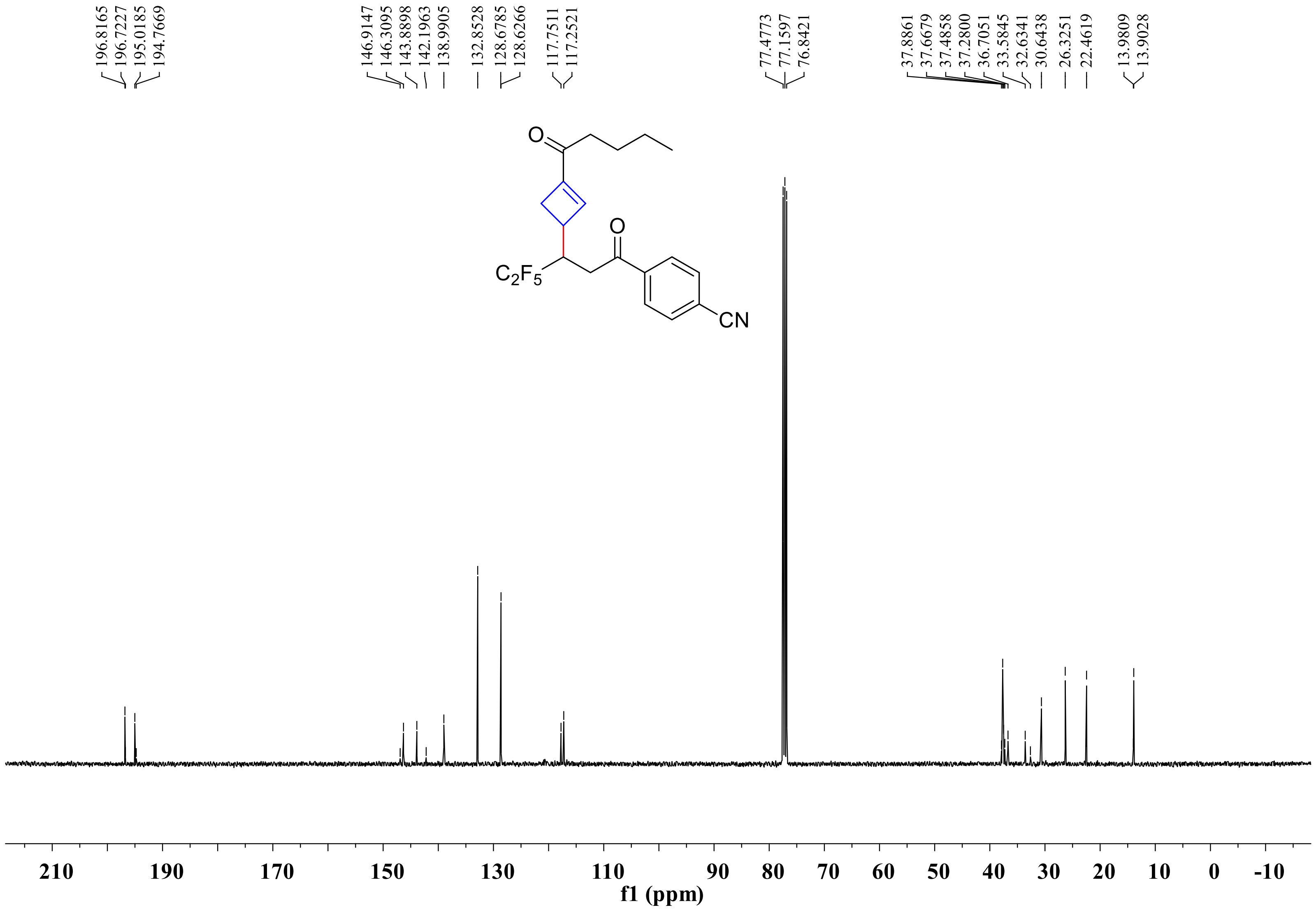

Supplementary Fig. 157 ^{13}C NMR (101 MHz, $CDCl_3$) of **4k**

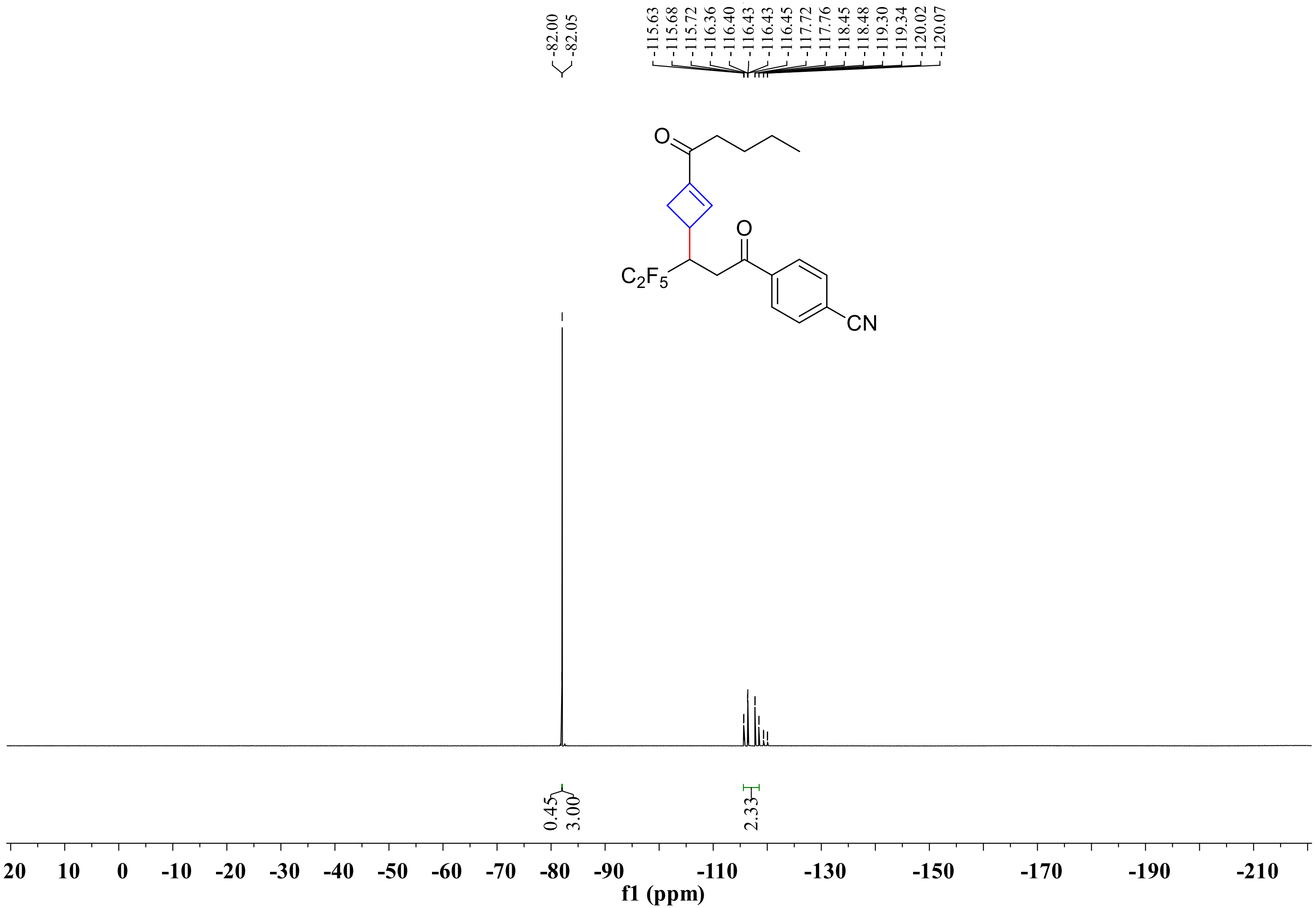

Supplementary Fig. 158 ^{19}F NMR (377 MHz, $CDCl_3$) of **4k**

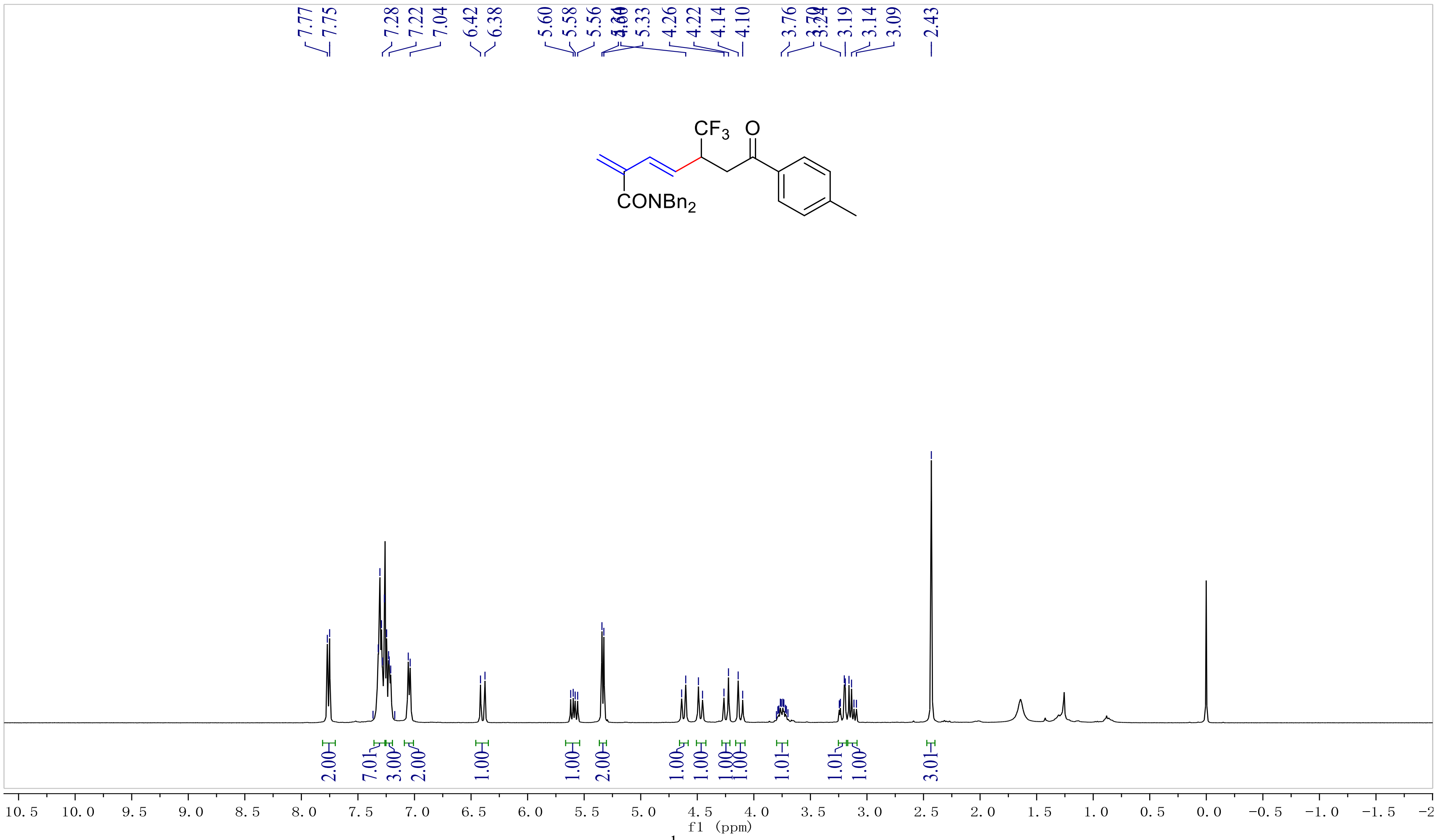

Supplementary Fig. 159 ^{1}H NMR (400 MHz, $CDCl_3$) of **5a**

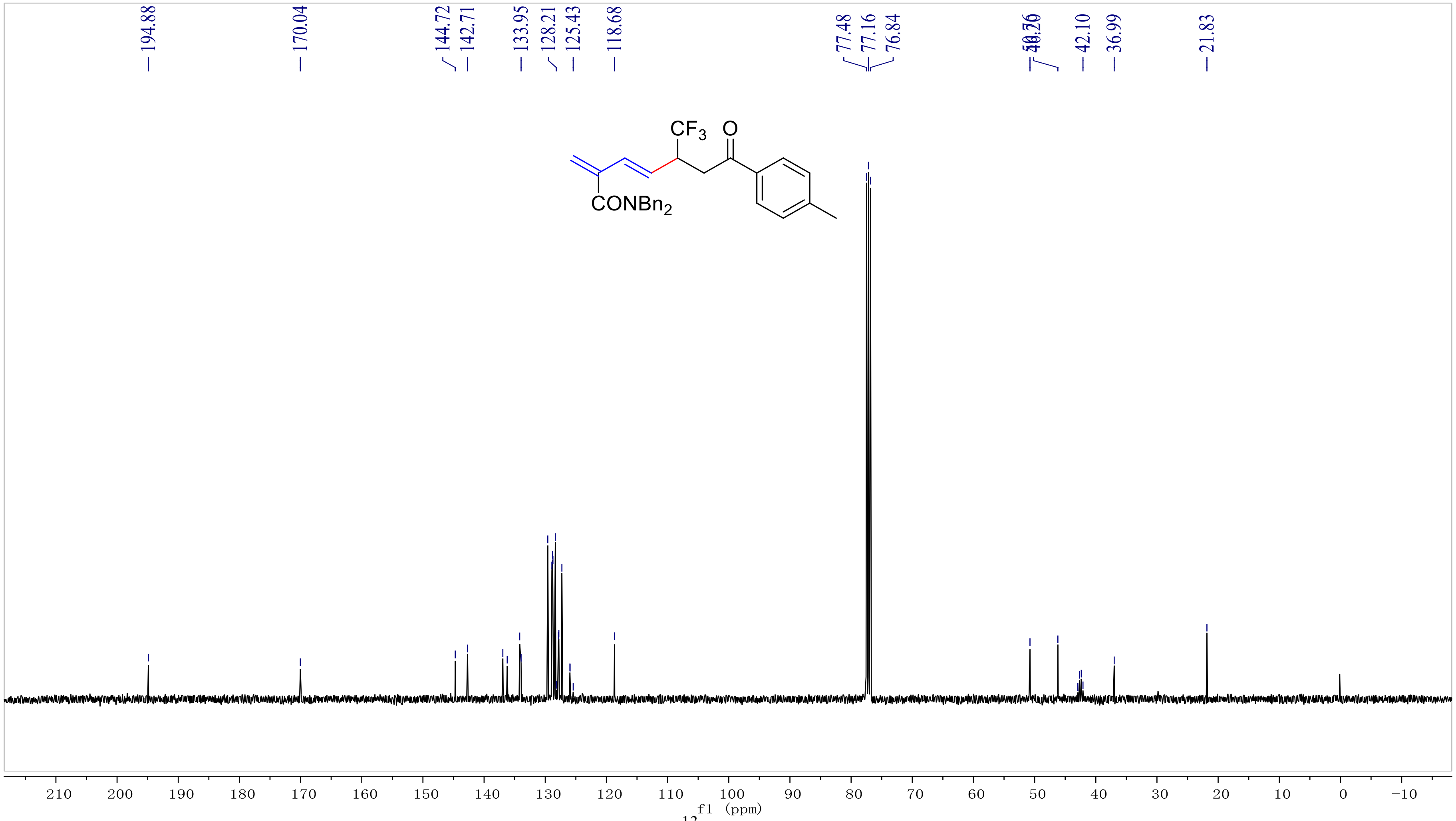

Supplementary Fig. 160 ^{13}C NMR (101 MHz, $CDCl_3$) of **5a**

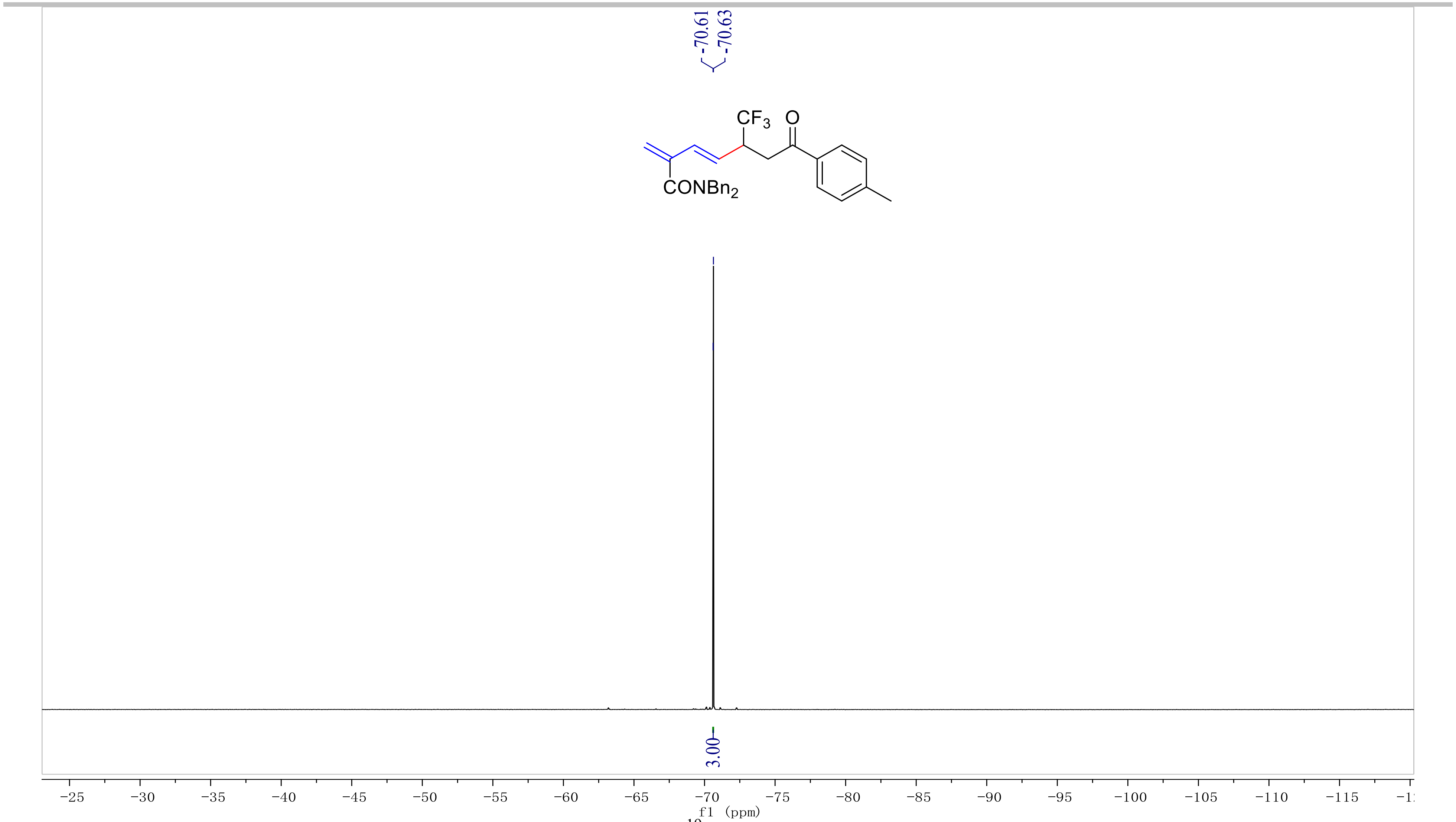

Supplementary Fig. 161 ^{19}F NMR (377 MHz, $CDCl_3$) of **5a**

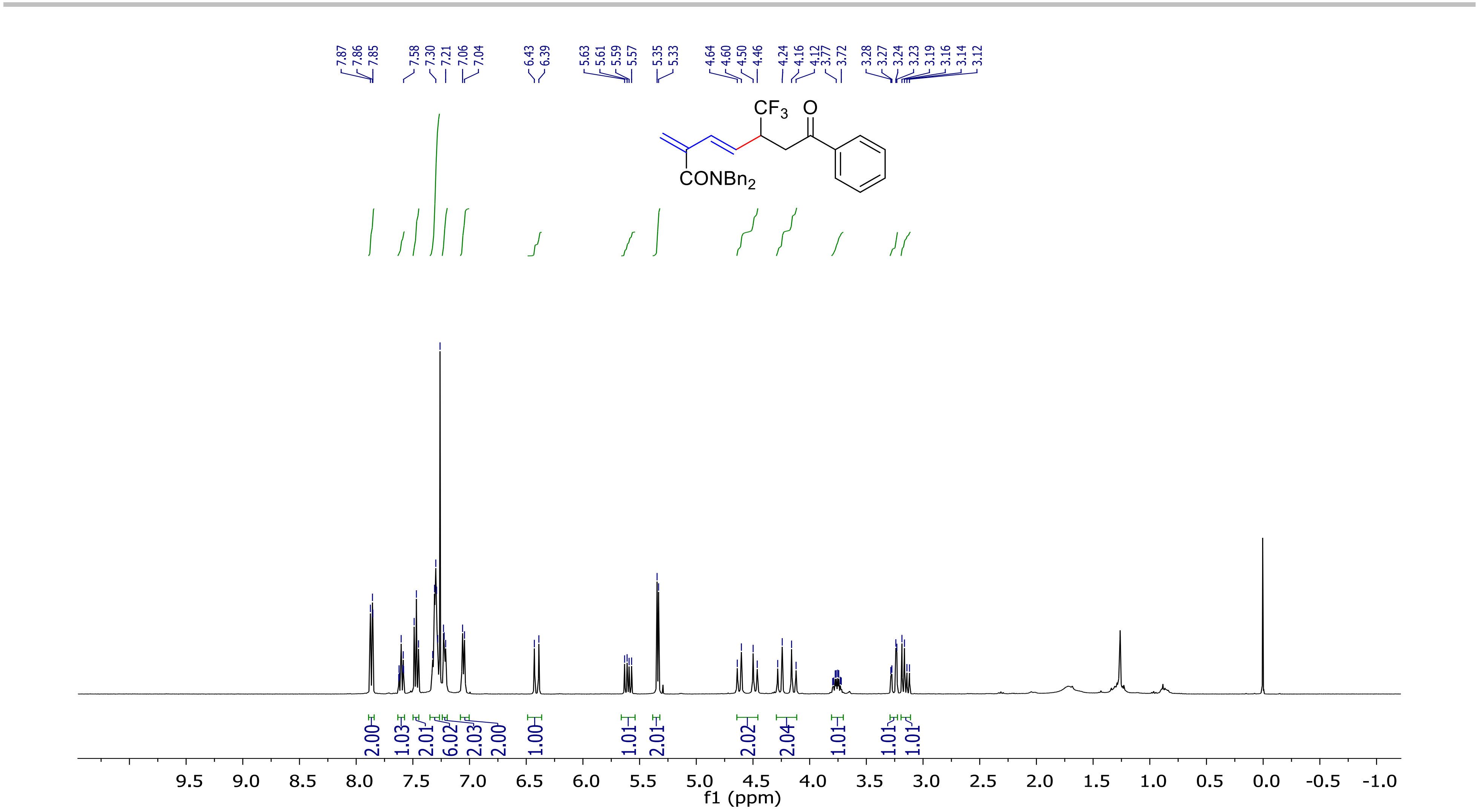

Supplementary Fig. 162 ^{1}H NMR (400 MHz, $CDCl_3$) of **5b**

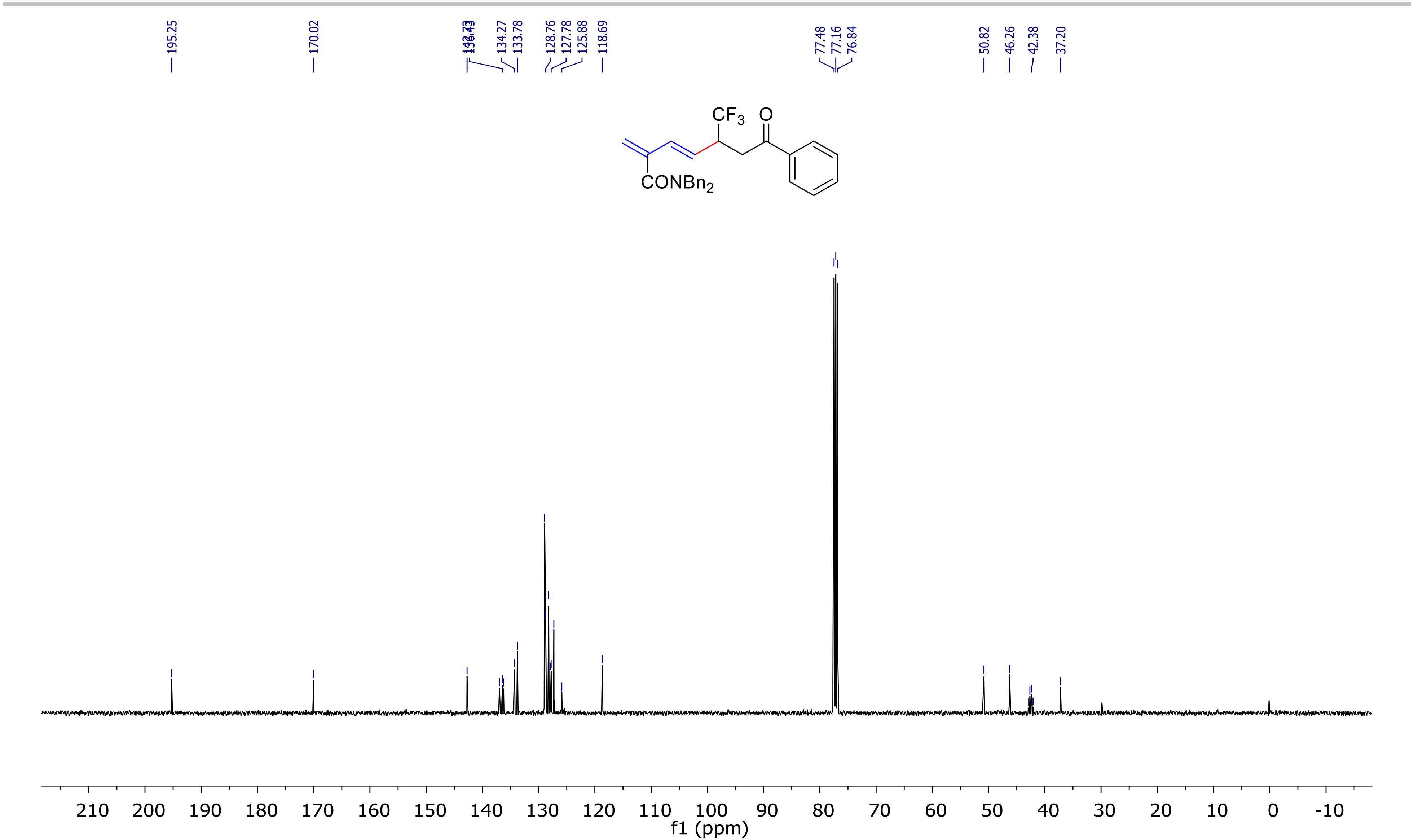

Supplementary Fig. 163 ^{13}C NMR (101 MHz, $CDCl_3$) of **5b**

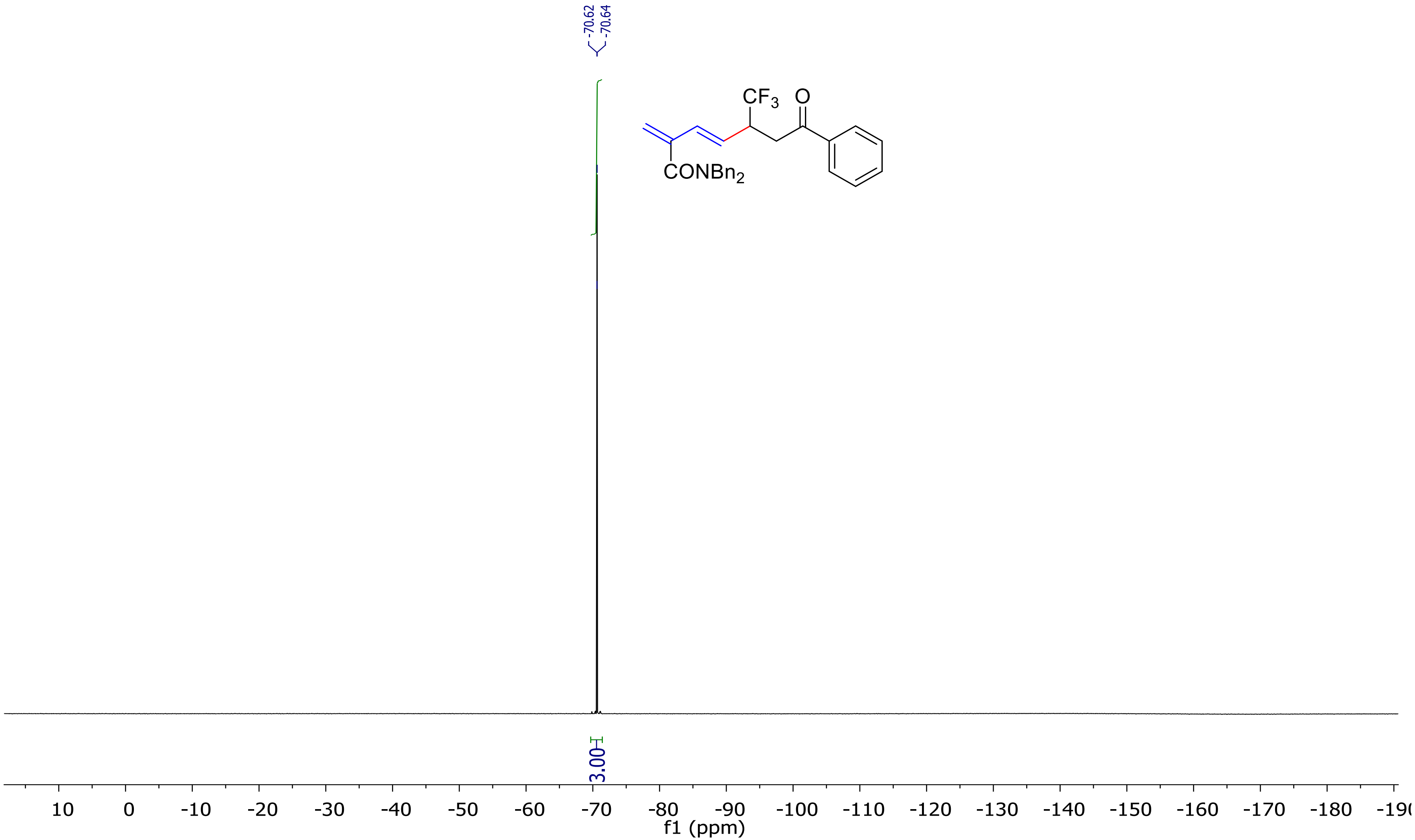

Supplementary Fig. 164 ^{19}F NMR (377 MHz, $CDCl_3$) of **5b**

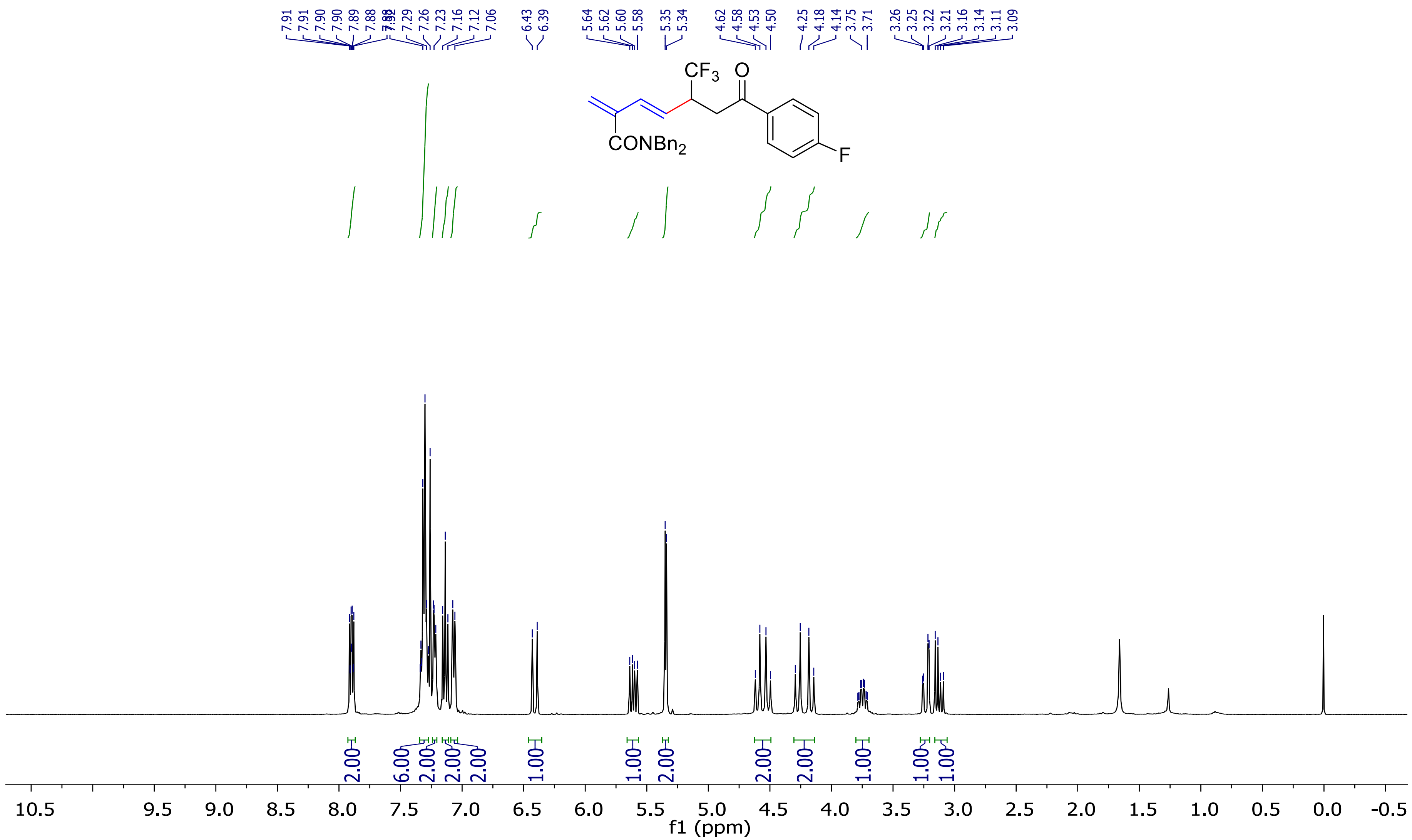

Supplementary Fig. 165 ^{1}H NMR (400 MHz, $CDCl_3$) of **5c**

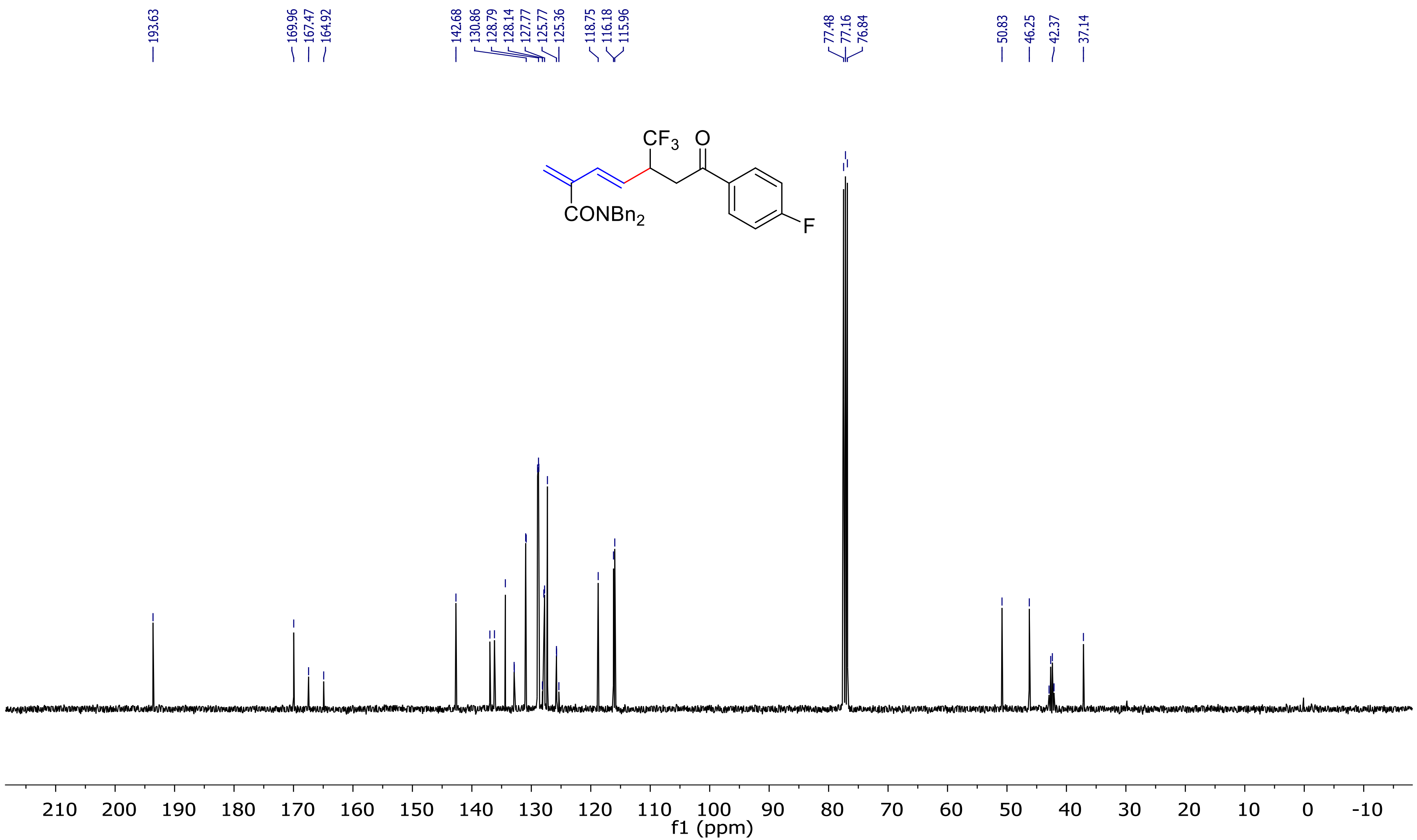

Supplementary Fig. 166 ^{13}C NMR (101 MHz, $CDCl_3$) of **5c**

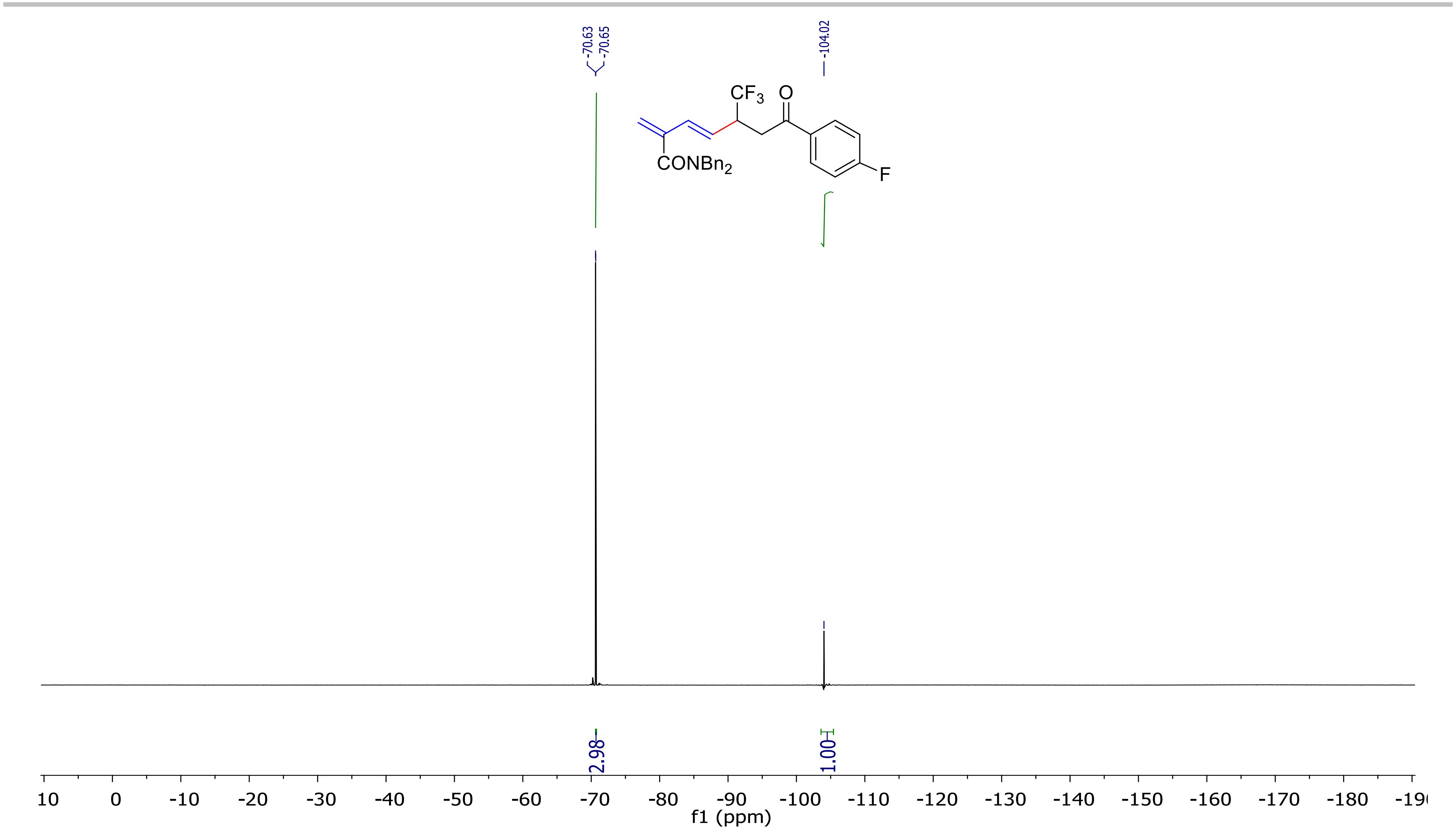

Supplementary Fig. 167 ^{19}F NMR (377 MHz, $CDCl_3$) of **5c**

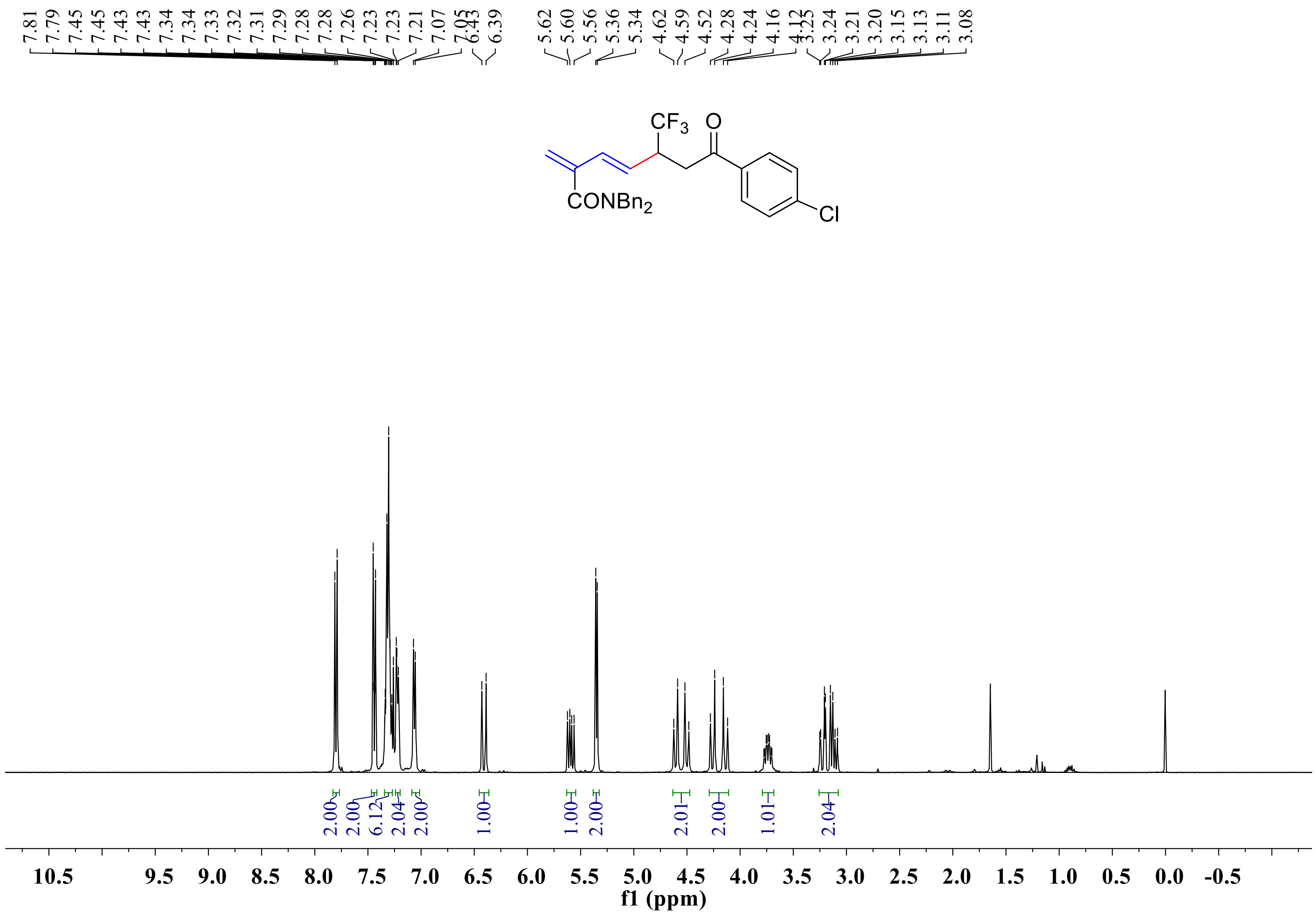

Supplementary Fig. 168 ^{1}H NMR (400 MHz, $CDCl_3$) of **5d**

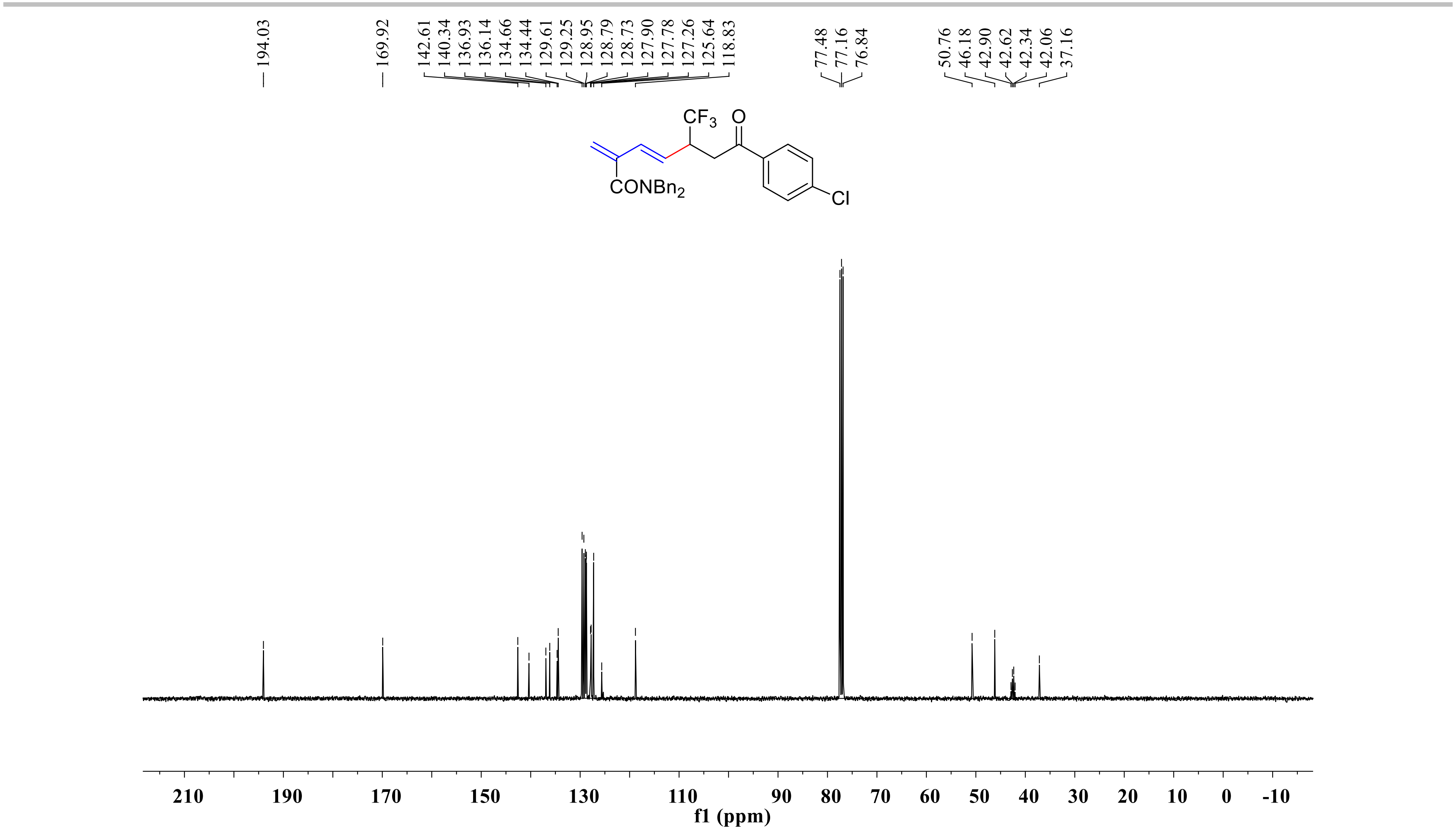

Supplementary Fig. 169 ^{13}C NMR (101 MHz, $CDCl_3$) of **5d**

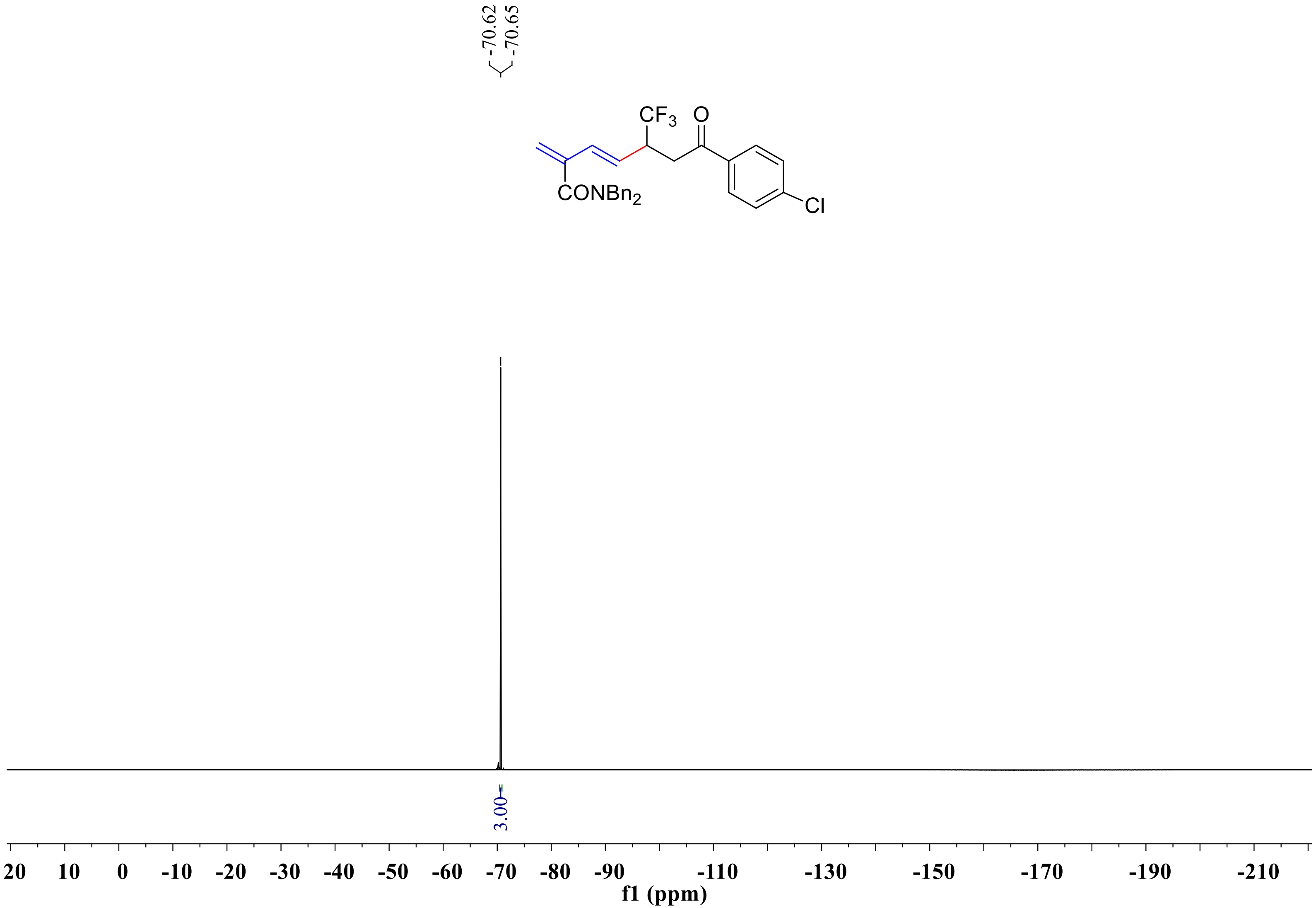

Supplementary Fig. 170 ^{19}F NMR (377 MHz, $CDCl_3$) of **5d**

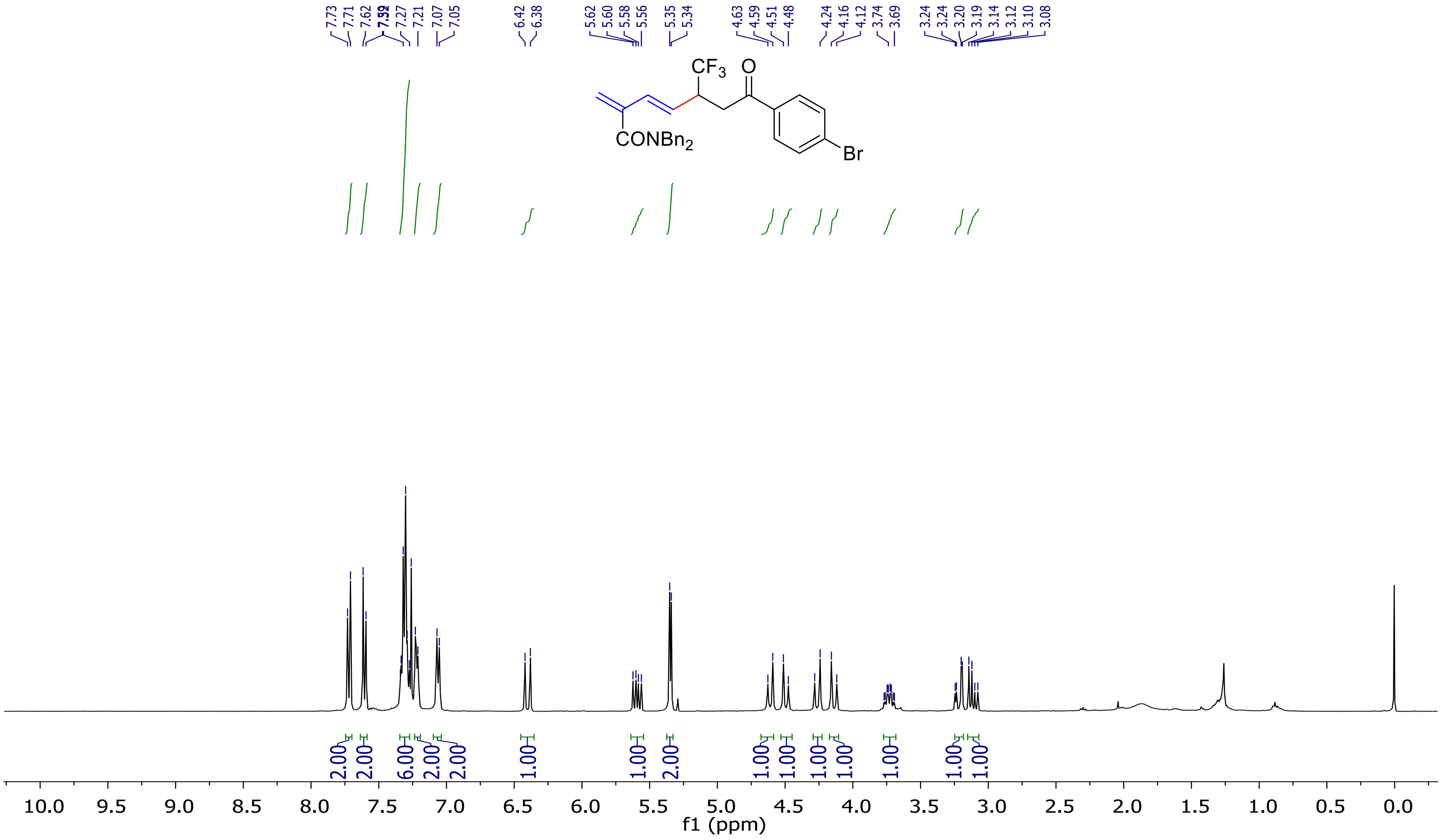

Supplementary Fig. 171 ^{1}H NMR (400 MHz, $CDCl_3$) of **5e**

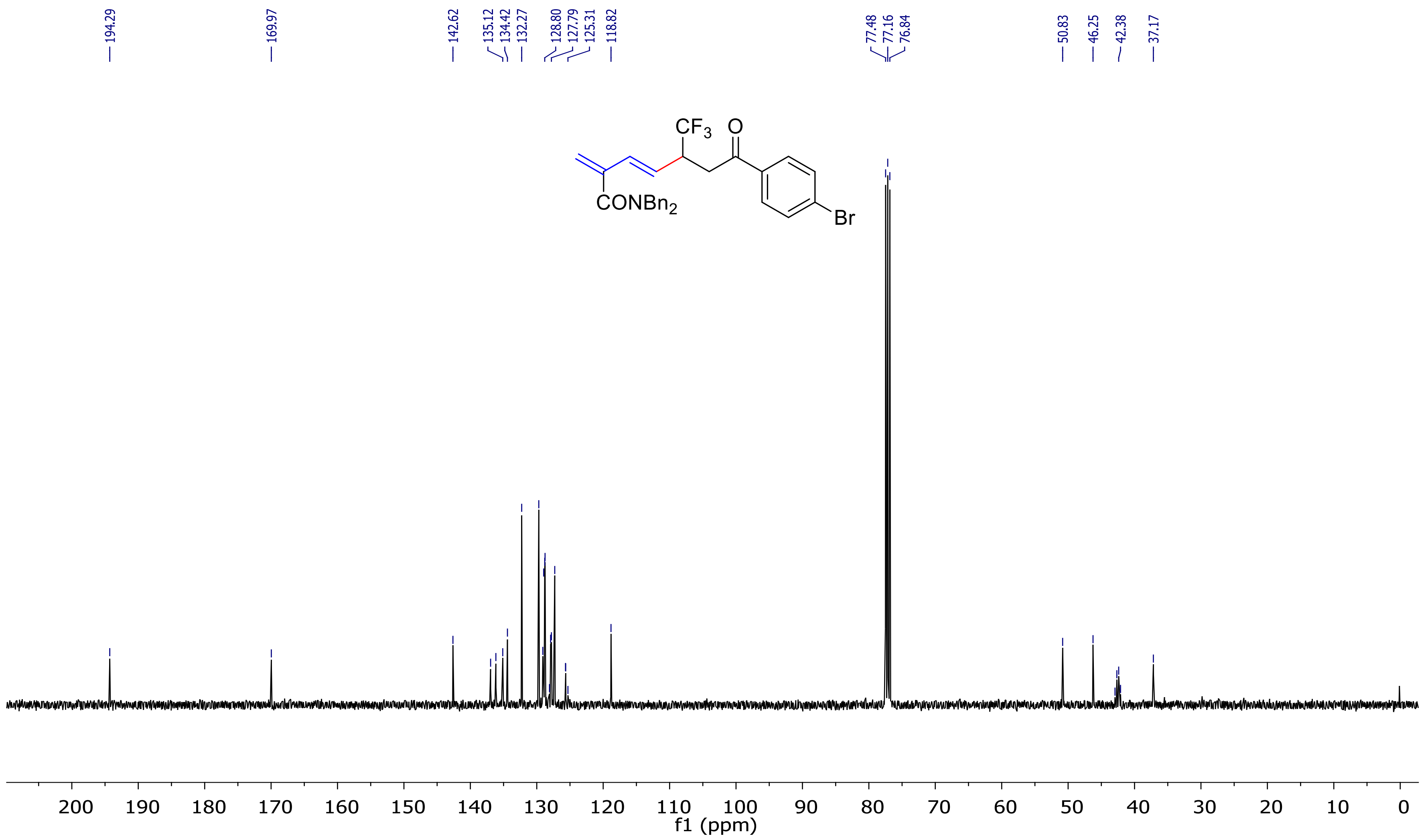

Supplementary Fig. 172 ^{13}C NMR (101 MHz, $CDCl_3$) of **5e**

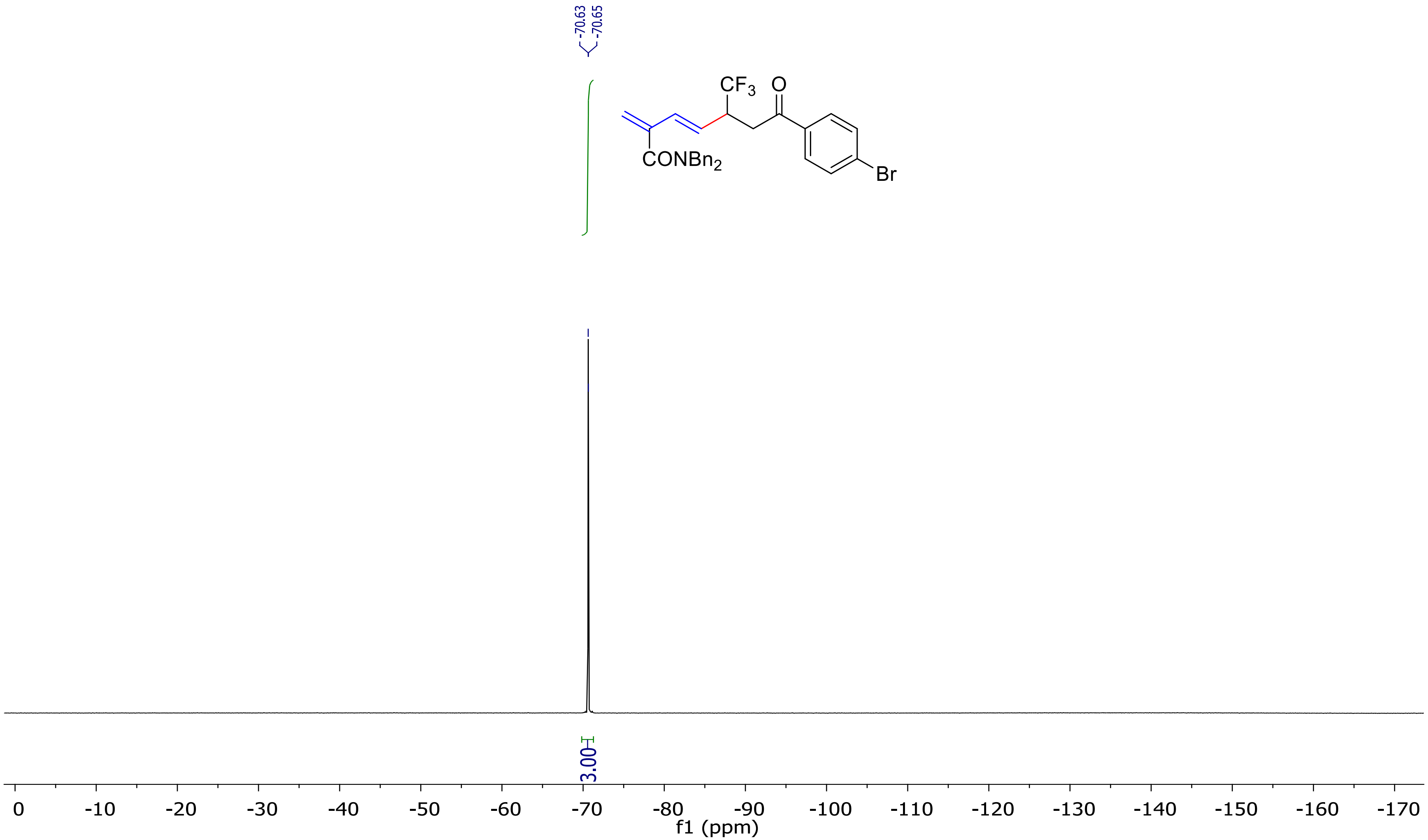

Supplementary Fig. 173 ^{19}F NMR (377 MHz, $CDCl_3$) of **5e**

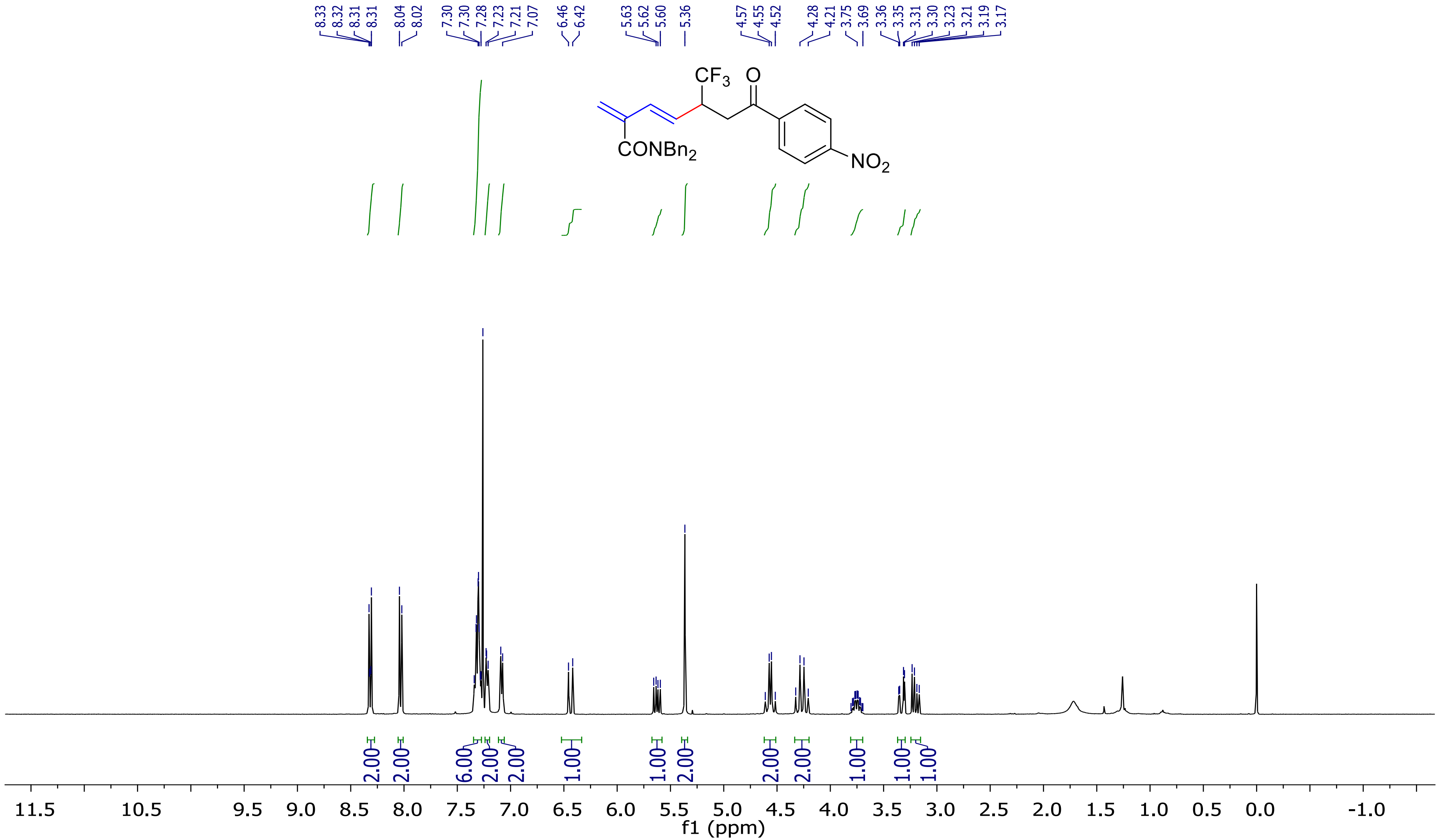

Supplementary Fig. 174 ^{1}H NMR (400 MHz, $CDCl_3$) of **5f**

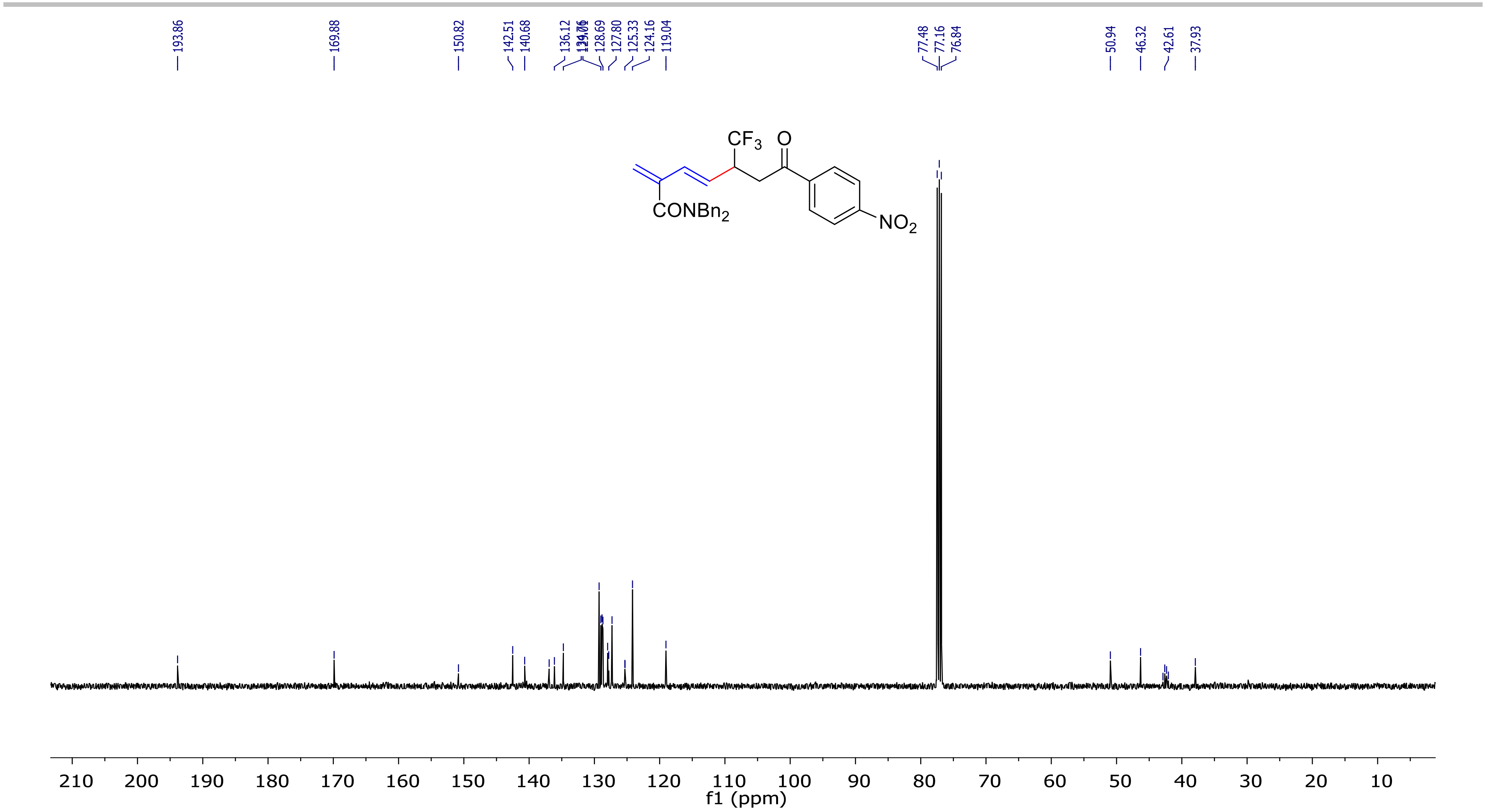

Supplementary Fig. 175 ^{13}C NMR (101 MHz, $CDCl_3$) of **5f**

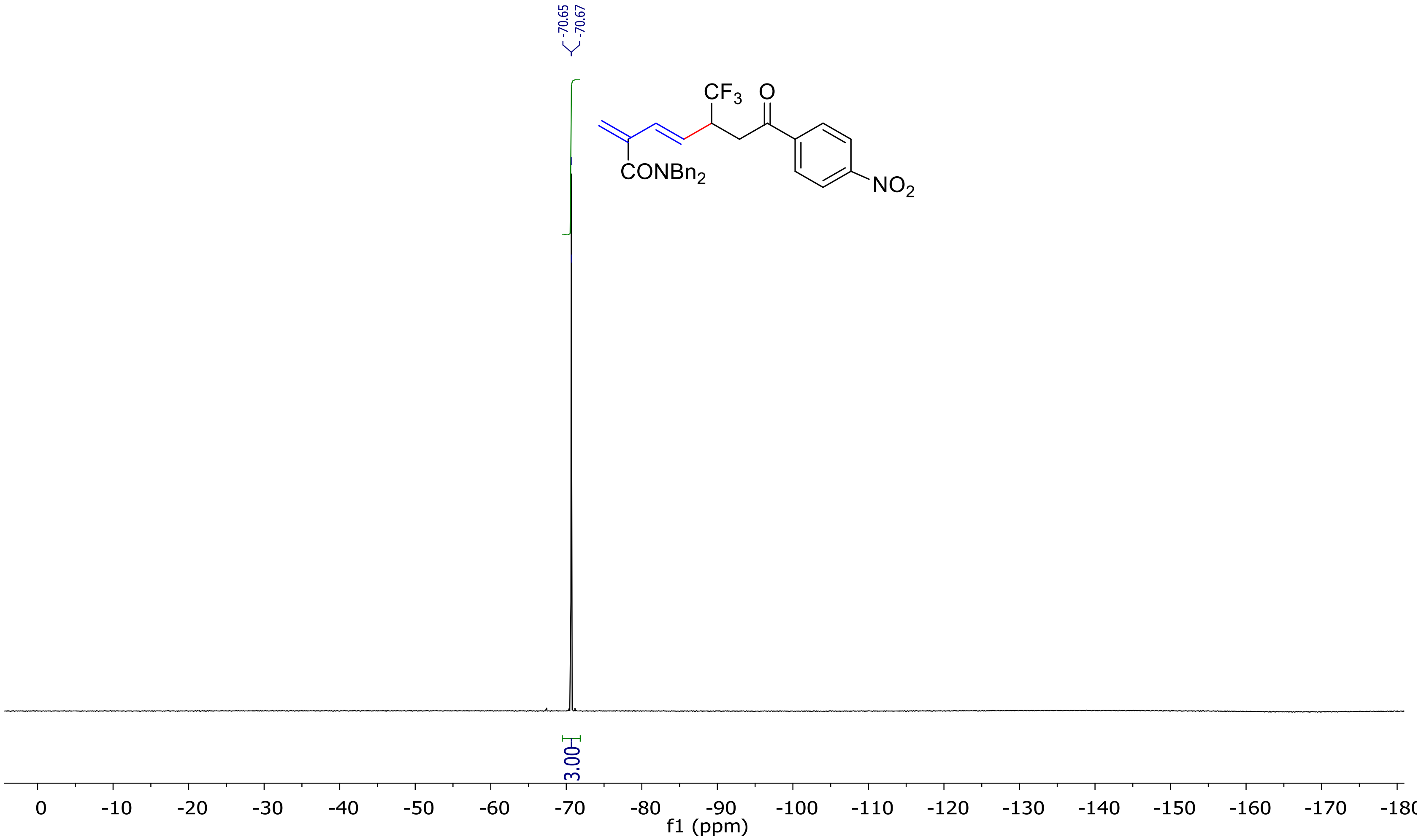

Supplementary Fig. 176 ^{19}F NMR (377 MHz, $CDCl_3$) of **5f**

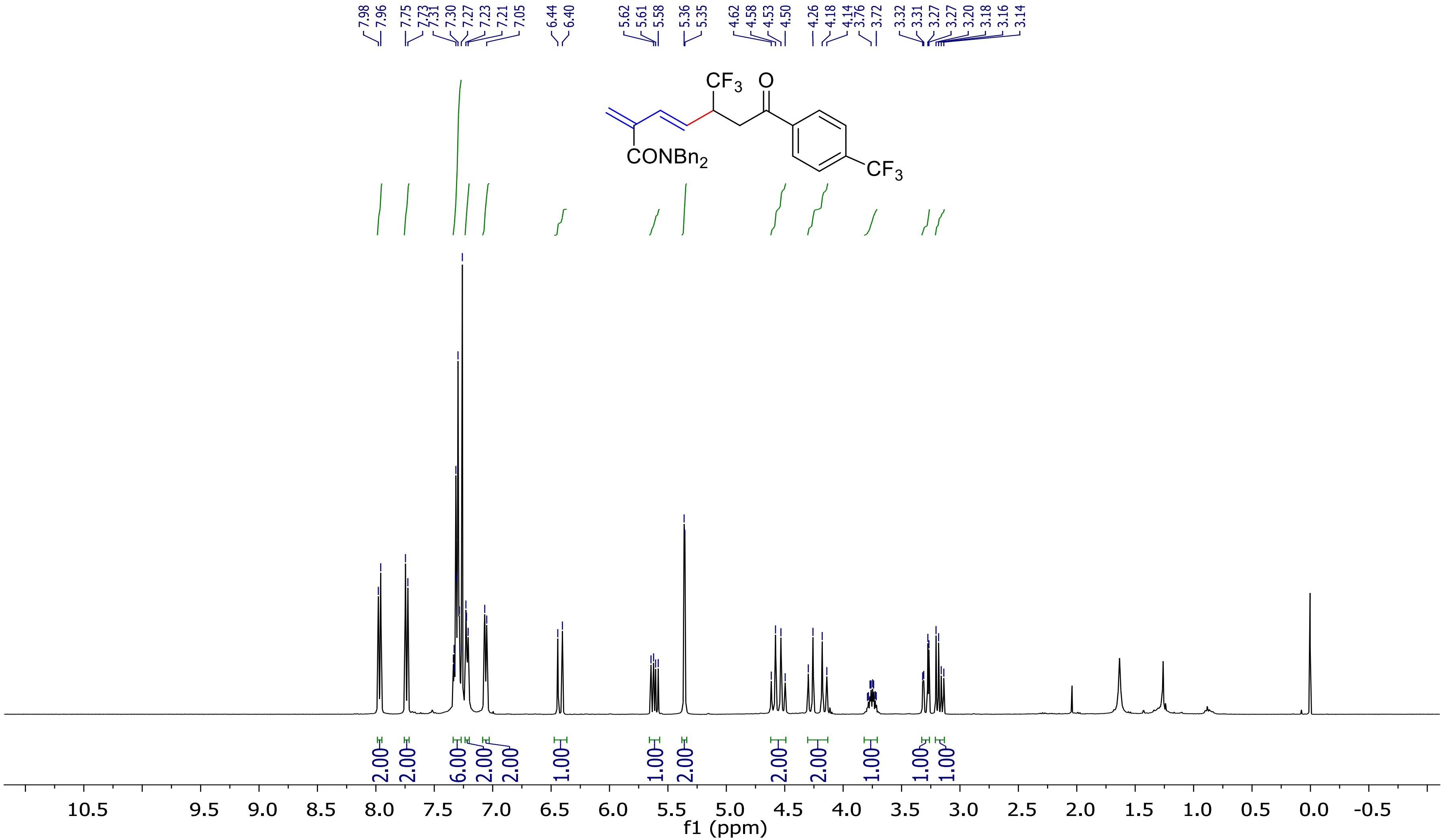

Supplementary Fig. 177 ^{1}H NMR (400 MHz, $CDCl_3$) of **5g**

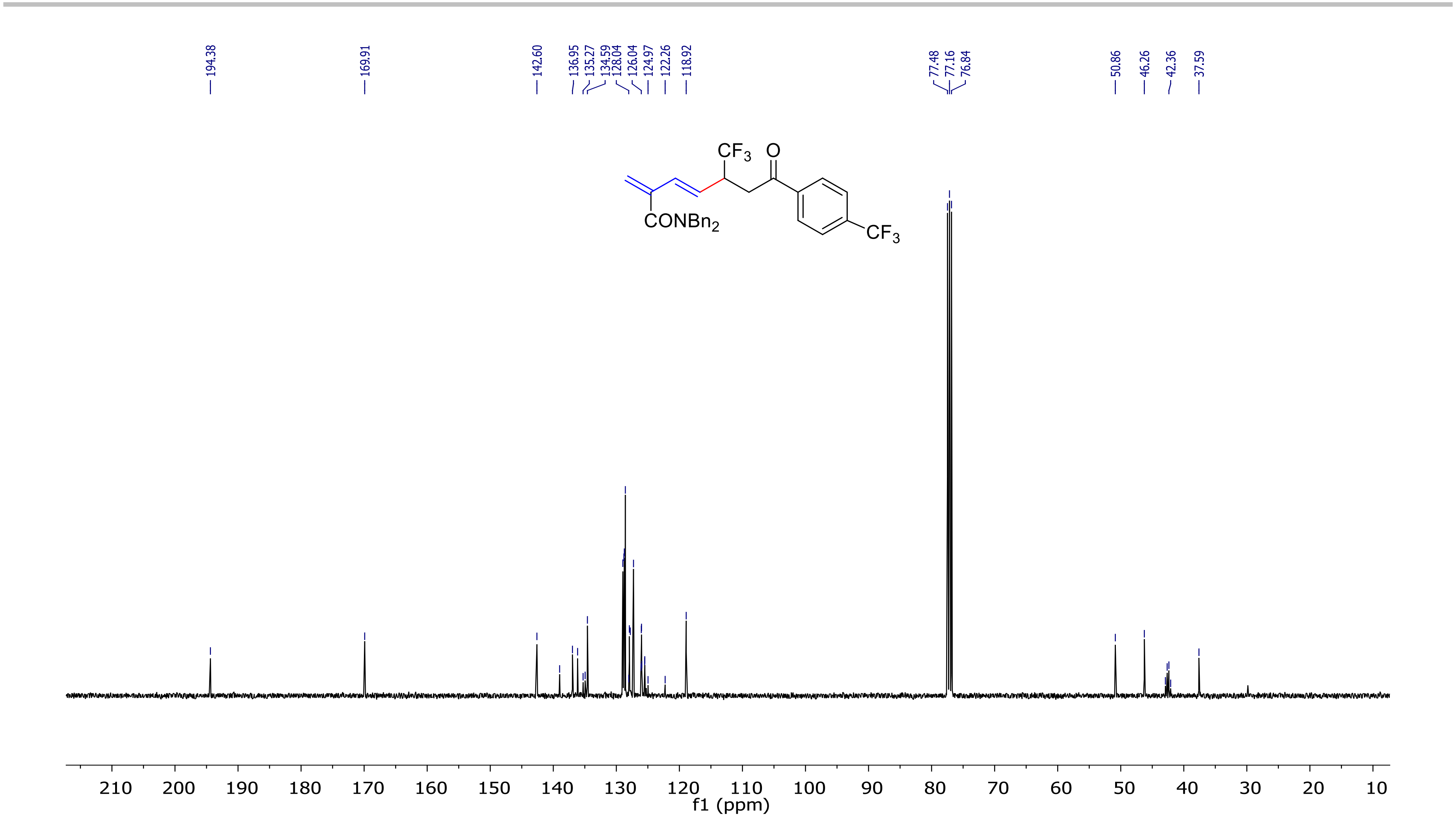

Supplementary Fig. 178 ^{13}C NMR (101 MHz, $CDCl_3$) of **5g**

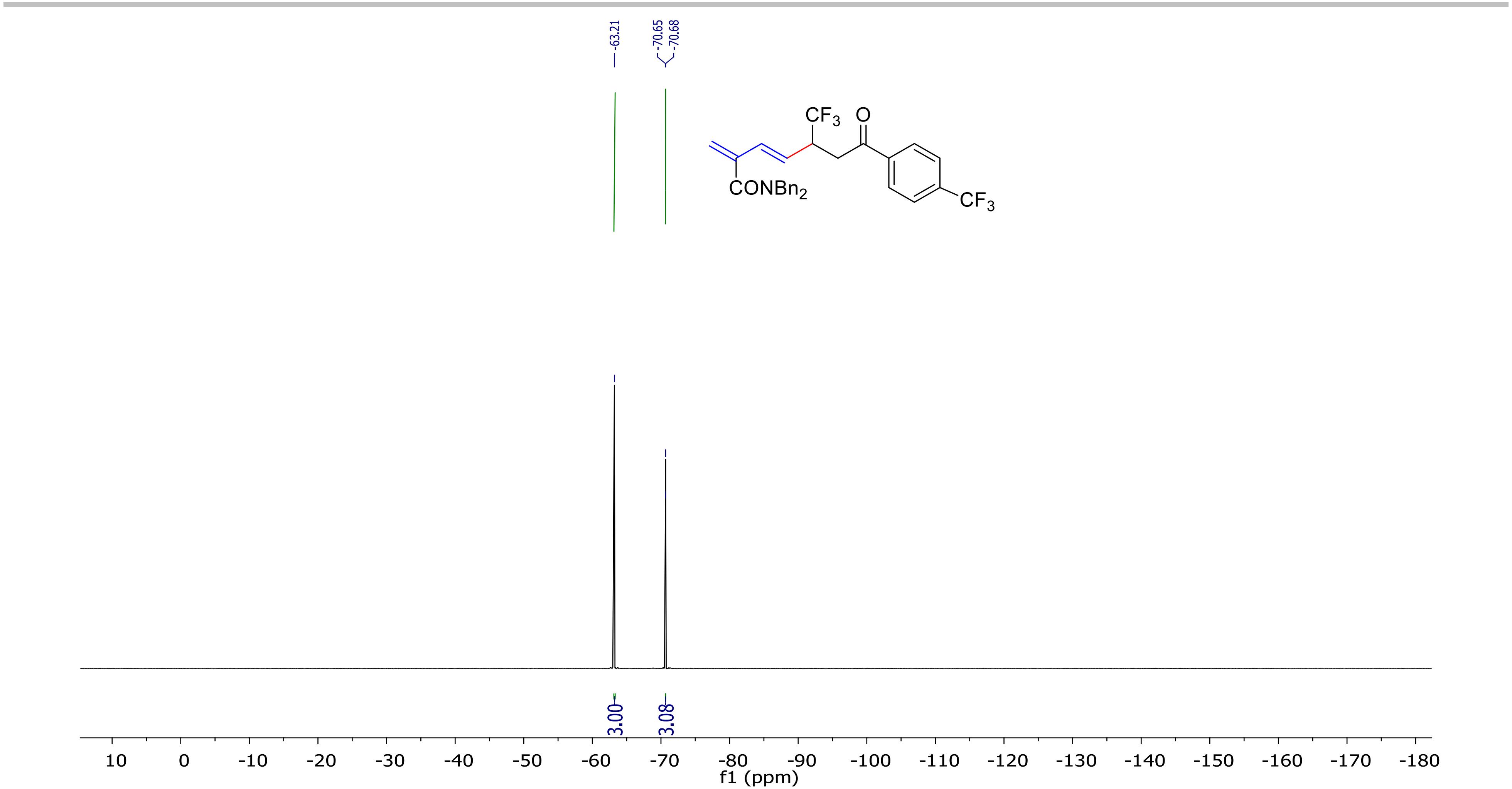

Supplementary Fig. 179 ^{19}F NMR (377 MHz, $CDCl_3$) of **5g**

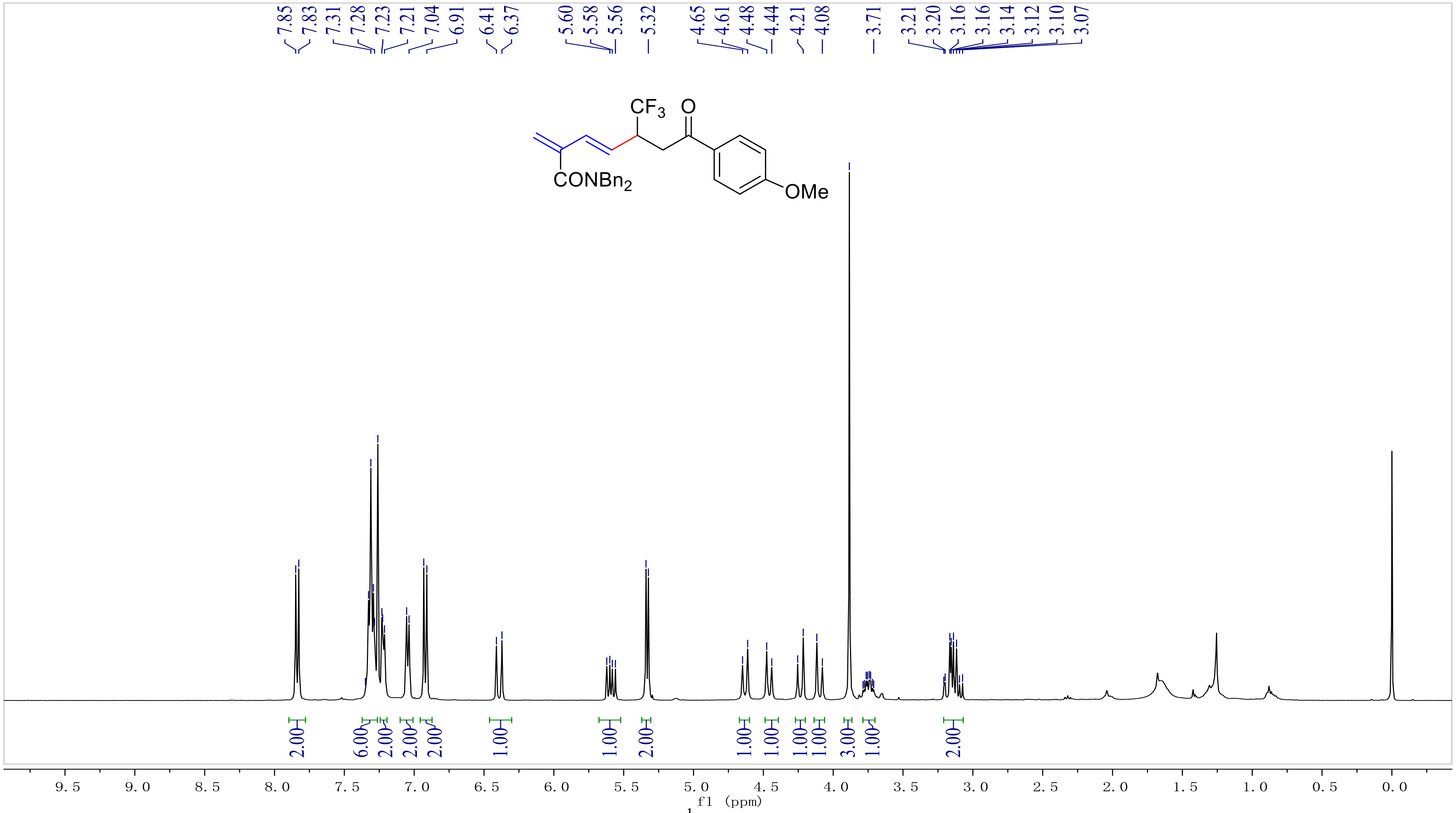

Supplementary Fig. 180 ^{1}H NMR (400 MHz, $CDCl_3$) of **5h**

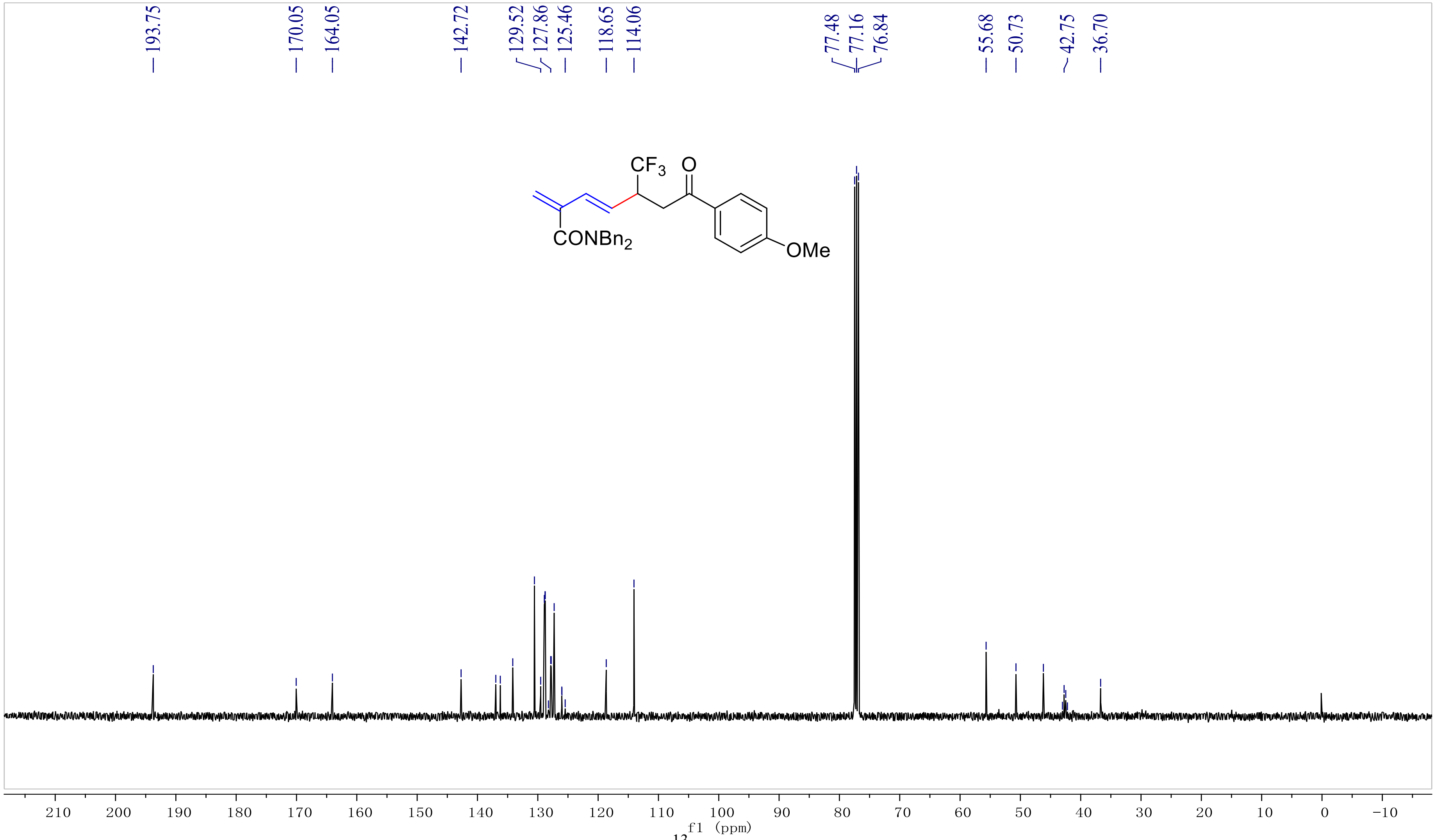

Supplementary Fig. 181 ^{13}C NMR (101 MHz, $CDCl_3$) of **5h**

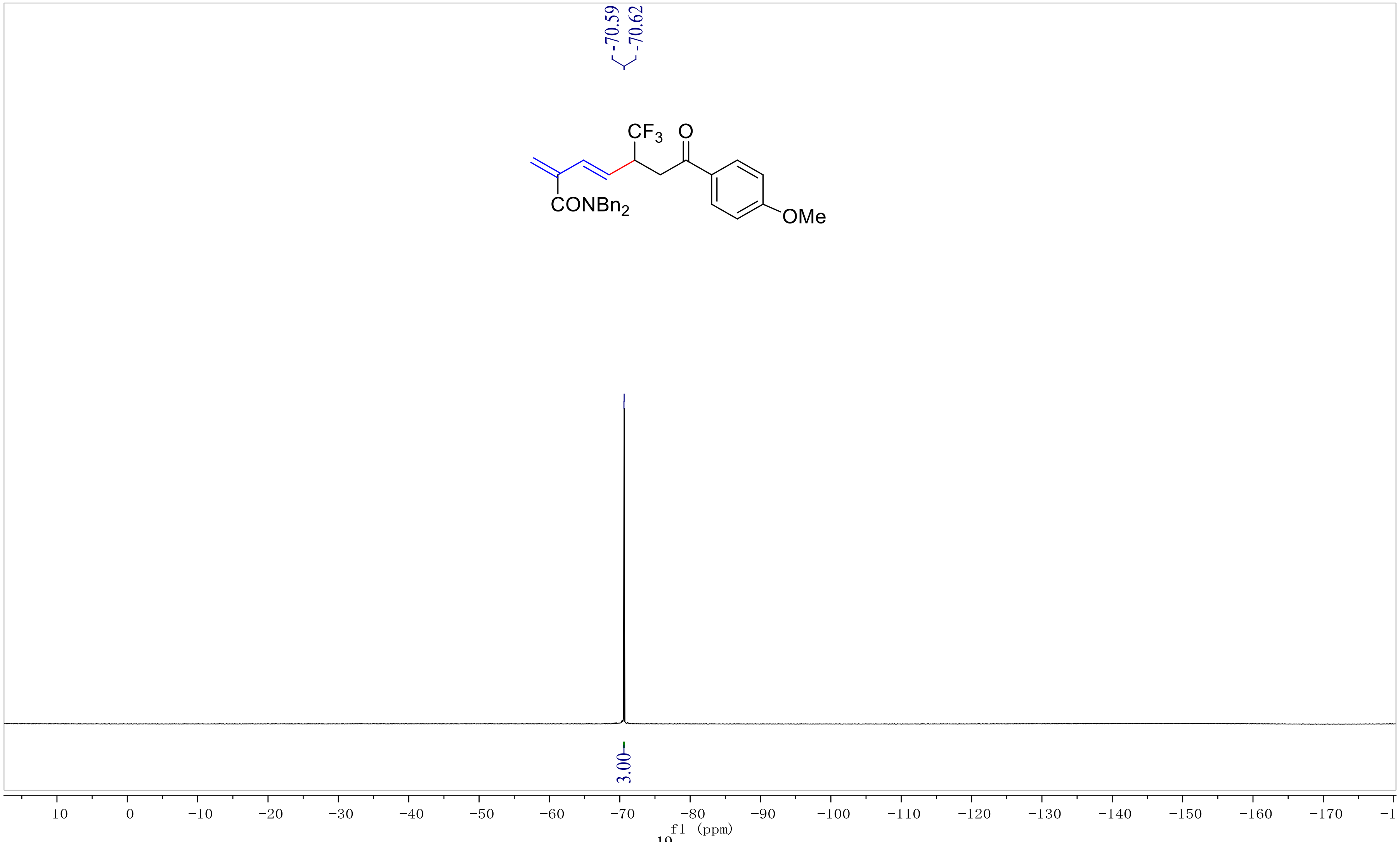

Supplementary Fig. 182 ^{19}F NMR (377 MHz, $CDCl_3$) of **5h**

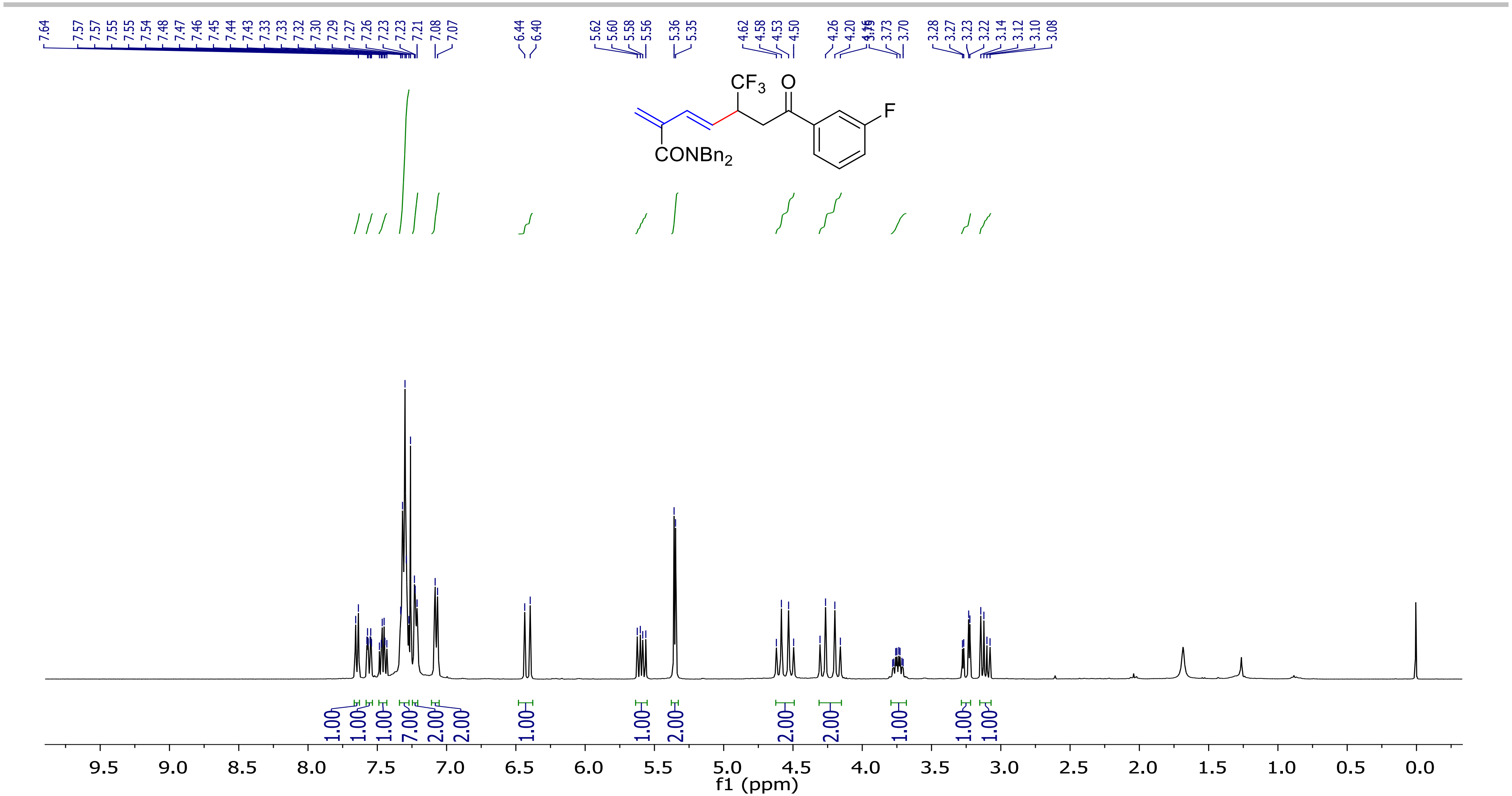

Supplementary Fig. 183 ^{1}H NMR (400 MHz, $CDCl_3$) of **5i**

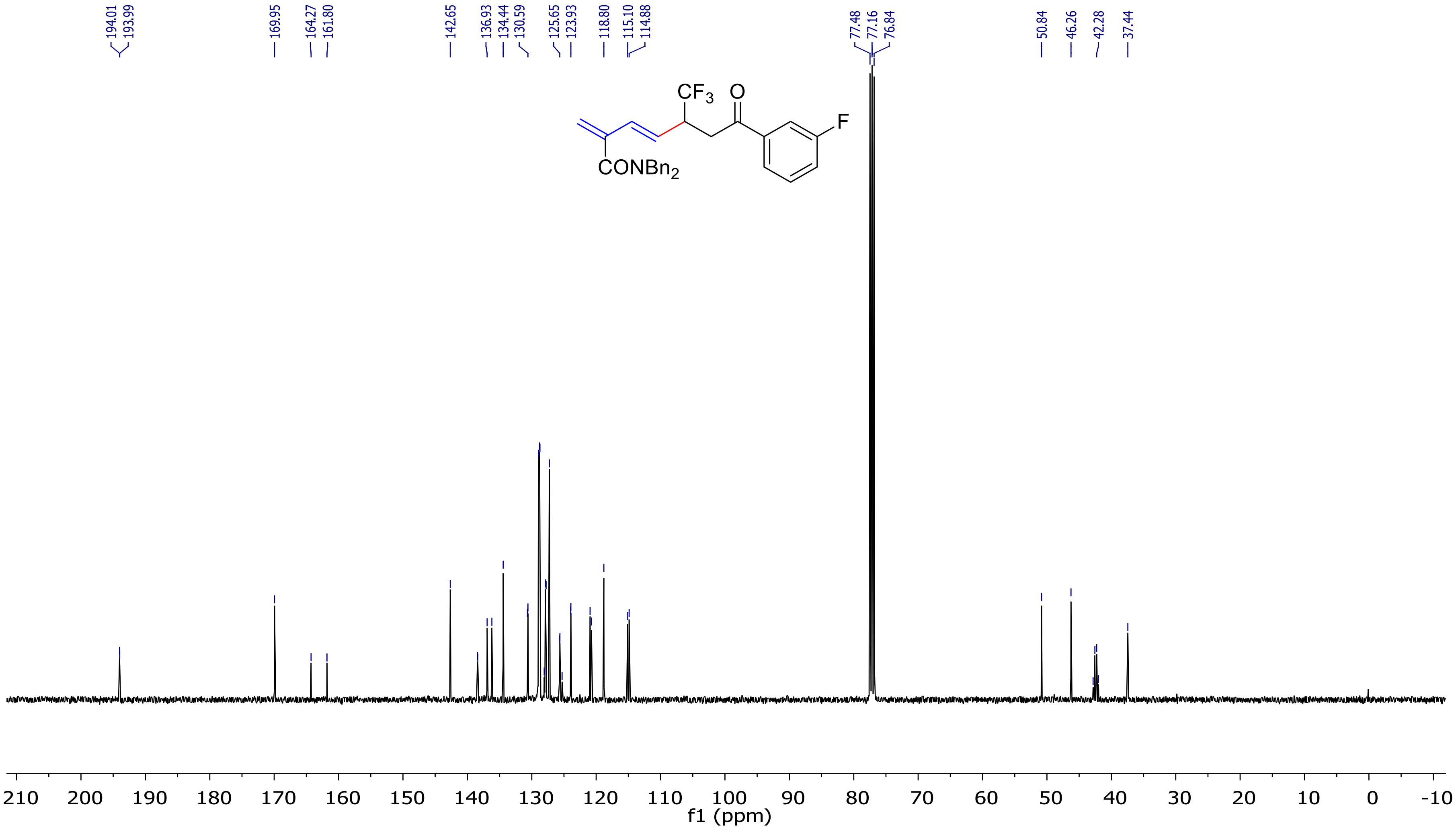

Supplementary Fig. 184 ^{13}C NMR (101 MHz, $CDCl_3$) of **5i**

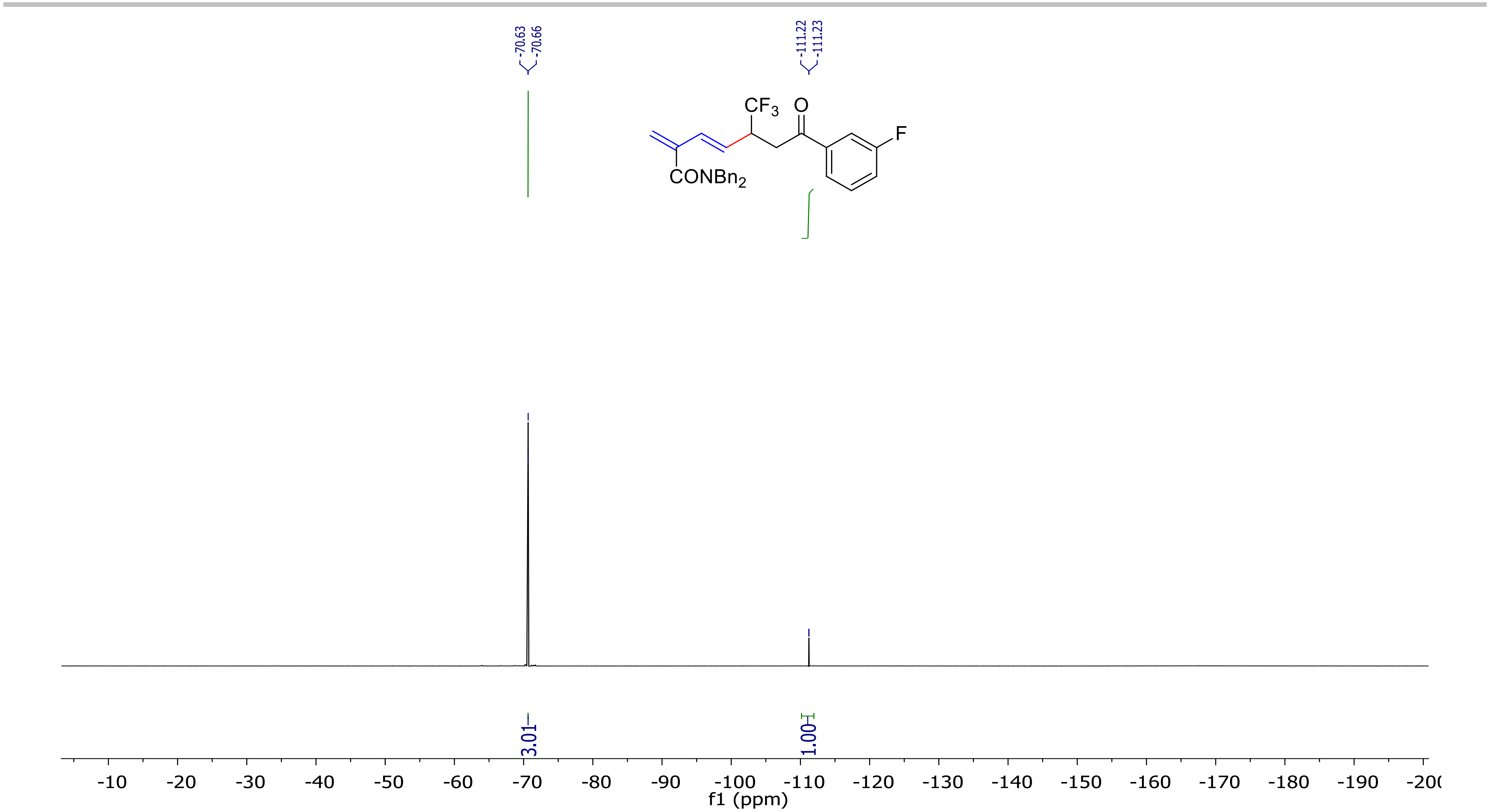

Supplementary Fig. 185 ^{19}F NMR (377 MHz, $CDCl_3$) of **5i**

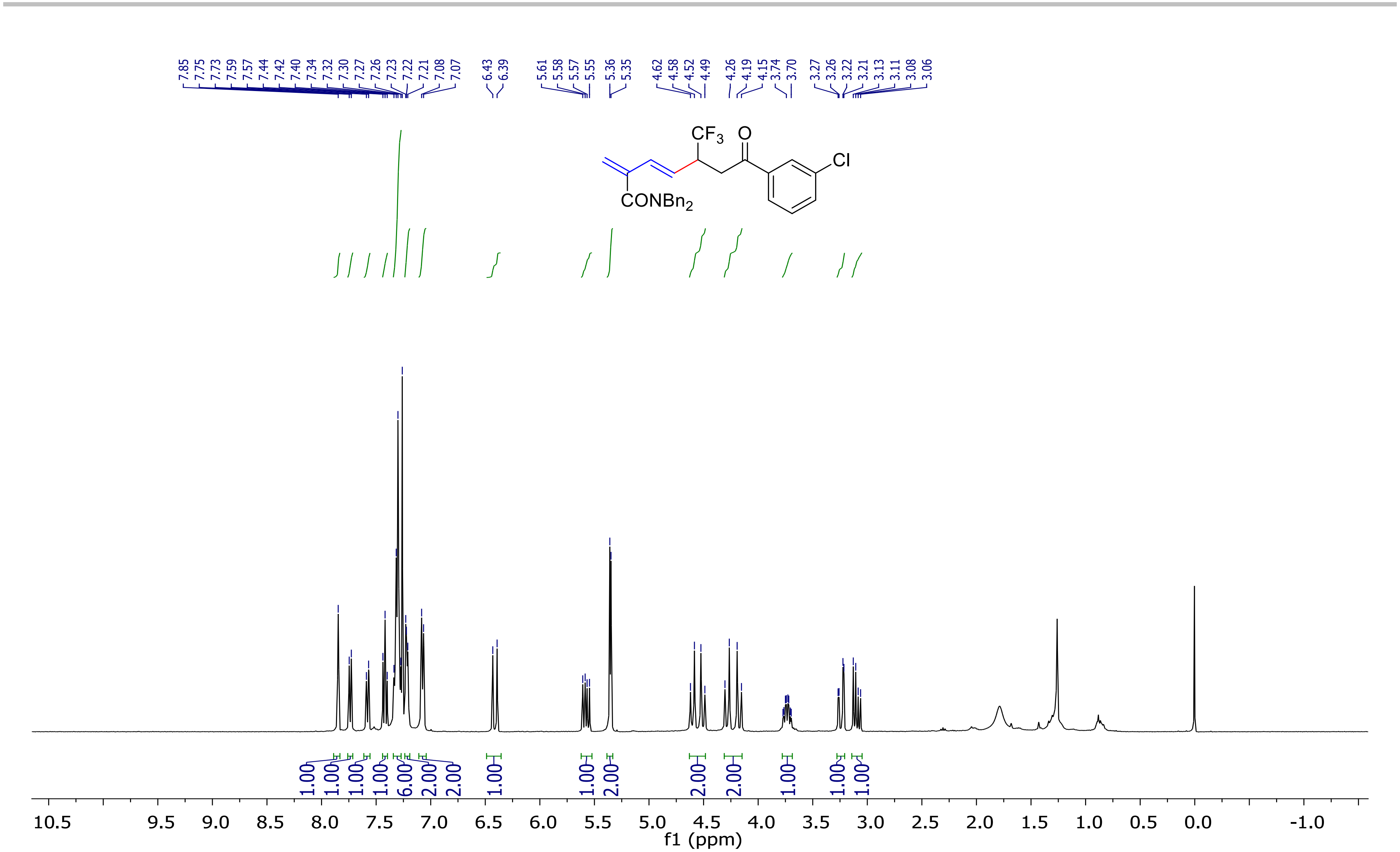

Supplementary Fig. 186 ^{1}H NMR (400 MHz, $CDCl_3$) of **5j**

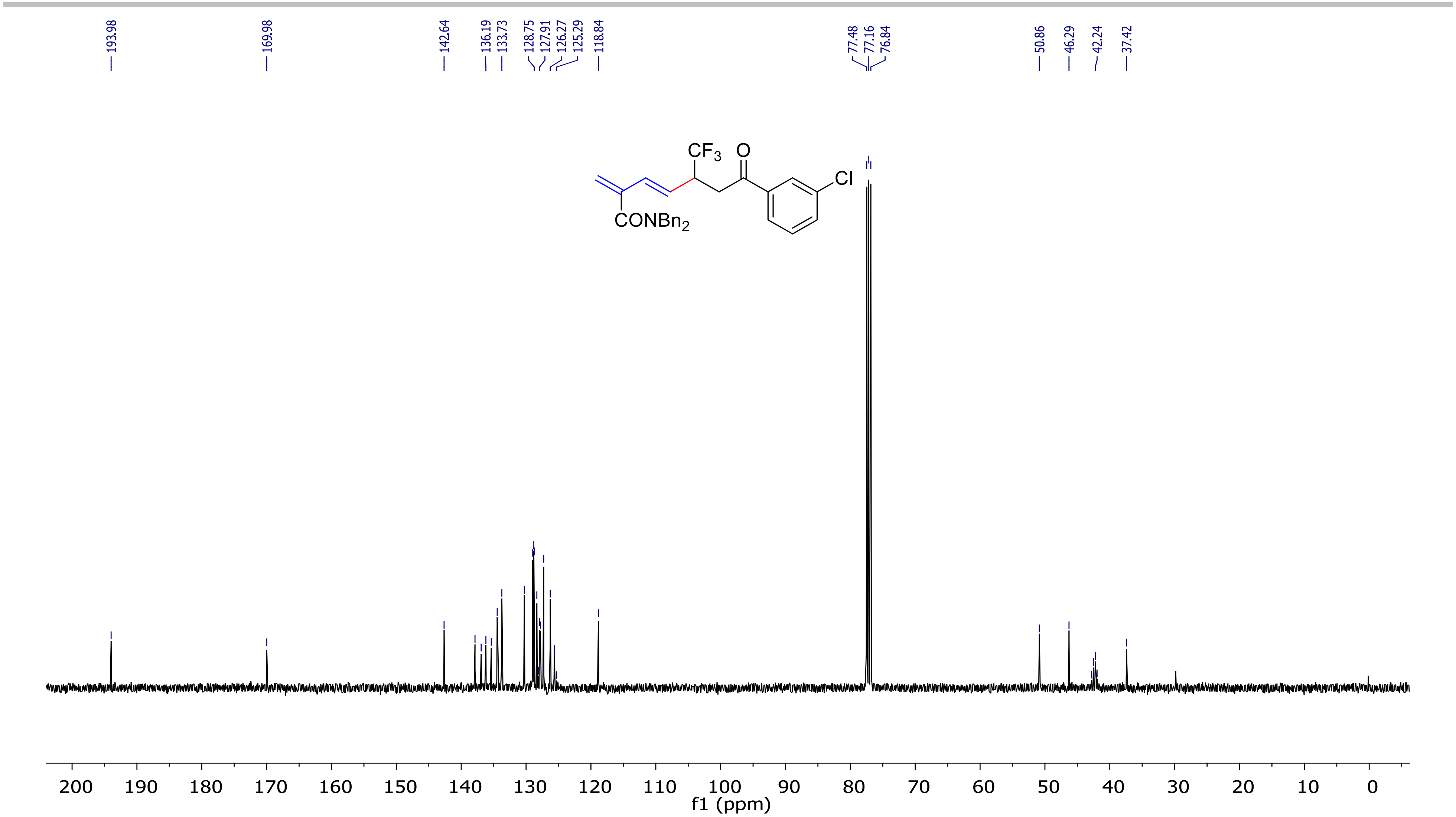

Supplementary Fig. 187 ^{13}C NMR (101 MHz, $CDCl_3$) of **5j**

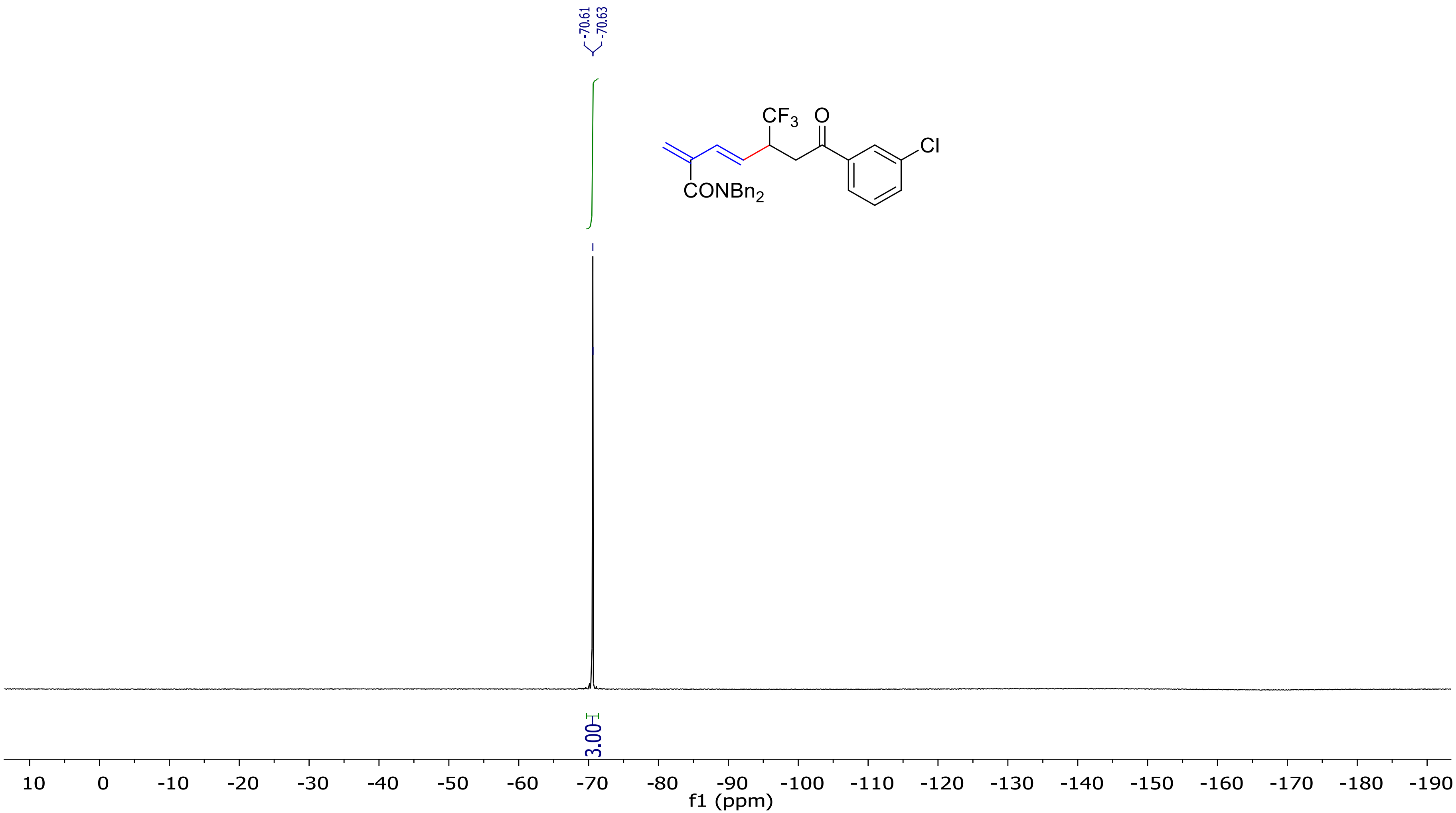

Supplementary Fig. 188 ^{19}F NMR (377 MHz, $CDCl_3$) of **5j**

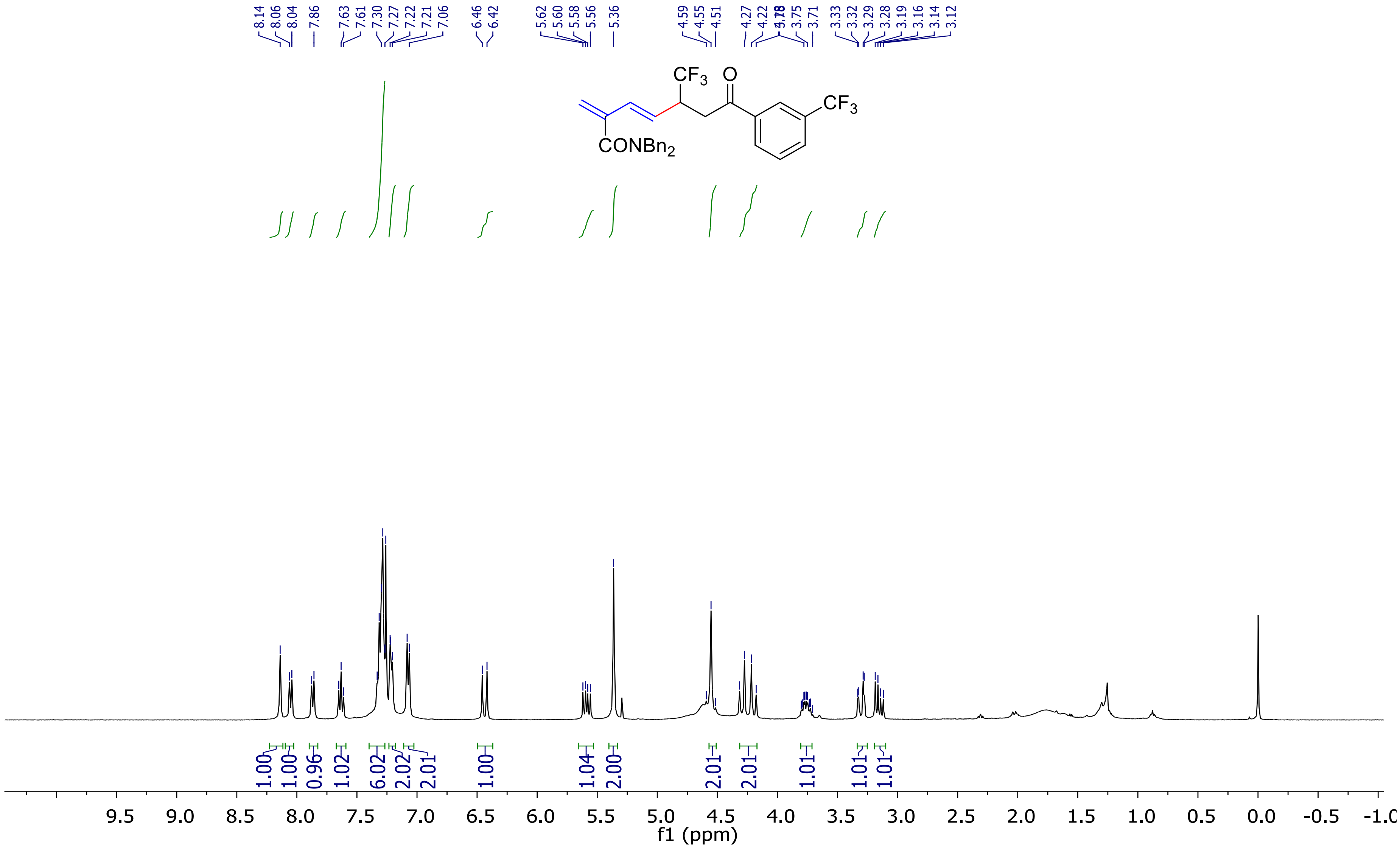

Supplementary Fig. 189 ^{1}H NMR (400 MHz, $CDCl_3$) of **5k**

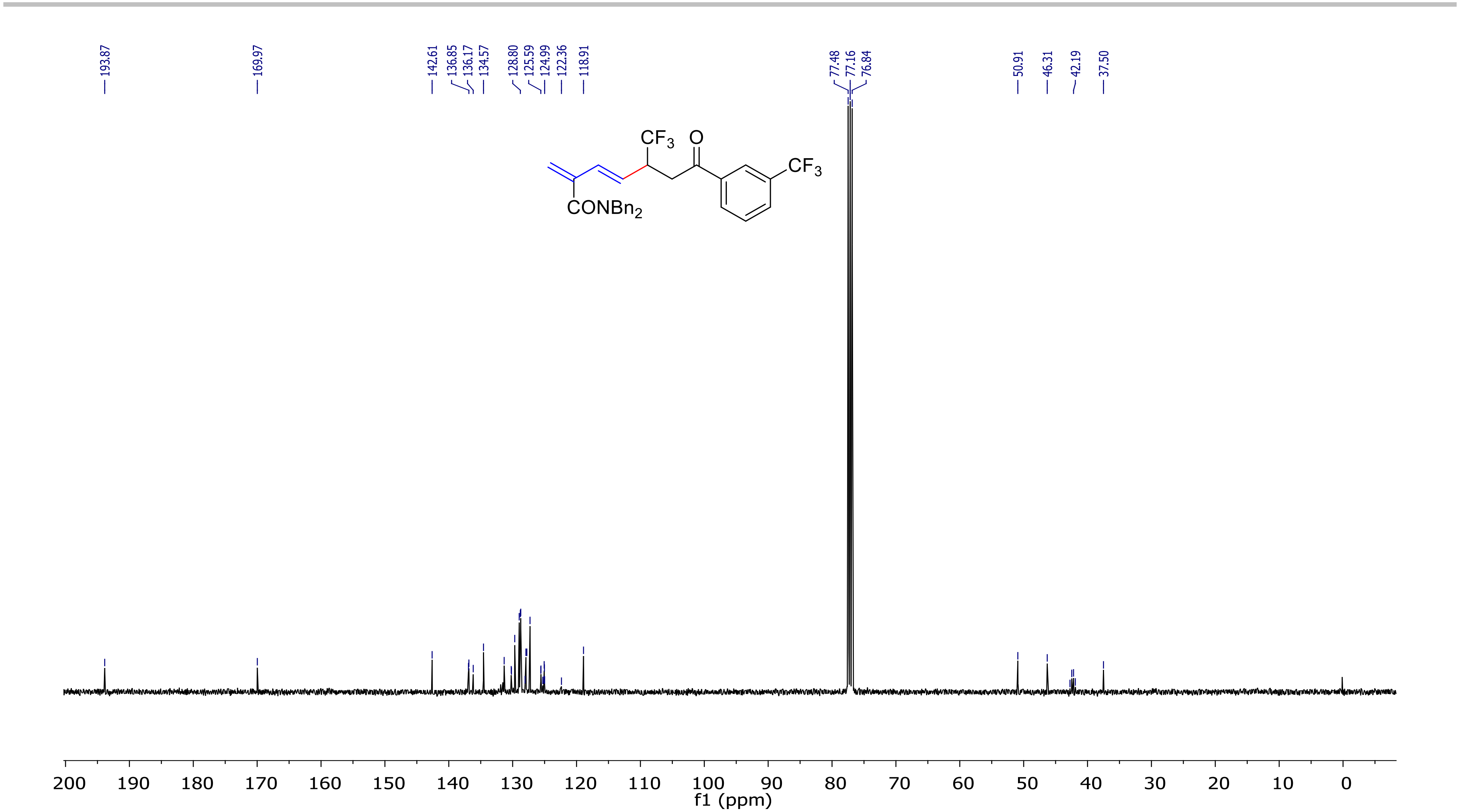

Supplementary Fig. 190 ^{13}C NMR (101 MHz, $CDCl_3$) of **5k**

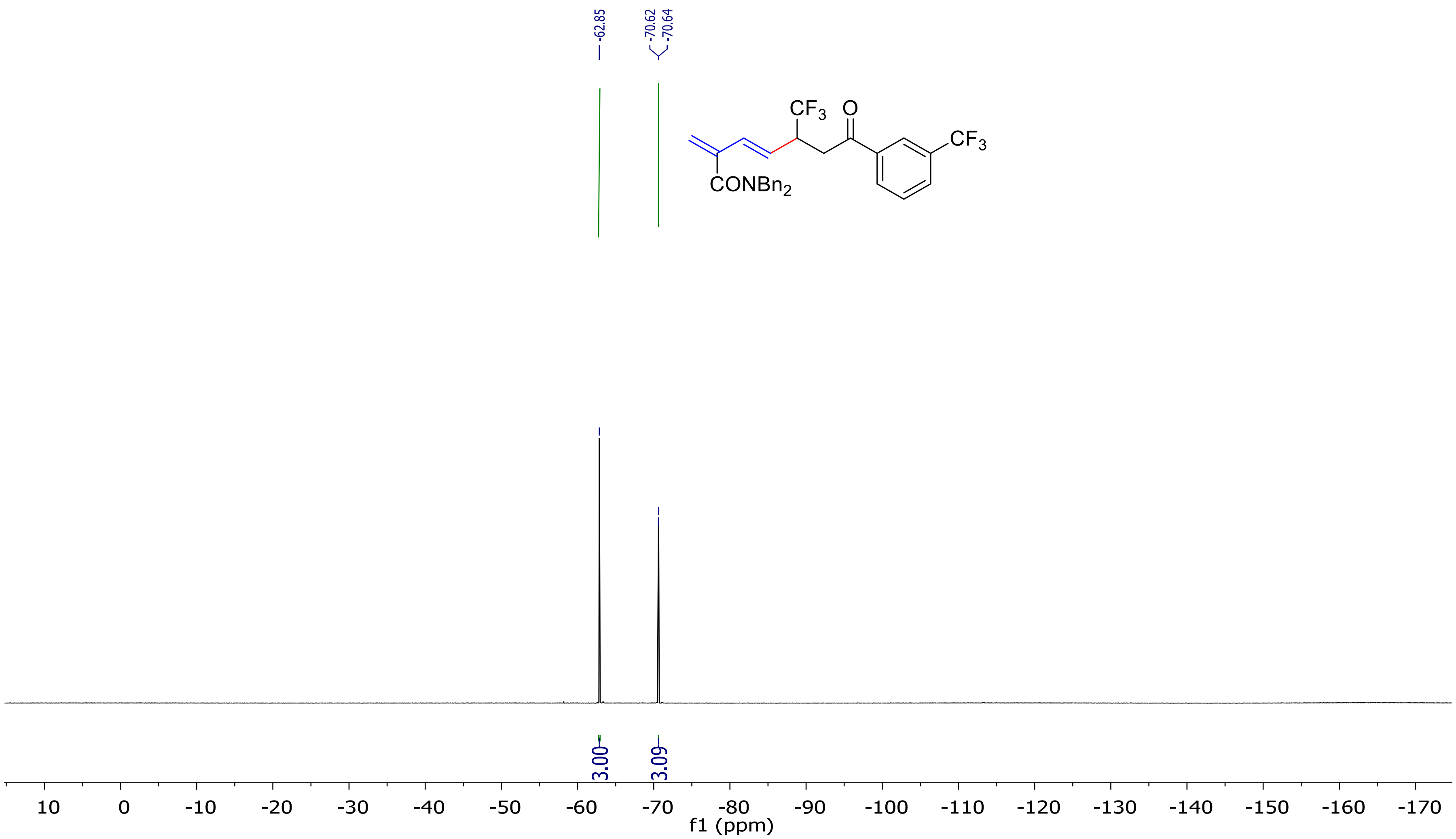

Supplementary Fig. 191 ^{19}F NMR (377 MHz, $CDCl_3$) of **5k**

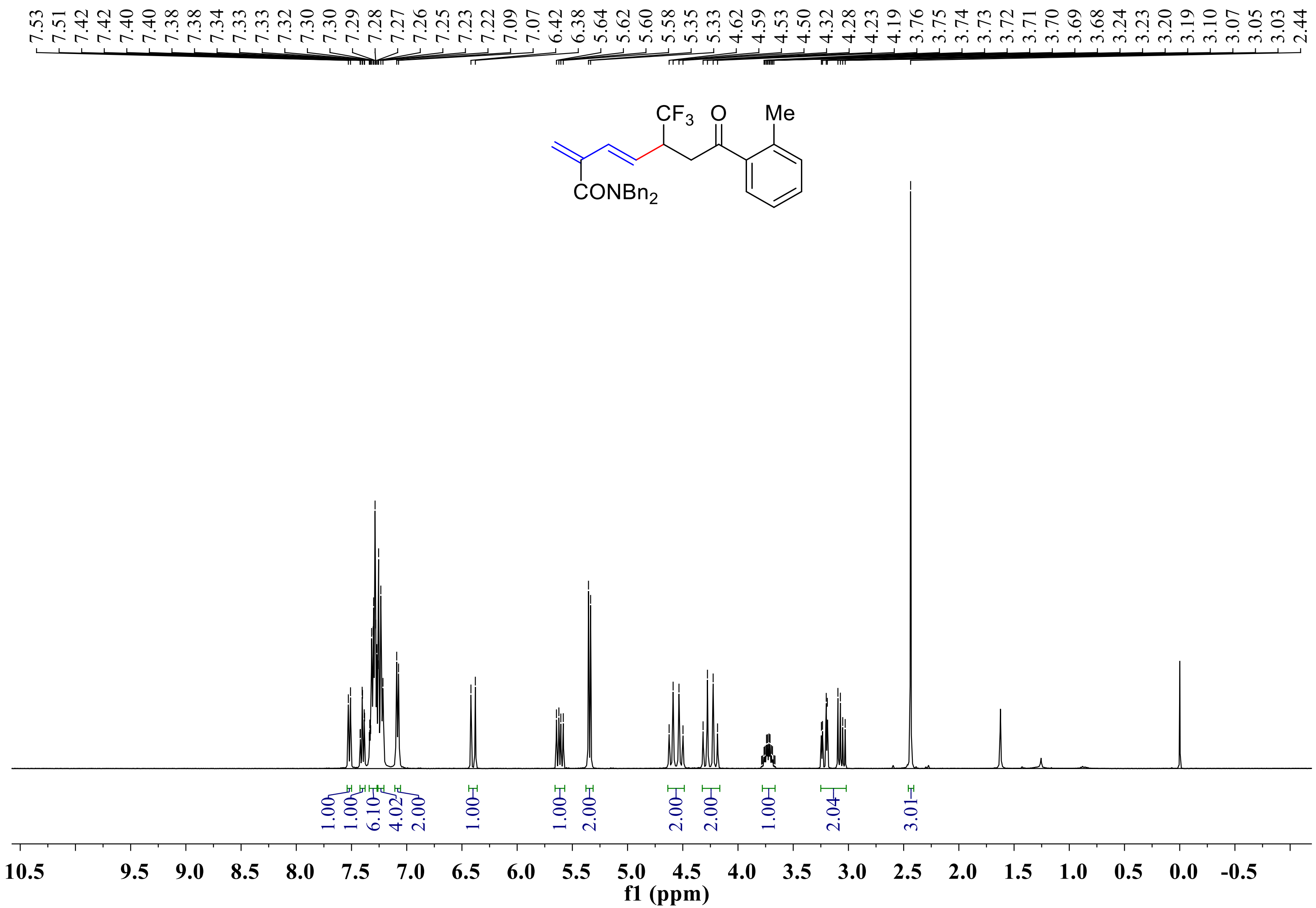

Supplementary Fig. 192 ^{1}H NMR (400 MHz, $CDCl_3$) of **5l**

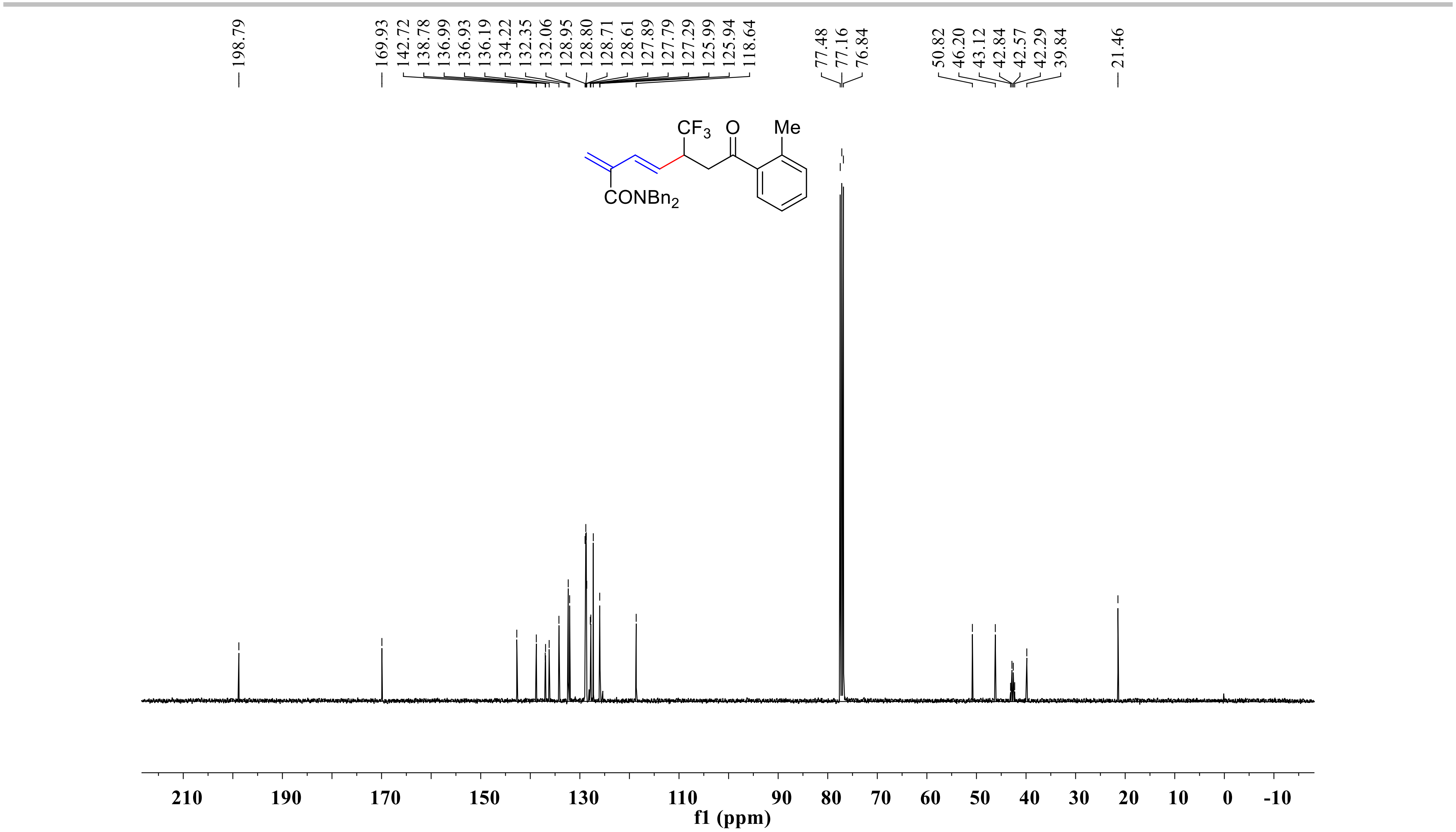

Supplementary Fig. 193 ^{13}C NMR (101 MHz, $CDCl_3$) of **5l**

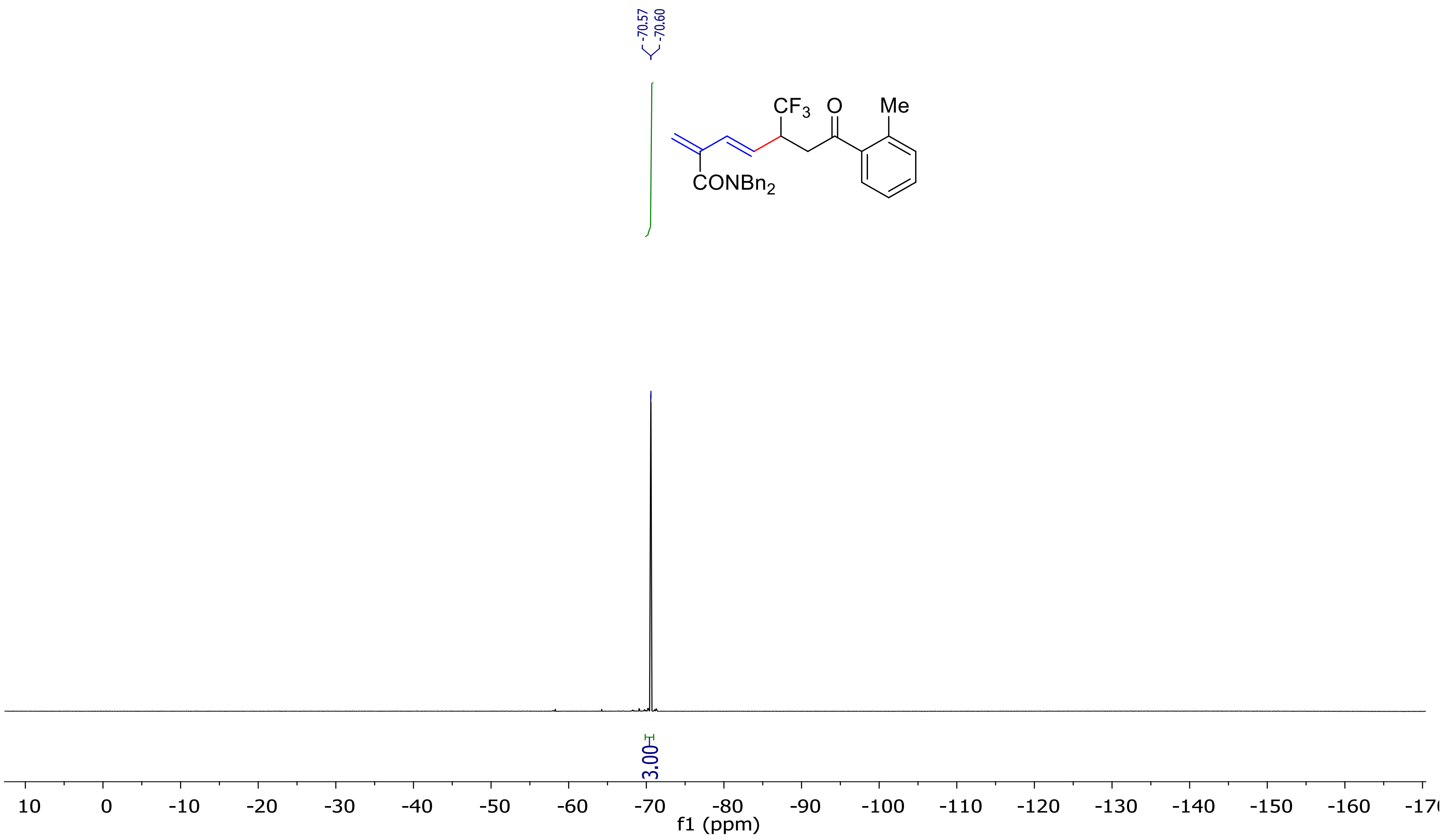

Supplementary Fig. 194 ^{19}F NMR (377 MHz, $CDCl_3$) of **5l**

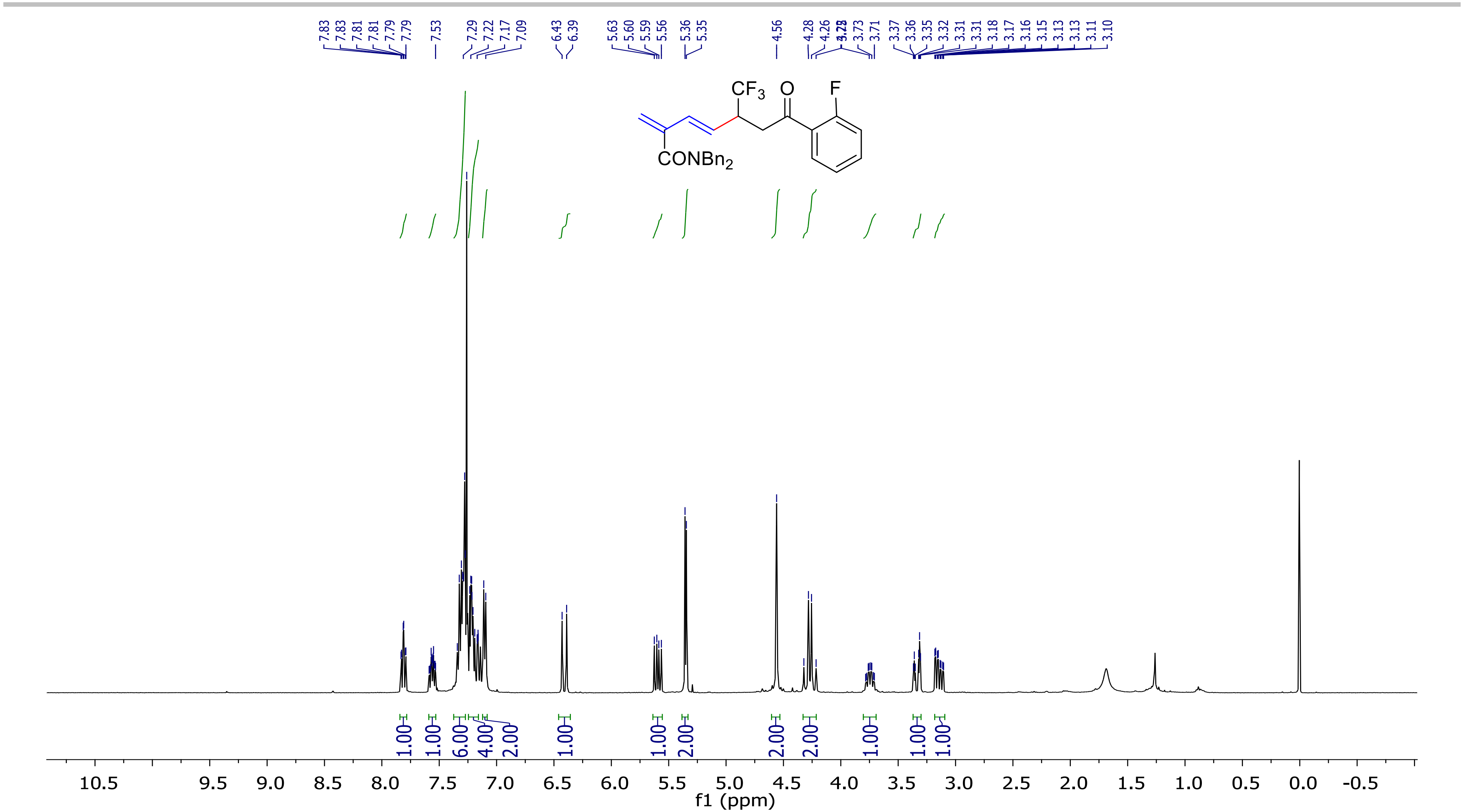

Supplementary Fig. 195 ^{1}H NMR (400 MHz, $CDCl_3$) of **5m**

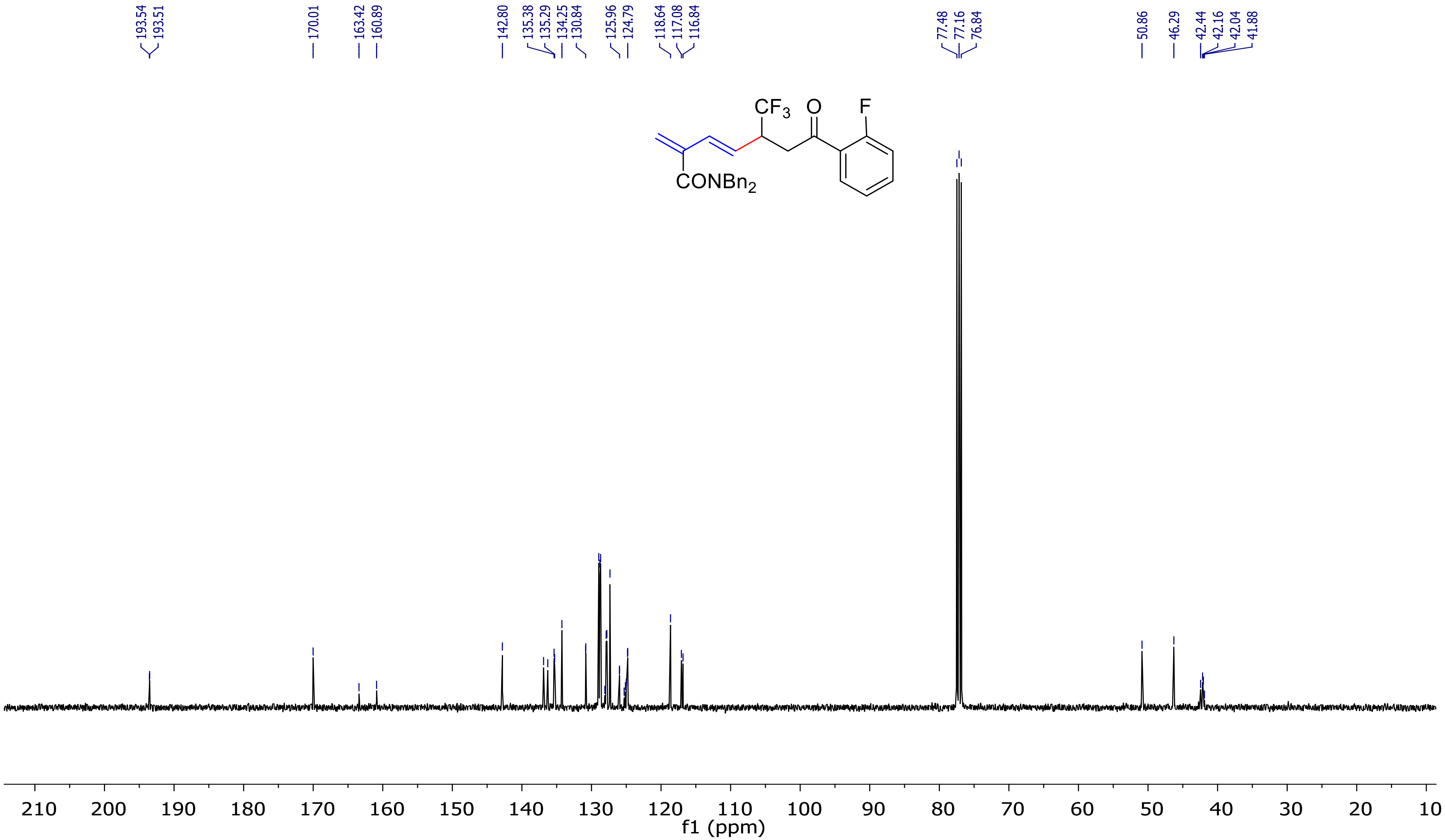

Supplementary Fig. 196 ^{13}C NMR (101 MHz, $CDCl_3$) of **5m**

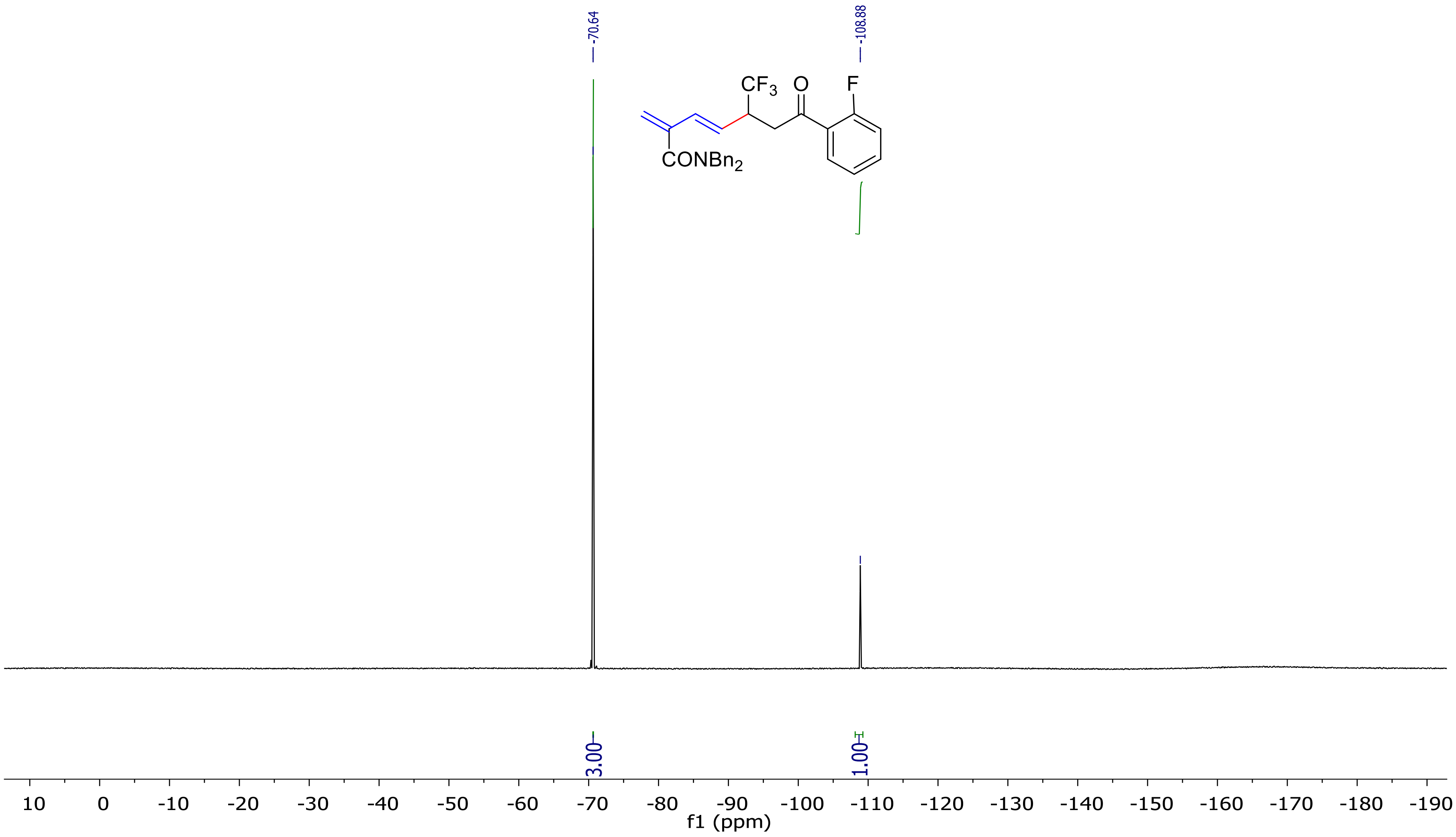

Supplementary Fig. 197 ^{19}F NMR (377 MHz, $CDCl_3$) of **5m**

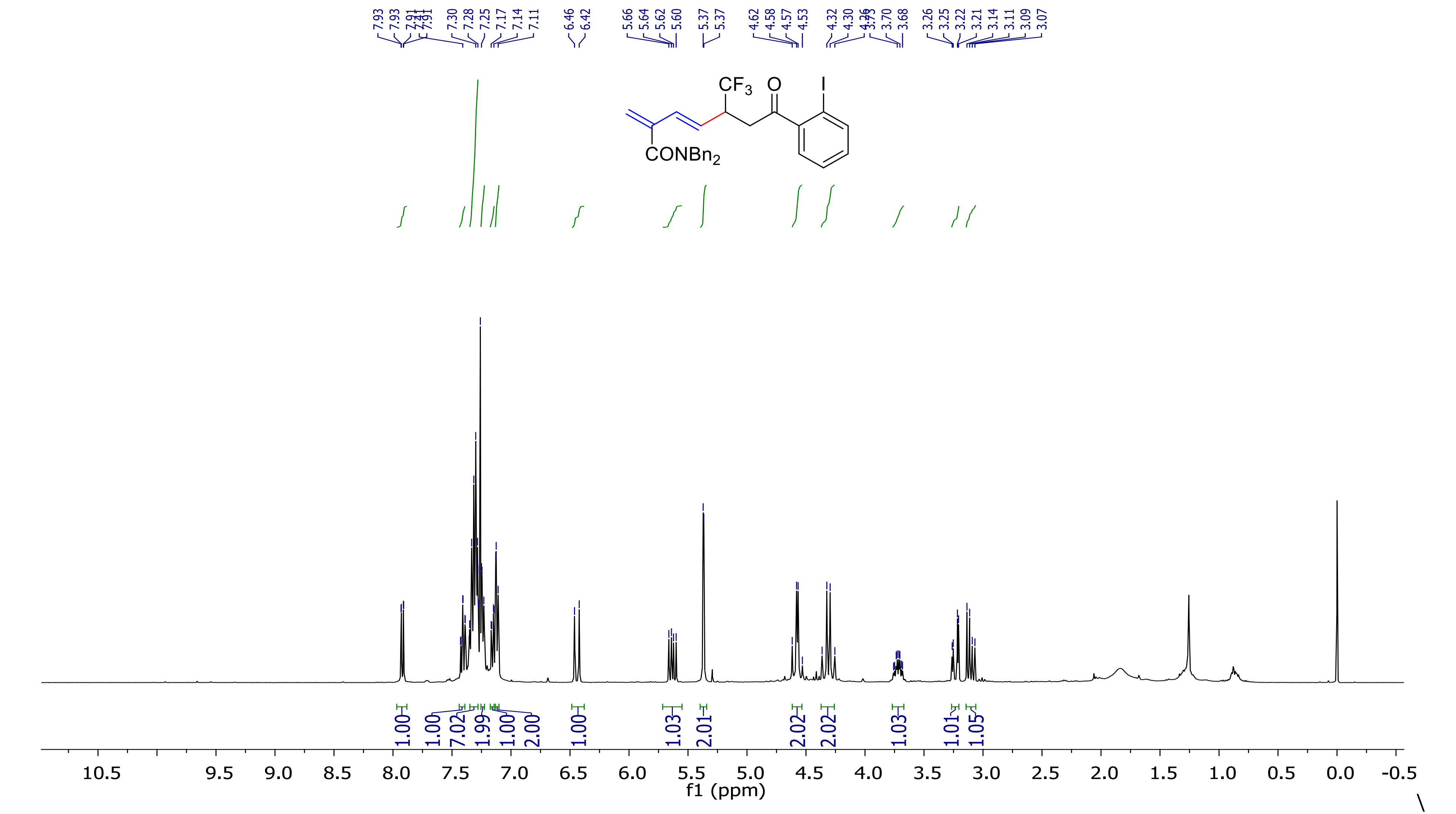

Supplementary Fig. 198 ^{1}H NMR (400 MHz, $CDCl_3$) of **5n**

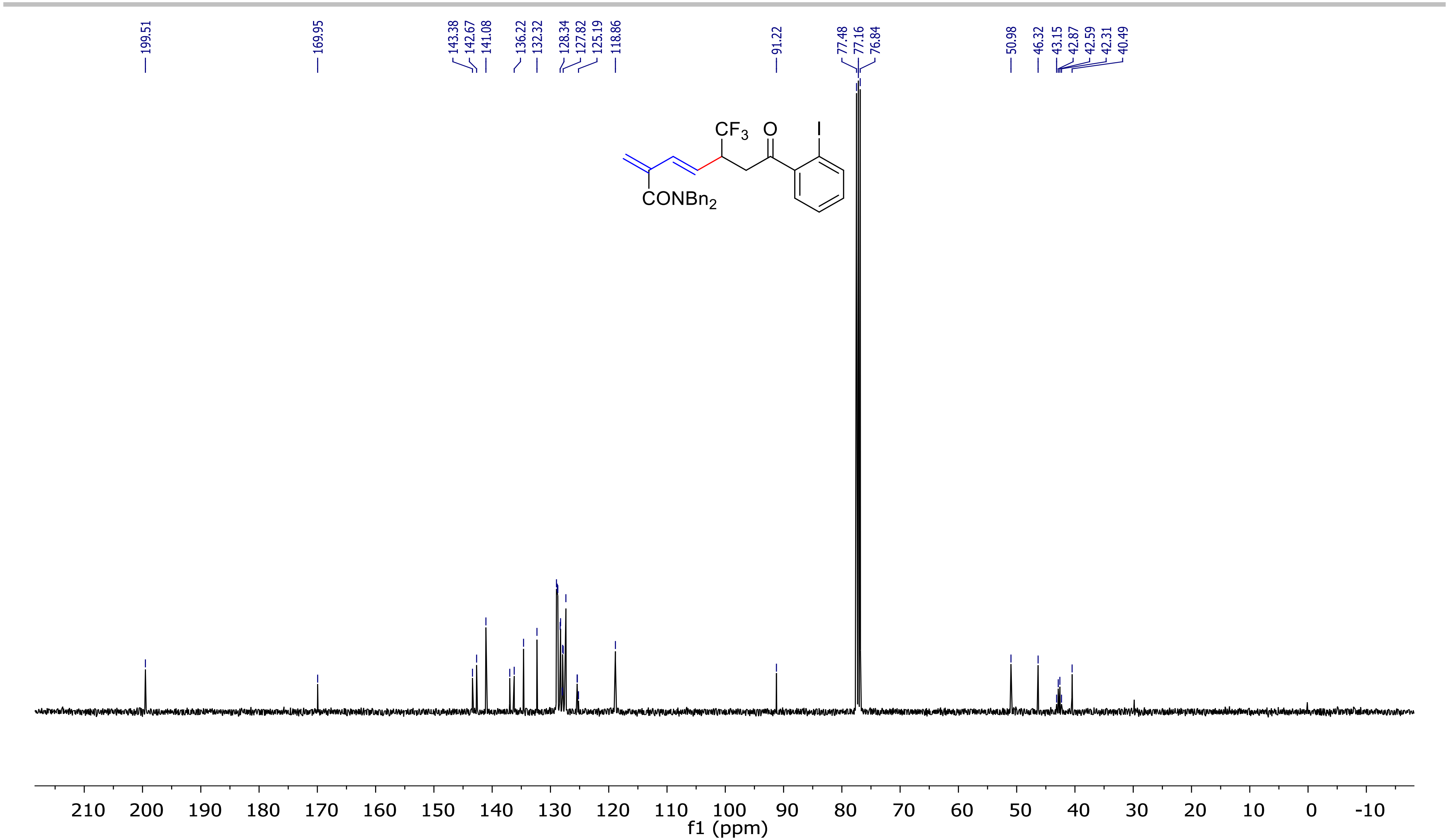

Supplementary Fig. 199 ^{13}C NMR (101 MHz, $CDCl_3$) of **5n**

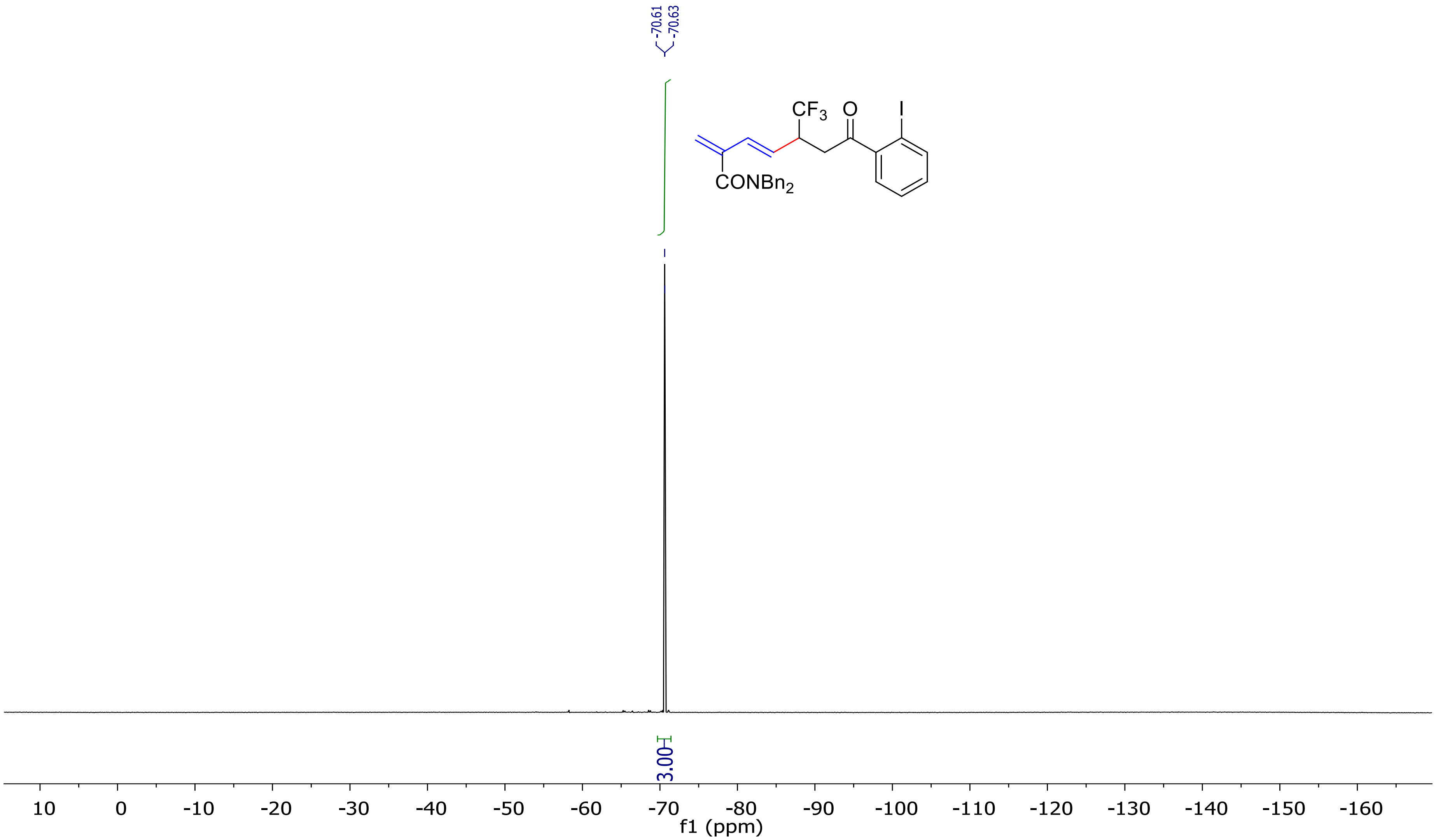

Supplementary Fig. 200 ^{19}F NMR (377 MHz, $CDCl_3$) of **5n**

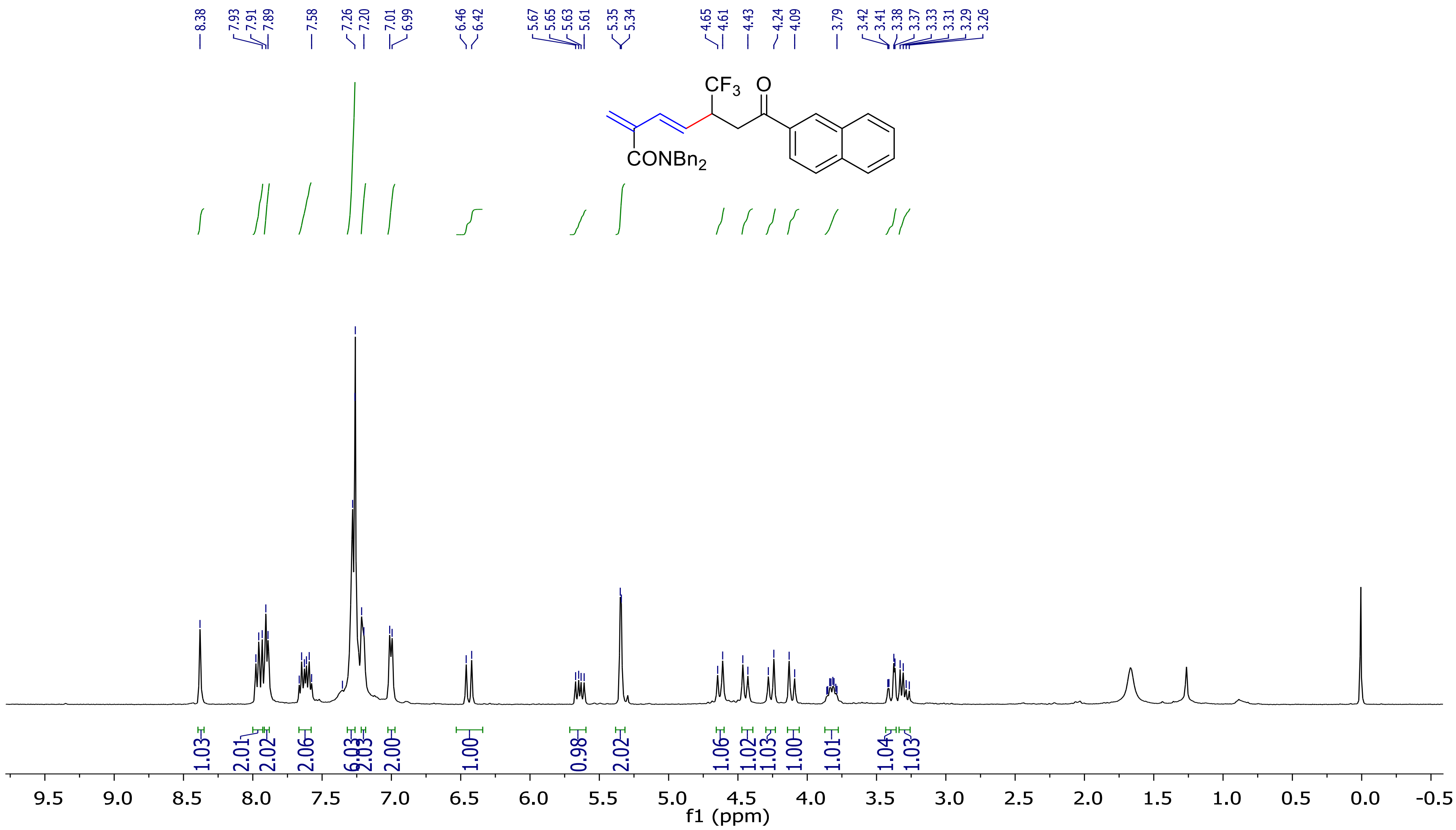

Supplementary Fig. 201 ^{1}H NMR (400 MHz, $CDCl_3$) of **5o**

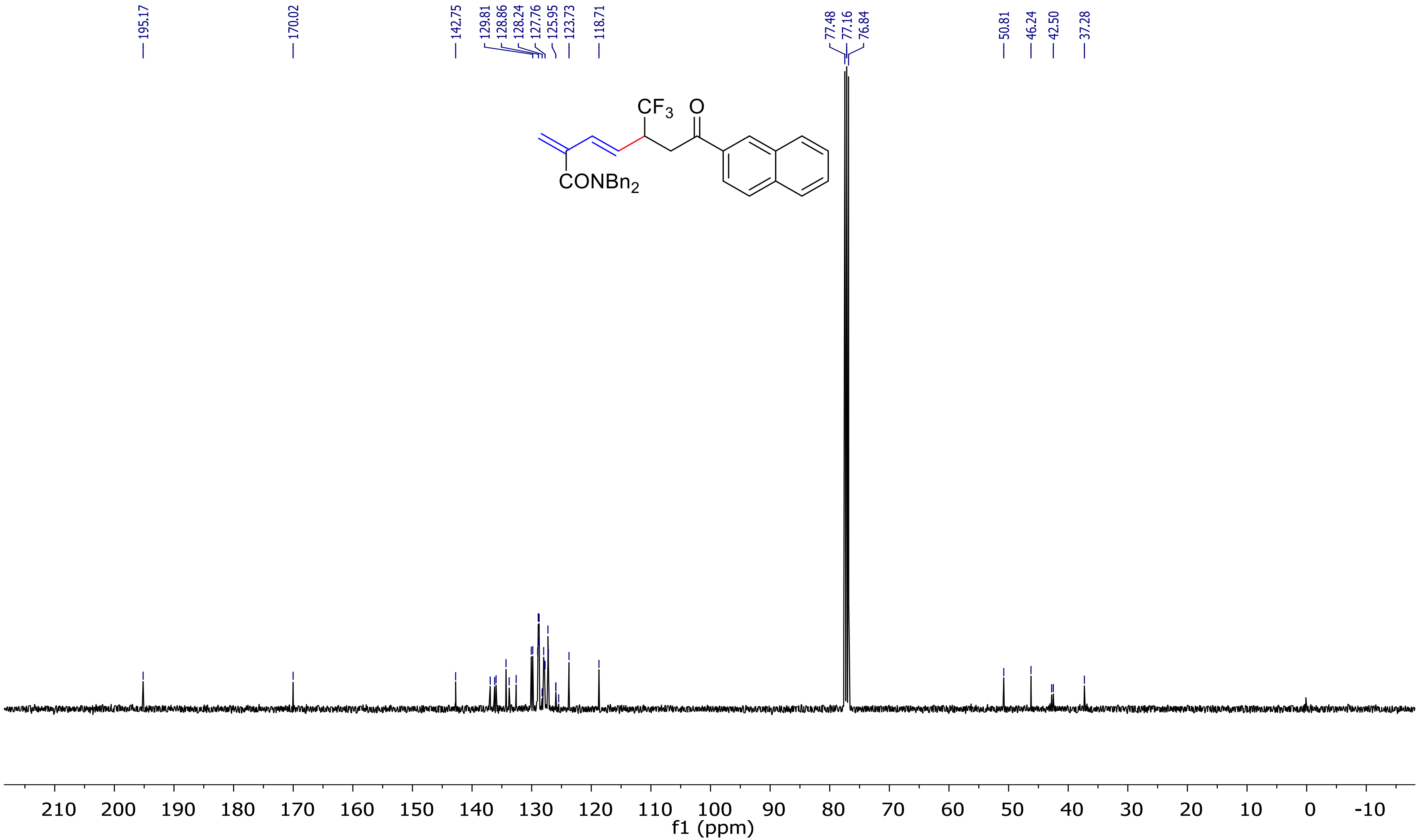

Supplementary Fig. 202 ^{13}C NMR (101 MHz, $CDCl_3$) of **5o**

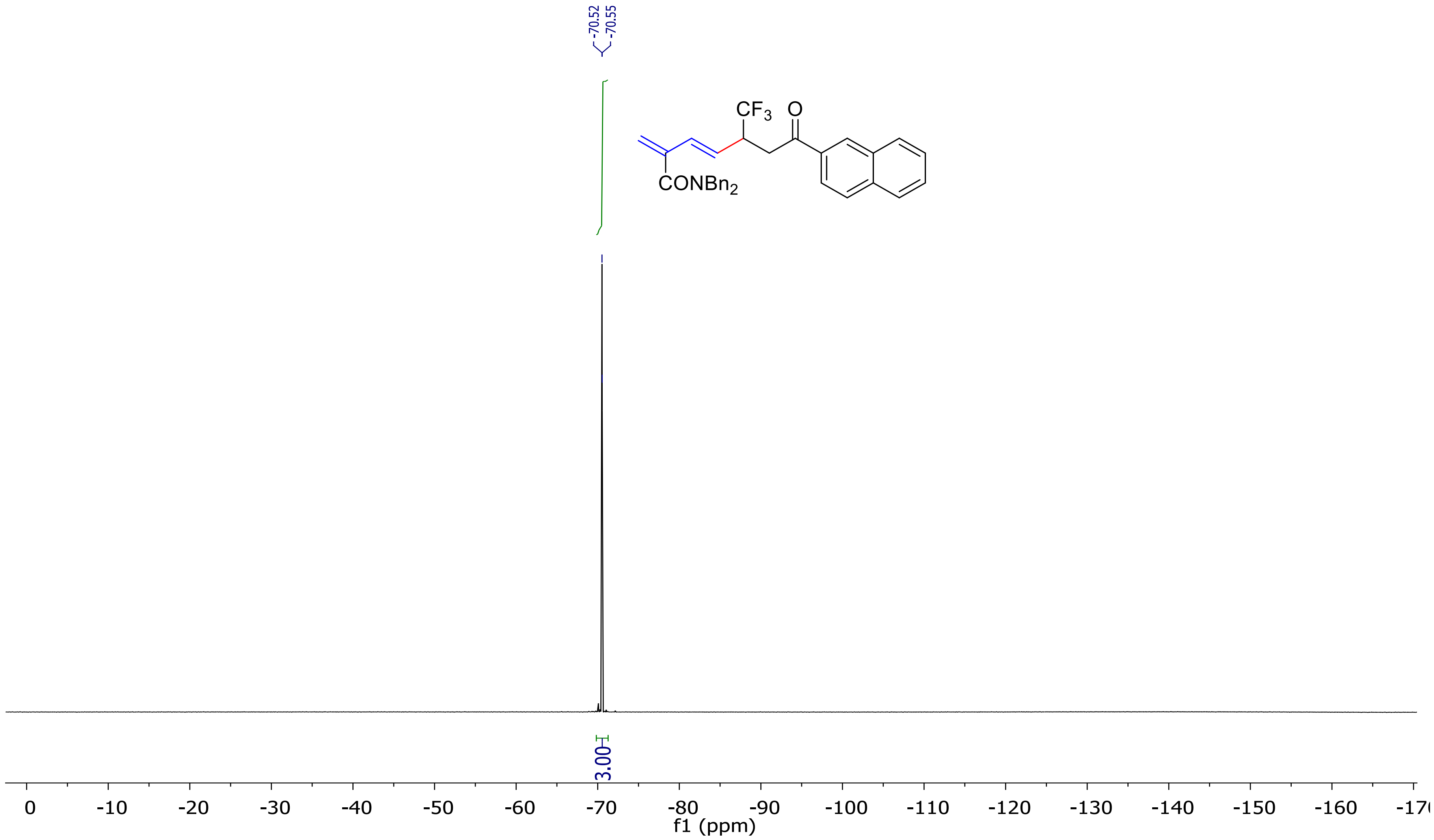

Supplementary Fig. 203 ^{19}F NMR (377 MHz, $CDCl_3$) of **5o**

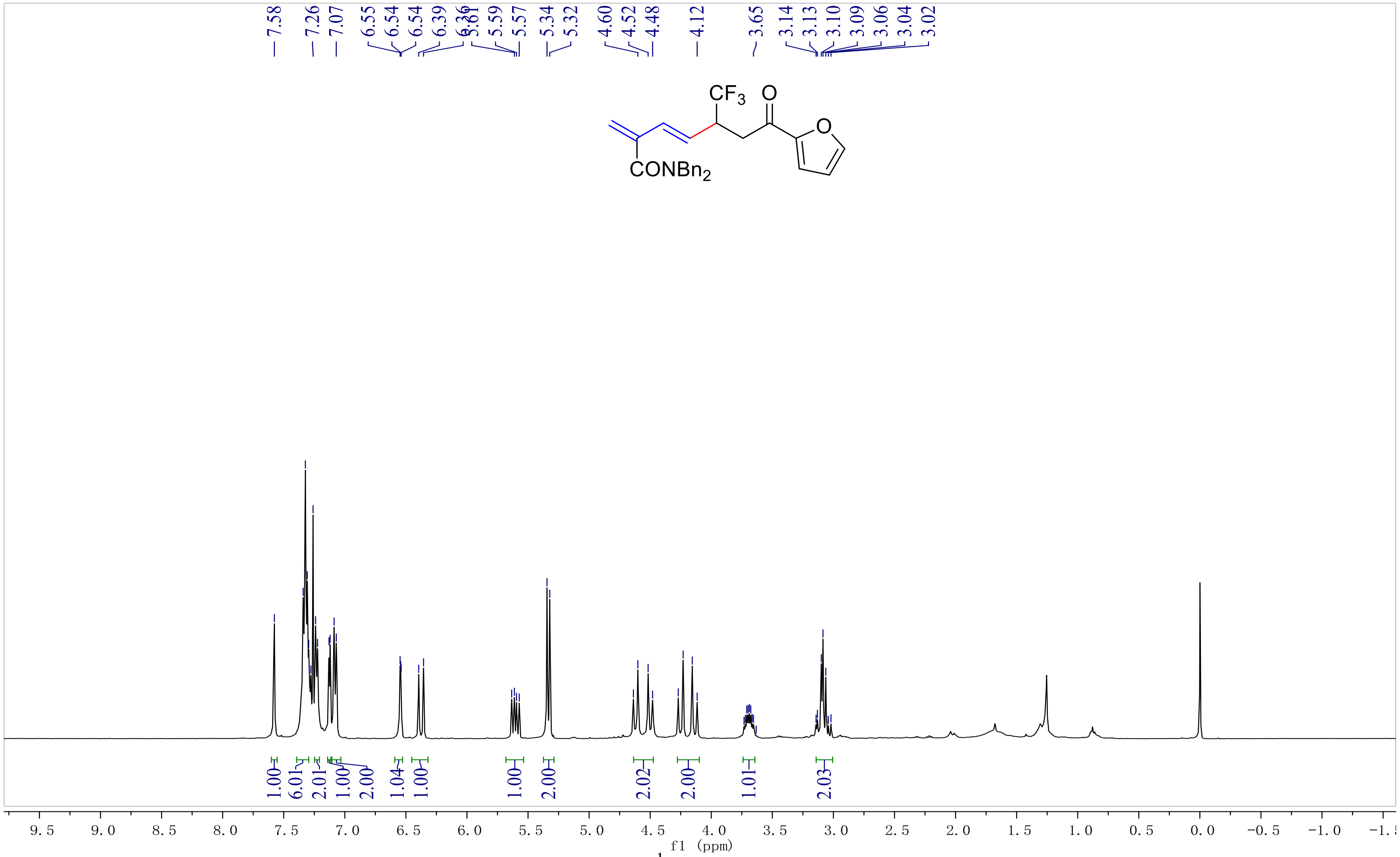

Supplementary Fig. 204 ^{1}H NMR (400 MHz, $CDCl_3$) of **5p**

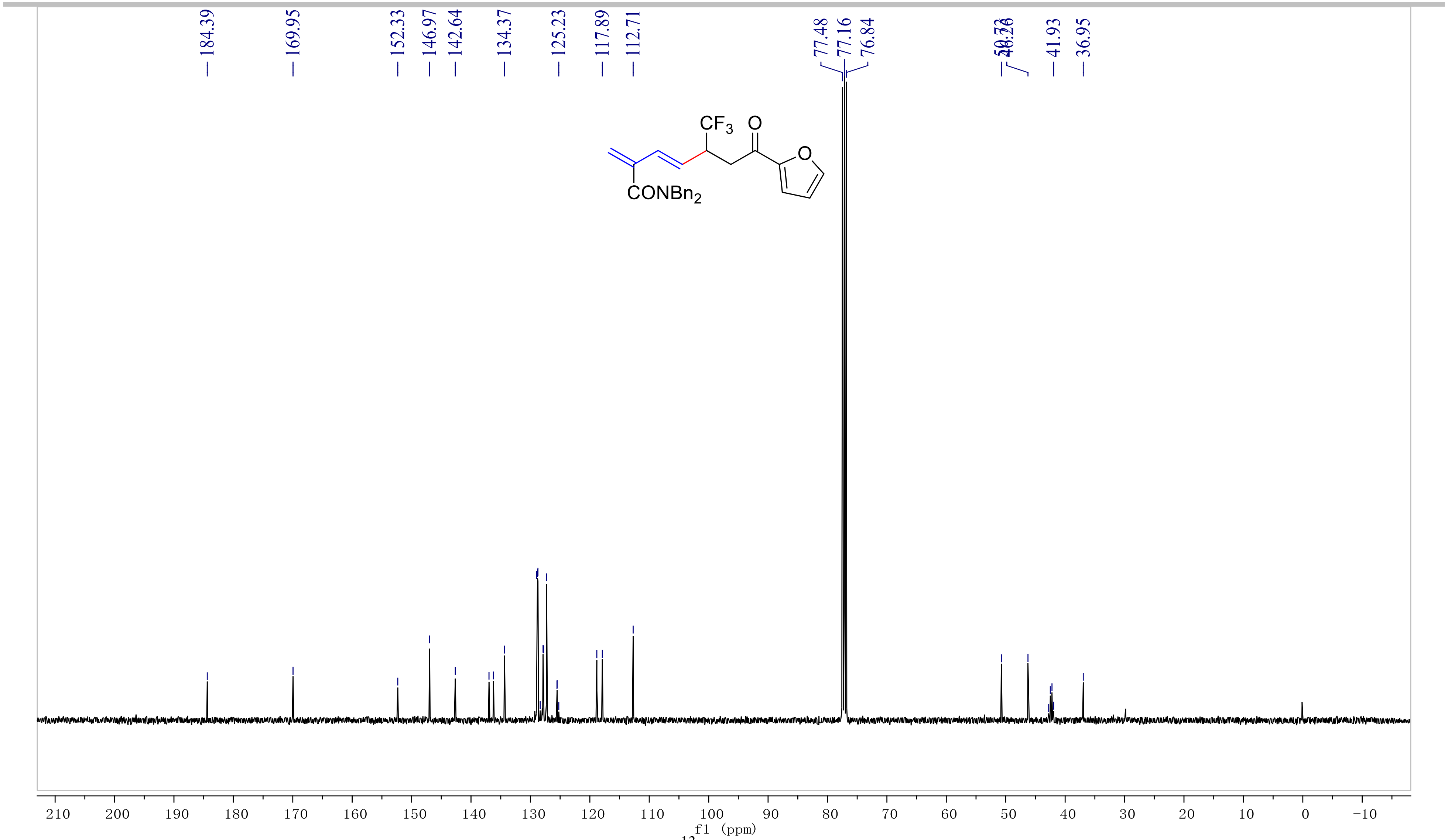

Supplementary Fig. 205 ^{13}C NMR (101 MHz, $CDCl_3$) of **5p**

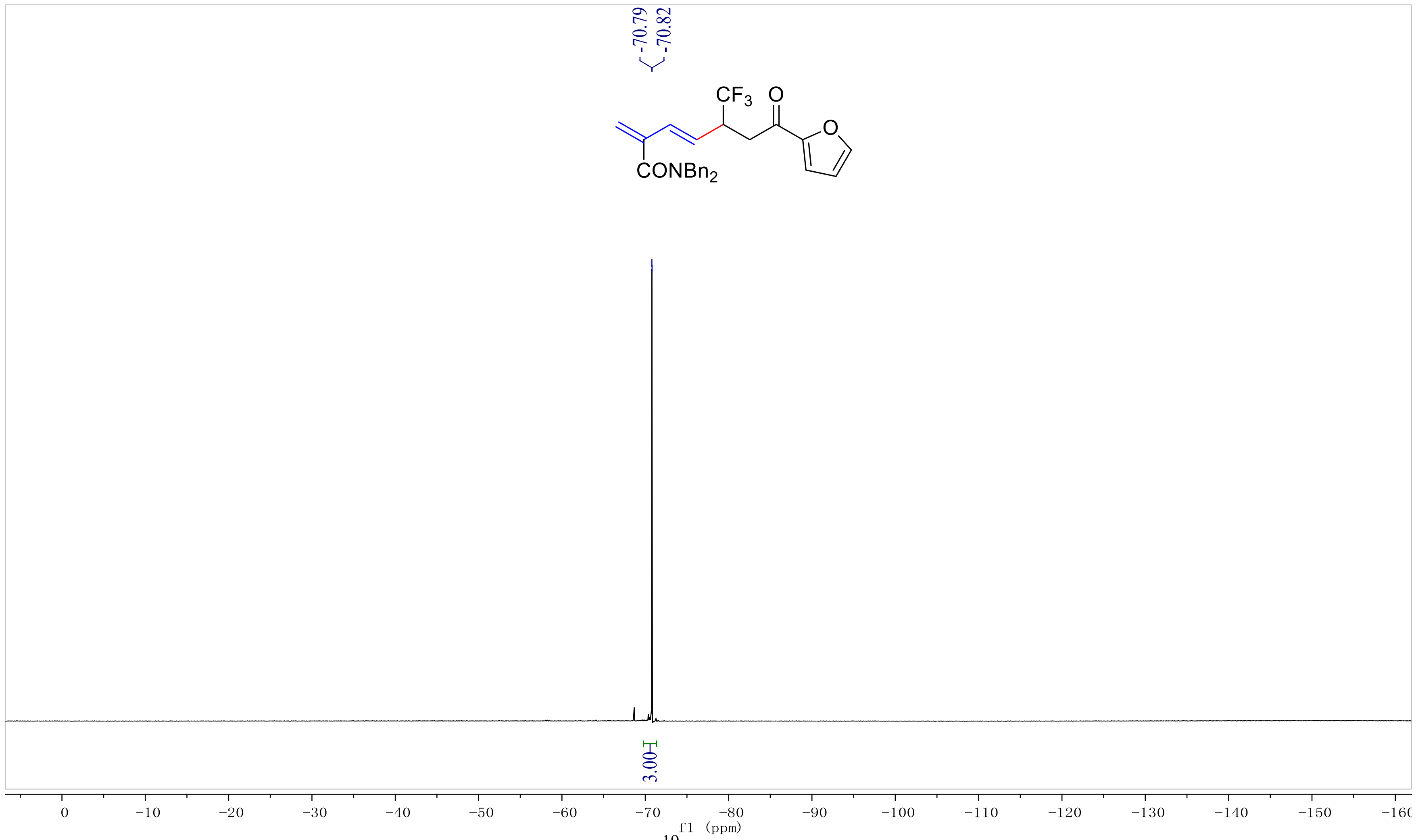

Supplementary Fig. 206 ^{19}F NMR (377 MHz, $CDCl_3$) of **5p**

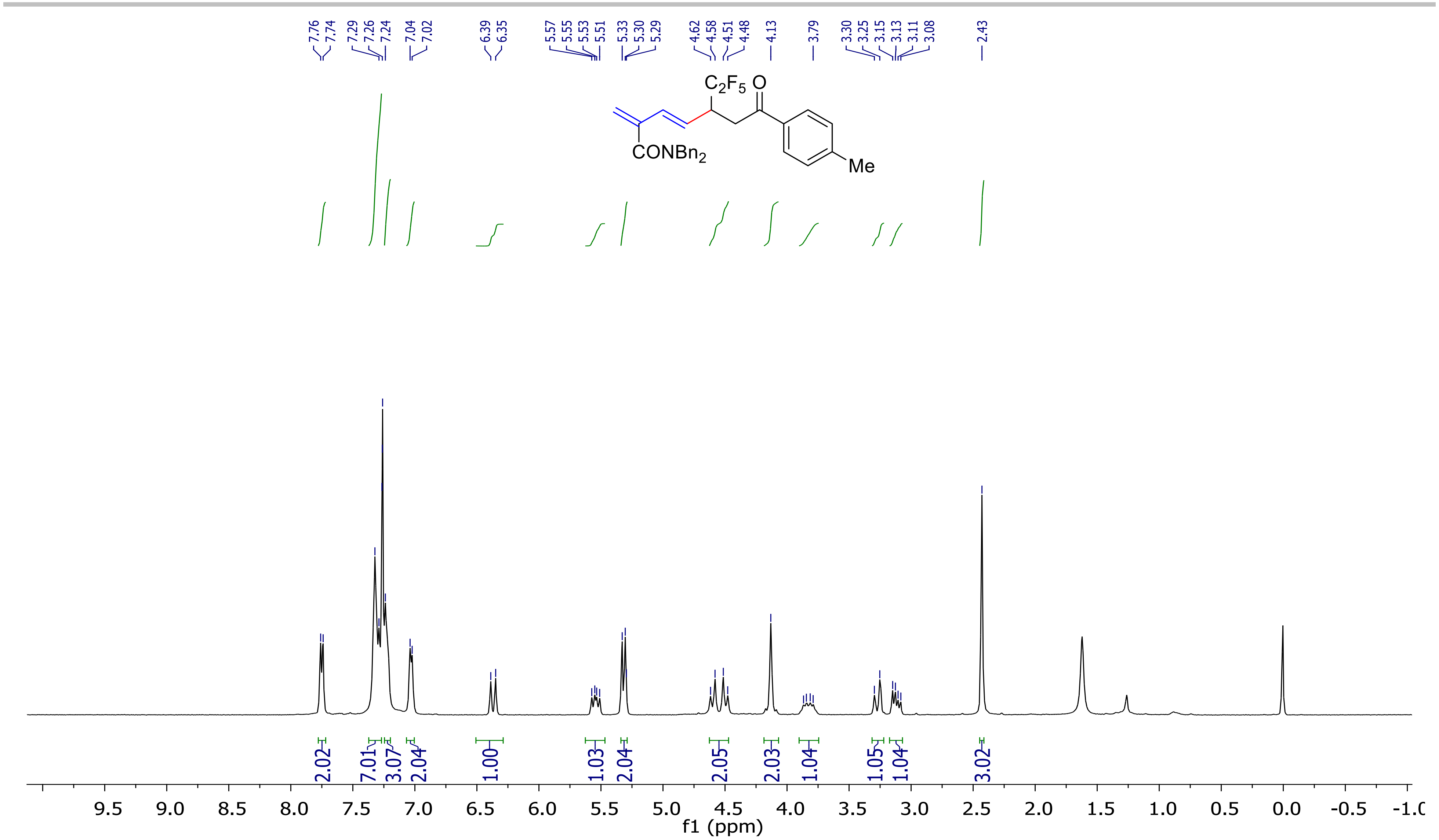

Supplementary Fig. 207 ^{1}H NMR (400 MHz, $CDCl_3$) of **5q**

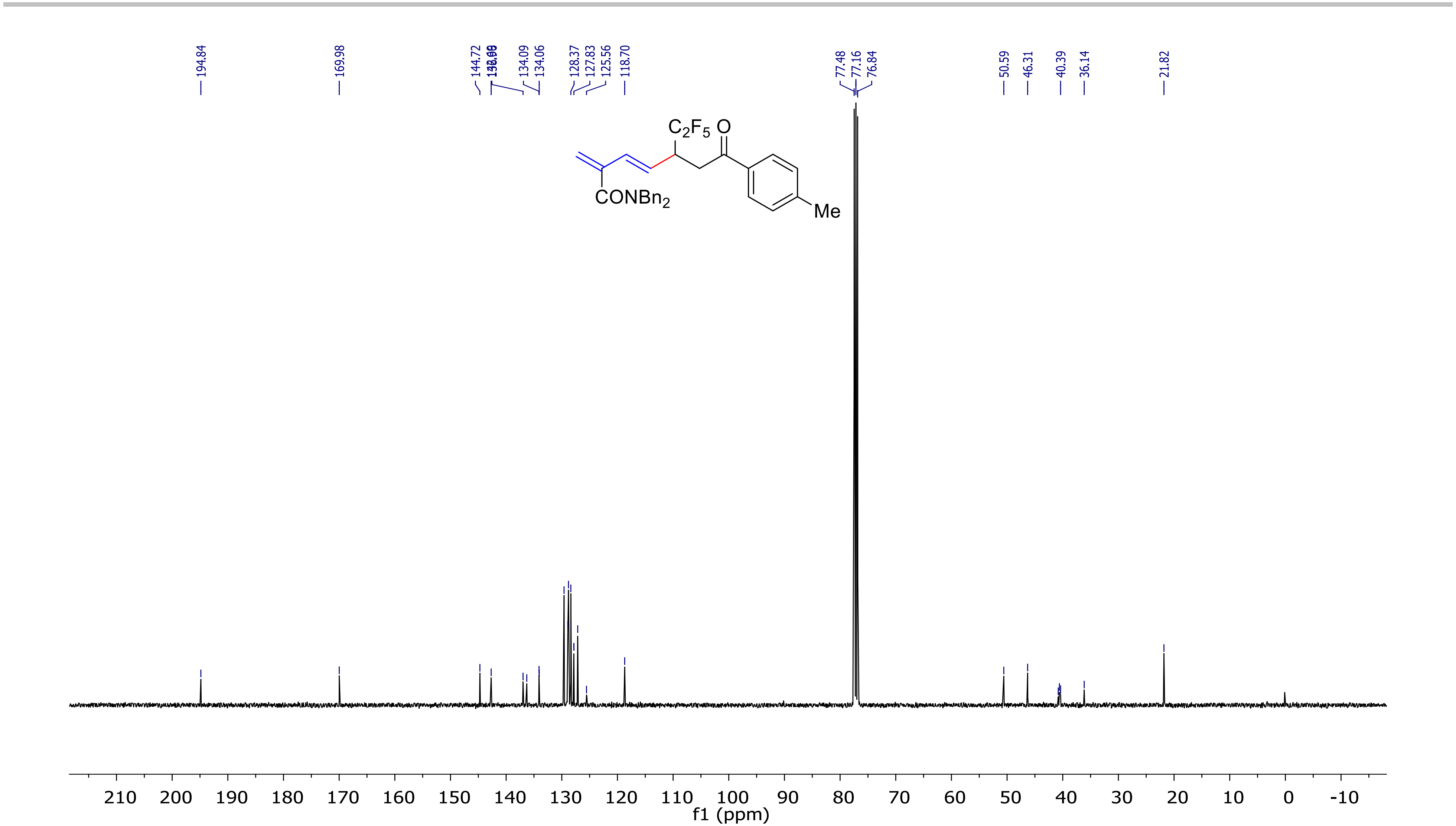

Supplementary Fig. 208 ^{13}C NMR (101 MHz, $CDCl_3$) of **5q**

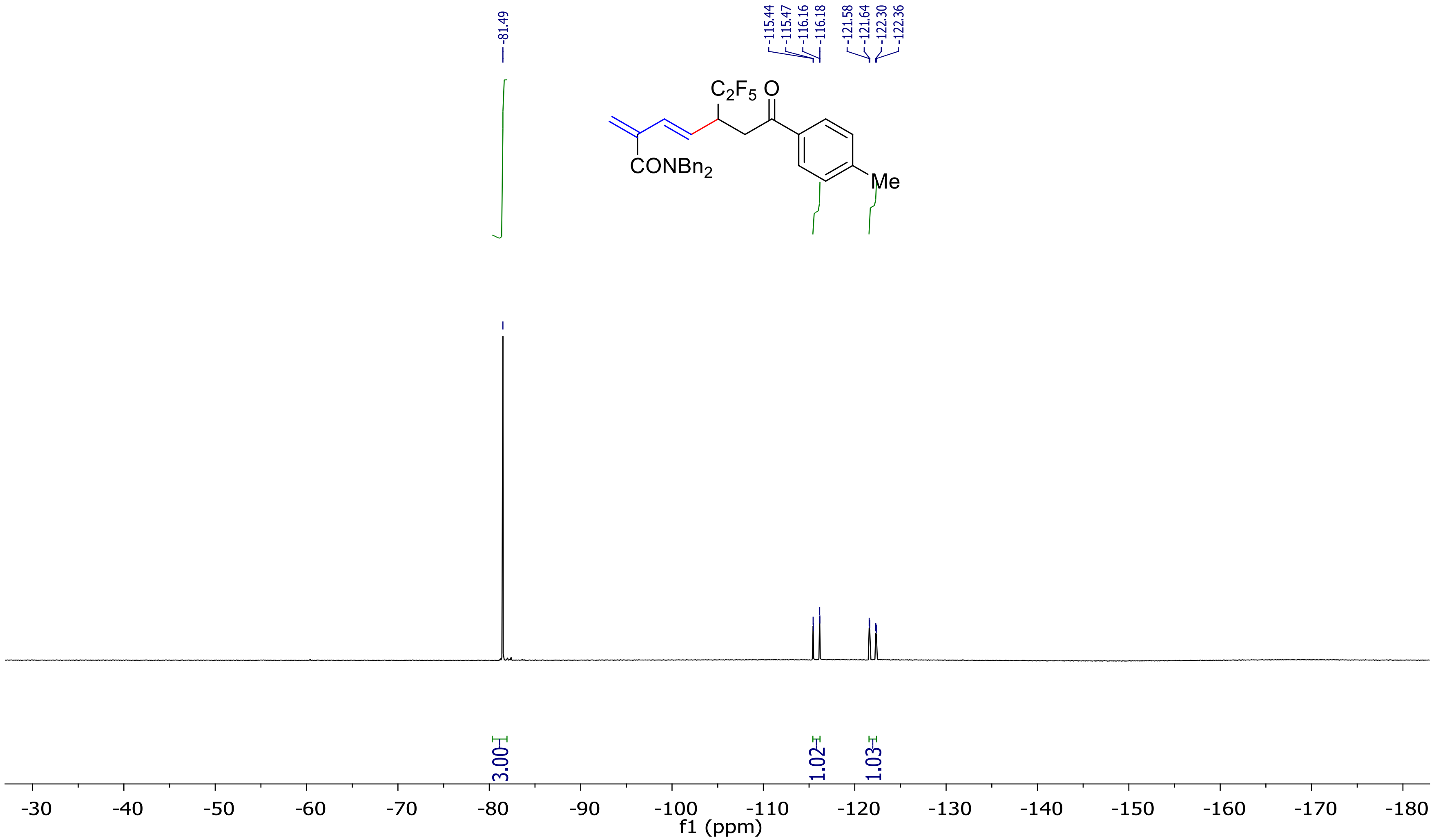

Supplementary Fig. 209 ^{19}F NMR (377 MHz, $CDCl_3$) of **5q**

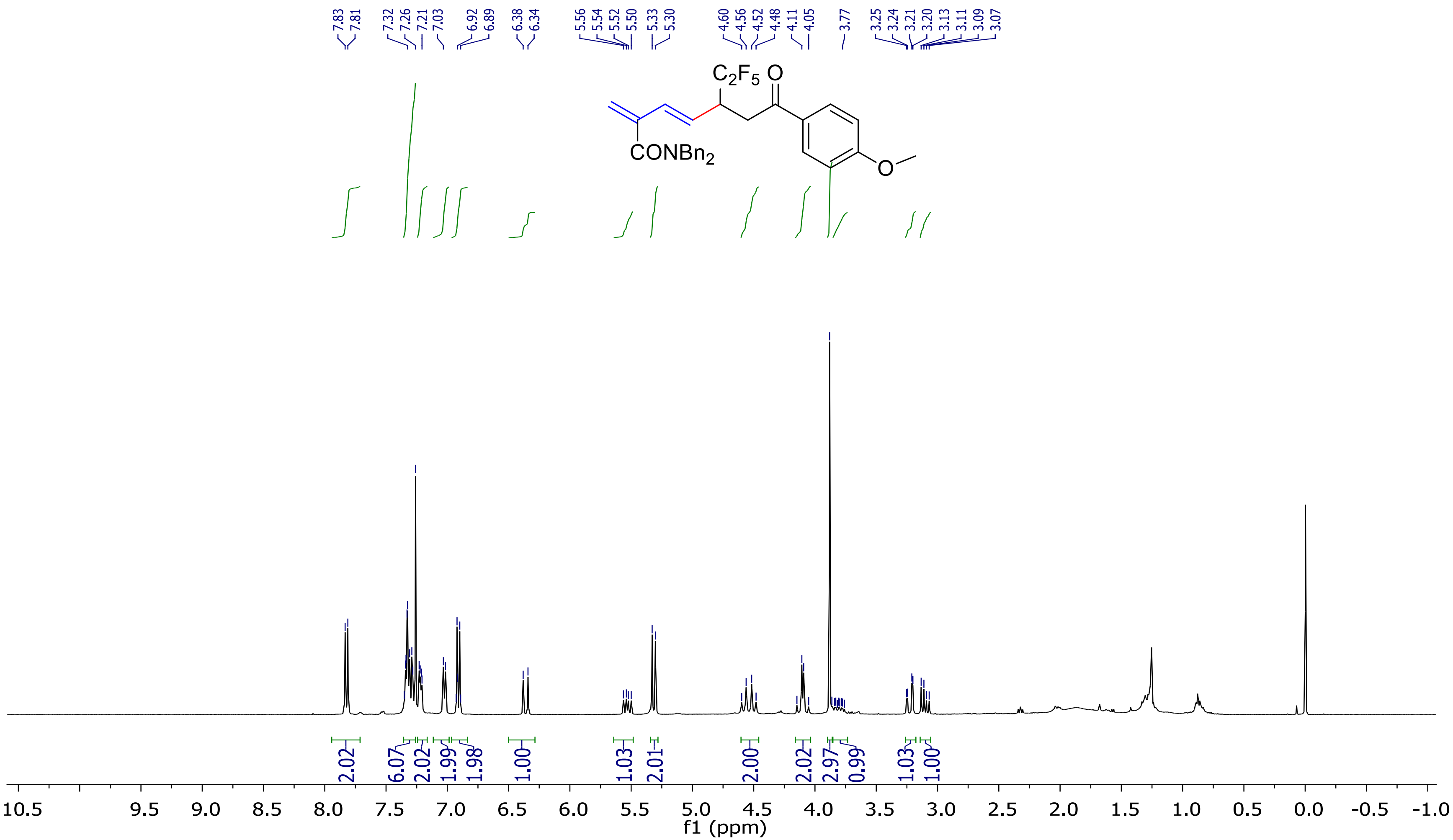

Supplementary Fig. 210 ^{1}H NMR (400 MHz, $CDCl_3$) of **5r**

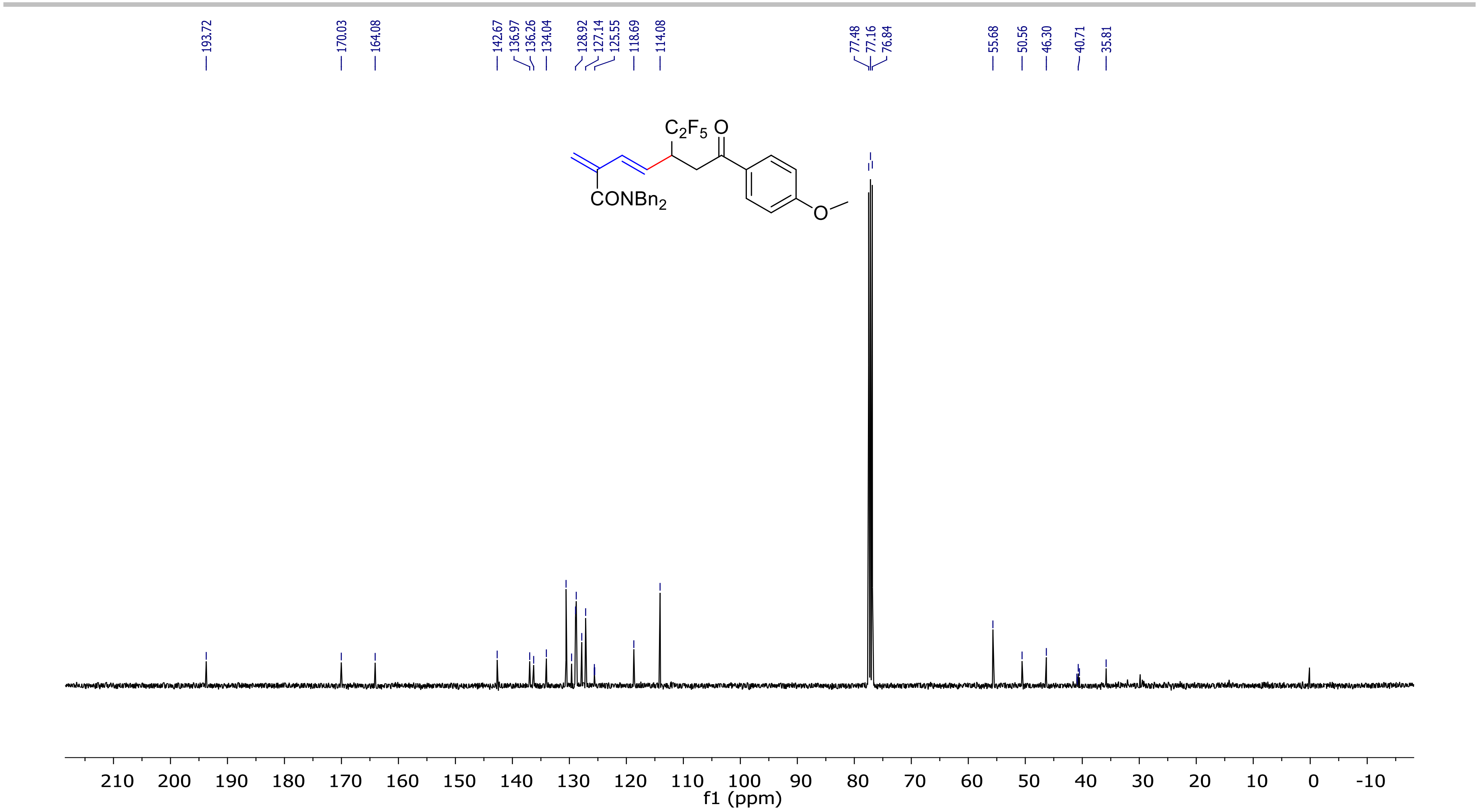

Supplementary Fig. 211 ^{13}C NMR (101 MHz, $CDCl_3$) of **5r**

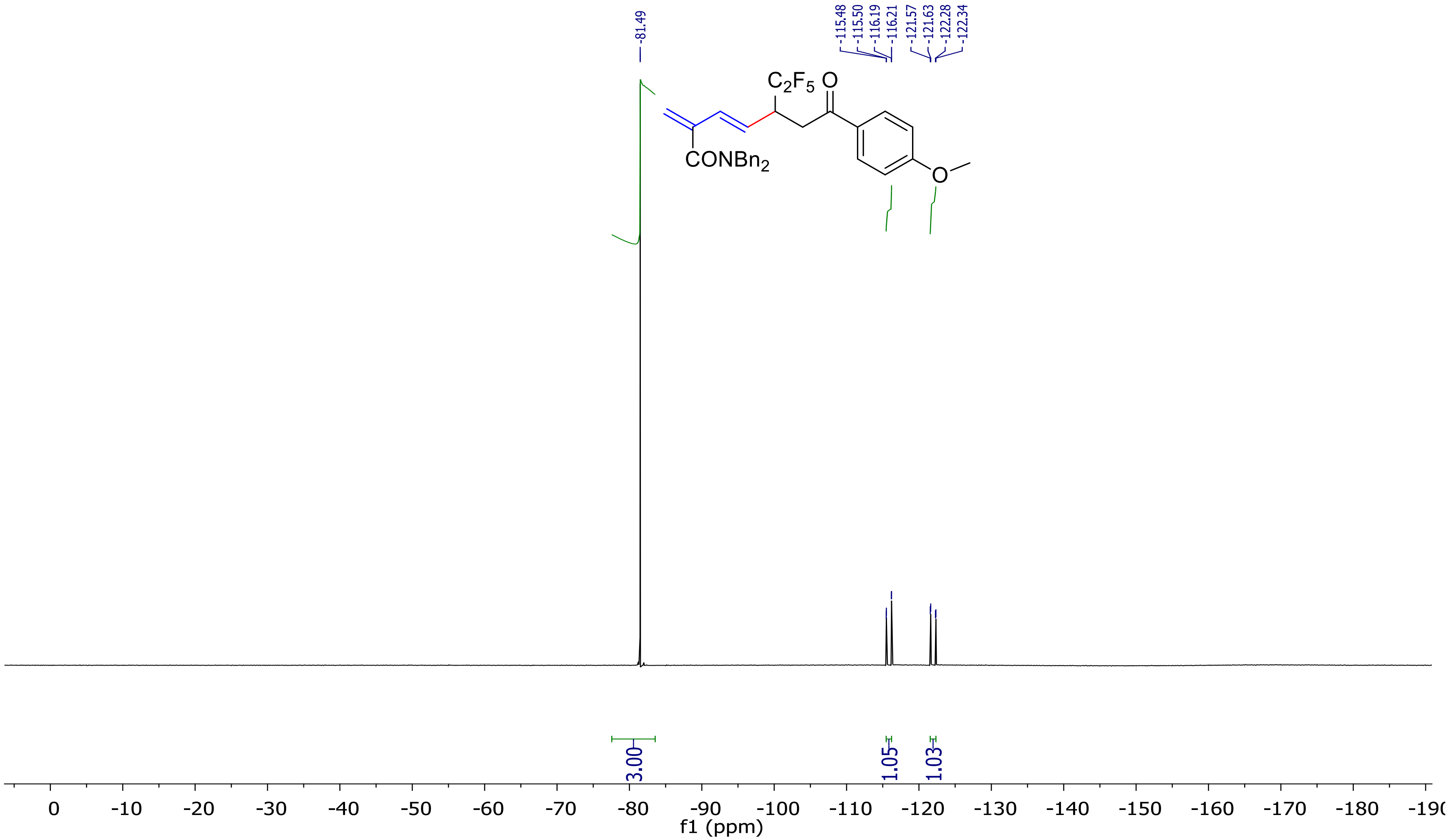

Supplementary Fig. 212 ^{19}F NMR (377 MHz, $CDCl_3$) of **5r**

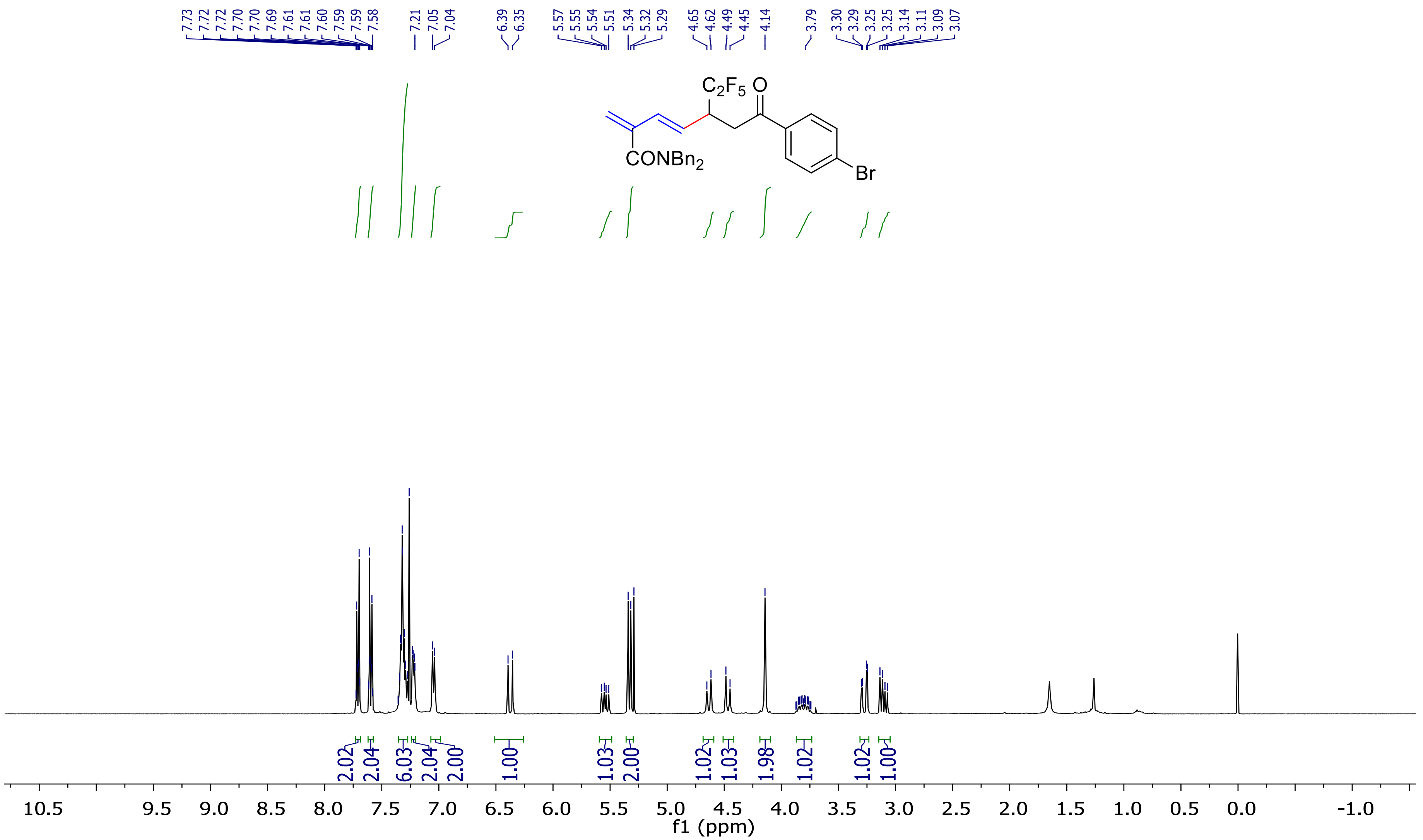

Supplementary Fig. 213 ^{1}H NMR (400 MHz, $CDCl_3$) of **5s**

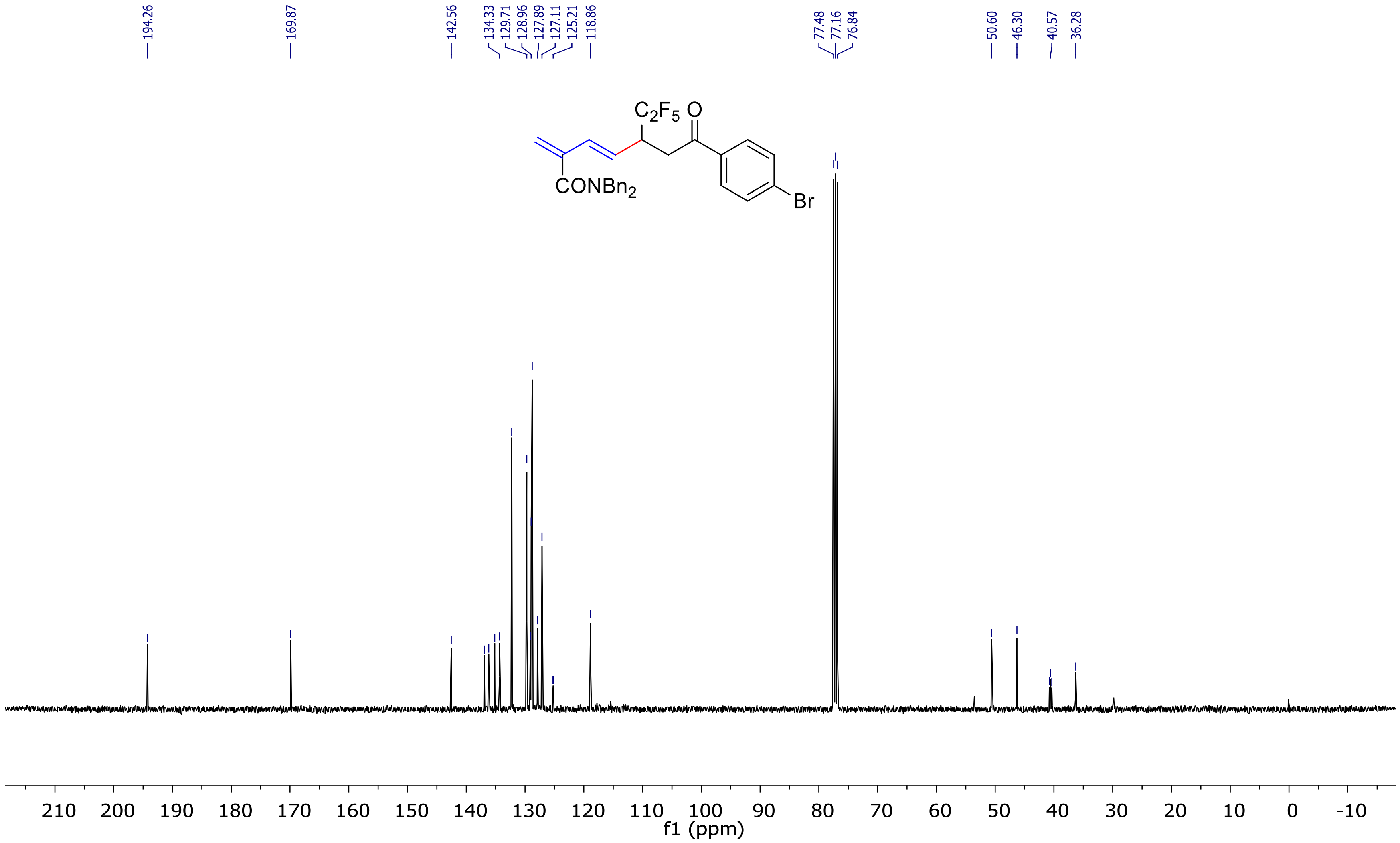

Supplementary Fig. 214 ^{13}C NMR (101 MHz, $CDCl_3$) of **5s**

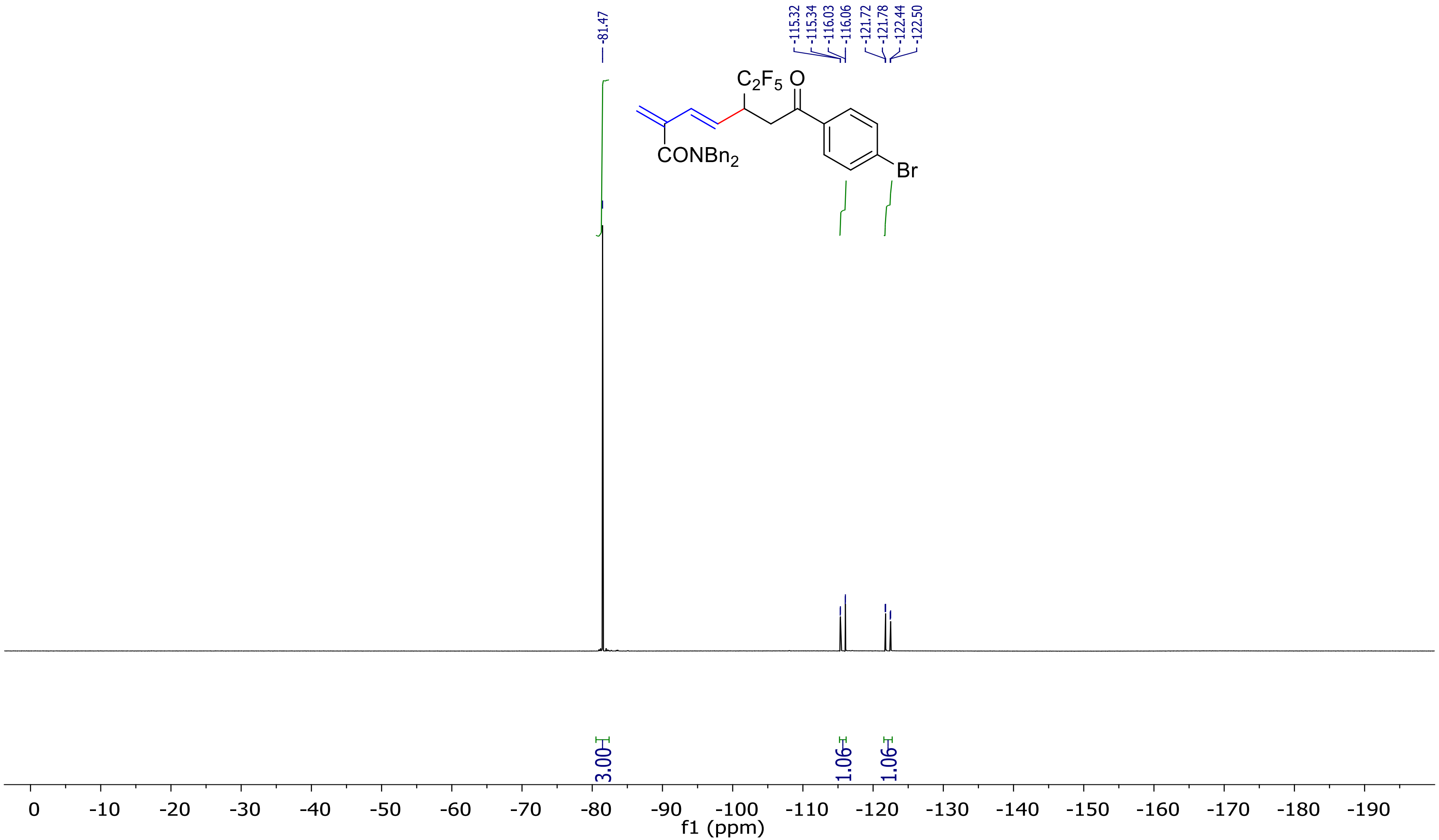

Supplementary Fig. 215 ^{19}F NMR (377 MHz, $CDCl_3$) of **5s**

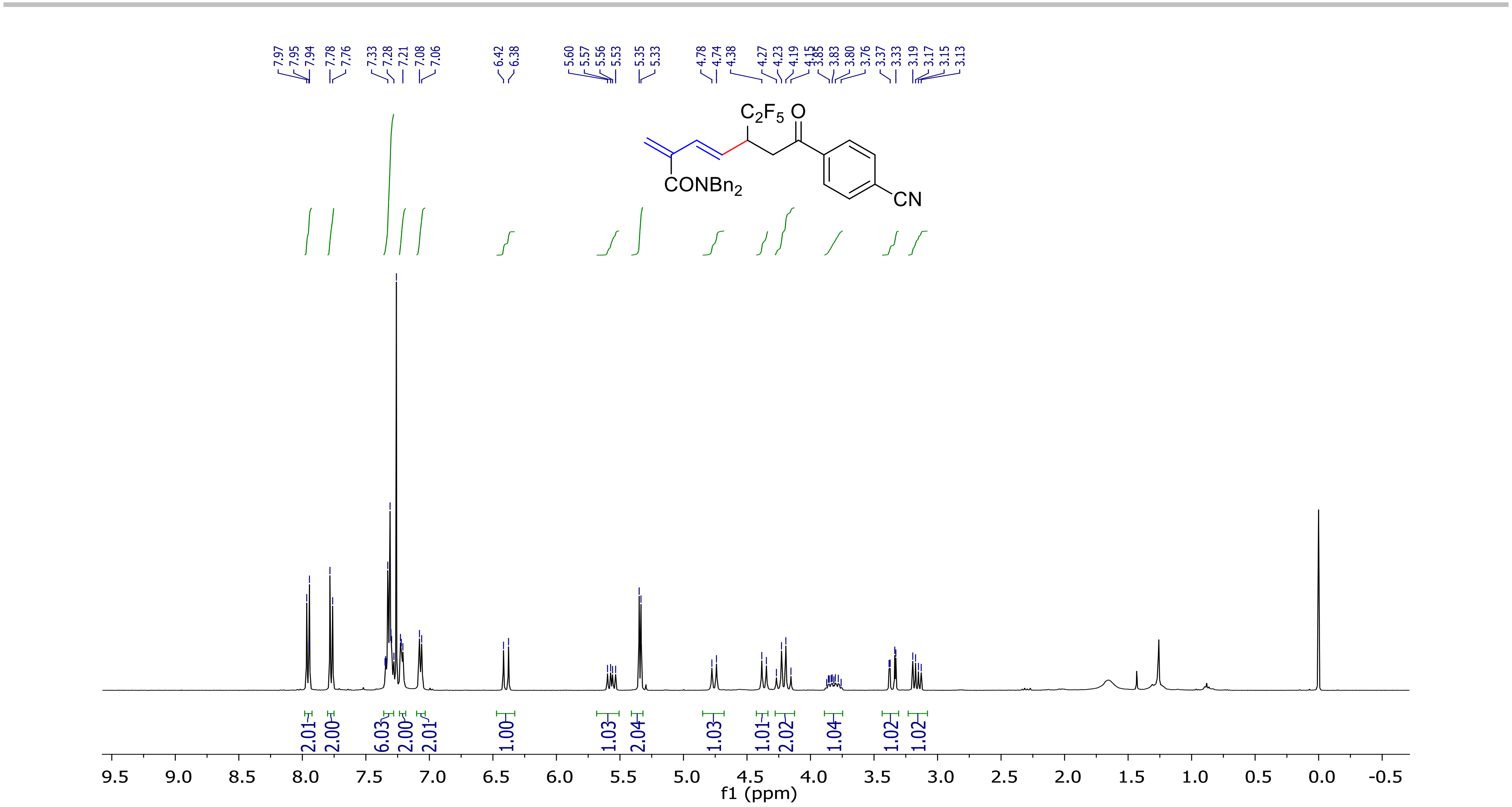

Supplementary Fig. 216 ^{1}H NMR (400 MHz, $CDCl_3$) of **5t**

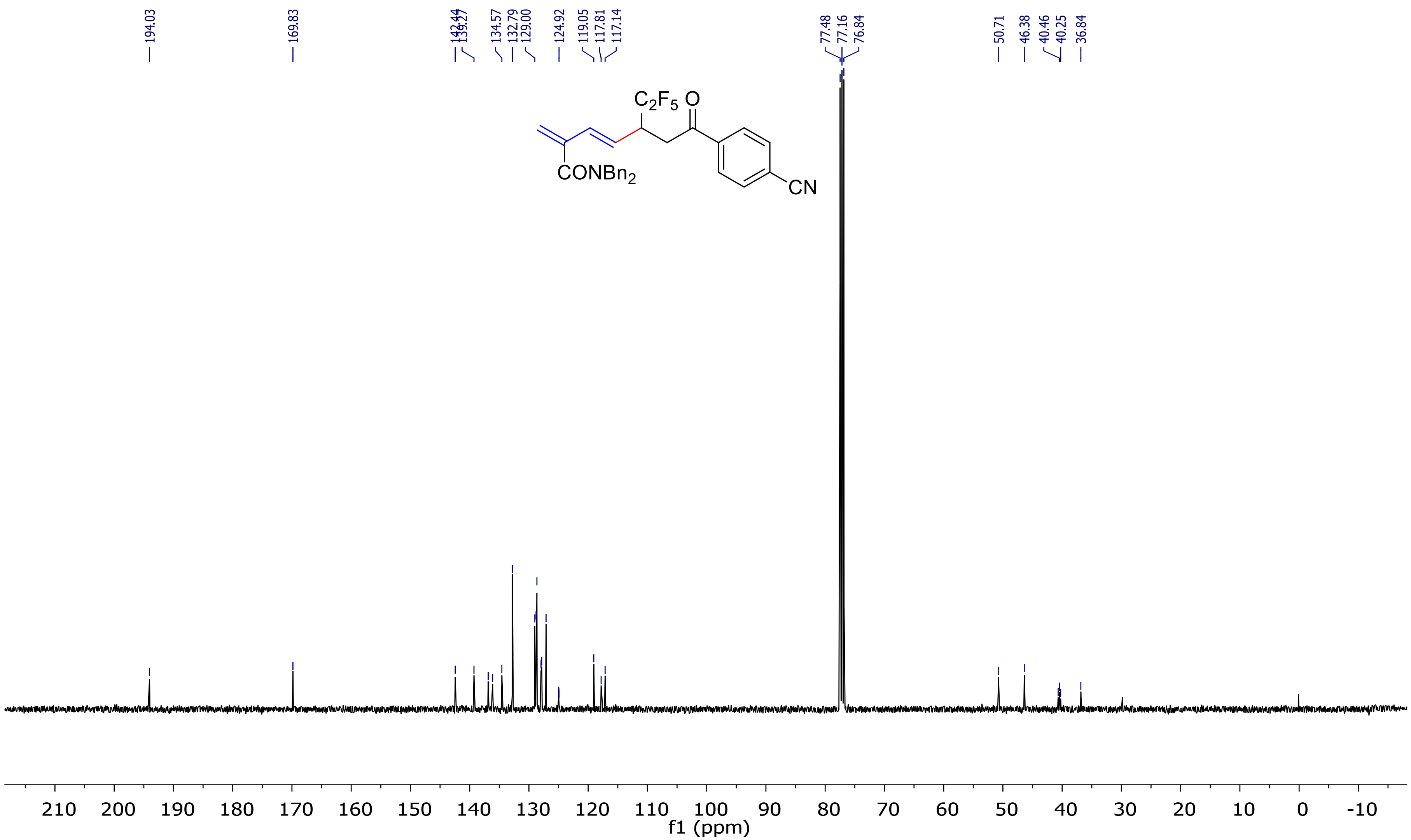

Supplementary Fig. 217 ^{13}C NMR (101 MHz, $CDCl_3$) of **5t**

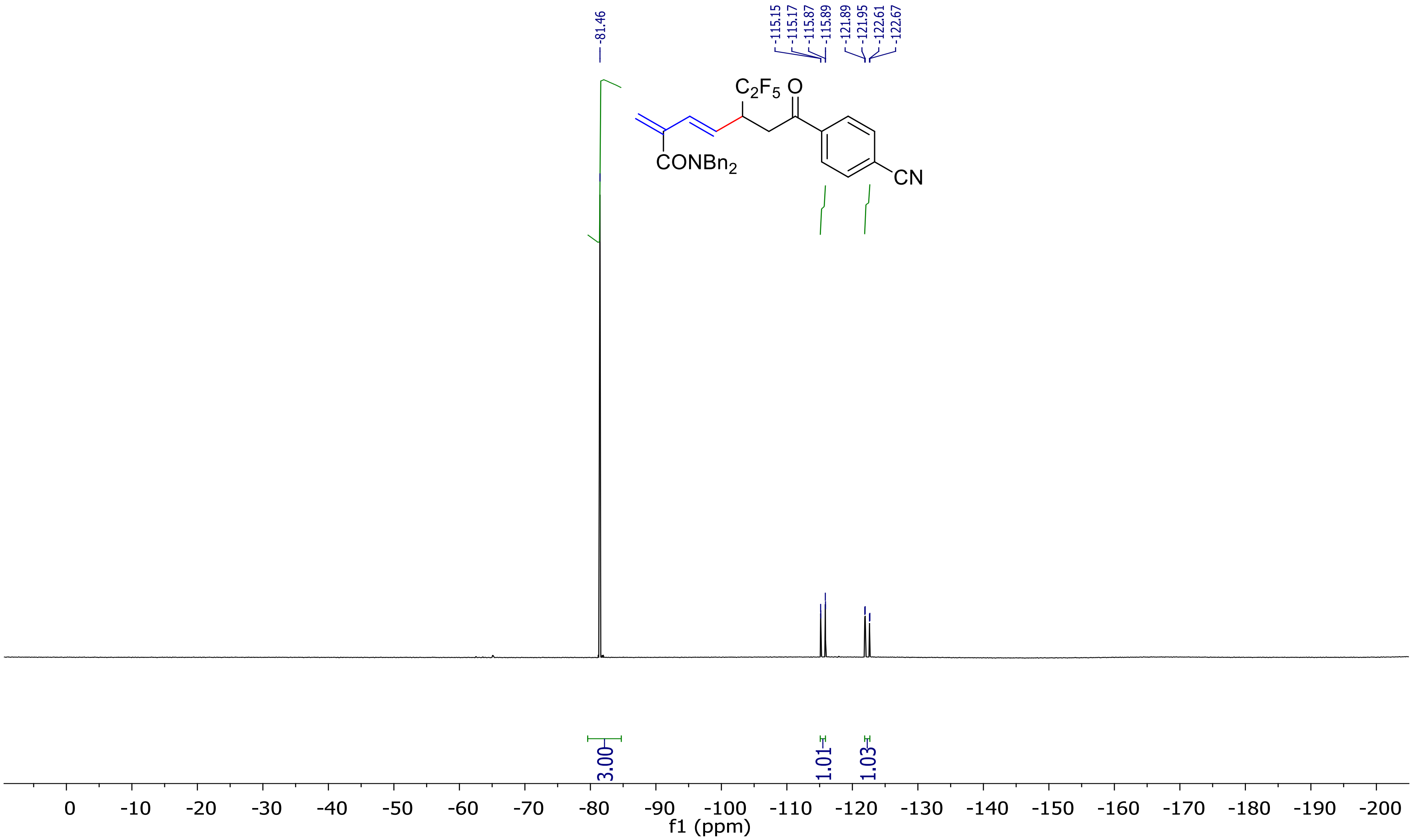

Supplementary Fig. 218 ^{19}F NMR (377 MHz, $CDCl_3$) of **5t**

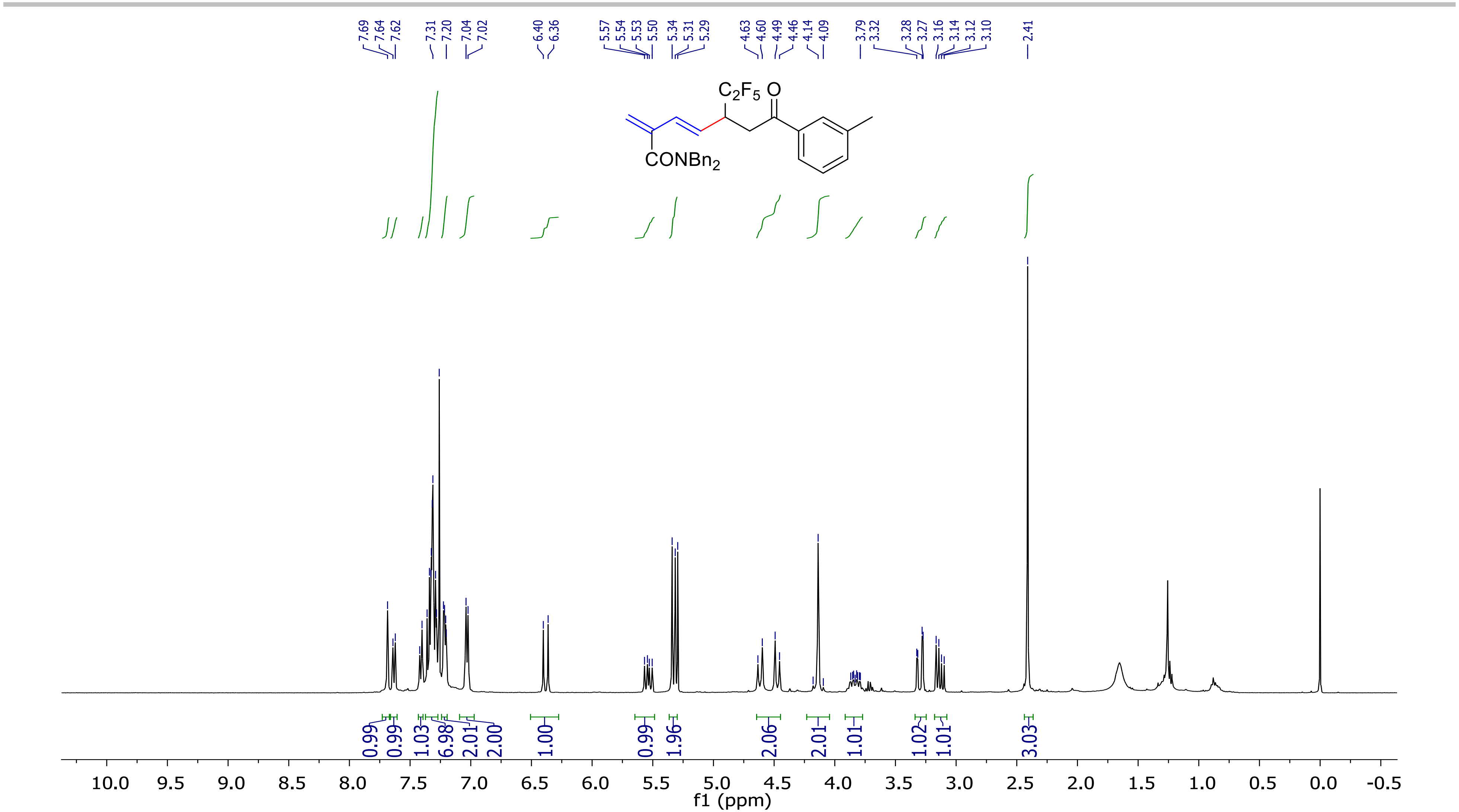

Supplementary Fig. 219 ^{1}H NMR (400 MHz, $CDCl_3$) of **5u**

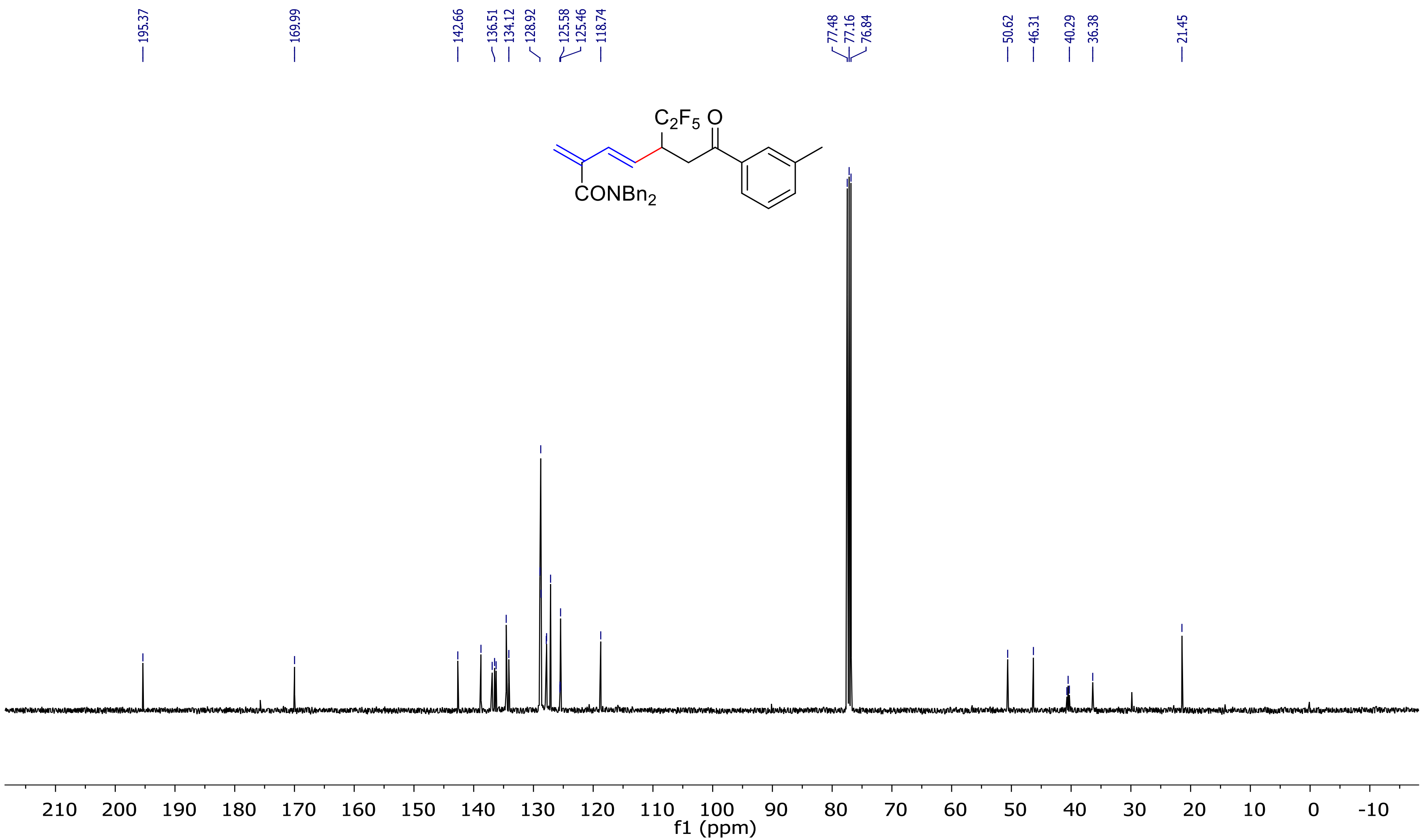

Supplementary Fig. 220 ^{13}C NMR (101 MHz, $CDCl_3$) of **5u**

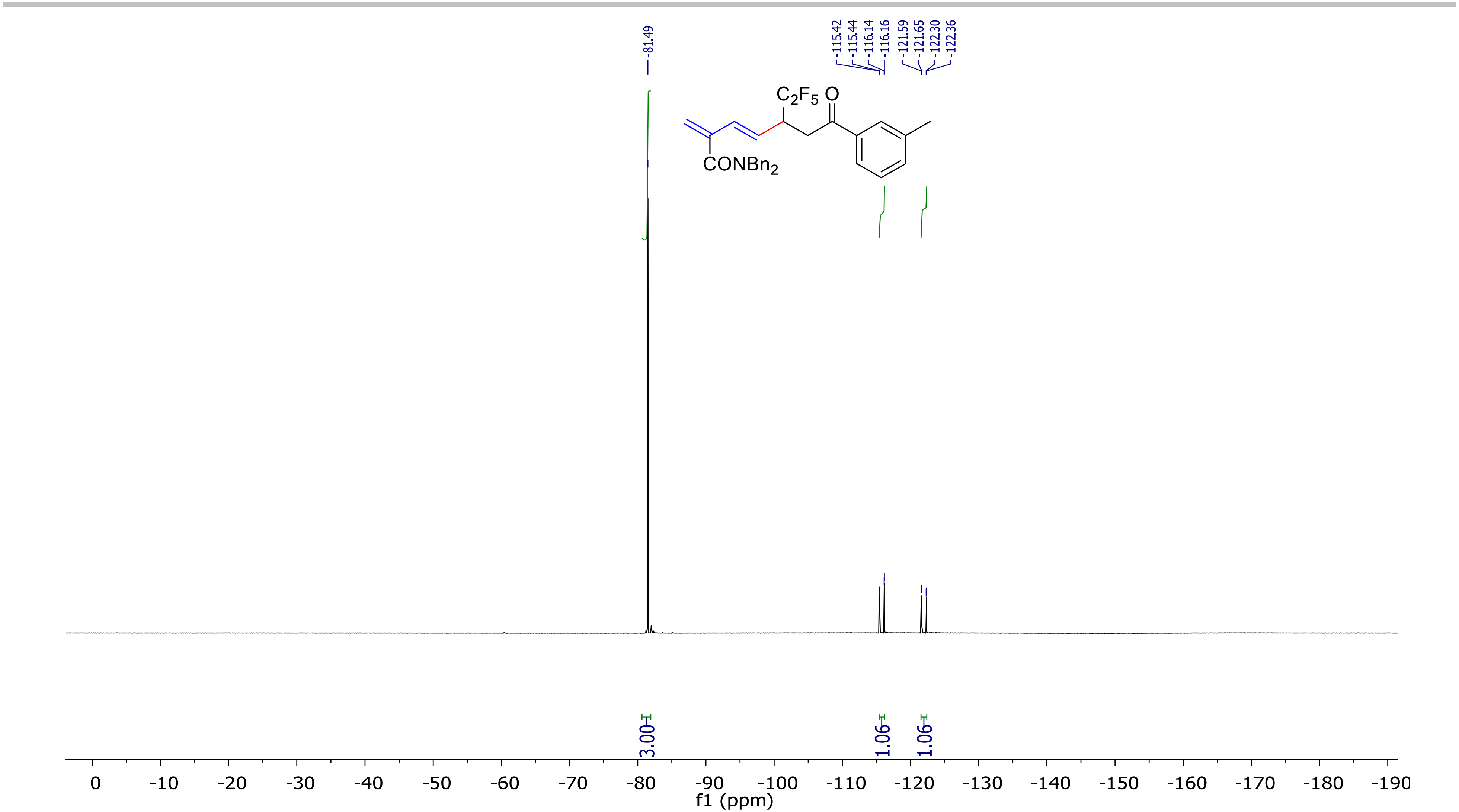

Supplementary Fig. 221 ^{19}F NMR (377 MHz, $CDCl_3$) of **5u**

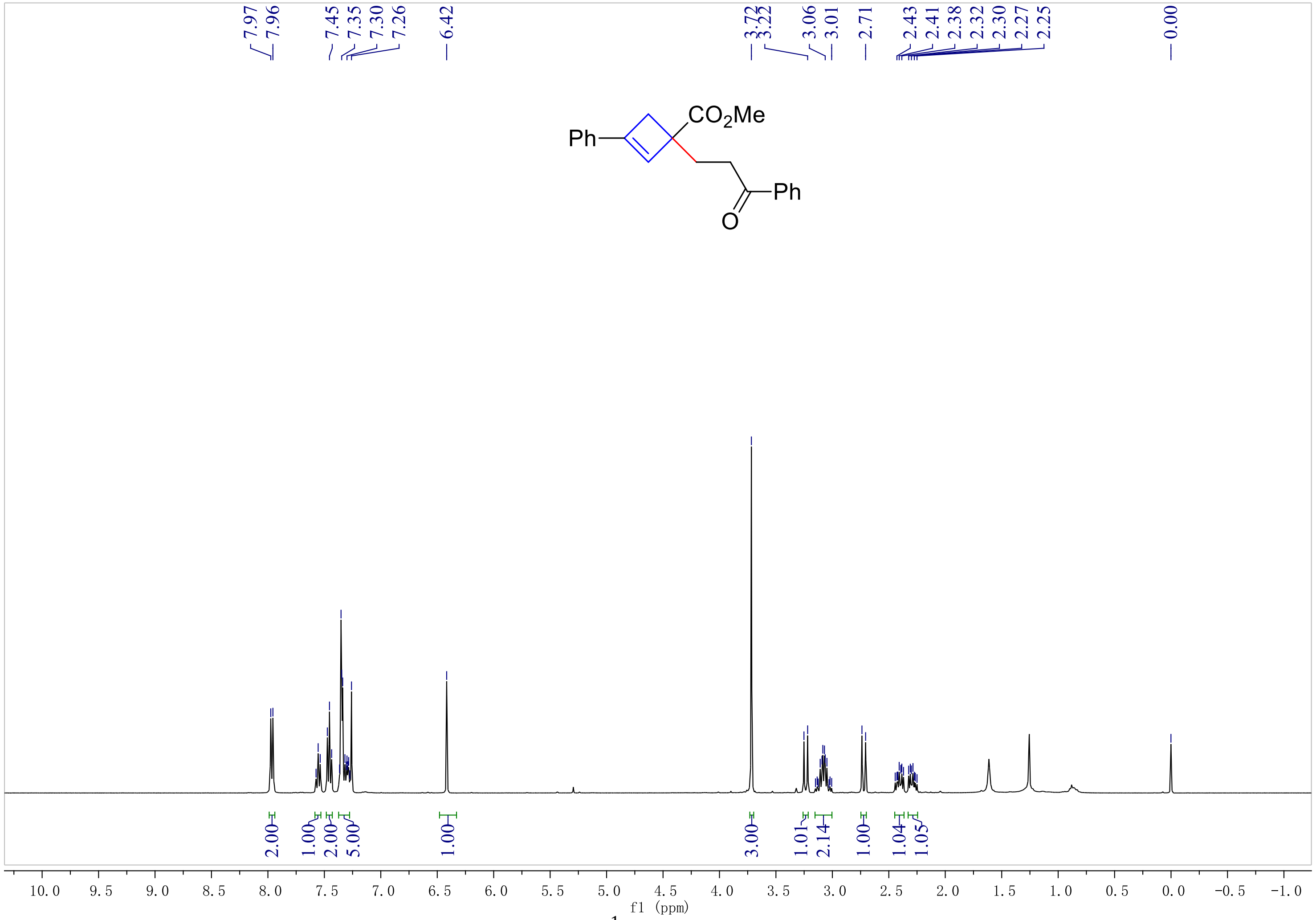

Supplementary Fig. 222 ^{1}H NMR (400 MHz, $CDCl_3$) of **6a**

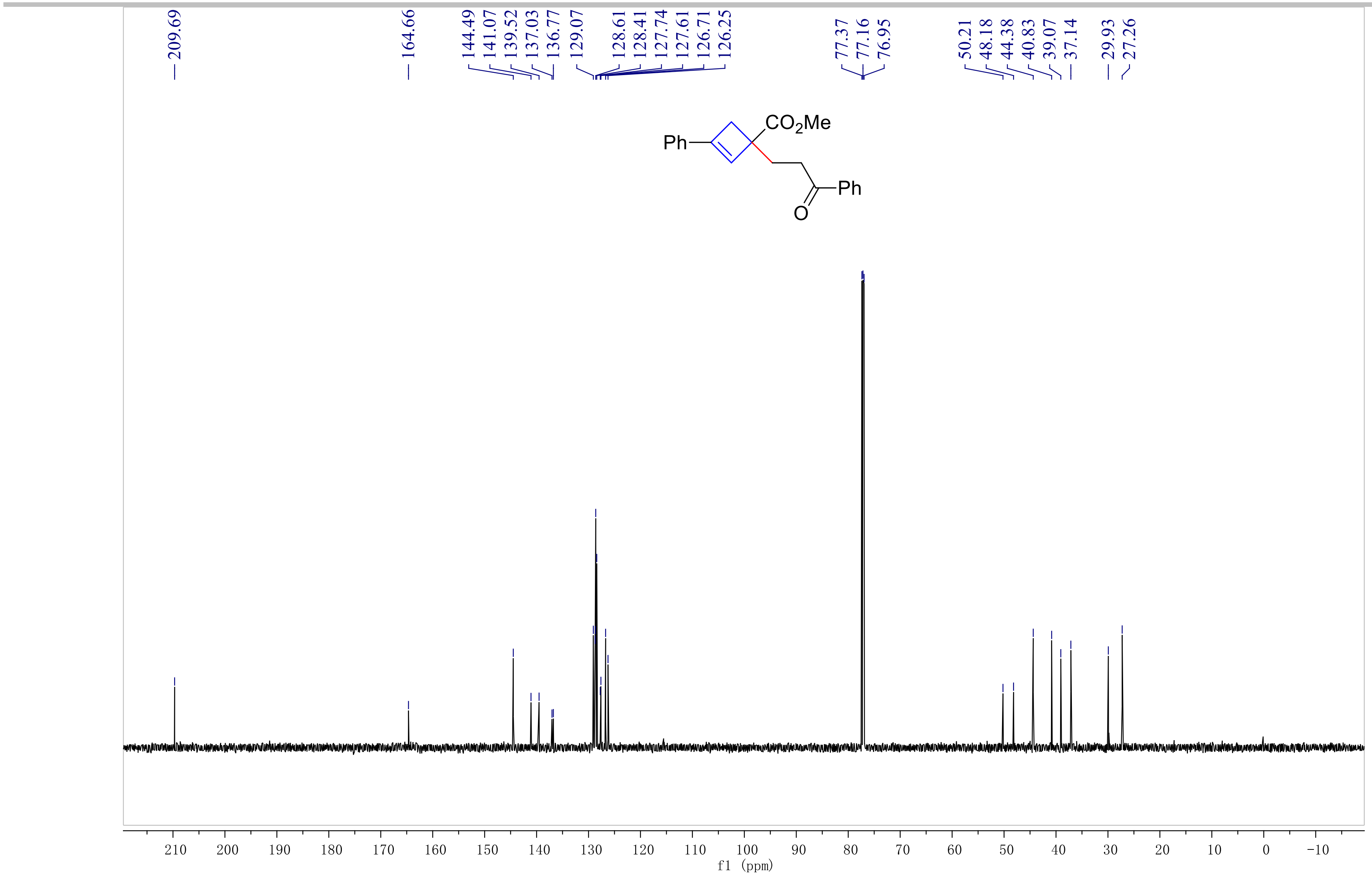

Supplementary Fig. 223 ^{13}C NMR (151 MHz, $CDCl_3$) of **6a**

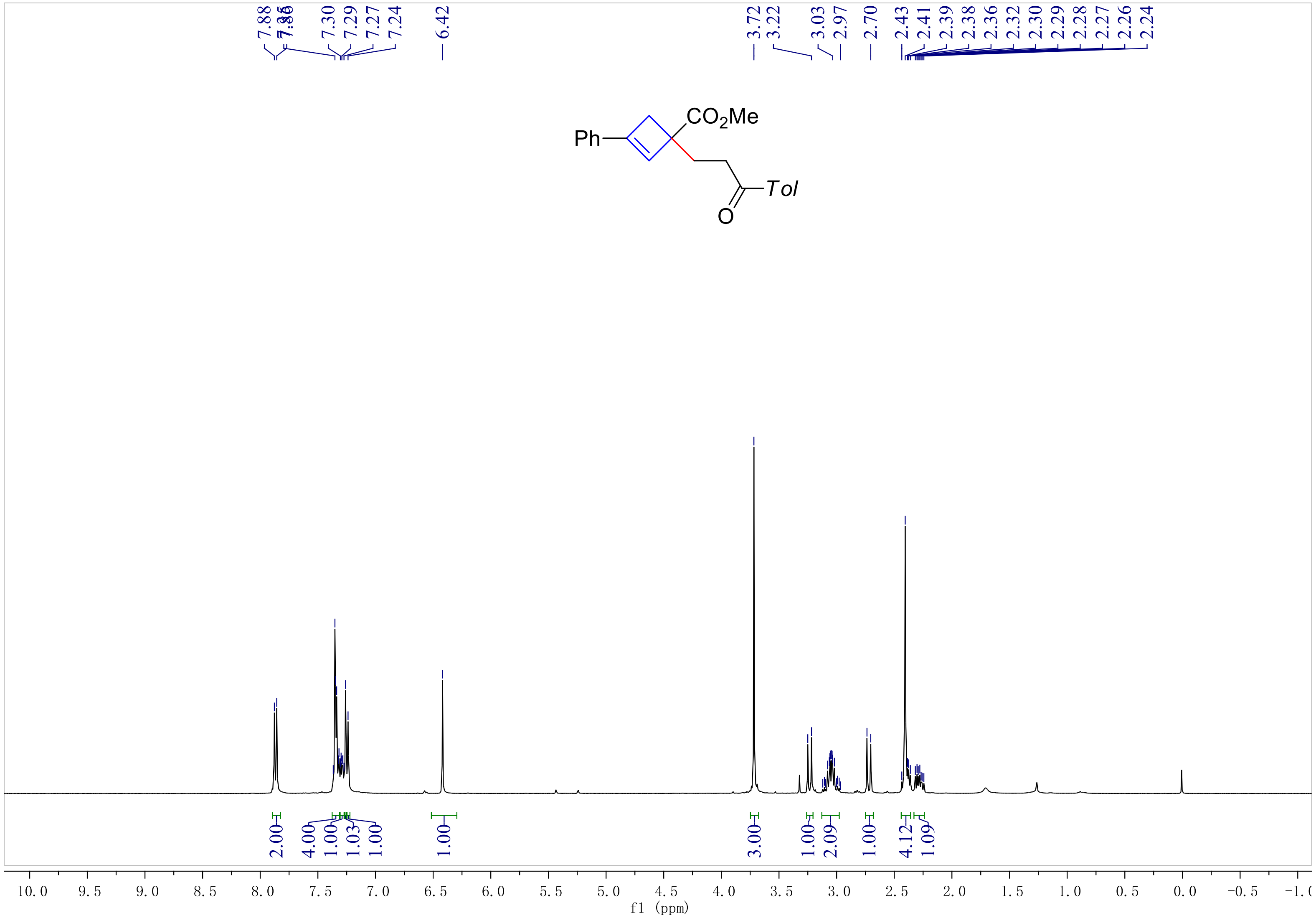

Supplementary Fig. 224 ^{1}H NMR (400 MHz, $CDCl_3$) of **6b**

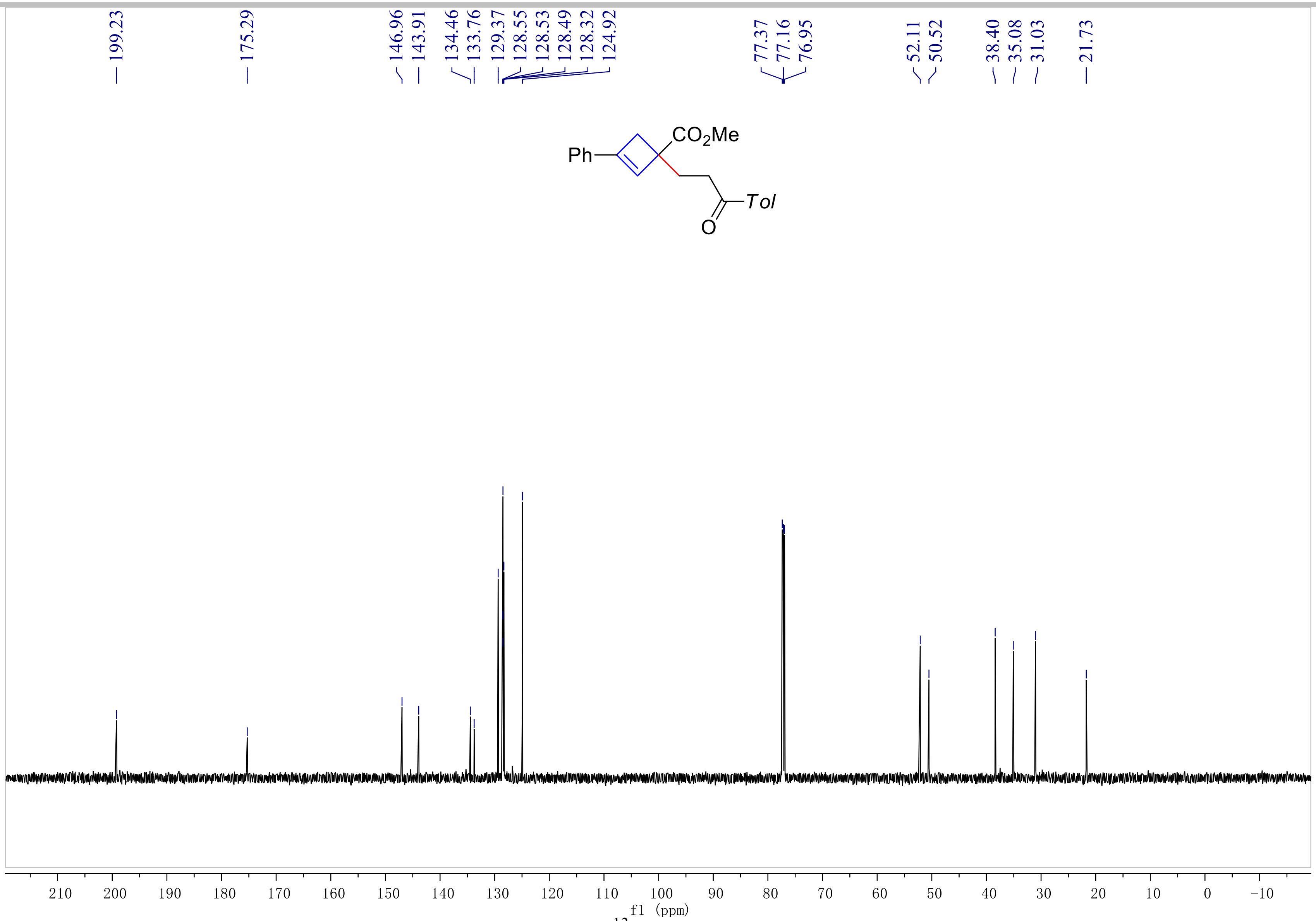

Supplementary Fig. 225 ^{13}C NMR (151 MHz, $CDCl_3$) of **6b**

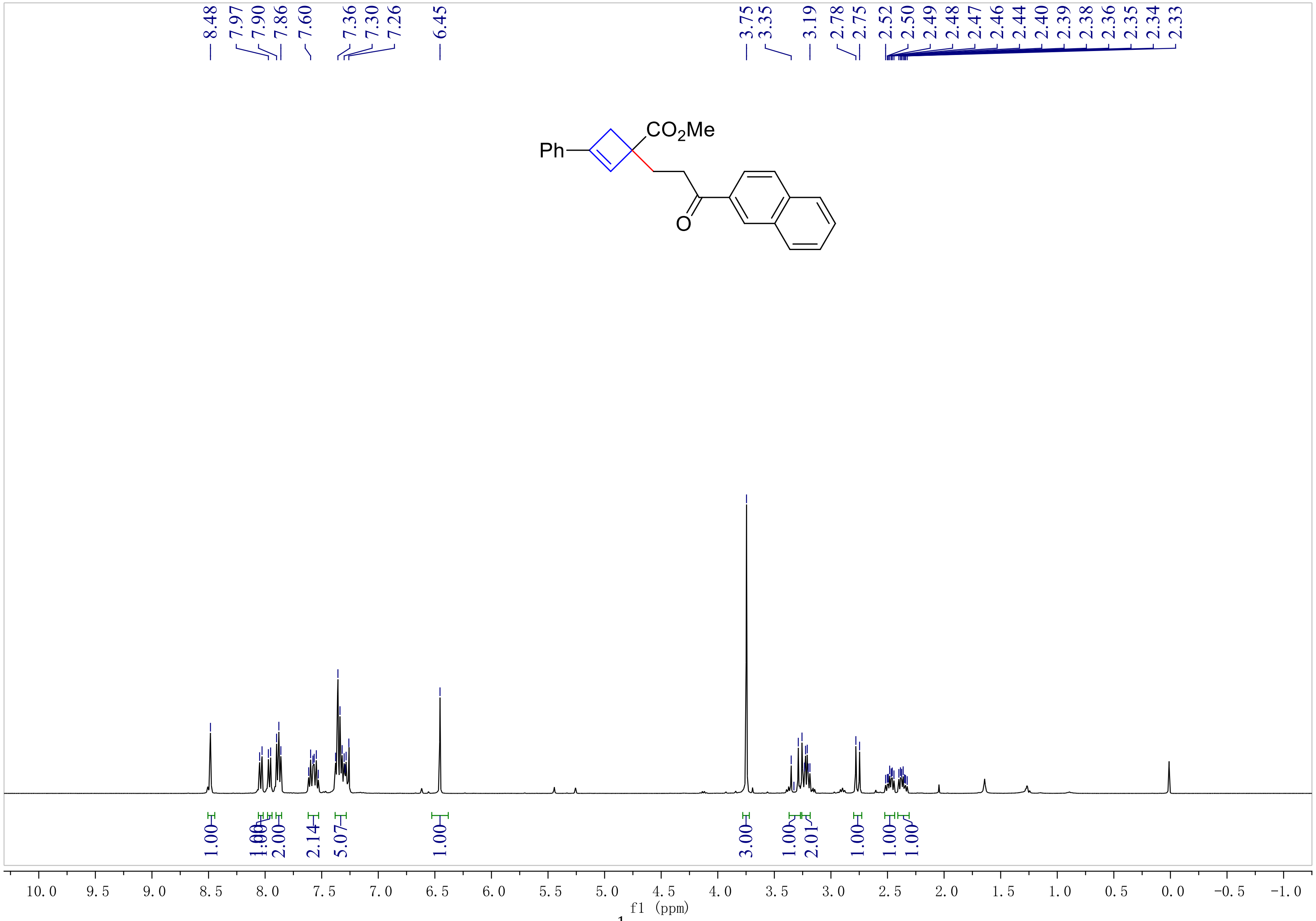

Supplementary Fig. 226 ^{1}H NMR (400 MHz, $CDCl_3$) of **6c**

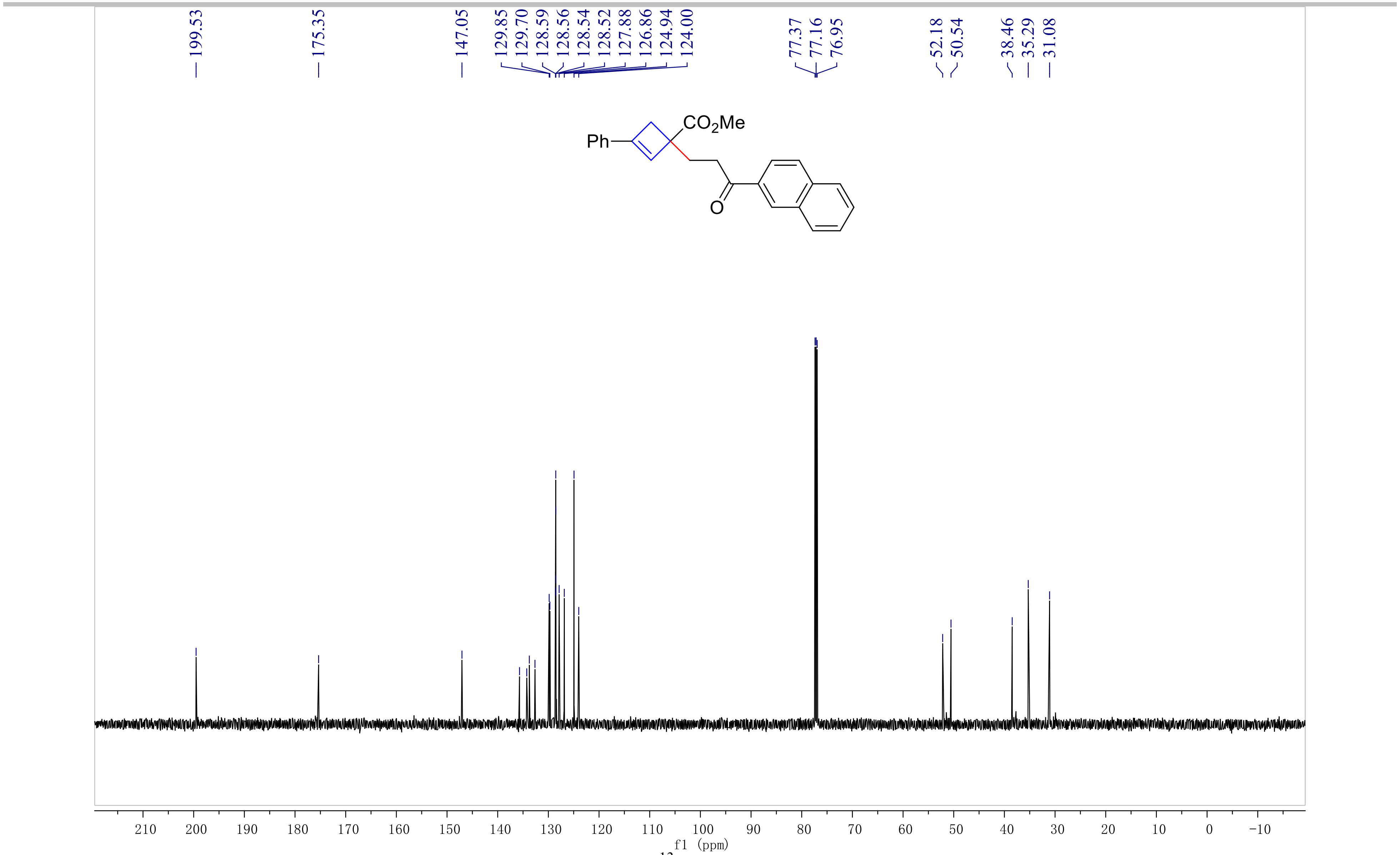

Supplementary Fig. 227 ^{13}C NMR (151 MHz, $CDCl_3$) of **6c**

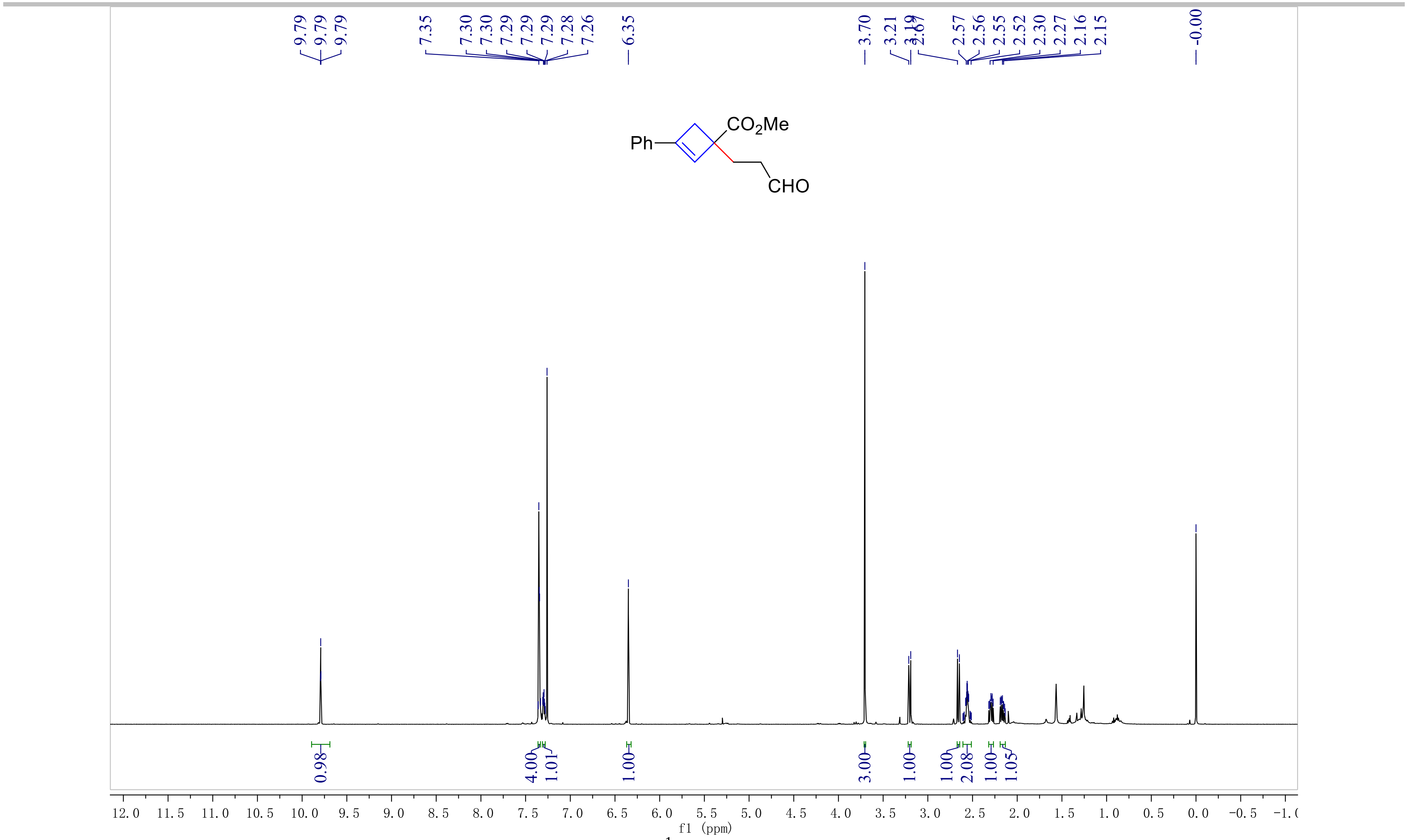

Supplementary Fig. 228 ^{1}H NMR (600 MHz, $CDCl_3$) of **6d**

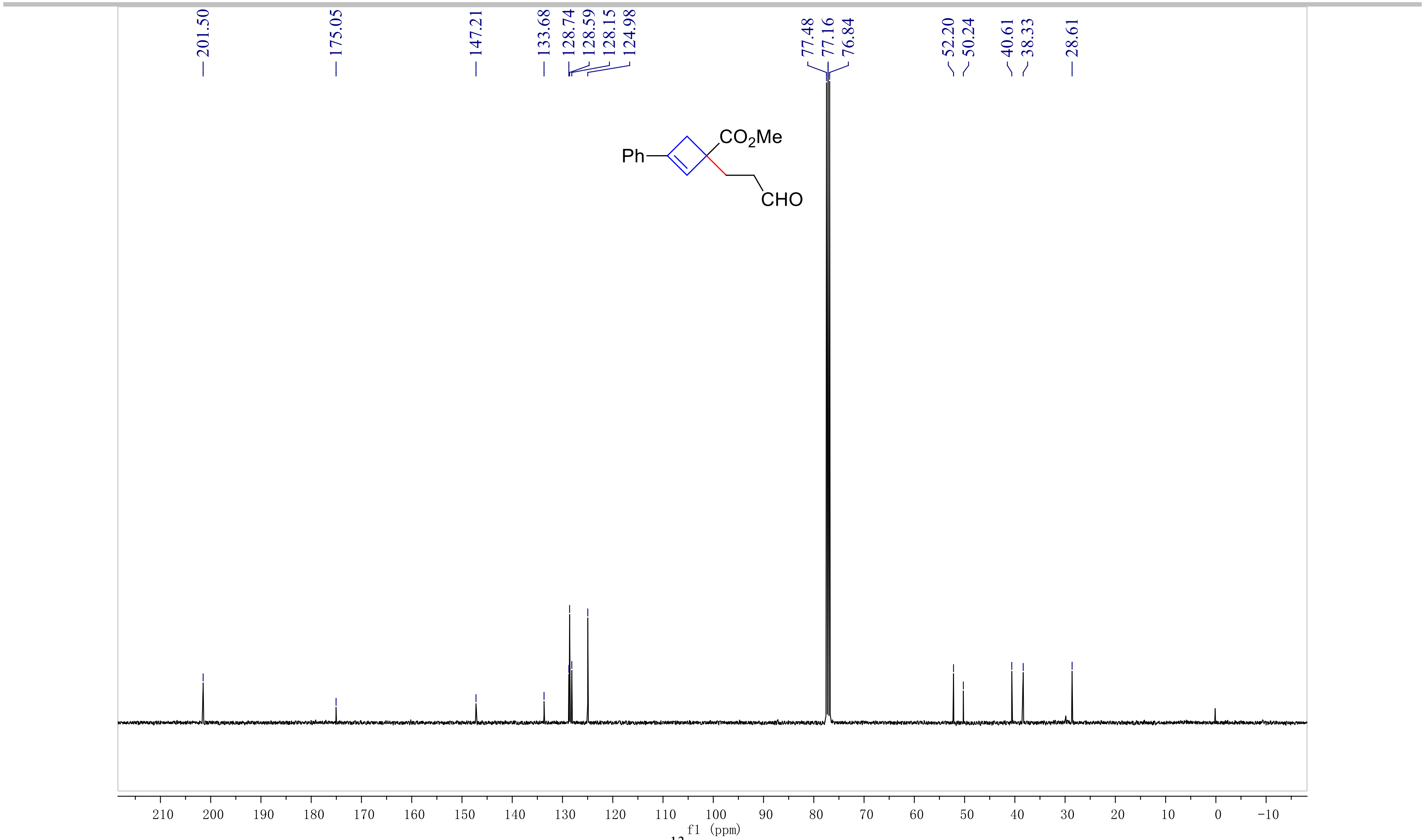

Supplementary Fig. 229 ^{13}C NMR (101 MHz, $CDCl_3$) of **6d**

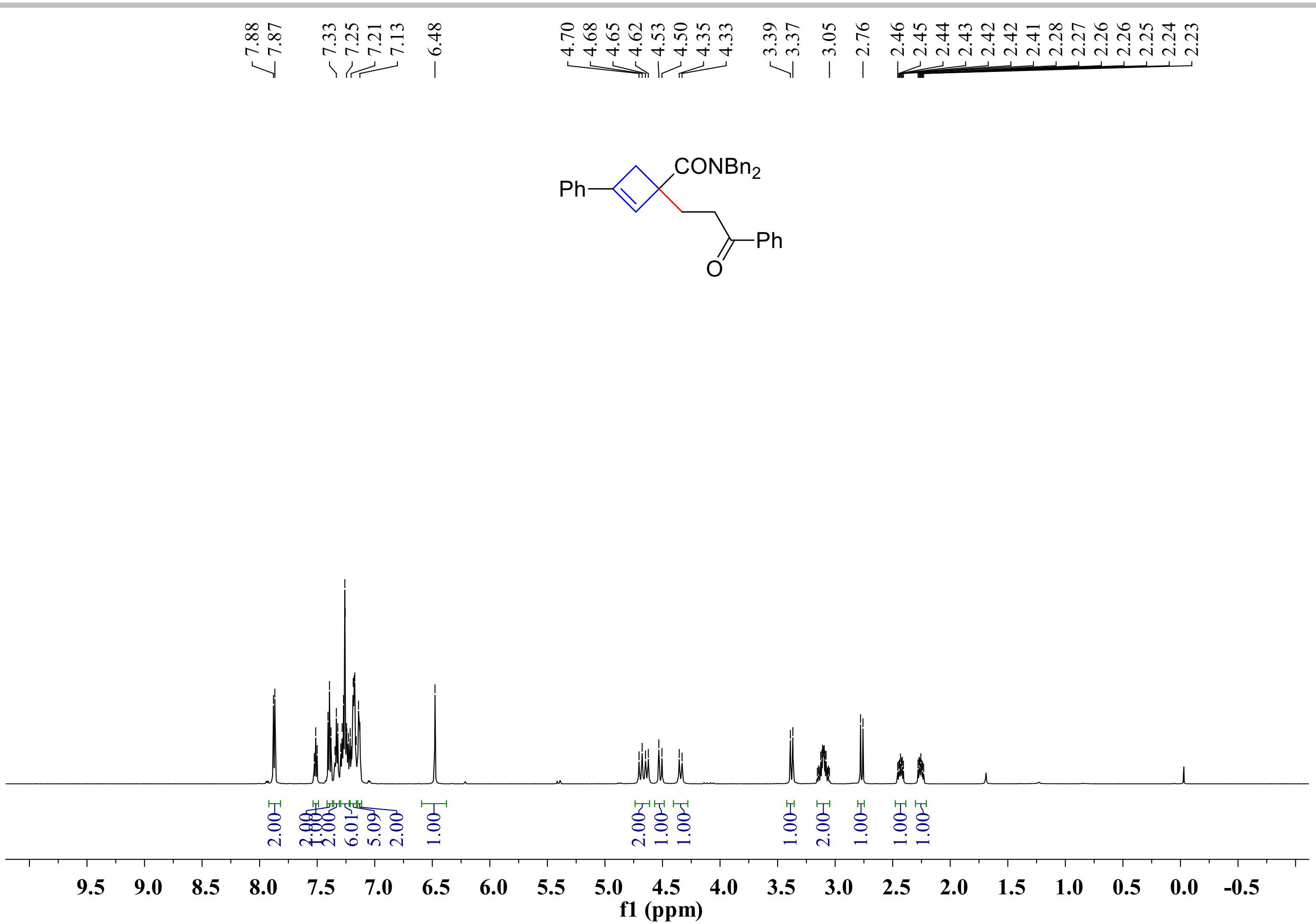

Supplementary Fig. 230 ^{1}H NMR (600 MHz, $CDCl_3$) of **6e**

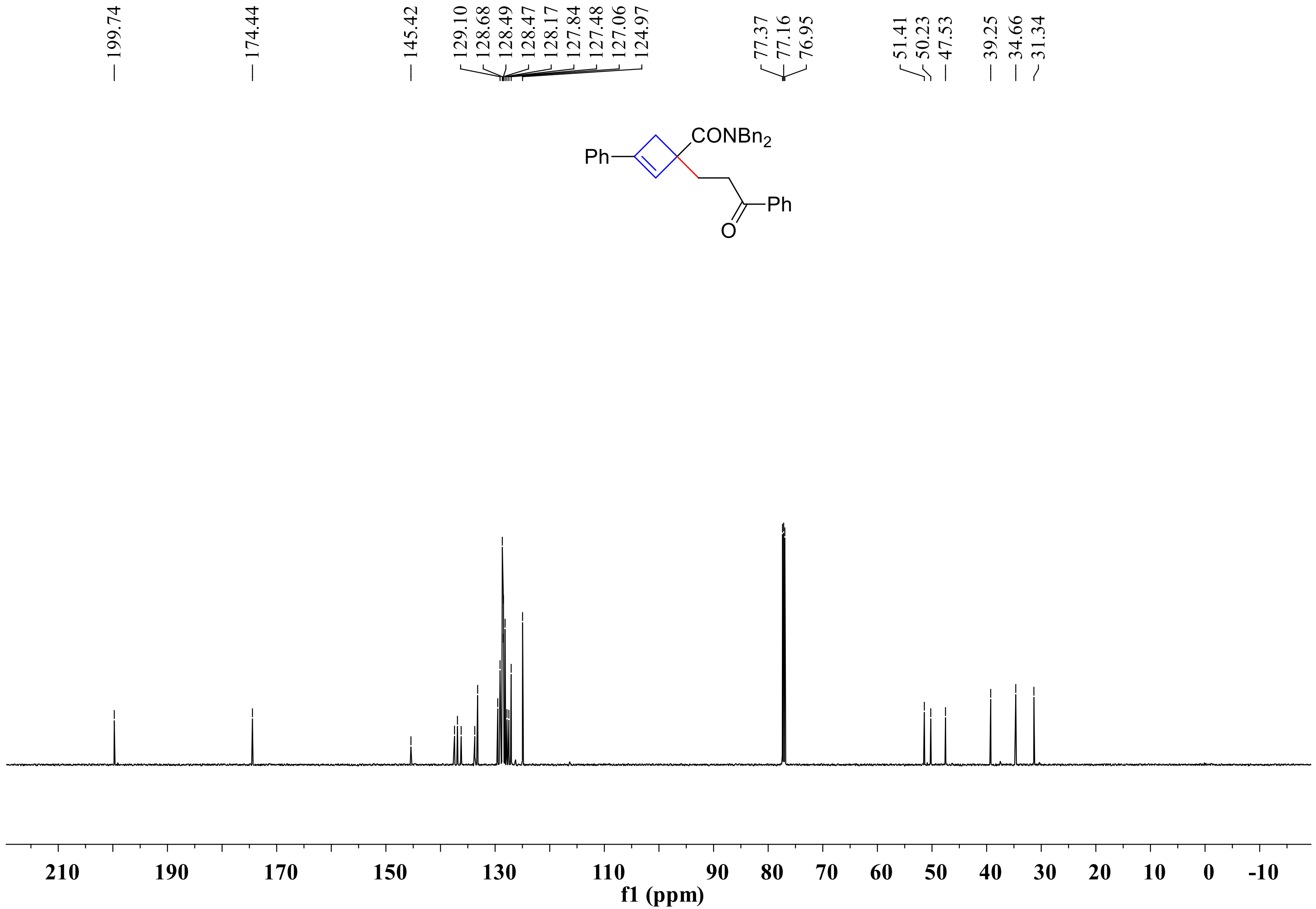

Supplementary Fig. 231 ^{13}C NMR (151 MHz, $CDCl_3$) of **6e**

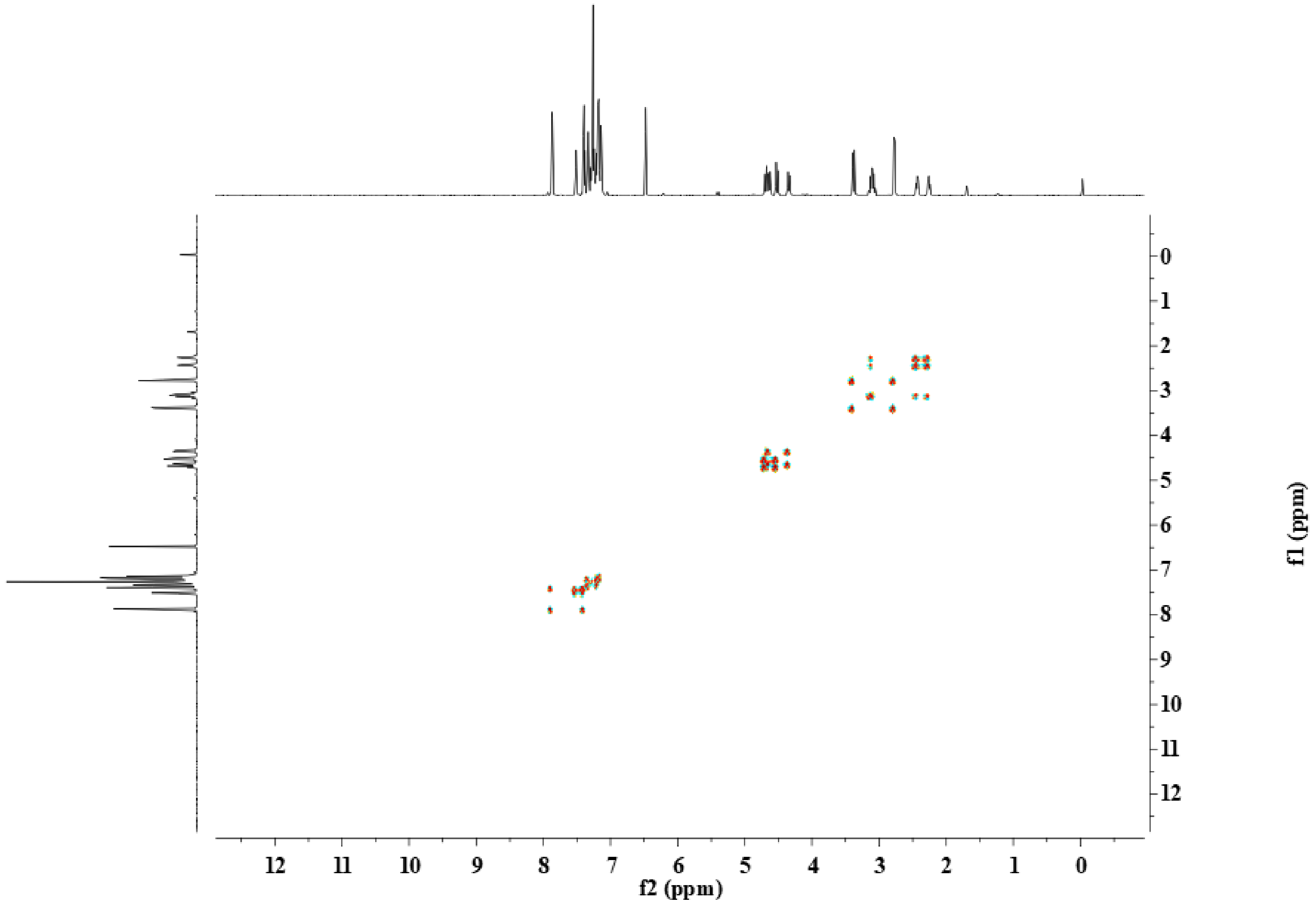

Supplementary Fig. 232 ^{1}H-H COSY - of **6e** (600 MHz, $CDCl_3$)

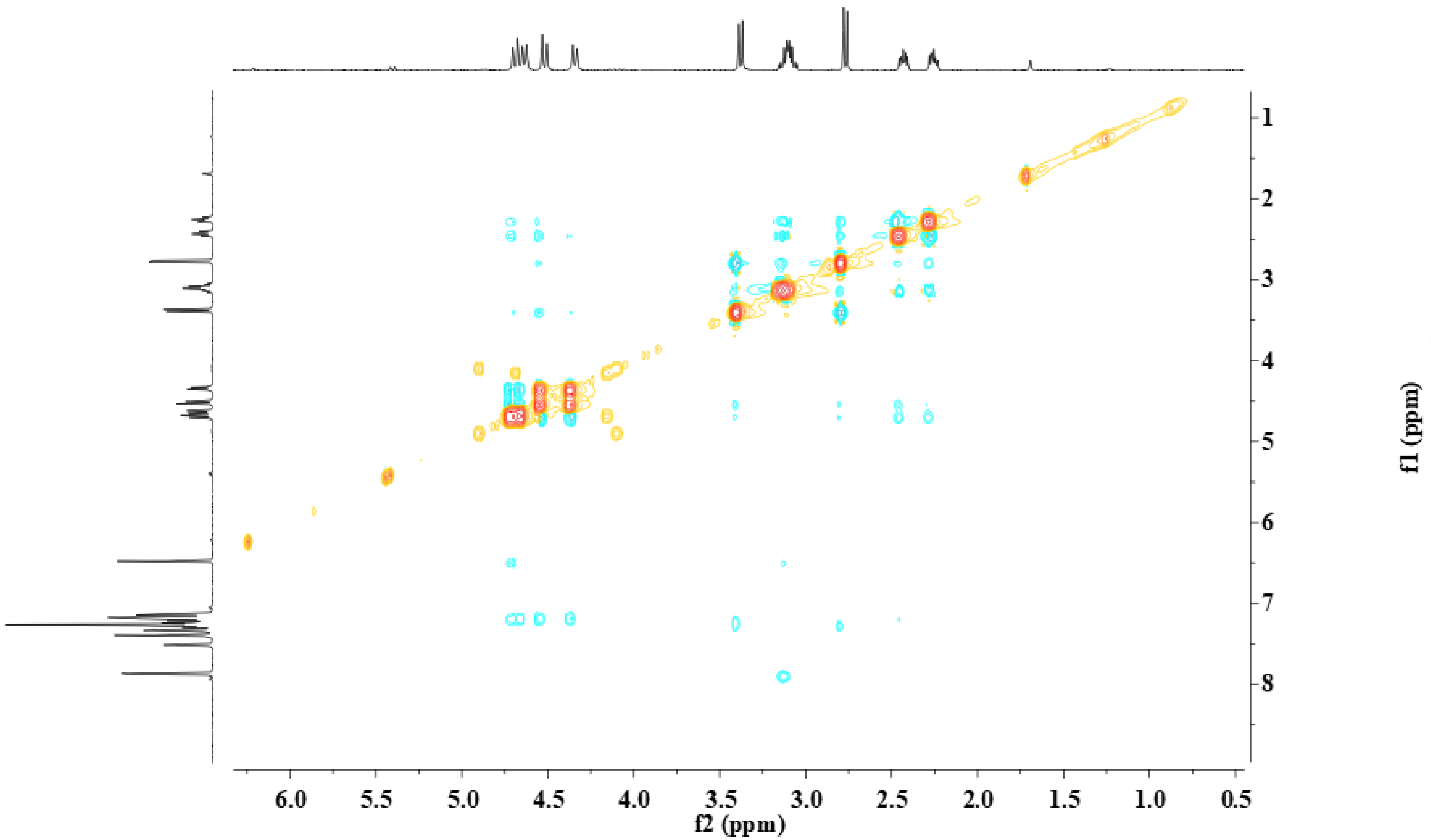

Supplementary Fig. 233 NOESY - 2D ^{1}H NMR of **6e** (600 MHz, $CDCl_3$)

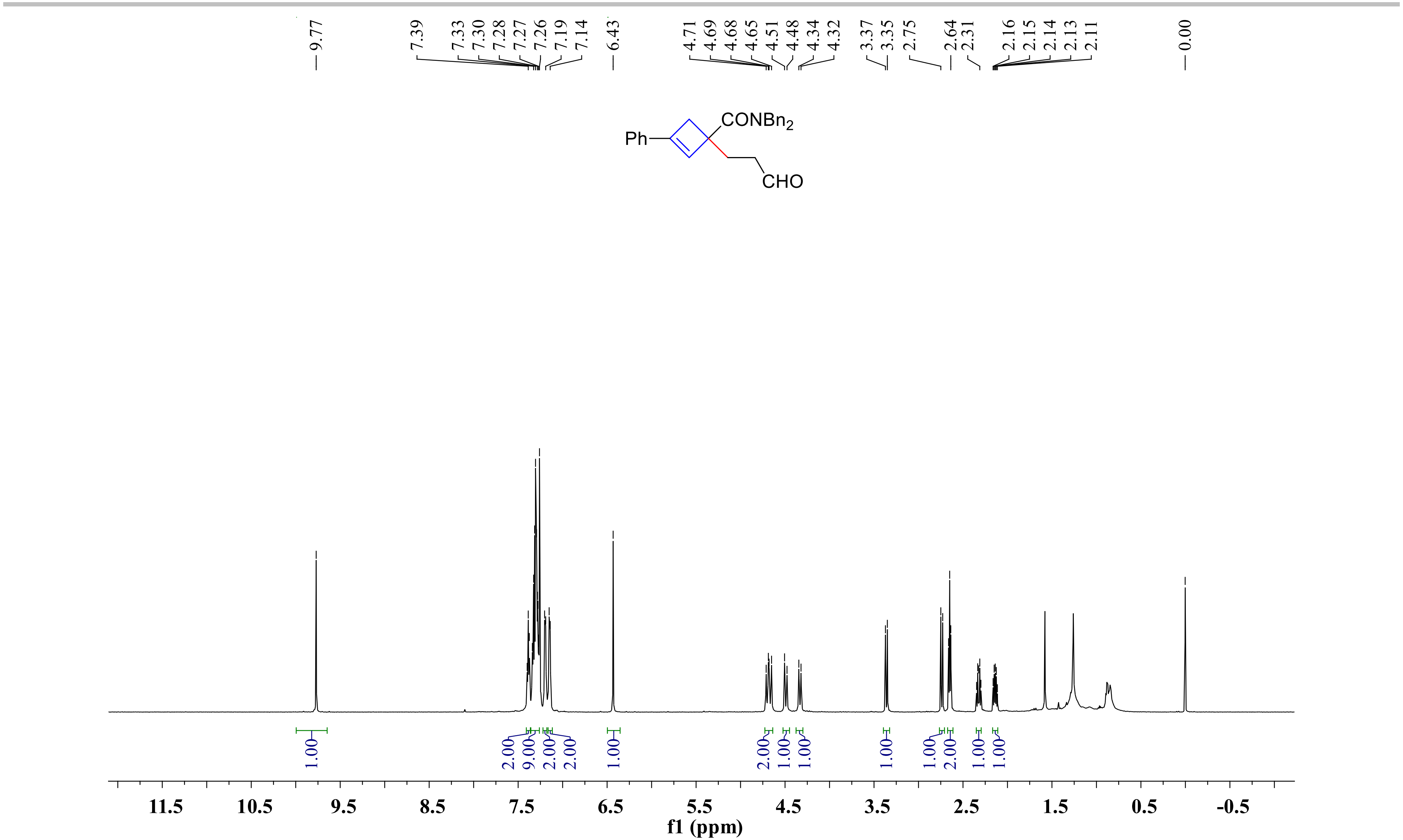

Supplementary Fig. 234 ^{1}H NMR (600 MHz, $CDCl_3$) of **6f**

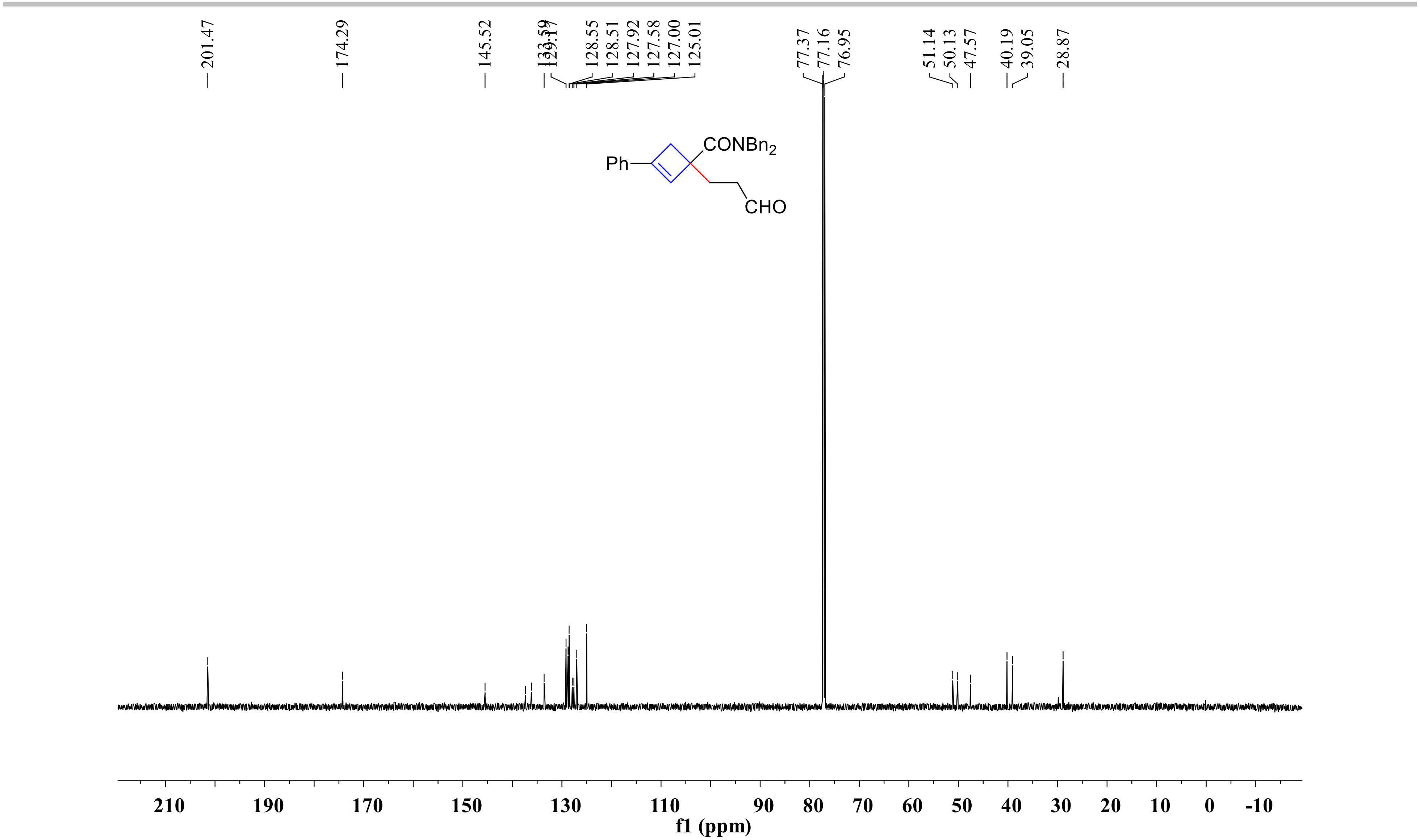

Supplementary Fig. 235 ^{13}C NMR (151 MHz, $CDCl_3$) of **6f**

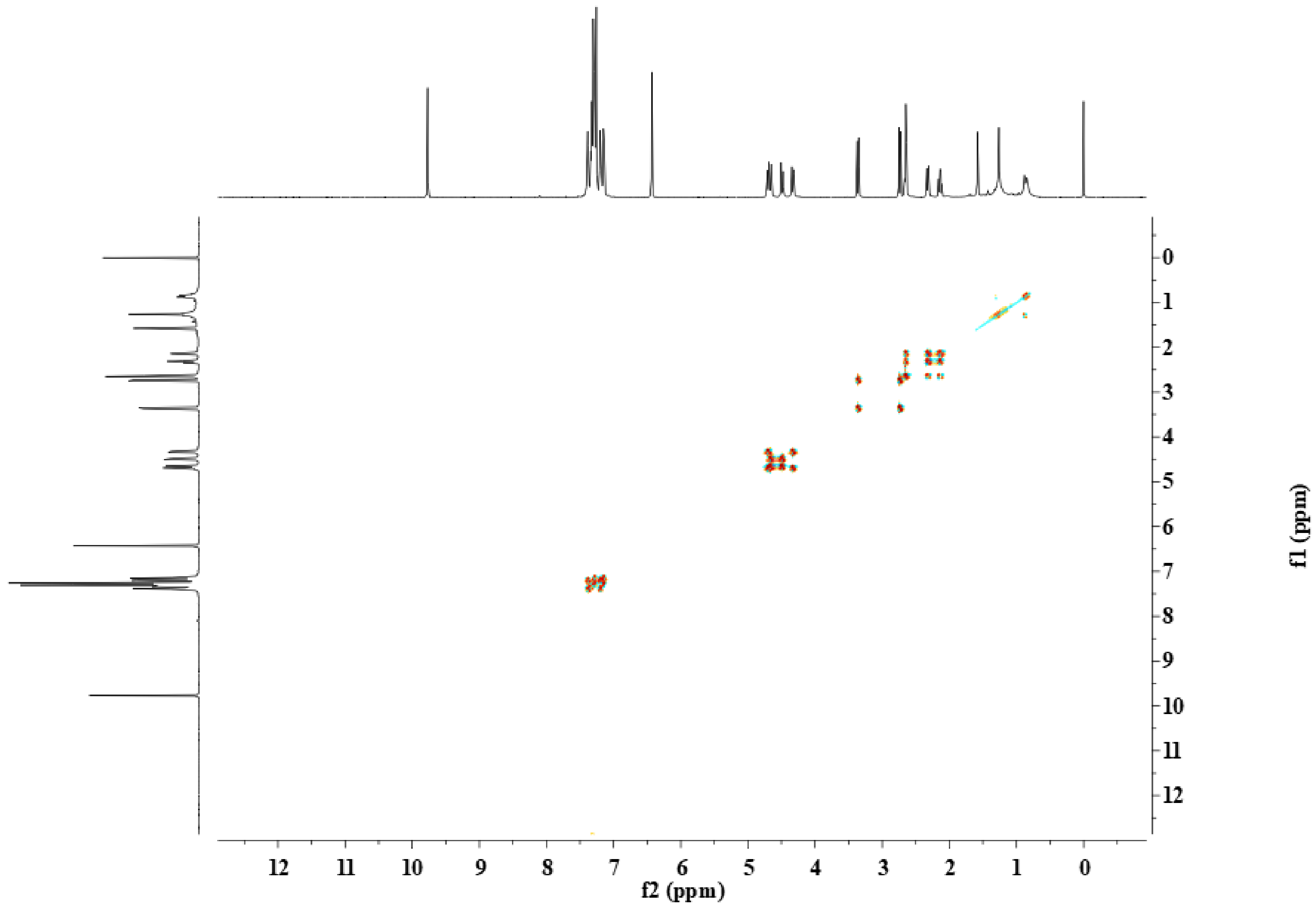

Supplementary Fig. 236 H-H COSY of **6f** (600 MHz, $CDCl_3$)

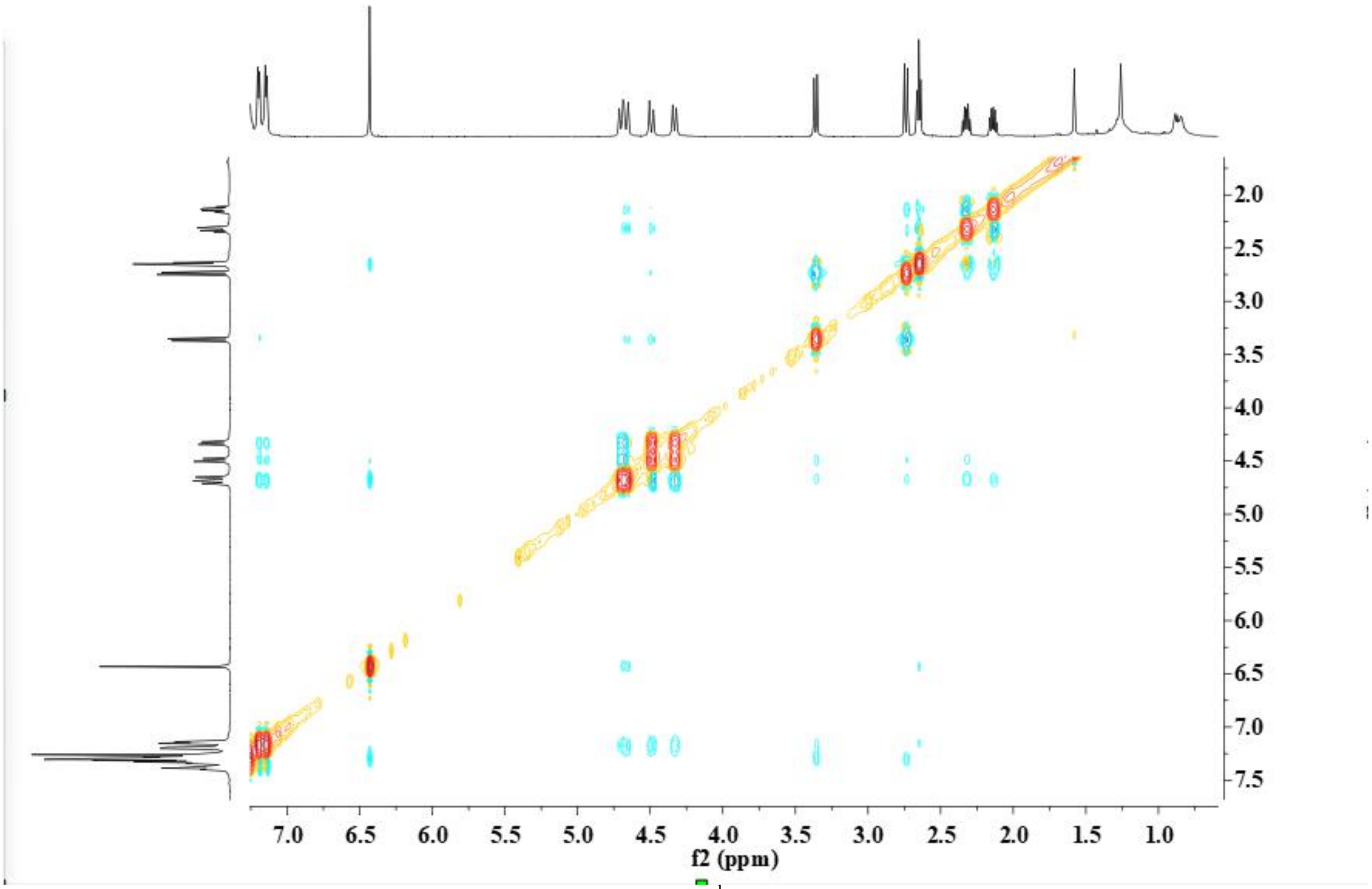

Supplementary Fig. 237 NOESY - 2D ^{1}H NMR of **6f** (600 MHz, $CDCl_3$)

4. Supplementary References

(1) (a) Binder, -J.; Fischer, R. -C.; Flock, M.; Torvisco, A.; Uhlig, F. *Phosphorus, Sulfur and Silicon and the Related Elements*. **2015**, *190*, 1980-1993. (b) Binder, J.; Fischer, R. -C.; Flock, M.; Stammler, H. –G.; Torvisco, A.; Uhlig, F. *Phosphorus, Sulfur and Silicon and the Related Elements.* **2016**, *191*, 478-487.

(2) Jung, M.; Lindsay, V. N. G. One-Pot Synthesis of Strain-Release Reagents from Methyl Sulfones. *J. Am. Chem. Soc.* **2022**, *144*, 4764−4769.

(3) (a) Zhou, R.; Wang, J.-F.; Song, H.-B.; He, Z.-J. Phosphine-Catalyzed Cascade [3 + 2] Cyclization−Allylic Alkylation, [2 + 2 + 1] Annulation, and [3 + 2] Cyclization Reactions between Allylic Carbonates and Enones. *Org. Lett*. **2011**, *13*, 580–583. (b) Pian, J.-X.; Chen, Q.-Q.; Luo, Y.-J.; Zhao, Z.-F.; Liu, J.-C.; He, L.; Li, S.-W. Asymmetric Synthesis of Chiral Cyclopropanes from Sulfoxonium Ylides Catalyzed by a Chiral-at-Metal Rh(III) Complex. *Org. Lett.* **2022**, *24*, 5641–5645.

(4) Frisch, M. J.; Trucks, G. W.; Schlegel, H. B.; Scuseria, G. E.; Robb, M. A.; Cheeseman, J. R.; Scalmani, G.; Barone, V.; Petersson, G. A.; Nakatsuji, H.; Li, X.; Caricato, M.; Marenich, A. V.; Bloino, J.; Janesko, B. G.; Gomperts, R.; Mennucci, B.; Hratchian, H. P.; Ortiz, J. V.; Izmaylov, A. F.; Sonnenberg, J. L.; Williams; Ding, F.; Lipparini, F.; Egidi, F.; Goings, J.; Peng, B.; Petrone, A.; Henderson, T.; Ranasinghe, D.; Zakrzewski, V. G.; Gao, J.; Rega, N.; Zheng, G.; Liang, W.; Hada, M.; Ehara, M.; Toyota, K.; Fukuda, R.; Hasegawa, J.; Ishida, M.; Nakajima, T.; Honda, Y.; Kitao, O.; Nakai, H.; Vreven, T.; Throssell, K.; Montgomery Jr, J. A.; Peralta, J. E.; Ogliaro, F.; Bearpark, M. J.; Heyd, J. J.; Brothers, E. N.; Kudin, K. N.; Staroverov, V. N.; Keith, T. A.; Kobayashi, R.; Normand, J.; Raghavachari, K.; Rendell, A. P.; Burant, J. C.; Iyengar, S. S.; Tomasi, J.; Cossi, M.; Millam, J. M.; Klene, M.; Adamo, C.; Cammi, R.; Ochterski, J. W.; Martin, R. L.; Morokuma, K.; Farkas, O.; Foresman, J. B.; Fox, D. J. *Gaussian 09*, Revision B.01; Gaussian, Inc.: Wallingford, CT, 2010.

(5) Zhao, Y.; Truhlar, D. G., The M06 suite of density functionals for main group thermochemistry, thermochemical kinetics, noncovalent interactions, excited states, and transition elements: two new functionals and systematic testing of four M06-class functionals and 12 other functionals. *Theoretical Chemistry Accounts* **2008,** *120*, 215-241.

(6) Weigend, F.; Ahlrichs, R., Balanced basis sets of split valence, triple zeta valence and quadruple zeta valence quality for H to Rn: Design and assessment of accuracy. *Physical Chemistry Chemical Physics* **2005,** *7*, 3297-3305.

(7) Marenich, A. V.; Cramer, C. J.; Truhlar, D. G., Universal Solvation Model Based on Solute Electron Density and on a Continuum Model of the Solvent Defined by the Bulk Dielectric Constant and Atomic Surface Tensions. *The Journal of Physical Chemistry B* **2009,** *113*, 6378-6396.

(8) (a) Zheng, J.; Xu, X.; Truhlar, D. G., Minimally augmented Karlsruhe basis sets. *Theoretical Chemistry Accounts* **2011,** *128*, 295-305; (b) Papajak, E.; Zheng, J.; Xu, X.; Leverentz, H. R.; Truhlar, D. G.,

Perspectives on Basis Sets Beautiful: Seasonal Plantings of Diffuse Basis Functions. *Journal of Chemical Theory and Computation* **2011,** *7*, 3027-3034.
